# Three new species of arbuscular mycorrhizal fungi (Glomeromycota) and *Acaulospora gedanensis* revised

**DOI:** 10.3389/fmicb.2024.1320014

**Published:** 2024-02-12

**Authors:** Piotr Niezgoda, Janusz Błaszkowski, Tomasz Błaszkowski, Anna Stanisławczyk, Szymon Zubek, Paweł Milczarski, Ryszard Malinowski, Edward Meller, Monika Malicka, Bruno Tomio Goto, Sylwia Uszok, Leonardo Casieri, Franco Magurno

**Affiliations:** ^1^Department of Environmental Management, West Pomeranian University of Technology in Szczecin, Szczecin, Poland; ^2^Department of General and Oncological Surgery, Pomeranian Medical University in Szczecin, Szczecin, Poland; ^3^Department of Genetics, West Pomeranian University of Technology in Szczecin, Szczecin, Poland; ^4^Institute of Botany, Faculty of Biology, Jagiellonian University, Krakow, Poland; ^5^Department of Genetic, Plant Breeding & Biotechnology, West Pomeranian University of Technology in Szczecin, Szczecin, Poland; ^6^Institute of Biology, Biotechnology and Environmental Protection, Faculty of Natural Sciences, University of Silesia in Katowice, Katowice, Poland; ^7^Departamento de Botânica e Zoologia, Universidade Federal do Rio Grande do Norte, Natal, Brazil; ^8^Mycorrhizal Applications LLC at Bio-Research & Development Growth Park, St. Louis, MO, United States

**Keywords:** arbuscular mycorrhizal fungi, Glomeromycota, new and emended taxa, morphology, nuc rDNA, phylogenetic taxonomy, *rpb1*

## Abstract

Studies of the morphology and the 45S nuc rDNA phylogeny of three potentially undescribed arbuscular mycorrhizal fungi (phylum Glomeromycota) grown in cultures showed that one of these fungi is a new species of the genus *Diversispora* in the family Diversisporaceae; the other two fungi are new *Scutellospora* species in Scutellosporaceae. *Diversispora vistulana* sp. nov. came from maritime sand dunes of the Vistula Spit in northern Poland, and *S. graeca* sp. nov. and *S. intraundulata* sp. nov. originally inhabited the Mediterranean dunes of the Peloponnese Peninsula, Greece. In addition, the morphological description of spores of *Acaulospora gedanensis*, originally described in 1988, was emended based on newly found specimens, and the so far unknown phylogeny of this species was determined. The phylogenetic analyses of 45S sequences placed this species among *Acaulospora* species with atypical phenotypic and histochemical features of components of the two inner germinal walls.

## Introduction

The order Diversisporales was originally created in the newly described phylum Glomeromycota (Schüßler et al., 2001) based on the phylogenetic analyses of the nuclear SSU rDNA gene sequences of members belonging to the *Glomus* group C sensu Schwarzott et al. (2001). The order included Diversisporaceae fam. ined., as well as the members of the families Acaulosporaceae and Gigasporaceae—both sensu Morton and Benny (1990), the former with the genera *Acaulospora* and *Entrophospora*, and the latter with *Gigaspora* and *Scutellospora*. Walker and Schüßler (2004) validated Diversisporaceae as a new family with the type genus and species *Diversispora* and *D. spurca*, respectively, originally described as *Glomus spurcum* (Pfeiffer et al., 1996). Then, two new families (Pacisporaceae, Sacculosporaceae) and nine new genera (*Corymbiglomus, Desertispora, Kuklospora, Otospora, Pacispora, Redeckera, Sacculospora, Sieverdingia, Tricispora*) were introduced into Diversisporales (Oehl and Sieverding, 2004; Walker and Schüßler, 2004; Sieverding and Oehl, 2006; Palenzuela et al., 2008; Schüßler and Walker, 2010; Oehl et al., 2011c; Błaszkowski, 2012; Symanczik et al., 2018; Błaszkowski et al., 2019a). While *Kuklospora* was invalidated (Kaonongbua et al., 2010), *Corymbiglomus, Otospora, Sacculospora*, and *Tricispora* were identified as genera requiring revision (Redecker et al., 2013); *Desertispora* and *Sieverdingia* are yet to be evaluated by experts of this group of fungi. In the meantime, Oehl et al. (2008) transferred known *Scutellospora* species into three new families (Dentiscutataceae, Racocetraceae, Scutellosporaceae) with five new genera (*Cetraspora, Dentiscutata, Fuscutata, Racocetra, Quatunica*), retaining in Gigasporaceae only *Gigaspora*. However, Morton and Msiska (2010), based on a larger number of taxa and congruence between morphological and molecular characters, rejected most of the families. Later, all the taxa and the genus *Orbispora* sensu Oehl et al. (2011a) were transferred to Gigasporales or. nov. (Oehl et al., 2011b), into which Goto et al. (2012) introduced a new family, Intraornatosporaceae, with the new genera *Intraornatospora* and *Paradentiscutata*, and Marinho et al. (2014) introduced *Bulbospora* gen. nov. in Scutellosporaceae. Redecker et al. (2013) classified all arbuscular mycorrhizal fungi (AMF) forming spores from a bulbous sporogenous cell (i.e., gigasporoid spores) in one family, Gigasporaceae, consisting of the genera *Cetraspora, Dentiscutata, Gigaspora, Scutellospora, Intraornatospora*, and *Paradentiscutata*, of which the latter two were considered as orphan taxa.

Since the genus *Acaulospora* (Gerdemann and Trappe, 1974) was described, there has been a significant increase in the knowledge about the characteristics of the members belonging to Diversisporales. Yet, identifying and classifying this group of fungi remains difficult or impossible for the following main reasons. First, the morphological characters proposed to distinguish taxa at the several taxonomic ranks are not always diagnostic. For example, *Cetraspora* species may be easily confused with, e.g., *Scutellospora* species because the number of spore walls and characters of the germination shields overlap (da Silva et al., 2012). Second, the subcellular structure of spores of many genera is very simple and similar or identical across different species (Morton, 1993). Moreover, the phenotypic and histochemical features of the spore wall components are highly variable and often invisible in field-collected and aged spores from trap cultures due to sloughing off (Morton, 1993). Third, approximately 23% of described species do not have known phylogeny. Fourth, growing many AMF in culture, especially those producing acaulo- and gigasporoid spores, is difficult or prone to failure. This makes it difficult to recognize their morphology, ontogeny, and phylogeny. Finally, DNA extracted from field-collected spores is usually of low quality and/or of uncertain provenance (Redecker et al., 2013).

About half of the phylogenetically characterized species of Diversisporales (approximately 48%) have been characterized molecularly with sequences comprising the 18S (partial), ITS1-5.8S-ITS2, and 28S (partial) nuc rDNA segment (=45S), which generally separates even closely related species (Krüger et al., 2012; Błaszkowski et al., 2019b). However, only the 28S sequences from a curated database, as established by Delavaux et al. (2021, 2022), can be used as a marker to separate closely related species in Glomeromycota. In addition, a large proportion of glomoid spore-producing species of this order have partial sequences of the protein-coding largest subunit of the RNA polymerase II (*rpb1*) gene. Compared to 45S sequences, the taxonomic resolution of the *rpb1* sequences is higher, and their use reduces the risk of creating potential phylogenetic artifacts due to paralogous sequences because *rpb1* is a single-copy gene in arbuscular mycorrhizal fungi (Stockinger et al., 2014). Other single-copy marker loci recommended for use in reconstructing AMF phylogenies are the ß*-tubulin* and glomalin genes (Corradi et al., 2004a,b; Msiska and Morton, 2009; Magurno et al., 2019; Kokkoris et al., 2023). However, lower resolution and poor support at the order level of the ß*-tubulin* phylogenies, compared to those reconstructed from SSU, LSU, and *rpb1* sequences (Msiska and Morton, 2009) and the relatively small number of sequences for the AMF glomalin gene (Magurno et al., 2019; Kokkoris et al., 2023), have made these two protein-coding loci of little use in taxonomic analyses of large numbers of Glomeromycota representatives.

It is now strongly recommended to reconstruct fungal phylogenies based on sequences derived from unlinked loci, including those encoding a protein (Chethana et al., 2021). Unfortunately, the vast majority of species of Diversisporales producing spores other than glomoid (ca. 68%), are still missing *rpb1* sequences, except for two *Acaulospora* species (out of 60 described), three *Gigaspora* species (9), and five species with gigasporoid spores (31). Due to the small number of mycologists involved in collecting and characterizing AMF (morphological and molecular), *rpb1* sequences for most non-glomoid spore-producing species are not expected to be obtained even in the distant future; overall, out of 278 *rpb1* sequences available in the GenBank for Glomeromycota, approximately 68% were obtained by J. Błaszkowski and his collaborators.

One of the relatively “old” non-sequenced members of Diversisporales is *A. gedanensis*, which was classified in *Acaulospora* because it formed acaulosporoid spores (Błaszkowski, 1988). However, the presence of only one germinal wall in the spore subcellular structure and the atypical phenotypic and histochemical properties of layers sensu Morton et al. (1995) of this wall (Błaszkowski, 1988) clearly separated this species from all previously described *Acaulospora* species. The emended definition of *A. gedanensis* informed about the presence of two germinal walls, typical of *Acaulospora* species. However, the rigidity and fragility of the one-layered thin germinal wall 1, the lack of a beaded ornamentation on the upper surface of germinal wall 2 layer 1, and the non-plasticity and non-reactivity of germinal wall 2 layer 2 in PVLG and Melzer's reagent, respectively, suggested this species belonged to the genus *Ambispora* (Błaszkowski, 2012) in the family Ambisporaceae (Archaeosporales). We grew *A. gedanensis* in culture, which allowed us to re-analyze the morphology of this species and carry out phylogenetic analyses to establish its position in the Glomeromycota phylogeny.

This study had the following aims: (i) to reconstruct the phylogenies of three potentially new species in Glomeromycota, to characterize their morphologies and compare them with those of the phylogenetically most closely related species; (ii) to determine the phylogeny of *A. gedanensis*, show morphological differences between this species and its closest relatives; and (iii) to calculate the genetic distance of the three potentially new species and *A. gedanensis* from their most closely related species.

## Materials and methods

### Origin of AMF isolates

The potentially new species were initially named *Diversispora 448, Scutellospora 431*, and *Scutellospora 437* (numbers are from an AMF database maintained by J. Błaszkowski). These isolates, as well as *A. gedanensis*, were characterized based on spores extracted from trap cultures because numerous attempts to grow the fungi in single-species cultures failed. The trap cultures were established from field mixtures of rhizosphere soils and root fragments collected under the following plant species. The field host of *Diversispora 448* was *Ammophila arenaria* (L.) Link. The plant colonized maritime sand dunes near Przebrno (54°22'30.3''N 19°23'13.6''E) located on the Vistula Spit in northern Poland. The soil sample was collected by P. Niezgoda on July 10, 2020. The data relating to the climatic and soil chemical properties of Vistula Spit are detailed in Błaszkowski et al. (2002). *Scutellospora 431* and *Scutellospora 437* were hosted in the field by *A. arenaria*, which colonized maritime sand dunes of the Voidokoilia beach (36°57'46''N 21°39'45''E) located on the Peloponnese peninsula, Greece. The soil sample was collected by J. Błaszkowski on September 8, 2015. Data relating to the climate and soil chemical properties of the sampled site are detailed in Błaszkowski et al. (2019a). In the field, *A. gedanensis* was associated with the roots of *Festuca rubra* L. growing among trees inhabiting a moist site located near Władysławowo (54°47'00''N 18°26'17''E) on the Hel Peninsula in northern Poland. The host plant was sampled by J. Błaszkowski on July 18, 2022. The trap and single-species cultures were established and grown, spores extracted, and mycorrhizal structures stained, as described in Błaszkowski et al. (2006, 2009). In all the attempts aimed at establishing single-species cultures, 5–15 spores of each isolate were used.

### Microscopy and nomenclature

Morphological features of spores, as well as phenotypic and histochemical characters of spore wall and germinal wall layers of *A. gedanensis, Diversispora 448, Scutellospora 431*, and *Scutellospora 437* were characterized based on at least 50–100 spores of each isolate mounted in water, lactic acid, polyvinyl alcohol/lactic acid/glycerol (PVLG, Omar et al., 1979), and a mixture of PVLG and Melzer's reagent (1:1, v/v). Spores for study and photography were prepared as described in Błaszkowski (2012) and Błaszkowski et al. (2012). The types of spore wall and germinal wall layers have been defined by Walker (1983), Morton (1986), Morton et al. (1995), and Błaszkowski (2012). Color names were obtained from Kornerup and Wanscher (1983). The nomenclature of fungi and the authors of fungal names were taken from the Index Fungorum website (http://www.indexfungorum.org/AuthorsOfFungalNames.htm). The term “glomerospores” was used for spores produced by AMF, as proposed by Goto and Maia (2006).

The voucher specimens of the proposed new species [spores permanently mounted in PVLG and a mixture of PVLG and Melzer's reagent (1:1, v/v) on slides] were deposited at ZT Myc (ETH Zurich, Switzerland; holotypes) and in the Laboratory of Plant Protection, Department of Environmental Management (LPPDEM), West Pomeranian University of Technology in Szczecin, Poland (isotypes and *A. gedanensis* specimens).

### DNA extraction, PCR, cloning, and DNA sequencing

Genomic DNA of *A. gedanensis, Diversispora 448, Scutellospora 431*, and *Scutellospora 437* was extracted from single or 2–5 spores. The method of processing the spores prior to PCR, conditions and primers used for PCR, as well as cloning and sequencing of PCR products to obtain 45S sequences of the four isolates were as those described by Błaszkowski et al. (2021). A nested PCR with the primers RPB1-4F2/RPB1-5R1 and RPB1-4F3/RPB1-5R2 was used to obtain *rpb1* sequences from *Diversispora 448* like in Błaszkowski et al. (2022). Both 45S and *rpb1* sequences were deposited in GenBank (OR669295, OR669296, OR669014–OR669021, OR669025–OR669038).

### Phylogenetic analyses

To confirm the BLAST results indicating the analyzed AMF as undescribed species of Diversisporaceae (*Diversispora 448*) and Scutellosporaceae (*Scutellospora 431, Scutellospora 437*), as well as to determine the positions of these isolates within these families and *A. gedanensis* among sequenced *Acaulospora* species, five alignments were prepared. The phylogeny of *Diversispora 448* was reconstructed based on the analyses of the 45S and *rpb1* sequences, while the phylogenies of the other isolates were reconstructed from the 45S sequences alone. The 45S and *rpb1* sequence sets were aligned separately in MAFFT 7 with the E-INS-i option.

For *Diversispora 448*, the ingroup of the 45S alignment consisted of 85 sequences of 22 *Diversispora* species and *Diversispora 448*, and the outgroup was *Corymbiglomus corymbiforme, Desertispora omaniana, Redeckera megalocarpum*, and *Sieverdingia tortuosa*. The species of the outgroup represented the most closely related genera to *Diversispora* selected through prior analyses of members of all genera of Diversisporaceae. The 45S alignment with *Scutellospora 431* and *Scutellospora 437* contained 28 sequences of 6 *Scutellospora* species and the 2 potentially new species in the ingroup and *Orbispora pernambucana* in the outgroup. The ingroup of the *A. gedanensis* 45S alignment counted 118 sequences, representing 48 *Acaulospora* species, and the outgroup included *Sacculospora baltica* and *S. felinovii* sequences.

The *Diversispora 448 rpb1* alignment included 36 *rpb1* sequences that characterized 18 *Diversispora* species of the *Diversispora 448* 45S alignment and *Diversispora 448*, i.e., all *Diversispora* species having sequences of the two loci. The *Diversispora 448* 45S+*rpb1* alignment was created by concatenation of the 45S and *rpb1* alignments.

The percentage genetic distances of *A. gedanensis, Diversispora 448, Scutellospora 431*, and *Scutellospora 437* from their closest relatives were calculated in BioEdit (Hall, 1999). All comparisons were performed on sequences of the same length.

The phylogenetic positions of *A. gedanensis, Diversispora 448, Scutellospora 431*, and *Scutellospora 437* were reconstructed based on Bayesian inference (BI) and maximum likelihood (ML) phylogenetic analyses of the 45S and 45S+*rpb1* alignments, performed via CIPRES Science Gateway 3.1 (Miller et al., 2010). The 45S and *rpb1* alignments were divided into five and three partitions, respectively (45S into 18S, ITS1, 5.8S, ITS2, and 28S; *rpb1* into two exons and one intron). In both BI and ML analyses, the nucleotide substitution model GTR+I+G was used for each nucleotide partition (Abadi et al., 2019).

The BI reconstruction was done in MrBayes 3.2 (Ronquist et al., 2012) based on four Markov chains run over one million generations, sampling every 1,000 generations with a burn-in at 30% of sampled trees. The ML phylogenetic tree inference was performed with RAxML-NG 1.0.1 (Kozlov et al., 2019), using a maximum likelihood/1000 bootstrapping run and ML estimated proportion of invariable sites and base frequencies. The alignments and tree files are available as online resources (Supplementary Tables 2–13).

Clades were considered supported when the Bayesian posterior probabilities and the ML bootstrap values were ≥0.95 and ≥70%, respectively. The phylogenetic trees were visualized and edited in MEGA6 (Tamura et al., 2013).

The occurrence of *A. gedanensis, Diversispora 448, Scutellospora 431*, and *Scutellospora 437* in other ecosystems worldwide was checked by comparing 45S sequences of these species/isolates with environmental sequences deposited in GenBank and retrieved using BLASTn. It was assumed that the sequences shown could represent the same species/isolates as the four AMF discussed here when their similarity was >96%.

## Results

### General data and phylogeny

Of the 45S and *rpb1* sequences analyzed, 22 and 2, respectively, were newly obtained in this study. The numbers of analyzed sequences and species/isolates, as well as the numbers of base pairs, variable, and parsimony informative sites of each of the alignments studied are presented in Supplementary Table 1.

**Figure 1:**
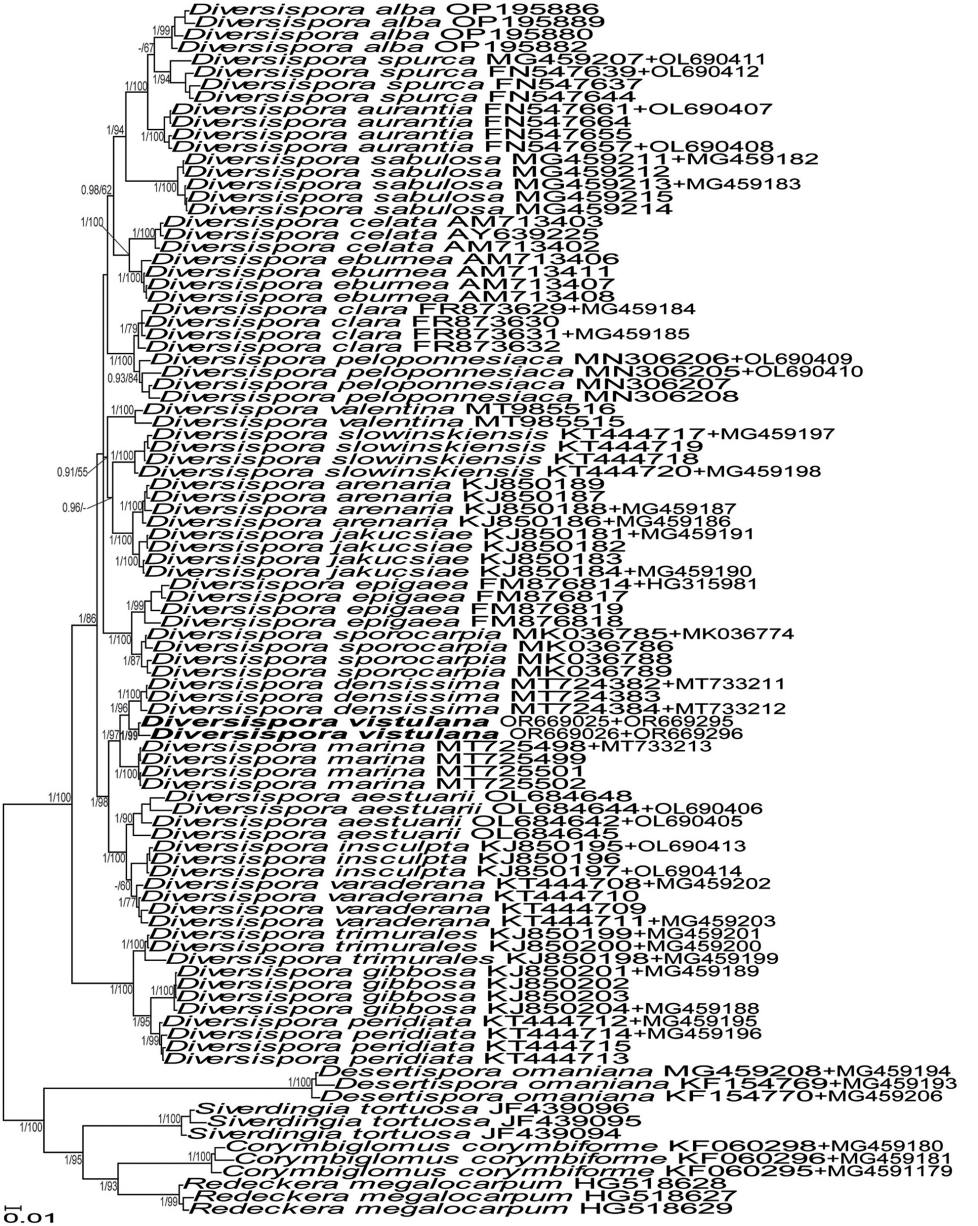
50% majority-rule consensus tree from the Bayesian analysis of sequences of 45S nuc rDNA concatenated with *rpb1* sequences of *Diversispora vistulana*, 22 other *Diversispora* species, as well as *Corymbiglomus corymbiforme, Desertispora omaniana, Redeckera megalocarpum*, and *Sieverdingia tortuosa* serving as outgroup. The new species is in bold font. The Bayesian posterior probabilities ≥0.90 and ML bootstrap values ≥50% are shown near the branches. The bar indicates 0.01 expected change per site per branch.

The topologies of the 45S and 45S+*rpb1* trees generated by BI and ML analyses of sequences of *Acaulospora, Diversispora*, and *Scutellospora* members were identical (Figures 1, 3, 7, and Supplementary Figure S1). In the trees with 45S+*rpb1* and 45S sequences (Figure 1, Supplementary Figure S1), *Diversispora 448* grouped in a clade sister to *D. densissima* sequences, and in both these trees, the *Diversispora 448* clade obtained full BI (=1.0) and very high ML (=99% and 97%, respectively) supports. Analyses of 45S sequences of *Scutellospora 431* and *Scutellospora 437* indicated that they were sister species and closely related to *Scutellospora ovalis*. Each of the potentially new species obtained full BI (=1.0) and full (=100% for *Scutellospora 437*) or high (=84% for *Scutellospora 431*) ML supports (Figure 3).

The divergence of 45S sequences of *Diversispora 448* from those of *D. densissima* ranged from 5.0% to 6.7%. The range of differences between 45S sequences of *Scutellospora 431* and *Scutellospora 437* was 3.6–5.3%.

*Acaulospora gedanensis* was placed in a basal clade sister to a clade consisting of two subclades, one with *A. brasiliensis* and the second with *A. pustulata* (Figure 7). The *A. gedanensis* clade was fully (BI =1.0) and strongly (ML = 89%) supported. Also, the clades with *A. brasiliensis* and *A. pustulata*, as well as the node linking the *A. gedanensis* clade with the *A. brasiliensis* and the *A. pustulata* clade obtained full BI and very high ML (98%−100%) supports.

*Acaulospora gedanensis* and *A. brasiliensis* differed in terms of the nucleotide composition of the 45S sequences by 3.2%−7.4% and *A. gedanensis* and *A. pustulata* by 3.4%−4.4%.

### Taxonomy

The results of phylogenetic analyses and comparisons of sequences described above confirmed our supposition resulting from the preliminary morphological and molecular studies of the three fungi considered here that they are undescribed species and showed the closest relatives of *A. gedanensis*. Consequently, *Diversispora 448, Scutellospora 431*, and *Scutellospora 437* have been described below as *D. vistulana* sp. nov., *S. graeca* sp. nov., and *S. intraundulata* sp. nov., respectively. As for *A. gedanensis*, its morphology was compared with the original one characterized by Błaszkowski (1988, 2012) and the morphology of the species closely related phylogenetically (Figure 3).

### Description of new species

***Diversispora vistulana*** Niezgoda, B.T. Goto, Magurno et Błaszk., **sp. nov**.

Figures 2A–H.

**Figure 2:**
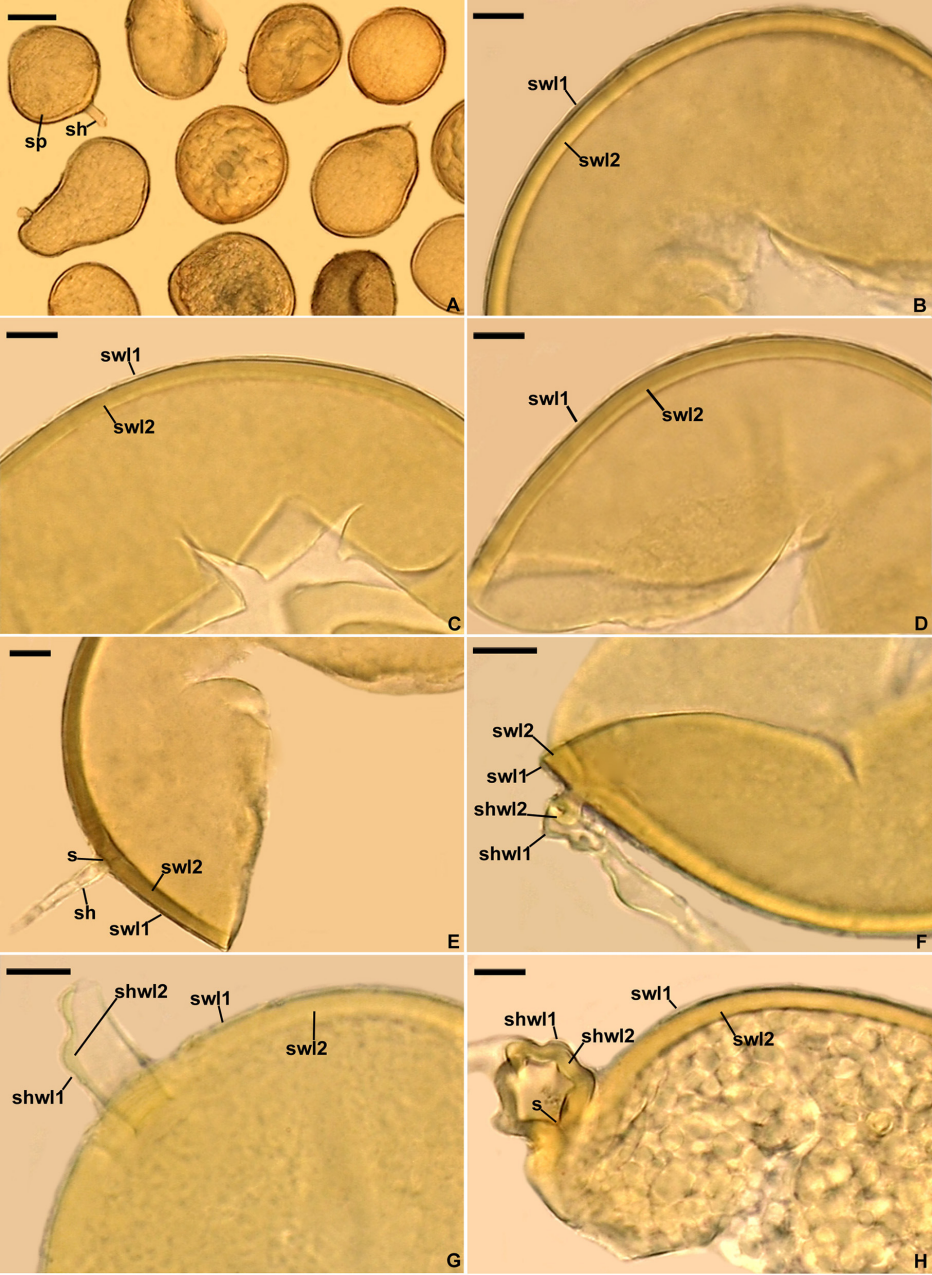
*Diversispora vistulana*. **(A)** Spores (sp) with subtending hyphae (sh). **(B–D)** Spore wall layers (swl) 1 and 2. **(E)** Spore wall layers (swl) 1 and 2, subtending hypha (sh), and septum (s) closing the space between the subtending hyphal lumen and the spore interior. **(F–H)** Subtending hyphal wall layers (shwl) 1 and 2 continuous with spore wall layers (swl) 1 and 2; septum (s) at the spore base is indicated in **(H)**. **(A)** Spores in lactic acid. **(B, C, E, H)** Spores in PVLG. **(D, F, G)** Spores in PVLG+Melzer's reagent. **(A–H)** Differential interference microscopy. Scale bars: **(A)** = 50 μm, **(B–H)** = 10 μm.

MycoBank No. MB 851412.

*Etymology*: Latin, *vistulana*, referring to the Vistula Bar, where the new species was originally found.

*Specimens examined*: POLAND. Spores from a trap culture inoculated with a field-collected mixture of the rhizosphere soil and root fragments of *Ammophila arenaria* growing in maritime sand dunes near Przebrno (54°22'30.3''N 19°23'13.6''E) on the Vistula Spit in northern Poland, July 10, 2020, P. Niezgoda (**holotype** slide with spores no. ZTMyc 0067007, **isotype** slides with spores no. 3910–3917, LPPDSE).

*Diagnosis*: Differs from (A) *D. densissima*, the closest phylogenetic relative (Figure 1, Supplementary Figure S1) in (i) the color of spores and their subtending hyphae, (ii) the number of spore wall layers, (iii) morphometric features of the spore wall and the spore subtending hypha, and (iv) nucleotide composition of sequences of the 45S nuc rDNA region and the *rpb1* gene (see Discussion for details); (B) *D. insculpta, D. peridiata*, and *D. sabulosa*, forming yellow-colored spores with the spore wall consisting of two permanent layers—a hyaline outer layer and a colored laminate inner layer—in morphometric features of spores, the spore wall, and the spore subtending hypha (Błaszkowski, 2012; Błaszkowski et al., 2015; Symanczik et al., 2018), and (iv) the phylogenetic position in Diversisporaceae (Figure 1, Supplementary Figure S1).

*Description*: Glomerospores (= spores) formed singly in soil, arise blastically at the tips of sporogenous subtending hyphae (Figures 2A, E–H). *Spores* are pale yellow (3A4) to dark yellow (4C8); globose to subglobose; (93–)102(−110) μm in diameter; frequently ovoid; 87–103 × 103–154 μm; with one subtending hypha (Figures 2A–H). The *spore wall* is composed of two permanent layers (Figures 2B–H). Layer 1, forming the spore surface, is uniform (not containing visible sublayers), semi-rigid, hyaline, usually uniform in thickness, (0.8–)1.2(−1.8) μm thick, sometimes with slightly thinner and thicker segments, then slightly wavy when viewed in cross-section, rarely with a slightly deteriorated upper surface, and always tightly adherent to the upper surface of spore wall layer 2 (Figures 2B–H). Layer 2 is laminate, smooth, semi-rigid, pale yellow (3A4) to dark yellow (4C8), (2.6–)3.3(−4.4) μm thick, consisting of very thin (< 0.5 μm) laminae, and tightly adherent to and not separating from each other in even vigorously crushed spores (Figures 2B–H). None of spore wall layers 1 and 2 stains in Melzer's reagent (Figures 2D, F, G). *Subtending hypha* is hyaline to pale yellow (3A4); straight or recurved, cylindrical or slightly constricted at the spore base; (4.6–)11.1(−13.1) μm wide at the spore base (Figures 2E–G); rarely irregular (Figure 2H); and not braking in crushed spores. *The wall of subtending hypha* is hyaline; (1.8–)3.4(−6.4) μm thick at the spore base; and consists of two layers that are continuous with spore wall layers 1 and 2 (Figures 2F–H). *The pore* is (0.6–)2.8(−7.0) μm wide at the spore base and open or occluded by a straight or slightly curved septum which is continuous with some outermost laminae of spore wall layer 2 (Figures 2E, H). The spore content is a hyaline oily substance. *Germination* unknown.

*Ecology and distribution*: In the field, *D. vistulana* probably lived in arbuscular mycorrhizal symbiosis with roots of *A. arenaria* in maritime sand dunes near Przebrno (54°22'30,3''N 19°23'13,6''E) located on the Vistula Spit in northern Poland, but no molecular analyses were performed to confirm this supposition. Numerous attempts at growing this species in single-species cultures with *P. lanceolata* as the host plant failed. According to BLASTn search and our phylogenetic analyses, *D. vistulana* appears to have a disjunct distribution since only two sequences (JF439143 with 97.18% of identity and coverage = 100% from China and EU350809 with 97.36% of identity and coverage = 34% from Netherlands: Island Terschelling, Atlantic Coast) clustered in the clade with 45S sequences of the new species (data not shown).

***Scutellospora graeca*** Błaszk., Niezgoda, B.T. Goto & Magurno, **sp. nov**.

Figures 4A–H

MycoBank No. MB 851413

*Etymology*: Latin, *graeca*, referring to the country, Greece, in which the species was originally found.

*Specimens examined*: GREECE. Spores from a trap culture inoculated with a field-collected mixture of the rhizosphere soil and root fragments of *Ammophila arenaria* inhabiting a maritime sand dune site of the beach Voidokoilia (36°57'N 21°39'E), the Peloponnese Peninsula, Greece, September 8, 2015, J. Błaszkowski (**holotype** slide with spores no. ZTMyc 0067008, **isotype** slides with spores no. 3918–3932, LPPDSE).

**Figure 3:**
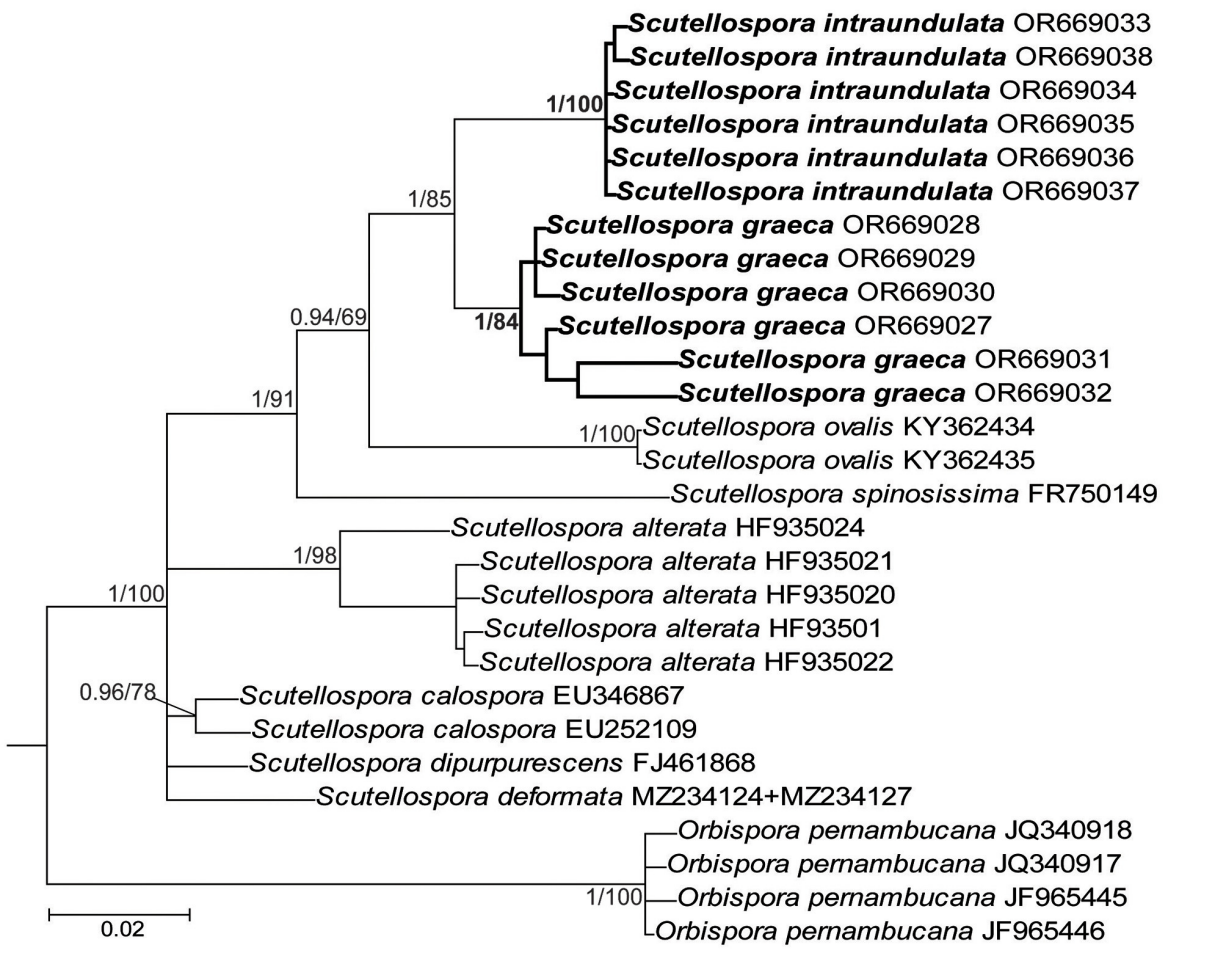
50% majority-rule consensus tree from the Bayesian analysis of sequences of 45S nuc rDNA of *Scutellospora graeca* and *S. intraundulata*, six other *Scutellospora* species, and *Orbispora pernambucana* serving as outgroup. The new species are in bold font. The Bayesian posterior probabilities ≥0.90 and ML bootstrap values ≥50% are shown near the branches. The bar indicates 0.02 expected change per site per branch.

*Diagnosis*: Differs from (A) *S. intraundulata*, the closest phylogenetic relative (Figure 3), in: (i) spore color, (ii) the thickness of the spore wall, (iii) having a laminate spore wall layer 2 that is uniform in thickness in its different regions (vs. often with local depressions on the lower surface), and (iv) nucleotide composition of sequences of the 45S nuc rDNA region (see Discussion for details); (B) other smooth-spored *Scutellospora* species, i.e., *S. arenicola, S. calospora, S. dipurpurescens*, and *S. deformata*, in (i) spore color and size, (ii) the permanence of spore wall layer 1 and the reactivity of spore wall layer 2 in Melzer's reagent, (iii) the thickness of spore wall layer 2, (iv) the composition and phenotypic and histochemical properties of germinal wall 1, (v) the composition of germinal wall 2 (Morton and Koske, 1988; Koske and Halvorson, 1989; Guillén et al., 2021; https://invam.ku.edu/species-descriptions), and (vi) the phylogenetic position among sequenced *Scutellospora* species (Figure 3).

**Figure 4:**
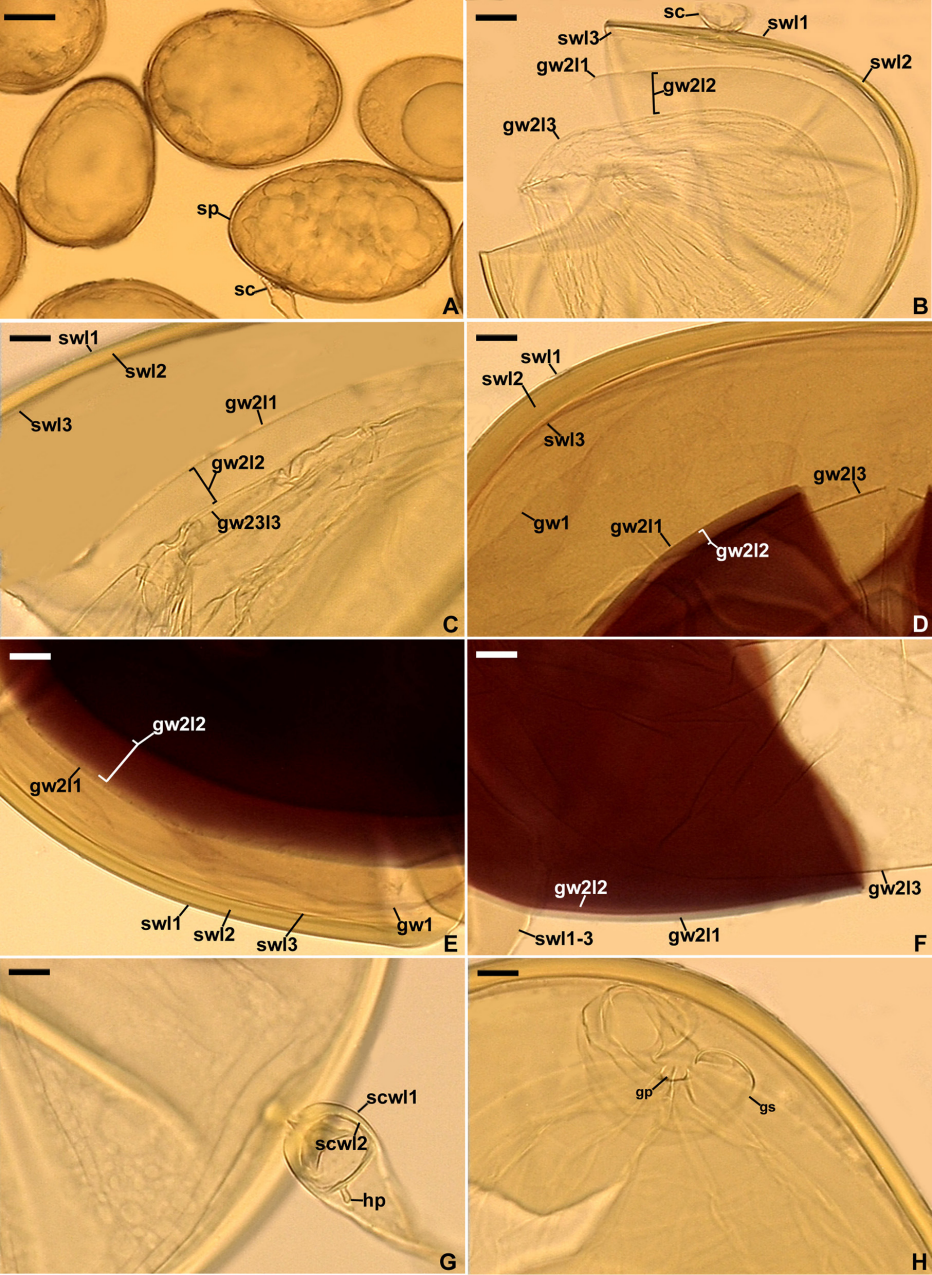
*Scutellospora graeca*. **(A)** Spores (sp) formed from the sporogenous cell (sc). **(B, C)** Layers 1–3 (l) of spore wall (sw) and germinal wall (gw) 2; gw 1 is invisible. **(D–F)** Layers (l) 1–3 of spore wall (sw) and germinal wall (gw) 2, and germinal wall 1. **(G)** Sporogenous cell wall layers (scwl) 1 and 2 and hyphal peg (hp). **(H)** Bi-lobed germination shield (gs) with germination pore (gp). **(A)** Spores in lactic acid. **(B, C, G, H)** Spores in PVLG. **(D–F)** Spores in PVLG+Melzer's reagent. **(A–H)** Differential interference microscopy. Scale bars: **(A)** = 50 μm, **(B–H)** = 10 μm.

*Description*: Glomerospores (= spores) formed singly in soil, terminally, rarely laterally, on bulbous sporogenous cells (Figures 4A, B, G). *Spores* are yellowish gray (3B2) to light yellow (4A4); ovoid; 109–162 × 164–228 μm; rarely globose; (140–)156(−168) μm in diameter; with a spore wall, two germinal walls, and a germination shield formed on the upper surface of germinal wall 2 (Figures 4A–H). The *spore wall* is composed of three permanent layers (spore wall layers 1–3; Figures 4B–F). Layer 1, forming the spore surface, is uniform, smooth, semi-rigid, hyaline, (1.0–)1.3(−1.5) μm thick, and always tightly adherent to layer 2 (Figures 4B–E). Layer 2 is laminate, smooth on the upper and lower surfaces, semi-rigid, yellowish gray (3B2) to light yellow (4A4), (2.8–)4.4(−7.0) μm thick, consisting of very thin (< 0.5 μm) laminae, tightly adherent to and not separating from each other in even vigorously crushed spores (Figures 4B–E). Layer 3 is uniform, smooth, hyaline to yellowish white (3A2), flexible to semi-flexible, (0.8–)1.0(−1.3) μm thick, usually slightly separating from the lower surface of layer 2 in even moderately crushed spores (Figures 4B, D, E), and staining reddish-white (10A2) to pale red (11A3) in Melzer's reagent (Figures 4D, E). *Germinal wall 1* consists of one layer that is flexible, hyaline, (0.8–)1.0(−1.3) μm thick, and usually difficult to detect in spores crushed in PVLG; generally, it is well visible in spores crushed in PVLG+Melzer's reagent where it stains reddish-white (10A2) to pale red (12A3) (Figures 4D, E). *Germinal wall 2* is composed of three smooth, hyaline layers (germinal wall 2 layers 1–3; Figures 4B–F). Layer 1 coriaceous sensu Walker (1986), (1.0–)2.4(−3.8) μm thick, is always inseparably covering layer 2 (Figures 4D–F), and is sometimes difficult to see in spores crushed in PVLG (Figures 4B, C) because of the lack of contrast with layer 2. Layer 2 is flexible, (1.3–)1.6(−1.8) μm thick in spores crushed in water, always plastic, amorphous sensu Morton (1986), strongly swelling, up to 48.0 μm thick, in spores crushed in lactic acid and PVLG (Figures 4B–F), and staining pastel pink (11A4) to violet brown (11F8) in Melzer's reagent (Figures 4D–F). Layer 3 is flexible, (0.8–)0.9(−1.0) μm thick, loosely associated with the lower surface of layer 2, and often pushed partly or completely out from under this layer in spores vigorously crushed in PVLG and PVLG+Melzer's reagent (Figures 4B–D, F). The *sporogenous cell* is concolorous or slightly lighter than the spore, usually ovoid, 13.8–59.0 × 18.8–38.4 μm, rarely globose, with a wall composed of two permanent layers that are continuous with spore wall layers 1 and 2; the sporogenous cell wall layer (scwl) 1 is hyaline, 0.8–1.3 μm thick, while scwl 2 is 2.0–3.3 μm thick (Figures 4A, B, G). The *germination shields* are hyaline, ellipsoidal, 24.6–47.0 × 6.8–38.4 μm, and bi-lobed (Figure 4H); the shields have one germ pore, are 4.0–6.4 μm in diameter, and located in the center of the shield; the shield wall is flexible to semi-flexible and 1.0–1.3 μm thick. The spore contains a hyaline oily substance. *Auxiliary cells* were not found.

*Ecology and distribution*: In the field, *S. graeca* probably lived in arbuscular mycorrhizal symbiosis with roots of *A. arenaria*, but no molecular analyses were performed to confirm this supposition. GenBank searches, using BLASTn, and our phylogenetic analyses (data not shown) revealed only two sequences, which suggested the presence of AMF identical to *S. gracea* and *S. intraundulata*, the latter described below, in other localities. The sequences were GU322902 with 99.28% identity and coverage = 49% from chalk grassland soil in the Netherlands and JX096615 with 96.87% identity and coverage = 87% from Qinghai-Tibet Plateau, China.

***Scutellospora intraundulata*** Błaszk., Niezgoda, B.T. Goto & Magurno, **sp. nov**.

Figures 5A–H, 6A, B

**Figure 5:**
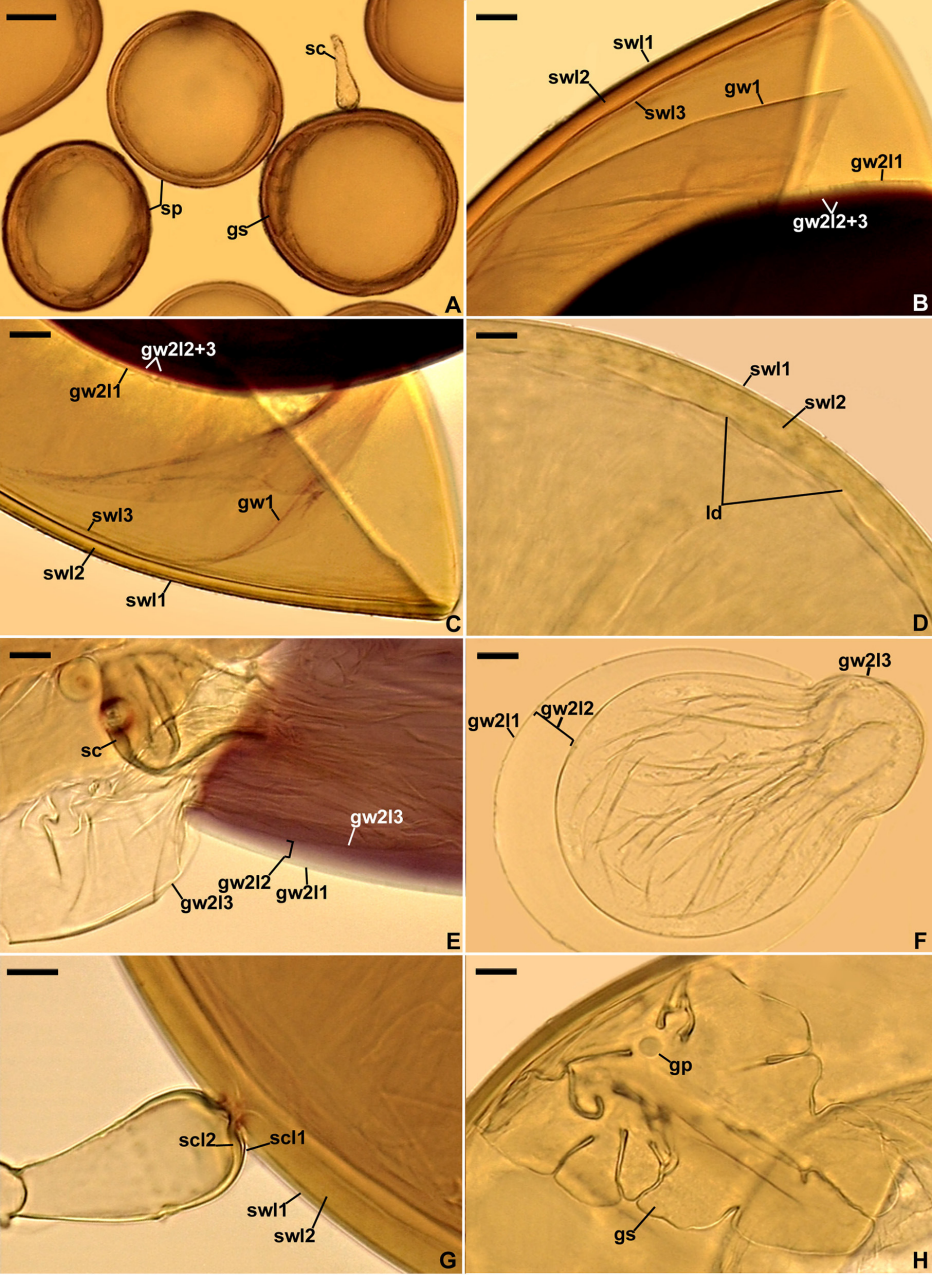
*Scutellospora intraundulata*. **(A)** Spores (sp) formed from the sporogenous cell (sc) and germination shield (gs) seen in cross-view. **(B, C)** Layers (l) 1–3 of spore wall (sw) and germinal wall (gw) 2. **(D)** Spore wall layers (swl) 1 and 2 and local depressions (ld) on the lower surface of the laminate swl2. **(E, F)** Germinal wall 2 layers (gw2l) 1–3; sporogenous cell (sc) is indicated in **(E)**. **(G)** Sporogenous cell wall layers (scwl) 1 and 2 continuous with spore wall layers (swl) 1 and 2. **(H)** Bi-lobed germination shield (gs) with germination pore (gp). (**A)** Spores in lactic acid. **(D, F, H)** Spores in PVLG. **(B, C, E, G)** Spores in PVLG+Melzer's reagent. **(A–H)** Differential interference microscopy. Scale bars: **(A)** = 50 μm, **(F)** = 20 μm, **(B–E, G, H)** = 10 μm.

**Figure 6:**
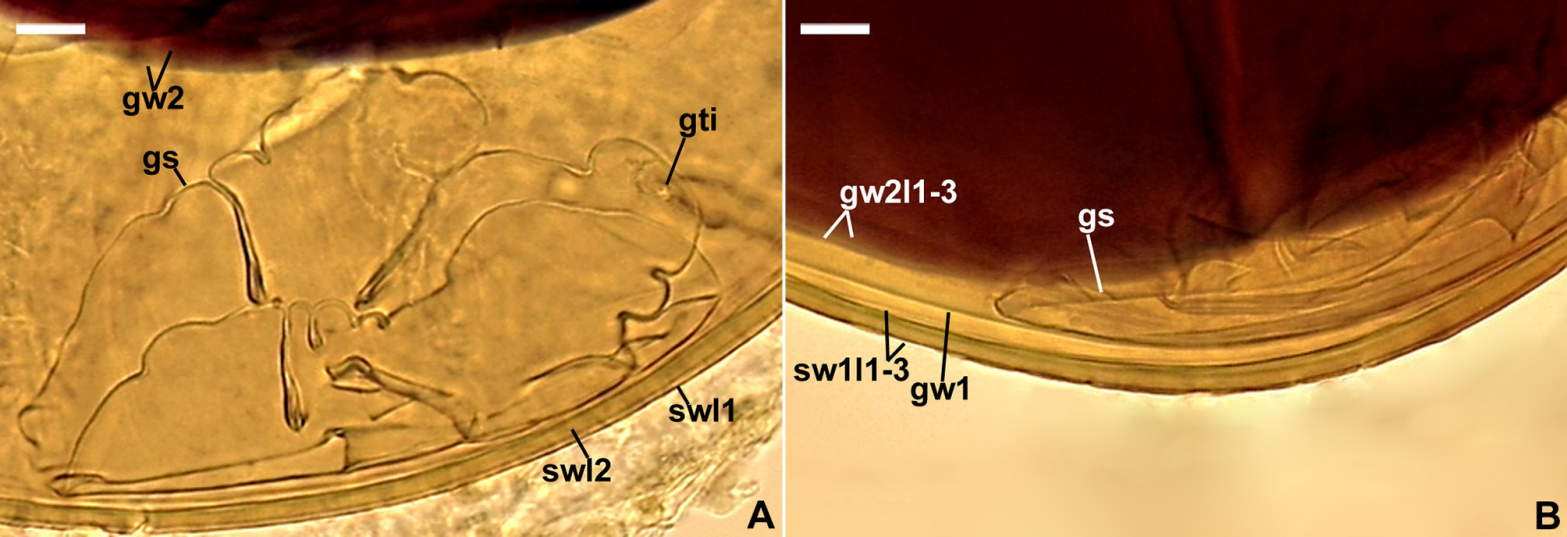
*Scutellospora intraundulata*. **(A)** Spore wall layers (swl) 1 and 2, germinal wall 2 (gw2), and germination shield (gs) with germ tube initiation (gti); swl 3 is invisible. **(B)** Layers (l) 1–3 of spore wall (sw), germinal 1 (gw1), germinal wall 2 layers (gw2l) 1–3, and germination shield (gs). **(A, B)** Spores in PVLG+Melzer's reagent. **(A, B)** Differential interference microscopy. Scale bars: **(A, B)** = 10 μm.

MycoBank No. MB 851414

*Etymology*: Latin, *intraundulata*, referring to the wavy lower surface of the laminate spore wall layer 2 of the species.

*Specimens examined*: GREECE. Spores from a trap culture inoculated with a field-collected mixture of the rhizosphere soil and root fragments of *Ammophila arenaria* inhabiting a maritime sand dune site of the beach Voidokoilia (36°57'N 21°39'E), the Peloponnese Peninsula, Greece, September 8, 2015, J. Błaszkowski (**holotype** slide with spores no. ZTMyc 0067009, **isotype** slides with spores no. 3933–3950, LPPDSE).

*Diagnosis*: For differences from *S. graeca*, the phylogenetic sister species (Figure 3), see the Diagnosis subsection under *S. graeca* (also, see Discussion for details). It differs from *Dentiscutata colliculosa* having a laminate spore wall layer with local depressions on its lower surface in (i) the size and color of spores, (ii) the composition of the spore wall and the phenotypic properties of the spore wall layer covering the laminate layer of this wall, (iii) the composition and the phenotypic properties of germinal wall 1, (iv) the features of the germination shield, and (v) the phylogenetic position among other sequenced species producing gigasporoid spores (Goto et al., 2010; also see Discussion for details).

*Description*: Forming glomerospores (= spores) and auxiliary cells in soil (Figure 5A). *Spores* are formed singly, terminally, and rarely laterally on bulbous sporogenous cells (Figures 5A, G). The spores are pale yellow (4A3) to golden yellow (5B8); globose; (128–)179(−220) μm in diameter; less often ovoid; 124–220 × 158–250 μm; with a spore wall, two germinal walls, and a germination shield on the upper surface of germinal wall 2 (Figures 5A–H, 6A, B). The *spore wall* is composed of three permanent layers (spore wall layers 1–3; Figures 5B–D, G, 6A, B). Layer 1, forming the spore surface, is uniform, smooth, semi-rigid, hyaline, (1.0–)1.5(−2.0) μm thick, always tightly adherent to the upper surface of layer 2 (Figures 5B–D, G, 6A, B), and often turning grayish yellow (4B3–B5) in Melzer's reagent (Figures 5B–D). Layer 2 is laminate, smooth on the upper surface, often with slight, local depressions, widely spaced on the lower surface, with the edge of this layer being wavy when observed in a cross view. It is semi-rigid, pale yellow (4A3) to golden yellow (5B8), (8.0–)10.4(−13.0) μm thick in thicker regions, (6.0–)7.9(−10.0) μm thick in thinner regions, consisting of very thin (< 0.5 μm) laminae, tightly adherent to and not separating from each other in even vigorously crushed spores (Figures 5B–D, G, 6A, B). Layer 3 is uniform, smooth, pale yellow (4A3), flexible to semi-flexible, approximately 1.0 μm thick, usually slightly separating from the lower surface of layer 2 in even moderately crushed spores (Figures 5B, C, 6B), staining reddish-white (10A2) to pale red (11A3) in Melzer's (Figures 5B, C, 6B). *Germinal wall 1* consists of one layer that is flexible, hyaline, approximately 1.0 μm thick, usually difficult to observe in spores crushed in PVLG, but better visible in spores crushed in PVLG+Melzer's reagent where it stains reddish-white (10A2–12A) in Melzer's (Figures 5B, C, 6B). *Germinal wall 2* is composed of three smooth, hyaline layers (germinal wall 2 layers 1–3; Figures 5B, C, E, F, 6A, B). Layer 1 is coriaceous sensu Walker (1986), (1.0–)1.7(−2.3) μm thick, always inseparably covering layer 2, and sometimes difficult to see in spores crushed in PVLG due to the lack of contrast with layer 2 (Figures 5B, C, E, F, 6A, B). Layer 2 is flexible, (1.0–)2.6(−4.3) μm thick in spores crushed in water and PVLG+Melzer's reagent, always plastic, amorphous sensu Morton (1986), strongly swelling (up to 29.0 μm thick) in spores crushed in lactic acid and PVLG (Figures 5B, C, E, F, 6A, B), and staining pastel pink (11A4) to violet brown (11F8) in Melzer's (Figures 5B, C, E, F, 6A, B). Layer 3 is flexible, (1.0–)1.1(−1.3) μm thick, loosely attached to the lower surface of layer 2, and often pushed partly or completely out from under this layer in spores vigorously crushed in PVLG and PVLG+Melzer's reagent (Figures 5B, C, E, F, 6A, B). The *sporogenous cell* is concolorous or slightly lighter than the spore, usually ovoid, 22.3–33.5 × 24.0–53.5 μm, rarely globose, with a wall composed of two permanent layers continuous with spore wall layers 1 and 2; the sporogenous cell wall layer (scwl) 1 is hyaline, 1.0–1.3 μm thick, scwl2 is pale yellow (4A3) to light yellow (4A4) and 1.5–3.8 μm thick (Figures 5A, E, G). The *germination shields* are hyaline, ellipsoidal to oblong, 87.8–144.4 × 42.3–79.5 μm, with two lobes of different sizes, one germ pore, 4.0–5.5 μm in diameter, located in the center of the shield or slightly below it, and one germ tube initiation located at the edge of the shield (Figures 5A, H, 6A, B); the shield wall is flexible to semi-flexible and 1.0–1.3 μm thick. The spore contains a hyaline oily substance. The *auxiliary cells* are hyaline, knobby, 7.5–8.8 × 14.6–16.5 μm, and formed on tips of straight or spirally coiled hyphae; both structures stain reddish-white (9A2) to pale red (9A3) in Melzer's.

*Ecology and distribution*: In the field, *S. intraundulata* probably lived in arbuscular mycorrhizal symbiosis with roots of *A. arenaria*, but no molecular analyses were performed to confirm this supposition. Many attempts at growing this species in single-species cultures with *P. lanceolata* as the host plant failed. Remarks on the possible occurrence of *S. intraundulata* in other sites are presented in the “*Ecology and distribution*” section under *S. graeca* (see above).

### Notes on *Acaulospora gedanensis*

***Acaulospora gedanensis*** Błaszk.

Karstenia 27: 38. 1988.

**Figure 7:**
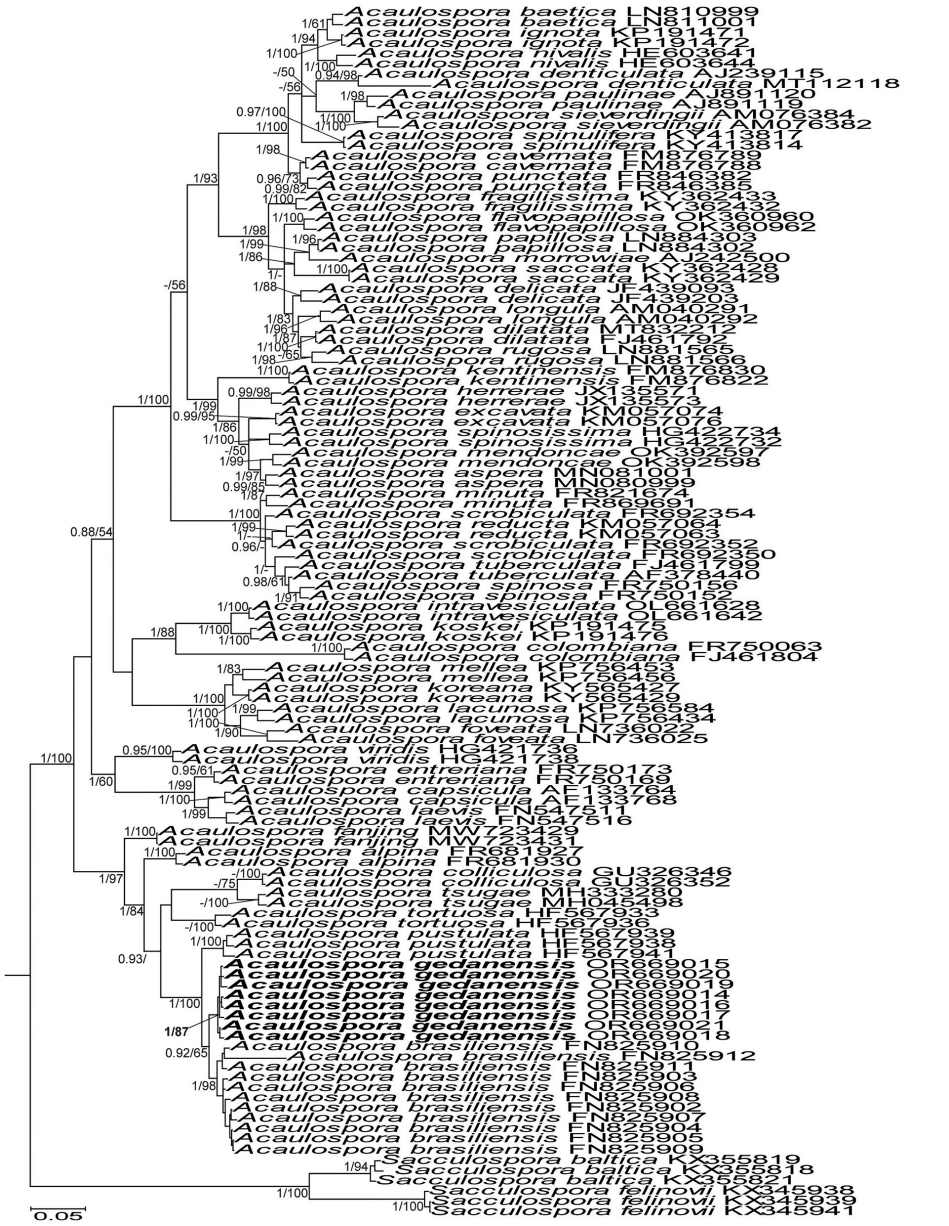
50% majority-rule consensus tree from the Bayesian analysis of sequences of 45S nuc rDNA of *Acaulospora gedanensis*, 47 other *Acaulospora* species, and two *Sacculospora* species serving as outgroup. *Acaulospora gedanensis* is in bold font. The Bayesian posterior probabilities ≥0.90 and ML bootstrap values ≥50% are shown near the branches. The bar indicates 0.05 expected change per site per branch.

**Figure 8:**
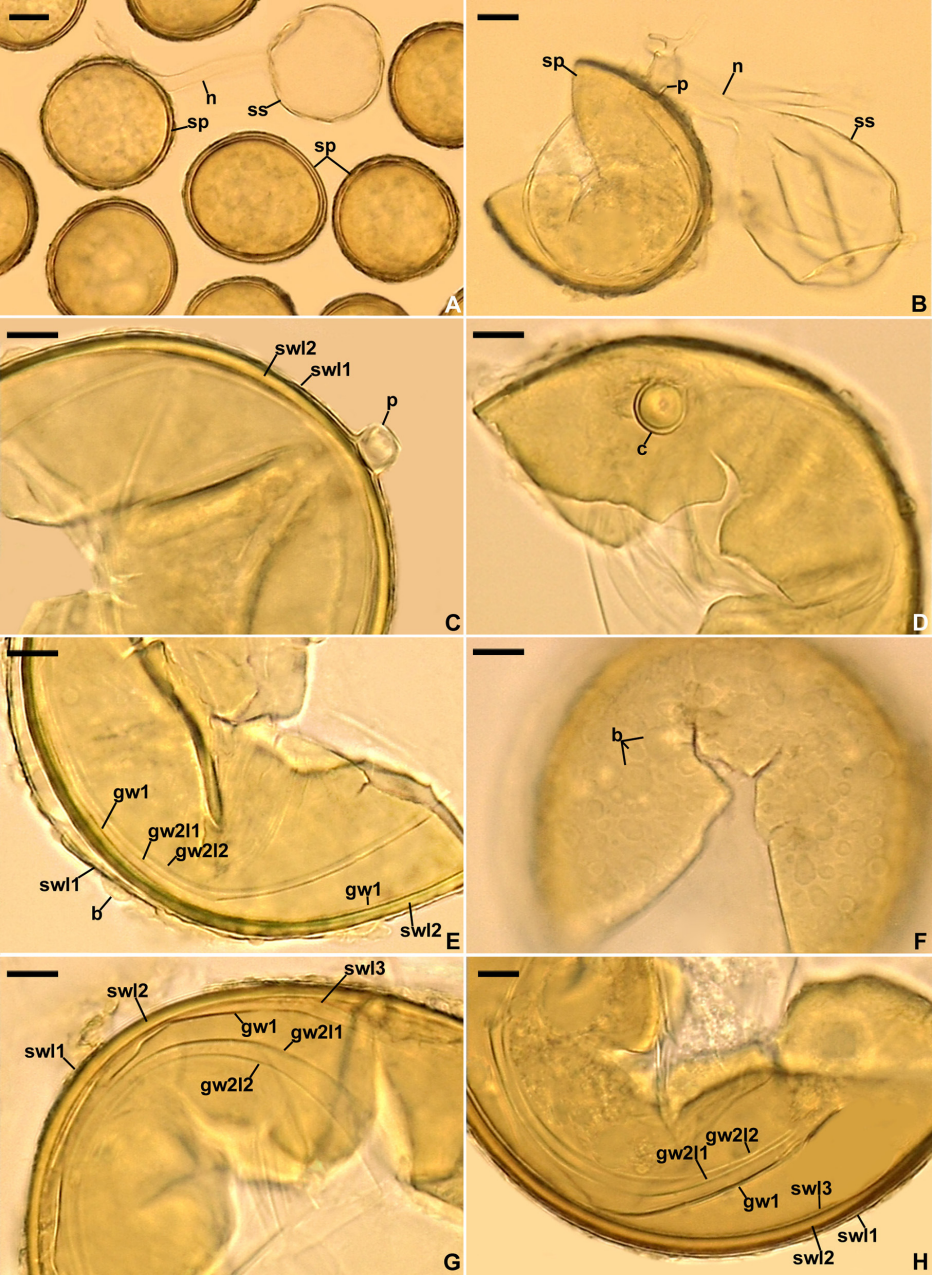
*Acaulospora gedanensis*. **(A, B)** Spores (sp) formed laterally on the sporiferous saccule (ss) neck (n); pedicel (p) of spore (sp) is indicated in **(B)**. **(C)** Spore wall layers (swl) 1 and 2 with swl1 continuous with the pedicel (p) wall. **(D)** Cicatrix (c) seen in plan view. **(E)** Spore wall layers (swl) 1 and 2, germinal wall (gw) 1, and germinal wall 2 layers (gw2l) 1 and 2; spore wall layer 3 is invisible; note the blister-like (b) ornamentation on the upper surface of swl1. **(F)** Blister-like (b) structures on the spore surface seen in plan view. **(G, H)** Spore wall layers (swl) 1–3, germinal wall 1 (gw1), and germinal wall 2 layers (gw2l) 1 and 2. **(A)** Spores in lactic acid. **(F, H)** Spores in PVLG. **B–E, G**. Spores in PVLG+Melzer's reagent. **(A–H)** Differential interference microscopy. Scale bars: **(A, B)** = 20 μm, **(C–H)** = 10 μm.

**Figure 9:**
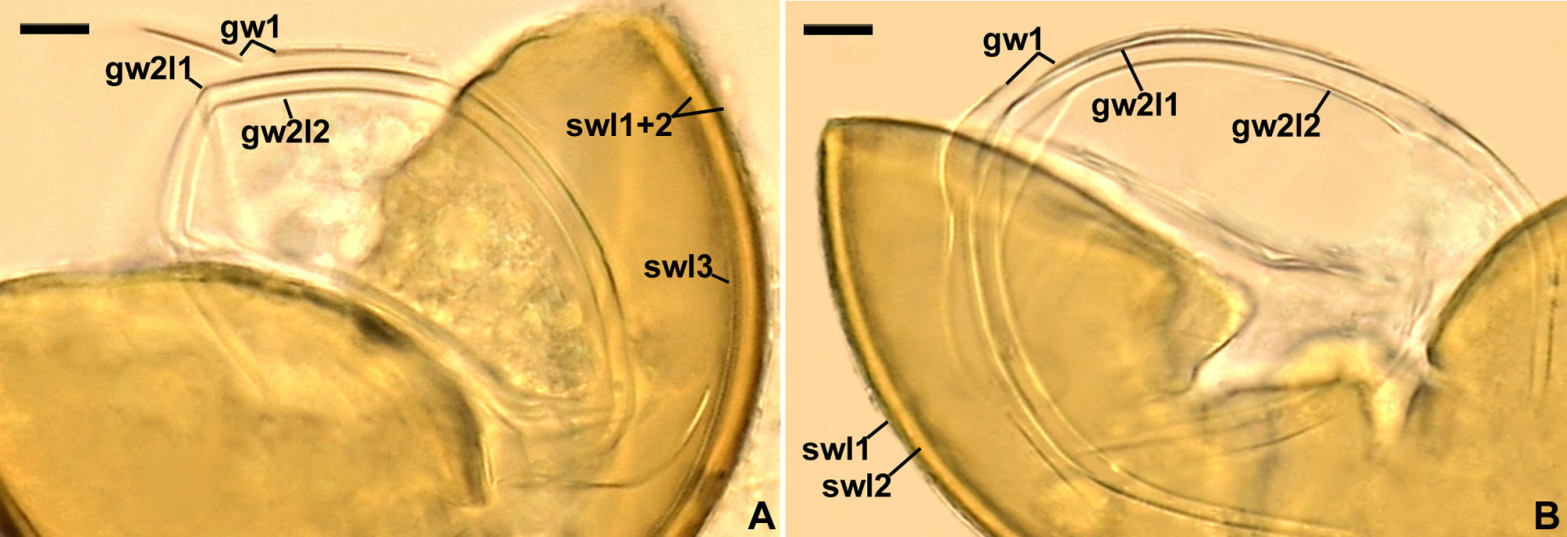
*Acaulospora gedanensis*. Spore wall layers (swl) 1–3, germinal wall 1 (gw1), and germinal 2 layers (gw2l) 1 and 2; spore wall layer 3 is invisible in **B**; note the fragility of gw1. **(A, B)** Spores in PVLG. **(A, B)** Differential interference microscopy. Scale bars: **(A, B)** = 10 μm.

Figures 7, 8A–H, 9A, B.

MycoBank No. MB 133511.

*Etymology*: Latin, *gedanensis*, referring to the Gdańsk Province in which the fungus was originally found.

*Specimens examined*: POLAND. Chałupy, under *Festuca ovina*, 23.08.1985, Błaszkowski J., 137 (holotype; DPP), 138–154 (isotypes; DPP); near Władysławowo, under *Festuca rubra*, July 18, 2022; Błaszkowski J., 3951–3968; Osłonino, among roots of *Juncus conglomeratus*, 3.04.1989, Błaszkowski J., 1856–1885; Chłapowo, in the root zone of *Juniperus communis*, 28.09.1988, Błaszkowski J., 1226–1229; Tatra Mountains, under *Leucanthemum waldsteinii* growing in Dolina Ku Dziurze valley, 1.100 m a.s.l., June–July 2003, and beneath *Senecio macrophyllus* growing in Szeroki Żleb gully in Chochołowska valley, 1.130 m a.s.l., leg. Zubek Sz., Błaszkowski J., unnumb. coll.

*Diagnosis*: Differs from (A) *A. brasiliensis* and *A. pustulata*, the phylogenetically closest species (Figure 7), in: (i) that the upper surface of spore wall layer 1 of *A. gedanensis* is usually smooth, rarely ornamented with small outgrowths (vs. always ornamented with relatively larger outgrowths in *A. brasiliensis* and *A. pustulata*), (ii) color and size of spores, as well as the morphometric features of the spore and germinal walls and the phenotypic properties of germinal wall 1 (to *A. brasiliensis*), (iii) the composition and flexibility of germinal wall 1 and the lack of granular ornamentation on the upper surface of germinal wall 2 layer 1 (to *A. pustulata*), and (iv) nucleotide composition of sequences of the 45S nuc rDNA region (see Discussion for details); (B) *A. flavopapillosa* and *A. tortuosa*, producing acaulosporoid spores with spore wall layer 1 ornamented on the upper surface, in (i) spore size, (ii) the morphology of the ornamentation, (iii) the composition and phenotypic properties of germinal walls 1 and 2 (Palenzuela et al., 2013; Corazon-Guivin et al., 2022), and (iv) the phylogenetic position to other sequenced *Acaulospora* species (Figure 7).

*Description*: Glomerospores (= spores) are formed singly in soil, laterally, either directly on the neck of a sporiferous saccule (sessile spores) or at the top of a short branch (pedicel) of the neck; the sporiferous saccules are hyaline, globose to subglobose, 60–70 μm in diameter, with a single-layered wall, 0.8–1.0 μm thick, continuous with the neck wall and spore wall layer 1, forming the spore surface; necks 36–60 μm long, tapering from a 17–20 μm diameter at the saccules to a 6–15 μm diameter at the point of spore attachments; the neck's wall is 0.8–1.0 μm thick (Figures 8A–C). Spores have a slightly raised, circular thickening of layer 2 of the spore wall (cicatrix) or a cylindrical to slightly funnel-shaped pedicel, 1.3–10.0 μm long, 5.0–9.4 μm wide, with a wall 1.4–2.0 μm thick, and continuous with spore wall layer 1 when observed in a cross view (Figures 8B, C); the cicatrix and pedicel surround a circular (2.0–3.5 μm in diameter) slight depression in layer 2 of the spore wall when seen in a plan view (Figure 8D). The sporiferous saccules usually collapse or fall off in mature spores. The *spores* are pale yellow (3A3) to lemon yellow (3B8), globose to subglobose, (55–)65(−75) μm in diameter, with a spore wall and two germinal walls (Figures 8A–H, 9A, B). The *spore wall* has three layers (spore wall layers 1–3; Figures 8E, G, H, 9A, B). Layer 1 is semi-permanent, rarely strongly deteriorated or completely sloughed off, hyaline, and (0.8–)1.3(−1.6) μm thick; the upper surface of this layer usually is smooth when intact or slightly roughened when deteriorated, rarely covered with blister-like outgrowths, 1.2–2.8 μm high and 1.8–7.6 μm wide when seen in cross and plan views, respectively (Figures 8C, E, G, H, 9A, B); in a plan view, the outgrowths are circular to ellipsoidal and more or less uniformly distributed (Figure 8F). Layer 2 is permanent, laminate, smooth, semi-rigid, pale yellow (3A3) to lemon yellow (3B8), and (2.2–)3.6(−4.3) μm thick (Figures 8C, E, G, H, 9A, B). Layer 3 is uniform, smooth, flexible to semi-flexible, concolorous with layer 2, 0.8–1.0 μm thick, and rarely separating from the lower surface of layer 2 in even vigorously crushed spores; therefore, it is usually difficult to detect (Figures 8G, H, 9A). The *germinal wall 1* has one rigid, fragile, hyaline, 0.3–0.5 μm thick layer, often breaking in even moderately crushed spores (Figures 8E, G, H, 9A, B). *Germinal wall 2* consists of two flexible to semi-flexible, smooth, hyaline layers (germinal wall 2 layers 1 and 2), 1.0–1.8 μm thick and 0.8–1.3 μm thick, respectively, always easily separating from one another in crushed spores (Figures 8E, G, H, 9A, B). None of the layers of the spore wall and germinal walls stain in Melzer's reagent (Figures 8B–E, G, H). A *germination orb* was not found, and *germination* is unknown.

*Ecology and distribution*: Associated in the field with vesicular-arbuscular mycorrhizal roots of *Festuca ovina, Helictotrichon pubescens, Juncus conglomeratus, Juniperus communis, Rosa rugosa* (Błaszkowski, 1988, 1993a,b, 1994), *Leucanthemum waldsteinii, Thymus pulcherrimus* (Zubek et al., 2008), *Senecio macrophyllus* (Zubek, pers. comm.), and *F. rubra* (this study). All attempts to establish this species in one-species cultures with *P. lanceolata* as the host plant failed. It occurred in maritime sand dunes near Chałupy on the Hel Peninsula, a forest near Żelistrzewo, a wet meadow in Osłonino, an uncultivated meadow located approximately 100 m from the Baltic Sea in Chłapowo (all sites located in Pomeranian voivodeship; Błaszkowski, 1988, 1993a,b, 1994), and in Tatra Mountains (Zubek et al., 2008). Apart from Poland, it was found only in Austria (Błaszkowski, pers. observ.) and Brazil (Lugo et al., 2023). Bayesian inference and ML analyses of sequences deposited in GenBank showed that no sequence with >96% identity to the *A. gedanensis* 45S sequences clustered with this species (data not shown).

## Discussion

The above described phylogenetic analyses with 45S and *rpb1* sequences of four AMF (i) confirmed the preliminary morphological studies and BLAST's indications that the fungus with glomoid spores and two morphotypes producing gigasporoid spores represented three new Glomeromycota species (Figures 1, 3, Supplementary Figure S1), (ii) showed their closest sequenced relatives (Figures 1, 3, Supplementary Figure S1), and (iii) grouped *A. gedanensis* with sequenced *Acaulospora* species characterized by a distinctively atypical morphology (Figure 7). These analyses showed that the closest relative of the new glomoid spore-producing species, described here as *D. vistulana*, was *D. densissima* in Diversisporaceae (Figure 1, Supplementary Figure S1), and the two new species with gigasporoid spores, named *S. graeca* and *S. intraundulata*, are sisters in Scutellosporaceae (Figure 3). The novelty of these species was also strongly supported by the magnitudes of divergences between the 45S sequences of *D. vistulana* vs. *D. densissima* and *S. graeca* vs. *S. intraundulata*. In addition, the morphology of the three new species clearly differed from that of their closest phylogenetic relatives.

The morphological features that most strongly separate *D. vistulana* from *D. densissima* are the number of spore wall layers and the thickness of the spore wall. *Diversispora vistulana* lacks the flexible to semi-flexible innermost spore wall layer 3 of *D. densissima*, and the spore wall of the former species is 1.9–2.4-fold thinner (Błaszkowski et al., 2022). In addition, *D. densissima* spores are clearly darker-colored [pale orange (5A3) to light brown (6D8)], and their subtending hypha may be up to 1.7-fold narrower. The genetic distance between these two species (5.0%−6.7%) is greater than the genetic distance between other closely related *Diversispora* species. For example, the distances between the *D. celata* AM713402 and *D. eburnea* AM713406 sequences and the *D. alba* OP195880 and *D. spurca* FN547639 sequences (Figure 1, Supplementary Figure S1) are 4.5% and 4.2%, respectively.

The main morphological feature distinguishing the two new *Scutellospora* species is the uniformness in thickness of the spore wall of *S. graeca* (Figures 4A–E, H) compared to the non-uniform thickness of the *S. intraundulata* spore wall (Figure 5D). This confirms the conclusion of Morton et al. (1995) that the morphological differences between species in Glomeromycota reside in the phenotypic characters of the spore wall. Moreover, compared to *S. intraundulata* spores, those of *S. graeca* are clearly lighter-colored, more frequently ovoid than globose (vs. usually globose), 1.1–1.3-fold smaller when globose, and their spore wall may be up to 1.6-fold thinner (Figures 4A–E, G, H, 5A–C).

Of the species producing gigasporoid spores, the lower surface of the laminate spore wall 1 is wavy only in *Dentiscutata colliculosa* (Goto et al., 2010). The morphological differences clearly separating *S. intraundulata* and *D. colliculosa* occur in the spore size and color, the spore wall, the germinal wall 1, and the germination shield. The spores of *S. intraundulata* are approximately 2.5-fold smaller when globose and much lighter-colored (vs. red-brown to black) and do not have spore wall layer 2 of *D. colliculosa*. The germinal wall 1 of *S. intraundulata* is single-layered (vs. two-layered in *D. colliculosa*) and much thinner (approximately 1 μm thick vs. 3.1–5.2 μm). The germination shield of *S. intraundulata* is colorless (vs. yellow-brown to brown) and is not dentate at the margin as in *D. colliculosa*.

Our phylogenetic analyses evidenced that *S. ovalis* is the closest relative of both *S. graeca* and *S. intraundulata*. However, the phenotypic features of the spore wall layer 1, the composition of the spore wall and two germinal walls, as well as the histochemical properties of germinal wall 1 differentiate *S. ovalis* from both species. In *S. ovalis*, the spore wall layer 1 often disappears due to sloughing off as the spores age (Crossay et al., 2018; vs. it is permanent in *S. graeca* and *S. intraundulata*; Figures 4B–E, 5B–D, G, 6A, B). The spore wall and the germinal wall 2 of *S. ovalis* lack the innermost layer 3 of the spore wall, and the germinal wall 2 of the two new *Scutellospora* species (Figures 4B–E, 5B, C, 6B), and germinal wall 1 is two-layered in *S. ovalis* (vs. single-layered; Figures 4D, E, 5B, C, 6B), of which none stains in Melzer's reagent (vs. it stains clearly; Figures 4D, E, 6B, C). In addition, the spore wall of *S. ovalis* vs. that of *S. intraundulata* is uniform in thickness (vs. often with local depressions on the lower surface; Figure 5D) and 1.3–1.5-fold thinner. Molecularly, the identities of *S. ovalis* 45S sequences differ from those of *S. graeca* and *S. intraundulata* by 4.3%−7.6% and 7.0%−7.2%, respectively.

The phylogenetic analyses showed that *A. gedanensis* is nested in a clade formed by other species with atypical spore morphology: (i) the lack or rare presence of a beaded layer 1 in germinal wall 2, (ii) the absence of Melzer's reaction in layer 2 of this wall, and (iii) the easy separation of these two layers from each other in even moderately crushed spores. Other species that share these features are *A. brasiliensis, A. pustulata, A. colliculosa*, and *A. tortuosa*, the first two being the closest relatives of *A. gedanensis* (Figure 7), except for *A. colliculosa*, in which layers 1 and 2 of the germinal wall 2 do not separate from each other.

*Acaulospora gedanensis* differs mainly from *A. brasiliensis* and *A. pustulata* in the properties of the structures ornamenting the upper surface of their spores. Krüger et al. (2011) characterized *A. brasiliensis* spores as having the upper surface of the spore wall ornamented with semi-permanent, large (up to 10 μm high, up to 20 × 30 μm wide) outgrowths, which rarely are highly deteriorated and difficult to see. According to Palenzuela et al. (2013), *A. pustulata* spores are covered with permanent, densely crowded projections that are 1.2–5.5 μm high and 1.2–9.5 μm wide at the base. In our study, most *A. gedanensis* spores were smooth, or the blister-like ornamentation of their surface was difficult to detect due to its small size or short duration (Figures 8E, F), even in freshly matured spores still associated with the sporiferous saccule necks (Figures 8A, B), which usually detach from older spores (Figures 8A, C–H, 9A, B). In addition, compared to *A. gedanensis*, the thickness of components of all three *A. brasiliensis* walls is 1.2–1.8-fold lower, and the single-layered germinal wall 1 is flexible. Although the germinal wall 1 of *A. gedanensis* is only 0.3–0.5 μm thick (vs. up to 1 μm thick in *A. brasiliensis*), it usually breaks in even moderately crushed spores due to its rigidity and fragility (Figures 8G, H, 9A, B)—a phenomenon not found in any other *Acaulospora* species. Finally, *A. brasiliensis* spores may be much darker-colored (yellowish brown to yellowish-red) and clearly larger (48–91 × 51–100 μm diam) when globose to subglobose (vs. pale yellow to lemon yellow; 55–75 μm in diameter in *A. gedanensis*). Other features that distinguish *A. gedanensis* from *A. pustulata* are the composition and phenotypic features of germinal walls 1 and 2. In *A. pustulata*, the germinal wall 1 consists of two semi-flexible layers (vs. one rigid layer) and the upper surface of layer 1 of the three-layered germinal wall 2 is rarely ornamented with granular excrescences (Palenzuela et al., 2013) (vs. is smooth), unlike the vast majority of *Acaulospora* species in which this ornamentation is present and easy to notice (da Silva et al., 2022).

## Data availability statement

The datasets presented in this study can be found in online repositories. The names of the repository/repositories and accession number(s) can be found below: NCBI–OR669295, OR669296, OR669014–OR669021, OR669025–OR669038.

## Author contributions

PN: Formal analysis, Funding acquisition, Investigation, Methodology, Writing—original draft, Writing—review & editing. JB: Conceptualization, Data curation, Formal analysis, Investigation, Methodology, Resources, Supervision, Visualization, Writing—original draft, Writing—review & editing. TB: Data curation, Formal analysis, Writing—review & editing. AS: Data curation, Formal analysis, Writing—review & editing. SZ: Formal analysis, Funding acquisition, Writing—review & editing. PM: Data curation, Writing—review & editing. RM: Data curation, Formal analysis, Writing—review & editing. EM: Formal analysis, Methodology, Writing—review & editing. MM: Data curation, Formal analysis, Investigation, Methodology, Writing—review & editing. BG: Conceptualization, Data curation, Formal analysis, Funding acquisition, Investigation, Methodology, Resources, Writing—original draft, Writing—review & editing. SU: Investigation, Data curation, Methodology, Writing —review & editing. LC: Data curation, Writing —review & editing. FM: Investigation, Data curation, Methodology, Formal analysis, Writing—review & editing.

## Supplementary material

Supplementary Figure S1 50% majority-rule consensus tree from the Bayesian analysis of sequences of 45S nuc rDNA of *Diversispora vistulana*, 22 other *Diversispora* species, as well as *Corymbiglomus corymbiforme, Desertispora omaniana, Redeckera megalocarpum*, and *Sieverdingia tortuosa* serving as outgroup. The new species is in bold font. The Bayesian posterior probabilities ≥0.90 and ML bootstrap values ≥50% are shown near the branches, respectively. The bar indicates 0.05 expected change per site per branch.

Supplementary Table 1 Characteristics of the sequence alignments with *Diversispora* 448, *Scutellospora* 431, *Scutellospora* 437, and *Acaulospora gedanensis*.

Supplementary Table 2 An alignment used to produce Figure 1.

Supplementary Table 3 An alignment used to produce Figure 3.

Supplementary Table 4 An alignment used to produce Figure 7.

Supplementary Table 5 An alignment used to produce Supplementary Figure S1.

Supplementary Table 6 Data obtained from a BI analysis of 45S sequences (see Supplementary Figure S1).

Supplementary Table 7 Data obtained from a RAxML-NG analysis of 45S sequences (see Supplementary Figure S1).

Supplementary Table 8 Data obtained from a BI analysis of 45S+rpb1 sequences (see Figure 1).

Supplementary Table 9 Data obtained from a RAxML-NG analysis of 45S+rpb1 sequences (see Figure 1).

Supplementary Table 10 Data obtained from a BI analysis of 45S sequences (see Figure 3).

Supplementary Table 11 Data obtained from a RAxML-NG analysis of 45S sequences (see Figure 3).

Supplementary Table 12 Data obtained from a BI analysis of 45S sequences (see Figure 7).

Supplementary Table 13 Data obtained from a RAxML-NG analysis of 45S sequences (see Figure 7).
