# Supplementary figures and images for: Three new species of arbuscular mycorrhizal fungi (Glomeromycota) and Acaulospora gedanensis revised
Source: Front Microbiol. 2024 Feb 12;15:1320014. doi: 10.3389/fmicb.2024.1320014 (PMC10896085; doi:10.3389/fmicb.2024.1320014)

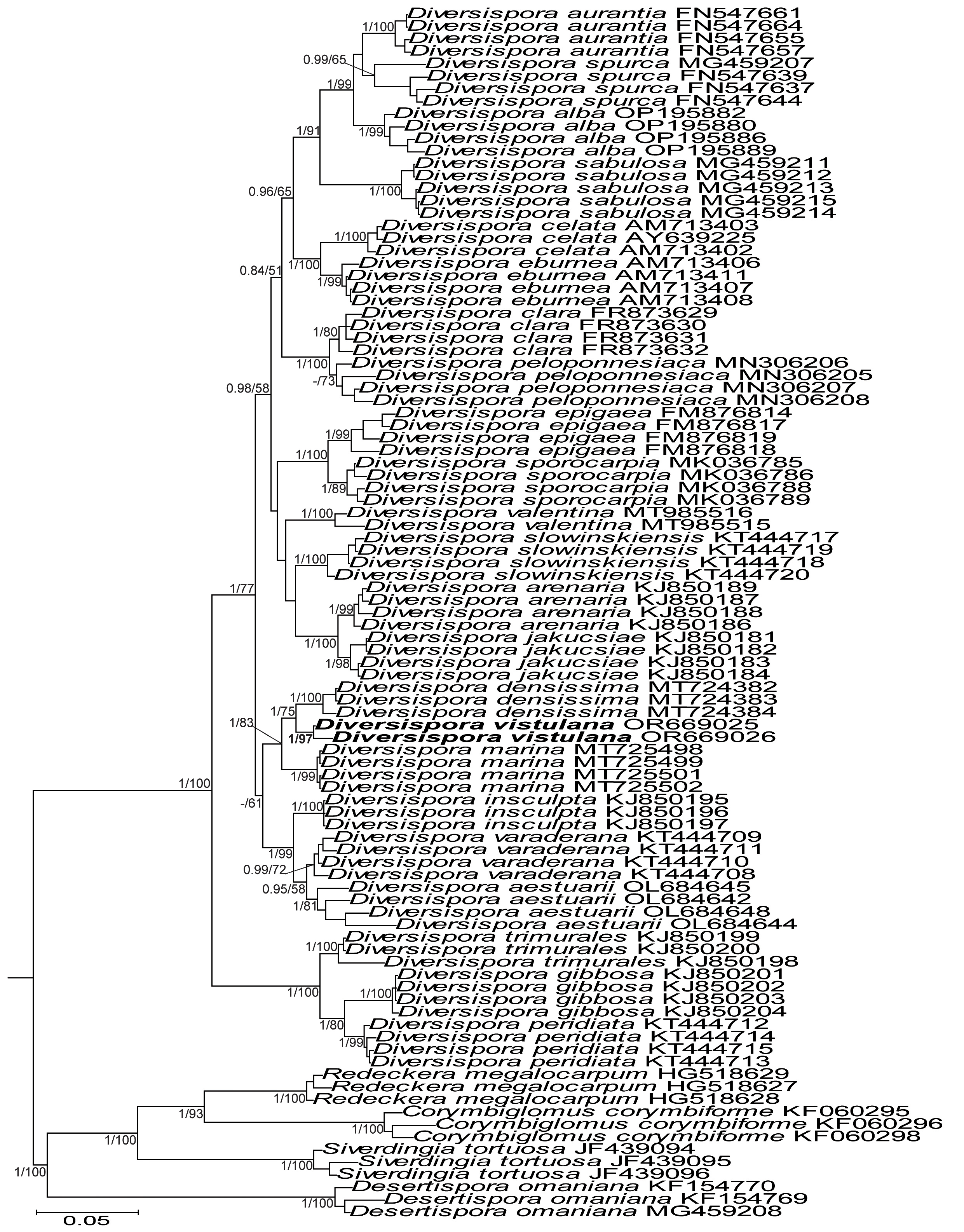

Supplement: Supplementary Figure S1 — 50% majority-rule consensus tree from the Bayesian analysis of sequences of 45S nuc rDNA of Diversispora vistulana, 22 other Diversispora species, as well as Corymbiglomus corymbiforme, Desertispora omaniana, Redeckera megalocarpum, and Sieverdingia tortuosa serving as outgroup. The new species is in bold font. The Bayesian posterior probabilities ≥0.90 and ML bootstrap values ≥50% are shown near the branches, respectively. The bar indicates 0.05 expected change per site per branch. [file Image_1.jpg]
