# Supplementary material for: Three new species of arbuscular mycorrhizal fungi (Glomeromycota) and Acaulospora gedanensis revised
Source: Front Microbiol. 2024 Feb 12;15:1320014. doi: 10.3389/fmicb.2024.1320014 (PMC10896085; doi:10.3389/fmicb.2024.1320014)
Supplement: Supplementary Table 1 — Characteristics of the sequence alignments with Diversispora 448, Scutellospora 431, Scutellospora 437, and Acaulospora gedanensis. [file Table_1.DOCX]

**Supplementary Table 1** Characteristics of the sequence alignments with *Diversispora* 448, *Scutellospora* 431, *Scutellospora* 437, and *Acaulospora gedanensis*.

| Name of alignment | No. of sequences | No. of fungal species | No. of base pairs | No. of variable sites | No. of parsimony informative sites |
| --- | --- | --- | --- | --- | --- |
| *Diversispora* 448 | | | | | |
| 45S | 94 | 26 | 1761 | 691 | 575 |
| *RPB1* | 42 | 21 | 1162 | 245 | 204 |
| 45S+*RPB1* | 94 | 26 | 2923 | 935 | 779 |
| *Scutellospora* 431 and *Scutellospora* 437 | | | | | |
| 45S | 28 | 9 | 1542 | 268 | 194 |
| *Acaulospora gedanensis* | | | | | |
| 45S | 118 | 50 | 1805 | 928 | 724 |
