# Supplementary material for: Three new species of arbuscular mycorrhizal fungi (Glomeromycota) and Acaulospora gedanensis revised
Source: Front Microbiol. 2024 Feb 12;15:1320014. doi: 10.3389/fmicb.2024.1320014 (PMC10896085; doi:10.3389/fmicb.2024.1320014)
Supplement: Supplementary Table 2 — An alignment used to produce Figure 1. [file Table_2.DOCX]

>448_3_5

AGGAATCCCTAGTAAGCATGAGTCATCAGCTCGTG-CTGATTACGTCCCTGCCCTTTGTACACACCGCCCGTCGCTACTACCGATTGAATGGCTTAGTGAGACCTTTGGATTGGGGTTTAGGGATCGGAAACACGGATCCTTATTCTCCGAGAAGTCGGTCAAACTTGGTCATTTAGAGGAAGTAAAAGTCGTAACAAGGTTTCCGTAGGTGAACCTGCGGAAGGATCATT--AAAAAT-TTATATTCCGGGAATTCG-----TTTCGT----T--------TTCCCGTG------ATTATTTGTATTCAAA-TCCCACTCTTT-------AT-AAAT-ATA---------TTAATTATATAAAAC--AAAA-A-TAAAAAAGAAAACTTTCAACAACGGATCTCTTGGCTCTCGCATCGATGAAGAACGCAGCGAAATGCGATACGTAGTGTGAATTGCAGAATTCCGTGAATCATCGAA-TCTTTGAACACAAATTGTACTTTCCAGTATTCTGGGAAGTATGCTTGGTTGAGGGTCATTAAAATAACA-TTCGTGAA--------------TTTTTTTG-------------CGGATTTGAG---TTTTT-CC--AGTA--TTT---AT---AAT------A--------TA-AAAATGTTGGTAACTTT-AAAATT-ATTT------ATTACTTGGTACAAGTT-GAAGACGTTC-TATA--TGTGT-GGTTCGCT-GACAACTTGTCCA--T-C--TT----T-ATAT-ATTATACA-CGCACTTGG--TTTTTT----TACGCTCTGTGC--GAGT--ATATA------TTTTTTTTATGAC-CTCAGCTCAAGTAAGAATACCC-GCTGAACTTAAGCATATCAATAAGCGGAGGAAAAGAAACTAACAAGGATTCCCCTAGTAACGGCGAGTGAAGAGGGAAAAGCTCAAATTTTAAATCTACC-TGG---TTC--CCAGGTCGAATTGTAATTTGAAGAAGCGATATC-GG-TG-TTGAGGTCTGGTTCAAGTTCTTTGGAACAAGACATCAT-GG-AGGGTGAGAATCCCGTGCATGATCAGA-CC---AAAAT--AC-TTAAT---ATTCGCTTTCTAAGAGTCGAGTTGTTTGGGAATGCAGCTCAAAATGGG-T-GGTAGAC-TTCACCTAAGGCTAAATATCAGCGAGAGACCGATAGCGAACAAGTACTGTG-AA-GGAAAGATGAAAAGAACTTTGAAAAGAGAGTTAAATAGTACGTGAAATTGTTGAAAGGGAAACGATTGAAGTCAGTCATGCTAG-TGAAAAATCAGTTTGACGGG-TTTTG-AGTTCGGGAGT--AGAGGCAGGGT---CAA---ACCGTTTCTCTTTTGGACTTGGGATTTGTTGGATGTACTTTTTCT-TTGGCAGGTCAGCGTCGATTTC-GGAGGTTGTAAAATACTTATTATACTTGAGGA-AAAGTAGCTCTGCTTCGGGA---GAGTA-TTATAGACCGTGGGGGATGCAGCCTGTGGGATCGAGGATTGCAGCAAATGCAAAGTACGGCCTATAACCGACTTT--GGC-TTGTCGCCTGATCTCTGG-ATGTTACCTCGCTTGTGACAACATT-CTTGCCACCGGTGAGTACTAATGCCCATTAGGTTAGAACGATCAAAAA-TTTTGCTAAGGATGCTGACGTAATGGCTTTAAACGACCCGTCTTGAAACACGGACCAAGGAGTCTAACATATGTGCGAGctgattggatgatcataactatacttcctgttcctcctcctcctgtacgtcccagtattcaaatggacggaacaagtcgaggtgaagatgatttgactcacaaattatccgacatcttgaaggcaaatcaaaacgtaaaacgttatgaagctgatggccatccaccacacgttgtaaacgaatttgaagcattgttacaggttcttaatgat---------------------------------------------------------------------------------------------------------tataa-tttca--------a-ttagtttaatatcaa--ttgaaa--attatacttaaa-tttatcatttat-----atgcaaatagtttcattgtgcaacttatatggacaatgaaatggctggtcaacctcaagctcttcagaaatccggtagacctttaaagtcaatacgtgcgcgtctcaagggtaaagaagggcgtttacgcggcaatctgatgggaaagcgtgtagatttctctgctcgtacagtaattacgggtgatccaaatatttcagttgatgaagtcggagttccgaaaagcatagctcaaaatctgacttttccagaattggtgactccctttaatattgactatcttcaaaaattagtagaaaatggcccttctacacatccaggggctaaatatgtaattagagatactggtgaaagaattgatctaaaacatatatcaggcatgactggtggcttaagattacactacggttggaaagttgaacgtcgtctcaatgatggtgacatcgttatattcaatcgtcagccatctttgcacaagatgtcgatgatgggacataaagttcgtgttatgccctattcgaccttccgtcttaatttatcagttacaacaccttataacgccgattttgatggtgacgaaatgaacatgcatgttccccaatcagtggaaactaaagcagaaatttcagaaatttgtatggttcctaaacaaattgtatctcctcaatcaaataaacctgttatgggtattgtacaggatactttatgtgctgttagaaaatttacaaaaagggattgctttttatctaaagatttggtaatgaacatt

>448_4_2

AGGAATCCCTAGTAAGCATGAGTCATCAGCTCGTG-CTGATTACGTCCCTGCCCTTTGTACACACCGCCCGTCGCTACTACCGATTGAATGGCTTAGTGAGACCTTTGGATTGGGGTTTAGGGATCGGAAACACGGATCCTTATTCTCCGAGAAGTCGGTCAAACTTGGTCATTTAGAGGAAGTAAAAGTCGTAACAAGGTTTCCGTAGGTGAACCTGCGGAAGGATCATT--AAAAAT-TTATATTCCGGGAATTCG-----TTTTGT----T--------TTCCCGTG------ATTATTTGTATTCAAA-TCCCACTCTTT-------AT-AAAT-ATA---------TTAATTATATAAAAC--AAAA-A-TAAAAAAGAAAACTTTCAACAACGGATCTCTTGGCTCTCGCATCGATGAAGAACGCAGCGAAATGCGATACGTAGTGTGAATTGCAGAATTCCGTGAATCATCGAA-TCTTTGAACACAAATTGTACTTTCTAGTATTCTGGGAAGTATGCTTGGTTGAGGGTCATTAAAATAACA-TTCGTGAA--------------TTTTTTTG-------------CGGATTTGAG----TTTT-CC--AGTA--TTT---AT---AAT------A--------TA-AAAATGTTGGTAACTTT-AAAATC-ATTT------ATTACTTGGTACAAGTT-GAAAACGTGC-TATA--TATGT-GGTTCGCT-GACAACTTGTCCA--T-C--TT----T-ATAT-ATTATACA-CGCACTTGG--TTTTTT----TACGCTTTGTGC--GAGT--ATATA------TTTTTTTTATGAC-CTCAGCTCAAGTAAGAATACCC-GCTGAACTTAAGCATATCAATAAGCGGAGGAAAAGAAACTAACAAGGATTCCCCTAGTAACGGCGAGTGAAGAGGGAAAAGCTCAAATTTTAAATCTACC-TGG---TTC--CCAGGTCGAATTGTAATTTGAAGAAGCGATATC-GG-TG-TTGAGGTCTGGTTCAAGTTCTTTGGAACAAGACATCAT-GG-AGGGTGAGAATCCCGTGCATGATCAGA-CC---AAAAT--AC-TTAAT---ATTCGCTTTCTAAGAGTCGAGTTGTTTGGGAATGCAGCTCAAAATGGG-T-GGTAGAC-TTCACCTAAGGCTAAATATCAGCGAGAGACCGATAGCGAACAAGTACTGTG-AA-GGAAAGATGAAAAGAACTTTGAAAAGAGAGTTAAATAGTACGTGAAATTGTTGAAAGGGAAACGATTGAAGTCAGTCATGCTAG-TGAAAAATCAGTTTGACGGG-TTTTG-AGTTCGGGAGT--AGAGGCAGGGT---CAA---ACCGTTTCTCTTTTGGACTTGGGATTTGTTAGATGTACTTTTTCT-TTGGCAGGTCAGCGTCGATTTC-GGAGGTTGTAAAATACTTATTATACTTGAGGA-AAAGTAGCTCTGCTTCGGGA---GAGTA-TTATAGACCGTGGGGGATGCAGCCTGCGGAATCGAGGATTGCAGCAAATGCAAAGTACGGCCTATAACCGACTTT--GGC-TTGTCGCCTGATCTCTGG-ATGTTACCTTGCTTGTGACAACATT-CTTGTCACCGGTGAGTACTAATGCCCATTAGGTTAGAACGATCAAAAA-TTTTGCTAAGGATGCTGACGTAATGGCTTTAAACGACCCGTCTTGAAACACGGACCAAGGAGTCTAACATATGTGCGAGctgattggatgatcataactatacttcctgttcctcctcctcctgtacgtcccagtattcaaatggacggaacaagtcgaggtgaagatgatttgactcacaaattatccgacatcttgaaggcaaatcaaaacgtaaaacgttatgaagctgatggccatccaccgcacgttgtaaacgaatttgaagcatcgttacaggttcttaatgat---------------------------------------------------------------------------------------------------------tataa-tttca--------a-ttagtttaatatcaa--ttgaaa--attatacttaaa-tttatcatttat-----atgcaaatagtttcactgtgcaacttatatggacaatgaaatggctggtcaacctcaagctcttcagaaatccggtagacctttaaagtcaatacgtgcgcgtctcaagggtaaagaagggcatttacgcggtaatctgatgggaaagcgtgtagatttctctgctcgtacagtaattacgggtgatccaaatatttcagttgatgaagtcggagttccgaaaagcatagctcaaagtctgacttttccagaattggtgactccctttaatattgactatcttcaaaaattagtagaaaatggcccttctacacatccaggggctaaatatgtaattagagatactggtgaaagaattgatctaaaacatatatcaggcatgactggtggcttaagattacactacggttggaaagttgaacgtcatctcaatgatggtgacatcgttatattcaatcgtcagccatctttgcacaagatgtcgatgatgggacataaagttcgtgttatgctctattcgaccttccgtcttaatttatcagttacaacaccttataacgccgattttgatggtgacgaaatgaacatgcatgttccccaatcagtggaaactaaagcagaaatttcagaaatttgtatggttcctaaacaaattgtatctcctcaatcaaataaacctgttatgggtattgtacaggatactttatgtgctgttagaaaatttacaaaaagggattgctttttatctaaagatttggtaatgaacatt

>Diversispora_densissima_MT724382_MT733211

AGGAATCCCTAGTAAGCATGAGTCATCAGCTCGTG-CTGATTACGTCCCTGCCCTTTGTACACACCGCCCGTCGCTACTACCGATTGAATGGCTTAGTGAGACCTTTGGATTGGGGTTTAGGGATCGGAAAC---GATCCTTATTCTCCGAGAAGTCGGTCAAACTTGGTCATTTAGAGGAAGTAAAAGTCGTAACAAGGTTTCCGTAGGTGAACCTGCGGAAGGATCATT--AAAAAT-TTATA-TCCGGGAATTCG-----TTTCGT----T--------TTCCCGTG------ATTATTTGTATTCAAA-TCCCACTCTTT-------AT-AAAT-ATA---------TTAATTATATAAAAC-AAAAA-A-TAAAAAAGAAAACTTTCAACAACGGATCTCTTGGCTCTCGCATCGATGAAGAACGCAGCGAAATGCGATACGTAATGTGAATTGCAGAATTCCGTGAATCATCGAA-TCTTTGAACACAAATTGTACTTTCCAGTATTCTGGGAAGTATGCTTGGTTGAGGGTCATTAAAATAACA-TTCGTGAA--------------TTTTTTTG-------------CGGATTTGAG----TTTT-CC--AGTA--TTT--------------------------AT-AAAATGTTGGTAACTTT-AAAATT-A-TT------ATTACTTGGTACAAGTT-GAAGACGTTC-TATATGTGTGT-GGTTCGCT-GACAACTTGTCCA--T-C--AT----C-ATAT-ATTATGCG-CGCACTTGG----TTTT----TACGCTCTGTGC--GAGT--ATATA-----TATTTTTTTGTGAC-CTCAGCTCAAGTAAGAATACCC-GCTGAACTTAAGCATATCAATAAGCGGAGGAAAAGAAACTAACAAGGATT-CCCTAGTAACGGCGAGTGAAGAGGGAAAAGCTCAAATTTTAAATCTACC-TGG---TTC--CCAGGTCGAATTGTAATTTGAAGAAGCGATATC-GG-TG-TGGAGGTCTGGTTCAAGTTCTTTGGAACAAGACATCAT-GG-AGGGTGAGAATCCCGTGCATGATCAGA-CC---AAAAT--AC-TTAAT---ATTCGTTTTCTAAGAGTCGAGTTGTTTGGGAATGCAGCTCAAAATGGG-T-GGTAGAC-TTCACCTAAGGCTAAATATCAGCGAGAGACCGATAGCGAACAAGTACTGTG-AA-GGAAAGATGAAAAGAACTTTGAAAAGAGAGTTAAATAGTACGTGAAATTGTTGAAAGGGAAACGATTGAAGTCAGTCATGCTAG-TGAAAAATCAGTTCGACGGG-TTTTG-AGTTCGGGAGT--AGAGGCAGGGT---CAA---ACCGTTTCTCTTTTGGACTTGGGATTTGTCGGACGTACTTTTTCT-TTGGCAGGTCAGCGTCGGTTTC-GGAGGTTGTAAA---------ATACTTGGGGG-AAAGTAGCTCTGCTTCGGGA---GAGTG-TTATAGACCCTGGGAGATGCAGCCTGTGGGATCGAGGATTGCAGCAAATGC--------------------CTTT--GGC-TTGTCGCCT-ATCTCTGG-TCGTTACCTCTCTTGTGACAACATT-CTTGCCACCGGAGGGTACTAATGTCCACTA-CTTAGAGTGATCGAAAA-TTTTGCTAAGGATGCTGACGTAATGGCTTTAAACGACCCGTCTTGAAACACGGACCAAGGAGTCTAACATATGTGCGAG----------------------------------------------------------------------------------------------------------------------------------------------------------------------------------------------------------------------------------------------------------------------------------------------------------------------------------------------------------------------------------------------------------------------------------------------------------------------------------------------------------------------------------------------------------------------------------------------------------------------------------------------------------------------------------------------------------------------------------------------------------------------------------------------------------------------------------------------------------------------------------------------------------------------------------------------------------------------------------------------------------------------------------------------------------------------------------------------------------------------------------------------------------------------------------------------------------

>Diversispora_densissima_MT724383

AGGAATCCCTAGTAAGCATGAGTCATCAGCTCGTG-CTGATTACGTCCCTGCCCTTTGTACACACCGCCCGTCGCTACTACCGATTGAATGGCTTAGTGAGACCTTTGGATTGGGGTTTAGGGATCGGAAAC---GATCCTTATTCTCCGAGAAGTCGGTCAAACTTGGTCATTTAGAGGAAGTAAAAGTCGTAACAAGGTTTCCGTAGGTGAACCTGCGGAAGGATCATT--AAAAAT-TTATA-TCCGGGAATTCG-----TTTCGT----T--------TTCCCGTG------ATTATTTGTATTCAAA-TCCCACTCTTT-------AT-AAAT-ATA---------TTAATTATATAAAAC-AAAAA-A-TAAAAAAGAAAACTTTCAACAACGGATCTCTTGGCTCTCGCATCGATGAAGAACGCAGCGAAATGCGATACGTAATGTGAATTGCAGAATTCCGTGAATCATCGAA-TCTTTGAACACAAATTGTACTTTCCAGTATTCTGGGAAGTATGCTTGGTTGAGGGTCATTAAAATAACA-TTCGTGAA--------------TTTTTTTG-------------CGGATTTGAG----TTTT-CC--AGTA--TTT--------------------------AT-AAAATGTTGGTAACTTT-AAAATT-A-TT------ATTACTTGGTACAAGTT-GAAGACGTTC-TATATGTGTGT-GGTTCGCT-GACAACTTGTCCA--T-C--AT----C-ATAT-ATTATGCG-CGCACTTGG----TTTT----TACGCTCTGTGC--GAGT--ATATA-----TATTTTTTTGTGAC-CTCAGCTCAAGTAAGAATACCC-GCTGAACTTAAGCATATCAATAAGCGGAGGAAAAGAAACTAACAAGGATT-CCCTAGTAACGGCGAGTGAAGAGGGAAAAGCTCAAATTTTAAATCTACC-TGG---TTC--CCAGGTCGAATTGTAATTTGAAGAAGCGATATC-GG-TG-TGGAGGTCTGGTTCAAGTTCTTTGGAACAAGACATCAT-GG-AGGGTGAGAATCCCGTGCATGATCAGA-CC---AAAAT--AC-TTAAT---ATTCGTTTTCTAAGAGTCGAGTTGTTTGGGAATGCAGCTCAAAATGGG-T-GGTAGAC-TTCACCTAAGGCTAAATATCAGCGAGAGACCGATAGCGAACAAGTACTGTG-AA-GGAAAGATGAAAAGAACTTTGAAAAGAGAGTTAAATAGTACGTGAAATTGTTGAAAGGGAAACGATTGAAGTCAGTCATGCTAG-TGAAAAATCAGTTCGACGGG-TTTTG-AGTTCGGGAGT--AGAGGCAGGGT---CAA---ACCGTTTCTCTTTTGGACTTGGGATTTGTCGGACGTACTTTTTCT-TTGGCAGGTCAGCGTCGGTTTC-GGAGGTTGTAAA---------ATACTTGGGGG-AAAGTAGCTCTGCTTCGGGA---GAGTG-TTATAGACCCTGGGAGATGCAGCCTGTGGGATCGAGGATTGCAGCAAATGC--------------------CTTT--GGC-TTGTCGCCT-ATCTCTGG-TCGTTACCTCTCTTGTGACAACATT-CTTGCCACCGGAGGGTACTAATGTCCACTA-CTTAGAGTGATCGAAAA-TTTTGCTAAGGATGCTGACGTAATGGCTTTAAACGACCCGTCTTGAAACACGGACCAAGGAGTCTAACATATGTGCGAG-------------------------------------------------------------------------------------------------------------------------------------cgtaaaacgttatgaagctgatggccatccaccacacgttgtaaacgaatttgaagcattgttacaggttcttaatgat---------------------------------------------------------------------------------------------------------tataa-tttca--------a-ttagtttaatatcaa--ttgaaa--attatacttaaa-tttatcatttat-----atgcaaatagtttcattgtgcaacttatatggacaatgaaatggctggtcaacctcaagctcttcagaaatccggtagacctttaaagtcaatacgtgcgcgtctcaagggtaaagaagggcgtttacgcggtaatctgatgggaaagcgtgtagatttctctgctcgtacagtaattacgggtgatccaaatatttcagttgatgaagtcggagttccgaaaagcatagctcaaaatctgacttttccagaattggtgactccctttaatattgactatcttcaaaaattagtagaaaatggcccttctacacatccgggggctaaatatgtaattagagatactggtgaaagaattgatctaaaacatatatcaggcatgactggtggcttaagattacactacggttggaaagttgaacgtcatctcaatgatggtgacatcgttatattcaatcgtcagccatctttgcacaaaa--------------------------------------------------------------------------------------------------------------------------------------------------------------------------------------------------------------------------------------------------------------------------------------------------

>Diversispora_densissima_MT724384_MT733212

AGGAATCCCTAGTAAGCATGAGTCATCAGCTCGTG-CTGATTACGTCCCTGCCCTTTGTACACACCGCCCGTCGCTACTACCGATTGAATGGCTTAGTGAGACCTTTGGATTGGGGTTTAGGGATCGGAAAC---GATCCTTATTCTCCGAGAAGTCGGTCAAACTTGGTCATTTAGAGGAAGTAAAAGTCGTAACAAGGTTTCCGTAGGTGAACCTGCGGAAGGATCATT--AAAAAT-TTATA-TCCGGGAATTCG-----TTTCGT----T--------TTCCCGTG------ATTATTTGTATTCAAA-TCCCACTCTTT-------AT-AAAT-ATA---------TTAATTATATAAAAC--AAAA-A-TAAAAAAGAAAACTTTCAACAACGGATCTCTTGGCTCTCGCATCGATGAAGAACGCAGCGAAATGCGATACGTAGTGTGAATTGCAGAATTCCGTGAATCATCGAA-TCTTTGAACACAAATTGTACTTTCCAGTATTCTGGGAAGTATGCTTGGTTGAGGGTCATTAAAATAACA-TTCGTGAA---------------TTTTTTG-------------CGGATTTGAG----TTTT-CC--AGTA--TTT--------TAT---------------TA-AAAATGTTGGTAACTTT-AAAATT-A-TT------ATTACTTGGTTCAAGTT-GAAGACGTTC-TATA--TGTGT-GGTTCGCT-GACAACTTGTCCA--T-C--TT----T-ATAT-ATTATGCG-CGCACTTGT----TTTT----AATGCTTTGTGT--GAGC--ATATA-----TTTTTTTTTATGAC-CTCAGCTCAAGTAAGAATACCC-GCTGAACTTAAGCATATCAATAAGCGGAGGAAAAGAAACTAACAAGGATTCCCCTAGTAACGGCGAGTGAAGAGGGAAAAGCTCAAATTTTAAATCTACC-TGG---TTC--CCAGGTCGAATTGTAATTTGAAGAAGCGATATC-GG-TG-TGGAGGTCTGGTTCAAGTTCTTTGGAACAAGACATCAT-GG-AGGGTGAGAATCCCGTGCATGATCAGA-CC---AAAAT--AC-TTAAT---ATTCGCTTTCTAAGAGTCGAGTTGTTTGGGAATGCAGCTCAAAATGGG-T-GGTAGAC-TTCACCTAAGGCTAAATATCAGCGAGAGACCGATAGCGAACAAGTACTGTG-AA-GGAAAGATGAAAAGAACTTTGAAAAGAGAGTTAAATAGTACGTGAAATTGTTGAAAGGGAAACGATTGAAGTCAGTCATGCTAG-TGAAAAATCAGTTCGACGGG-TTTTG-AGTTCGGGAGT--AGAGGCAGGGT---CAA---ACCGTTTCTCTTTTGGACTTGGGATTTGTCGGACGTACTTTTTCT-TTGGCAGGTCAGCGTCGGTTTC-GGAGGTTGTAAA---------ATACTTGGGGG-AAAGTAGCTCTGCTTCGGGA---GAGTG-TTATAGACCCTGGGAGATGCAGCCTGTGGGATCGAGGATTGCAGCAAATGC--------------------CTTT--GGC-TTGTCGCCT-ATCTCTGG-TCGTTACCTCTCTTGTGACAACATT-CTTGCCACCGGAGGGTACTAATGTCCACTA-CTTAGAGTGATCGAAAA-TTTTGCTAAGGATGCTGACGTAATGGCTTTAAACGACCCGTCTTGAAACACGGACCAAGGAGTCTAACATATGTGCGAG---------------------------------------------------------------------------------------------------------------------------------aaaacgtaaaacgttatgaagctgatggccatccaccacacgttgtaaacgaatttgaagcattgttacaggttcttaatgat---------------------------------------------------------------------------------------------------------tataa-tttca--------a-ttagtttaatatcaa--ttgaaa--attatacttaaa-tttatcatttat-----atgcaaatagtttcattgtgcaacttatatggacaatgaaatggctggtcaacctcaagctcttcagaaatccggtagacctttaaagtcaatacgtgcgcgtctcaagggtaaagaagggcgtttacgcggtaatctgatgggaaagcgtgtagatttctctgctcgtacagtaattacgggtgatccaaatatttcagttgatgaagtcggagttccgaaaagcatagctcaaaatctgacttttccagaattggtgactccctttaatattgactatcttcaaaaattagtagaaaatggcccttctacacatccgggggctaaatatgtaattagagatactggtgaaagaattgatctaaaacatatatcaggcatgactggtggcttaagattacactacggttggaaagttgaacgtcatctcaatgatggtgacatcgttatattcaatcgtcagccatctttgcacaaaatgt-----------------------------------------------------------------------------------------------------------------------------------------------------------------------------------------------------------------------------------------------------------------------------------------------

>Diversispora_marina_MT725498

AGGAATCCCTAGTAAGCATGAGTCATCAGCTCATG-TTGATTACGTCCCTGCCCTTTGTACACACCGCCCGTCGCTACTACCGATTGAATGGCTTAGTGAGACCTTTGGATTGGGGTTTAGGGATCGGAAAC---GATTCTTTTTTTCCGAGAAGTCGGTCAAACTTGGTCATTTAGAGGAAGTAAAAGTCGTAACAAGGTTTCCGTAGGTGAACCTGCGGAAGGATCATT--AAAAAT-TTATATTCCGGGAATTCG-----TTTCGT----T--------TTCCCG-G------ATTATTTGTATTCAAA-TCCCACTCTTT-------AT-AAAT-ATA---------TTAATTATATAAAAC--AAAA-T-AAAAAAAGAAAACTTTCAACAACGGATCTCTTGGCTCTCGCATCGATGAAGAACGCAGCGAAATGCGATACGTAGTGTGAATTGCAGAATTCCGTGAATCATCGAA-TCTTTGAACACAAATTGTACTTTCCAGTAATCTGGGAAGTATGCTTGGTTGAGGGTCATTAAAATAACA-TTCGTGAA--------------TTTTTTTG-------------CGGATTTGAG----TTTT-CC--AGCA--TTT-----------------A--------TA-AAAATGTTGGTAACTTT-AAAATT-TATT------ATTACTTGGTACAAGTT-GAAAACGTTC-TATATGTGTGT-GGTTCGCT-GACAACTTGTCCA--T-C--TT----T-ATAT-ATTATGCG-CGCACTTGT----TTTT----TTGGCTTCGTGC--GAGT--ATATA------TTTTTTTTATGAC-CTCAGCTCAAGTAAGAATACCC-GCTGAACTTAAGCATATCAATAAGCGGAGGAAAAGAAACTAACAAGGATTCCCCTAGTAACGGCGAGTGAAGAGGGAAAAGCTCAAATTTTAAATCTACC-TGG---TTC--CCAGGTCGAATTGTAATTTGAAGAATCGATATC-GGTTG-TGGAGGTCTGGTTCAAGTTCTTTGGAACAAGACATCAT-GGAAGGGTGAGAATCCCGTGCATGATCAGA-CC---AAAAT--AC-CTAAT---ATTCGTTTTCTAAGAGTCGAGTTGTTTGGGAATGCAGCTCAAAATGGG-T-GGTAGAC-TTCACCTAAGGCTAAATATCAGCGAGAGACCGATAGCGAACAAGTACTGTG-AA-GGAAAGATGAAAAGAACTTTGAAAAGAGAGTTAAATAGTACGTGAAATTGTTGAAAGGGAAACGATTGAAGTCAGTCATGCTAG-TGAAAAATCAGTTTGACGGG-TTTTG-AGTTCGGGAGT--AGAGGCAGGGT---CAA---ACCGTTTCTCTTTTGAACTTGGGATTTGTTAGATGTACTTTTTCT-TTGGCAGGTCAGCGTCGATTTC-GGAGGTTGTAAA---------ATACTTGGGGG-AAAGTAACTCTGCTTCGGGA---GAGTA-TTATAGACCCTGGGGGATGCAGCCTGTGGGATCGAGGATTGCAGCAAATGC--------------------CTTT--GGC-TTGTCGCCTGATCTCTGG-ATGTTACCTCGCTTTTGACAACATT-CTTGCCACTTGTGGGTACTAATGCCCATCAGGTTAGAGCGATCAAAAA-TTTTGCTAAGGATGCTGACGTAATGGCTTTAAACGACCCGTCTTGAAACACGGACCAAGGAGTCTAACATATATGCGAG------------------------------------------------------------------------------gtggagaagatgatttgacacacaaattatccgacatcttgaaggcaaatcaaaacgtaaaacgttatgaagctgatggtcatccaccacacgttgtaaacgaatttgaagcattgttacaggttcttaatgat---------------------------------------------------------------------------------------------------------tataa-tttca--------a-ttagtttaatatcaa--ttgaaa--attatacttaaa-tttattatttat-----atgcaaatagtttcattgtgcaacttatatggacaatgaaatggctggtcaacctcaagctcttcagaaatctggtagacctttaaagtcaatacgtgcgcgtctcaagggtaaagaagggcgtttacgcggtaatctgatgggaaagcgtgtagatttctctgctcgtacagtaattacgggtgatccaaatatttcagttgatgaagtcggggttccgaaaagcatagctcaaaatctgacttttccggaattggtgactccctttaatattgactatcttcaaaaattagtagaaaatggcccttctacacatccgggggctaaatatgtaattagagatactggtgaaagaattgatctaaaacatatatcaggcatgactggtggcttaagattacactacggttggaaagttgaacgtcatctcaatgatggtgacatcgttatattcaatcgtcagccntctttgcacaaaatgtcaatgatggg-------------------------------------------------------------------------------------------------------------------------------------------------------------------------------------------------------------------------------------------------------------------------------------

>Diversispora_marina_MT725499

AGGAATCCCTAGTAAGCATGAGTCATCAGCTCATG-TTGATTACGTCCCTGCCCTTTGTACACACCGCCCGTCGCTACTACCGATTGAATGGCTTAGTGAGACCTTTGGATTGGGGTTTAGGGATCGGAAAC---GATTCTTTTTTTCCGAGAAGTCGGTCAAACTTGGTCATTTAGAGGAAGTAAAAGTCGTAACAAGGTTTCCGTAGGTGAACCTGCGGAAGGATCATT--AAAAAT-TTATATTCCGGGAATTCG-----TTTCGT----T--------TTCCCG-G------ATTATTTGTATTCAAA-TCCCACTCTTT-------AT-AAAT-ATA---------TTAATTATATAAAAC--AAAA-T-AAAAAAAGAAAACTTTCAACAACGGATCTCTTGGCTCTCGCATCGATGAAGAACGCAGCGAAATGCGATACGTAGTGTGAATTGCAGAATTCCGTGAATCATCGAA-TCTTTGAACACAAATTGTACTTTCCAGTAATCTGGGAAGTATGCTTGGTTGAGGGTCATTAAAATAACA-TTCGTGAA--------------TTTTTTTG-------------CGGATTTGAG----TTTT-CC--AGCA--TTT-----------------A--------TA-AAAATGTTGGTAACTTT-AAAATT-TATT------ATTACTTGGTACAAGTT-GAAAACGTTC-TATATGTGTGT-GGTTCGCT-GACAACTTGTCCA--T-C--TT----T-ATAT-ATTATGCG-CGCACTTGT----TTTT----TTGGCTTCGTGC--GAGT--ATATA------TTTTTTTTATGAC-CTCAGCTCAAGTAAGAATACCC-GCTGAACTTAAGCATATCAATAAGCGGAGGAAAAGAAACTAACAAGGATTCCCCTAGTAACGGCGAGTGAAGAGGGAAAAGCTCAAATTTTAAATCTACC-TGG---TTC--CCAGGTCGAATTGTAATTTGAAGAATCGATATC-GG-TG-TGGAGGTCTGGTTCAAGTTCTTTGGAACAAGACATCAT-GGAAGGGTGAGAATCCCGTGCATGATCAGA-CC---AAAAT--AC-CTAAT---ATTCGTTTTCTAAGAGTCGAGTTGTTTGGGAATGCAGCTCAAAATGGG-T-GGTAGAC-TTCACCTAAGGCTAAATATCAGCGAGAGACCGATAGCGAACAAGTACTGTG-AA-GGAAAGATGAAAAGAACTTTGAAAAGAGAGTTAAATAGTACGTGAAATTGTTGAAAGGGAAACGATTGAAGTCAGTCATGCTAG-TGAAAAATCAGTTTGACGGG-TTTTG-AGTTCGGGAGT--AGAGGCAGGGT---CAA---ACCGTTTCTCTTTTGAACTTGGGATTTGTTAGATGTACTTTTTCT-TTGGCAGGTCAGCGTCGATTTC-GGAGGTTGTAAA---------ATACTTGGGGG-AAAGTAACTCTGCTTCGGGA---GAGTA-TTATAGACCCTGGGGGATGCAGCCTGTGGGATCGAGGATTGCAGCAAATGC--------------------CTTT--GGC-TTGTCGCCTGATCTCTGG-ATGTTACCTCGCTTTTGACAACATT-CTTGCCACTTGTGGGTACTAATGCCCATCAGGTTAGAGCGATCAAAAA-TTTTGCTAAGGATGCTGACGTAATGGCTTTAAACGACCCGTCTTGAAACACGGACCAAGGAGTCTAACATATATGCGAG----------------------------------------------------------------------------------------------------------------------------------------------------------------------------------------------------------------------------------------------------------------------------------------------------------------------------------------------------------------------------------------------------------------------------------------------------------------------------------------------------------------------------------------------------------------------------------------------------------------------------------------------------------------------------------------------------------------------------------------------------------------------------------------------------------------------------------------------------------------------------------------------------------------------------------------------------------------------------------------------------------------------------------------------------------------------------------------------------------------------------------------------------------------------------------------------------------

>Diversispora_marina_MT725501

AGGAATCCCTAGTAAGCATGAGTCATCAGCTCATG-TTGATTACGTCCCTGCCCTTTGTACACACCGCCCGTCGCTACTACCGATTGAATGGCTTAGTGAGACCTTTGGATTGGGGTTTAGGGATCGGAAAC---GATTCTTTTTTTCCGAGAAGTCGGTCAAACTTGGTCATTTAGAGGAAGTAAAAGTCGTAACAAGGTTTCCGTAGGTGAACCTGCGGAAGGATCATT--AAAAAT-TTATATTCCGGGAATTCG-----TTTCGT----T--------TTCCCG-G------ATTATTTGTATTCAAA-TCCCACTCTTT-------AT-AAAT-ATA---------TTAATTATATAAAAC--AAAA-T-AAAAAAAGAAAACTTTCAACAACGGATCTCTTGGCTCTCGCATCGATGAAGAACGCAGCGAAATGCGATACGTAGTGTGAATTGCAGAATTCCGTGAATCATCGAA-TCTTTGAACACAAATTGTACTTTCCAGTAATCTGGGAAGTATGCTTGGTTGAGGGTCATTAAAATAACA-TTCGTGAA--------------TTTTTTTG-------------CGGATTTGAG----TTTT-CC--AGCA--TTT-----------------A--------TA-AAAATGTTGGTAACTTT-AAAATT-A-TT------ATTACTTGGTACAAGTT-GAAAACGTTC-TATATGTGTGT-GGTTCGCT-GACAACTTGTCCA--T-C--TT----T-ATAT-ATTATGCG-CGCACTTGT----TTTT----TTGGCTTCGTGC--GAGT--ATATA------TTTTTTTTATGAC-CTCAGCTCAAGTAAGAATACCC-GCTGAACTTAAGCATATCAATAAGCGGAGGAAAAGAAACTAACAAGGATTCCCCTAGTAACGGCGAGTGAAGAGGGAAAAGCTCAAATTTTAAATCTACC-TGG---TTC--CCAGGTCGAATTGTAATTTGAAGAATCGATATCGGG-TG-TGGAGGTCTGGTTCAAGTTCTTTGGAACAAGACATCAT-GGAAGGGTGAGAATCCCGTGCATGATCAGA-CC---AAAAT--AC-CTAAT---ATTCGTTTTCTAAGAGTCGAGTTGTTTGGGAATGCAGCTCAAAATGGG-T-GGTAGAC-TTCACCTAAGGCTAAATATCAGCGAGAGACCGATAGCGAACAAGTACTGTG-AA-GGAAAGATGAAAAGAACTTTGAAAAGAGAGTTAAATAGTACGTGAAATTGTTGAAAGGGAAACGATTGAAGTCAGTCATGCTAG-TGAAAAATCAGTTTGACGGG-TTTTG-AGTTCGGGAGT--AGAGGCAGGGT---CAA---ACCGTTTCTCTTTTGAACTTGGGATTTGTTAGATGTACTTTTTCT-TTGGCAGGTCAGCGTCGATTTC-GGAGGTTGTAAA---------ATACTTGGGGG-AAAGTAACTCTGCTTCGGGA---GAGTA-TTATAGACCCTGGGGGATGCAGCCTGTGGGATCGAGGATTGCAGCAAATGC--------------------CTTT--GGC-TTGTCGCCTGATCTCTGG-ATGTTACCTCGCTTTTGACAACATT-CTTGCCACTTGTGGGTACTAATGCCCATCAGGTTAGAGCGATCAAAAA-TTTTGCTAAGGATGCTGACGTAATGGCTTTAAACGACCCGTCTTGAAACACGGACCAAGGAGTCTAGCATATATGCGAG----------------------------------------------------------------------------------------------------------------------------------------------------------------------------------------------------------------------------------------------------------------------------------------------------------------------------------------------------------------------------------------------------------------------------------------------------------------------------------------------------------------------------------------------------------------------------------------------------------------------------------------------------------------------------------------------------------------------------------------------------------------------------------------------------------------------------------------------------------------------------------------------------------------------------------------------------------------------------------------------------------------------------------------------------------------------------------------------------------------------------------------------------------------------------------------------------------

>Diversispora_marina_MT725502

AGGAATCCCTAGTAAGCATGAGTCATCAGCTCATG-TTGATTACGTCCCTGCCCTTTGTACACACCGCCCGTCGCTACTACCGATTGAATGGCTTAGTGAGACCTTTGGATTGGGGTTTAGGGATCGGAAAC---GATTCTTTTTTTCCGAGAAGTCGGTCAAACTTGGTCATTTAGAGGAAGTAAAAGTCGTAACAAGGTTTCCGTAGGTGAACCTGCGGAAGGATCATT--AAAAAT-TTATATTCCGGGAATTCG-----TTTCGT----T--------TTCCCG-G------ATTATTTGTATTCAAA-TCCCACTCTTT-------AT-AAAT-ATA---------TTAATTATATAAAAC--AAAA-T-AAAAAAAGAAAACTTTCAACAACGGATCTCTTGGCTCTCGCATCGATGAAGAACGCAGCGAAATGCGATACGTAGTGTGAATTGCAGAATTCCGTGAATCATCGAA-TCTTTGAACACAAATTGTACTTTCCAGTAATCTGGGAAGTATGCTTGGTTGAGGGTCATTAAAATAACATTTCGTGAA--------------TTTTTTTG-------------CGGATTTGAG----TTTT-CC--AGCA--TTT-----------------A--------TA-AAAATGTTGGTAACTTT-AAAATT-A-TT------ATTACTTGGTACAAGTT-GAAAACGTTC-TATATGTGTGT-GGTTCGCT-GACAACTTGTCCA--T-C--TT----T-ATAT-ATTATGCG-CGCACTTGT----TTTT----TTGGCTTCGTGC--GAGT--ATATA------TTTTTTTTATGAC-CTCAGCTCAAGTAAGAATACCC-GCTGAACTTAAGCATATCAATAAGCGGAGGAAAAGAAACTAACAAGGATTCCCCTAGTAACGGCGAGTGAAGAGGGAAAAGCTCAAATTTTAAATCTACC-TGG---TTC--CCAGGTCGAATTGTAATTTGAAGAATCGATATC-GG-TG-TGGAGGTCTGGTTCAAGTTCTTTGGAACAAGACATCAT-GG-AGGGTGAGAATCCCGTGCATGATCAGA-CC---AAAAT--AC-CTAAT---ATTCGTTTTCTAAGAGTCGAGTTGTTTGGGAATGCAGCTCAAAATGGG-T-GGTAGAC-TTCACCTAAGGCTAAATATCAGCGAGAGACCGATAGCGAACAAGTACTGTG-AA-GGAAAGATGAAAAGAACTTTGAAAAGAGAGTTAAATAGTACGTGAAATTGTTGAAAGGGAAACGATTGAAGTCAGTCATGCTAG-TGAAAAATCAGTTTGACGGG-TTTTG-AGTTCGGGAGT--AGAGGCAGGGT---CAA---ACCGTTTCTCTTTTGAACTTGGGATTTGTTAGATGTACTTTTTCT-TTGGCAGGTCAGCGTCGATTTC-GGAGGTTGTAAA---------ATACTTGGGGG-AAAGTAACTCTGCTTCGGGA---GAGTA-TTATAGACCCTGGGGGATGCAGCCTGTGGGATCGAGGATTGCAGCAAATGC--------------------CTTT--GGC-TTGTCGCCTGATCTCTGG-ATGTTACCTCGCTTTTGACAACATT-CTTGCCACTTGTGGGTACTAATGCCCATCAGGTTAGAGCGATCAAAAA-TTTTGCTAAGGATGCTGACGTAATGGCTTTAAACGACCCGTCTTGAAACACGGACCAAGGAGTCTAGCATATATGCGAG----------------------------------------------------------------------------------------------------------------------------------------------------------------------------------------------------------------------------------------------------------------------------------------------------------------------------------------------------------------------------------------------------------------------------------------------------------------------------------------------------------------------------------------------------------------------------------------------------------------------------------------------------------------------------------------------------------------------------------------------------------------------------------------------------------------------------------------------------------------------------------------------------------------------------------------------------------------------------------------------------------------------------------------------------------------------------------------------------------------------------------------------------------------------------------------------------------

>Diversispora_insculpta_KJ850195_OL690413

AGGAATCCCTAGTAAGCATGAGTCATCAGCTCATG-CTGATTACGTCCCTGCCCTTTGTACACACCGCCCGTCGCTACTACCGATTGAATGGCTTAGTGAGACCTTTGGATTGGGGTTTAGGGATCGGAAAC---GATTCTTATTCTCCGAGAAGTCGGTCAAACTTGGTCATTTAGAGGAAGTAAAAGTCGTAACAAGGTTTCCATAGGTGAACCTGCGGAAGGATCATT--AAAAAT-TTTTA-TCCGGGAATTCG-----ATTCGT----TTCG-----TTTCCCGG------ATTATTTGTATTCAAA-TCCCACTCTTT-------AT-AAAT-ATA---------TTAATTATATAAAAC---AAA-A-TAAAAAAGAAAACTTTCAACAACGGATCTCTTGGCTCTCGCATCGATGAAGAACGCAGCGAAATGCGATACGTAGTGTGAATTGCAGAATTCCGTGAATCATCGAA-TCTTTGAACGCAAATTGTACTTTCCAGTAATCTGGGAAGTATGCTTGGTTGAGGGTCATAATAATAACA-TTCGTGAA--------------TTTTTTCG-------------CGGATTTGAG----TTTT-CC--AGTA--TTT-TTAT---AAT---------------AT-AAAATGTTGGTAACTTT-AAAATT-ATTT------ATTACTTGGTACAAGTT-GAAAACGTGC-TATA----TGT-GGTTCGCT-GACAACTTGTCCA--T-C--TT----C-ATAT-ATTATGCG-CGCACTTGG----TTTT----TACGCTCTGTGC--GAGT--ATATA-----TTTTTTTTTATGAC-CTCAGCTCAAGCAAGAATACCC-GCTGAACTTAAGCATATCAATAAGCGGAGGAAAAGAAACTAACAAGGATTCCCCTAGTAACGGCGAGTGAAGAGGGAAAAGCTCAAATTTTAAATCTACC-TGG---TTC--CCAGGTCGAATTGTAATTTGAAGAAGCGATATC-GG-TT-TTGAGGTCTGGTTTAAGTTCTTTGGAACAAGACATCATGGG-AGGGTGAGAATCCCGTGCATGATCAGA-CC---AAGAT--AC-CCAAT---ATTCGTTTTCTAAGAGTCGAGTTGTTTGGGAATGCAGCTCAAAATGGG-T-GGTAGAC-TTCACCTAAGGCTAAATATCAGCGAGAGACCGATAGCGAACAAGTACTGTG-AA-GGAAAGATGAAAAGAACTTTGAAAAGAGAGTTAAATAGTACGTGAAATTGTTGAAAGGGAAACGATTGAAGTCAGTCATGCTAG-TGAAAAATCAGTTTAACGGG-TTTTG-AGTTCTTGAGT--AGAGGCAGGGT---CAA---ACCGTTTCTCTTTTGGACTTGAGATTTGTTGGACGTACTTTTTCT-TTGGCAGGTCAACGTCGGTTTC-GGGGGTTGTAAA---------ATACTTGGGGG-AAAGTAGCTCTGCTTCGGGA---GAGTG-TTATAGACCCTAGGAGATGCAGCCTGCGGGATCGAGGATTGCAGCAAATGC-------------------CTTCT--GGC-TTGTCGCCTGATCTCTGG-TTGTTACCTCTCTTGTGACAACATT-CTTGTCACCGGAGGGTACTAATGCCCATTAGGTTAGAGCGATCAAAAA-TTTTGCTAAGGATGTTGACGTAATGGCTTTAAACGACCCGTCTTGAAACACGGACCAAGGAGTCTAACATATGTGCGAGctgattggatgatcataactatacttcccgttcctcctcctcctgtacgtcccagtatacaaatggacggaacaagtcgaggtgaagatgatttgactcacaaattatccgacatcttgaaggcaaatcaaaacgtaaaacgttatgaagctggtggtcatccaccacacgttgtaaacgaatttgaagcattattacaggttcttaatgat---------------------------------------------------------------------------------------------------------tataa-tttca--------a-ttagtttaatatcaa--ttgaag--tttatacttaga-tttattatttat-----atgcaaacagtttcattgtgcaacttatatggacaatgaaatggctggtcaacctcaagctcttcagaaatctggtagacctttaaagtcaatacgtgcgcgtctcaagggtaaagaagggcgtttacgcggtaatctgatgggaaagcgtgtagatttctctgctcgtacagtaattacgggtgatccaaatatctcagttgatgaagtcggagttccgaaaagcatagctcaaaatttaacttttccagaattggtgactccctttaatattgactatctccaaaaattagtagaaaatggcccttctgcacatccgggggctaaatacgtaattagagatactggtgaaagaatagatctaaaacatatatcaggtatgactggtggcttaagattacactacggttggaaagttgaacgtcatctcaatgatggtgacatcgttatattcaatcgtcagccatctttgcacaagatgtcgatgatgggacataaagttcgtgttatgccctattcgaccttccgtcttaatttatcagttacaacaccttataacgccgattttgatggtgacgaaatgaacatgcatgttccccaatcagtggaaactaaagcagaaatttcagaaatccgtatggttcctaaacagattgtatctcctcaatcaaataaacctgttatgggtattgtacaggatactttatgtgctgttagaaaatttacaaaaagggattgctttttatctaaagatttggtaatgaacatt

>Diversispora_insculpta_KJ850196

AGGAATCCCTAGTAAGCATGAGTCATCAGCTCATG-CTGATTACGTCCCTGCCCTTTGTACACACCGCCCGTCGCTACTACCGATTGAATGGCTTAGTGAGACCTTTGGATTGGGGTTTAGGGATCGGAAAC---GATTCTTATTCTCCGAGAAGTCGGTCAAACTTGGTCATTTAGAGGAAGTAAAAGTCGTAACAAGGTTTCCATAGGTGAACCTGCGGAAGGATCATT--AAAAAT-TTTTA-TCCGGGAATTCG-----ATTCGT----TTCG-----TTTCCCGG------ATTATTTGTATTCAAA-TCCCACTCTTT-------AT-AAAT-ATA---------TTAATTATATAAAAC---AAA-A-TAAAAAAGAAAACTTTCAACAACGGATCTCTTGGCTCTCGCATCGATGAAGAACGCAGCGAAATGCGATACGTAGTGTGAATTGCAGAATTCCGTGAATCATCGAA-TCTTTGAACGCAAATTGTACTTTCCAGTAATCTGGGAAGTATGCTTGGTTGAGGGTCATAATAATAACA-TTCGTGAA--------------TTTTTTCG-------------CGGATTTGAG----TTTT-CC--AGTA--TTT-TTAT---AAT---------------AT-AAAATGTTGGTAACTTT-AAAATT-ATTT------ATTACTTGGTACAAGTT-GAAAACGTGC-TATA----TGT-GGTTCGCT-GACAACTTGTCCA--T-C--TT----C-ATAT-ATTATGCG-CGCACTTGG----TTTT----TACGCTCTGTGC--GAGT--ATATA------TTTTTTTTATGAC-CTCAGCTCAAGCAAGAATACCC-GCTGAACTTAAGCATATCAATAAGCGGAGGAAAAGAAACTAACAAGGATTCCCCTAGTAACGGCGAGTGAAGAGGGAAAAGCTCAAATTTTAAATCTACC-TGG---TTC--CCAGGTCGAATTGTAATTTGAAGAAGCGATATC-GG-TT-TTGAGGTCTGGTTTAAGTTCTTTGGAACAAGACATCATGGG-AGGGTGAGAATCCCGTGCATGATCAGA-CC---AAGAT--AC-CCAAT---ATTCGTTTTCTAAGAGTCGAGTTGTTTGGGAATGCAGCTCAAAATGGG-T-GGTAGAC-TTCACCTAAGGCTAAATATCAGCGAGAGACCGATAGCGAACAAGTACTGTG-AA-GGAAAGATGAAAAGAACTTTGAAAAGAGAGTTAAATAGTACGTGAAATTGTTGAAAGGGAAACGATTGAAGTCAGTCATGCTAG-TGAAAAATCAGTTTAACGGG-TTTTG-AGTTCTTGAGT--AGAGGCAGGGT---CAA---ACCGTTTCTCTTTTGGACTTGAGATTTGTTGGACGTACTTTTTCT-TTGGCAGGTCAACGTCGGTTTC-GGGGGTTGTAAA---------ATACTTGGGGG-AAAGTAGCTCTGCTTCGGGA---GAGTG-TTATAGACCCTAGGAGATGCAGCCTGCGGGATCGAGGATTGCAGCAAATGC-------------------CTTCT--GGC-TTGTCGCCTGATCTCTGG-TTGTTACCTCTCTTGTGACAACATT-CTTGTCACCGGAGGGTACTAATGCCCATTAGGTTAGAGCGATCAAAAA-TTTTGCTAAGGATGTTGACGTAATGGCTTTAAACGACCCGTCTTGAAACACGGACCAAGGAGTCTAACATATGTGCGAG----------------------------------------------------------------------------------------------------------------------------------------------------------------------------------------------------------------------------------------------------------------------------------------------------------------------------------------------------------------------------------------------------------------------------------------------------------------------------------------------------------------------------------------------------------------------------------------------------------------------------------------------------------------------------------------------------------------------------------------------------------------------------------------------------------------------------------------------------------------------------------------------------------------------------------------------------------------------------------------------------------------------------------------------------------------------------------------------------------------------------------------------------------------------------------------------------------

>Diversispora_insculpta_KJ850197_OL690414

AGGAATCCCTAGTAAGCATGAGTCATCAGCTCATG-CTGATTACGTCCCTGCCCTTTGTACACACCGCCCGTCGCTACTACCGATTGAATGGCTTAGTGAGACCTTTGGATTGGGGTTTAGGGATCGGAAAC---GATTCTTATTCTCCGAGAAGTCGGTCAAACTTGGTCATTTAGAGGAAGTAAAAGTCGTAACAAGGTTTCCATAGGTGAACCTGCGGAAGGATCATT--AAAAAT-TTTTA-TCCGGGAATTCG-----ATTCGT----TTCG-----TTTCCCGG------ATTATTTGTATTCAAA-TCCCACTCTTT-------AT-AAAT-ATA---------TTAATTATATAAAAC---AAA-A-TAAAAAAGAAAACTTTCAACAACGGATCTCTTGGCTCTCGCATCGATGAAGAACGCAGCGAAATGCGATACGTAGTGTGAATTGCAGAATTCCGTGAATCATCGAA-TCTTTGAACGCAAATTGTACTTTCCAGTAATCTGGGAAGTATGCTTGGTTGAGGGTCATAATAATAACA-TTCGTGAA--------------TTTTTTCG-------------CGGATTTGAG----TTTT-CC--AGTA--TTT-TTAT---AAT---------------AT-AAAATGTTGGTAACTTT-AAAATT-ATTT------ATTACTTGGTACAAGTT-GAAAACGTGC-TATA----TGT-GGTTCGCT-GACAACTTGTCCA--T-C--TT----CAATAT-ATTATGCG-CGCACTTGG----TTTT----TACGCTCTGTGC--GAGT--ATATA------TTTTTTTTATGAC-CTCAGCTCAAGCAAGAATACCC-GCTGAACTTAAGCATATCAATAAGCGGAGGAAAAGAAACTAACAAGGATTCCCCTAGTAACGGCGAGTGAAGAGGGAAAAGCTCAAATTTTAAATCTACC-TGG---TTC--CCAGGTCGAATTGTAATTTGAAGAAGCGATATC-GG-TT-TTGAGGTCTGGTTTAAGTTCTTTGGAACAAGACATCAT-GG-AGGGTGAGAATCCCGTGCATGATCAGA-CC---AAGAT--AC-CCAAT---ATTCGTTTTCTAAGAGTCGAGTTGTTTGGGAATGCAGCTCAAAATGGG-TGGGTAGAC-TTCACCTAAGGCTAAATATCAGCGAGAGACCGATAGCGAACAAGTACTGTG-AA-GGAAAGATGAAAAGAACTTTGAAAAGAGAGTTAAATAGTACGTGAAATTGTTGAAAGGGAAACGATTGAAGTCAGTCATGCTAG-TGAAAAATCAGTTTAACGGG-TTTTG-AGTTCTTGAGT--AGAGGCAGGGT---CAA---ACCGTTTCTCTTTTGGACTTGAGATTTGTTGGACGTACTTTTTCT-TTGGCAGGTCAACGTCGGTTTC-GGGGGTTGTAAA---------ATACTTGGGGG-AAAGTAGCTCTGCTTCGGGA---GAGTG-TTATAGACCCTAGGAGATGCAGCCTGCGGGATCGAGGATTGCAGCAAATGC-------------------CTTCT--GGC-TTGTCGCCTGATCTCTGG-TTGTTACCTCTCTTGTGACAACATT-CTTGTCACCGGAGGGTACTAATGCCCATTAGGTTAGAGCGATCAAAAA-TTTTGCTAAGGATGTTGACGTAATGGCTTTAAACGACCCGTCTTGAAACACGGACCAAGGAGTCTAACATATGTGCGAGctgattggatgatcataactatacttcccgttcctcctcctcctgtacgtcccagtatacaaatggacggaacaagtcgaggtgaagatgatttgactcacaaattatccgacatcttgaaggcaaatcaaaacgtaaaacgttatgaagctgatggtcatccaccacacgttgtaaacgaatttgaagcattattacaggttcttaatgat---------------------------------------------------------------------------------------------------------tataa-tttca--------a-ttagtttaataccaa--ttgaag--tttatacttaaa-tttattatttat-----atgcaaacagtttcattgtgcaacttatatggacaatgaaatggctggtcaacctcaagctcttcagaaatctggtagacctttaaagtcaatacgtgcgcgtctcaagggtaaagaagggcgtttacgcggtaatctgatgggaaagcgtgtagatttctctgctcgtacagtaattacgggtgatccaaatatctcagttgatgaagtcggagttccgaaaagcatagctcaaaatttaacttttccagaattggtgactccctttaatattgactatctccaaaaattagtagaaagtggcccttctacacatccgggggctaaatacgtaattagagatactggtgaaagaatagatctaaaacatatatcaggcatgactggtggcttaagattacactacggttggnaagttgaacgtcatctcaatgatggtgacatcgttatattcaatcgtcagccatctttgcacaagatgtcgatgatgggacataaagttcgtgttatgccctattcgaccttccgtcttaatttatcagttacaacaccttataacgccgattttgatggtgacgaaatgaacatgcatgttccccaatcagtggaaactaaagcagaaatttcagaaatctgtatggttcctaaacaaattgtatctcctcaatcaaataaacctgttatgggtattgtacaggatactttatgtgctgttagaaaatttacaaaaagggattgctttttatctaaagatttggtaatgaacatt

>Diversispora_varaderana_KT444708_MG459202

AGGAATCCCTAGTAAGCATGAGTCATCAGCTCATG-TTGATTACGTCCCTGCCCTTTGTACACACCGCCCGTCGCTACTACCGATTGAATGGCTTAGTGAGACCTTTGGATTAGGGTTTAGGGATCGGAAAC---GATTCTTATTCTCCGAGAAGTCGGTCAAACTTGGTCATTTAGAGGAAGTAAAAGTCGTAACAAGGTTTCCGTAGGTGAACCTGCGGAAGGATCATT--AAAAAT-TTTTA-TCCGGGAATTCGATTCGTTTCGT----T--------TTCCCG-G------ATTATTTGTATTCAAA-TTCCACTCTTT-------AT-AAAT-ATA---------TTAATTATATAAAAC--AAAA-A-TAAAAAAGAAAACTTTCAACAACGGATCTCTTGGCTCTCGCATCGATGAAGAACGCAGCGAAATGCGATACGTAGTGTGAATTGCAGAATTCCGTGAATCATCGAA-TCTTTGAACGCAAATTGTACTTTCCAGTAATCTGGGAAGTATGCTTGGTTGAGGGTCATTAAAATAACA-TTCGTGAA--------------TTTTTTCG-------------CGGATTTGAG----TTTT-CC--AGTA--TTT-TTAT---TAT------A--------AA-AAAATGTTGGTAACTTT-AAAATT-ATTT------ATTACTTGGTATAAGTT-GAAAACGTAC-TATA--TGTGT-GGTTCGCT-GACAACTTGTCCA--T-C-TTT----T-ATAT-ATTATGCG-CGCACTTGG-----TTT----TACGCTCTGTGC--GAGT--ATATA------TTTTTTTTATGAC-CTCAGCTCAAGCAAGAATACCC-GCTGAACTTAAGCATATCAATAAGCGGAGGAAAAGAAACTAACAAGGATTCCCCTAGTAACGGCGAGTGAAGAGGGAAAAGCTCAAATTTTAAATCTACC-TGG---TTC--CCAGGTCGAATTGTAATTTGAAGAAGCGATATC-GG-TT-TTGAGGTCTGGTTTAAGTTCTTTGGAACAAGACATCATGGG-AGGGTGAGAATCCCGTGCATGATCAGA-CC---AAGAT--AC-CCAAT---ATTCGTTTTCTAAGAGTCGAGTTGTTTGGGAATGCAGCTCAAAATGGG-T-GGTAGAC-TTCACCTAAGGCTAAATATCAGCGAGAGACCGATAGCGAACAAGTACTGTG-AA-GGAAAGATGAAAAGAACTTTGAAAAGAGAGTTAAATAGTACGTGAAATTGTTGAAAGGGAAACGATTGAAGTCAGTCATGCTAG-TGAAAAATCAGTTTGACGGG-TTTTG-AGTTCTTGAGT--AGAGGCAGGGT---CAA---ACCGTTTCTCTTTTGGACTTGAGATTTGTTAGATGTACTTTTTCT-TTGGCAGGTCAACGTCGGTTTC-GGGGGTTGTAAA---------ATACTTGGGGA-AAAGTAGCTCTGCTTCGGGA---GAGTG-TTATAGACCCTGGGAGATGCAGCCTGCGGGATCGAGGATTGCAGCAAATGC-------------------CTTCG--GGC-TTGTCGCCTG-TTCCTGG-GTGTTACCTCTCTTGTGACAACATT-CTTGCCACCGGAGGGTACTAATGCTCACT-GTTTAGAGCGATCGAAAA-TTTTGCTAAGGATGTTGACGTAATGGCTTTAAACGACCCGTCTTGAAACACGGACCAAGGAGTCTAACATATATGCGAG------------------------------------------------------------------------------gtggagaagatgatttgactcacaaattatccgacatcttgaaggcaaatcaaaacgtaaaacgttatgaagctgatggtcatccaccacacgttgtaaacgaatttgaagcattattacaggttcttaatgat---------------------------------------------------------------------------------------------------------tataa-tttca--------a-ttagtttaatatcaa--ttgaag--tttatacttaaa-tttattatttat-----atgcaaacagtttcattgtgcaacttatatggacaatgaaatggctggtcaacctcaagctcttcagaaatctggtagacctttaaagtcaatacgtgcgcgtctcaagggtaaagaagggcgtttacgcggtaatctgatgggaaagcgtgtagatttctctgctcgtacagtaattacgggtgatccaaatatctcagttgatgaagtcggagttccgaaaagcatagctcaaaatttaacttttccagaattggtgactccctttaatattgactatctccaaaaattagtagaaaatggcccttctacacatccgggggctaaatacgtaattagagatactggtgaaagaatagatctaaaacatatatcaggcatgactggtggcttaagattacactacggttggaaagttgaacgtcatctcaatgatggtgacatcgttatattcaatcgtcagccatctttgcacaaaatgtcaatgat----------------------------------------------------------------------------------------------------------------------------------------------------------------------------------------------------------------------------------------------------------------------------------------

>Diversispora_varaderana_KT444709

AGGAATCCCTAGTAAGCATGAGTCATCAGCTCATG-TTGATTACGTCCCTGCCCTTTGTACACACCGCCCGTCGCTACTACCGATTGAATGGCTTAGTGAGACCTTTGGATTGGGGTTTAGGGATCGGAAAC---GATCCTTATTCTCCGAGAAGTCGGTCAAACTTGGTCATTTAGAGGAAGTAAAAGTCGTAACAAGGTTTCCGTAGGTGAACCTGCGGAAGGATCATT-AAAAAAT-CTTTA-TCCGGGAATTCG-----TTTCGT----T--------TTCCCG-G------ATTATTTGTATTCAAA-TCCCACTCTTT-------AT-AAAT-ATA---------TTAATTATATAAAAC--AAAA-A-TAAAAGAGAAAACTTTCAACAACGGATCTCTTGGCTCTCGCATCGATGAAGAACGCAGCGAAATGCGATACGTAGTGTGAATTGCAGAATTCCGTGAATCATCGAA-TCTTTGAACACAAATTGTACTTTCCAGTAATCTGGGAAGTATGCTTGGTTGAGGATCATTAAAATAACA-TTCGTGAG--------------TTTTTTCG-------------CGGATTTGAG----TTTT-CC--AGTA--TTT-TTAT---TAT-----AA--------AA-AAAATGTTGGTAACTTT-AAAATT-ATTT------ATTACTTGGTACAAGTT-GAAAACGTAC-TATA--TGTGT-GGTTCGCT-GACAACTTGTCCA--T-C--TT----T-ATAT-ATTATGCC-CGCACTTGG----TTTT----TATGCTCTGTGCG-GAGT--ATATA-----TTTTTTTTTATGAC-CTCAGCTCAAGCAAGAACACCCGGCTGAACTTAAGCATATCAATAAGCGGAGGAAAAGAAACTAACAAGGATTCCCCTAGTAACGGCGAGTGAAGAGGGAAAAGCTCAAATTTTAAATCTACC-TGG---TTC--CCAGGTCGAATTGTAATTTGAAGAAGCGATATCGGG-TT-TTGAGGTCTGGTTCAAGTTCTTTGGAACAAGACATCAT-GG-AGGGTGAGAATCCCGTGCATGATCAGA-CC---AAGAT--AC-CCAAT---ATTCGTTTTCTAAGAGTCGAGTTGTTTGGGAATGCAGCTCAAAATGGG-T-GGTAGAC-TTCACCTAAGGCTAAATATCAGCGAGAGACCGATAGCGAACAAGTACTGTG-AA-GGAAAGATGAAAAGAACTTTGAAAAGAGAGTTAAATAGTACGTGAAATTGTTGAAAGGGAAACGATTGAAGTCAGTCATGCTAG-TGAAAAATCAGTTTGACGGG-TTTTG-AGTTCTTGAGT--AGAGGCAGGGT---CAA---ACCGTTTCTCTTTTGGACTTGAGATTTGTTAGATGTACTTTTTCT-TTGGCAGGTCAACGTCGGTTTC-GGGGGTTGTAAA---------ATACTCGGGGA-AAAGTAGCTCTGCTTCGGTA---GAGTG-TTATAGACCCTGGGAGATGCAGCCTGCGGGATCGAGGATTGCAGCAAATGC-------------------CTTCG--GGC-TTGTCGCCTG-TTCCTGG-GTGTTACCTCTCTTGTGACAACATT-CTTGCCACCGGAGGGTACTAATGCTCACT-GTTTAGAGCGATCGAAAA-TTTTGCTAAGGATGTTGACGTAATGGCTTTAAACGACCCGTCTTGAAACACGGACCAAGGAGTCTAACATGTGTGCGAG----------------------------------------------------------------------------------------------------------------------------------------------------------------------------------------------------------------------------------------------------------------------------------------------------------------------------------------------------------------------------------------------------------------------------------------------------------------------------------------------------------------------------------------------------------------------------------------------------------------------------------------------------------------------------------------------------------------------------------------------------------------------------------------------------------------------------------------------------------------------------------------------------------------------------------------------------------------------------------------------------------------------------------------------------------------------------------------------------------------------------------------------------------------------------------------------------------

>Diversispora_varaderana_KT444710

AGGAATCCCTAGTAAGCATGAGTCATCAGCTCATG-TTGATTACGTCCCTGCCCTTTGTACACACCGCCCGTCGCTACTACCGATTGAATGGCTTAGTGAGACCTTTGGATTGGGGTTTAGGGATCGGAAAC---GATCCTTATTCTCCGAGAAGTCGGTCAAACTTGGTCATTTAGAGGAAGTAAAAGTCGTAACAAGGTTTCCGTAGGTGAACCTGCGGAAGGATCATT-AAAAAAT-CTTTA-TCCGGGAATTCG-----TTTCGT----T--------TTCCCG-G------ATTATTTGTATTCAAA-TCCCACTCTTT-------AT-AAAT-ATA---------TTAATTATATAAAAC--AAAA-A-TAAAAGAGAAAACTTTCAACAACGGATCTCTTGGCTCTCGCATCGATGAAGAACGCAGCGAAATGCGATACGTAGTGTGAATTGCAGAATTCCGTGAATCATCGAA-TCTTTGAACGCAAATTGTACTTTCCAGTAATCTGGGAAGTATGCTTGGTTGAGGGTCATTAAAATAACA-TTCGTGAA--------------TTTTTTCG-------------CGGATTTGAG----TTTT-CC--AGTA--TTT-TTAT---TAT------A--------AA-AAAATGTTGGTAACTTT-AAAATT-ATTT------ATTACTTGGTACAAGTT-GAAAACGTAC-TATA--TGTGT-GGTTCGCT-GACAACTTGTCCA--T-C--TT----C-ATAT-ATTATGCG-CGCACTTGG----TTTT----TACGCTCTGTGC--GAGT--ATATA------TTTTTTTTATGAC-CTCAGCTCAAGCAAGAATACCC-GCTGAACTTAAGCATATCAATAAGCGGAGGAAAAGAAACTAACAAGGATTCCCCTAGTAACGGCGAGTGAAGAGGGAAAAGCTCAAATTTTAAATCTACC-TGG---TTC--CCAGGTCGAATTGTAATTTGAAGAAGCGATATC-GG-TT-TTGAGGTCTGGTTCAAGTTCTTTGGAACAAGACATCAT-GG-AGGGTGAGAATCCCGTGCATGATCAGA-CC---AAGAT--AC-CCAAT---ATTCGTTTTCTAAGAGTCGAGTTGTTTGGGAATGCAGCTCAAAATGGG-T-GGTAGAC-TTCACCTAAGGCTAAATATCAGCGAGAGACCGATAGCGAACAAGTACTGTG-AA-GGAAAGATGAAAAGAACTTTGAAAAGAGAGTTAAATAGTACGTGAAATTGTTGAAAGGGAAACGATTGAAGTCAGTCATGCTAG-TGAAAAATCAGTTTGACGGG-TTTTG-AGTTCTTGAGT--AGAGGCAGGGT---CAA---ACCGTTTCTCTTTTGGACTTGAGATTTGTTAGATGTACTTTTTCT-TTGGCAGGTCAACGTCGGTTTC-GGGGGTTGTAAA---------ATACTTGGGGA-AAAGTAGCTCTGCTTCGGGA---GAGTG-TTATAGACCCTAGGAGATGCAGCCTGCGGGATCGAGGATTGCAGCAAATGC-------------------CTTCG--GGC-TTGTCGCCTG-TTCCTGG-GTGTTACCTCTCTTGTGACAACATT-CTTGCCACCGGAGGGTACTAATGCTCACT-GTTTAGAGCGATCGAAAA-TTTTGCTAAGGATGTTGACGTAATGGCTTTAAACGACCCGTCTTGAAACACGGACCAAGGAGTCTAACATGTGTGCGAG----------------------------------------------------------------------------------------------------------------------------------------------------------------------------------------------------------------------------------------------------------------------------------------------------------------------------------------------------------------------------------------------------------------------------------------------------------------------------------------------------------------------------------------------------------------------------------------------------------------------------------------------------------------------------------------------------------------------------------------------------------------------------------------------------------------------------------------------------------------------------------------------------------------------------------------------------------------------------------------------------------------------------------------------------------------------------------------------------------------------------------------------------------------------------------------------------------

>Diversispora_varaderana_KT444711_MG459203

AGGAATCCCTAGTAAGCATGAGTCATCAGCTCATG-CTGATTACGTCCCTGCCCTTTGTACACACCGCCCGTCGCTACTACCGATTGAATGGCTTAGTGAGACCTTTGGATTGGGGTTTAGGGATCGGAAAC---GATTCTTATTCTCCGAGAAGTCGGTCAAACTTGGTCATTTAGAGGAAGTAAAAGTCGTAACAAGGTTTCCATAGGTGAACCTGCGGAAGGATCATT--AAAAAT-TTTTA-TCCGGGAATTCG-----ATTCGTTTCGT--------TTCCCG-G------ATTATTTGTATTCAAA-TCCCACTCTTT-------AT-AAAT-ATA---------TTAATTATATAAAAC--AAAA-A-TAAAAGAGAAAACTTTCAACAACGGATCTCTTGGCTCTCGCATCGATGAAGAACGCAGCGAAATGCGATACGTAGTGTGAATTGCAGAATTCCGTGAATCATCGAA-TCTTTGAACGCAAATTGTACTTTCCAGTAATCTGGGAAGTATGCTTGGTTGAGGGTCATTAAAATAACA-TTCGTGAA--------------TTTTTTCG-------------CGGATTTGAG----TTTT-CC--AGTA--TTT-TTAT---TAT---------------AA-AAAATGTTGGTAACTTT-AAAATT-ATCT------ATTACTTGGTATAAGTT-GAAAACGTAC-TATA--TGTGT-GGTTCGCT-GACAACTTGTCCA--T-C--TT----A-ATAT-ATTATGCG-CGCACTTGG-----TTT----TACGCTCTGTGC--GAGT--ATATA-----TTTTTTTTTATGAC-CTCAGCTCAAGCAAGAATACCC-GCTGAACTTAAGCATATCAATAAGCGGAGGAAAAGAAACTAACAAGGATTCCCCTAGTAACGGCGAGTGAAGAGGGAAAAGCTCAAATTTTAAATCTACC-TGG---TTC--CCAGGTCGAATTGTAATTTGAAGAAGCGATATC-GG-TT-TTGAGGTCTGGTTCAAGTTCTTTGGAACAAGACATCATGGG-AGGGTGAGAATCCCGTGCATGATCAGA-CC---AAGAT--AC-CCAAT---ATTCGTTTTCTAAGAGTCGAGTTGTTTGGGAATGCAGCTCAAAATGGG-TGGGTAGAC-TTCACCTAAGGCTAAATATCAGCGAGAGACCGATAGCGAACAAGTACTGTG-AA-GGAAAGATGAAAAGAACTTTGAAAAGAGAGTTAAATAGTACGTGAAATTGTTGAAAGGGAAACGATTGAAGTCAGTCATGCTAG-TGAAAAATCAGTTTGACGGG-TTTTG-AGTTCTTGAGT--AGAGGCAGGGT---CAA---ACCGTTTCTCTTTTGGACTTGAGATTTGTTAGATGTACTTTTTCT-TTGGCAGGTCAACGTCGGTTTC-GGGGGTTGTAAA---------ATACTCGGGGA-AAAGTAGCTCTGCTTCGGTA---GAGTG-TTATAGACCCTGGGAGATGCAGCCTGCGGGATCGAGGATTGCAGCAAATGC-------------------CTTCG--GGC-TTGTCGCCTG-TTCCTGG-GTGTTACCTCTCTTGTGACAACATT-CTTGCCACCGGAGGGTACTAATGCTCACT-GTTTAGAGCGATCGAAAA-TTTTGCTAAGGATGTTGACGTAATGGCTTTAAACGACCCGTCTTGAAACACGGACCAAGGAGTCTAACATGTGTGCGAG------------------------------------------------------------------------------gtggagaagatgatttgacacacaaattatccgacatcttgaaggcaaatcaaaacgtaaaacgttatgaagctgatggtcatccaccacacgttgtaaacgaatttgaagcattattacaggttcttaatgat---------------------------------------------------------------------------------------------------------tataa-tttca--------a-ttagtttaatatcaa--ttgaag--tttatacttaaa-tttattatttat-----atgcaaacagtttcattgtgcaacttatatggacaatgaaatggctggtcaacctcaagctcttcagaaatctggtagacctttaaagtcaatacgtgcgcgtctcaagggtaaagaagggcgtttacgcggtaatctgatgggaaagcgtgtagatttctctgctcgtacagtaattacgggtgatccaaatatctcagttgatgaagtcggagttccgaaaagcatagctcaaaatttaacttttccagaattggtgactccctttaatattgactatctccaaaaattagtagaaaatggcccttctacacatccgggggctaaatacgtaattagagatactggtgaaagaatagatctaaaacatatatcaggcatgactggtggcttaagattacactacggttggaaagttgaacgtcatctcaatgatggtgacatcgttatattcaatcgtcagccatctttgcacaaaatgtcaatgat----------------------------------------------------------------------------------------------------------------------------------------------------------------------------------------------------------------------------------------------------------------------------------------

>Diversispora_aestuarii_OL684642_OL690405

AGGAATCCCTAGTAAGCATGAGTCATCAGCTCATG-TTGATTACGTCCCTGCCCTTTGTACACACCGCCCGTCGCTACTACCGATTGAATGGCTTAGTGAGACCTTTGGATTGGGGTTTAGGGATCGGCAAC---GATCCTTATTCTCCGAGAAGTCGGTCAAACTTGGTCATTTAGAGGAAGTAAAAGTCGTAACAAGGTTTCCGTAGGTGAACCTGCGGAAGGATCATT-AAAAAAT-CTTTA-TCCAGGAATTCG-----TTTCGT----C--------TTCCTG-G------ATTATTTGTATTCAAA-TCCCACTCTTT-------AT-AAAT-ATA---------TTAATTATATAAAAC--AAAA-A-TATAAAAGAAAACTTTCAACAACGGATCTCTTGGCTCTCGCATCGATGAAGAACGCAGCGAAATGCGATACGTAGTGTGAATTGCAGAATTCCGTGAATCATCGAA-TCTTTGAACGCAAATTGTACTTTCCAGTAATCTGGGAAGTATGCTTGGTTGAGGGTCATTAAAATAACA-TTCGTGAA--------------TTTTTTCG-------------CGGATTTGAG----TTTT-CC--AGTA--TTT-TTAT---TAT------A--------AA-AAAATGTTGATAACTTT-AAAATT-ATTT------ATTACTTGGTACAAGTT-GAAAACGTAC-TATA--TGTGT-GGTTCGCT-GACAACTTGTCCG--T-C--TT----T-ATAT-ATTATGCG-CGCACTTGG----TTTT----TATGCTCTGTGC--GAGT--ATATA-------TTTTTTTATGAC-CTCAGTTCAAGCAAGAATACCC-ACTGAACTTAAGCATATCAATAAGCGGAGGAAAAGAAACTAACAAGGATTCCCTTAGTAACGGCGAGTGAAGAGGGAAAAGTTCAAATTTTAAATCTACC-TGG---TTT--CCAGGTCGAATTGTAATTTGAAGAAGCGATATC-AG-TA-TTGAGGTCTGGTTCAAGTTCTTTGGAACAAGACATCAT-GG-AGGGTGAGAATCCCGTGCATGATCAGA-CC---AAGAT--AC-CTAAT---ATTCGTTCTCTAAGAGTCGAGTTGTTTGGGAATGCAGCTCAAAATGGG-T-GGTAGAC-TTCACCTAAGGCTAAATATCAGCGAGAGACCGATAGCGAACAAGTACTGTG-AA-GGAAAGATGAAAAGAACTTTGAAAAGAGAGTTAAATAGTACGTGAAATTGTTGAAAGGGAAACGATTGAAGTCAGTCATGCTAG-TGAAAAATCAGTTTGACGGG-TTTTG-AGTTCTTGAGT--AGAGGCAGGGT---CAA---ACCGTTTCTCTTTTGAACTTGGGATTTGTTAGATGTACTTTTTCT-TTGGCAGGTCAACGTCGGTTTC-GGGGGTTGTAAA---------TTACTTGGGGG-AAAGTAGCTCTGCTTCGGGA---GAGTG-TTATAGACCCTGGGAGATGCAGCCTGTGGGATCGAGGATTGCAGCAAATGC--------------------CTTT--GGC-TTGTCGCCTG-TTCCTGG-GTGTTACCTCTCTTGTGACAACATT-CTTGCCACCGGAGGGTTCTAATGCTCACT-GTTTAGTGCGATCAAAAA-TTTTGCTAAGGATGTTGACGTAATGGCTTTAAACGACCCGTCTTGAAACACGGACCAAGGAGTCTAACATATGTGCGAGctgattggatgatcataactatacttcccgttcctcctcctcctgtacgtcccagtatacaaatggacggaacaagtcgaggtgaagatgatttgactcacaaattatccgacatcttgaaggcaaatcaaaacgtaaaacgttatgaagctgatggtcatccaccacacgttgtaaacgaatttgaagcattattacaggttcgtaatgat---------------------------------------------------------------------------------------------------------tataa-tttca--------a-ttagtttgatatcaa--ttgaag--tttatacctaaa-tttattatttat-----atgcaaacagtttcattgtgcaacttatatggacaatgaaatggctggtcaacctcaagctcttcaaaaatctggtagacctttaaagtcaatacgtgcgcgtctcaagggtaaagaagggcgtttacgcggtaatctgatgggaaagcgtgtagatttctctgctcgtacagtaattacgggtgatccaaatatctcagttgatgaagtcggagttccgaaaagcatagctcaaaatttgacttttccagaattagtgactccctttaatattgactatcttcaaaaattagtagaaaatggcccttctacacatccaggggctaaatacgtaattagagataccggtgaaagaatagatctaaaacatatatcaggtatgactggtggcttaagattacactacggttggaaagttgaacgtcatctcaatgatggtgacatcgttatattcaatcgtcagccatctttgcacaagatgtcgatgatgggacataaagttcgtgttatgccctattcgaccttccgtcttaatttatcagttacaacaccatataacgccgattttgatggtgacgaaatgaacatgcatgttccccaatcagtggaaactaaagcagaaatttcagaaatctgtatggttcctaaacaaattgtatctcctcaatcaaataaacctgttatgggtattgtacaggatactttatgtgctgttagaaaatttacaaaaagggattgctttttatctaaagatttggtaatgaacatt

>Diversispora_aestuarii_OL684645

AGGAATCCCTAGTAAGCATGAGTTATCAGCTCATG-TTGATTACGTCCCTGCCCTTTGTACACACCGCCTGTCGTTACTACCGATTGAATGGCTTAGTGAGACCTTTGGATTGGGGTTTAGGGATCGGCAAC---GATCCTTATTCTCCGAGAAGTCAGTCAAACTTGGTCATTTAGAGGAAGTAAAAGTCGTAACAAGGTTTCCGTAGGTGAACCTGCGGAAGGATCATT--AAAAAT-CTTTA-TCCGGGAATTCG-----TTTCGT----T--------TTCTCGGA------TTTATTTGTATTCAAA-TTCCACTCTTT-------AT-AAAT-ATA---------TTAATTATATAAAAC--AAAA-A-TAAAAAAGAAAACTTTCAACAACGGATCTCTTGGCTCTCGCATCGATGAAGAACGCAGCGAAATGCGATACGTAGTGTGAATTGCAGAATTCCGTGAATCATCGAA-TCTTTGAACGCAAATTGTACTTTCCAGTAATCTGGGAAGTATGCTTGGTTGAGGGTCATTAAAATAACA-TTCGTGAA--------------TTTTTTCG-------------CGGATTTGAG----TTTT-CC--AGTA--TTT-TTAT---TAT------A--------AA-AAAATGTTGGTAACTTT-AAAATT-ATTT------ATTACTTGGTACAAGTT-GAAAACGTAC-TATA--TGTGT-GGTTCGCT-GACAACTTGTCCA--T-C--AT----C-ATAT-ATTATGCG-CGCACTTGG----TTTT----TACGCTCTGTGC--GAGT--ATATA-------TTTTTTTATGAC-CTCAGCTCAAGCAAGAATACCC-GCTGAACTTAAGCATATCAATAAGCGGAGGAAAAGAAACTAACAAGGATTCCCCTAGTAACGGCGAGTGAAGAGGGAAAAGCTCAAATTTTAAATCTACC-TGG---GTTGACCAGGTCGAATTGTAATTTGAAGAAGCGATATC-AG-TG-CCGA-GTCTGGTTCAAGTTCTTTGGAACAAGACATCAT-GG-AGGGTGAGAATCCCGTGCATGATCAGACCC---AAGGT--AC-CTAAT---ATTCGTTTTCTAAGAGTCGAGTTGTTTGGGAATGCAGCTCAAAATGGG-T-GGTAGAC-TTCACCTAAGGCTAAATATCAGCGAGAGACCGATAGCGAACAAGTACTGTG-AA-GGAAAGATGAAAAGAACTTTGAAAAGAGAGTTAAATAGTACGTGAAATTGTTGAAAGGGAAACGATTGAAGTCAGTCATGCTAG-TGAAAAATCAGTTTGACGGG-TTTTG-AGTTCTTGAGT--TGAGGCAGGGT---CAA---ACCGTTTCTCTTTTGAACTTGGGATTTGTTAGATGTACTTTTTCT-TTGGCAGGTCAACGTCGGTTTC-GGGGGTTGTAAA---------TTACTTGGGGG-AAAGTAGCTCTGCTTCGGGA---GAGTG-TTATAGACCCTGGGAGATGCAGCCTGTGGGATCGAGGATTGCAGCAAATGC-------------------CTTCT--GGC-TTGTCGCCTG-TTCCTGG-GTGTTACCTCTCTTGTGACAACATT-CTTGCCACCGGAGGGCACTAATGCCTACT-GTTTAGAGCGATCGAAAA-TTTTGCTAAGGATGTTGACGTAATGGCTTTAAACGACCCGTCTTGAAACACGGACCAAGGAGTCTAACATATGTGCGAG----------------------------------------------------------------------------------------------------------------------------------------------------------------------------------------------------------------------------------------------------------------------------------------------------------------------------------------------------------------------------------------------------------------------------------------------------------------------------------------------------------------------------------------------------------------------------------------------------------------------------------------------------------------------------------------------------------------------------------------------------------------------------------------------------------------------------------------------------------------------------------------------------------------------------------------------------------------------------------------------------------------------------------------------------------------------------------------------------------------------------------------------------------------------------------------------------------

>Diversispora_aestuarii_OL684648

AGGAATCCCTAGTAAGCATGAGTCATCAGCTCATG-TTGATTACGTCCCTGCCCTTTGTACACACCGCCCGTCGCTACTACCGATTGAATGGCTTAGTGAGACCTTTGGATTGGGGTTTAGGGATCGGCAAC---GATCCTTATTCTCCGAGAAGTCGGTCAAACTTGGTCATTTAGAGGAAGTAAAAGTCGTAACAAGGTTTCCGTAGGTGAACCTGCGGAAGGATCATT-AAAAAAT-CTTTA-TCCGGGAATTCG-----TTTCGT----T--------TTCCCG-G------ATTATTTGTATTCAAA-TCTCACTCTTT-------AT-AAAT-ATA---------TTAATTATATAAAAC--AAAA---ATAAAAAGAAAACTTTCAACAACGGATCTCTTGGCTCTCGCATCGATGAAGAACGCAGCGAAATGCGATACGTAGTGTGAATTGCAGAATTCCGTGAATCATCGAA-TCTTTGAACGCAAATTGTACTTTCCAGTAATCTGGGAAGTATGCTTGGTTGAGGGTCATTAAAATAACA-TTCGTGAA--------------TTTTTTCG-------------CGGATTTGAG----TTTT-CC--AGTA--TTT-TTAT---TAT------A--------AA-AAAATGTTGGTAACTTT-AAAATT-ATTT------ATTACTTGGTACAAGTT-GAAAACGTAC-TATA--TGTGT-GGTTCGCT-GACAACTTGTCCA--T-C--AT----C-ATAT-ATTATGCG-CGCACTTGG----TTTT----TACGCTCTGTGC--GAGT--ATATA------TTTTTTTTATGAC-CTCAGCTCAAGCAAGAATACCC-GCTGAACTTAAGCATATCAATAAGCGGAGGAAAAGAAACTAACAAGGATTCCCCTAGTAACGGCGAGTGAAGAGGGAAAAGCTCAAATTTTAAATCTACC-TGG---GTTGACCAGGTCGAATTGTAATTTGAAGAAGCGATATC-GG-TA-TTGAGGTCTGGTTCAAGTTCTTTGGAACAAGACATCAT-GG-AGGGTGAGAATCCCGTGCATGATCAGA-CCC-AAGGTA--CC-TTAAT---ATTCGTTTTCTAAGAGTCGAGTTGTTTGGGAATGCAGCTC-AAATGGG-T-GGTAGAC-TTCACCTAAGGCTAAATATCAGCGAGAGACTGATAGCGAACAAGTATTGTG-AA-GGAAAGATGAAAAGAACTTTGAAAAGAGAGTTAAATAGTATGTGAAATTGTTGAAAGGGAAATGATTGAAGTCAGTCATGCTAG-TGAAAAATCAGTTTGACGGG-TTTTG-AGTTCTTGAGT--AGAGGCAGGGT---CAA---ACCGTTTCTCTTTTGAACTTGGGATTTGTTAGACGTACTTTTTCT-TTGGCAGGTCAACGTCAGTTTC--GGGGTTGTAAA---------ATATTTGGGAG-AAAGTAGCTCTGCTTCGGGA---GAGTG-TTATAGA-CCTGGGAGATGCAGCCTGCGGGATCGAGGATTGCAGCAAATGC--------------------CTTT--GGC-TTGTCGCCTG-TTCCTGG-GTGTTACCTCTCTTGTGACAACATT-CTTGCCTCCGGAGGGTTCTAATGCTCACT-GTTTAGTGCGATCAAAAA-TTTTGCTAAGGATGTTGACGTAATGGCTTTAAACGACCCATCTTGAAATACGGACCAAGGAGTCTAACATATGTGCGAG----------------------------------------------------------------------------------------------------------------------------------------------------------------------------------------------------------------------------------------------------------------------------------------------------------------------------------------------------------------------------------------------------------------------------------------------------------------------------------------------------------------------------------------------------------------------------------------------------------------------------------------------------------------------------------------------------------------------------------------------------------------------------------------------------------------------------------------------------------------------------------------------------------------------------------------------------------------------------------------------------------------------------------------------------------------------------------------------------------------------------------------------------------------------------------------------------------

>Diversispora_aestuarii_OL684644_OL690406

AGGAATCCCTAGTAAGCATGAGTTATCAGCTCATG-TTGATTACGTCCCTGCCCTTTGTACACACCGCCTGTCGTTACTACCGATTGAATGGCTTAGTGAGACCTTTGGATTGGGGTTTAGGGATCGGCAAC---GATCCTTATTCTCCGAGAAGTCAGTCAAACTTGGTCATTTAGAGGAAGTAAAAGTCGTAACAAGGTTTCCGTAGGTGAACCTGCGGAAGGATCATT--AAAAAT-CTTTA-TCCGGGAATTCG-----TTTCGT----T--------TTCTCGGA------TTTATTTGTATTCAAA-TTCCACTCTTT-------AT-AAAT-ATA---------TTAATTATATAAAAC--AAAA-T-AAAAAAAGAAAACTTTTAACAACGGATCTCTTGGCTCTCGTGTCGATGAAGAACGCAGCGAAATGCGATACGTAGTGTGAATTGCAAAATTCCGTGAATCATTGAA-TCTTTGAACGCAAATTGTACTTTCCAGTAATCTGGAAAGTATGCTTAGTTGAGGGTCATTAAAATAACA-TTTGTGAA---------------TTTTTCG-------------CGAATTTGAG----TTTT-CC--AGTA--TTT-TTAT---TAT------A--------AA-AAAATGTTGATAACTTT-AAAATT-ATTT------ATTACTTGGTACAAGTT-GAAAACGTAC-TATA--TGTGT-GGTTCGCT-GACAACTTGTCCG--T-C--TT----T-ATAT-ATTATGCG-CGCACTTGG----TTTT----TATGCTCTGTGC--GAGT--ATATA-------TTTTTTTATGAC-CTCAGTTCAAGCAAGAATACCC-ACTGAACTTAAGCATATCAATAAGCGGAGGAAAAGAAACTAACAAGGATTCCCTTAGTAACGGCGAGTGAAGAGGGAAAAGTTCAAATTTTAAATCTACC-TGG---TTT--CCAGGTCGAATTGTAATTTGAAGAAGCGATATC-AG-TG-CCGA-GTCTGGTTCAAGTTCTTTGGAACAAGACATCAT-GG-AGGGTGAGAATCCCGTGCATGATCAGA-CCC-AAGGTA--CC-TTAAT---ATTCGTTTTCTAAGAGTCGAGTTGTTTGGGAATGCAGCTCAAAATGGG-T-GGTAGAC-TTCACCTAAGGCTAAATATCAGCGAGAGACCGATAGCGAACAAGTATTGTG-AA-GGAAAGATGAAAAGAACTTTGAAAAGAGAGTTAAATAGTATGTGAAATTGTTGAAAGGGAAATGATTGAAGTCAGTCATGCTAG-TGAAAAATCAGTTTGACGGG-TTTTG-AGTTCTTGAGT--TGAGGCAGGGT---TAA---ACCGTTTCTCTTTTGAACTTGGGATTTGTTAGACGTACTTTTTCT-TTGGCAGGTCAACGTCGGTTTC-GGGGGTTGTAAA---------ATACTTGGGGG-AAAGTAGCTCTGCTTCGGGA---GAGTG-TTATAGACCCTGGGAGATGCAGCCTGTGGGATCGAGGATTGCAGCAAATGC-------------------CTTCT--GGC-TTGTCGCCTG-TTCCTGG-GTGTTACCTCTCTTGTGACAACATT-CTTGCCTCCGGAGGGTTCTAATGCTCACT-GTTTAGTGCGATCAAAAA-TTTTGCTAAGGATGTTGACGTAATGGCTTTAAACGACCCGTCTTGAAACACGGACCAAGGAGTCTAACATATGTGCGAGctgattggatgatcataactatacttcccgttcctcctcctcctgtacgtcccagtatacaaatggacggaacaagtcgaggtgaagatgatttgactcacaaattatccgacatcttgaaggcaaatcaaaacgtaaaacgttatgaagctgatggtcatccaccacacgttgtaaacgaatttgaagcattattacaggttcgtaatgat---------------------------------------------------------------------------------------------------------tataa-tttca--------a-ttagtttgatatcaa--ttgaag--tttatacctaaa-tttattatttat-----atgcaagcagtttcattgtgcaacttatatggacaatgaaatggctggtcaacctcaagctcttcaaaaatctggtagacctttaaagtcaatacgtgcgcgtctcaagggtaaagaagggcgtttacgcggtaatctgatgggaaagcgtgtagatttctctgctcgtacagtaattacgggtgatccaaatatctcagttgatgaagtcggagttccgaaaagcatagctcaaaatttgacttttccagaattagtgactccctttaatattgactatcttcaaaaattagtagaaaatggcccttctacacatccaggggctaaatacgtaattagagataccggtgaaagaatagatctaaaacatatatcaggtatgactggtggcttaagattacactacggttggaaagttgaacgtcatctcaatgatggtgacatcgttatattcaatcgtcagccatctttgcacaagatgtcgatgatgggacataaagttcgtgttatgccctattcgaccttccgtcttaatttatcagttacaacaccttataacgccgattttgatggtgacgaaatgaacatgcatgttccccaatcagtggaaactaaagcagaaatttcagaaatctgtatggttcctaaacaaattgtatctcctcaatcaaataaacctgttatgggtgttgtacaggatactttatgtgctgttagaaaatttacaaaaagggattgctttttatctaaagatttggtaatgaacatt

>Diversispora_clara_FR873629_MG459184

AGGAATCCCTAGTAAGCATGAGTCATCAGCTCATG-TTGATTACGTCCCTGCCCTTTGTACACACCGCCCGTCGCTACTACCGATTGAATGGCTTAGTGAGACCTTTGGATTGGGGTTTAGAGATCGGAAAC---GACCTTTATTCTCCGAGAAGTCGGTCAAACTTGGTCATTTAGAGGAAGTAAAAGTCGTAACAAGGTTTCCGTAGGTGAACCTGCGGAAGGATCATT--AAAAAT-ATTTA-TCCGAGAATTCT-----TTACGT----T--------TTCCCG-G------ATTATTTGTATTCAAA-TCCCACTCTTT-------AT-AAAT-ATA---------CCAATTATATAAAAC-AAAAA-T--AAATAAGAAAACTTTCAACAACGGATCTCTTGGCTCTCGCATCGATGAAGAACGCAGCGAAATGCGATACGTAATGTGAATTGCAG-ATTCCGTGAATCATCGAA-TCTTTGAACGCAAATTGTACTTTCTAGTAATCTAGGAAGTATGCTTGGTTGAGGGTCATTAAAATAACA-TTCGTGAA--------------TTCTTTCG-------------CGGATTTGAG----TTTT-CC--GGCA--TTT---AT-----------AA--------TA-TAAACGTTGGTAACTTT-AAAATT-ATT--------TTACTAGGTACAAGTT-GAAGACGTTC-TATA--TGTTT-GGTTCGCT-GACAACTTGTCCA--T-C--TC----T-ATAT-ATTATGCG-CGCACTTGG---TTTTT----AAAGCTCTGTAC--GAGT--ATATA------TTTTTTTTATGAC-CTCAGCTCAAGCAAGAATACCC-GCTGAACTTAAGCATATCAATAAGCGGAGGAAAAGAAACTAACAAGGATTCCCCTAGTAACGGCGAGTGAAGAGGGAAAAGCTCAAATTTTAAATCTACC-TGG---TTC--CCAGGTCGAATTGTAATTTGAAGAAGCGATATC-GG-TT-TTGAGGTCTGGTTCAAGTTCTTTGGAACAAGACATCAT-GG-AGGGTGAGAATCCCGTGCATGATCAGA-CC---AAGAT--AC-CTAAT---ATTCGTTTTCTAAGAGTCGAGTTGTTTGGGAATGCAGCTCAAAATGGG-T-GGTAGAC-TTCACCTAAGGCTAAATATCAGCGAGAGACCGATAGCGAACAAGTACTGTG-AA-GGAAAGATGAAAAGAACTTTGAAAAGAGAGTTAAATAGTACGTGAAATTGTTGAAAGGGAAACGATTGAAGTCAGTCATGCTAG-TGAAAATTCAGTTTGGCGGA-TTTTG-AGTTCAGGAGTAAGGAGACAGGGT---CAA---ACCGTTTCTCTTCTGGACTTCGAATTTGTCAGATGCACTTTTTCT-TTGGCAGGTCAGCGTCGGTTTC-GGAGGTTGTAAA---------ATACTTGGGGT-AAAGTAGCTCTGCTTCGGGA---GAGTG-TTATAGACCTTGGGGGATGCAGCCTGCGGGATCGAGGATTGCAGCAAATGC--------------------CTTT--GGC-TTGTCGCCTGGTCTCTGG-ACGTTACCTCGCTTGTGACAACATT-CTTGCCACCGGTGGGTACTAATGCTCATTAGGTTAGAGCGATCAAAAA--TTTGCTAAGGATGTTGACGTAATGGCTTTAAACGACCCGTCTTGAAACACGGACCAAGGAGTCTAACATATGTGCGAG------------------------------------------------------------------------------gtggagaagatgatttgacacacaaattgtccgacatcttgaaggcaaatcaaaacgtaaaacgttatgaagctgatggtcatcccccacacgttgtaaacgaatttgaagcattgttacaggttcttaatgat---------------------------------------------------------------------------------------------------------tataa-tttca--------a-ttagtttaatatcaa--ttgaaatttttttacttaaa-tttattatttac-----atacaaatagtttcattgtgcaacttatatggacaatgaaatggctggtcaacctcaagctcttcagaaatctggtagacctttaaagtcaatacgtgcgcgtctcaagggtaaagaaggacgtttacgcggtaacctgatgggaaagcgtgtagatttctctgctcgtacagtaattacgggtgatccaaatatttcagttgatgaagtcggagttccgaaaagcatagctcaaaatttgacttttccagaattggtgactccctttaatattgattatcttcaaaaattagtagaaaatggtccttctacacatccaggggctaaatacgtcattagcgatactggtgaaaggattgatctaaaacatatatcaggcatgactggtggcttaagattacattacggttggaaagttgaacgtcatctcaatgatggtgacatcgttatattcaatcgtcagccatctttgcataaaatgtcaatgatggg-------------------------------------------------------------------------------------------------------------------------------------------------------------------------------------------------------------------------------------------------------------------------------------

>Diversispora_clara_FR873632

AGGAATCCCTAGTAAGCATGAGTCATCAGCTCATG-TTGATTACGTCCCTGCCCTTTGTACACACCGCCCGTCGCTACTACCGATTGAATGGCTTAGTGAGACCTTTGGATTGGGGTTTAGAGATCGGAAAC---GACCTTTATTCTCCGAGAAGTCGGTCAAACTTGGTCATTTAGAGGAAGTAAAAGTCGTAACAAGGTTTCCGTAGGTGAACCTGCGGAAGGATCATT--AAAAAT-ATTTA-TCCGAGAATTCT-----TTACGT----T--------TTCCCG-G------ATTATTTGTATTCAAA-TTCCACTCTTT-------AT-AAAT-ATA---------TCAATTATATAAAAC-AAAAA-T-AAAAAAAGAAAACTTTCAACAACGGATCTCTTGGCTCTCGCATCGATGAAGAACGCAGCGAAATGCGATACGTAATGTGAATTGCAG-ATTCCGTGAATCATCGAA-TCTTTGAACGCAAATTGTACTTCCTAGTAATCTAGAAAGTATGCTTGGTCGAGGGTCATTAAAATAACA-TTCGTGAA--------------TTCTTTCG-------------CGGATTTGAG----TTTT-CC--GGCA--TTT---AT-----------AA--------TA-TAAACGTTGGTAACTTT-AAAATT-ATT--------TTACTTGATACAAGTT-GAAAACGTTC-TATA--TGTTT-GGTTCGCT-GACAACTTGTCCA--T-C--TC----T-ATAT-ATTATGCG-CGCACTTGG---TTTTT----AAAGCTCTGTAC--GAGT--ATATA------TTTTTTTTATGAC-CTCAGCTCAAGCAAGAATACCC-GCTGAACTTAAGCATATCAATAAGCGGAGGAAAAGAAACTAACAAGGATTCCCCTAGTAACGGCGAGTGAAGAGGGAAAAGCTCAAATTTTAAATCTACC-TGG---TTC--CCAGGTCGAATTGTAATTTGAAGAAGCGATATC-GG-TT-TTGAGGTCTGGTTCAAGTTCTTTGGAACAAGACATCAT-GG-AGGGTGAGAATCCCGTGCATGATCAGA-CC---AAGAT--AC-CTAAT---ATTCGTTTTCTAAGAGTCGAGTTGTTTGGGAATGCAGCTCAAAATGGG-T-GGTAGAC-TTCACCTAAGGCTAAATATCAGCGAGAGACCGATAGCGAACAAGTACTGTG-AA-GGAAAGATGAAAAGAACTTTGAAAAGAGAGTTAAATAGTACGTGAAATTGTTGAAAGGGAAACGATTGAAGTCAGTCATGCTAG-TGAAAATTCAGTTTGGCGGA-TTTTG-AGTTCAGGAGTAAGGAGGCAGGGT---CAA---ACCGTTTCTCTTCTGGACTTCGAATTTGTCGGATGCACTTTTTCT-TTGGCAGGTCAGCGTCGGTTTC-GGAGGCTGTAAA---------ATACTTGGGGG-AAAGTAGCTCTGCTTCGGTA---GAGTG-TTATAGACCTTGGGGGATGCAGCCTGTGGGATCGAGGATTGCAGCAAATGC-------------------TTTTT--GGC-TTGTCGCCTGGTCTCTGG-ATGTTACCTCGCTTGTGACAACATT-CTTGTCACCGGTGAGTACTAATGCCCATTAGGTTAGAGCGATCAAAAA--TTTGCTAAGGATGCTGACGTAATGGCTTTAAACGACCCGTCTTGAAACACGGACCAAGGAGTCTAACATATGTGCGAG----------------------------------------------------------------------------------------------------------------------------------------------------------------------------------------------------------------------------------------------------------------------------------------------------------------------------------------------------------------------------------------------------------------------------------------------------------------------------------------------------------------------------------------------------------------------------------------------------------------------------------------------------------------------------------------------------------------------------------------------------------------------------------------------------------------------------------------------------------------------------------------------------------------------------------------------------------------------------------------------------------------------------------------------------------------------------------------------------------------------------------------------------------------------------------------------------------

>Diversispora_clara_FR873630

AGGAATCCCTAGTAAGCATGAGTCATCAGCTCATG-TTGATTACGTCCCTGCCCTTTGTACACACCGCCCGTCGCTACTACCGATTGAATGGCTTAGTGAGACCTTTGGATTGGGGTTTAGAGATCGGAAAC---GACCTTTATTCTCCGAGAAGTCGGTCAAACTTGGTCATTTAGAGGAAGTAAAAGTCGTAACAAGGTTTCCGTAGGTGAACCTGCGGAAGGATCATT--AAAAAT-ATTTA-TCCGAGAATTCT-----TTACGT----T--------TTCCCG-G------ATTATTTGTATTCAAA-TCCCACTCTTT-------AT-AAAT-ATA---------CCAATTATATAAAAC-AAAAA-T--AAATAAGAAAACTTTCAACAACGGATCTCTTGGCTCTCGCATCGATGAAGAACGCAGCGAAATGCGATACGTAATGTGAATTGCAG-ATTCCGTGAATCATCGAA-TCTTTGAACGCAAATTGTACTTTCTAGTAATCTAGGAAGTATGCTTGGTTGAGGGTCATTAAAATAACA-TTCGTGAA--------------TTCTTTCG-------------CGGATTTGAG----TTTT-CC--GGCA--TTT---AT-----------AA--------TA-TAAACGTTGGTAACTTT-AAAATT-ATT--------TTACTTGGTACAAGTT-GAAAACGTTC-TATA--TGTTT-GGTTCGCT-GACAACTTGTCCA--T-C--TC----T-ATAT-ATTATGCG-CGCACTTGG---TTTTT----AAAGCTCTGTAC--GAGT--ATATA------TTTTTTTTATGAC-CTCAGCTCAAGCAAGAATACCC-GCTGAACTTAAGCATATCAATAAGCGGAGG-AAAGAAACTAACAAGGA-TCCCCTAGTAACGGCGAGTGAAGAGGGAAAAGCTCAAATTTTAAATCTACC-TGG---TTC--CCAGGTCGAATTGTAATTTGAAGAAGCGATATC-GG-TT-TTGAGGTCTGGTTCAAGTTCTTTGGAACAAGACATCAT-GG-AGGGTGAGAATCCCGTGCATGATCAGA-CC---AAGAT--AC-CTAAT---ATTCGTTTTCTAAGAGTCGAGTTGTTTGGGAATGCAGCTCAAAATGGG-T-GGTAGAC-TTCACCTAAGGCTAAATATCAGCGAGAGACCGATAGCGAACAAGTACTGTG-AA-GGAAAGATGAAAAGAACTTTGAAAAGAGAGTTAAATAGTACGTGAAATTGTTGAAAGGGAAACGATTGAAGTCAGTCATGCTAG-TGAAAATTCAGTTTGGCGGA-TTTTG-AGTTCAGGAGTAAGGAGGCAGGGT---CAA---ACCGTTTCTCTTCTGGACTTCGAATTTGTCGGATGCACTTTTTCT-TTGGCAGGTCAGCGTCGGTTTC-GGAGGTTGTAAA---------ATACTTGGGGG-AAAGTAGCTCTGCTTCGGTA---GAGTG-TTATAGACCTTGGGGGATGCGGCCTGCGGGATCGAGGATTGCAGCAAATGC-------------------TTTTT--GGC-TTGTCGCCTGGTCTCTGG-ATGTTACCTCGCTTGTGACAACATT-CTTGTCACCGGTGGGTACTAATGCCCATTAGGTTAGAGCGATCAAAAA--TTTGCTAAGGATGCTGACGTAATGGCTTTAAACGACCCGTCTTGAAACACGGACCAAGGGGTCTAACATATGTGCGAG----------------------------------------------------------------------------------------------------------------------------------------------------------------------------------------------------------------------------------------------------------------------------------------------------------------------------------------------------------------------------------------------------------------------------------------------------------------------------------------------------------------------------------------------------------------------------------------------------------------------------------------------------------------------------------------------------------------------------------------------------------------------------------------------------------------------------------------------------------------------------------------------------------------------------------------------------------------------------------------------------------------------------------------------------------------------------------------------------------------------------------------------------------------------------------------------------------

>Diversispora_clara_FR873631_MG459185

AGGAATCCCTAGTAAGCATGAGTCATCAGCTCATG-TTGATTACGTCCCTGCCCTTTGTACACACCGCCCGTCGCTACTACCGATTGAATGGCTTAGTGAGACCTTTGGATTGGGGTTTAGAGATCGGAAAC---GACCTTTATTCTCCGAGAAGTCGGTCAAACTTGGTCATTTAGAGGAAGTAAAAGTCGTAACAAGGTTTCCGTAGGTGAACCTGCGGAAGGATCATT--AAAAAT-ATTTA-TCCGAGAATTCT-----TTACGT----T--------TTCCCG-G------ATTATTTGTATTCAAA-TCCCACTCTTT-------AT-AAAT-ATA---------CCAATTATATAAAAC-AAAAA-T--AAATAAGAAAACTTTCAACAACGGATCTCTTGGCTCTCGCATCGATGAAGAACGCAGCGAAATGCGATACGTAATGTGAATTGTAG-ATTCCGTGAATCATCGAA-TCTTTGAACGCAAATTGTACTTTCTAGTAATCTAGGAAGTATGCTTGGTTGAGGGTCATTAAAATAACA-TTCGTGAA--------------TTCTTTCG-------------CGGATTTGAG----TTTT-CC--GGCA--TTT---AT-----------AA--------TA-TAAACGTTGGTAACTTT-AAAATT-ATT--------TTACTTGGTACAAGTT-GAAAACGTTC-TATA--TGTTT-GGTTCGCT-GACAACTTGTCCA--T-C--TC----T-ATAT-ATTATGCG-CGCACTTGG---TTTTT----AAAGCTCTGTAC--GAGT--ATATA-----TTTTTTTTTATGAC-CTCAGCTCAAGCAAGAATACCC-GCTGAACTTAAGCATATCAATAAGCGGAGGAAAAGAAACTAACAAGGATTCCCCTAGTAACGGCGAGTGAAGAGGGAAAAGCTCAAATTTTAAATCTACC-TGG---TTC--CCAGGTCGAATTGTAATTTGAAGAAGCGATATC-GG-TT-TTGAGGTCTGGTTCAAGTTCCTTGGAACAAGACATCAT-GG-AGGGTGAGAATCCCGTGCATGATCAGA-CC---AAGAT--AC-CTAAT---ATTCGTTTTCTAAGAGTCGAGTTGTTTGGGAATGCAGCTCAAAATGGG-T-GGTAGAC-TTCACCTAAGGCTAAATATCAGCGAGAGACCGATAGCGAACAAGTACTGTG-AA-GGAAAGATGAAAAGAACTTTGAAAAGAGAGTTAAATAGTACGTGAAATTGTTGGAAGGGAAACGATTGAAGTCAGTCATGCTAG-TGAAAATTCAGTTTGGCGGA-TTTTG-AGTTCAGGAGTAAGGAGGCAGGGT---CAA---ACCGTTTCTCTTCTGGACTTCGAATTTGTCGGATGCACTTTTTCT-TTGGCAGGTCAGCGTCGGTTTC-GGAGGTTGTAAA---------ATACTTGGGGG-AAAGTAGCTCTGCTTCGGTA---GAGTG-TTATAGACCTTGGGGGATGCAGCCTGCGGGATCGAGGATTGCAGCAAATGC-------------------TTTTT--GGC-TTGTCGCCTGGTCTCTGG-ATGTTACCTCGCTTGTGACAACATT-CTTGTCACCGGTGGGTACTAATGCCCATTAGGTTAGAGCGATCAAAAA--TTTGCTGAGGATGCTGACGTAATGGCTTTAAACGACCCGTCTTGAAACACGGACCAAGGAGTCTAACATATGTGCGAG-------------------------------------------------------------------------------tggagaagatgatttgactcacaaattgtccgacatcttgaaggcaaatcaaaacgtaaaacgttatgaagctgatggtcatcccccacacgttgtaaacgaatttgaagcattgttacaggttcttaatgat---------------------------------------------------------------------------------------------------------tataa-tttca--------a-ttagtttaatatcaa--ttgaaatttttttacttaaa-tttattatttac-----atacaaatagtttcattgtgcaacttatatggacaatgaaatggctggtcaacctcaagctcttcagaaatctggtagacctttaaagtcaatacgtgcgcgtctcaagggtaaagaaggacgtttacgcggtaacctgatgggaaagcgtgtagatttctctgctcgtacagtaattacgggtgatccaaatatttcagttgatgaagtcggagttccgaaaagcatagctcaaaatttgacttttccagaattggtgactccctttaatattgattatcttcaaaaattagtagaaaatggtccttctacacatccaggggctaaatacgtaattagagatactggtgaaaggattgatctaaaacatatatcaggcatgactggtggcttaagattacattacggttggaaagttgaacgtcatctcaatgatggtgacatcgttatattcaatcgtcagccatctttgcataaaatgtcaatgatggg-------------------------------------------------------------------------------------------------------------------------------------------------------------------------------------------------------------------------------------------------------------------------------------

>Diversispora_peloponnesiaca_MN306206_OL690409

GGGAATCCCTAGTAAGCATGAGTCATCAGCTCATG-TTGATTACGTCCCTGCCCTTTGTACACACCGCCCGTCGCTACTACCGATTGAATGGCTTAGTGAGACCTTTGGATTGGGGTTTAGAGATCGGAAAC---GATCTTTATTCTCCGAGAAGTCGGTCAAACTTGGTCATTTAGAGGAAGTAAAAGTCGTAACAAGGTTTCCGTAGGTGAACCTGCGGAAGGATCATT--AAAAAT-ATTTA-TCCGAGAATTCT-----TCACGT----T--------TTCTCG-G------ATTATTTGTATTCAAA-TTCCACTCTTT-------AT-AAAT-ATA---------TCAATTATATAAAAC-AAAAA-T-AAAAAAAGAAAACTTTCAACAACGGATCTCTTGGCTCTCGCATCGATGAAGAACGCAGCGAAATGCGATACGTAATGTGAATTGCAG-ATTCCGTGAATCATCGAA-TCTTTGAACGCAAATTGTACTTCCTAGTAATCTAGAAAGTATGCTTGGTTGAGGGTCATTAAAATAACA-TTCGTGAA--------------TTCTTTCG-------------CGGATTTGAG----TTTT-CC--GGCA--TTT---AT-----------AA--------TA-TAAACGTGGGTAACTTT-AAAATC-ATTT-------TTACTTGGTACAAGTT-GAAAACGTGC-TATA--TGTTT-GGTTCGCT-GACAACTTGTCCATCT-C--TC----T-ATAT-ATTATGCG-CGTACTTGG-TTTTTTT----AAAGCTCTGTGC--GAGT--ATATA-----TTTTTTTTTATGAC-CTCAGCTCAAGCAAGAATACCC-GCTGAACTTAAGCATATCAATAAGCGGAGGAAAAGAAACTAACAAGGATTCCCCTAGTAACGGCGAGTGAAGAGGGAAAAGCTCAAATTTTAAATCTACC-TGG---TTC--CCAGGTCGAATTGTAATTTGAAGAAGCGATATC-GG-TT-TTGCGGTCTGGTTCAAGTTCTTTGGAACAAGACATCAT-GG-AGGGTGAGAATCCCGTGCATGATCAGA-CC---AAGAT--AC-CTAAT---ATTCGTTTTCTAAGAGTCGAGTTGTTTGGGAATGCAGCTCAAAATGGG-T-GGTAGAC-TTCACCTAAGGCTAAATATCAGCGAGAGACCGATAGCGAACAAGTACTGTG-AA-GGAAAGATGAAAAGAACTTTGAAAAGAGAGTTAAATAGTACGTGAAATTGTTGAAAGGGAAACGATTGAAGTCAGTCATGCTAG-TGAAAATTCAGTTTGGCGGA-TTTTG-GGTTCAGGAGT-AGGAGGCAGGGT---CAA---ACCGTTTCTCTTCTGGACTTCGAATTTGTCAGATGCACTTTTTCT-TTGGCAGGTCAGCGTCGGTTTC-GGAGGTTGTAAA---------ATACTTGGGGT-AAAGTAGCTCTGCTTCGGGA---GAGTG-TTATAGACCTTGGGGGATGCAGCCTGCGGGATCGAGGATTGCAGCAAATGC-------------------TTTTT--GGC-TTGTCGCCTGGTCTCTGG-ACGTTACCTCGCTTGTGACAACATT-CTTGCCACCGGTGGGTACTAATGCCCATTAGGTTAGAGCGATCAAAAA--TTTGCTAAGGATGCTGACGTAATGGCTTTAAACGACCCGTCTTGAAACACGGACCAAGGAGTCTAACATATGTGCGAGctgattggatgatcataactatacttcctgttcctcctcctcctgtacgtcccagtatacaaatggatggaacaagtcgaggtgaagatgatttgactcacaaattgtccgacatcttgaaggcaaatcaaaacgtaaaacgttatgaagctgatggtcatcccccacacgttgtaaacgaatttgaagcattgttacaggttcttaatgat---------------------------------------------------------------------------------------------------------tataa-tttca--------a-ttagtttaatatcaa--ttgaaatttttttacttaaa-tttattatttac-----atacaaatagtttcattgcgcagcttatatggacaatgaaatggctggtcaacctcaagctcttcagaaatctggtagacctttaaagtcaatacgtgcgcgtctcaagggtaaagaaggacgtttacgcggtaacctgatgggaaagcgtgtagatttctctgctcgtacagtaattacgggtgatccaaatatttcagttgatgaagtcggagttccgaaaagcatagcccaaaatttgacttttccagaattggtgactccctttaatattgattatcttcaaaaattagtagaaaatggtccttctacacatccaggggctaaatacgtaattagagatactggtgaaaggattgatctaaaacatatatcaggcatgactggtggcttaagattacattacggttggaaagttgaacgtcatctcaatgatggtgacatcgttatattcaatcgtcagccatttttgcataagatgtcgatgatgggacataaagttcgtgttatgccctattcgaccttccgtcttaatttatcagttacaacaccttataatgccgattttgatggtgacgaaatgaacatgcatgttccccaatcagtggaaactaaagcagaagtttcagaaatctgcatggttcctaaacaaattgtatctcctcaatcaaataaaccagttatgggtattgtacaggatactttatgtgctgttagaaaatttacaaagagggattgctttttatctaaagatttggtaatgaacatt

>Diversispora_peloponnesiaca_MN306207

AGGAATCCCTAGTAAGCATGAGTCATCAGCTCATG-TTGATTACGTCCCTGCCCTTTGTACACACCGCCCGTCGCTACTACCGATTGAATGGCTTAGTGAGACCTTTGGATTGGGGTTTAGAGATCGGAAAC---GATCTTTATTCTCCGAGAAGTCGGTCAAACTTGGTCATTTAGAGGAAGTAAAAGTCGTAACAAGGTTTCCGTAGGTGAACCTGCGGAAGGATCATT--AAAAAT-ATTTA-TCCGAGAATTCT-----TTACGT----T--------TTCCCG-G------ATTATTTGTATTCAAA-TCCCACTCTTT-------AT-AAAT-ATA---------TCAATTATATAAAAC-AAAAA-T-AAAAAAAGAAAACTTTCAACAACGGATCTCTTGGCTCTCGCATCGATGAAGAACGCAGCGAAATGCGATACGTAATGTGAATTGCAG-ATTCCGTGAATCATCGAA-TCTTTGAACGCAAATTGTACTTCCTAGTAATCTAGAAAGTATGCTTGGTTGAGGGTCATTAAAATAACA-TTCGTGAA--------------TTCTTTCG-------------CGGATTTGAG----TTTT-CC--GGCA--TTT---AT-----------AA--------TA-TAAACGTGGGTAACTTT-AAAATC-ATTT-------TTACTTGGTACAAGTT-GAAAACGTGC-TATA--TGTTT-GGTTCGCT-GACAACTTGTCCA--T-C--TC----T-ATAT-ATTATGCG-CGCACTTGG---TTTTT----AAAGCTCTGTGC--GAGT--ATATA------TTTTTTTTATGAC-CTCAGCTCAAGCAAGAATACCC-GCTGAACTTAAGCATATCAATAAGCGGAGGAAAAGAAACTAACAAGGATTCCCCTAGTAACGGCGAGTGAAGAGGGAAAAGCTCAAATTTTAAATCTACC-TGG---TTC--CCAGGTCGAATTGTAATTTGAAGAAGCGATATC-GG-TT-TTGAGGTCTGGTTCAAGTTCTTTGGAACAAGACATCAT-GG-AGGGTGAGAATCCCGTGCATGATCAGA-CC---GAGAT--AC-CTAAT---ATTCGTTTTCTAAGAGTCGAGTTGTTTGGGAATGCAGCTCAAAATGGG-T-GGTAGAC-TTCACCTAAGGCTAAATATCAGCGAGAGACCGATAGCGAACAAGTACTGTG-AA-GGAAAGATGAAAAGAACTTTGAAAAGAGAGTTAAATAGTACGTGAAATTGTTGAAAGGGAAACGATTGAAGTCAGTCATGCTAG-TGAAAATTCAGTTTGGCGGA-TTTTG-AGTTCAGGAGGTGGAAAGCAGGGTGTCAAA---CCCGTTTCTCTTCTGGACTTCGAATTTGTCGGATGCATTTTTTCT-TTGGCAGGTCAGCGTCGGTTTC-GGAGGTTGTAAA---------ATACTTGGGGT-AAAGTAGCTCTGCTTCGGGA---GAGTG-TTATAGACCTTGGGGGATGCAGCCTGCGGGATCGAGGAGTGCAGCAAATGC-------------------TTTTT--GGC-TTGTCGCCTGGTCTCTGG-ATGTTACCTCGCTTGTGACAACATT-CTTGTCACCGGTGGGTACTAATGCCCATTAGGTTAGAGCGATCAAAAA--TTTGCTAAGGATGCTGACGTAATGGCTTTAAACGACCCGTCTTGAAACACGGACCAAGGAGTCTAACATG------------------------------------------------------------------------------------------------------------------------------------------------------------------------------------------------------------------------------------------------------------------------------------------------------------------------------------------------------------------------------------------------------------------------------------------------------------------------------------------------------------------------------------------------------------------------------------------------------------------------------------------------------------------------------------------------------------------------------------------------------------------------------------------------------------------------------------------------------------------------------------------------------------------------------------------------------------------------------------------------------------------------------------------------------------------------------------------------------------------------------------------------------------------------------------------------------------------

>Diversispora_peloponnesiaca_MN306208

AGGAATCCCTAGTAAGCATGAGTCATCAGCTCATG-TTGATTACGTCCCTGCCCTTTGTACACACCGCCCGTCGCTACTACCGATTGAATGGCTTAGTGAGACCTTTGGATTGGGGTTTAGAGATCGGCAAC---GATCTTTATCCTCCGAGAAGTCGGTCAAACTTGGTCATTTAGAGGAAGTAAAAGTCGTAACAAGGTTTCCGTAGGTGAACCTGCGGAAGGATCATT--AAAAAT-ATTTA-TCCGAGAATTCT-----TTACGT----T--------TTCCCG-G------ATTATTTGTATTCAAA-TCCCACTCTTT-------AT-AAAT-ATA---------TCAATTATATAAAAC-AAAAA-A-TAAAAAAGAAAACTTTCAACAACGGATCTCTTGGCTCTCGCATCGATGAAGAACGCAGCGAAATGCGATACGTAATGTGAATTGCAG-ATTCCGTGAATCATCGAA-TCTTTGAACGCAAATTGTACTTCATAGTAATCTAGGAAGTATGCTTGGTTGAGGGTCATTAAAATAACA-TTCGTGAA--------------TTCTTTCG-------------CGGATTTGAG----TTTT-CC--AGCA--TTT---AT-----------AA--------TA-TAAACGTTGGTAACTTT-AAAATC-ATTT------ATTACTTGGTACAAGTT-GAAAACGTGC-TATA--TGTTT-GGTTCGCT-GACAACTTGTCCA--T-C--TC----T-ATAT-ATTATGCG-CGCACCTAG--TTTTTT----AAAGCTCTGTGC--GAGT--ATATA------TTTTTTTTATGAC-CTCAGCTCAAGCAAGAATACCC-GCTGAACTTAAGCATATCAATAAGCGGAGGAAAAGAAACTAACAAGGATTCCCCTAGTAACGGCGAGTGAAGAGGGAAAAGCTCAAATTTTAAATCTACC-TGG---TTC--CCAGGTCGAATTGTAATTTGAAGAAGCGATATC-GG-TG-TTGAGGTCTGGTTCAAGTTCTTTGGAACAAGACATCAT-GG-AGGGTGAGAATCCCGTGCATGATCAGA-CC---GAGAT--AC-CTAAT---ATTCGTTTTCTAAGAGTCGAGTTGTTTGGGAATGCAGCTCAAAATGGG-T-GGTAGAC-TTCACCTAAGGCTAAATATCAGCGAGAGACCGATAGCGAACAAGTACTGTG-AA-GGAAAGATGAAAAGAACTTTGAAAAGAGAGTTAAATAGTACGTGAAATTGTTGAAAGGGAAACGATTGAAGTCAGTCATGCTAG-TGAAAATTCAGTTTGGCGGA-TTTTG-AGTTCAGGAGGTGGAAAGCAGGGTGTCAAA---CCCGTTTCTCTTCTGGACTTCGAATTTGTCGGATGCATTTTTTCT-TTGGCAGGTCAGCGTCGGTTTC-GGAGGTTGTAAA---------ATACTTGGGGT-AAAGTAGCTCTGCTTCGGTA---GAGTG-TTATAGACCTTGGGGGATGCAGCCTGCGGGATCGAGGATTGCAGCAAATGC-------------------TTTTT--GGC-TTGTCGCCTGGTCTCTGG-ATGTTACCTCGCTTGTGACAACATT-CTTGTCACCGGTGGGTACTAATGCCCATTAGGTTAGAGCGATCAAAAA--TTTGCTAAGGATGCTGACGTAATGGCTTTAAACGACCCGTCTTGAAACACGGACCAAGGAGTCTAACATATGTGCGAG----------------------------------------------------------------------------------------------------------------------------------------------------------------------------------------------------------------------------------------------------------------------------------------------------------------------------------------------------------------------------------------------------------------------------------------------------------------------------------------------------------------------------------------------------------------------------------------------------------------------------------------------------------------------------------------------------------------------------------------------------------------------------------------------------------------------------------------------------------------------------------------------------------------------------------------------------------------------------------------------------------------------------------------------------------------------------------------------------------------------------------------------------------------------------------------------------------

>Diversispora_peloponnesiaca_MN306205_OL690410

AGGAATCCCTAGTAAGCATGAGTCATCAGCTCATG-TTGATTACGTCCCTGCCCTTTGTACACACCGCCCGTCGCTACTACCGATTGAATGGCTTAGTGAGACCTTTGGATTGGGGTTTAGAGATCGGCAAC---GATCTTTATCCTCCGAGAAGTCGGTCAAACTTGGTCATTTAGAGGAAGTAAAAGTCGTAACAAGGTTTCCGTAGGTGAACCTGCGGAAGGATCATT--AAAAAT-ATTTA-TCCGAGAATTCT-----TTACGT----T--------TTCCCG-G------ATTATTTGTATTCAAA-TCCCACTCTTT-------AT-AAAT-ATA---------TCAATTATATAAAAC-AAAAA-A-TAAAAAAGAAAACTTTCAACAACGGATCTCTTGGCTCTCGCATCGATGAAGAACGCAGCGAAATGCGATACGTAATGTGAATTGCAG-ATTCCGTGAATCATCGAA-TCTTTGAACGCAAATTGTACTTCCTAGTAATCTAGAAAGTATGCTTGGTTGAGGGTCATTAAAATAACA-TTCGTGAA--------------TTCTTTCG-------------CGGATTTGAG----TTTT-CC--GGCA--TTT---AT-----------AA--------TA-TAAACGTGGGTAACTTT-AAAATC-ATTT------ATTACTTGGTACAAGTT-GAAAACGTGC-TATA--TGTGT-GGTTCGCT-GACAACTTGTCCA--T-C--AT----C-ATAT-ATTATGCG-CGTTACTTA-TCTTTTT----AAAGTTCTGTGC--GAGC--ATATA------TTTTTTTTATGAC-CTCAGCTCAAGCAAGAATACCC-GCTGAACTTAAGCATATCAATAAGCGGAGGAAAAGAAACTAACAAGGATTCCCCTAGTAACGGCGAGTGAAGAGGGAAAAGCTCAAATTTTAAATCTACC-TGG---TTC--CCAGGTCGAATTGTAATTTGAAGAAGCGATATC-GG-TG-TTGAAGTCTGGTTCAAGTTCTTTGGAACAAGACATCAT-GG-AGGGTGAGAATCCCGTGCATGATCAGA-CC---GAGAT--AC-CTAAT---ATTCGTTTTCTAAGAGTCGAGTTGTTTGGGAATGCAGCTCAAAATGGG-T-GGTAGAC-TTCACCTAAGGCTAAATATCAGCGAGAGACCGATAGCGAACAAGTACTGTG-AA-GGAAAGATGAAAAGAACTTTGAAAAGAGAGTTAAATAGTACGTGAAATTGTTGAAAGGGAAACGATTGAAGTCAGTCATGCTAG-TGAAAATTCAGTTTGGCGGA-TTTTG-AGTTCAGGAGT-AGGAAGCAGGGT---CAA---ACCGTTTCTCTTCTGGACTTCGAATTTGTCGGATGCACTTTTTCT-TTGGCAGGTCAGCGTCGGTTTC-GGAGGTTGTAAA---------ATACTTGGGGT-AAAGTAGCTCTGCTTCGGGA---GAGTG-TTATAGACCTTGGGGGATGCAGCCTGCGGGATCGAGGATTGCAGCAAATGC-------------------TTTTT--GGC-TTGTCGCCTGGTCTCTGG-ACGTTACCTCGCTTGTGACAACATT-CTTGTCACCGGTGGGTACTAATGCCCATTAGATTAGAGCGGTCAAAAA--TTTGCTAAGGATGCTGACGTAATGGCTTTAAACGACCCGTCTTGAAACACGGACCAAGGAGTCTAACATATGTGCGAGctgattggatgatcataactatacttcctgttcctcctcctcctgtacgtcccagtatacaaatggatggaacaagccgaggtgaagatgatttgactcacaaattgtccgacatcttgaaggcaaatcaaaacgtaaaacgttatgaagctgatggtcatcccccacacgttgtaaacgaatttgaagcattgttacaggttcttaatgat---------------------------------------------------------------------------------------------------------tataa-tttca--------a-ttagtttaatatcaa--ttgaaatttttttacttaaa-tttattatttac-----atacaaatagtttcattgtgcaacttatatggacaatgaaatggctggtcaacctcgagctcttcagaaatctggtagacctttaaagtcaatacgtgcgcgtctcaagggtaaagaaggacgtttacgcggtaacctgatgggaaagcgtgtagatttctctgctcgtacagtaattacgggtgatccaaatatttcagttgatgaagtcggagttccgaaaagcatagctcaaaatttgacttttccanaattggtgactccctttaatattgattatcttcaaaaattagtagaaaatggtccttctacacatccaggggctaaatacgtagttagagatactggtgaaaggattgatctaaaacacatatcaggcatgactggtggcttaagattacattacggttggaaagttgaacgtcatctcaatgatggtgacatcgttatactcaatcgtcagccacctttgcacaagatgtcgatgatgggacataaagttcgtgttatgccctattcgaccttccgtcttaatttatcagttacaacaccttataatgccgattttgatggtgacgaaatgaacatgcatgttccccaatcagtggaaactaaagcagaaatttcagaaatctgcatggttcctaaacaaattgtatctcctcaatcaaataaaccagttatgggtattgtacaggatactttatgtgctgttagaaaatttacaaaaagggattgctttttatctaaagatttggtaatgaacatt

>Diversispora_celata_AM713402

----------------------------------------TTACGTCCCTGCCC-TTGTACACACCGCCCGTCGCTACTACCGATTGAATGGCTTAGTGAGACCTTTGGATTGGGATTTAGGGATCGGAAAC---GATCTTTATTTTTCGAGAAGTCGGTCAAACTTGGTCATTTAGAGGAAGTAAAAGTCGTAACAAGGTTTCCGTAGGTGAACCTGCGGAAGGATCATT--AAAAAT-ATATA-TCCGAGAA----------TTAAT----T--------TTCTCG-G------ATTATTTGTATTCAAA-TCCCACTCTTT-------AT-AAAT-ATA--------TTTAATTATATAAAAC--AAAA-T-AAAAAAAGAAAACTTTCAACAACGGATCTCTTGGCTCTCGCATCGATGAAGAACGCAGCGAAATGCGATACGTAGTGTGAATTGCAGAATTCCGTGAATCATCGAA-TCTTTGAACGCAAATTGTACTCTCCAGTAATCTGGGGAGTATGCTTGGTTGAGGGTCATTAAAATAACA--TCGTGA----------------TTTTTTG-------------CGGATTTGAG----TTTT-CC--AGTA--TTTATTAT---TAT------A--------AA-TAAATGTTGGTGACTTT-AAAATT-ATTT------ATTACTTGGTACAAGTT-GAAAACGTGC-TATA--TGTGT-GGTTCGCT-GACAACTTGTCCA--T-C--TT----T-ATAT-ATTATGCG-CGCACTTGA----ATT-----TTTTGTCTGTAC--GAGC--ATATA------TTTTTTTTATGAC-CTCAGCTCAAGCAAGAATACCC-GCTGAACTTAAGCATATCAATAAGCGGAGGAAAAGAAACTAACAAGGATTCCCCTAGTAATGGCGAATGAAGAGGGAAAAGCTCAAATTTTAAATCTACC-TGG---TTC--CCAGGTCGAGTTGTAATTTGAAGAAGCGATATC-GG-TG-TGGAGGTCTGGTTCAAGTTCTTTGGAACAAGACATCAT-GG-AGGGTGAGAATCCCGTGCATGATCAGA-CC---AAGAT--AC-TTAAT---ATTCGCTTTCTAAGAGTCGAGTTGTTTGGGAATGCAGCTCAAATTGGG-T-GGTAGAC-TTCACCTAAGGCTAAATATCAGCGAGAGACCGATAGCGAACAAGTACTGTG-AA-GGAAAGATGAAAAGAACTTTGAAAAGAGAGTTAAATAGTACGTGAAATTGTTGAAAGGGAAACGATTGAAGTCAGTCATGCCAG-TGAAAATTCAGTTTGGTGGG-TTTTG-GGTTCAGGAGT--TGAGGCAGGGT---CAA---ACCGTTTCTCTTTTGATCTTGGAATTTGTCAGATGCACTTTTTCT-TTGGCAGGTCAGCGTCGATTTC-GGAGGTTGTAAA---------ATACTTGGGGA-AAAGTAGCTCTGCTTCGGGA---GAGTGTTTATAGACCTTGGGTGATGCGGCCTGCGGGATCGAGGATTGCAGCAAATGC--------------------TTTT--GGC-TTGTCGCCTGATCTCTGG-ACGTCATCTCGCTTGTATCAACATT-CTTGGTACCGGTGAGTAATGGTGCTCA-TAGGTTAGAGCGATCAAAAA--TTTGCTAAGGATGCTGACGTAATGGCTTTAAACGACCCGTCTTGAAACACGGACCA--------------------------------------------------------------------------------------------------------------------------------------------------------------------------------------------------------------------------------------------------------------------------------------------------------------------------------------------------------------------------------------------------------------------------------------------------------------------------------------------------------------------------------------------------------------------------------------------------------------------------------------------------------------------------------------------------------------------------------------------------------------------------------------------------------------------------------------------------------------------------------------------------------------------------------------------------------------------------------------------------------------------------------------------------------------------------------------------------------------------------------------------------------------------------------------------------------------------------------

>Diversispora_celata_AM713403

---------------------------------------ATTACGTCCCTGCCCTTTGTACACACCGCCCGTCGCTACTACCGATTGAATGGCTTAGTGAGACCTTTGGATTGGGATTTAGGGATCGGAAAC---GATCTTTATTTTTCGAGAAGTCGGTCAAACTTGGTCATTTAGAGGAAGTAAAAGTCGTAACAAGGTTTCCGTAGGTGAACCTGCGGAAGGATCATTAAAAAAAT-ATATA-TCCGAGAA----------TTAAT----T--------TTCTCG-G------ATTATTTGTATTCAAA-TCCCACTCTTT-------AT-AAAT-ATA---------TTAATTATATAAAAC--AAAA-T-AAAAAAAGAAAACTTTCAACAACGGATCTCTTGGCTCTCGCATCGATGAAGAACGCAGCGAAATGCGATACGTAGTGTGAATTGCAGAATTCCGTGAATCATCGAA-TCTTTGAACGCAAATTGTACTTTCCAGTAATCTGGGGAGTATGCTTGGTTGAGGGTCATTAAAATAACA--TCGTGA---------------TTTTTTTG-------------CGGATTTGAG----TTTT-CC--AGTA--TTTATTAT---TAT------A--------AA-TATTTGTTGGTGACTTT-AAAATT-ATTT------ATAACTTGGTACAAGTT-GAAAACGTGC-TATA--TTTGT-GGTTCGCT-GACAACTTGTCCA--T-C--CTTATAT-ATAT-ATTATGCG-CGCACTTGA----TTTTT---TTTTATCTGTAC--GAGC--ATATA-----TTTTTTTTTATGAC-CTCAGCTCAAGCAAGAACACCC-GCTGAACTTAAGCATATCAATAAGCGGAGGAAAAGAAACTAACAAGGATTCCCCTAGTAATGGCGAATGAAGAGGGAAAAGCTCAAATTTTAAATCTACC-TGG---TTC--CCAGGTCGAGTTGTAATTTGAAGAAGCGATATC-GG-TG-TGGAGGTCTGGTTCAAGTTCTTTGGAACAAGACATCAT-GG-AGGGTGAGAATCCCGTGCATGATCAGA-CC---AAGAT--AC-TTAAT---ATTCGCTTTCTAAGAGTCGAGTTGTTTGGGAATGCAGCTCAAATTGGG-T-GGTAGAC-TTCACCTAAGGCTAAATATCAGCGAGAGACCGATAGCGAACAAGTACTGTG-AA-GGAAAGATGAAAAGAACTTTGAAAAGAGAGTTAAATAGTACGTGAAATTGTTGAAAGGGAAACGATTGAAGTCAGTCATGCCAG-TGAAAATTCAGTTTGGTGGG-TTTTG-GGTTCAGGAGT--TGAAGCAGGGT---CAA---ACCGTTTCTCTTTTGAACTTGGAATTTGTCAGATGCACTTTTTCT-TTGGCAGGTCAGCGTCGATTTC-GGAGGTTGTAAA---------ATACTTGGGGA-AAAGTAGCTCTGCTTCGGGA---GAGTG-TTATAGACCTTGGGTGATGCGGCCTGCGGGATCGAGGATTGCAGCAAATGC--------------------TTTT--GGC-TTGTCGCCTGATCTCTGG-ACGTCATCTCGCTTGTATCAACATT-CTTGGTACCGGTGAGTAATGGTGCTCA-TAGGTTAGAGCGATCAAAAA--TTTGCTAAGGATGCTGACGTAATGGCTTTAAACGACCCGTCTTGAAACACGGACCA--------------------------------------------------------------------------------------------------------------------------------------------------------------------------------------------------------------------------------------------------------------------------------------------------------------------------------------------------------------------------------------------------------------------------------------------------------------------------------------------------------------------------------------------------------------------------------------------------------------------------------------------------------------------------------------------------------------------------------------------------------------------------------------------------------------------------------------------------------------------------------------------------------------------------------------------------------------------------------------------------------------------------------------------------------------------------------------------------------------------------------------------------------------------------------------------------------------------------------

>Diversispora_celata_AY639225

-------------------------------------------------------------------------------------------------------------------------------------------------------------------------------------------------------------------------------------------------------------------------------------------------------------------------------------------------------------------------------------------------------------------------------------------------------------------------------------------------------------------------------------------------------------------------------------------------------------------------------------------------------------------------------------------------------------------------------------------------------------------------------------------------------------------------------------------------------------------------------------------------------------------GCATATCAATAAGCGGAGGAAAAGAAACTAACAAGGATTCCCCTAGTAATGGCGAATGAAGAGGGAAAAGCTCAAATTTTAAATCTACC-TGG---TTC--CCAGGTCGAGTTGTAATTCGAAGAAGCGATATC-GG-TG-TGGAGGTCTGGTTCAAGTTCTTTGGAACAAGACATCAT-GG-AGGGTGAGAATCCCGTGCATGATCAGA-CC---AAGAT--AC-TTAAT---ATTCGCTTTCTAAGAGTCGAGTTGTTTGGGAATGCAGCTCAAATTGGG-T-GGTAGAC-TTCACCTAAGGCTAAATATCAGCGAGAGACCGATAGCGAACAAGTACTGTG-AA-GGAAAGATGAAAAGAACTTTGAAAAGAGAGTTAAATAGTACGTGAAATTGTTGAAAGGGAAACGATTGAAGTCAGTCATGCCAG-TGAAAATTCAGTTTGGTGGG-TTTTG-GGTTCAGGAGT--TGAAGCAGGGT---CAA---ACCGTTTCTCTTTTGAACTTGGAATTTGTCAGATGCACTTTTTCT-TTGGCAGGTCAGCGTCGATTTC-GGAGGTTGTAAA---------ATACTTGGGGA-AAAGTAGCTCTGCTTCGGGA---GAGTG-TTATAGACCTTGGGTGATGCGGCCTGCGGGATCGAGGATTGCAGCAAATGC--------------------TTTT--GGC-TTGTCGCCTGATCTCTGG-ACGTCATCTCGCTTGTATCAACATTACTTGGTACCGGTGAGTAATGGTGCTCA-TAGGTTAGAGCGATCAAAAA--TTTGCTAAGGATGCTGACGTAATGGCTTTAAACGAC----------------------------------------------------------------------------------------------------------------------------------------------------------------------------------------------------------------------------------------------------------------------------------------------------------------------------------------------------------------------------------------------------------------------------------------------------------------------------------------------------------------------------------------------------------------------------------------------------------------------------------------------------------------------------------------------------------------------------------------------------------------------------------------------------------------------------------------------------------------------------------------------------------------------------------------------------------------------------------------------------------------------------------------------------------------------------------------------------------------------------------------------------------------------------------------------------------------------------------------------------

>Diversispora_eburnea_AM713407

AGGAATCCCTAGTAAGCGTGAGTCATCAGCTCACG-TTGATTACGTCCCTGCCCTTTGTACACACCGCCCGTCGCTACTACCGATTGAATGGCTTAGTGAGACCTTTGGATTGGGATTTAGGGATCGGAAAC---GATCTTTATTTTCCGAGAAGTCGGTCAAACTTGGTCATTTAGAGGAAGTAAAAGTCGTAACAAGGTTTCCGTAGGTGAACCTGCGGAAGGATCATT--AAAAAT-ATATA-ACCGAGAA----------TTAAT----T--------TTCCCG-G------ATTATTTGTATTCAAA-TCCCACTCTTT-------AT-AAAT-ATA---------TCAATTATACAAAAA-CAAAA-T-TAAAAAAGAAAACTTTCAACAACGGATCTCTTGGCTCTCGCATCGATGAAGAACGCAGCGAAATGCGATACGTAGTGTGAATTGCAGAATTCCGTGAATCATCGAA-TCTTTGAACGCAAATTGTACTTTCCAGTAATCTGGGAAGTATGCTTGGTTGAGGGTCATTAAATTAACA-TTCGTGAAT-----------TTTTTTTTTG-------------CGGATTTGAG----TTTT-CC--AGTA--TTT---AT---TAT------A--------AA-TAAATGTTGGTGACTTT-AAAATT-ATTT------ATTACTTGGTACAAGTT-GAAAACGTGC-TATA--TGTGT-GGTTCGCT-GACAACTTGTCCA--T-C--TC----T-ATAT-ATTATGCG-CGCACTTGG------TT----TTAAATCTGTAC--GAGC--ATATA-----TTTTTTTTTATGAC-CTCAGCTCAAGCAAGAATACCC-GCTGAACTTAAGCATATCAATAAGCGGAGGAAAAGAAACTAACAAGGATTCCCCTAGTAATGGCGAATGAAGAGGGAAAAGCTCAAATTTTAAATCTACC-TGG---TTC--CCAGGTCGAATTGTAATTTGAAGAAACGATGTC-GG-TG-TTAAGGTCTGGTTCAAGTTCTTTGGAACAAGACATCAT-GG-AGGGTGAGAATCCCGTGCATGATCAGA-CC---GAGAT--AC-TTAAT---ATTCGTTCTCTAAGAGTCGAGTTGTTTGGGAATGCAGCTCAAAATGGG-T-GGTAGAC-TTCACCTAAGGCTAAATATCAGCGAGAGACCGATAGCGAACAAGTACTGTG-AA-GGAAAGATGAAAAGAACTTTGAAAAGAGAGTTAAATAGTACGTGAAATTGTTGAAAGGGAAACGATTGAAGTCAGTCATGCTAG-TGAAAATTCAGTTTGGCGGA-TTTTG-GGTTTAGGAGT--AGAGGCAGGGT---CAA---ACCGTTTCTCTTTTGAACTTGGAATTTGTCAGATGCACTTTTTCT-TTGGCAGGTCAGCGTCGATTTC-GGAGGTTGTAAA---------ATACTTGGGGT-AAAGTAGCTCTGCTTCGGGA---GAGTG-TTATAGACCTTGGGGGATGCAGCCTGCGGGATCGAGGATTGCAGCAAATGC--------------------TTTT--GGC-TTGTCGCCTGATCTCTGG-ATGTTACCTCGCTTGTAACAACATT-CTTGTTGCCGGTGAGTACTAATGCCTATTAGGTTAGAGCGATCAAAAA-TTTTGCTAAGGATGCTGACGTAATGGCTTTAAACGACCCGTCTTGAAACACGGACCAAGGAGTCTAACATATGTGCGAG----------------------------------------------------------------------------------------------------------------------------------------------------------------------------------------------------------------------------------------------------------------------------------------------------------------------------------------------------------------------------------------------------------------------------------------------------------------------------------------------------------------------------------------------------------------------------------------------------------------------------------------------------------------------------------------------------------------------------------------------------------------------------------------------------------------------------------------------------------------------------------------------------------------------------------------------------------------------------------------------------------------------------------------------------------------------------------------------------------------------------------------------------------------------------------------------------------

>Diversispora_eburnea_AM713408

AGGAATCCCTAGTAAGCGTGAGTCATCAGCTCACG-TTGATTACGTCCCTGCCCTTTGTACACACCGCCCGTCGCTACTACCGATTGAATGGCTTAGTGAGACCTTTGGATTGGGATTTAGGGATCGGAAAC---GATCTTTATTTTCCGAGAAGTCGGTCAAACTTGGTCATTTAGAGGAAGTAAAAGTCGTAACAAGGTTTCCGTAGGTGAACCTGCGGAAGGATCATT--AAAAAT-ATATA-ACCGAGAA----------TTAAT----T--------TTCCCG-G------ATTATTTGTATTCAAATTCCCACTCTTT-------AT-AAAT-ATA---------TCAATTATACAAAAA-CAAAA-T-TAAAAAAGAAAACTTTCAACAACGGATCTCTTGGCTCTCGCATCGATGAAGAACGCAGCGAAATGCGATACGTAGTGTGAATTGCAGAATTCCGTGAATCATCGAA-TCTTTGAACGCAAATTGTACTTTCCAGTAATCTGGGAAGTATGCTTGGTTGAGGGTCATTAAATTAACA-TTCGTGAAT-----------TTTTTTTTTG-------------CGGATTTGAG----TTTT-CC--AGTA--TTT---AT---TAT------A--------AA-TAAATGTTGGTGACTTT-AAAATT-ATTT------ATTACTTGGTACAAGTT-GAAAACGTGC-TATA--TGTGT-GGTTCGCT-GACAACTTGTCCA--T-C--TC----T-ATAT-ATTATGCG-CGCACTTGG------TT----TTAAATCTGTAC--GAGC--ATATA-----TTTTTTTTTATGAC-CTCAGCTCAAGCAAGAATACCC-GCTGAACTTAAGCATATCAATAAGCGGAGGAAAAGAAACTAACAAGGATTCCCCTAGTAATGGCGAATGAAGAGGGAAAAGCTCAAATTTTAAATCTACC-TGG---TTC--CCAGGTCGAATTGTAATTTGAAGAAACGATGTC-GG-TG-TTAAGGTCTGGTTCAAGTTCTTTGGAACAAGACATCAT-GG-AGGGTGAGAATCCCGTGCATGATCAGA-CC---GAGAT--AC-TTAAT---ATTCGTTCTCTAAGAGTCGAGTTGTTTGGGAATGCAGCTCAAAATGGG-T-GGTAGAC-TTCACCTAAGGCTAAATATCAGCGAGAGACCGATAGCGAACAAGTACTGTG-AA-GGAAAGATGAAAAGAACTTTGAAAAGAGAGTTAAATAGTACGTGAAATTGTTGAAAGGGAAACGATTGAAGTCAGTCATGCTAG-TGAAAATTCAGTTTGGCGGA-TTTTG-GGTTTAGGAGT--AGAGGCAGGGT---CAA---ACCGTTTCTCTTTTGAACTTGGAATTTGTCAGATGCACTTTTTCT-TTGGCAGGTCAGCGTCGATTTC-GGAGGTTGTAAA---------ATACTTGGGGT-AAAGTAGCTCTGCTTCGGGA---GAGTG-TTATAGACCTTGGGGGATGCAGCCTGCGGGATCGAGGATTGCAGCAAATGC--------------------TTTT--GGC-TTGTCGCCTGATCTCTGG-ATGTTACCTCGCTTGTAACAACATT-CTTGTTGCCGGTGAGTACTAATGCCTATTAGGTTAGAGCGATCAAAAA-TTTTGCTAAGGATGCTGACGTAATGGCTTTAAACGACCCGTCTTGAAACACGGACCAAGGAGTCTAACATATGTGCGAG----------------------------------------------------------------------------------------------------------------------------------------------------------------------------------------------------------------------------------------------------------------------------------------------------------------------------------------------------------------------------------------------------------------------------------------------------------------------------------------------------------------------------------------------------------------------------------------------------------------------------------------------------------------------------------------------------------------------------------------------------------------------------------------------------------------------------------------------------------------------------------------------------------------------------------------------------------------------------------------------------------------------------------------------------------------------------------------------------------------------------------------------------------------------------------------------------------

>Diversispora_eburnea_AM713411

AGGAATCCCTAGTAAGCGTGAGTCATCAGCTCACG-TTGATTACGTCCCTGCCCTTTGTACACACCGCCCGTCGCTACTACCGATTGAATGGCTTAGTGAGACCTTTGGATTGGGATTTAGGGATCGGAAAC---GATCTTTATTTTCCGAGAAGTCGGTCAAACTTGGTCATTTAGAGGAAGTAAAAGTCGTAACAAGGTTTCCGTAGGTGAACCTGCGGAAGGATCATT--AAAAAT-ATATA-ACCGAGAA----------TTAAT----T--------TTCCCG-G------ATTATTTGTATTCAAA-TCCCACTCTTT-------AT-AAAT-ATA---------TCAATTATACAAAAA-CAAAA-T-TAAAAAAGAAAACTTTCAACAACGGATCTCTTGGCTCTCGCATCGATGAAGAACGCAGCGAAATGCGATACGTAGTGTGAATTGCAGAATTCCGTGAATCATCGAA-TCTTTGAACGCAAATTGTACTTTCCAGTAATCTGGGAAGTATGCTTGGTTGAGGGTCATTAAATTAACA-TTCGTGAA------------TTTTTTTTTG-------------CGGATTTGAG----TTTT-CC--AGTA--TTT---AT---TAT------A--------AA-TAAATGTTGGTGACTTT-AAAATT-ATTT------ATTACTTGGTACAAGTT-GAAAACGTGC-TATA--TGTGT-GGTTCGCT-GACAACTTGTCCA--T-C--TC----T-ATAT-ATTATGCG-CGCACTTGG--TTTTTT----TTTAATCTGTAC--GAGC--ATATA------TTTTTTTTATGAC-CTCAGCTCAAGCAAGAATACCC-GCTGAACTTAAGCATATCAATAAGCGGAGGAAAAGAAACTAACAAGGATTCCCCTAGTAATGGCGAATGAAGAGGGAAAAGCTCAAATTTTAAATCTACC-TGG---TTC--CCAGGTCGAATTGTAATTTGAAGAAACGATATC-GG-TG-TTAAGGTCTGGTTCAAGTTCTTTGGAACAAGACATCAT-GG-AGGGTGAGAATCCCGTGCATGATCAGA-CC---GAGAT--AC-TTAAT---ATTCGTTCTCTAAGAGTCGAGTTGTTTGGGAATGCAGCTCAAAATGGG-T-GGTAGAC-TTCACCTAAGGCTAAATATCAGCGAGAGACCGATAGCGAACAAGTACTGTG-AA-GGAAAGATGAAAAGAACTTTGAAAAGAGAGTTAAATAGTACGTGAAATTGTTGAAAGGGAAACGATTGAAGTCAGTCATGCTAG-TGAAAATTCAGTTTGGCGGA-TTTTG-GGTTTAGGAGT--AGAGGCAGGGT---CAA---ACCGTTTCTCTTTTGAACTTGGAATTTGTCAGATGCACTTTTTCT-TTGGCAGGTCAGCGTCGATTTC-GGAGGTTGTAAA---------ATACTTGGGGT-AAAGTAGCTCTGCTTCGGGA---GAGTG-TTATAGACCTTGGGGGATGCAGCCTGCGGGATCGAGGATTGCAGCAAATGC-------------------TTTTT--GGC-TTGTCGCCTGATCTCTGG-ATGTTACCTCGCTTGTAACAACATT-CTTGTTGCCGGTGAGTACTAATGCCTATTAGGTTAGAGCGATCAAAAA-TTTTGCTAAGGATGCTGACGTAATGGCTTTAAACGACCCGTCTTGAAACACGGACCAAGGAGTCTAACATATGTGCGAG----------------------------------------------------------------------------------------------------------------------------------------------------------------------------------------------------------------------------------------------------------------------------------------------------------------------------------------------------------------------------------------------------------------------------------------------------------------------------------------------------------------------------------------------------------------------------------------------------------------------------------------------------------------------------------------------------------------------------------------------------------------------------------------------------------------------------------------------------------------------------------------------------------------------------------------------------------------------------------------------------------------------------------------------------------------------------------------------------------------------------------------------------------------------------------------------------------

>Diversispora_eburnea_AM713406

AGGAATCCCTAGTAAGCGTGAGTCATCAGCTCACG-TTGATTACGTCCCTGCCCTTTGTACACACCGCCCGTCGCTACTACCGATTGAATGGCTTAGTGAGACCTTTGGATTGGGATTTAGGGATCGGAAAC---GATCTTTATTTTCCGAGAAGTCGGTCAAACTTGGTCATTTAGAGGAAGTAAAAGTCGTAACAAGGTTTCCGTAGGTGAACCTGCGGAAGGATCATT--AAAAAT-ATATA-ACCGAGAA----------TTAAT----T--------TTCCCG-G------ATTACTTGTATTCAAATCCCCACTCTTT-------AT-AAAT-ATA---------TCAATTATATAAAAA-CAAAA-T-AAAAAAAGAAAACTTTCAACAACGGATCTCTTGGCTCTCGCATCGATGAAGAACGCAGCGAAATGCGATACGTAGTGTGAATTGCAGAATTCCGTGAATCATCGAA-TCTTTGAACGCAAATTGTACTTTCCAGTAATCTGGAGAGTGTGCTTGGTTGAGGGTCATTAAATTAACA-TTCGCGAA------------TTTTTTTTTG-------------CGGATTTGAG----TTTT-CC--AGTA--TTT---AT---TAT------A--------AA-TAAATGTTGGTGACTTT-AAAATT-ATTT------ATTACTTGGTTCAAGTT-GAAAACGTGC-TATA--TGTGT-GGTTCGCT-GACAACTTGTCCA--T-C--TC-------TAT-ATTATGCG-CGCACTTGG---TTTTT----TTTAATCTGTAC--GAGC--ATATA-----TTTTTTTTTATGAC-CTCAGCTCAAGCAAGAATACCC-GCTGAACTTAAGCATATCAATAAGCGGAGGAAAAGAAACTAACAAGGATTCCCCTAGTAATGGCGAATGAAGAGGGAAAAGCTCAAATTTTAAATCTACC-TGG---TTC--CCAGGTCGAATTGTAATTTGAAGAAACGATATC-GG-TG-TTAAGGTCTGGTTCAAGTTCTTTGGAACAAGACATCAT-GG-AGGGTGAGAATCCCGTGCATGATCAGA-CC---GAGAT--AC-TTAAT---ATTCGTTCTCTAAGAGTCGAGTTGTTTGGGAATGCAGCTCAAAATGGG-T-GGTAGAC-TTCACCTAAGGCTAAATATCAGCGAGAGACCGATAGCGAACAAGTACTGTG-AA-GGAAAGATGAAAAGAACTTTGAAAAGAGAGTTAAATAGTACGTGAAATTGTTGAAAGGGAAACGATTGAAGTCAGTCATGCTAG-TGAAAATTCAGTTTGGCGGA-TTTTG-GGTTTAGGAGT--AGAGGCAGGGT---CAA---ACCGTTTCTCTTTTGAACTTGGAATTTGTCAGATGCACTTTTTCT-TTGGCAGGTCAGCGTCGATTTC-GGAGGTTGTAAA---------ATACTTGGGGT-AAAGTAGCTCTGCTTCGGGA---GAGTG-TTATAGACCTTGGGGGATGCAGCCTGCGGGATCGAGGATTGCAGCAAATAC--------------------CTTT--GGC-TTGTCGCCTGATCTCTGG-ATGTTACCTCGCTTGCAACAACATT-CTTGTTGCCGGTGAGTACTAATGCCTATTAGGTTAGAGCGATCAAAAA-TTTTGCTAAGGATGCTGACGTAATGGCTTTAAACGACCCGTCTTGAAACACGGACCAAGGAGTCTAACATATGTGCGAG----------------------------------------------------------------------------------------------------------------------------------------------------------------------------------------------------------------------------------------------------------------------------------------------------------------------------------------------------------------------------------------------------------------------------------------------------------------------------------------------------------------------------------------------------------------------------------------------------------------------------------------------------------------------------------------------------------------------------------------------------------------------------------------------------------------------------------------------------------------------------------------------------------------------------------------------------------------------------------------------------------------------------------------------------------------------------------------------------------------------------------------------------------------------------------------------------------

>Diversispora_slowinskiensis_KT444717_MG459197

AGGAATCCCTAGTAAGCATGAGTCATCAGCTCATG-TTGATTACGTCCCTGCCCTTTGTACACACCGCCCGTCGCTACTACCGATTGAATGGCTTAGTGAGACCTTTGGATTGGGGTTTAGGGATCGGCAAC---GATCCTTATTCTTCGAGAAGTCGGTCAAACTTGGTCATTTAGAGGAAGTAAAAGTCGTAACAAGGTTTCCGTAGGTGAACCTGCGGAAGGATCATT-AAAAAAT-TTTTA-TCCGGGAATTCT-----TTTCGT----T--------TTCCCG-G------ATTATTTGTATTCAAA-TCCCACTCTTT-------AT-AAAT-ATA---------TCAATTATATAAAAC--AAAA-A-TAAAAAAGAAAACTTTCAACAACGGATCTCTTGGCTCTCGCATCGATGAAGAACGCAGCGAAATGCGATACGTAGTGTGAATTGCAGAATTCCGTGAATCATCGAA-TCTTTGAACGCAAATTGTACTTTCCAGTAATCTGGGGAGTATGCTTGGTTGAGGGTCATTGAAATAACA-TTCGTGAA----------------TTTTCG-------------CGGATTTGAG----TTTT-CC--AGTA--TTTATTAT---TAT---------------AA-TAAATGTTGGTAACTTT-AAAATT-ATTT------ATTACTTGGTACAAGTT-GAAGACGTGC-TATA--TATGT-GGTTCGCT-GACAACTTGTCCA--T-C--T-----T-ATAT-ATTATGCG-CGCGCTTAG----TATT----ACACTTTTGTGC--AAGC--ATATA------TTTTTTTTTTGAC-CTCAGCTCAAGCAAGAATACCC-GCTGAACTTAAGCATATCAATAAGCGGAGGAAAAGAAACTAACAAGGATTCCCCTAGTAACGGCGAGTGAAGAGGGAAAAGCTCAAATTTTAAATCTACC-TGGT--TTC--CCAGGTCGAATTGTAATTTGAAGAAGCGATATC-GG-TG-TTGAGGTCTGGTTCAAGTTCTTTGGAACAAGACATCATGGG-AGGGTGAGAATCCCGTGCGTGATCAGA-CC---AAGAT--AC-TTAAT---ATTCGTTTTCTAAGAGTCGAGTTGTTTGGGAATGCAGCTCAAAATGGG-T-GGTAGAC-TTCACCTAAGGCTAAATATCAGCGAGAGACCGATAGCGAACAAGTACTGTG-AA-GGAAAGATGAAAAGAACTTTGAAAAGAGAGTTAAATAGTACGTGAAATTGTTGAAAGGGAAACGATTGAAGTCAGTCATGCTAG-TGAAAATTCAGTTTGACGGG-TTTTA-GGTTTGGGAGT--AGAATCAGGGT---TAA---ACCGTTTCTCTTTTGAACTTTTAATTTGTTGGATGCACTTTTTCT-TTGGCAGGTCAGCGTCGATTTC-GGAGGTTGTAAA---------ATACTTGGGGG-AAAGTAGCTCTGCTTCGGGA---GAGTG-TTATAGACCCTGGGGGATGCGGCCTGCGGGACCGAGGATTGCAGCAAATGC--------------------TATT--GGC-TTGTCGCCTGATCTCTAG-ATGTTACCTCGCTTGTGACAACATT-CTTGCCACCGGTGGGTACTAATGCCTATTAGGTTAGAGCGATCAAAAA-TTTTGCTAAGGATGCTGACGTAATGGCTTTAAACGACCCGTCTTGAAACACGGACCAAGGAGTCTAACATATGTGCGAG------------------------------------------------------------------------------gtggagaagatgatttgacacacaaattgtccgacatcttgaaggcaaatcaaaacgtaaaacgttatgaagccgatggtcatcctccacacgttgtaaacgaatttgaagcattgttacaggttcttaatgat---------------------------------------------------------------------------------------------------------tataa-tttca--------a-ttagtttaatatcaa--ttgaaa--tttatacttaaa-tttattatttac-----atgcaaacagtttcattgtgcaacttatatggacaatgaaatggctggtcaacctcaagctcttcagaaatctggtagacctttaaagtcaatacgtgcgcgtctcaagggtaaagaaggacgtttacgcggtaatctgatgggaaagcgcgtagatttctctgctcgtacagtaattacgggtgatccaaatatttcagtcgatgaagtcggagttccgaaaagcatagctcaaaatttgacatttccagaattggtgactccctttaatattgattatcttcaaaaattagtagaaaatggtccttctacacatccaggagctaaatacgtaattagagatactggcgaaaggattgatctaaaacatatatcaggcatgactggtggcttaagattacactacggttggaaagttgaacgtcatctcaatgatggtgacatcgtaatattcaatcgtcaaccatctttgcacaaaatgtcaatgatggg-------------------------------------------------------------------------------------------------------------------------------------------------------------------------------------------------------------------------------------------------------------------------------------

>Diversispora_slowinskiensis_KT444719

AGGAATCCCTAGTAAGCATGAGTCATCAGCTCATG-TTGATTACGTCCCTGCCCTTTGTACACACCGCCCGTCGCTACTACCGATTGAATGGCTTAGTGAGACCTTTGGATTGGGGTTTAGGGATCGGCAAC---GATCCTTATTCTTCGAGAAGTCGGTCAAACTTGGTCATTTAGAGGAAGTAAAAGTCGTAACAAGGTTTCCGTAGGTGAACCTGCGGAAGGATCATT-AAAAAAT-TTTTA-TCCGGGAATTCT-----TTTCGT----T--------TTCCCG-G------ATTATTTGTATTCAAA-TCCCACTCTTT-------AT-AAAT-ATA---------TCAATTATATAAAAC--AAAA-A-TAAAAAAGAAAACTTTCAACAACGGATCTCTTGGCTCTCGCATCGATGAAGAACGCAGCGAAATGCGATACGTAGTGTGAATTGCAGAATTCCGTGAATCATCGAA-TCTTTGAACGCAAATTGTACTTTCCAGTAATCTGGGGAGTATGCTTGGTTGAGGGTCATTGAAATAACA-TTCGTGAA----------------TTTTCG-------------CGGATTTGAG----TTTT-CC--AGTA--TTTATTAT---TAT---------------AA-TAAATGTTGGTAACTTT-AAAATT-ATTT------ATTACTTGGTACAAGTT-GAAGACGTGC-TATA--TATGT-GGTTCGCT-GACAACTTGTCCA--T-C--T-----T-ATAT-ATTATGCG-CGCGCTTAG----TATT----ACACTTTTGTGC--AAGC--ATATA------TTTTTTTTTTGAC-CTCAGCTCAAGCAAGAATACCC-GCTGAACTTAAGCATATCAATAAGCGGAGGAAAAGAAACTAACAAGGATTCCCCTAGTAACGGCGAGTGAAGAGGGAAAAGCTCAAATTTTAAATCTACC-TGGT--TTC--CCAGGTCGAATTGTAATTTGAAGAAGCGATATC-GG-TG-TTGAGGTCTGGTTCAAGTTCTTTGGAACAAGACATCAT-GG-AGGGTGAGAATCCCGTGCATGATCAGA-CC---AAGAT--AC-TTAAT---ATTCGTTTTCTAAGAGTCGAGTTGTTTGGGAATGCAGCTCAAAATGGG-T-GGTAGAC-TTCACCTAAGGCTAAATATCAGCGAGAGACCGATAGCGAACAAGTACTGTG-AA-GGAAAGATGAAAAGAACTTTGAAAAGAGAGTTAAATAGTACGTGAAATTGTTGAAAGGGAAACGATTGAAGTCAGTCATGCTAG-TGAAAATTCAGTTTGACGGG-TTTTA-GGTTTAGGAGT--AGAATCAGGGT---TAA---ACCGTTTCTCTTTTGAACTTTTAATTTGTTGGATGCACTTTTTCT-TTGGCAGGTCAGCGTCGATTTC-GGAGGTTGTAAA---------ATACTTGGGGG-AAAGTAGCTCTGCTTCGGGA---GAGTG-TTATAGACCCTGGGGGATGCGGCCTGCGGGACCGAGGATTGCAGCAAATGC--------------------TATT--GGC-TTGTCGCCTGATCTCTGG-ATGTTACCTCGCTTGTGACAACATT-CTTTCCACCGGTGAGTACTAATGCCCATTAGGTTAGAGCGATCAAAAA-TTTTGCTAAGGATGCTGACGTAATGGCTTTAAACGACCCGTCTTGAAACACGGACCAAGGAGTCTAACATATGTGCGAG----------------------------------------------------------------------------------------------------------------------------------------------------------------------------------------------------------------------------------------------------------------------------------------------------------------------------------------------------------------------------------------------------------------------------------------------------------------------------------------------------------------------------------------------------------------------------------------------------------------------------------------------------------------------------------------------------------------------------------------------------------------------------------------------------------------------------------------------------------------------------------------------------------------------------------------------------------------------------------------------------------------------------------------------------------------------------------------------------------------------------------------------------------------------------------------------------------

>Diversispora_slowinskiensis_KT444718

AGGAATCCCTAGTAAGCATGAGTCATCAGCTCATG-TTGATTACGTCCCTGCCCTTTGTACACACCGCCCGTCGCTACTACCGATTGAATGGCTTAGTGAGACCTTTGGATTGGGGTTTAGGGATCGGCAAC---GATCCTTATTCTTCGAGAAGTCGGTCAAACTTGGTCATTTAGAGGAAGTAAAAGTCGTAACAAGGTTTCCGTAGGTGAACCTGCGGAAGGATCATT-AAAAAAT-TTTTA-TCCGGGAATTCT-----TTTCGT----T--------TTCCCG-G------ATTATTTGTATTCAAA-TCCCACTCTTT-------AT-AAAT-ATA---------TCAATTATATAAAAC--AAAA-A-TAAAAAAGAAAACTTTCAACAACGGATCTCTTGGCTCTCGCATCGATGAAGAACGCAGCGAAATGCGATACGTAGTGTGAATTGCAGAATTCCGTGAATCATCGAA-TCTTTGAACGCAAATTGTACTTTCCAGTAATCTGGGGAGTATGCTTGGTTGAGGGTCATTGAAATAACA-TTCGTGAA----------------TTTTCG-------------CGGATTTGAG----TTTT-CC--AGTA--TTC---AT---TTT---------------AA-TAAATGCTGGTGACTTT-AAAATT-ATTT------ATTACTTGGTACAAGTT-GAAGACGTGC-TATA--TATGT-GGTTCGCT-GACAACTTGTCCA--T-C--T-----T-ATAT-ATTATGCG-CGCGCTTAG----TATT----ACACTTTTGTGC--AAGC--ATATA------TTTTTTTTTTGAC-CTCAGCTCAAGCAAGAATACCC-GCTGAACTTAAGCATATCAATAAGCGGAGGAAAAGAAACTAACAAGGATTCCCCTAGTAACGGCGAGTGAAGAGGGAAAAGCTCAAATTTTAAATCTACC-TGGT--TTC--CCAGGTCGAATTGTAATTTGAAGAAGCGATATC-GG-TG-TTGAGGTCTGGTTCAAGTTCTTTGGAACAAGACATCAT-GG-AGGGTGAGAATCCCGTGCATGATCAGA-CC---AAGAT--AC-TTAAT---ATTCGTTTTCTAAGAGTCGAGTTGTTTGGGAATGCAGCTCAAAATGGG-T-GGTAGAC-TTCACCTAAGGCTAAATATCAGCGAGAGACCGATAGCGAACAAGTACTGTG-AA-GGAAAGATGAAAAGAACTTTGAAAAGAGAGTTAAATAGTACGTGAAATTGTTGAAAGGGAAACGATTGAAGTCAGTCATGCTAG-TGAAAATTCAGTTTGACGGG-TTTTA-GGTTTAGGAGT--AGAATCAGGGT---TAA---ACCGTTTCTCTTTTGAACTTTTAATTTGTTGGATGCACTTTTTCT-TTGGCAGGTCAGCGTCGATTTC-GGAGGTTGTAAA---------ATACTTGGGGG-AAAGTAGCTCTGCTTCGGGA---GAGTG-TTATAGACCCTGGGGGATGCGGCCTGCGGGACCGAGGATTGCAGCAAATGC--------------------TATT--GGC-TTGTCGCCTGATCTCTGG-ATGTTACCTCGCTTGTGACAACATTTCTTGCCACCGGTGAGTACTAATGCCCATTAGGTTAGAGCGATCAAAAA-TTTTGCTAAGGATGCTGACGTAATGGCTTTAAACGACCCGTCTTGAAACACGGACCAAGGAGTCTAACATATGTGCGAG----------------------------------------------------------------------------------------------------------------------------------------------------------------------------------------------------------------------------------------------------------------------------------------------------------------------------------------------------------------------------------------------------------------------------------------------------------------------------------------------------------------------------------------------------------------------------------------------------------------------------------------------------------------------------------------------------------------------------------------------------------------------------------------------------------------------------------------------------------------------------------------------------------------------------------------------------------------------------------------------------------------------------------------------------------------------------------------------------------------------------------------------------------------------------------------------------------

>Diversispora_slowinskiensis_KT444720_MG459198

AGGAATCCCTAGTAAGCATGAGTCATCAGCTCATG-TTGATTACGTCCCTGCCCTTTGTACACACCGCCCGTCGCTACTACCGATTGAATGGCTTAGTGAGACCTTTGGATTGGGGTTTAGGGATCGGCAAC---GATCCTTATTCTTCGAGAAGTCGGTCAAACTTGGTCATTTAGAGGAAGTAAAAGTCGTAACAAGGTTTCCGTAGGTGAACCTGCGGAAGGATCATT-AAAAAAT-TTTTA-TCCGGGAATTCT-----TTTCGT----T--------TTCCCG-G------ATTATTTGTATTCAAA-TCCCACTCTTT-------AT-AAAT-ATA---------TCAATTATATAAAAC--AAAA-T-AAAAAAAGAAAACTTTCAACAACGGATCTCTTGGCTCTCGCATCGATGAAGAACGCAGCGAAATGCGATACGTAGTGTGAATTGCAG-ATTCCGTGAATCATCGAA-TCTTTGAACGCAAATTGTACTTTCCAGTAATCTGGGAAGTATGCTTGGTTGAGGGTCATTAAAATAACA-TTCGTG-----------------TTTTTCG-------------CGGATTTGAG----TTTT-CC--AGTA--TTC---AT---TTT---------------AA-TAAATGCTGGTGACTTT-AAAATT-ATTT------ATTACTTGGTACAAGTT-GAAAACGTGC-TATA--TATGT-GGTTCGCT-GACAACTTGTCCA--T-C--T-----T-ATAT-ATTATGCG-CGCGCTTAG----TTTT-----ACACTTTGTGC--AAGC--ATATA------TTTTTTTTTTGAC-CTCAGCTCAAGCAAGAATACCC-GCTGAACTTAAGCATATCAATAAGCGGAGGAAAAGAAACTAACAAGGATTCCCCTAGTAACGGCGAGTGAAGAGGGAAAAGCTCAAATTTTAAATCTACC-TGGT--TTC--CCAGGTCGAATTGTAATTTGAAGAAGCGATATC-GG-TG-TTGAAGTCTGGTTCAAGTTCTTTGGAACAAGACATCAT-GG-AGGGTGAGAATCCCGTGCATGATCAGA-CC---AAGAT--AC-TTAAT---ATTCGTTTTCTAAGAGTCGAGTTGTTTGGGAATGCAGCTCAAAATGGG-T-GGTAGAC-TTCACCTAAGGCTAAATATCAGCGAGAGACCGATAGCGAACAAGTACTGTG-AA-GGAAAGATGAAAAGAACTTTGAAAAGAGAGTTAAATAGTACGTGAAATTGTTGAAAGGGAAACGATTGAAGTCAGTCATGCTAG-TGAAAATTCAGTTTGACGGG-TTTTA-GGTTTAGGAGT--AGAATCAGGGT---TAA---ACCGTTTCTCTTTTGAACTTTTAATTTGTTGGATGCACTTTTTCT-TTGGCAGGTCAGCGTCGATTTC-GGAGGTTGTAAA---------ATACTTGGGGG-AAAGTAGCTCTGCTTCGGGA---GAGTG-TTATAGACCCTGGGGGATGCGGCCTGCGGGACCGAGGATTGCAGCAAATGC--------------------TATT--GGC-TTGTCGCCTGATCTCTGG-ACGTTACCTCGCTTGTGACAACATT-CTTGCCACCGGTGGTTACTAATGCCTATTAGGTTAGAGCGATCAAAAA-TTTTGCTAAGGATGCTGACGTAATGGCTTTAAACGACCCGTCTTGAAACACGGACCAAGGAGTCTAACATATATGCGAG------------------------------------------------------------------------------gtggagaagatgatttgacacacaaattgtccgacatcttgaaggcaaatcaaaacgtaaaacgttatgaagccgatggtcatcctccacacgttgtaaacgaatttgaagcattgttacaggttcttaatgat---------------------------------------------------------------------------------------------------------tataa-tttca--------a-ttagtttaatatcaa--ttgaaa--tttatacttaaa-tttattatttac-----atgcaaacagtttcattgtgcaacttatatggacaatgaaatggctggtcaacctcaagctcttcagaaatctggtagacctttaaagtcaatacgtgcgcgtctcaagggtaaagaaggacgtttacgcggtaatctgatgggaaagcgcgtagatttctctgctcgtacagtaattacgggtgatccaaatatttcagtcgatgaagtcggagttccgaaaagcatagctcaaaatttgacatttccagaattggtgactccctttaatattgattatcttcaaaaattagtagaaaatggtccttctacacatccaggagctaaatacgtaattagagatactggcgaaaggattgatctaaaacatatatcaggcatgactggtggcttaagattacactacggttggaaagttgaacgtcatctcaatgatggtgacatcgtaatattcaatcgtcaaccatctttgcacaaaatgtcaatgatggg-------------------------------------------------------------------------------------------------------------------------------------------------------------------------------------------------------------------------------------------------------------------------------------

>Diversispora_epigaea_FM876814_HG315981

AGGAATCCCTAGTAAGCATGAGTCATCAGCTCATG-TTGATTACGTCCCTGCCCTTTGTACACACCGCCCGTCGCTACTACCGATTGAATGGCTTAGTGAGACCTTTGGATTGGGGTTTAAGAATCGGCAAC---GAATTTTATTCTCCGAGAAGTCGGTCAAACTTGGTCATTTAGAGGAAGTAAAAGTCGTAACAAGGTTTCCGTAGGTGAACCTGCGGAAGGATCATT--AAAAAC-TTTTA-TCCGGGAATTCG-----TTTCGT----T--------TTCTCG-G------ATTATTTGTATTCAAA-TCCCACTCTTT-------AT-AAATAATA---------TCAATTATATAAAAC--AATA-T--AAAAAAGAAAACTTTCAACAACGGATCTCTTGGCTCTCGCATCGATGAAGAACGCAGCGAAATGCGATACGTAATGTGAATTGCAGAATTCCGTGAATCATCGAA-TCTTTGAACACAAATTGTACTTTTCAGTAATCTGGGAAGTATGCTTGGTTGAGGGTCATTAAAATAACA-TTCGTGAT--------------TTTTTTCG-------------CGGATTTGAG----TTTT-CC--AGTA--TTT---AT---TAT-----AA--------AA-TAAATGTTGGTAACTTT-AAAATT-ATTT------ATAACTTGGTACAAGTT-GAAAACGTGC-TATA--TGTGT-GGTTCGCT-GACAACTTGTCCA--C-C--TT----T-ATAT-ATTATGCG-CGTGCTTGGTTTTTTTT----AAAGCTGTGTGC--GAGT--ATATC------TTTTTTTTATGAC-CTCAGCTCAAGCAAGAAAACCC-GCTGAACTTAAGCATATCAATAAGCGGAGGAAAAGAAACTAACAAGGATTCCCCTAGTAACGGCGAGTGAAGAGGGAAAAGCTCAAATTTTAAATCTACC-TGG---TTC--CCAGGTCGAATTGTATTTTGAAGAATCGATATC-GG-TG-TTGAGGTCTGGTTCAAGTTCTTTGGAACAAGACATCAT-GG-AGGGTGAGAATCCCGTGCATGATCAGA-CC---AAGAT--AC-TTTAT---ATTCGTTTTCTAAGAGTCGAGTTGTTTGGGAATGCAGCTCAAATTGGG-T-GGTAGAC-TTCACCTAAGGCTAAATATCAGCGAGAGACCGATAGCGAACAAGTACTGTG-AA-GGAAAGATGAAAAGCACTTTGAAAAGAGAGTTAAATAGTACGTGAAATTGTTGAAAGGGAAACGATTGAAGTCAGTCATGCTAG-TGAAAATTCAGTTTGACGGG-TTTTG-GGTTTAGGAGT-AAGAGATAGGGT---CAA---ACCATCTCTCTTTTAAACTTGGGATTTGTCGGATGCACTTTTTCT-TTGGCAGGTCAGCGTCGATTTC-GGGGGTTGTAAA---------ATACTTGGGGG-AAAGTAGCTCTGCTTCGGGA---GAGTG-TTATAGACCTTGGGAGATGCAGCCCGTGGGATCGAGGATTGCAGCAAATGC--------------------CTTT--GGC-TTGTCTTCTGATCTTTGA-TTGTTACCTCGTTTATGACAACATT-CTTGCCATCGATGAGTACTAGTGGTTA-TGGGTTAGAATGATCAAAAA--TTTGCTAAGGATGCTGACGTAATGGCTTTAAACGACCCGTCTTGAAACACGGACCAAGGAGTCTAACATATGTGCGAGcggattggatgattataactatacttcctgttcctcctccacctgtacgtcccagtatacaaatggatggaacaagtcgaggtgaagatgatttgactcataaattgtccgatatcttgaaggcaaatcaaaacgtaaaacgttacgaagctgatggtcatcctccacatgttgtaaacgaatttgaagcattgttacaggttcttaataat---------------------------------------------------------------------------------------------------------tataa-tttca--------a-ttattttaatatcaa--ttgaaa--tttatacttaaa-tttattatttac-----atgcaaacagtttcattgtgcaacttatatggacaatgaaatggctggtcaacctcaagctcttcagaaatctggtagacctttaaagtcaatacgtgcgcgtctcaagggtaaagaaggacgtttacgcggtaatctgatgggaaagcgtgtagatttctctgctcgtacagtaattacgggtgatccaaatatttcagttgatgaagtcggagttccgaaaagcatagctcaaaatttgacttttccagaattggtgactccctttaacattgattatcttcaaaaattagtagaaaatggcccttctacacatccaggagctaaatacgtaattagagatactggtgaaaggattgatctaaaacatatttcaggtatgactggtggtttaagattacattacggttggaaagttgaacgtcatctcaatgatggtgacatcgttatattcaatcgtcagccatctttg---------------------------------------------------------------------------------------------------------------------------------------------------------------------------------------------------------------------------------------------------------------------------------------------------------

>Diversispora_epigaea_FM876817

AGGAATCCCTAGTAAGCATGAGTCATCAGCTCATG-TTGATTACGTCCCTGCCCTTTGTACACACCGCCCGTCGCTACTACCGATTGAATGGCTTAGTGAGACCTTTGGATTGGGGTTTAAGAATCGGCAAC---GAATTTTATTCTCCGAGAAGTCGGTCAAACTTGGTCATTTAGAGGAAGTAAAAGTCGTAACAAGGTTTCCGTAGGTGAACCTGCGGAAGGATCATT--AAAAAC-TTTTA-TCCGGGAATTCG-----TTTCGT----T--------TTCCCG-G------ATTATTTGTATTCAAA-TCCCACTCTTT-------AT-AAATAATA---------TCAATTATATAAACA--AATA-T-AAAAAAAGAAAACTTTCAACAACGGATCTCTTGGCTCTCGCATCGATGAAGAACGCAGCGAAATGCGATACGTAATGTGAATTGCAGAATTCCGTGAATCATCGAA-TCTTTGAACACAAATTGTACTTTTCAGTAATCTGGAAAGTATGCTTGGTTGAGGGTCATTAAAATAACA-TTCGTGAA--------------TTTTTTCG-------------CGGATTTGAG----TTTT-CC--AGTA--TTC---AT---TAT-----AA--------AA-TAAATGTTGGTAACTTT-AAAATT-ATTT------ATAACTTGGTACAAGTT-GAAAACGTGC-TATA--TGTGT-GGTTCGCT-GACAACTTGTCCA--T-C--TT----T-ATAT-ATTATGCG-CGTGCTTGG-TTTTTTT----AAAGCTATGTGC--GAGT--ATATA------TTTTTTTTATGAC-CTCAGCTCAAGCAAGAAAACCC-GCTGAACTTAAGCATATCAATAAGCGGAGGAAAAGAAACTAACAAGGATTCCCCTAGTAACGGCGAGTGAAGAGGGAAAAGCTCAAATTTTAAATCTACC-TGG---TTC--CCAGGTCGAATTGTATTTTGAAGAATCGATATC-GG-TG-TTGAGGTCTGGTTCAAGTTCTTTGGAACAAGACATCAT-GG-AGGGTGAGAATCCCGTGCATGATCAGA-CC---AAGAT--AC-TTAAT---ATTCGTTTTCTAAGAGTCGAGTTGTTTGGGAATGCAGCTCAAATTGGG-T-GGTAGAC-TTCACCTAAGGCTAAATATCAGCGAGAGACCGATAGCGAACAAGTACTGTG-AA-GGAAAGATGAAAAGCACTTTGAAAAGAGAGTTAAATAGTACGTGAAATTGTTGAAAGGGAAACGATTGAAGTCAGTCATGCTAG-TGAAAATTCAGTTTGACGGG-TTTTG-GGTTTAGGAGT-AAGAGATAGGGT---CAA---ACCGTCTCTCTTTTAAACTTGGGATTTGTCGGATGCACTTTTTCT-TTGGCAGGTCAGCGTCGATTTC-GGGGGTTGTAAA---------ATACTTGGGGG-AAAGTAGCTCTGCTTCGGGA---GAGTG-TTATAGACCTTGGGAGATGCAGCCCGTGGGATCGAGGATTGCAGCAAATGC--------------------CTTT--GGC-TTGTCTTCTGATCTTTGA-TTGTTACCTCGTTTATGACAACATT-CTTGCCATCGATGAGTACTAGTGGTTA-TGGGTTAGAATGATCAAAAA--TTTGCTAAGGATGCTGACGTAATGGCTTTAAACGACCCGTCTTGAAACACGGACCAAGGAGTCTAACATATGTGCGAG----------------------------------------------------------------------------------------------------------------------------------------------------------------------------------------------------------------------------------------------------------------------------------------------------------------------------------------------------------------------------------------------------------------------------------------------------------------------------------------------------------------------------------------------------------------------------------------------------------------------------------------------------------------------------------------------------------------------------------------------------------------------------------------------------------------------------------------------------------------------------------------------------------------------------------------------------------------------------------------------------------------------------------------------------------------------------------------------------------------------------------------------------------------------------------------------------------

>Diversispora_epigaea_FM876819

AGGAATCCCTAGTAAGCATGAGTCATCAGCTCATG-TTGATTACGTCCCTGCCCTTTGTACACACCGCCCGTCGCTACTACCGATTGAATGGCTTAGTGAGACCTTTGGATTGGGGTTTAAGAATCGGCAAC---GAATTTTATTCTCCGAGAAGTCGGTCAAACTTGGTCATTTAGAGGAAGTAAAAGTCGTAACAAGGTTTCCGTAGGTGAACCTGCGGAAGGATCATT--AAAAAC-TTTTA-TCCGGGAATTCG-----TTTCGT----T--------TTTTCG-G------ATTATTTGTATTCAAA-TCCCACTCTTT-------AT-AAAT-ATA---------TCAATTATATAAAAC--AATA-TAAAAAAAAGAAAACTTTCAACAACGGATCTCTTGGCTCTCGCATCGATGAAGAACGCAGCGAAATGCGATACGTAATGTGAATTGCAGAATTCCGTGAATCATCGAA-TCTTTGAACACAAATTGTACTTTTCAGTAATCTGGAAAGTATGCTTGGTTGAGGGTCATTAAAATAACA-TTCGTGAA--------------TTTTTTCG-------------CGGATTTGAG----TTTT-CC--AGTA--TTC---AT---TAT------------------AAAATGTTGGTAACTTT-AAAATT-ATTT------ATAACTTGGTACAAGTT-GAAAACGTGC-TATA--TTTGT-GGTTCGCT-GACAACTTGTCCA--T-C--TT----T-ATAT-ATTATGCG-CGTGCTTGG--CTTTTT----AAAGTTCTGTGC--GAGT--ATATA------TTTTTTTTATGAC-CTCAGCTCAAGCAAGAAAACCC-GCTGAACTTAAGCATATCAATAAGCGGAGGAAAAGAAACTAACAAGGATTCCCCTAGTAACGGCGAGTGAAGAGGGAAAAGCTCAAATTTTAAATCTACC-TGG---TTC--CCAGGTCGAATTGTATTTTGAAGAATCGATATT-TG-TG-TTGAGGTCTGGTTCAAGTTCTTTGGAACAAGACATCAT-GG-AGGGTGAGAATCCCGTGCATGATCAGA-CC---AAGAT--AC-TTTAT---ATTCGTTTTCTAAGAGTCGAGTTGTTTGGGAATGCAGCTCAAATTGGG-T-GGTAGAC-TTCACCTAAGGCTAAATATCAGCGAGAGACCGATAGCGAACAAGTACTGTG-AA-GGAAAGATGAAAAGCACTTTGAAAAGAGAGTTAAATAGTACGTGAAATTGTTGAAAGGGAAACGATTGAAGTCAGTCATGCTAG-TGAAAATTCAGTTTGACGGG-TTTTG-GGTTTAGGAGT-AAGAGGTAGGGT---CAA---ACCGTCTCTCTTTTAAACTTGGGATTTGTCGGATGCACTTTTTCT-TTGGCAGGTCAGTGTCGATTTC-GGGGGTTGTAAA---------ATACTTGGGGG-AAAGTAGCTCTGCTTCGGGA---GAGTG-TTATAGACCTTGGGAGATACAGCCTGTGGGATCGAGGATTGCAGCAAATGC--------------------CTTT--GGC-TTGTCGCCTGATCTTTGG-TTGTTACCTCGTTTATGACAACATT-CTTGCCATCGATGAGTACTAATGATCA-TGGGTTAGAACGATCAAAAA--TTTGCTAAGGATGCTGACGTAATGGCTTTAAACGACCCGTCTTGAAACACGGACCAAGGAGTCTAACATATGTGCGAG----------------------------------------------------------------------------------------------------------------------------------------------------------------------------------------------------------------------------------------------------------------------------------------------------------------------------------------------------------------------------------------------------------------------------------------------------------------------------------------------------------------------------------------------------------------------------------------------------------------------------------------------------------------------------------------------------------------------------------------------------------------------------------------------------------------------------------------------------------------------------------------------------------------------------------------------------------------------------------------------------------------------------------------------------------------------------------------------------------------------------------------------------------------------------------------------------------

>Diversispora_epigaea_FM876818

AGGAATCCCTAGTAAGCATGAGTCATCAGCTCATG-TTGATTACGTCCCTGCCCTTTGTACACACCGCCCGTCGCTACTACCGATTGAATGGCTTAGTGAGACCTTTGGATTGGGGTTTAAGAATCGGCAAC---GAATTTTATTCTCCGAGAAGTCGGTCAAACTTGGTCATTTAGAGGAAGTAAAAGTCGTAACAAGGTTTCCGTAGGTGAACCTGCGGAAGGATCATT--AAAAAC-TTTTA-TCCGGGAATTCG-----TTTCGT----T--------TTCCCG-G------ATTATTTGTATTCAAA-TCCCACTCTTT-------AT-AAATAATA---------TCAATTATATAAAAC--AAAT-A-TAAAAAAGAAAACTTTCAACAACGGATCTCTTGGCTCTCGCATCGATGAAGAACGCAGCGAAATGCGATACGTAATGTGAATTGCAGAATTCCGTGAATCATCGAA-TCTTTGAACACAAATTGTACTTTTCAGTAATCTGGGAAGTATGCTTGGTTGAGGGTCATTAAAATAACA-TTCGTGAA--------------TTTTTTCG-------------CGGATTTGAG----TTTT-CC--AGTA--TTC---AT---TAT------A--------AA-TAAATGTTGGTAACTTT-AAAATT-ATTT------ATAACTTGGTACAAGTT-GAAAACGTGC-TATA--TGTGT-GGTTCGCT-GACAACTTGTCCA--T-C--TT----T-ATAT-ATTATGCG-CGTGCTTGG---CTTTT----ATAGTTTTGTGC--GAGT--ATATA-----TTTTTTTTTATGAC-CTCAGCTCAAGCAAGAAAACCC-GCTGAACTTAAGCATATCAATAAGCGGAGGAAAAGAAACTAACAAGGATTCCCCTAGTAACGGCGAGTGAAGAGGGAAAAGCTCAAATTTTAAATCTACC-TGG---TTC--CCAGGTCGAATTGTATTTTGAAGAATCGATATT-TG-TG-TTGAGGTCTGGTTCAAGTTCTTTGGAACAAGACATCAT-GG-AGGGTGAGAATCCCGTGCATGATCAGA-CC---AAGAT--AC-TTTAT---ATTCGTTTTCTAAGAGTCGAGTTGTTTGGGAATGCAGCTCAAATTGGG-T-GGTAGAC-TTCACCTAAGGCTAAATATCAGCGAGAGACCGATAGCGAACAAGTACTGTG-AA-GGAAAGATGAAAAGCACTTTGAAAAGAGAGTTAAATAGTACGTGAAATTGTTGAAAGGGAAACGATTGAAGTCAGTCATGCTAG-TGAAAATTCAGTTTGACGGG-TTTTG-GGTTTGGGAGT-AAGAGGTAGGGT---CAA---ACCGTCTCTCTTTTAAACTTGGGATTTGTCGGATGCACTTTTTCT-TTGGCAGGTCAGCGTCGATTTC-AGAGGCTGTAAA---------ATACTTGGGGG-AAAGTAGCTCTGCTTCGGGA---GAGTG-TTATAGATCCTGGGGGATGCAACCTGTGGGATCGAGGATTGCAGCAAATGC--------------------CTTT--GGC-TTGTCGCCTGATCTCTGG-TTGTTACCTCATTTATGACAACATT-CTTGTCATCGATGAGTGCTAATGGCTATTAGGTTAGAACGATCAAAAA--TTTGCTAAGGATGCTGGCGTAATGGCTTTAAACGACCCGTCTTGAAACACGGACCAAGGAGTCTAACATATGTGCGAG----------------------------------------------------------------------------------------------------------------------------------------------------------------------------------------------------------------------------------------------------------------------------------------------------------------------------------------------------------------------------------------------------------------------------------------------------------------------------------------------------------------------------------------------------------------------------------------------------------------------------------------------------------------------------------------------------------------------------------------------------------------------------------------------------------------------------------------------------------------------------------------------------------------------------------------------------------------------------------------------------------------------------------------------------------------------------------------------------------------------------------------------------------------------------------------------------------

>Diversispora_sporocarpia_MK036785_MK036774

AGGAATCCCTAGTAAGCATGAGTCATCAGCTCATG-TTGATTACGTCCCTGCCCTTTGTACACACCGCCCGTCGCTACTACCGATTGAATGGCTTAGTGAGACCTTTGGATT-GGGTTTAGGGATCGGCAAC---TTTCCTTATT-TTCGAGAAGTCGGTCAAACTTGGTCATTTAGAGGAAGTAAAAGTCGTAACAAGGTTTCCGTAGGTGAACCTGCGGAAGGATCATT--AAAAAC-TTTTA-TCCGGGAATTCG-----TTTCGT----T--------TTTCCG-G------ATTATTTGTATTCAAA-TCCCACTCTTT-------AT-AAAT-ATA---------TTAATTATATAAAACAAAAAA-T-AAAAAAAGAAAACTTTCAACAACGGATCTCTTGGCTCTCGCATCGATGAAGAACGCAGCGAAATGCGATACGTAATGTGAATTGCAGAATTCCGTGAATCATCGAA-TCTTTGAACACAAATTGTACTTTTCAGTAATCTGGAAAGTATGCTTGGTTGAGGGTCATTAAAATAACA-TTCGTGAA---------------TTTTTCG-------------CGGATTTGAG----TTTT-CC--AGTA--TTC---AT---TAT------A--------AA-TAAATGTTGGTAACTTT-AAAATT-ATTT------ATTACTTGGTACAAGTT-GAAAACGTAC-TATA--TGTGT-GGTTCGCT-GACAACTTGTCCA--T-C--T-----T-ATAT-ATTATGCG-CGTGCTTGG--CTTTTT----AAAGTTTTGTGC--GAGT--ATATA------TTTTTTTTATGAC-CTCAGCTCAAGCAAGAATACCC-GCTGAACTTAAGCATATCAATAAGCGGAGGAAAAGAAACTAACAAGGATTCCCCTAGTAACGGCGAGTGAAGAGGGAAAAGCTCAAATTTTAAATCTACC-TGG---TTC--CCAGGTCGAATTGTATTTTGAAGAAGCGATATC-TG-TG-TTGAGGTCTGGTTCAAGTTCTTTGGAACAAGACATCAT-GG-AGGGTGAGAATCCCGTGCATGATCAGA-CC---AAGAT--AC-TTAAT---ATTCGTTTTCTAAGAGTCGAGTTGTTTGGGAATGCAGCTCAAATTGGG-T-GGTAGAC-TTCACCTAAGGCTAAATATCAGCGAGAGACCGATAGCGAACAAGTACTGTG-AA-GGAAAGATGAAAAGCACTTTGAAAAGAGAGTTAAATAGTACGTGAAATTGTTGAAAGGGAAACGATTGAAGTCAGTCATGCTAG-TGAAAATTCAGTTTGACGGG-TTTTG-GGTTTAGGAGT-AAGAGGTAGGGT---CAA---ACCGTCTCTCTTTTAAACTTGGAATTTGTCGGATGCACTTTTTCT-TTGGCAGGTCAGCGTCGATTTC-GGGGGTTGTAAA---------ATACATGGGGA-AAAGTAGCTCTGCTTCGGGA---GAGTG-TTATAGACCCTAGGAGATGCAGCCTGCGGGATCGAGGATTGCAGCAAATGC--------------------CTTT--GGC-TTGTCGCCTGATCTCTGG-ACGTTACCTCGTTTGTGACAACATT-CTTATCACCGATGAGTACTAATGGCTATTGGGTTAGAGCGATCAAAAA--TTTGCTAAGGATGCTGGCGTAATGGCTTTAAACGACCCGTCTTGAAACACGGACCAAGGAGTCTAACATATGTGCGAG----------------------------------------------------------------------------------------------gacacataaattgtccgacatcttgaaggcaaatcaaaacgtaaaacgttacgaagctgatggtcatcccccacacgttgtaaacgaatttgaagcattgttacaggttcttaataat---------------------------------------------------------------------------------------------------------tataa-tttca--------a-ttagtttaatatcaa--ttgaaa--tttatacttaaa-tttattatttac-----atgcaaacagtttcattgtgcaacttatatggacaatgaaatggctggtcaacctcaagctcttcagaaatctggtagacctttaaagtcaatacgtgcgcgtctcaagggtaaagaaggacgtttacgcggtaatctgatgggaaagcgtgtagatttctctgctcgtacagtaattacgggtgatccgaatatttcagttgatgaagtcggagttccgaaaagcatagctcaaaatttgacttttccagaattggtgactccctttaatatcgattatcttcaaaaattagtagaaaatggcccttctacacatccaggagctaaatacgtaattagagatactggtgaaaggattgatctaaaacatatttcaggcatgactggtggtttaagattacattacggttggaaagttgaacgtcatctcaatgatggtgacatc------------------------------------------------------------------------------------------------------------------------------------------------------------------------------------------------------------------------------------------------------------------------------------------------------------------------------------

>Diversispora_sporocarpia_MK036786

AGGAATCCCTAGTAAGCATGAGTCATCAGCTCATG-TTGATTACGTCCCTGCCCTTTGTACACACCGCCCGTCGCTACTACCGATTGAATGGCTTAGTGAGACCTTTGGATT-GGGTTTAGGGATCGGCAAC---TTTCCTTATT-TTCGAGAAGTCGGTCAAACTTGGTCATTTAGAGGAAGTAAAAGTCGTAACAAGGTTTCCGTAGGTGAACCTGCGGAAGGATCATT--AAAAAC-TTTTA-TCCGGGAATTCG-----TTTCGT----C--------TTTCCG-G------ATTATTTGTATTCAAA-TCCCACTCTTT-------AT-AAAT-ATA---------TTAATTATATAAAAC--AAAA-T-AAAAAAAGAAAACTTTCAACAACGGATCTCTTGGCTCTCGCATCGATGAAGAACGCAGCGAAATGCGATACGTAATGTGAATTGCAAAATTCCGTGAATCATCGAA-TCTTTGAACACAAATTGTACTTTTCAGTAATCTGGGAAGTATGCTTGGTTGAGGGTCATTAAAATAACA-TTCGTGAA--------------TTTTTTCG-------------CGGATTTGAG----TTTT-CC--AGTA--TTT---AT---TAT------A--------AA-TAAATGTTGGTAACTTT-AAAATT-ATTT------ATTACTTGGTACAAGTT-GAAAACGTAC-TATATGTGTGT-GGTTCGCT-GACAACTTGTCCA--T-C--TT---AT-ATAT-ATTATGCG-CGTGCTTGG--CTTTTT----AAAGTTTTGTGC--GAGT--ATATA------TTTTTTTTATGAC-CTCAGCTCAAGCAAGAATACCC-GCTGAACTTAAGCATATCAATAAGCGGAGGAAAAGAAACTAACAAGGATTCCCCTAGTAACGGCGAGTGAAGAGGGAAAAGCTCAAATTTTAAATCTACC-TGG---TTC--CCAGGTCGAATTGTATTTTGAAGAAGCGATATT-TG-TG-TTGAGGTCTGGTTCAAGTTCTTTGGAACAAGACATCAT-GG-AGGGTGAGAATCCCGTGCATGATCAGA-CC---AAGAT--AC-TTTAT---ATTCGTTTTCTAAGAGTCGAGTTGTTTGGGAATGCAGCTCAAATTGGG-T-GGTAGAC-TTCACCTAAGGCTAAATATCAGCGAGAGACCGATAGCGAACAAGTACTGTGAAA-GGAAAGATGAAAAGCACTTTGAAAAGAGAGTTAAATAGTACGTGAAATTGTTGAAAGGGAAACGATTGAAGTCAGTCATGCTAG-TGAAAATTCAGTTTGACGGG-TTTTG-GGTTTGGGAGT-AAGAGGTAGGGT---CAA---ACCGTCTCTCTTTTAAACTTGGAATTTGTCGGATGCACTTTTTCT-TTGGCAGGTCAGCGTCGATTTC-GGGGGTTGTAAA---------ATACATGGGGA-AAAGTAGCTCTGCTTCGGGA---GAGTG-TTATAGACCCTAGGAGATGCAGCCTGCGGGATCGAGGATTGCAGCAAATGC--------------------CTTT--GGC-TTGTCGCCTGATCTCTGG-ACGTTACCTCGTTTGTGACAACATT-CTTATCACCGATGAGTACTAATGGCTATTGGGTTAGAGCGATCAAAAA--TTTGCTAAGGATGCTGGCGTAATGGCTTTAAACGACCCGTCTTGAAACACGGACCAAGGAGTCTAACATATGTGCGAG----------------------------------------------------------------------------------------------------------------------------------------------------------------------------------------------------------------------------------------------------------------------------------------------------------------------------------------------------------------------------------------------------------------------------------------------------------------------------------------------------------------------------------------------------------------------------------------------------------------------------------------------------------------------------------------------------------------------------------------------------------------------------------------------------------------------------------------------------------------------------------------------------------------------------------------------------------------------------------------------------------------------------------------------------------------------------------------------------------------------------------------------------------------------------------------------------------

>Diversispora_sporocarpia_MK036788

AGGAATCCCTAGTAAGCATGAGTCATCAGCTCATG-TTGATTACGTCCCTGCCCTTTGTACACACCGCCCGTCGCTACTACCGATTGAATGGCTTAGTGAGACCTTTGGATT-GGGTTTAGGGATCGGCAAC---TTTCCTTATT-TTCGAGAAGTCGGTCAAACTTGGTCATTTAGAGGAAGTAAAAGTCGTAACAAGGTTTCCGTAGGTGAACCTGCGGAAGGATCATT--AAAAAC-TTTTA-TCCGGGAATTCG-----TTTCGT----T--------TTTCCG-G------ATTATTTGTATTCAAA-TCCCACTCTTT-------AT-AAAT-ATA---------TTAATTATATAAAACAAAAAA-T-AAAAAAAGAAAACTTTCAACAACGGATCTCTTGGCTCTCGCATCGATGAAGAACGCAGCGAAATGCGATACGTAATGTGAATTGCAGAATTCCGTGAATCATCGAA-TCTTTGAACACAAATTGTACTTTTCAGTAATCTGGAAAGTATGCTTGGTTGAGGGTCATTAAAATAACA-TTCGTGAA--------------TTTTTTCG-------------CGGATTTGAG----TTTT-CC--AGTA--TTC---AT---TAT------A--------AA-TAAATGTTGGTAACTTT-AAAATT-ATTT------ATTACTTGGTACAAGTT-GAAAACGTAC-TATA--TTTGT-GGTTCGCT-GACAACTTGTCCA--T-C--T-----T-ATAT-ATTATGCG-CGTGCTTGG--CTTTTT----AAAGTTTTGTGC--GAGT--ATATA------TTTTTTTTATGAC-CTCAGCTCAAGCAAGAATACCC-GCTGAACTTAAGCATATCAATAAGCGGAGGAAAAGAAACTAACAAGGATTCCCCTAGTAACGGCGAGTGAAGAGGGAAAAGCTCAAATTTTAAATCTACC-TGG---TTC--CCAGGTCGAATTGTATTTTGAAGAAGCGATATC-TG-TG-TTAAGGTCTGGTTCAAGTTCTTTGGAACAAGACATCAT-GG-AGGGTGAGAATCCCGTGCATGATCAGA-CC---AAGAT--AC-TTAAT---ATTCGTTTTCTAAGAGTCGAGTTGTTTGGGAATGCAGCTCAAATTGGGTT-GGTAGAC-TTCACCTAAGGCTAAATATCAGCGAGAGACCGATAGCGAACAAGTACTGTG-AAGGGAAAGATGAAAAGCACTTTGAAAAGAGAGTTAAATAGTACGTGAAATTGTTGAAAGGGAAACGATTGAAGTCAGTCATGCTAG-TGAAAATTCAGTTTGACGGG-TTTTA-GGTTTAGGAGT-AAGAGGTAGGGT---CAA---ACCGTCTCTCTTTTAAACTTGGAATTTGTCGGATGCACTTTTTCT-TTGGCAGGTCAGCGTCGATTTC-GGGGGTTGTAAA---------ATACATGGGGA-AAAGTAGCTCTGCTTCGGGA---GAGTG-TTATAGACCCTAGGAGATGCAGCCTGCGGGATCGAGGATTGCAGCAAATGC--------------------TTT----GC-TTGTCGCCTGATTTCTGG-TTGTTACCTCGTTTATGACAACATT-CTTGTCATCGATGAGTACTAATGGCTATTAGGTTAGAGCGATCTAAAA--TTTGCTAAGGATGTTGGCGTAATGGCTTTAAACGACCCGTCTTGAAACACGGACCAAGGAGTCTAACATATATGCGAG----------------------------------------------------------------------------------------------------------------------------------------------------------------------------------------------------------------------------------------------------------------------------------------------------------------------------------------------------------------------------------------------------------------------------------------------------------------------------------------------------------------------------------------------------------------------------------------------------------------------------------------------------------------------------------------------------------------------------------------------------------------------------------------------------------------------------------------------------------------------------------------------------------------------------------------------------------------------------------------------------------------------------------------------------------------------------------------------------------------------------------------------------------------------------------------------------------

>Diversispora_sporocarpia_MK036789

AGGAATCCCTAGTAAGCATGAGTCATCAGCTCATG-TTGATTACGTCCCTGCCCTTTGTACACACCGCCCGTCGCTACTACCGATTGAATGGCTTAGTGAGACCTTTGGATT-GGGTTTAGGGATCGGCAAC---TTTCCTTATT-TTCGAGAAGTCGGTCAAACTTGGTCATTTAGAGGAAGTAAAAGTCGTAACAAGGTTTCCGTAGGTGAACCTGCGGAAGGATCATT--AAAAAC-TTTTA-TCCGGGAATTCG-----TTTCGT----T--------TTTCCG-G------ATTATTTGTATTCAAA-TCCCACTCTTT-------AT-AAAT-ATA---------TTAATTATATAAAAC-AAAAA-T-AAAAAAAGAAAACTTTCAACAACGGATCTCTTGGCTCTCGCATCGATGAAGAACGCAGCGAAATGCGATACGTAATGTGAATTGCAGAATTCCGTGAATCATCGAA-TCTTTGAACACAAATTGTACTTTTCAGTAATCTGGAAAGTATGCTTGGTTGAGGGTCATTAAAATAACA-TTCGTGAA---------------TTTTTCG-------------CGGATTTGAG----TTTTCCC--AGTA--TTC---AT---TAT------A--------AA-TAAATGTTGGTAACTTT-AAAATT-ATTT------ATTACTTGGTACAAGTT-GAAAACGTAC-TATA--TTTGT-GGTTCGCT-GACAACTTGTCCA--T-C--T-----T-ATAT-ATTATGCG-CGTGCTTGG--CTTTTT----AAAGTTTTGTGC--GAGT--ATATA------TTTTTTTTATGAC-CTCAGCTCAAGCAAGAATACCC-GCTGAACTTAAGCATATCAATAAGCGGAGGAAAAGAAACTAACAAGGATTCCCCTAGTAACGGCGAGTGAAGAGGGAAAAGCTCAAATTTTAAATCTACC-TGG---TTC--CCAGGTCGAATTGTATTTTGAAGAAGCGATATC-TG-TG-TTGAGGTCTGGTTCAAGTTCTTTGGAACAAGACATCAT-GGAAGGGTGAGAATCCCGTGCATGATCAGA-CC---AAGAT--AC-TTAAT---ATTCGTTTTCTAAGAGTCGAGTTGTTTGGGAATGCAGCTCAAATTGGGTT-GGTAGACTTTCACCTAAGGCTAAATATCAGCGAGAGACCGATAGCGAACAAGTACTGTG-AA-GGAAAGATGAAAAGCACTTTGAAAAGAGAGTTAAATAGTACGTGAAATTGTTGAAAGGGAAACGATTGAAGTCAGTCATGCTTG-TGAAAATTCAGTTTGACGAG-TTTTG-GGTTTAGGAGT-AAGAGGTAGGGT---CAA---ACCGTCTCTCTTTTAAACTTGGAATTTGTCGGATGCACTTTTTCT-TTGGCAGGTCAGCGTCGGTTTC-GGGGGTTGTAAA---------ATACATGGGGA-AAAGTAGCTCTGCTTCGGGA---GAGTG-TTATAGACCCTAGGAGATGCAGCCTGCGGGATCGAGGATTGCAGCAAATGC--------------------CTTT--GGC-TTGTCGCCTGATTTCTGG-TTGTTACCTCGTTTATGACAACATT-CTTGTCATCGATGAGTACTAATGGCTATTAGGTTAGAGCGATCTAAAA--TTTGCTAAGGATGTTGGCGTAATGGCTTTAAACGACCCGTCTTGAAACACGGACCAAGGAGTCTAACATATGTGCGAG----------------------------------------------------------------------------------------------------------------------------------------------------------------------------------------------------------------------------------------------------------------------------------------------------------------------------------------------------------------------------------------------------------------------------------------------------------------------------------------------------------------------------------------------------------------------------------------------------------------------------------------------------------------------------------------------------------------------------------------------------------------------------------------------------------------------------------------------------------------------------------------------------------------------------------------------------------------------------------------------------------------------------------------------------------------------------------------------------------------------------------------------------------------------------------------------------------

>Diversispora_arenaria_KJ850188_MG459187

AGGAATCCCTAGTAAGCATGAGTCATCAGCTCATG-TTGATTACGTCCCTGCCCTTTGTACACACCGCCCGTCGCTACTACCGATTGAATGGCTTAGTGAGACCTTTGGATTGAGGTTTAGGGATCGGAAAC---GATCCTTATTCCACGAGAAGTCGGTCAAACTTGGTCATTTAGAGGAAGTAAAAGTCGTAACAAGGTTTCCGTAGGTGAACCTGCGGAAGGATCATT-AAAAAAT-ATTTT-TCCGGGAATACG-----TTTCGT----T--------TGCCCG-G------ATTTATTGTATTCAAA-TCCCACTCTTT-------AT-AAAT-ATA---------TCAATTATATAAAAC-AAAAA-A-TAAAAAAGAAAACTTTCAACAACGGATCTCTTGGCTCTCGCATCGATGAAGAACGCAGCGAAATGCGATACGTAGTGTGAATTGCAGAATTCCGTGAATCATCGAA-TCTTTGAACGCAAATTGTACTTTCCAGTAATCTGGGAAGTATGCTTGGTTGAGGGTCATCGAAATAACC-TTCGTGAA--------------TTTTTTTG-------------CGGATTTGAG----TTTT-CC--AGTA--TTA---AT---TAT------AATAAATATGA-TAAATGTTGGTGACTTT-AAAATT-ATTT------ATAACTTGGTACAAGTT-GAAAACGTGC-TATG--TGTGT-GGTTCGCT-GACAACTTGTCCA--T-C--TC----T-ATAT-ACTATGCG-CGCACTTGG----TTTT----TATACTCTGTGC--GAAC--ATATA------TTCTTTTTTTGAC-CTCAGCTCAAGCAAGAATACCC-GCTGAACTTAAGCATATCAATAAGCGGAGGAAAAGAAACTAACAAGGATTCCCCTAGTAACGGCGAGTGAAGAGGGAAAAGCTCAAATTTTAAATCTACC-TGG---TTC--CCAGGTCGAGTTGTAATTTGAAGAAGCGATATC-GG-TG-TGGAGGTCTGGTTCAAGTTCTTTGGAACAAGACATCATGGG-AGGGTGAGAATCCCGTGCATGATCAGA-CC---AAAAT--AC-CTAAT---ATTCGTTTTCTAAGAGTCGAGTTGTTTGGGAATGCAGCTCAAAATGGG-T-GGTAGAC-TTCACCTAAGGCTAAATATCAGCGAGAGACCGATAGCGAACAAGTACTGTG-AA-GGAAAGATGAAAAGAACTCTGAAAAGAGAGTTAAATAGTACGTGAAATTGTTGAAAGGGAAACGATTGAAGTCAGTCATGCTAG-TGAAAATTCAGTTTGGCGGGTTTTTA-AGTTTGGGAGT--AGGAGGCAAGC--------------CTCTCTTTTGAACTTTTAATCTGTCAGATGCACTTTTTCT-TTGGCAGGTCAGCGTCGATTTC-GGAGGCTGTAAA---------ATACTTGGGGG-AAAGTAGCTCTGCTTCGGGA---GAGTG-TTATAGACCCTGGGGGATGCAGCCTGCGGGATCGAGGATTGCAGCAAATGC------------------TTTTTT--GGC-TTGTCGCCTGATCTCTGG-ACGTTACCTCGCTTGTGACAACATT-CTTGCCACTGGTGAGTACTAATGGCTATTAGGTTGGAGCGATCAAAAA--TTTGCTAAGGATGCTGACGTAATGGCTTTAAACGACCCGTCTTGAAACACGGACCAAGGAGTCTAACATGTGTGCGAG-------------------------------------------------------------------------------tggagaagatgatttgactcacaaattgtccgacatcttgaaggcaaatcaaaacgttaaacgttatgaagctgatggtcatcccccacacgttgtaaacgaatttgaagcattattacaggttcttaatgat---------------------------------------------------------------------------------------------------------tataa-tttca--------a-ttagtttaatatcat--ttgaaa--tttatacttaaattttattatttac-----atgcaaacagtttcattgtgcaacttatatggacaatgaaatggctggtcaacctcaagctcttcagaaatctggtagacctttaaagtcaatacgtgcgcgtctcaagggtaaagaaggacgtttacgcggtaatctgatgggaaagcgtgtagatttctctgctcgtacagtaattacgggtgatccaaatatttcagttgatgaagtcggagttccgaaaagcatagctcaaaatttaacatttccagaattggtgactccctttaatattgattatcttcaaaaattagtagaaaatggcccttctacacatccaggggctaaatacgtaattagagatactggtgaaaggattgatctaaaacatatatcaggcatgactggtggcttaagattacactacggttggaaagttgaacgtcatctcaatgatggtgacatcgttatattcaatcgtcagccatctttgcacaaaatgtcaatgatggg-------------------------------------------------------------------------------------------------------------------------------------------------------------------------------------------------------------------------------------------------------------------------------------

>Diversispora_arenaria_KJ850189

AGGAATCCCTAGTAAGCATGAGTCATCAGCTCATG-TTGATTACGTCCCTGCCCTTTGTACACACCGCCCGTCGCTACTACCGATTGAATGGCTTAGTGAGACCTTTGGATTGAGGTTTAGGGATCGGAAAC---GATCCTTATTCCACGAGAAGTCGGTCAAACTTGGTCATTTAGAGGAAGTAAAAGTCGTAACAAGGTTTCCGTAGGTGAACCTGCGGAAGGATCATT-AAAAAAT-ATTTT-TCCGGGAATACG-----TTTCGT----T--------TGCCCG-G------ATTTATTGTATTCAAA-TCCCACTCTTT-------AT-AAAT-ATA---------TCAATTATATAAAAC-AAAAA-A-TAAAAAAGAAAACTTTCAACAACGGATCTCTTGGCTCTCGCATCGATGAAGAACGCAGCGAAATGCGATACGTAGTGTGAATTGCAGAATTCCGTGAATCATCGAA-TCTTTGAACGCAAATTGTACTTTCCAGTAATCTGGGGAGTATGCTTGGTTGAGGGTCATCAAAATAACA-TTCGTGAA--------------TTTTTTTG-------------CGGATTTGAG----TTTT-CC--AGTA--TTA---AT---TAT------AATAAATATGA-TAAATGTTGGTGACTTT-AAAATT-ATTT------ATAACTTGGTACAAGTT-GAAAACGTGCTTATA--TGTGT-GGTTCGCT-GACAACTTGTCCA--T-C--TC----T-ATAT-ACTATGCG-CGCACTTGG----TTTT----TATACTCTGTGC--GAAC--ATATA-----TTTTTTTTTTTGAC-CTCAGCTCAAGCAAGAATACCC-GCTGAACTTAAGCATATCAATAAGCGGAGGAAAAGAAACTAACAAGGATTCCCCTAGTAACGGCGAGTGAAGAGGGAAAAGCTCAAATTTTAAATCTACC-TGG---TTC--CCAGGTCGAGTTGTAATTTGAAGAAGCGATATC-GG-TG-TGGAGGTCTGGTTCAAGTTCTTTGGAACAAGACATCATGGG-AGGGTGAGAATCCCGTGCATGATCAGA-CC---AAAAT--AC-CTAAT---ATTCGTTTTCTAAGAGTCGAGTTGTTTGGGAATGCAGCTCAAAATGGG-T-GGTAGAC-TTCACCTAAGGCTAAATATCAGCGAGAGACCGATAGCGAACAAGTACTGTG-AA-GGAAAGATGAAAAGAACTTTGAAAAGAGAGTTAAATAGTACGTGAAATTGTTGAAAGGGAAACGATTGAAGTCAGTCATGCTAG-TGAAAATTCAGTTTGGCGGGTTTTTA-AGTTTGGGAGT--AGGAGGCAAGC--------------CTCTCTTTTGAACTTTTAATCTGTCAGATGCACTTTTTCT-TTGGCAGGTCAGCGTCGATTTC-GGAGGCTGTAAA---------ATACTTGGGGG-AAAGTAGCTCTGCTTCGGGA---GAGTG-TTATAGACCCTGGGGGATGCAGCCTGCGGGATCGAGGATTGCAGCAAATGC-------------------TTTTT--GGC-TTGTCGCCTGATCTCTGG-ACGTTACCTCGCTTGTGACAACATT-CTTGCCACCGGTGAGTACTAATGGCTATTAGGTTAGAGCGATCAAAAA--TTTGCTAAGGATGCTGACGTAATGGCTTTAAACGACCCGTCTTGAAACACGGACCAAGGAGTCTAACATATATGCGAG----------------------------------------------------------------------------------------------------------------------------------------------------------------------------------------------------------------------------------------------------------------------------------------------------------------------------------------------------------------------------------------------------------------------------------------------------------------------------------------------------------------------------------------------------------------------------------------------------------------------------------------------------------------------------------------------------------------------------------------------------------------------------------------------------------------------------------------------------------------------------------------------------------------------------------------------------------------------------------------------------------------------------------------------------------------------------------------------------------------------------------------------------------------------------------------------------------

>Diversispora_arenaria_KJ850187

AGGAATCCCTAGTAAGCATGAGTCATCAGCTCATG-TTGATTACGTCCCTGCCCTTTGTACACACCGCCCGTCGCTACTACCGATTGAATGGCTTAGTGAGACCTTTGGATTGAGGTTTAGGGATCGGAAAC---GATCCTTATTCCACGAGAAGTCGGTCAAACTTGGTCATTTAGAGGAAGTAAAAGTCGTAACAAGGTTTCCGTAGGTGAACCTGCGGAAGGATCATT-AAAAAAT-ATTTT-TCCGGGAATACG-----TTTCGT----T--------TGCCCG-G------ATTTATTGTATTCAAA-TCCCACTCTTT-------AT-AAAT-ATA---------TCAATTATATAAAAC-AAAAA-A-TAAAAAAGAAAACTTTCAACAACGGATCTCTTGGCTCTCGCATCGATGAAGAACGCAGCGAAATGCGATACGTAGTGTGAATTGCAGAATTCCGTGAATCATCGAA-TCTTTGAACGCAAATTGTACTTTCCAGTAATCTGGGAAGTATGCTTGGTTGAGGGTCATCAAAATAACA-TTCGTGAA--------------TTTTTTCG-------------CGGATTTGAG----CTTT-CC--AGTA--TTA---AT---TAT------AATAAATATAA-TAAATGTTGGTGACTTT-AAAATTAATTT------ATAACTTGGTACAAGTT-GAAAACGTGC-TATA--TGTGT-GGTTCGCT-GACAACTTGTCCA--T-C--TC----T-ATAT-ACTATGCG-CGCACTTGG----TTTT----TATACTCTGTGC--GAAC--ATATA-----TTTTTTTTTTTGAC-CTCAGCTCAAGCAAGAATACCC-GCTGAACTTAAGCATATCAATAAGCGGAGGAAAAGAAACTAACAAGGATTCCCCTAGTAACGGCGAGTGAAGAGGGAAAAGCTCAAATTTTAAATCTACC-TGG---TTC--CCAGGTCGAGTTGTAATTTGAAGAAGCGATATCGGG-TG-TGGAGGTCTGGTTCAAGTTCTTTGGAACAAGACATCATGGG-AGGGTGAGAATCCCGTGCATGATCAGA-CC---AAAAT--AC-CTAAT---ATTCGTTTTCTAAGAGTCGAGTTGTTTGGGAATGCAGCTCAAAATGGG-T-GGTAGAC-TTCACCTAAGGCTAAATATCAGCGAGAGACCGATAGCGAACAAGTACTGTG-AA-GGAAAGATGAAAAGAACTTTGAAAAGAGAGTTAAATAGTACGTGAAATTGTTGAAAGGGAAACGATTGAAGTCAGTCATGCTAG-TGAAAATTCAGTTTGGCGGG-TTTTA-AGTTTGGGAGT--AGGAGGCAAGC--------------CTCTCTTTTGAACTTTTAATCTGTCAGATGCACTTTTTCT-TTGGCAGGTCAGCGTCGATTTC-GGAGGCTGTAAA---------ATACTTGGGGG-AAAGTAGCTCTGCTTCGGGA---GAGTG-TTATAGACCCTGGGGGATGCAGCCTGCGGGATCGAGGATTGCAGCAAATGC-------------------TTTTT--GGC-TTGTCGCCTGATCTCTGG-ACGTTACCTCGCTTGTGACAACATT-CTTGGCACCGGTGAGTACTAATGGCTATTAGGTTAGAGCGATCAAAAA-TTTTGCTAAGGATGCTGACGTAATGGCTTTAAACGACCCGTCTTGAAACACGGACCAAGGAGTCTAACATATATGCGAG----------------------------------------------------------------------------------------------------------------------------------------------------------------------------------------------------------------------------------------------------------------------------------------------------------------------------------------------------------------------------------------------------------------------------------------------------------------------------------------------------------------------------------------------------------------------------------------------------------------------------------------------------------------------------------------------------------------------------------------------------------------------------------------------------------------------------------------------------------------------------------------------------------------------------------------------------------------------------------------------------------------------------------------------------------------------------------------------------------------------------------------------------------------------------------------------------------

>Diversispora_arenaria_KJ850186_MG459186

AGGAATCCCTAGTAAGCATGAGTCATCAGCTCATG-TTGATTACGTCCCTGCCCTTTGTACACACCGCCCGTCGCTACTACCGATTGAATGGCTTAGTGAGACCTTTGGATTGAGGTTTAGGGATCGGAAAC---GATCCTTATTCCACGAGAAGTCGGTCAAACTTGGTCATTTAGAGGAAGTAAAAGTCGTAACAAGGTTTCCGTAGGTGAACCTGCGGAAGGATCATT-AAAAAAT-ATTTT-TCCGGGAATACG-----TTTCGT----T--------TGCCCG-G------ATTTATTGTATTCAAA-TCCCACTCTTT-------AT-AAAT-ATA---------TCAATTATAT-AAAC-AAAAA-A-TAAAAAAGAAAACTTTCAACAACGGATCTCTTGGCTCTCGCATCGATGAAGAACGCAGCGAAATGCGATACGTAGTGTGAATTGCAGAATTCCGTGAATCATCGAA-TCTTTGAACGCAAATTGTACTTTCCAGTAATCTGGGAAGTATGCTTGGTTGAGGGTCATCAAAATAACA-TTCGTGAA--------------TTTTTTCG-------------CGGATTTGAG---CTTTC-CC--AGTA--TTA---AT---TAT------AATAAATATGA-TAAATGTTGGTGACTTT-AAAATT-ATTT------ATAACTTGGTACAAGTT-GAAAACGTGC-TATA--TGTGT-GGTTCGCT-GACAACTTGTCCA--T-C--TC----T-ATAT-ACTATGCG-CGCACTTGG----TTTT----TACACTCTGTGT--GAAC--ATATA------TTTTTTTTTTGAC-CTCAGCTCAAGCAAGAATACCC-GCTGAACTTAAGCATATCAATAAGCGGAGGAAAAGAAACTAACAAGGATTCCCCTAGTAACGGCGAGTGAAGAGGGAAAAGCTCAAATTTTAAATCTACC-TGG---TTC--CCAGGTCGAGTTGTAATTTGAAGAAGCGATATCCGG-TG-TGGAGGTCTGGTTCAAGTTCTTTGGAACAAGACATCATGGG-AGGGTGAGAATCCCGTGCATGATCAGA-CC---AAAAT--AC-CTAAT---ATTCGTTTTCTAAGAGTCGAGTTGTTTGGGAATGCAGCTCAAAATGGG-T-GGTAGAC-TTCACCTAAGGCTAAATATCAGCGAGAGACCGATAGCGAACAAGTACTGTG-AA-GGAAAGATGAAAAGAACTTTGAAAAGAGAGTTAAATAGTACGTGAAATTGTTGAAAGGGAAACGATTGAAGTCAGTCATGCTAG-TGAAAATTCAGTTTGGCGGG-TTTTA-AGTTTGGGAGT--AGGAGGCAAGC--------------CTCTCTTTTGAACTTTTAATCTGTCAGATGCACTTTTTCT-TTGGCAGGTCAGCGTCGATTTC-GGAGGTTGTAAA---------ATACTTGGGGG-AAAGTAGCTCTGCTTCGGGA---GAGTG-TTATAGACCCTGGGGGATGCAGCCTGCGGGATCGAGGATTGCAGCAAATGC-------------------TTTTT--GGC-TTGTCGCCTGATCTCTGG-ACGTTACCTCGCTTGTGACAACATT-CTTGCCACCGGTGAGTACTAATGGCTATTAGGTTAGAGCGATCAAAAA--TTTGCTAAGGATGCTGACGTAATGGCTTTAAACGACCCGTCTTGAAACACGGACCAAGGAGTCTAACATGTGTGCGAG----------------------------------------------------------------------------------agaagatgatttgacacacaaattgtccgacatcttgaaggcaaatcaaaacgttaaacgttatgaagctgatggtcatcccccacacgttgtaaacgaatttgaagcattattacaggttcttaatgat---------------------------------------------------------------------------------------------------------tataa-tttca--------a-ttagtttaatatcat--ttgaaa--tttatacttaaattttattatttac-----atgcaaacagtttcattgtgcaacttatatggacaatgaaatggctggtcaacctcaagctcttcagaaatctggtagacctttaaagtcaatacgtgcgcgtctcaagggtaaagaaggacgtttacgcggtaatctgatgggaaagcgtgtagatttctctgctcgtacagtaattacgggtgatccaaatatttcagttgatgaagtcggagttccgaaaagcatagctcaaaatttaacatttccagaattggtgactccctttaatattgattatcttcaaaaattagtagaaaatggcccttctacacatccaggggctaaatacgtaattagagatactggtgaaaggattgatctaaaacatatatcaggcatgactggtggcttaagattacactacggttggaaagttgaacgtcatctcaatgatggtgacatcgttatattcaatcgtcagccatctttgcacaaaatgtcaatgatggg-------------------------------------------------------------------------------------------------------------------------------------------------------------------------------------------------------------------------------------------------------------------------------------

>Diversispora_jakucsiae_KJ850181_MG459191

AGGAATCCCTAGTAAGCATGAGTCATCAGCTCATG-TTGATTACGTCCCTGCCCTTTGTACACACCGCCCGTCGCTACTACCGATTGAATGGCTTAGTGAGACCTTTGGATTGGGGTTTAGGGATCGGAAAC---GATCTTTATTCCACGAGAAGTCGGTCAAACTTGGTCATTTAGAGGAAGTAAAAGTCGTAACAAGGTTTCCGTAGGTGAACCTGCGGAAGGATCATT--AAAAAT-ATTTA-TCCGGGAATACG----TTTTCGT----T--------TTCCCG-G------ATTATTTGTATTCAAA-TCCCACTCTTT-------AT-AAAT-ATA---------TCAATTATATAAAAC-AAAAA-A-TAAAAAAGAAAACTTTCAACAACGGATCTCTTGGCTCTCGCATCGATGAAGAACGCAGCGAAATGCGATACGTAATGTGAATTGCAG-ATTCCGTGAATCATCGAA-TCTTTGAACGCAAATTGTACTTTCCAGTAATCTGGGGAGTATGCTTGGTTGAGGGTCATTAAAATAACA-TTCGTGAA--------------TTTTTTCG-------------CGGATTTGAG----TTTT-CC--ATTA--TTC---AT---TAT------AATAATAATAA-TAATAAATGGTGACTTT-AAAATT-ATTT------ATAACTTGGTACAAGTT-GAAAACGTGC-TATA--TGTGT-GGTTCGCT-GACAACTTGTCCA--T-C--TC----T-ATAT-ACTATGCG-CGCACTTGG----TTTT----TTCACTCTGTGC--GAAC--ATATA------TTTTTTTTATGAC-CTCAGCTCAAGCAAGAATACCC-GCTGAACTTAAGCATATCAATAAGCGGAGGAAAAGAAACTAACAAGGATTCCCCTAGTAACGGCGAGTGAAGAGGGAAAAGCTCAAATTTTAAATCTACC-TGG---TTC--CCAGGTCGAGTTGTAATTTGAAGAAGCGATATC-GG-TG-TGGAGGTCTGGTTCAAGTTCTTTGGAACAAGACATCATGGA-AGGGTGAGAATCCCGTGCATGATCAGA-CC---AAGAT--AC-CTAAT---ATTCGTTTTCTAAGAGTCGAGTTGTTTGGGAATGCAGCTCAAAATGGG-T-GGTAGAC-TTCACCTAAGGCTAAATATCAGCGAGAGACCGATAGCGAACAAGTACTGTG-AA-GGAAAGATGAAAAGAACTTTGAAAAGAGAGTTAAATAGTACGTGAAATTGTTGAAAGGGAAACGATTGAAGTCAGTCATGCTAG-TGAAAATTCAGTTTGTCGGGTTTTTA-AGTTTGGGAGT--AGGAGGCAAGC--------------CTCTCTTTTGAACTTTTAATCTGTCAGATGCACTTTTTCT-TTGGCAGGTCAGCGTCGATTTC-GGAGGTTGTAAA---------ATACTTGGGGG-AAAGTAGCTCTGCCTCGGGA---GAGTG-TTATAGACCCTGGGGGATGCAGCCTGCGGGATCGAGGATTGCAGCAAATGC------------------TTTTTT--GGC-TTGTCGCCTGATCTCTGG-ACGTTACCTCGCTTGTGACAACATT-CTTGCCACCGGTGAGTACTAATGGCTATTAGGTTAGAGCGATCAAAAA-TATTGCTAAGGATGCTGACGTAATGGCTTTAAACGACCCGTCTTGAAACACGGACCAAGGAGTCTAACATATATGCGAG------------------------------------------------------------------------------gtggagaagatgatttgactcacaaattgtccgacatcttgaaggcaaatcaaaacgtaaaacgttatgaagctgatggtcatcccccacacgttgtaaacgaatttgaagcattgttacaggttcttaattat---------------------------------------------------------------------------------------------------------tataa-tttca--------a-ttagtttaatatcaa--ttgaaa--tttatacttaaattttattatttac-----atgcaaacagtttcattgtgcaacttatatggacaatgaaatggctggtcaacctcaagctcttcagaaatctggtagacctttaaagtcaatacgtgcgcgtctcaagggtaaagaaggacgtttacgcggtaatctgatgggaaagcgtgtagatttctctgctcgtacagtaattacgggtgatccaaatatttcagttgatgaagtcggagttccgaaaagcatagctcaaaatttaacatttccagaattggtgactccctttaatattgattatcttcaaaaattagtagaaaatggcccttctacacatccaggggctaaatacgtaattagagatactggtgaaaggattgatctaaaacatatatcaggcatgactggtggcttaagattacactacggttggaaagttgaacgtcatctcaatgatggtgacatcgttatattcaatcgtcagccatctttgcacaaaatgtcaatgatggg-------------------------------------------------------------------------------------------------------------------------------------------------------------------------------------------------------------------------------------------------------------------------------------

>Diversispora_jakucsiae_KJ850182

AGGAATCCCTAGTAAGCATGAGTCATCAGCTCATG-TTGATTACGTCCCTGCCCTTTGTACACACCGCCCGTCGCTACTACCGATTGAATGGCTTAGTGAGACCTTTGGATTGGGGTTTAGGGATCGGAAAC---GATCTTTATTCCACGAGAAGTCGGTCAAACTTGGTCATTTAGAGGAAGTAAAAGTCGTAACAAGGTTTCCGTAGGTGAACCTGCGGAAGGATCATT--AAAAAT-ATTTA-TCCGGGAATACG----TTTTCGT----T--------TTCCCG-G------ATTATTTGTATTCAAA-TCCCACTCTTT-------AT-AAAT-ATA---------TCAATTATATAAAAC-AAAAA-A-TAAAAAAGAAAACTTTCAACAACGGATCTCTTGGCTCTCGCATCGATGAAGAACGCAGCGAAATGCGATACGTAATGTGAATTGCAG-ATTCCGTGAATCATCGAA-TCTTTGAACGCAAATTGTACTTTCCAGTAATCTGGGGAGTATGCTTGGTTGAGGGTCATTAAAATAACA-TTCGTGAA--------------TTTTTTCG-------------CGGATTTGAG----TTTT-CC--ATTA--TTC---AT---TAT------AATAATAATAA-TAATAAATGGTGACTTT-AAAATT-ATTT------ATAACTTGGTACAAGTT-GAAAACGTGC-TATA--TGTGT-GGTTCGCT-GACAACTTGTCCA--T-C--TC----T-ATAT-ACTATGCG-CGCACTTGG----TTTT----TTCACTCTGTGC--GAAC--ATATA------TTTTTTTTATGAC-CTCAGCTCAAGCAAGAATACCC-GCTGAACTTAAGCATATCAATAAGCGGAGGAAAAGAAACTAACAAGGATTCCCCTAGTAACGGCGAGTGAAGAGGGAAAAGCTCAAATTTTAAATCTACC-TGG---TTC--CCAGGTCGAGTTGTAATTTGAAGAAGCGATATCGGG-TG-TGGAGGTCTGGTTCAAGTTCTTTGGAACAAGACATCATGGA-AGGGTGAGAATCCCGTGCATGATCAGA-CC---AAGAT--AC-CTAAT---ATTCGTTTTCTAAGAGTCGAGTTGTTTGGGAATGCAGCTCAAAATGGG-TGGGTAGAC-TTCACCTAAGGCTAAATATCAGCGAGAGACCGATAGCGAACAAGTACTGTG-AA-GGAAAGATGAAAAGAACTTTGAAAAGAGAGTTAAATAGTACGTGAAATTGTTGAAAGGGAAACGATTGAAGTCAGTCATGCTAG-TGAAAATTCAGTTTGTCGGGTTTTTA-AGTTTGGGAGT--AGGAGGCAAGC--------------CTCTCTTTTGAACTTTTAATCTGTCAGATGCACTTTTTCT-TTGGCAGGTCAGCGTCGATTTC-GGAGGTTGTAAA---------ATACTTGGGGG-AAAGTAGCTCTGCCTCGGGA---GAGTG-TTATAGACCCTGGGGGATGCAGCCTGCGGGATCGAGGATTGCAGCAAATGC------------------TTTTTT--GGC-TTGTCGCCTGATCTCTGG-ACGTTACCTCGCTTGTGACAACATT-CTTGCCACCGGTGAGTACTAATGGCTATTAGGTTAGAGCGATCAAAAA-TATTGCTAAGGATGCTGACGTAATGGCTTTAAACGACCCGTCTTGAAACACGGACCAAGGAGTCTAACATATATGCGAG----------------------------------------------------------------------------------------------------------------------------------------------------------------------------------------------------------------------------------------------------------------------------------------------------------------------------------------------------------------------------------------------------------------------------------------------------------------------------------------------------------------------------------------------------------------------------------------------------------------------------------------------------------------------------------------------------------------------------------------------------------------------------------------------------------------------------------------------------------------------------------------------------------------------------------------------------------------------------------------------------------------------------------------------------------------------------------------------------------------------------------------------------------------------------------------------------------

>Diversispora_jakucsiae_KJ850183

AGGAATCCCTAGTAAGCATGAGTCATCAGCTCATG-TTGATTACGTCCCTGCCCTTTGTACACACCGCCCGTCGCTACTACCGATTGAATGGCTTAGTGAGACCTTTGGATTGGGGTTTAGGGATCGGAAAC---GATCTTTATTCCACGAGAAGTCGGTCAAACTTGGTCATTTAGAGGAAGTAAAAGTCGTAACAAGGTTTCCGTAGGTGAACCTGCGGAAGGATCATT--AAAAAT-ATTTA-TCCGGGAATACG----TTTCCGT----T--------TTCCCG-G------ATTATTTGTATTCAAA-TCCCACTCTTT-------AT-AAAT-ATA---------TCAATTATATAAAAC-AAAAA-A-TAAAAAAGAAAACTTTCAACAACGGATCTCTTGGCTCTCGCATCGATGAAGAACGCAGCGAAATGCGATACGTAATGTGAATTGCAG-ATTCCGTGAATCATCGAA-TCTTTGAACGCAAATTGTACTTTCCAGTAATCTGGGGAGTATGCTTGGTTGAGGGTCATTAAAATAACA-TTCGTGAA---------------TTTTTCG-------------CGGATTTGAG----TTTT-CC--AGTA--TTC---AT---TAT------AAT------GA-TAAATGTTGGTGACTTT-AAAATT-ATTT------ATAACTTGATACAAGTT-GAAAACGTGC-TATA--TGTGT-GGTTCGCT-GACAACTTGTCCA--T-C--TC----T-ATAT-ACTATGCG-CGCACTTGG----TTTT----TTCACTCTGTGC--GAAC--ATATA------TTTTTTTTATGAC-CTCAGCTCAAGCAAGAATACCC-GCTGAACTTAAGCATATCAATAAGCGGAGGAAAAGAAACTAACAAGGATTCCCCTAGTAACGGCGAGTGAAGAGGGATAAGCTCAAATTTTAAATCTACC-TGG---TTC--CCAGGTCGAGTTGTAATTTGAAGAAGCGATATC-GG-TG-TGGAGGTCTGGTTCAAGTTCTTTGGAACAAGACATCATGGA-AGGGTGAGAATCCCGTGCATGATCAGA-CC---AAGAT--AC-CTAAT---ATTCGTTTTCTAAGAGTCGAGTTGTTTGGGAATGCAGCTCAAAATGGG-T-GGTAGAC-TTCACCTAAGGCTAAATATCAGCGAGAGACCGATAGCGAACAAGTACTGTG-AA-GGAAAGATGAAAAGAACTTTGAAAAGAGAGTTAAATAGTACGTGAAATTGTTGAAAGGGAAACGATTGAAGTCAGTCATGCTAG-TGAAAATTCAGTTTGTCGGGTTTTTA-AGTTTGGGAGT--AGGAGGCAAGC--------------CTCTCTTTTGAACTTTTAATCTGTCAGATGCACTTTTTCT-TTGGCAGGTCAGCGTCGATTTC-GGAGGTTGTAAA---------ATACTTGGGGG-AAAGTAGCTCTGCTTCGGGA---GAGTG-TTATAGGCCCTGGGGGATGCAGCCTGCGGGATCGAGGATTGCAGCAAATGC------------------TTTTTT--GGC-TTGTCGCCTGATCTCTGG-ACGTTACCTCGCTTGTGACAACATT-CTTGCCACCGGTGAGTACTAATGGCTATTAGGTTAGAGCGATCAAAAA-TATTGCTAAGGATGCTGACGTAATGGCTTTAAACGACCCGTCTTGAAACACGGACCAAGGAGTCTAACATGTGTGCGAG----------------------------------------------------------------------------------------------------------------------------------------------------------------------------------------------------------------------------------------------------------------------------------------------------------------------------------------------------------------------------------------------------------------------------------------------------------------------------------------------------------------------------------------------------------------------------------------------------------------------------------------------------------------------------------------------------------------------------------------------------------------------------------------------------------------------------------------------------------------------------------------------------------------------------------------------------------------------------------------------------------------------------------------------------------------------------------------------------------------------------------------------------------------------------------------------------------

>Diversispora_jakucsiae_KJ850184_MG459190

AGGAATCCCTAGTAAGCATGAGTCATCAGCTCATG-TTGATTACGTCCCTGCCCTTTGTACACACCGCCCGTCGCTACTACCGATTGAATGGCTTAGTGAGACCTTTGGATTGGGGTTTAGGGATCGGAAAC---GATCTTTATTCCACGAGAAGTCGGTCAAACTTGGTCATTTAGAGGAAGTAAAAGTCGTAACAAGGTTTCCGTAGGTGAACCTGCGGAAGGATCATT--AAAAAT-ATTTA-TCCGGGAATACG----TTTCCGT----T--------TTCCCG-G------ATTATTTGTATTCAAA-TCCCACTCTTT-------AT-AAAT-ATA---------TCAATTATATAAAAC-AAAAA-A-TAAAAAAGAAAACTTTCAACAACGGATCTCTTGGCTCTCGCATCGATGAAGAACGCAGCGAAATGCGATACGTAATGTGAATTGCAG-ATTCCGTGAATCATCGAA-TCTTTGAACGCAAATTGTACTTTCCAGTAATCTGGGGAGTATGCTTGGTTGAGGGTCATTAAAATAACA-TTCGTGAA---------------TTTTTCG-------------CGGATTTGAG----TTTT-CC--AGTA--TTC---AT---TAT------AAT------GA-TAAATGTTGGTGACTTT-AAAATT-ATTT------ATAACTTGATACAAGTT-GAAAACGTGC-TATA--TGTGT-GGTTCGCT-GACAACTTGTCCA--T-C--TC----T-ATAT-ACTATGCG-CGCACTTGG----TTTT----TTCACTCTGTGC--GAAC--ATATA------TTTTTTTTATGAC-CTCAGCTCAAGCAAGAATACCC-GCTGAACTTAAGCATATCAATAAGCGGAGGAAAAGAAACTAACAAGGATTCCCCTAGTAACGGCGAGTGAAGAGGGATAAGCTCAAATTTTAAATCTACC-TGG---TTC--CCAGGTCGAGTTGTAATTTGAAGAAGCGATATC-GG-TG-TGGAGGTCTGGTTCAAGTTCTTTGGAACAAGACATCATGGG-AGGGTGAGAATCCCGTGCATGATCAGA-CC---AAGAT--AC-CTAAT---ATTCGTTTTCTAAGAGTCGAGTTGTTTGGGAATGCAGCTCAAAATGGG-T-GGTAGAC-TTCACCTAAGGCTAAATATCAGCGAGAGACCGATAGCGAACAAGTACTGTG-AA-GGAAAGATGAAAAGAACTTTGAAAAGAGAGTTAAATAGTACGTGAAATTGTTGAAAGGGAAACGATTGAAGTCAGTCATGCTAG-TGAAAATTCAGTTTGTCGGGTTTTTA-AGTTTGGGAGT--AGGAGGCAAGC--------------CTCTCTTTTGAACTTTTAATCTGTCAGATGCACTTTTTCT-TTGGCAGGTCAGCGTCGATTTC-GGAGGTTGTAAA---------ATACTTGGGGG-AAAGTAGCTCTGCTTCGGGA---GAGTG-TTATAGGCCCTGGGGGATGCAGCCTGCGGGATCGAGGATTGCAGCAAATGC------------------TTTTTT--GGC-TTGTCGCCTGATCTCTGG-ACGTTACCTCGCTTGTGACAACATT-CTTGCCACCGGTGAGTACTAATGGCTATTAGGTTAGAGCGATCAAAAA-TATTGCTAAGGATGCTGACGTAATGGCTTTAAACGACCCGTCTTGAAACACGGACCAAGGAGTCTAACATGTGTGCGAG------------------------------------------------------------------------------gtggagaagatgatttgacacacaaattgtccgacatcttgaaggcaaatcaaaacgtaaaacgttatgaagctgatggtcatcccccacacgttgtaaacgaatttgaagcattgttacaggttcttaattat---------------------------------------------------------------------------------------------------------tataa-tttca--------a-ttagtttaatatcaa--ttgaaa--tttatacttaaattttattatttac-----atgcaaacagtttcattgtgcaacttatatggacaatgaaatggctggtcaacctcaagctcttcagaaatctggtagacctttaaagtcaatacgtgcgcgtctcaagggtaaagaaggacgtttacgcggtaatctgatgggaaagcgtgtagatttctctgctcgtacagtaattacgggtgatccaaatatttcagttgatgaagtcggagttccgaaaagcatagctcaaaatttaacatttccagaattggtgactccctttaatattgattatcttcaaaaattagtagaaaatggcccttctacacatccaggggctaaatacgtaattagagatactggtgaaaggattgatctaaaacatatatcaggcatgactggtggcttaagattacactacggttggaaagttgaacgtcatctcaatgatggtgacatcgttatattcaatcgtcagccatctttgcacaaaatgtcaatgatggg-------------------------------------------------------------------------------------------------------------------------------------------------------------------------------------------------------------------------------------------------------------------------------------

>Diversispora_aurantia_FN547661_OL690407

AGGAATCCCTAGTAAGCGTGAGTCATCAGCTCACG-TTGATTACGTCCCTGCCCTTTGTACACACCGCCCGTCGCTACTACCGATTGAATGGCTTAGTGAGACCTTTGGATTGGGGTTTAGGGATCGGAAAC---GATCCTTATTCTCCGAGAAGTCGGTCAAACTTGGTCATTTAGAGGAAGTAAAAGTCGTAACAAGGTTTCCGTAGGTGAACCTGCGGAAGGATCATT-AAAAAAT-ATTTA-TCCGAGAATTCG-----TTTCGT----T--------TTCTCGGA------TTAA-TTGTATTCAAA-TCCCACTCTTT-------AT-AAAT-ACA---------TCAATTATATAAAAC--AAAA-A-TAAAAAAGAAAACTTTCAACAACGGATCTCTTGGCTCTCGCATCGATGAAGAACGCAGCGAAATGCGATACGTAGTGTGAATTGCAGAATTCCGTGAATCATCGAA-TCTTTGAACGCAAATTGTACTTTCCAGTAATCTGGGAAGTATGCTTGGTTGAGGGTCATCGAAATAACA-TTCGTGAA--------------TTTTTTCG-------------CGGATTTGAG----TTTT-CC--GGTA--TTT-ATCT---ATA------A--------TA-TAAATGTTGGTAACTTT-AAAATT-ATAT------ACTACTTGGTACAAGTT-GAAAACGTAC-TATG--T-TGT-GGTTCGCT-GACAACTTGTCCA--T-CTTTT----T-ATAT-ATTATGCG-CGCACTGAG----TTT-----TATATTCTGTGC--AAGT--ATATA-------TTTTTTTATGAC-CTCAGCTCAAGCAAGAGTACCC-GCTGAACTTAAGCATATCAATAAGCGGAGGAAAAGAAACTAACAAGGATTCCCCTAGTAACGGCGAGTGAAGAGGGAAAAGCTCAAATTTTAAATCTACC-TGGTTTAT---CCAGGTCGAGTTGTAATTTGAAGAAGCGATATC-GG-TG-TTGAGGTCTGGTTTAAGTTCTTTGGAACAAGACATCAT-GG-AGGGTGAGAATCCCGTGCATGATCAGA-CC---AAGAT--AC-TAAGT---ATATGCTTTCTAAGAGTCGAGTTGTTTGGGAATGCAGCTCAAAATGGG-T-GGTAGAC-TTCACCTAAGGCTAAATATCAGCGAGAGACCGATAGCGAACAAGTACTGTG-AA-GGAAAGATGAAAAGAACTTTGAAAAGAGAGTTAAATAGTACGTGAAATTGTTGAAAGGGAAACGATTGAAGTCAGTCATGCCAG-TGAAAATTCAGTTTGGCGGG-TTTCT-GGTTC-GGAGT--AGAGGCAGGGT---CAA---ACCGTCTCTCTTTTGGACTTGGGATTTGTCAGATGCATTTTTTCT-TTGGCAGGTCAGCGTCGGTTTC-GGGAGTTGTAAA---------ATACTTGGGGT-AAAGTAGCTCTGCTTCGGGA---GAGTG-TTATAGACCTTGGGGGATGCAGCTCGCGGGATCGAGGATTGCAGCAAATGC-------------------CTTTT--GGC-TTGTCGCCTGATCTCTGG-ATGTTACCTTGCTTGTGGCAACATT-CTTGCCACCGGCGAGTACTAATGCTCACTGAATTAGAGCGATCAAAAA-TTTTGCTAAGGATGCTGACGTAATGGCTTTAAACGACCCGTCTTGAAACACGGACCAAGGAGTCTAACATATGTGCGAGctgattggatgatcataactatacttcctgttcctcctcctcctgtacgtcccagtgtacaaatggacggaacaagtcgaggtgaagatgatttgactcacaaattatccgacatcttgaaggcaaatcaaaacgtaaaacgttatgaagctgatggtcatcccccacacgttgtaaacgaatttgaagcattgttacaggttcttaatggt---------------------------------------------------------------------------------------------------------tataa--ttca--------a-ttagtttattatcaa--ttaaga--tttatacttaaa-tttattatttat-----atgcaaacagtttcattgtgcaacttatatggacaatgaaatggccggtcaaccacaagctcttcagaaatctggtagacctttaaagtcaatacgtgcgcgtctcaagggtaaagaaggacgtttacgtggtaatctgatgggaaagcgtgtagatttctctgctcgtacagtaattacgggcgatccaaatatttcagttgatgaagtcggagttccgaaaagcatagctcaaaatttgacttttccagaattggtgactccctttaatattgactatcttcaaaaattagtagaaaatggcccttctacacatccaggggctaaatatgtaattagagatactggtgaaaggattgacctaaaacatatatcaggcatgactggtggattaagattacactacggttggaaagttgaacgtcatctcaatgatggtgacatcgttatattcaatcgtcagccatctttgcacaagatgtcgatgatgggacataaagttcgtgttatgccctattcgaccttccgtcttaatttatcagttacaacaccttataacgccgattttgacggtgacgaaatgaacatgcatgttccccaatcagttgaaactaaagcagaaatttcagaaatatgcatggttcctaaacaaattgtatctcctcaatcaaataaacctgttatgggtattgtacaggatactttatgtgctgttagaaaatttacaaaaagagattgctttttatctaaagatttggtaatgaacatt

>Diversispora_aurantia_FN547664

AGGAATCCCTAGTAAGCGTGAGTCATCAGCTCACG-TTGATTACGTCCCTGCCCTTTGTACACACCGCCCGTCGCTACTACCGATTGAATGGCTTAGTGAGACCTTTGGATTGGGGTTTAGGGATCGGAAAC---GATCCTTATTCTCCGAGAAGTCGGTCAAACTTGGTCATTTAGAGGAAGTAAAAGTCGTAACAAGGTTTCCGTAGGTGAACCTGCGGAAGGATCATT-AAAAAAT-ATTTA-TCCGAGAATTCG-----TTTCGT----T--------TTCTCGGA------TTAA-TTGTATTCAAA-TCCCACTCTTT-------AT-AAAT-ACA---------TCAATTATATAAAAC--AAAA-A-TAAAAAAGAAAACTTTCAACAACGGATCTCTTGGCTCTCGCATCGATGAAGAACGCAGCGAAATGCGATACGTAGTGTGAATTGCAGAATTCCGTGAATCATCGAA-TCTTTGAACGCAAATTGTACTTTCCAGTAATCTGGGAAGTATGCTTGGTTGAGGGTCATCGAAATAACA-TTCGTGAA--------------TTTTTTCG-------------CGGATTTGAG----TTTT-CC--GGTA--TTT-ATCT---ATA------A--------TA-TAAATGTTGGTAACTTT-AAAATT-ATAT------ACTACTTGGTACAAGTT-GAAAACGTAC-TATG--T-TGT-GGTTCGCT-GACAACTTGTCCA--T-CTTTT----T-ATAT-ATTATGCG-CGCACTGAG----TTT-----TATATTCTGTGC--AAGT--ATATA-------TTTTTTTATGAC-CTCAGCTCAAGCAAGAGTACCC-GCTGAACTTAAGCATATCAATAAGCGGAGGAAAAGAAACTAACAAGGATTCCCCTAGTAACGGCGAGTGAAGAGGGAAAAGCTCAAATTTTAAATCTACC-TGGTTTAT---CCAGGTCGAGTTGTAATTTGAAGAAGCGATATC-GG-TG-TTGAGGTCTGGTTTAAGTTCTTTGGAACAAGACATCAT-GG-AGGGTGAGAATCCCGTGCATGATCAGA-CC---AAGAT--AC-TAAGT---ATATGCTTTCTAAGAGTCGAGTTGTTTGGGAATGCAGCTCAAAATGGG-T-GGTAGAC-TTCACCTAAGGCTAAATATCAGCGAGAGACCGATAGCGAACAAGTACTGTG-AA-GGAAAGATGAAAAGAACTTTGAAAAGAGAGTTAAATAGTACGTGAAATTGTTGAAAGGGAAACGATTGAAGTCAGTCATGCCAG-TGAAAATTCAGTTTGGCGGG-TTTCT-GGTTC-GGAGT--AGAGGCAGGGT---CAA---ACCGTCTCTCTTTTGGACTTGGGATTTGTCAGATGCATTTTTTCT-TTGGCAGGTCAGCGTCGGTTTC-GGGAGTTGTAAA---------ATACTTGGGGT-AAAGTAGCTCTGCTTCGGGA---GAGTG-TTATAGACCTTGGGGGATGCAGCTCGCGGGATCGAGGATTGCAGCAAATGC-------------------CTTTT--GGC-TTGTCGCCTGATCTCTGG-ATGTTACCTTGCTTGTGGCAACATT-CTTGCCACCGGCGAGTACTAATGCTCACTGAATTAGAGCGATCAAAAA-TTTTGCTAAGGATGCTGACGTAATGGCTTTAAACGACCCGTCTTGAAACACGGACCAAGGAGTCTAACATATGTGCGAG----------------------------------------------------------------------------------------------------------------------------------------------------------------------------------------------------------------------------------------------------------------------------------------------------------------------------------------------------------------------------------------------------------------------------------------------------------------------------------------------------------------------------------------------------------------------------------------------------------------------------------------------------------------------------------------------------------------------------------------------------------------------------------------------------------------------------------------------------------------------------------------------------------------------------------------------------------------------------------------------------------------------------------------------------------------------------------------------------------------------------------------------------------------------------------------------------------

>Diversispora_aurantia_FN547655

AGGAATCCCTAGTAAGCGTGAGTCATCAGCTCACGCTTGATTACGTCCCTGCCCTTTGTACACACCGCCCGTCGCTACTACCGATTGAATGGCTTAGTGAGACCTTTGGATTGGGGTTTAGGGATCGGAAAC---GATCCTTATTCTCCGAGAAGTCGGTCAAACTTGGTCATTTAGAGGAAGTAAAAGTCGTAACAAGGTTTCCGTAGGTGAACCTGCGGAAGGATCATT-AAAAAAT-ATTTA-TCCGAGAATTCG-----TTTCGT----T--------TTCTCGGA------TTAA-TTGTATTCAAA-TCCCACTCTTT-------AT-AAAT-ACA---------TCAATTATATAAAAC--AAAA-A-TAAAAAAGAAAACTTTCAACAACGGATCTCTTGGCTCTCGCATCGATGAAGAACGCAGCGAAATGCGATACGTAGTGTGAATTGCAGAATTCCGTGAATCATCGAA-TCTTTGAACGCAAATTGTACTTTCCAGTAATCTGGGAAGTATGCTTGGTTGAGGGTCATCGAAATAACA-TTCGTGAA--------------TTTTTTCG-------------CGGATTTGAG----TTTT-CC--AGGA--TTT-ATCT---ATA------A--------TA-TGAATGTTGGTAACTTT-AAAATT-ATAT------ACTACTTGTTACAAGTT-GAAAACGTAC-TATG--T-TGT-GGTTCGCT-GACAACTTGTCCA--T-CTTTT----T-ATAT-ATTATGCG-CGCACTGAG----TTT-----TATATTCTGTGC--AAGT--ATATA-----TTTTTTTTTATGAC-CTCAGCTCAAGCAAGAGTACCC-GCTGAACTTAAGCATATCAATAAGCGGAGGAAAAGAAACTAACAAGGATTCCCCTAGTAACGGCGAGTGAAGAGGGAAAAGCTCAAATTTTAAATCTACT-TGGTTCGTC--CCAGGTCGAGTTGTAATTTGAAGAAGCGATATC-GG-TG-TTGAGGTCTGGTTTAAGTTCTTTGGAACAAGACATCAT-GG-AGGGTGAGAATCCCGTGCATGATCAGA-CC---AAGAT--AC-TAAGT---ATATGCTTTCTAAGAGTCGAGTTGTTTGGGAATGCAGCTCAAAATGGG-T-GGTAGAC-TTCACCTAAGGCTAAATATCAGCGAGAGACCGATAGCGAACAAGTACTGTG-AA-GGAAAGATGAAAAGAACTTTGAAAAGAGAGTTAAATAGTACGTGAAATTGTTGAAAGGGAAACGATTGAAGTCAGTCATGCCAG-TGAAAACTCAGTTTGGCGGG-TTTCT-GGTTC-GGAGT--AGAGGCAGGGT---CAA---ACCGTCTCTCTTTTGGACTTGGGATTTGTCAGATGCATTTTTTCT-TTGGCAGGTCAGCGTCGGTTTC-GGGAGTTGTAAA---------ATACTTGGGGT-AAAGTAGCTCTGCTTCGGGA---GAGTG-TTATAGACCTTGGGGGATGCAGCTCGTGGGATCGAGGATTGCAGCAAATGC-------------------TTTTT--GGC-TTGTCGCCTGATCTCTGG-ATGTTACCTTGCTTGTGGCAACATT-CTTGCCACCGGCGAGTACTAATGCTCACTAGGTTAGAGCGATCAAAAA-TTTTGCTAAGGATGCTGACGTAATGGCTTTAAACGACCCGTCTTGAAACACGGACCAAGGAGTCTAACATATATGCGAG----------------------------------------------------------------------------------------------------------------------------------------------------------------------------------------------------------------------------------------------------------------------------------------------------------------------------------------------------------------------------------------------------------------------------------------------------------------------------------------------------------------------------------------------------------------------------------------------------------------------------------------------------------------------------------------------------------------------------------------------------------------------------------------------------------------------------------------------------------------------------------------------------------------------------------------------------------------------------------------------------------------------------------------------------------------------------------------------------------------------------------------------------------------------------------------------------------

>Diversispora_aurantia_FN547657_OL690408

AGGAATCCCTAGTAAGCGTGAGTCATCAGCTCACG-TTGATTACGTCCCTGCCCTTTGTACACACCGCCCGTCGCTACTACCGATTGAATGGCTTAGTGAGACCTTTGGATTGGGGTTTAGGGATCGGAAAC---GATCCTTATTCTCCGAGAAGTCGGTCAAACTTGGTCATTTAGAGGAAGTAAAAGTCGTAACAAGGTTTCCGTAGGTGAACCTGCGGAAGGATCATT-AAAAAAT-CTTTA-TCCGAGAATTCG-----TTTCGT----T--------TTCTCGGA------TTAATTTGTATTCAAA-TCCCACTCTTT-------ATAAAAT-ATA---------TCAATTATATAAAAC--AAAA-A-TAAAAAAGAAAACTTTCAACAACGGATCTCTTGGCTCTCGCATCGATGAAGAACGCAGCGAAATGCGATACGTAGTGTGAATTGCAGAATTCCGTGAATCATCGAA-TCTTTGAACGCAAATTGTACTTTCCAGTAATCTGGGAAGTATGCTTGGTTGAGGGTCATCGAAATAACA-TTCGTGAA--------------TTTTTTCG-------------CGGATTTGAG----TTTT-CC--AGGA--TTT-ATCT---ATA------A--------TA-TGAATGTTGGTAACTTT-AAAATT-ATAT------ACTACTTGGTACAAGTT-GAAAACGTAC-TATG--T-TGT-GGTTCGCT-GACAACTTGTCCA--T-CTTTT----T-ATAT-ATTATGCG-CGCACTGAG----TTT-----TATATTCTGTGC--AAGT--ATATA-----TTTTTTTTTATGAC-CTCAGCTCAAGCAAGAGTACCC-GCTGAACTTAAGCATATCAATAAGCGGAGGAAAAGAAACTAACAAGGATTCCCCTAGTAACGGCGAGTGAAGAGGGAAAAGCTCAAATTTTAAATCTACT-TGGTTCGTC--CCAGGTCGAGTTGTAATTTGAAGAAGCGATATC-GG-TG-TTGAGGTCTGGTTTAAGTTCTTTGGAACAAGACATCAT-GG-AGGGTGAGAATCCCGTGCATGATCAGA-CC---AAGAT--AC-TAAGT---ATATGCTTTCTAAGAGTCGAGTTGTTTGGGAATGCAGCTCAAAATGGG-T-GGTAGAC-TTCACCTAAGGCTAAATATCAGCGAGAGACCGATAGCGAACAAGTACTGTG-AA-GGAAAGATGAAAAGAACTTTGAAAAGAGAGTTAAATAGTACGTGAAATTGTTGAAAGGGAAACGATTGAAGTCAGTCATGCCAG-TGAAAACTCAGTTTGGCGGG-TTTCT-GGTTC-GGAGT--AGAGGCAGGGT---CAA---ACCGTCTCTCTTTTGGACTTGGGATTTGTCAGATGCATTTTTTCT-TTGGCAGGTCAGCGTCGGTTTC-GGGAGTTGTAAA---------ATACTTGGGGT-AAAGTAGCTCTGCTTCGGGA---GAGTG-TTATAGACCTTGGGGGATGCAGCTCGTGGGATCGAGGATTGCAGCAAATGC-------------------TTTTT--GGC-TTGTCGCCTGATCTCTGG-ATGTTACCTTGCTTGTGGCAACATT-CTTGCCACCGGCGAGTACTAATGCTCACTAGGTTAGAGCGATCAAAAA-TTTTGCTAAGGATGCTGACGTAATGGCTTTAAACGACCCGTCTTGAAACACGGACCAAGGAGTCTAACATGTGTGCGAGctgattggatgatcataactatacttcctgttcctcctcctcctgtacgtcccagtatacaaatggacggaacaagtcgaggtgaagatgatttgactcacaaattatccgacatcttgaaggcaaatcaaaacgtaaaacgttatgaagctgatggtcatcccccacacgttgtaaacgaatttgaagcattgttacaggttcttaatggt---------------------------------------------------------------------------------------------------------tataa--ttca--------a-ttagtttattatcaa--ttaaaa--tttatacttaaa-tttattatttat-----atgcaaacagtttcattgtgcaacttatatggacaatgaaatggccggtcaaccacaagctcttcagaaatctggtagacctttaaagtcaatacgtgcgcgtctcaagggtaaagaaggacgtttacgtggtaatctgatgggaaagcgtgtagatttctctgctcgtacagtaattacgggcgatccaaatatttcagttgatgaagtcggagttccgaaaagcatagctcaaaatttgacttttccagaattggtgactccctttaatattgactatcttcaaaaattagtagaaaatggcccttctacacatccaggggctaaatatgtaattagagatactggtgaaaggattgacctaaaacatatatcaggcatgactggtggattaagattacactacggttgggaagttgaacgtcatctcaatgatggtgacatcgttatattcaatcgtcagccatctttgcacaagatgtcgatgatgggacataaagttcgtgttatgccctattcgaccttccgtcttaatttatcagttacaacaccttataacgccgattttgacggtgacgaaatgaacatgcatgttccccaatcagttgaaactaaagcagaaatttcagaaatatgcatggttcctaaacaaattgtatctcctcaatcaaataaacctgttatgggtattgtacaggatactttatgtgctgttagaaaatttacaaaaagagattgctttttatctaaagatttggtaatgaacatt

>Diversispora_alba_OP195880

AGGAATCCCTAGTAAGCGTGAGTCATCAGCTCACG-TTGATTACGTCCCTGCCCTTTGTACACACCGCCCGTCGCTACTACCGATTGAATGGCTTAGTGAGACCTTTGGATTGGGGTTTAGGGATCGGAAAC---GATCCTTATTCTTCGAGAAGTCGGTCAAACTTGGTCATTTAGAGGAAGTAAAAGTCGTAACAAGGTTTCCGTAGGTGAACCTGCGGAAGGATCATT-AAAAAAT-ATTTA-TCCGAGAATTCG-----TTTCGT----T--------TTCCGG-A------TTAATTTGTATTCAAA-TCCCACTCTTT-------AT-AAAT-ATA---------TCAATTATATAAAAT-AAAAA-T-AAAAAAAGAAAACTTTCAACAACGGATCTCTTGGCTCTCGCATCGATGAAGAACGCAGCGAAATGCGATACGTAGTGTGAATTGCAGAATTCCGTGAATCATCGAA-TCTTTGAACGCAAATTGTACTTTCCAGTAATCTGGGAAGTATGCTTGGTTGAGGGTCATTGAAATAACA-TTCGTGAT--------------TTTTTTCG-------------CGGATTTGAG----TTTT-CC--AGTA--TTT-ATTA------------A--------TA-TAGATGTTGGTAACTTT-AAAATT-ATTT------ATTACTTGGTACAAGTT-GAAAACGTAC-TATG--T-TGT-GGTTCGCT-GACAACTTGTCCA--T-C--TT----T-ATAT-ATTATGCG-CGCACTTGG----TTT-----TATATTTCGTGC--AAGC--ATATA--------TTTTTTTTGAC-CTCAGCTCAAGCAAGAGTACCC-GCTGAACTTAAGCATATCAATAAGCGGAGGAAAAGAAACTAACAAGGATTCCCCTAGTAACGGCGAGTGAAGAGGGAAAAGCTCAAATTTTAAATCTACC-TGG---TTC--CCAGGTCGAGTTGTAATTTGAAGAAGCGATATC-GG-TG-TTGAGGTCTGGTTTAAGTTCTTTGGAACAAGACATCAT-GG-AGGGTGAGAATCCCGTGCATGATCAGA-CC---AAGAT--AC-TTAGT---ATTCGCTTTCCAAGAGTCGAGTTGTTTGGGGATGCAGCTCAAAATGGG-T-GGTAGAC-TTCACCTAAGGCTAAATATCAGCGAGAGACCGATAGCGAACAAGTACTGTG-AA-GGAAAGATGAAAAGAACTTTGAAAAGAGAGTTAAATAGTACGTGAAATTGTTGAAAGGGAAACGATTGAAGTCAGTCATGCCAG-TGAAAATTCAGTTTGGCGGG-TTTCT-CGTTTAGGAGT--AGAGGCAGGGT---TAA---ACCGTCTCTCTTTTGAAC-GGATTCTTGTCAGATGCATTTTTTCT-CTTGCAGGTCAGCGTCGGTTTC-GGGGGTTGTAAA---------ATACTTGGGGT-AAAGTAGCTCTGCTTCGGGA---GAGTG-TTATAGACCTTGGGGGATGCAGCCCGCGGGATCGAGGATTGCAGCAAATGC--------------------CTTT--GGC-TTGTCGCCTGATCTCTGG-ATGTTACCTTGCTTGCAACAACATT-CTTGTTGCCGGTGAGTACTAATGCTCATTAGGTTAGAGCGATCAAAAA-TATTGCTAAGGATGCTGACGTAATGGCTTTAAACGACCCGTCTTGAAACACGGACCAAGGAGTCTAACATATGTGCGAG----------------------------------------------------------------------------------------------------------------------------------------------------------------------------------------------------------------------------------------------------------------------------------------------------------------------------------------------------------------------------------------------------------------------------------------------------------------------------------------------------------------------------------------------------------------------------------------------------------------------------------------------------------------------------------------------------------------------------------------------------------------------------------------------------------------------------------------------------------------------------------------------------------------------------------------------------------------------------------------------------------------------------------------------------------------------------------------------------------------------------------------------------------------------------------------------------------

>Diversispora_alba_OP195882

AGGAATCCCTAGTAAGCGTGAGTCATCAGCTCACG-TTGATTACGTCCCTGCCCTTTGTACACACCGCCCGTCGCTACTACCGATTGAATGGCTTAGTGAGACCTTTGGATTGGGGTTTAGGGATCGGAAAC---GATCCTTATTCTCCGAGAAGTCGGTCAAACTTGGTCATTTAGAGGAAGTAAAAGTCGTAACAAGGTTTCCGTAGGTGAACCTGCGGAAGGATCATT-AAAAAAT-ATTTA-TCCGAGAATTCG-----TTTCGT----T--------TTCTCGGA------TTAATTTGTATTCAAA-TCCCGCTCTTT-------AT-AAAT-ATA---------TCAATTATATAAAAT-AAAAA-T-AAAAAAAGAAAACTTTCGACAACGGATCTCTTGGCTCTCGCATCGATGAAGAACGCAGCGAAATGCGATACGTAGTGTGAATTGCAGAATTCCGTGAATCATCGAA-TCTTTGAACGCAAATTGTACTTTCCAGTAATCTGGGAAGTATGCTTGGTTGAGGGTCATTGAAATAACA-TTCGTGAT--------------TTTTTTCG-------------CGGATTTGAG----TTTT-CC--AGTA--TTT-ATTA------------A--------TA-TAGATGTTGGTAATTTT-AAAATT-ATTT------ATTACTTGGTACAAGTT-GAAAACGTAC-TATG--T-TGT-GGTTCGCT-GACAACTTGTCCA--T-C--TT----T-ATAT-ATTATGCG-CGCACTTGG----TTT-----TATATTTCGTGC--AAGC--ATATA--------TTTTTTTTGAC-CTCAGCTCAAGCAAGAGTACCC-GCTGAACTTAAGCATATCAATAAGCGGAGGAAAAGAAACTAACAAGGATTCCCCTAGTAACGGCGAGTGAAGAGGGAAAAGCTCAAATTTTAAATCTACC-TGG---TTC--CCAGGTCGAGTTGTAATTTGAAGAAGCGATATT-GG-TG-TTGAGGTCTGGTTTAAGTTCTTTGGAACAAGACATCAT-GG-AGGGTGAGAATCCCGTGCATGATCAGA-CC---AAGAT--AC-TAAGT---ATATGCTTTCTAAGAGTCGAGTTGTTTGGGAATGCAGCTCAAAATGGG-T-GGTAGAC-TTCACCTAAGGCTAAATATCAGCGAGAGACCGATAGCGAACAAGTACTGTG-AA-GGAAAGATGAAAAGAACTTTGAAAAGAGAGTTAAATAGTACGTGAAATTGTTGAAAGGGAAACGATTGAAGTCAGTCATGCCAG-TGAAAATTCAGTTTGGCGGG-TTTCT-CGTTTAGGAGT--AGAGGCAGGGT---CAA---ACCGTCTCTCTTTTGAAC-GGATTCTTGTCAGATGCATTTTTTCT-CTTGCAGGTCAGCGTCGGTTTC-GGGGGTTGTAAA---------ATACTTGGGGT-AAAGTAGCTCTGCTTCGGGA---GAGTG-TTATAGACCTTGGGGGATGCAGCCCGCGGGATCGAGGATTGCAGCAAATGC--------------------CTTT--GGC-TTGTCGCCTGATCTCTGG-ATGTTACCTTGCTTGCAACAACATT-CTTGTTGCCGGTGAGTACTAATGCTCACTAGGTTAGAGCGATCAAAAATTTTTGCTAAGGATGCTGACGTAATGGCTTTAAACGACCCGTCTTGAAACACGGACCAAGGAGTCTAACATATGTGCGAG----------------------------------------------------------------------------------------------------------------------------------------------------------------------------------------------------------------------------------------------------------------------------------------------------------------------------------------------------------------------------------------------------------------------------------------------------------------------------------------------------------------------------------------------------------------------------------------------------------------------------------------------------------------------------------------------------------------------------------------------------------------------------------------------------------------------------------------------------------------------------------------------------------------------------------------------------------------------------------------------------------------------------------------------------------------------------------------------------------------------------------------------------------------------------------------------------------

>Diversispora_alba_OP195886

AGGAATCCCTAGTAAGCGTGAGTCATCAGCTCACG-TTGATTACGTCCCTGCCCTTTGTACACACCGCCCGTCGCTACTACCGATTGAATGGCTTAGTGAGACCTTTGGATTGGGGTTTAGGGATCGGAAAC---GATCCTTATTCTCCGAGAAGTCGGTCAAACTTGGTCATTTAGAGGAAGTAAAAGTCGTAACAAGGTTTCCGTAGGTGAACCTGCGGAAGGATCATTAAAAAAAT-ATTTA-TCCGAGAATTCG-----TTTCGT----T--------TTCCGGAT------TAAATTTGTATTCAAA-TCCTACTCTTT-------AT-AAAT-ATA---------TCAATTATATAAAAC--AAAA-T-AAAAAAAGAAAACTTTCAACAACGGATCTCTTGGCTCTCGCATCGATGAAGAACGCAGCGAAATGCGATACGTAGTGTGAATTGCAGAATTCCGTGAATCATCGAA-TCTTTGAACGCAAATTGTACTTTCCAGTACTCTGGGAAGTATGCTTGGTTGAGGGTCATCGAAATAACA-TTCGTGAT--------------TTTTTTCG-------------CGGATTTGAG----TTTT-CC--AGTA--TTT-ATTA------------A--------TA-CAGATGTTGGTAACTTT-AAAATT-ATTT------ATTACTTGGTACAAGTT-GAAAACGTAC-TATG--T-TGT-GGTTCGCT-GACAACTTGTCCA--T-C--TT----T-ATAT-ATTATGCG-CGCACTTGG----TTT-----TATATTCTGTGC--AAGC--ATATA-------TTTTTTTTTGAC-CTCAGCTCAGGCAAGAGTACCC-GCTGAACTTAAGCATATCAATAAGCGGAGGAAAAGAAACTAACAAGGATTCCCTTAGTAACGGCGAGTGAAGAGGGAAAAGCTCAAATTTTAAATCTACC-TGGTTCTTC--CCAGGTCGAGTTGTAATTTGAAGAAGCGATATC-GG-TA-TTGAGGTCTGGTTTAAGTTCTTTGGAACAAGACATCAT-GG-AGGGTGAGAATCCCGTGCATGATCAGA-CC---AAGAT--GC-TAAGT---ATATGCTTTCTAAGAGTCGAGTTGTTTGGGAATGCAGCTCAAAATGGG-T-GGTAGAC-TTCACCTAAGGCTAAATATCAGCGAGAGACCGATAGCGAACAAGTACTGTG-AA-GGAAAGATGAAAAGAACTTTGAAAAGAGAGTTAAATAGTACGTGAAATTGTTGAAAGGGAAACGATTGAAGTCAGTCATGCCAG-TGAAAATTCAGTTTGGCGGG-TTTCT-CGTTTAGGAGT--AGAGGCAGGGT---TAA---ACCGTCTCTCTTTTGAAC-GGATTCTTGTCAGATGCATTTTTTCT-CTTGCAGGTCAGCGTCGGTTTC-GGGGGTTGTAAA---------ATACTTGGGGT-AAAGTAGCTCTGCTTCGGGA---GAGTG-TTATAGACCTTGGGGGATGCAGCCCGCGGGATCGAGGATTGCAGCAAATGC--------------------CTTT--GGC-TTGTCGCCTGATCTCTGG-ATGTTACCTTGCTTGCAACAACATT-CTTGTTGCCGGTGAGTACTAATGCTCACTAGGTTAGAGCGATCAAAAA-TATTGCTAAGGATGCTGACGTAATGGCTTTAAACGACCCGTCTTGAAACACGGACCAAGGAGTCTAACATATGTGCGAG----------------------------------------------------------------------------------------------------------------------------------------------------------------------------------------------------------------------------------------------------------------------------------------------------------------------------------------------------------------------------------------------------------------------------------------------------------------------------------------------------------------------------------------------------------------------------------------------------------------------------------------------------------------------------------------------------------------------------------------------------------------------------------------------------------------------------------------------------------------------------------------------------------------------------------------------------------------------------------------------------------------------------------------------------------------------------------------------------------------------------------------------------------------------------------------------------------

>Diversispora_alba_OP195889

AGGAATCCCTAGTAAGCGTGAGTCATCAGCTCACG-TTGATTACGTCCCTGCCCTTTGTACACACCGCCCGTCGCTACTACCGATTGAATGGCTTAGTGAGACCTTTGGATTGGGGTTTAGGGATCGGAAAC---GATTCTTATTCTCCGAGAAGTCGGTCAAACTTGGTCATTTAGAGGAAGTAAAAGTCGTAACAAGGTTTCCGTAGGTGAACCTGCGGAAGGATCATT-AAAAAAT-ATTTA-TCCGAGAATTCG-----TTTCGT----T--------TTCCGGAT------TAAATTTGTATTCAAA-TCCTACTCTTT-------AT-AAAT-ATA---------TCAATTATATAAAAT--AAAA-TAAAAAAAAGAAAACTTTCAACAACGGATCTCTTGGCTCTCGCATCGATGAAGAACGCAGCGAAATGCGATACGTAGTGTGAATTGCAGAATTCCGTGAATCATCGAA-TCTTTAAACGCAAATTGTACTTTCCAGTACTCTGGGAAGTATGCTTGGTTGAGGGTCATCGAAATAACA-TTCGTGAA--------------TTTTTTCG-------------CGGATTTGAG----TTTT-CC--AGTA--TTT-ATTA------------A--------TA-TAGATGTTGGTAACTTT-AAAATT-ATTT------ATTACTTGGTACAAGTT-GAAAACGTAC-TATG--T-TGT-GGTTCGCT-GACAACTTGTCCA--T-C--TT----T-ATAT-ATTATGCG-CGCACTTGG----TTTTA---TATATTCTGTGC--AAGC--ATATA------TTTTTTTTTTGAC-CTCAGCTCAAGCAAGAGTACCC-GCTGAACTTAAGCATATCAATAAGCGGAGGAAAAGAAACTAACAAGGATTCCCTTAGTAACGGCGAGTGAAGAGGGAAAAGCTCAAATTTTAAATCTACC-TGGTTCTTC--CCAGGTCGAGTTGTAATTTGAAGAAGCGATATC-GG-TA-TTGAGGTCTGGTTTAAGTTCTTTGGAACAAGACATCAT-GG-AGGGTGAGAATCCCGTGCATGATCAGA-CC---AAGAT--GC-TAAGT---ATATGCTTTCTAAGAGTCGAGTTGTTTGGGAATGCAGCTCAAAATGGG-T-GGTAGAC-TTCACCTAAGGCTAAATATCAGCGAGAGACCGATAGCGAACAAGTACTGTG-AA-GGAAAGATGAAAAGAACTTTGAAAAGAGAGTTAAATAGTACGTGAAATTGTTGAAAGGGAAACGATTGAAGTCAGTCATGCCAG-TGAAAATTCAGTTTGGCGTG-GATCT-CCTTCAGGAGT--AGAGGCAGGGT---CAA---ACCGTCTCTCTTTTGAAC-GGATTCTTGTCAGATGCATTTTTTCT-CTTGCAGGTCAGCGTCGGTTTC-GGGGGTTGTAAA---------ATACTTGGGGT-AAAGTAGCCCTGCTTCGGGA---GAGTG-TTATAGACCTTGGGGGATGCAGCCCGCGGGATCGAGGATTGCAGCAAATGC--------------------CTTT--GGC-TTGTCGCCTGATCTCTGG-ATGTTACCTTGCTTGCAACAACATT-CTTGTTGCCGGTGAGTACTAATGCTCACTAGGTTAGAGCGATCAAAAA-TTTTGCTAAGGATGCTGACGTAATGGCTTTAAACGACCCGTCTTGAAACACGGACCAAGGAGTCTAACATATGTGCGAG----------------------------------------------------------------------------------------------------------------------------------------------------------------------------------------------------------------------------------------------------------------------------------------------------------------------------------------------------------------------------------------------------------------------------------------------------------------------------------------------------------------------------------------------------------------------------------------------------------------------------------------------------------------------------------------------------------------------------------------------------------------------------------------------------------------------------------------------------------------------------------------------------------------------------------------------------------------------------------------------------------------------------------------------------------------------------------------------------------------------------------------------------------------------------------------------------------

>Diversispora_spurca_MG459207_OL690411

AGGAATCCCTAGTAAGCGTGAGTCATCAGCTCACG-TTGATTACGTCCCTGCCCTTTGTACACACCGCCCGTCGCTACTACCGATTGAATGGCTTAGTGAGACCTTTGGATTGGTGTTTAGGGATCGGAAAC---GATCCTTATTCTCCGAGAAGTCGGTCAAACTTGGTCATTTAGAGGAAGTAAAAGTCGTAACAAGGTTTCCGTAGGTGAACCTGCGGAAGGATCATT-AAAAAAT-CAATA-TCCGAGAATTCGT---TTTTCGT----T--------TTCTCGGA------TTTATTTGTATTCAAA-TCCCACTCTTT-------AT-AAAT-ATA---------TCAATTATATAAAAC--AAAA-T-AAAAAAAGAAAACTTTCAACAACGGATCTCTTGGCTCTCGCATCGATGAAGAACGCAGCGAAATGCGATACGTAGTGTGAATTGCAGAATTCCGTGAATCATCGAA-TCTTTGAACGCAAATTGTACTTTTCAGTAATCTGGGAAGTATGCTTGGTTGAGGGTCATCGAAATTACA-TTCGTGA---------------TTTTTTCG-------------CGGATTTGAG----TTTT-CC--AGTA--TTT-ATTA------------A--------TA-TAAATGTTGGTAACTTT-AAAATT-ATTT------ATTACTTGGTGCAAGTT-GAAAACGTAC-TATG--T-TGT-GGTTCGCT-GACAACTTGTTCA--T-C--TTT-TAT-ATAT-AATATGCG-CGCACTTGG----TTT-----TATATTCTGTGC--AAGC--ATATA-----TTTTTTTTTATGAC-CTCAGCTCAAGCAAGAGTACCC-GCTGAACTTAAGCATATCAATAAGCGGAGGAAAAGAAACTAACAAGGATTCCCCTAGTAACGGCGAGTGAAGAGGGAAAAGCTCAAATTTTAAATCTACC-TGGTTCTTC--CCAGGTCGAGTTGTAATTTGAAGAAGCGATATC-GG-TG-TTGAGGTCTGGTTTAAGTTCTTTGGAACAAGACATCAT-GG-AGGGTGAGAATCCCGTGCATGATCAGA-CC---AAGAT--AC-TAAGT---ATACGCTTTCCAAGAGTCGAGTTGTTTGGGAATGCAGCTCAAAATGGG-T-GGTAGAC-TTCACCTAAGGCTAAATATCAGCGAGAGACCGATAGCGAACAAGTACTGTG-AA-GGAAAGATGAAAAGAACTTTGAAAAGAGAGTTAAATAGTACGTGAAATTGTTGAAAGGGAAACGATTGAAGTCAGTCATGCCAG-TGAAAACTCAGTTTGTTGGT-AATCT-AGTTCAGGAAT--GGAGGCAGGGT---CAA---ACCGTCTCTCTTTTGGACGCGATTATTGGCAGATGGAATTTTTTT-CTGGTAGGTCAGCGTCGGTTTC-GGGGGTTGTAAA---------ATACTTGGGGGTAAAGTAGCTCTGCTTCGGGA---GAGTG-TTATAGACCTTGGGGGATGCAGCCCGCGGGATCGAGGATTGCAGCAAATGC-------------------CTTCA--GGC-TTGTCGCCTGATCTCTGG-ATGTTACCTTGCTTGTGACAACATT-CTTGTCACCGGTGAGTACTAATGCTCACTAGGTTAGAGCGATCAAAAA-TTTTGCTAAGGATGCTGACGTAATGGCTTTAAACGACCCGTCTTGAAACACGGACCAAGGAGTCTAACATATGTGCGAGctgattggatgatcataactatacttcctgttcctcctcctcctgtacgtcctagtatacaaatggacggaacaagtcgaggtgaagatgatttgactcacaaattgtccgacatcttgaaggccaatcaaaacgtaaaacgttatgaagctgatggtcatcccccacatgttgtaaacgaatttgaagcattgttacaggttcttaatgat---------------------------------------------------------------------------------------------------------tataa-tttca--------a-ttagtttattatcaa--ttaaaa--ttttatacttaa-attattatttgt-----atgcaaacagtttcattgtgcaacttatatggacaatgaaatggctggtcaaccgcaagcccttcagaaatctggtagacctttaaagtcaatacgtgcgcgtctcaagggtaaagaaggacgtttacgtggtaatctgatgggaaagcgtgtagatttctctgctcgtacagtaattacgggggatcctaacatttcagttgatgaagtcggagttccgaaaagcatagctcaaaatttgacttttccagaattggtgactcccttcaatattgactatcttcaaaaattagtagaaaatggcccttctacacatccaggggctaaatatgtaattagagatactggtgaaaggattgacctaaaacatatatcaggcatgactggtggattaagattacactacggttggaaagttgaacgtcatctcaatgatggtgacatcgttatattcaatcgtcagccatctttgcacaagatgtcgatgatgggacataaagttcgtgttatgccctattcgaccttccgtcttaatttatcagttacaacaccttataacgccgattttgacggtgacgaaatgaacatgcatgttccccaatcagttgaaactaaagcagaaatttcagaaatctgcatggttcctaaacaaattgtatctcctcaatctaataaacctgttatgggtattgtacaggatactttatgtgctgttagaaaatttacaaaaagagattgctttttatctaaagatttggtaatgaacatt

>Diversispora_spurca_FN547637

AGGAATCCCTAGTAAGCGTGAGTCATCAGCTCACG-TTGATTACGTCCCTGCCCTTTGTACACACCGCCCGTCGCTACTACCGATTGAATGGCTTAGTGAGACCTTTGGATTGGGGTTTAGGGATCGGAAAC---GAACCTTATTCTCCGAGAAGTCGGTCAAACTTGGTCATTTAGAGGAAGTAAAAGTCGTAACAAGGTTTCCGTAGGTGAACCTGCGGAAGGATCATT-AAAAAAT-ATTTA-TCCGAGAATTCGT--TTTTTCGT----T--------TTCTCGGA------TTAATTTGTATTCAAA-TCCCACTCTTT-------AT-AAAT-ATA---------TCAATTATATAAAAC--AAAATAAAAAAAAAGAAAACTTTCAACAACGGATCTCTTGGCTCTCGCATCGATGAAGAACGCAGCGAAATGCGATACGTAGTGTGAATTGCAGAATTCCGTGAATCATCGAA-TCTTTGAATGCAAATTGTACTTTCCAGTAATCTGGGAAGTATGCTTGGTTGAGGGTCATCGAAATAACA-TTCGTGA----------------TTTTTCG-------------CGGATTTGAG----TTTT-CC--AGCA--TTC-ATTA----AT------A--------TG-TAAATGTTGGTAACTTT-AAAATT-ATTT------ATTACTTGGTGTAAGTT-GAAAACGTAC-TATA--T-TGT-GGTTCGCT-GACAACTTGTCCA--T-C--TT----T-ATAT-ATTATGTG-CGCACTTAT-------T----TTAATTCTGTGC--GAGCATATATA------TTTTTTTTTGACC-TTCAGCTCAAGCAAGAGTACCC-GCTGAACTTAAGCATATCAATAAGCGGAGGAAAAGAAACTAACAAGGATTCCCCTAGTAACGGCGAGTGAAGAGGGAAAAGCTCAAATTTTAAATCTACC-TGG---TTC--CCAGGTCGAGTTGTAATTTGAAGAAGCGATATC-GG-TG-TTGAGGTCTGGTTTAAGTTCTTTGGAACAAGACATCAT-GG-AGGGTGAGAATCCCGTGCATGATCAGA-CC---AAGAT--AC-TAAGT---ATACGCTTTCCAAGAGTCGAGTTGTTTGGGAATGCAGCTCAAAATGGG-T-GGTAGAC-TTCACCTAAGGCTAAATATCAGCGAGAGACCGATAGCGAACAAGTACTGTG-AA-GGAAAGATGAAAAGAACTTTGAAAAGAGAGTTAAATAGTACGTGAAATTGTTGAAAGGGAAACGATTGAAGTCAGTCATGCCAG-TGAAAACACAGTTTGGCGGATTTTTCAAGTTCAAGAGT--AAAGGCAGGGT---CAA---ACCGTCTCTCTTTTGGACTTGGAATTTGCCAGATGGAATTTTTCT-CTGGCAGGTCAGCGTCGGTTTCGGGGGGTTGTAAA---------ATACTTGGGGT-AAAGTAGCTCTGCTTTGGGA---GAGTG-TTATAGACCTTGGGGGATGCAGCCCGCGGGATCGAGGATTGCAGCAAATGC-------------------TTTCG--GGC-TTGTCGCCTGATCTCTGG-ATGTTACCTTGCTTGTGACAACATT-CTTGCCACCTGCGAGTACTAATGCTCATTAGGTTAGAGCGATCAAAAA-TTTTGCTAAGGATGCTGACGTAATGGCTTTAAACGACCCGTCTTGAAACACGGACCAAGGAGTCTAACATATGTGCGAG----------------------------------------------------------------------------------------------------------------------------------------------------------------------------------------------------------------------------------------------------------------------------------------------------------------------------------------------------------------------------------------------------------------------------------------------------------------------------------------------------------------------------------------------------------------------------------------------------------------------------------------------------------------------------------------------------------------------------------------------------------------------------------------------------------------------------------------------------------------------------------------------------------------------------------------------------------------------------------------------------------------------------------------------------------------------------------------------------------------------------------------------------------------------------------------------------------

>Diversispora_spurca_FN547644

AGGAATCCCTAGTAAGCGTGAGTCATCAGCTCACG-TTGATTACGTCCCTGCCCTTTGTACACACCGCCCGTCGCTACTACCGATTGAATGGCTTAGTGAGACCTTTGGATTGGGGTTTAGGGATCGGAAAC---GAACCTTATTCTCCGAGAAGTCGGTCAAACTTGGTCATTTAGAGGAAGTAAAAGTCGTAACAAGGTTTCCGTAGGTGAACCTGCGGAAGGATCATT-AAAAAAT-ATTTA-TCCGAGAATTCGT--TTTTTCGT----T--------TTCTCGGA------TTAATTTGTATTCAAA-TCCCACTCTTT-------AT-AAAT-ATA---------TCAATTATATAAAAC--AAAATAAAAAAAAAGAAAACTTTCAACAACGGATCTCTTGGCTCTCGCATCGATGAAGAACGCAGTGAAATGCGATACGTAGTGTGAATTGCAGAATTCCGTGAATCATCGAA-TCTTTGAACGCAAATTGTACTTTCCAGTAATCTGGGAAGTATGCTTGGTTGAGGGTCATCGAAATAACA-TTCGTGA----------------TTTTTCG-------------CGGATTTGAG----TTTT-CC--AGCA--TTC-ATTA----AT------A--------TG-TAAATGTTGGTAACTTT-AAAATT-ATTT------ATTACTTGGTGTAAGTT-GAAAACGTAC-TATA--T-TGT-GGTTCGCT-GACAACTTGTCCA--T-C--TT----T-ATAT-ATTATGCG-CGCACTTAT-------T----TTAATTCTGTGC--AAGC--ATATA------TTTTTTTTTTGAC-CTCAGCTCAAGCAAGAGTACCC-GCTGAACTTAAGCATATCAATAAGCGGAGGAAAAGAAACTAACAAGGATTCCCCTAGTAACGGCGAGTGAAGAGGGAAAAGCTCAAATTTTAAATCTACC-TGG---TTC--CCAGGTCGAGTTGTAATTTGAAGAAGCGATATC-GG-TG-TTGAGGTCTGGTTTAAGTTCTTTGGAACAAGACATCAT-GG-AGGGTGAGAATCCCGTGCATGATCAGA-CC---AAGAT--AC-TAAGT---ATACGCTTTCTAAGAGTCGAGTTGTTTGGGAATGCAGCTCAAAATGGG-T-GGTAGAC-TTCACCTAAGGCTAAATATCAGCGAGAGACCGATAGCGAACAAGTACTGTG-AA-GGAAAGATGAAAAGAACTTTGAAAAGAGAGTTAAATAGTACGTGAAATTGTTGAAAGGGAAACGATTGAAGTCAGTCATGCCAG-TGAAAACTCAGTTTGGCGGA-TTTTCAAGTTCAAGAGT--AAAGGTAGGGT---CAA---ACCGTCTCTCTTTTGGACTTGGAATTTGCCAGATGGAATTTTTCT-CTGACAGGTCAGCGTCGGTTTC-GGGGGTTGTAAA---------ATACTTGGGGT-AAAGTAGCTCTGCTTCGGGA---GAGTG-TTATAGACCTTGGGGGATGCAGCCCGCGGGATCGAGGATTGCAGCAAATGC-------------------TTTCG--GGC-TTGTCGCCTGATCTCTGG-ATGTTACCTTGCTTGTGACAACATT-CTTGCCACCTGCGAGTACTAATGCTCATTAGGTTAGAGCGATCAAAAA-TTTTGCTAAGGATGCTGACGTAATGGCTTTAAACGACCCGTCTTGAAACACGGACCAAGGAGTCTAACATGTGTGCGAG----------------------------------------------------------------------------------------------------------------------------------------------------------------------------------------------------------------------------------------------------------------------------------------------------------------------------------------------------------------------------------------------------------------------------------------------------------------------------------------------------------------------------------------------------------------------------------------------------------------------------------------------------------------------------------------------------------------------------------------------------------------------------------------------------------------------------------------------------------------------------------------------------------------------------------------------------------------------------------------------------------------------------------------------------------------------------------------------------------------------------------------------------------------------------------------------------------

>Diversispora_spurca_FN547639_OL690412

AGGAATCCCTAGTAAGCGTGAGTCATCAGCTCACG-TTGATTACGTCCCTGCCCTTTGTACACACCGCCCGTCGCTACTACCGATTGAATGGCTTAGTGAGACCTTTGGATTGGGGTTTAGGGATCGGAAAC---GAACCTTATTCTCCGAGAAGTCGGTCAAACTTGGTCATTTAGAGGAAGTAAAAGTCGTAACAAGGTTTCCGTAGGTGAACCTGCGGAAGGATCATT-AAAAAAT-ATTTA-TCCGAGAATTCG-----TTTCGT----T--------TTCTTGGA------TTAATTTGTATTCAAA-TCCCACTCTTT-------AT-AAAT-ATA---------TCAATTATATAAAAC--AAAA-ATAAAAAAAGAAAACTTTCAACAACGGATCTCTTGGCTCTCGCATCGATGAAGAACGCAGCGAAATGCGATACGTAGTGTGAATTGCAGAATTCCGTGAATCATCGAATTTTTTGAACGCAAATTGTACTTTTCAGTAATCTGGAAAGTATGCTTGGTTGAGGGTCATCGAAATAACA-TTCGTGA---------------TTTTTTCG-------------CGGATTTGAG----TTTT-CC--AGCA--TTT-ATTA------------A--------TA-TAAATGTAGGTAACTTT-AAAATT-ATTT------ATTAATTGGTGCAAGTT-GAAAACGTAC-TATG--T-TGT-GGTTCGCT-GACAACTTGTCCA--T-C--CT----T-ATAT-ATTATGTG-CGCACTTAT-------T----TTAATTCTGTGC--GAGC--ATATA------TTTTTTTTTTGAC-CTCAGCTCAAGCAAGAGTACCC-GCTGAACTTAAGCATATCAATAAGCGGAGGAAAAGAAACTAACAAGGATTCCCCTAGTAACGGCGAGTGAAGAGGGAAAAGCTCAAATTTTAAATCTACC-TGG---TTC--CCAGGTCGAGTTGTAATTTGAAGAAGCGATATC-GG-TG-TTGAGGTCTGGTTTAAGTTCTTTGGAACAAGACATCAT-GG-AGGGTGAGAATCCCGTGCATGATCAGA-CC---AAGAT--AC-TAAGT---ATACGCTTTCTAAGAGTCGAGTTGTTTGGGAATGCAGCTCAAAATGGG-T-GGTAGAC-TTCACCTAAGGCTAAATATCAGCGAGAGACCGATAGCGAACAAGTACTGTG-AA-GGAAAGATGAAAAGAACTTTGAAAAGAGAGTTAAATAGTACGTGAAATTGTTGAAAGGGAAACGATTGAAGTCAGTCATGCCAG-TGAAAACTCAGTTTGGCGGA-TTTTCAAGTTCAAGAGT--AAAGGTAGGGT---CAA---ACCGTCTCTCTTTTGGACTTGGAATTTGCCAGATGGAATTTTTCT-CTGACAGGTCAGCGTCGGTTTC-GGGGGTTGTAAA---------ATACTTGGGGT-AAAGTAGCTCTGCTTCGGGA---GAGTG-TTATAGACCTTGGGGGATGCAGCCCGCGGGATCGAGGATTGCAGCAAATGC-------------------TTTCG--GGC-TTGTCGCCTGATCTCTGG-ATGTTACCTTGCTTGTGACAACATT-CTTGCCACCTGCGAGTACTAATGCTCATTAGGTTAGAGCGATCAAAAA-TTTTGCTAAGGATGCTGACGTAATGGCTTTAAACGACCCGTCTTGAAACACGGACCAAGGAGTCTAACATATGTGCGAGctgattggatgatcataactatacttcctgttcctcctcctcctgtacgtcctagtatacaagtggacggaacaagtcgaggtgaagatgatttgactcacaaattgtccgacatcttgaaggccaatcaaaacgtaaaacgttatgaagctgatggtcatcccccacatgttgtaaacgaatttgaagcattgttacaggttcttaatgat---------------------------------------------------------------------------------------------------------tataa-tttca--------a-ttagtttattatcaa--ttaaaa--ttttatacttaa-attattatttgt-----atgcaaacagtttcattgtgcaacttatatggacaatgaaatggctggtcaaccgcaagcccttcagaaatctggtagacctttaaagtcaatacgtgcgcgtctcaagggtaaagaaggacgtttacgtggtaatctgatgggaaagcgtgtagatttctctgctcgtacagtaattacgggggatcctaatatttcagttgatgaagtcggagttccgaaaagcatagctcaaaatttgacttttccagaattggtgactcccttcaatattgactatcttcaaaaattagtagaaaatggcccttctacacatccaggggctaaatatgtaattagagatactggtgaaaggattgacctaaaacatatatcaggcatgactggtggattaagattacactacggttggaaagttgaacgtcatctcaatgatggtgacatcgttatattcaatcgtcagccatctttgcacaagatgtcgatgatgggacataaagttcgtgttatgccctattcgaccttccgtcttaatttatcagttacaacaccttataacgccgattttgacggtgacgaaatgaacatgcatgttccccaatcagttgaaactaaagcagaaatttcagaaatctgcatggttcctaaacaaattgtatctcctcaatctaataaacccgttatgggtattgtacaggatactttatgtgctgttagaaaatttacaaaaagagattgctttttatctaaagatttggtaatgaacatt

>Diversispora_sabulosa_MG459211_MG459182

AGGAATCCCTAGTAAGCGTGAGTCATCAGCTCACG-TTGATTACGTCCCTGCCCTTTGTACACACCGCCCGTCGCTACTACCGATTGAATGGCTTAGTGAGACCTTTGGATTGGGGTTTAGGGATCGGAAAC---GATTCTTTTTCTTCGAGAAGTCGGTCAAACTTGGTCATTTAGAGGAAGTAAAAGTCGTAACAAGGTTTCCGTAGGTGAACCTGCGGAAGGATCATT-AAAAAATCATTTA-TCCGAGAATTCA-----TTTCGT----T--------TTCTCGGA------TTAATTTGTATTCAAA-TTCCACTCTTTAA-----AT-AAAT-ATA---------TTAATTATATAAAAT---AAA-T-AAAAAAAGAAAACTTTCAACAACGGATCTCTTGGCTCTCGCATCGATGAAGAACGCAGCGAAATGCGATACGTAATGTGAATTGCAGAATTCCGTGAATCATCGAA-TCTTCGAACGCAAATTGCACTTTCCAGTAATCTGGGAAGTATGCTTGGTTGAGGGTCATTAAAATAACA-TTCGTGAA-------------TTTTTTTCG-------------CGGATTTGAG----TTTT-CC--AGTA--TTT-ATAAT-TTAT------A--------TATAAATGGTTGGTAACTTT-AAAATT-ATC-------ATCACTTGGTATAAGTC-GAAAACGTAC-TATA--TGTGT-GGTTCGTT-GACAACTTGTCCA--T-C--TT----T-ACAT-ACTATGCA-TGCACTTGG----GTTT----TGTACTTTGTGT--AAGC--ATATA-----TTTTTTTTTTTGAC-CTCAACTCAAGCAAGAATACCC-GCTGAACTTAAGCATATCAATAAGCGGAGGAAAAGAAACTAACAAGGATTCCCCTAGTAACGGCGAGTGAAGAGGGAAAAGCTCAAATTTTAAATCTACC-TGG---TTC--CCAGGTCGAATTGTAATTTGAAGAAGCGATATC-GG-TG-TTGAGGTCTGGTTTAAGTTCTTTGGAACAAGATATCAT-GGAAGGGTGAGAATCCCGTGCATGATCAGG-CC---AAGATGCAC-TTAAT---ATTCGTTTTCAAAGAGTCGAGTTGTTTGGGAATGCAGCTCAAAATGGG-T-GGTAGAC-TTCACCTAAGGCTAAATATCAGCGAGAGACCGATAGCGAACAAGTACTGTG-AA-GGAAAGATGAAAAGAACTTTGAAAAGAGAGTTAAATAGTACGTGAAATTGTTGAAAGGGAAACGATTGAAGTCAGTCATGCCAG-TGAAAATTCAGTTTGGCGGG-TTTCT-GGTCTTTTGAT--AGAGGCAG--------------CGTTTCTTTTTTGGAC-CAGGATCTGTCAGATGCACTTTTTCT-TTGGCAGGTCAGCGTCGATTTC-GGGGGTTGTAAA---------ATACTTGGGGA-AAAGTAGCTCTGCTTCGGGA---GAGTG-TTATAGACCTTGGGGGATGCAGCCCGCGAGATCGAGGATTGCAGCAAATGC-------------------CTTTT--GGC-TTGTCGCCTGATCTCTGG-ATGTTACCTCGCTTGTAACAACATT-CTTGTTGCCGGTGAGTACTAATGCCTATCAGGTTAGAGCGATCAAAAA--TTTGCTAAGGATGCTGACGTAATGGCTTTAAACGACCCGTCTTGAAACACGGACCAAGGAGTCTAACATATGTGCGAG------------------------------------------------------------------------------gtggagaagatgatttgacacacaaattgtccgacatcttgaaagcaaatcaaaatgtaaaacgttatgaagctgatggtcatcctccacacgttgtaaacgaatttgaagcattgttacaggttcttaatgat---------------------------------------------------------------------------------------------------------tataattttta--------a-ttagtttaatatcaa--ttgaaa--tctatacttaaa-tttattatttat-----atacaaacagtttcattgtgcaacttatatggacaatgaaatggctggtcaacctcaagctcttcagaaatctggtagacctttaaagtcaatacgtgcgcgtctcaagggtaaagaagggcgtttacgtggtaatctgatgggaaaacgtgtagatttctctgctcgtacagtaattacgggtgatccaaatatttcagtcgatgaagtcggagttccgaaaagcatagctcaaaatttgacttttccggaattggtaactccctttaatattgactatcttcaaaaattagtagaaaatggcccttctacacatccaggggctaaatacgtaattagagatactggtgaaaggattgatctaaaacatatatcaggcatgactggtggattaagattacactacggttggaaggttgaacgtcatcttaatgatggtgacatcgttatattcaatcgtcagccatctttgcacaaaatgtcaatgatggg-------------------------------------------------------------------------------------------------------------------------------------------------------------------------------------------------------------------------------------------------------------------------------------

>Diversispora_sabulosa_MG459212

AGGAATCCCTAGTAAGCGTGAGTCATCAGCTCACG-TTGATTACGTCCCTGCCCTTTGTACACACCGCCCGTCGCTACTACCGATTGAATGGCTTAGTGAGACCTTTGGATTGGGGTTTAGGGATCGGAAAC---GATTCTTTTTCTTCGAGAAGTCGGTCAAACTTGGTCATTTAGAGGAAGTAAAAGTCGTAACAAGGTTTCCGTAGGTGAACCTGCGGAAGGATCATT-AAAAAATCATTTA-TCCGAGAATTCA-----TTTCGT----T--------TTCTCGGA------TTAATTTGTATTCAAA-TTCCACTCTTTAA-----AT-AAAT-ATA---------TTAATTATATAAAAT---AAA-T-AAAAAAAGAAAACTTTCAACAACGGATCTCTTGGCTCTCGCATCGATGAAGAACGCAGCGAAATGCGATACGTAATGTGAATTGCAGAATTCCGTGAATCATCGAA-TCTTCGAACGCAAATTGCACTTTCCAGTAATCTGGGAAGTATGCTTGGTTGAGGGTCATTAAAATAACA-TTCGTGAA-------------TTTTTTTCG-------------CGGATTTGAG----TTTT-CC--AGTA--TTT-ATAAT-TTAT------A--------TATAAATGGTTGGTAACTTT-AAAATT-ATC-------ATCACTTGGTATAAGTC-GAAAACGTAC-TATA--TGTGT-GGTTCGTT-GACAACTTGTCCA--T-C--TT----T-ACAT-ACTATGCA-TGCACTTGG----GTTT----TGTACTTTGTGT--AAGC--ATATA-----TTTTTTTTTTTGAC-CTCAACTCAAGCAAGAATACCC-GCTGAACTTAAGCATATCAATAAGCGGAGGAAAAGAAACTAACAAGGATTCCCCTAGTAACGGCGAGTGAAGAGGGAAAAGCTCAAATTTTAAATCTACC-TGG---TTC--CCAGGTCGAATTGTAATTTGAAGAAGCGATATC-GG-TG-TTGAGGTCTGGTTTAAGTTCTTTGGAACAAGATATCAT-GG-AGGGTGAGAATCCCGTGCATGATCAGG-CC---AAGATGCAC-TTAAT---ATTCGTTTTCAAAGAGTCGAGTTGTTTGGGAATGCAGCTCAAAATGGG-T-GGTAGAC-TTCACCTAAGGCTAAATATCAGCGAGAGACCGATAGCGAACAAGTACTGTG-AA-GGAAAGATGAAAAGAACTTTGAAAAGAGAGTTAAATAGTACGTGAAATTGTTGAAAGGGAAACGATTGAAGTCAGTCATGCCAG-TGAAAATTCAGTTTGGCGGG-TTTCT-GGTCTTTTGAT--AGAGGCAG--------------CGTTTCTTTTTTGGAC-CAGGATCTGTCAGATGCACTTTTTCT-TTGGCAGGTCAGCGTCGATTTC-GGGGGTTGTAAA---------ATACTTGGGGA-AAAGTAGCTCTGCTTCGGGA---GAGTG-TTATAGACCTTGGGGGATGCAGCCCGCGAGATCGAGGATTGCAGCAAATGC-------------------CTTTT--GGC-TTGTCGCCTGATCTCTGG-ATGTTACCTCGCTTGTAACAACATT-CTTGTTGCCGGTGAGTACTAATGCCTATCAGGTTAGAGCGATCAAAAA--TTTGCTAAGGATGCTGACGTAATGGCTTTAAACGACCCGTCTTGAAACACGGACCAAGGAGTCTAACATATGTGCGAG----------------------------------------------------------------------------------------------------------------------------------------------------------------------------------------------------------------------------------------------------------------------------------------------------------------------------------------------------------------------------------------------------------------------------------------------------------------------------------------------------------------------------------------------------------------------------------------------------------------------------------------------------------------------------------------------------------------------------------------------------------------------------------------------------------------------------------------------------------------------------------------------------------------------------------------------------------------------------------------------------------------------------------------------------------------------------------------------------------------------------------------------------------------------------------------------------------

>Diversispora_sabulosa_MG459215

AGGAATCCCTAGTAAGCGTGAGTCATCAGCTCACG-TTGATTACGTCCCTGCCCTTTGTACACACCGCCCGTCGCTACTACCGATTGAATGGCTTAGTGAGACCTTTGGATTGGGGTTTAGGGATCGGAAAC---GATTCTTTTTCTTCGAGAAGTCGGTCAAACTTGGTCATTTAGAGGAAGTAAAAGTCGTAACAAGGTTTCCGTAGGTGAACCTGCGGAAGGATCATT-AAAAAATCATTTA-TCCGAGAATTCA-----TTTCGT----T--------TTCTCGGA------TTAATTTGTATTCAAA-TTTCACTCTTTAA-----AT-AAAT-ATA---------TTAATTATATAAAAT---AAA-T-AAAAAAAGAAAACTTTCAACAACGGATCTCTTGGCTCTCGCATCGATGAAGAACGCAGCGAAATGCGATACGTAATGTGAATTGCAGAATTCCGTGAATCATCGAA-TCTTCGAACGCAAATTGCACTTTCCAGTAATCTGGGAAGTATGCTTGGTTGAGGGTCATTAAAATAACA-TTCGTGAA-------------TTTTTTTCG-------------CGGATTTGAG----TTTT-CC--AGTA--TTT-ATA----TAT------A--------TA-TAAATGTTGGTAACTTC-AAAATT-ATTATT----ATCACTTGGTACAAGTC-GAAAACGTAC-TATA--TGTGT-GGTTCGTT-GACAACTTGTCCA--T-C--TT----T-ACAT-ACTATGCG-TGCACTTGG-----TTT----TGTACTTTGTGT--AAGC--ATATA-------TTTTTTTTTGAC-CTCAACTCAAGCAAGAATACCC-GCTGAACTTAAGCATATCAATAAGCGGAGGAAAAGAAACTAACAAGGATTCCCCTAGTAACGGCGAGTGAAGAGGGAAAAGCTCGAATTTTAAATCTACC-TGG---TTC--CCAGGTCGAATTGTAATTTGAAGAAGCGATATC-GG-TG-TTGAGGTCTGGTTTAAGTTCTTTGGAACAAGATATCATGGG-AGGGTGAGAATCCCGTGCATGATCAGG-CC---AAGATGCAC-TTAAT---ATTCGTTTTCAAAGAGTCGAGTTGTTTGGGAATGCAGCTCAAAATGGG-T-GGTAGAC-TTCACCTAAGGCTAAATATCAGCGAGAGACCGATAGCGAACAAGTACTGTG-AA-GGAAAGATGAAAAGAACTTTGAAAAGAGAGTTAAATAGTACGTGAAATTGTTGAAAGGGAAACGATTGAAGTCAGTCATGCCAG-TGAAAATTCAGTTTGGCGGG-TTTTT-GGTCTTTTGAT--AGAGGCAGCG----------AGCGTTTCTTTTTTTGGACCAGGATCTGTCAGATGCACTTTTTCT-TTGGCAGGTCAGCGTCGATTTC-GGGGGTTGTAAA---------ATACTCGGGGA-AAAGTAGCTCTGCTTCGGGA---GAGTG-TTATAGACCTTGGGGGATGCAGCCCGCGGGATCGAGGATTGCAGCAAATGC-------------------CTTTA--GGC-TTGTCGCCTGATCTCTGG-ATGTTACCTCGCTTGTAACAACATT-CTTGTTGCCGGTGAGTACTAATGCCTATCAGGTTAGAGCGATCAAAAA--TTTGCTAAGGATGCTGACGTAATGGCTTTAAACGACCCGTCTTGAAACACGGACCAAGGAGTCTAACATATGTGCGAG----------------------------------------------------------------------------------------------------------------------------------------------------------------------------------------------------------------------------------------------------------------------------------------------------------------------------------------------------------------------------------------------------------------------------------------------------------------------------------------------------------------------------------------------------------------------------------------------------------------------------------------------------------------------------------------------------------------------------------------------------------------------------------------------------------------------------------------------------------------------------------------------------------------------------------------------------------------------------------------------------------------------------------------------------------------------------------------------------------------------------------------------------------------------------------------------------------

>Diversispora_sabulosa_MG459214

AGGAATCCCTAGTAAGCGTGAGTCATCAGCTCACG-TTGATTACGTCCCTGCCCTTTGTACACACCGCCCGTCGCTACTACCGATTGAATGGCTTAGTGAGACCTTTGGATTGGGGTTTAGGGATCGGAAAC---GATTCTTTTTCTTCGAGAAGTCGGTCAAACTTGGTCATTTAGAGGAAGTAAAAGTCGTAACAAGGTTTCCGTAGGTGAACCTGCGGAAGGATCATT-AAAAAATCATTTA-TCCGAGAATTCA-----TTTCGT----T--------TTCTCGGA------TTAATTTGTATTCAAA-TTTCACTCTTTAA-----AT-AAAT-ATA---------TTAATTATATAAAAT---AAA-T-AAAAAAAGAAAACTTTCAACAACGGATCTCTTGGCTCTCGCATCGATGAAGAACGCAGCGAAATGCGATACGTAATGTGAATTGCAGAATTCCGTGAATCATCGAA-TCTTCGAACGCAAATTGCACTTTCCAGTAATCTGGGAAGTATGCTTGGTTGAGGGTCATTAAAATAACA-TTCGTGAA-------------TTTTTTTCG-------------CGGATTTGAG----TTTT-CC--AGTA--TTT-ATA----TAT------A--------TA-TAAATGTTGGTAACTTC-AAAATT-ATTATT----ATCACTTGGTACAAGTC-GAAAACGTAC-TATA--TGTGT-GGTTCGTT-GACAACTTGTCCA--T-C--TT----T-ACAT-ACTATGCG-TGCACTTGG-----TTT----TGTACTTTGTGT--AAGC--ATATA-------TTTTTTTTTGAC-CTCAACTCAAGCAAGAATACCC-GCTGAACTTAAGCATATCAATAAGCGGAGGAAAAGAAACTAACAAGGATTCCCCTAGTAACGGCGAGTGAAGAGGGAAAAGCTCGAATTTTAAATCTACC-TGG---TTC--CCAGGTCGAATTGTAATTTGAAGAAGCGATATC-GG-TG-TTGAGGTCTGGTTTAAGTTCTTTGGAACAAGATATCAT-GG-AGGGTGAGAATCCCGTGCATGATCAGG-CC---AAGATGCAC-TTAAT---ATTCGTTTTCAAAGAGTCGAGTTGTTTGGGAATGCAGCTCAAAATGGG-T-GGTAGAC-TTCACCTAAGGCTAAATATCAGCGAGAGACCGATAGCGAACAAGTACTGTG-AA-GGAAAGATGAAAAGAACTTTGAAAAGAGAGTTAAATAGTACGTGAAATTGTTGAAAGGGAAACGATTGAAGTCAGTCATGCCAG-TGAAAATTCAGTTTGGCGGG-TTTTT-GGTCTTTTGAT--AGAGGCAGCG----------AGCGTTTCTTTTTTTGGACCAGGATCTGTCAGATGCACTTTTTCT-TTGGCAGGTCAGCGTCGATTTC-GGGGGTTGTAAA---------ATACTCGGGGA-AAAGTAGCTCTGCTTCGGGA---GAGTG-TTATAGACCTTGGGGGATGCAGCCCGCGGGATCGAGGATTGCAGCAAATGC-------------------CTTTA--GGC-TTGTCGCCTGATCTCTGG-ATGTTACCTCGCTTGTAACAACATT-CTTGTTGCCGGTGAGTACTAATGCCTATCAGGTTAGAGCGATCAAAAA--TTTGCTAAGGATGCTGACGTAATGGCTTTAAACGACCCGTCTTGAAACACGGACCAAGGAGTCTAACATATGTGCGAG----------------------------------------------------------------------------------------------------------------------------------------------------------------------------------------------------------------------------------------------------------------------------------------------------------------------------------------------------------------------------------------------------------------------------------------------------------------------------------------------------------------------------------------------------------------------------------------------------------------------------------------------------------------------------------------------------------------------------------------------------------------------------------------------------------------------------------------------------------------------------------------------------------------------------------------------------------------------------------------------------------------------------------------------------------------------------------------------------------------------------------------------------------------------------------------------------------

>Diversispora_sabulosa_MG459213_MG459183

AGGAATCCCTAGTAAGCGTGAGTCATCAGCTCACG-TTGATTACGTCCCTGCCCTTTGTACACACCGCCCGTCGCTACTACCGATTGAATGGCTTAGTGAGACCTTTGGATTGGGGTTTAGGGATCGGAAAC---GATTCTTTTTCTTCGAGAAGTCGGTCAAACTTGGTCATTTAGAGGAAGTAAAAGTCGTAACAAGGTTTCCGTAGGTGAACCTGCGGAAGGATCATT-AAAAAATCATTTA-TCCGAGAATTCA-----TTTCGT----T--------TTCTCGGA------TTAATTTGTATTCAAA-TTTCACTCTTTAA-----AT-AAAT-ATA---------TTAATTATATAAAAT---AAA-T-AAAAAAAGAAAACTTTCAACAACGGATCTCTTGGCTCTCGCATCGATGAAGAACGCAGCGAAATGCGATACGTAATGTGAATTGCAGAATTCCGTGAATCATCGAA-TCTTCGAACGCAAATTGCACTTTCCAGTAATCTGGGAAGTATGCTTGGTTGAGGGTCATTAAAATAACA-TTCGTGAA-------------TTTTTTTCG-------------CGGATTTGAG----TTTT-CC--AGTA--TTT-ATA----TAT------A--------TA-TAAATGTTGGTAACTTC-AAAATT-ATTATT----ATCACTTGGTACAAGTC-GAAAACGTAC-TATA--TGTGT-GGTTCGTT-GACAACTTGTCCA--T-C--TT----T-ACAT-ACTATGCG-TGCACTTGG-----TTT----TGTACTTTGTGT--AAGC--ATATA-------TTTTTTTTTGAC-CTCAACTCAAGCAAGAATACCC-GCTGAACTTAAGCATATCAATAAGCGGAGGAAAAGAAACTAACAAGGATTCCCCTAGTAACGGCGAGTGAAGAGGGAAAAGCTCAAATTTTAAATCTACC-TGG---TTC--CCAGGTCGAATTGTAATTTGAAGAAGCGATATC-GG-TG-TTGAGGTCTGGTTTAAGTTCTTTGGAACAAGATATCAT-GG-AGGGTGAGAATCCCGTGCATGATCAGG-CC---AAGATGCAC-TTAAT---ATTCGTTTTCAAAGAGTCGAGTTGTTTGGGAATGCAGCTCAAAATGGG-T-GGTAGAC-TTCACCTAAGGCTAAATATCAGCGAGAGACCGATAGCGAACAAGTACTGTG-AA-GGAAAGATGAAAAGAACTTTGAAAAGAGAGTTAAATAGTACGTGAAATTGTTGAAAGGGAAACGATTGAAGTCAGTCATGCCAG-TGAAAATTCAGTTTGGCGGG-TTTTT-GGTCTTTTGAT--AGAGGCAGCG----------AGCGTTTCTTTTTTTGGACCAGGATCTGTCAGATGCACTTTTTCT-TTGGCAGGTCAGCGTCGATTTC-GGGGGTTGTAAA---------ATACTCGGGGA-AAAGTAGCTCTGCTTCGGGA---GAGTG-TTATAGACCTTGGGGGATGCAGCCCGCGGGATCGAGGATTGCAGCAAATGC-------------------CTTTA--GGC-TTGTCGCCTGATCTCTGG-ATGTTACCTCGCTTGTAACAACATT-CTTGTTGCCGGTGAGTACTAATGCCTATCAGGTTAGAGCGATCAAAAA--TTTGCTAAGGATGCTGACGTAATGGCTTTAAACGACCCGTCTTGAAACACGGACCAAGGAGTCTAACATATGTGCGAG------------------------------------------------------------------------------gtggagaagatgatttgacacacaaattgtccgacatcttgaaagcaaatcaaaatgtaaaacgttatgaagctgatggtcatcctccacacgttgtaaacgaatttgaagcattgttacaggttcttaatgat---------------------------------------------------------------------------------------------------------tataattttta--------a-ttagtttaatatcaa--ttgaaa--tctatacttaaa-tttattatttat-----atacaaacagtttcattgtgcaacttatatggacaatgaaatggctggtcaacctcaagctcttcagaaatctggtagacctttaaagtcaatacgtgcgcgtctcaagggtaaagaagggcgtttacgtggtaatctgatgggaaaacgtgtagatttctctgctcgtacagtaattacgggtgatccaaatatttcagtcgatgaagtcggagttccgaaaagcatagctcaaaatttgacttttccggaattggtaactccctttaatattgactatcttcaaaaattagtagaaaatggcccttctacacatccaggggctaaatacgtaattagagatactggtgaaaggattgatctaaaacatatatcaggcatgactggtggattaagattacactacggttggaaggttgaacgtcatcttaatgatggtgacatcgttatattcaatcgtcagccatctttgcataaaatgtcaatgatggggt-----------------------------------------------------------------------------------------------------------------------------------------------------------------------------------------------------------------------------------------------------------------------------------

>Diversispora_valentina_MT985516

-------------------------------------------------------------------------------------------------------------------GTTTAGGGATCGGAAAC---GATTCTTATTCTCTGAAAAGTCGGTCAAACTTGGTCATTTAGAGGAAGTAAAAGTCGTAACAAGGTTTCCGTAGGTGAACCTGCGGAAGGATCATT--AAAAAC-TTTTA-TCCGGGAATTCG-----TTTCGT----C--------TTCCCG-G------ATTATTTGTATTCAAA-TCCCACTCTTT-------AT-AAAT-ATA---------TTAATTATATAAAAC--AATA-T-AAAAAAAGAAAACTTTCAACAACGGATCTCTTGGCTCTCGCATCGATGAAGAACGCAGCGAAATGCGATACGTAGTGTGAATTGCAGAATTCCGTGAATCATCGAA-TCTTTGAACGCAAATTGCACTCCCCAGTAATCTGGGAAGTATGCTTGGTTGAGGGTCATCAAAATAACC-TTCGTGAA---------------TTTTTCG-------------CGGATTTGAG----TTTT-CC--AGTA--TTT-ACTTA-TTAT------A--------AA-TAAATGTTGGTGACTTT-AAAATT-ATTT-------TTACTTGATACAAGTT-GAAAACGTGC-TATA--TGTGT-GGTTCGCT-GACAACTTGTTCA--T-C--AT----T-ATAT-ATTATGTG-CGCACTTAG----TTTT----TATACTCTGTAC--GAAC--ATATA------TTTTTTTTTTGAC-CTCAGCTCAAGCAAGGAAACCC-GCTGAACTTAAGCATATCAATAAGCGGAGGAAAAGAAACTAACAAGGATTCTCCTAGTAACGGCGAGTGAAGAGGGAAAAGCTCAAATTTTAAATCTACC-TGG---TTC--CCAGGTCGAATTGTAATTTGAAGAAGCGATATC-GG-TG-TTGAGGTCTGGTTCAAGTTCTTTGGAACAAGACATCAT-GG-AGGGTGAGAATCCCGTGCATGATCAGG-CC---AAGAT--AC-TTAAT---ATTCGTTCTCTAAGAGTCGAGTTGTTTGGGAATGCAGCTCAAAATGGG-T-GGTAGAC-TTCACCTAAGGCTAAATATCAGCGAGAGACCGATAGCGAACAAGTACTGTG-AA-GGAAAGATGAAAAGAACTTTGAAAAGAGAGTTAAATAGTACGTGAAATTGTTGAAAGGGAAACGATTGAAGTCAGTCATGCTAG-TGAAAATTCAGTTTGGCGGGTTTTTG-AGTTTAGGAGT--AGAAGCAGGGT---CAA---ACCGTTTCTCTTTTGAACTTGGGATTTGTCAGATGCACTTTTTCT-TTGGCAGGTCAGCGTCGATTTC-GGAGGTTGTAAA---------ATACTTGGGGG-AAAGTAGCTCTGCTTCGGGA---GAGTG-TTATAGACCCTGGGGGATGCAGCCTGCGGGACCGAGGATTGCAGCAAATGC-------------------TTTTT--GGC-TTGTCGCCTGATCTCTGG-ACGTTACCCTGCTTGTGACAACATT-TTTGTCACCGGTGGGTACTAATGGCTATTAGGTTAGAGCGATCAAAAA--TTTGCTAA--------------------------------------------------------------------------------------------------------------------------------------------------------------------------------------------------------------------------------------------------------------------------------------------------------------------------------------------------------------------------------------------------------------------------------------------------------------------------------------------------------------------------------------------------------------------------------------------------------------------------------------------------------------------------------------------------------------------------------------------------------------------------------------------------------------------------------------------------------------------------------------------------------------------------------------------------------------------------------------------------------------------------------------------------------------------------------------------------------------------------------------------------------------------------------------------------------------------------------------------------------------------------------

>Diversispora_valentina_MT985515

-------------------------------------------------------------------------------------------------------------------GTTTAGGGATCGGCAAC---GATTCTTATTCTCTGAAAAGTCGGTCAAACTTGGTCATTTAGAGGAAGTAAAAGTCGTAACAAGGTTTCCGTAGGTGAACCTGCGGAAGGATCATTAAAAAAAT-CTTTA-TCCGGGAATTCA-----TTTCGT----C--------TTCCCG-G------ATTATTTGTATTCAAA-TCCCACTCTTT-------AT-AAAT-ATA---------TCAATTATATAAAAC--AATA-T-AAAAAAAGAAAACTTTCAACAACGGATCTCTTGGCTCTCGCATCGATGAAAAACGCAGCGAAACGCGATACGTAGTGTGAATTGCAGAATTCCGTGAATCATCGAA-TCTTTGAACGCAAATTGCACTTCCCAGTAATCTGGGAAGTATGCTTGGTTGAGGGTCATCAAAATAACC-TTCGTGAA---------------TTTTTCG-------------CGGATTTGAG----TTTT-CC--AGTA--TTT-ATT-------------------------TAAATGTTGGTAACTTT-AAAATT-ATTT-------TTACTTGATACAAGTT-GAAAACGTGC-TATA--TGTGT-GGTTCGCT-GACAACTTGTTCA--T-C--TT----T-ATAT-ATTATGTG-CGCACTTAG----TTTT----TATACTCTGTAC--GAACATATATA------TATTTTTTTTGAC-CTCACCTCACGCAAGGAAACCC-GCTGAACTTAAGCATATCAATAAGCGGAGGAGAAGAAACTAACAAGGATTCCCCTAGTAACGGCGAGTGAAGAGGGAAAAGCTCAAATTTTAAATCTACC-TGG---TTC--CCACGTCGAATTGTAATTTGAAGAAGCGATATC-GG-TG-TTGAGGTCTGGTTCAAGTTCTTTGGAACAAGACATCAT-GG-AGGGTGAGAATCCCGTGCATGATCAGA-CC---AAGAT--AC-TTAAT---ATTCGTTCTCTAAGAGTCGAGTTGTTTGGGAATGCAGCTCAAAATGGG-T-GGTAGAC-TTCACCTAAGGCTAAATATCAACGAGAGACCGATAGCGAACAAGTACTGTG-AA-GGAAAGATGAAAAGAACTTTGAAAAGAGAGTTAAAAAGTACGTGAAATTGTTGAAAGGGAAACGATTGAAGTCAGTCACGCTAG-TGAAAATTCAGTTTGGCGGATTTTTG-AGTTTAGGAGT--AGAAGCAGGGT---CAA---ACCGTTTCTCTTTTGAACTTGGGATTTGTCAGATGCACTTTTTCT-TTGGCAGGTCAGCGTCGATTTC-GGAGGTTGTAAA---------ATACTTGGGGG-AAAGTAGCTCTGCTTCGGGA---GAGTG-CTATAGACCCTGGGGGATGCAGCCTGCGGGACCGAGGATTGCAGCAAATGC-------------------TTTTT--GGC-TTGTCGCCTGATCTCTGG-ACGTTACCCTGCTTGTGACAACATT-TTTGTCACCGGTGGGTACTAATGGCTATTGGGTTAGAGCGATCAAAA---TTTGCTAA--------------------------------------------------------------------------------------------------------------------------------------------------------------------------------------------------------------------------------------------------------------------------------------------------------------------------------------------------------------------------------------------------------------------------------------------------------------------------------------------------------------------------------------------------------------------------------------------------------------------------------------------------------------------------------------------------------------------------------------------------------------------------------------------------------------------------------------------------------------------------------------------------------------------------------------------------------------------------------------------------------------------------------------------------------------------------------------------------------------------------------------------------------------------------------------------------------------------------------------------------------------------------------

>Diversispora_gibbosa_KJ850201_MG459196

AGGAATCCCTAGTAAGCGTGAGTCATCAGCTCACG-TTGATTACGTCCCTGCCCTTTGTACACACCGCCCGTCGCTACTACCGATTGAATGGCTTAGTGAGACCTTTGGATTGGGGTTTTGGGATCGGCAAC---GATCCTATTTCACCGAGAAGTCGGTCAAACTTGGTCATTTAGAGGAAGTAAAAGTCGTAACAAGGTTTCCGTAGGTGAACCTGCGGAAGGATCATT-AAAAAAT-TTTTA-ACCGAGAATTCGT---TTTTCGT----T--------TTCTCG-G------ATAATTTGTATTCAAA-TTCCCACTCTT----------------AAATAATA--TATTATTATATAAACA-TATAT-A-AAAAAAAGAAAACTTTCAACAACGGATCTCTTGGCTCTCGCATCGATGAAGAACGCAGCGAAATGCGATACGTAGTGTGAATTGCAGAATTCCGTGAATCATCGAA-TTTTTGAACGCAAATTGTACTTTTCAGTATTCTGGAAAGTATGCTTGGTTGAGGGTCATTAAAATAACA-TTCGTGAA------------TTTTTTTTTG-------------CGGATTTGAG----TTTT-CC--AGTA--TTT--TATTTATTT---------------AA-TAAATGTTGGTAACTTT-AAAATT-ATTTTA----TTTTCTTGGTTAAAATT-AAAAACGTAC-TATA--TGTGT-GGTTCGTT-GATAATTTGTCCA--T-C--TA----T-ATAT-ATTATGTT-TATACTTGG----TCCACAT-TGGCCTTTGTGTGTGAAT--ATATA-----TTTTTTTTTATGAC-CTCAGCTCAAGCAAGAATACCC-GCTGAACTTAAGCATATCAATAAGCGGAGGAAAAGAAACTAACAAGGATTCCCCTAGTAACGGCGAGTGAAGAGGGAAAAGCTCAAATTTTAAATCTACC-TGGTT-CTT--CTAGGTCGAATTGTAATTTGAAGAAACGATATCTTA-TT-TTGAGGTCTGGTTTAAGTCTTTTGGAACAAGACATCAT-GGAAGGGTGAGAATCCCGTGCATGATCAGA-CC---GAAAT--TTATTAAT---ATTCGTTCTCTAAGAGTCGAGTTGTTTGGGAATGCAGCTCAAAATGGG-T-GGTAGAC-TTCACCTAAGGCTAAATATCAGCGAGAGACCGATAGCGAACAAGTACTGTG-AA-GGAAAGATGAAAAGAACTTTGAAAAGAGAGTTAAAAAGTACGTGAAATTGTTGAAAGGGAAACGATTGAAGTCAGTCATGCCAG-TGAAAAATCAGTTTAACGGA-TTTTT-GGTTCGGAGCA--GGAGGCAGGGT---CAA---ACCGTCTCTCTTTTGGACTTGGAATTTGTTAGATGTACTTTTTCT-TTGGCAGGTCAGTGTCGATTTC-GGAGGTTGTAAA---------ATAACTGGGGG-AAAGTAGCTCTGCTTCGGGA---GAGTG-TTATAGACTCTGGGGGATGCAGCCTGCGGGATCGAGGATTGCAGCAAATGC-------------------TATTT--GGC-TTGTCGCCTGATCTCTGG-ACGTTACCTCATTTGTGACAATATT-TTTGACACCGGTGGGTACTAATGGCCAATAGGTTAGAGCGATCAAAAA-TTTTGCTAAGGATGCTGACGTAATGGCTTTAAACGACCCGTCTTGAAACACGGACCAAGGAGTCTAACATATATGCGAG-------------------------------------------------------------------------------tggagaagatgatttgacacacaaattgtctgacattttgaaggccaatcaaaacgtaaagcgttatgaagctgatggtcatcccccacacgttgtaaatgaatttgaagcattgttacaggttcttaataata----------------------------------------------------------------------------------------------------attatataa-tttca--------atttaatataatatcaa--ttgaaa--tttatacttaaa-tttattatttacttgcaatgcaaacagtttcattgtgctacttatatggacaatgaaatggctggtcaacctcaagctcttcagaaatctggtagacctttaaaatcaatacgtgcgcgtctcaagggtaaagaaggtcgtttacgcggtaatctgatgggaaaacgtgtggatttctctgctcgtacagtaattactggtgatccaaacatttcagttgatgaagtcggagttccgaaaagcatagctcaaaacttgacttttccagaattggtgaccccctttaatattgactatcttcaaaaattagtagaaaatggcccttctacacatcctggggctaaatacgtaattagagataccggtgaaaggattgatttaaaacatatatcaggcatgactggtggtttaagattacactacggttggaaagttgagcgtcatctcaatgatggtgacatcgttatattcaatcgtcagccatctttgcacaaaatgtcaatgatggg-------------------------------------------------------------------------------------------------------------------------------------------------------------------------------------------------------------------------------------------------------------------------------------

>Diversispora_gibbosa_KJ850202

AGGAATCCCTAGTAAGCGTGAGTCATCAGCTCACG-TTGATTACGTCCCTGCCCTTTGTACACACCGCCCGTCGCTACTACCGATTGAATGGCTTAGTGAGACCTTTGGATTGGGGTTTTGGGATCGGCAAC---GATCCTATTTCACCGAGAAGTCGGTCAAACTTGGTCATTTAGAGGAAGTAAAAGTCGTAACAAGGTTTCCGTAGGTGAACCTGCGGAAGGATCATT-AAAAAAT-TTTTA-ACCGAGAATTCGT---TTTTCGT----T--------TTCTCG-G------ATAATTTGTATTCAAA-TTCCCACTCTT----------------AAATAATA--TATTATTATATAAACA-TATAT-A-AAAAAAAGAAAACTTTCAACAACGGATCTCTTGGCTCTCGCATCGATGAAGAACGCAGCGAAATGCGATACGTAGTGTGAATTGCAGAATTCCGTGAATCATCGAA-TTTTTGAACGCAAATTGTACTTTTCAGTATTCTGGAAAGTATGCTTGGTTGAGGGTCATTAAAATAACA-TTCGTGAA-------------TTTTTTTTG-------------CGGATTTGAG----TTTT-CC--AGTA--TTT--TATTTATTT---------------AA-TAAATGTTGGTAACTTT-AAAATT-ATTTTA----TTTTCTTGGTTAAAATT-AAAAACGTAC-TATA--TGTGT-GGTTCGTT-GATAATTTGTCCA--T-C--TA----T-ATAT-ATTATGTT-TATACTTGG----TCCACAT-TGGCCTTTGTGTGTGAAT--ATATA-----TTTTTTTTTATGAC-CTCAGCTCAAGCAAGAATACCC-GCTGAACTTAAGCATATCAATAAGCGGAGGAAAAGAAACTAACAAGGATTCCCCTAGTAACGGCGAGTGAAGAGGGAAAAGCTCAAATTTTAAATCTACC-TGGTT-CTT--CTAGGTCGAATTGTAATTTGAAGAAACGATATCTTA-TT-TTGAGGTCTGGTTTAAGTCTTTTGGAACAAGACATCAT-GGGAGGGTGAGAATCCCGTGCATGATCAGA-CC---GAAAT--TTATTAAT---ATTCGTTCTCTAAGAGTCGAGTTGTTTGGGAATGCAGCTCAAAATGGG-T-GGTAGAC-TTCACCTAAGGCTAAATATCAGCGAGAGACCGATAGCGAACAAGTACTGTG-AA-GGAAAGATGAAAAGAACTTTGAAAAGAGAGTTAAAAAGTACGTGAAATTGTTGAAAGGGAAACGATTGAAGTCAGTCATGCCAG-TGAAAAATCAGTTTAACGGA-TTTTT-GGTTCGGAGCA--GGAGGCAGGGT---CAA---ACCGTCTCTCTTTTGGACTTGGAATTTGTTAGATGTACTTTTTCT-TTGGCAGGTCAGTGTCGATTTC-GGAGGTTGTAAA---------ATAACTGGGGG-AAAGTAGCTCTGCTTCGGGA---GAGTG-TTATAGACTCTGGGGGATGCAGCCTGCGGGATCGAGGATTGCAGCAAATGC-------------------TATTT--GGC-TTGTCGCCTGATCTCTGG-ACGTTACCTCATTTGTGACAATATT-TTTGACACCGGTGGGTACTAATGGCCAATAGGTTAGAGCGATCAAAAA-TTTTGCTAAGGATGCTGACGTAATGGCTTTAAACGACCCGTCTTGAAACACGGACCAAGGAGTCTAACATATATGCGAG----------------------------------------------------------------------------------------------------------------------------------------------------------------------------------------------------------------------------------------------------------------------------------------------------------------------------------------------------------------------------------------------------------------------------------------------------------------------------------------------------------------------------------------------------------------------------------------------------------------------------------------------------------------------------------------------------------------------------------------------------------------------------------------------------------------------------------------------------------------------------------------------------------------------------------------------------------------------------------------------------------------------------------------------------------------------------------------------------------------------------------------------------------------------------------------------------------

>Diversispora_gibbosa_KJ850203

AGGAATCCCTAGTAAGCGTGAGTCATCAGCTCACG-TTGATTACGTCCCTGCCCTTTGTACACACCGCCCGTCGCTACTACCGATTGAATGGCTTAGTGAGACCTTTGGATTGGGGTTTTGGGATCGGCAAC---GATCCTATTTCACCGAGAAGTCGGTCAAACTTGGTCATTTAGAGGAAGTAAAAGTCGTAACAAGGTTTCCGTAGGTGAACCTGCGGAAGGATCATT-AAAAAAT-TTTTA-ACCGAGAATTCGT---TTTTCGT----T--------TTCTCG-G------ATAATTTGTATTCAAA-TTCCCACTCTT----------------AAATAATA--TATTATTATATAAACA-TATAT-A-AAAAAAAGAAAACTTTCAACAACGGATCTCTTGGCTCTCGCATCGATGAAGAACGCAGCGAAATGCGATACGTAGTGTGAATTGCAGAATTCCGTGAATCATCGAA-TTTTTGAACGCAAATTGTACTTTTCAGTATTCTGGAAAGTATGCTTGGTTGAGGGTCATTAAAATAACA-TTCGTGAA-------------TTTTTTTTG-------------CGGATTTGAG----TTTT-CC--AGTA--TTT--TATTTATTT---------------AA-TAAATGTTGGTAACTTTAAAAATT-ATTTTA----TTTTCTTGGTTAAAATT-AAAAACGTAC-TATA--TGTGT-GGTTCGTT-GATAATTTGTCCA--T-C--TA----T-ATATAATTATGTT-TATACTTGG----TCCACAT-TGGCCTTTGTGTGTGAAT--ATATA-----TTTTTTTTTATGAC-CTCAGCTCAAGCAAGAATACCC-GCTGAACTTAAGCATATCAATAAGCGGAGGAAAAGAAACTAACAAGGATTCCCCTAGTAACGGCGAGTGAAGAGGGAAAAGCTCAAATTTTAAATCTACC-TGGTT-CTT--CTAGGTCGAATTGTAATTTGAAGAAACGATATCTTA-TT-TTGAGGTCTGGTTTAAGTCTTTTGGAACAAGACATCAT-GGGAGGGTGAGAATCCCGTGCATGATCAGA-CC---GAAAT--TTATTAAT---ATTCGTTCTCTAAGAGTCGAGTTGTTTGGGAATGCAGCTCAAAATGGG-T-GGTAGAC-TTCACCTAAGGCTAAATATCAGCGAGAGACCGATAGCGAACAAGTACTGTG-AA-GGAAAGATGAAAAGAACTTTGAAAAGAGAGTTAAAAAGTACGTGAAATTGTTGAAAGGGAAACGATTGAAGTCAGTCATGCCAG-TGAAAAATCAGTTTAACGGA-TTTTT-GGTTCGGAGCA--GGAGGCAGGGT---CAA---ACCGTCTCTCTTTTGGACTTGGAATTTGTTAGATGTACTTTTTCT-TTGGCAGGTCAGTGTCGATTTC-GGAGGTTGTAAA---------ATAACTGGGGG-AAAGTAGCTCTGCTTCGGGA---GAGTG-TTATAGACTCTGGGGGATGCAGCCTGCGGGATCGAGGATTGCAGCAAATGC-------------------TATTT--GGC-TTGTCGCCTGATCTCTGG-ACGTTACCTCATTTGTGACAATATT-TTTGACACCGGTGGGTACTAATGGCCAATAGGTTAGAGCGATCAAAAA-TTTTGCTAAGGATGCTGACGTAATGGCTTTAAACGACCCGTCTTGAAACACGGACCAAGGAGTCTAACATATATGCGAG----------------------------------------------------------------------------------------------------------------------------------------------------------------------------------------------------------------------------------------------------------------------------------------------------------------------------------------------------------------------------------------------------------------------------------------------------------------------------------------------------------------------------------------------------------------------------------------------------------------------------------------------------------------------------------------------------------------------------------------------------------------------------------------------------------------------------------------------------------------------------------------------------------------------------------------------------------------------------------------------------------------------------------------------------------------------------------------------------------------------------------------------------------------------------------------------------------

>Diversispora_gibbosa_KJ850204_MG459188

AGGAATCCCTAGTAAGCGTGAGTCATCAGCTCACG-TTGATTACGTCCCTGCCCTTTGTACACACCGCCCGTCGCTACTACCGATTGAATGGCTTAGTGAGACCTTTGGATTGGGGTTTTAGGATCGGCAAC---GATCCTATTTCACCGAGAAGTCGGTCAAACTTGGTCATTTAGAGGAAGTAAAAGTCGTAACAAGGTTTCCGTAGGTGAACCTGCGGAAGGATCATT-AAAAAAT-TTTTA-ACCGAGAATTCGT---TTTTCGT----T--------TTCTCG-G------ATAATTTGTATTCAAA-TTCCCACTCTT----------AAAT-AAAATAATA--TATTATTATATAAACA-TATAT-A-AAAAAAAGAAAACTTTCAACAACGGATCTCTTGGCTCTCGCATCGATGAAGAACGCAGCGAAATGCGATACGTAGTGTGAATTGCAGAATTCCGTGAATCATCGAA-TTTTTGAACGCAAATTGTACTTTTCAGTATTCTGGAAAGTATGCTTGGTTGAGGGTCATTAAAATAACA-TTCGTGAA-------------TTTTTTTTG-------------CGGATTTGAG----TTTT-CC--AGTA--TTT--TATTTATTT---------------AATAAAATGTTGGTAACTTTAAAAATT-ATTTTA----TTTTCTTGGTTAAAATT-AAAAACGTAC-TATA--TGTGT-GGTTCGTT-GATAATTTGTCCA--T-C--TA----T-ATAT-ATTATGTT-TATACTTGG----TCCACAT-TGGCCTTTGTGTGTGAAT--ATATA-----TTTTTTTTTATGAC-CTCAGCTCAAGCAAGAATACCC-GCTGAACTTAAGCATATCAATAAGCGGAGGAAAAGAAACTAACAAGGATTCCCCTAGTAACGGCGAGTGAAGAGGGAAAAGCTCAAATTTTAAATCTACC-TGGTT-CTT--CTAGGTCGAATTGTAATTTGAAGAAACGATATCTTA-TT-TTGAGGTCTGGTTTAAGTCTTTTGGAACAAGACATCAT-GGGAGGGTGAGAATCCCGTGCATGATCAGA-CC---GAAAT--TTATTAAT---ATTCGTTCTCTAAGAGTCGAGTTGTTTGGGAATGCAGCTCAAAATGGG-T-GGTAGAC-TTCACCTAAGGCTAAATATCAGCGAGAGACCGATAGCGAACAAGTACTGTG-AA-GGAAAGATGAAAAGAACTTTGAAAAGAGAGTTAAAAAGTACGTGAAATTGTTGAAAGGGAAACGATTGAAGTCAGTCATGCCAG-TGAAAAATCAGTTTAACGGA-TTTTT-GGTTCGGAGCA--GGAGGCAGGGT---CAA---ACCGTCTCTCTTTTGGACTTGGAATTTGTTAGATGTACTTTTTCT-TTGGCAGGTCAGTGTCGGTTTC-GAAGGTTGTAAA---------ATAACTGGGGG-AAAGTAGCTCTGCTTCGGGA---GAGTG-TTATAGACTCTGGGGGATGCAGCCTGCGGGATCGAGGATTGCAGCAAATGC-------------------TATTT--GGC-TTGTCGCCTGATCTCTGG-ACGTTACCTCATTTGTGACAATATT-TTTGACACCGGTGGGTACTAATGGCCAATAGGTTAGAGCGATCAAAAA-TTTTGCTAAGGATGCTGACGTAATGGCTTTAAACGACCCGTCTTGAAACACGGACCAAGGAGTCTAACATATATGCGAG-------------------------------------------------------------------------------tggagaagatgatttgacacacaaattgtctgacattttgaaggccaatcaaaacgtaaagcgttatgaagctgatggtcatcccccacacgttgtaaatgaatttgaagcattgttacaggttcttaataata----------------------------------------------------------------------------------------------------attatataa-tttca--------atttaatataatatcaa--ttgaaa--tttatacttaaa-tttattatttacttgcaatgcaaacagtttcattgtgctacttatatggacaatgaaatggctggtcaacctcaagctcttcagaaatctggtagacctttaaaatcaatacgtgcgcgtctcaagggtaaagaaggtcgtttacgcggtaatctgatgggaaaacgtgtggatttctctgctcgtacagtaattactggtgatccaaacatttcagttgatgaagtcggagttccgaaaagcatagctcaaaacttgacttttccagaattggtgaccccctttaatattgactatcttcaaaaattagtagaaaatggcccttctacacatcctggggctaaatacgtaattagagataccggtgaaaggattgatttaaaacatatatcaggcatgactggtggtttaagattacactacggttggaaagttgagcgtcatctcaatgatggtgacatcgttatattcaatcgtcagccatctttgcacaaaatgtcaatgatggg-------------------------------------------------------------------------------------------------------------------------------------------------------------------------------------------------------------------------------------------------------------------------------------

>Diversispora_peridiata_KT444712_MG459195

AGGAATCCCTAGTAAGCGTGAGTCATCAGCTCACG-TTGATTACGTCCCTGCCCTTTGTACACACCGCCCGTCGCTACTACCGATTGAATGGCTTAGTGAGACCTTTGGATTGGGGTTTTGGGATCGGCAAC---GATCCTATTTCACCGAGAAGTCGGTCAAACTTGGTCATTTAGAGGAAGTAAAAGTCGTAACAAGGTTTCCGTAGGTGAACCTGCGGAAGGATCATT-AAAAAAT-TTTTA-ACCGAGAATTCGT---TTTTCGT----T--------TTCTCG-G------ATAATTTGTATTCAAA-TTCCCACTCTT-------TT-AAAT-AAATTAATA--TATTATTATATAAAAC-ATATA-T-AAAAAAAGAAAACTTTCAACAACGGATCTCTTGGCTCTCGCATCGATGAAGAACGCAGCGAAATGCGATACGTAGTGTGAATTGCAGAATTCCGTGAATCATCGAA-TTTTTGAACGCAAATTGTACTTTTCAGTATTCTGGAAAGTATGCTTGGTTGAGGGTCATAATAATAACA-TTCGTGAA------------TTTTTTTTCG-------------CGGATTTGAG----TTTT-CC--AGTA--TTT--TAT---TAT------A--------AA-AAAATGTTGGTAACTTT-AAAATT-ATTTTAT---TTTCTTGGTTACAAGTT-AAAAACGTAC-TATA--TGTGT-GGTTCGTT-GATATCTTGTCCA--T-C--CT----T-ATAT-ATTATGTT-TATACTTGG----TCCACAT-TGGGTTTTGTGT--AAAT--ATATA------TTTTTTTTATGAC-CTCAGCTCAAGCAAGAATACCC-GCTGAACTTAAGCATATCAATAAGCGGAGGAAAAGAAACTAACAAGGATTCCCCTAGTAACGGCGAGTGAAGAGGGAAAAGCTCAAATTTTAAATCTACC-TGGTT-TTT--CTAGGTCGAATTGTAATTTGAAGAAACGATATCTTA-TT-TTGAGGTCTGGTTTAAGTCTTTTGGAACAAGACATCAT-GGGAGGGTGAGAATCCCGTGCATGATCAGA-CC---GAAAT--TTATTAAT---ATTCGTTTTCTAAGAGTCGAGTTGTTTGGGAATGCAGCTCAAAATGGG-T-GGTACAC-TTCACCTAAGGCTAAATATCAGCGAGAGACCGATAGCGAACAAGTACTGTG-AA-GGAAAGATGAAAAGAACTTTGAAAAGAGAGTTAAAAAGTACGTGAAATTGTTGAAAGGGAAACGATTGAAGTCAGTCATGCCAG-TGAAAAATCAGTTTAACGGA-TTTTT-GGTTCGGAGTA--GGAGGCAGGGT---CAA---ACCGTCTCTCTTTTGAACTTGAAATTTGTTAAATGTACTTTTTCT-TTGGCAGGTCAGTGTCGATTTC-GAAGGTTGTAAA---------ATAACTGGGGG-AAAGTAGCTCTGCTTCGGGA---GAGTG-TTATAGACTCCGGGGGATGCAGCCTGCGGGATCGAGGATTGCAGCAAATGC-------------------TATTT--GGC-TTGTCGCCTGATCTCTGG-ATGTTAACTCATTTGTGACAATATT-TTTGACACCGGTGGGTACTAATAACCAATAGGTTAGAGCGATCAAAAA-TTTTGCTAAGGATGCTGACGTAATGGCTTTAAACGACCCGTCTTGAAACACGGACCAAGGAGTCTAACATATGTGCGAG-----------------------------------------------------------------------------tgtggagaagatgatttgacacacaaattgtccgacattttgaaggccaatcaaaacgtaaagcgttatgaagctgatggtcatcccccacacgttgtaaatgaatttgaagcattgttacaggttcttaataata----------------------------------------------------------------------------------------------------attatataa-tttca--------atttaatataatatcaa--ttgaaa--tttatacttaaa-tttattatttacttgcaatgcaaacagtttcattgtgctacttatatggacaatgaaatggctggtcaacctcaagctcttcagaaatctggtagacctttaaaatcaatacgtgcacgtctcaagggtaaagaaggtcgtttacgcggtaatctgatgggaaaacgtgtggatttctctgctcgtacagtaattaccggtgatccaaacatttcagttgatgaagtcggagttccgaaaagcatagctcaaaacttgacttttccagaattggtgaccccctttaatattgactatcttcaaaaattagtagaaaatggcccttctacacatcctggggctaaatacgtaattagagataccggtgaaaggattgatttaaaacatatatcaggcatgactggtggtttaagattacactacggttggaaagttgagcgtcatctcaatgatggtgacatcgttatattcaatcgtcagccatctttgcacaaaatgtcaatgatggg-------------------------------------------------------------------------------------------------------------------------------------------------------------------------------------------------------------------------------------------------------------------------------------

>Diversispora_peridiata_KT444715

AGGAATCCCTAGTAAGCGTGAGTCATCAGCTCACG-TTGATTACGTCCCTGCCCTTTGTACACACCGCCCGTCGCTACTACCGATTGAATGGCTTAGTGAGACCTTTGGATTGGGGTTTTGGGATCGGCAAC---GATCCTATTTCACCGAGAAGTCGGTCAAACTTGGTCATTTAGAGGAAGTAAAAGTCGTAACAAGGTTTCCGTAGGTGAACCTGCGGAAGGATCATT-AAAAAAT-TTTTA-ATCGAGAATTCGT---TTTTCGT----T--------TTCTCG-G------ATAATTTGTATTCAAA-TTCCCACTCTT-------TT-AAAT-AAATTAATA--TATTATTATATAAAAC-ATATA-T-AAAAAAAGAAAACTTTCAACAACGGATCTCTTGGCTCTCGCATCGATGAAGAACGCAGCGAAATGCGATACGTAGTGTGAATTGCAGAATTCCGTGAATCATCGAA-TTTTTGAACGCAAATTGTACTTTTCAGTATTCTGGAAAGTATGCTTGGTTGAGGGTCATAATAATAACA-TTCGTGAA------------TTTTTTTTCG-------------CGGATTTGAG----TTTT-CC--AGTA--TTT--TAT---TAT------A--------AA-AAAATGTTGGTAACTTT-AAAATT-ATTTTAT---TTTCTTGGTTACAAGTT-AAAAACGTAC-TATA--TGTGT-GGTTCGTT-GATATCTTGTCCA--T-C--CT----T-ATAT-ATTATGTT-TATACTTGG----TCCACAT-TGGGTTTTGTGT--AAAT--ATATA------TTTTTTTTATGAC-CTCAGCTCAAGCAAGAATACCC-GCTGAACTTAAGCATATCAATAAGCGGAGGAAAAGAAACTAACAAGGATTCCCCTAGTAACGGCGAGTGAAGAGGGAAAAGCTCAAATTTTAAATCTACC-TGGTT-TTT--CTAGGTCGAATTGTAATTTGAAGAAACGATATCTTA-TT-TTGAGGTCTGGTTTAAGTCTTTTGGAACAAGACATCAT-GGAAGGGTGAGAATCCCGTGCATGATCAGA-CC---TAAAT--TTATTAAT---ATTCGTTTTCTAAGAGTCGAGTTGTTTGGGAATGCAGCTCAAAATGGG-T-GGTAGAC-TTCACCTAAGGCTAAATATCAGCGAGAGACCGATAGCGAACAAGTACTGTG-AA-GGAAAGATGAAAAGAACTTTGAAAAGAGAGTTAAAAAGTACGTGAAATTGTTGAAAGGGAAACGATTGAAGTCAGTCATGCCAG-TGAAAAATCAGTTTAACGGA-TTTTT-GGTTCGGAGTA--GGAGGCAGGGT---CAA---ACCGTCTCTCTTTTGAACTTGAAATTTGTTAAATGTACTTTTTCT-TTGGCAGGTCAGTGTCGATTTC-GAAGGTTGTAAA---------ATAACTGGGGG-AAAGTAGCTCTGCTTCGGGA---GAGTG-TTATAGACTCCGGGGGATGCAGCCTGCGGGATCGAGGATTGCAGCAAATGC-------------------TATTT--GGC-TTGTCGCCTGATCTCTGG-ATGTTACCTCATTTGTGACAATATT-TTTGACACCGGCGGGTACTAATAACCAATAGGTTAGAGCGATCAAAAA-TTTTGCTAAGGATGCTGACGTAATGGCTTTAAACGACCCGTCTTGAAACACGGACCAAGGAGTCTAACATATATGCGAG----------------------------------------------------------------------------------------------------------------------------------------------------------------------------------------------------------------------------------------------------------------------------------------------------------------------------------------------------------------------------------------------------------------------------------------------------------------------------------------------------------------------------------------------------------------------------------------------------------------------------------------------------------------------------------------------------------------------------------------------------------------------------------------------------------------------------------------------------------------------------------------------------------------------------------------------------------------------------------------------------------------------------------------------------------------------------------------------------------------------------------------------------------------------------------------------------------

>Diversispora_peridiata_KT444713

AGGAATCCCTAGTAAGCGTGAGTCATCAGCTCACG-TTGATTACGTCCCTGCCCTTTGTACACACCGCCCGTCGCTACTACCGATTGAATGGCTTAGTGAGACCTTTGGATTGGGGTTTTGGGATCGGCAAC---GATCCTATTTCACCGAGAAGTCGGTCAAACTTGGTCATTTAGAGGAAGTAAAAGTCGTAACAAGGTTTCCGTAGGTGAACCTGCGGAAGGATCATT-AAAAAAT-TTTTA-ACCGAGAATTCGT---TTTTCGT----T--------TTCTCG-G------ATAATTTGTATTCAAA-TTCCCACTCTT-------TT-AAAT-AAATTAATA--TATTATTATATAAAAC-ATATA-TAAAAAAAAGAAAACTTTCAACAACGGATCTCTTGGCTCTCGCATCGATGAAGAACGCAGCGAAATGCGATACGTAGTGTGAATTGCAGAATTCCGTGAATCATCGAA-TTTTTGAACGCAAATTGTACTTTTCAGTATTCTGGAAAGTATGCTTGGTTGAGGGTCATAATAATAACA-TTCGTGAA------------TTTTTTTTCG-------------CGGATTTGAG----TTTT-CC--AGTA--TTT--TAT---TAT------A--------AA-AAAATGTTGGTAACTTT-AAAATT-ATTTTAT---TTTCTTGGTTACAAGTT-AAAAACGTAC-TATA--TGTGT-GGTTCGTT-GATATCTTGTCCA--TCC--CT----T-ATATAATTATGTT-TATACTTGG----TCCACAT-TGGGTTTTGTGT--AAAT--ATATA------TTTTTTTTATGAC-CTCAGCTCAAGCAAGAATACCC-GCTGAACTTAAGCATATCAATAAGCGGAGGAAAAGAAACTAACAAGGATTCCCCTAGTAACGGCGAGTGAAGAGGGAAAAGCTCAAATTTTAAATCTACC-TGGTT-TTT--CTAGGTCGAATTGTAATTTGAAGAAACGATATCTTA-TT-TTGAGGTCTGGTTTAAGTCTTTTGGAACAAGACATCAT-GGAAGGGTGAGAATCCCGTGCATGATCAGA-CC---TAAAT--TTATTAAT---ATTCGTTTTCTAAGAGTCGAGTTGTTTGGGAATGCAGCTCAAAATGGG-T-GGTAGAC-TTCACCTAAGGCTAAATATCAGCGAGAGACCGATAGCGAACAAGTACTGTG-AA-GGAAAGATGAAAAGAACTTTGAAAAGAGAGTTAAAAAGTACGTGAAATTGTTGAAAGGGAAACGATTGAAGTCAGTCATGCCAG-TGAAAAATCAGTTTAACGGA-TTTTT-GGTTCGGAGTA--GGAGGCAGGGT---CAA---ACCGTCTCTCTTTTGAACTTGAAATTTGTTAAATGTACTTTTTCT-TTGGCAGGTCAGTGTCGATTTC-GAAGGTTGTAAA---------ATAACTGGGGG-AAAGTAGCTCTGCTTCGGGA---GAGTG-TTATAGACTCCGGGGGATGCAGCCTGCGGGATCGAGGATTGCAGCAAATGC-------------------TATTT--GGC-TTGTCGCCTGATCTCTGG-ATGTTACCTCATTTGTGACAATATT-TTTGACACCGGTGGGTACTAATAACCAATAGGTTAGAGCGATCAAAAA-TTTTGCTAAGGATGCTGACGTAATGGCTTTAAACGACCCGTCTTGAAACACGGACCAAGGAGTCTAACATATATGCGAG----------------------------------------------------------------------------------------------------------------------------------------------------------------------------------------------------------------------------------------------------------------------------------------------------------------------------------------------------------------------------------------------------------------------------------------------------------------------------------------------------------------------------------------------------------------------------------------------------------------------------------------------------------------------------------------------------------------------------------------------------------------------------------------------------------------------------------------------------------------------------------------------------------------------------------------------------------------------------------------------------------------------------------------------------------------------------------------------------------------------------------------------------------------------------------------------------------

>Diversispora_peridiata_KT444714_MG459196

AGGAATCCCTAGTAAGCGTGAGTCATCAGCTCACG-TTGATTACGTCCCTGCCCTTTGTACACACCGCCCGTCGCTACTACCGATTGAATGGCTTAGTGAGACCTTTGGATTGGGGTTTTGGGATCGGCAAC---GATCCTATTTCACCGAGAAGTCGGTCAAACTTGGTCATTTAGAGGAAGTAAAAGTCGTAACAAGGTTTCCGTAGGTGAACCTGCGGAAGGATCATT-AAAAAAT-TTTTA-ACCGAGAATTCGT---TTTTCGT----T--------TTCTCG-G------ATAATTTGTATTCAAA-TTCCCACTCTT-------TT-AAAT-AAATTAATA--TATTATTATATAAAAC-ATATA-T-AAAAAAAGAAAACTTTCAACAACGGATCTCTTGGCTCTCGCATCGATGAAGAACGCAGCGAAATGCGATACGTAGTGTGAATTGCAGAATTCCGTGAATCATCGAA-TTTTTGAACGCAAATTGTACTTTTCAGTATTCTGGAAAGTATGCTTGGTTGAGGGTCATAATAATAACA-TTCGTGAA------------TTTTTTTTCG-------------CGGATTTGAG----TTTT-CCCAGTAT--TTT--TAT---TAT-----AA--------AA-AAAATGTTGGTAACTTTAAAAATT-ATTTTAT---TTTCTTGGTTACAAGTT-AAAAACGTAC-TATA--TGTGT-GGTTCGTT-GATATCTTGTCCA--T-C--CT----T-ATAT-ATTATGTT-TATACTTGG----TCCACAT-TGGGTTTTGTGT--AAAT--ATATA-----TTTTTTTTTATGAC-CTCAGCTCAAGCAAGAATACCC-GCTGAACTTAAGCATATCAATAAGCGGAGGAAAAGAAACTAACAAGGATTCCCCTAGTAACGGCGAGTGAAGAGGGAAAAGCTCAAATTTTAAATCTACC-TGGTT-TTT--CTAGGTCGAATTGTAATTTGAAGAAACGATATCTTA-TT-TTGAGGTCTGGTTTAAGTCTTTTGGAACAAGACATCAT-GG-AGGGTGAGAATCCCGTGCATGATCAGA-CC---TAAAT--TTATTAAT---ATTCGTTTTCTAAGAGTCGAGTTGTTTGGGAATGCAGCTCAAAATGGG-T-GGTAGAC-TTCACCTAAGGCTAAATATCAGCGAGAGACCGATAGCGAACAAGTACTGTG-AA-GGAAAGATGAAAAGAACTTTGAAAAGAGAGTTAAAAAGTACGTGAAATTGTTGAAAGGGAAACGATTGAAGTCAGTCATGCCAG-TGAAAAATCAGTTTAACGGA-TTTTT-GGTTCGGAGTA--GGAGGCAGGGT---CAA---ACCGTCTCTCTTTTGAACTTGAAATTTGTTAAATGTACTTTTTCT-TTGGCAGGTCAGTGTCGATTTC-GAAGGTTGTAAA---------ATAACTGGGGG-AAAGTAGCTCTGCTTCGGGA---GAGTG-TTATAGACTCCGGGGGATGCAGCCTGCGGGATCGAGGATTGCAGCAAATGC-------------------TATTT--GGC-TTGTCGCCTGATCTCTGG-ATGTTACCTCATTTGTGACAATATT-TTTGACACCGGTGGGTACTAATAACCAATAGGTTAGAGCGATCAAAAA-TTTTGCTAAGGATGCTGACGTAATGGCTTTAAACGACCCGTCTTGAAACACGGACCAAGGAGTCTAACATGTGTGCGAG--------------------------------------------------------------------------------------------------------ttgtccgacattttgaaggccaatcaaaacgtaaagcgttatgaagctgatggtcatcccccacacgttgtaaatgaatttgaagcattgttacaggttcttaataata----------------------------------------------------------------------------------------------------attatataa-tttca--------atttaatataatatcaa--ttgaaa--tttatacttaaa-tttattatttacttgcaatgcaaacagtttcattgtgctacttatatggacaatgaaatggctggtcaacctcaagctcttcacaaatctggtagacctttaaaatcaatacgtgcacgtctcaagggtaaagaaggtcgtttacgcggtaatctgatgggaaaacgtgtggatttctctgctcctacagtaattaccggtgatccaaacatttcagttgatgaagtcggagttccgaaaagcatagctcaaaacttgacttttccagaattggtgaccccctttaatattgactatcttcaaaaattagtagaaaatggcccttctacacatcctggggctaaatacgtaattagagataccggtgaaaggattgatttaaaacatatatcaggcatgactggtggtttaagattacactacggttggaaagttgagcgtcatctcaatgatggtgacatcgttatattcaatcgtcagccatctttgcacaaaatgtcaatgatgggtaa----------------------------------------------------------------------------------------------------------------------------------------------------------------------------------------------------------------------------------------------------------------------------------

>Diversispora_trimurales_KJ850199_MG459201

AGGAATCCCTAGTAAGCATGAGTCATCAGCTCATG-TTGATTACGTCCCTGCCCTTTGTACACACCGCCCGTCGCTACTACCGATTGAATGGCTTAGTGAGACCTTTGGATTGGGGTTTTGGGATCGGCAAC---GACCCTATTTCTCCGAGAAGTCGGTCAAACTTGGTCATTTAGAGGAAGTAAAAGTCGTAACAAGGTTTCCGTAGGTGAACCTGCGGAAGGATCATT-AAAAAAT-TTTTA-ACCGAGAA----------TTCGT----T--------TTCTCG-G------ATAATTTGTATTCAAA-TTCCCACTCTT-------AT-AAAT---------A--ATAAATTATATAAAAC-ATATA-T-AAAAAAAGAAAACTTTCAACAACGGATCTCTTGGCTCTCGCATCGATGAAGAACGCAGCGAAATGCGATACGTAGTGTGAATTGCAGAATTCCGTGAATCATCGAA-TTTTTGAACGCAAATTGTACTTTTCAGTATTCTGGAAAGTATGCTTGGTTGAGGGTCATTAATATAACA-TTCGTGAA------------TTTTTTTTCG-------------CGGATTTGAG----TTTT-CC--AGTA--TTT--TAT---TAT---------------AA-AAAATGTTGGTAACTTT-AAAATT-ATTTTA----TTTCTTGGTTACAAGTTAAAAAACGTACTTATA--TGTGT-GGTTCGTT-GATAATTTGTCCCA-T-C--TA----TAATAT-ATTATGTT-TGTACTTGG----TCCACAT-TGGGTTCTGTAT--GAAC--ATATA------TTTTTTTTATGAC-CTCAGCTCAAGCAAGAATACCC-GCTGAACTTAAGCATATCAATAAGCGGAGGAAAAGAAACTAACAAGGATTCCCCTAGTAACGGCGAGTGAAGAGGGAAAAGCTCAAATTTTAAATCTACC-TGGTT-TTT--CTAGGTCGAATTGTAATTTGAAGAAGCGATATCTTA-TT-TTGAGGTCTGGTTTAAGTCTTTTGGAACAAGACATCAT-GGAAGGGTGAGAATCCCGTGCATGATCAGA-CC---GAAAT--ATATTAAT---ATTCGTTTTCTAAGAGTCGAGTTGTTTGGGAATGCAGCTCAAAATGGG-T-GGTAGAC-TTCACCTAAGGCTAAATATCAGCGAGAGACCGATAGCGAACAAGTACTGTG-AA-GGAAAGATGAAAAGAACTTTGAAAAGAGAGTTAAATAGTACGTGAAATTGTTGAAAGGGAAACGATTGAAGTCAGTCATGCCAG-TGATAAATCAGTTTAACGGA-TTGAT-GGTTCGGGGT---TGAGGCAGGGT---CAA---ACCGTCTCTCTTTTGAACTTGAAATTTGGTAAATGTACTTTTTCT-TTGGCAGGTCAGTGTCGATTTC-GGAGGTTGTACA---------ATAACTGGGGG-AAAGTAGCTCTGCTTCGGGA---GAGTG-TTATAGACTCTGGGGGATGCAGCCTGCGGGATCGAGGATTGCAGCAAATGC-------------------TATTT--GGC-TTGTCGCCTGATCTCTGG-ACGTTACCTCATTTGTGACAACATT-TTTGACACCGGTGGGTACTAATGGCCAATAGGTTAGAGCGATCAAAAA-TTTTGCTAAGGATGCTGACGTAATGGCTTTAAACGACCCGTCTTGAAACACGGACCAAGGAGTCTAACATATGTGCGAG-------------------------------------------------------------------------------tggagaagatgatttgacacacaaattgtccgacattttgaaggccaatcaaaacgtaaagcgttatgaagctgatggtcatcccccacacgttgtaaatgaatttgaagcattgttacaggttcttaataata----------------------------------------------------------------------------------------------------attatataa-tttca--------a-ttaatttaatatcaa--ttgaaa--tttatacttaaa-tttattatttacttgcaatgcaaacagtttcattgtgctacttatatggacaatgaaatggctggtcaacctcaagctcttcagaaatctggtagacctttaaaatcaatacgcgcacgtctcaagggtaaagaaggacgtttacgcggtaatctgatgggaaaacgtgtagatttctctgctcgtacagtaattaccggtgatccaaatatttcagttgatgaagtcggagttccgaaaagcatagctcaaaacttgacctttccagaattggtgaccccctttaatattgactatcttcaaaaattagtagaaaatggtccttctacacatcctggggctaaatacgtaattagagatactggcgaaaggattgatttaaaacatatatcaggcatgactggtggcttaagattacactacggttggaaagttgagcgtcatctcaatgatggtgacatcgttatattcaatcgtcagccatctttgcacaaaatgtcaatgatggg-------------------------------------------------------------------------------------------------------------------------------------------------------------------------------------------------------------------------------------------------------------------------------------

>Diversispora_trimurales_KJ850200_MG459200

AGGAATCCCTAGTAAGCATGAGTCATCAGCTCATG-TTGATTACGTCCCTGCCCTTTGTACACACCGCCCGTCGCTACTACCGATTGAATGGCTTAGTGAGACCTTTGGATTGGGGTTTTGGGATCGGCAAC---GACCCTATTTCTCCGAGAAGTCGGTCAAACTTGGTCATTTAGAGGAAGTAAAAGTCGTAACAAGGTTTCCGTAGGTGAACCTGCGGAAGGATCATT-AAAAAAT-TTTTA-ACCGAGAA----------TTCGT----T--------TTCTCG-G------ATAATTTGTATTCAAA-TTCCCACTCTT-------AT-AAAT---------A--ATAAATTATATAAAAC-ATATA-T-AAAAAAAGAAAACTTTCAACAACGGATCTCTTGGCTCTCGCATCGATGAAGAACGCAGCGAAATGCGATACGTAGTGTGAATTGCAGAATTCCGTGAATCATCGAA-TTTTTGAACGCAAATTGTACTTTTCAGTATTCTGGAAAGTATGCTTGGTTGAGGGTCATTAATATAACA-TTCGTGAA------------TTTTTTTTCG-------------CGGATTTGAG----TTTT-CC--AGTA--TTT--TAT---TAT---------------AA-AAAATGTTGGTAACTTT-AAAATT-ATTTTA----TTTCTTGGTTACAAGTT-AAAAACGTAC-TATA--TGTGT-GGTTCGTT-GATAATTTGTCCA--T-C--TA----T-ATAT-ATTATGTT-TGTACTTGG----TCCACAT-TGGGTTCTGTAT--GAAC--ATATA------TTTTTTTTATGAC-CTCAGCTCAAGCAAGAATACCC-GCTGAACTTAAGCATATCAATAAGCGGAGGAAAAGAAACTAACAAGGATTCCCCTAGTAACGGCGAGTGAAGAGGGAAAAGCTCAAATTTTAAATCTACC-TGGTT-TTT--CTAGGTCGAATTGTAATTTGAAGAAGCGATATCTTA-TT-TTGAGGTCTGGTTTAAGTCTTTTGGAACAAGACATCAT-GGAAGGGTGAGAATCCCGTGCATGATCAGA-CC---GAAAT--ATATTAAT---ATTCGTTTTCTAAGAGTCGAGTTGTTTGGGAATGCAGCTCAAAATGGG-T-GGTAGAC-TTCACCTAAGGCTAAATATCAGCGAGAGACCGATAGCGAACAAGTACTGTG-AA-GGAAAGATGAAAAGAACTTTGAAAAGAGAGTTAAATAGTACGTGAAATTGTTGAAAGGGAAACGATTGAAGTCAGTCATGCCAG-TGATAAATCAGTTTAACGGA-TTGAT-GGTTCGGGGT---TGAGGCAGGGT---CAA---ACCGTCTCTCTTTTGAACTTGAAATTTGGTAAATGTACTTTTTCT-TTGGCAGGTCAGTGTCGATTTC-GGAGGTTGTACA---------ATAACTGGGGG-AAAGTAGCTCTGCTTCGGGA---GAGTG-TTATAGACTCTGGGGGATGCAGCCTGCGGGATCGAGGATTGCAGCAAATGC-------------------TATTT--GGC-TTGTCGCCTGATCTCTGG-ACGTTACCTCATTTGTGACAACATT-TTTGACACCGGTGGGTACTAATGGCCAATAGGTTAGAGCGATCAAAAA-TTTTGCTAAGGATGCTGACGTAATGGCTTTAAACGACCCGTCTTGAAACACGGACCAAGGAGTCTAACATATGTGCGAG-------------------------------------------------------------------------------tggagaagatgatttgactcacaaattgtccgacattttgaaggccaatcaaaacgtaaagcgttatgaagctgatggtcatcccccacacgttgtaaatgaatttgaagcattgttacaggttcttaataata----------------------------------------------------------------------------------------------------attatataa-tttca--------a-ttaatttaatatcaa--ttgaaa--tttatacttaaa-tttattatttacttgcaatgcaaacagtttcattgtgctacttatatggacaatgaaatggctggtcaacctcaagctcttcagaaatctggtagacctttaaaatcaatacgcgcacgtctcaagggtaaagaaggacgtttacgcggtaatctgatgggaaaacgtgtagatttctctgctcgtacagtaattaccggtgatccaaatatttcagttgatgaagtcggagttccgaaaagcatagctcaaaacttgacctttccagaattggtgaccccctttaatattgactatcttcaaaaattagtagaaaatggtccttctacacatcctggggctaaatacgtaattagagatactggcgaaaggattgatttaaaacatatatcaggcatgactggtggcttaagattacactacggttggaaagttgagcgtcatctcaatgatggtgacatcgttatattcaatcgtcagccatctttgcacaaaatgtcaatgatggg-------------------------------------------------------------------------------------------------------------------------------------------------------------------------------------------------------------------------------------------------------------------------------------

>Diversispora_trimurales_KJ850198_MG459199

AGGAATCCCTAGTAAGCATGAGTCATCAGCTCATG-TTGATTACGTCCCTGCCCTTTGTACACACCGCCCGTCGCTACTACCGATTGAATGGCTTAGTGAGACCTTTGGATTGGGGTTTTGGGATCGGCAAC---GACCCTATTTCTCCGAGAAGTCGGTCAAACTTGGTCATTTAGAGGAAGTAAAAGTCGTAACAAGGTTTCCGTAGGTGAACCTGCGGAAGGATCATT-AAAAAAT-TTTTA-ACCGAGAA----------TTCGT----T--------TTCTCG-G------ATAATTTGTATTCAAA-TTCCCACTCTT----------AAAT---------A--ATAAATTATATAAAAC-ATATA-T-AAAAAAAGAAAACTTTCAACAACGGATCTCTTGGCTCTCGCATCGATGAAGAACGCAGCGAAATGCGATACGTAGTGTGAATTGCAGAATTCCGTGAATCATCGAA-TTTTTGAACGCAAATTGTACTTTTCAGTATTCTGGAAAGTATGCTTGGTTGAGGGTCATTAAAATAACA-TTCGTGAA------------ATTTTTTTCG-------------CGGATTTGAG----TTTT-CC--AGTA--TTT--TAT---TAT---------------AA-AAAATGTTGGTAACTTT-AAAATT-ATTTTA----TTTCTTGGTTACAAGTT-AAAAACGTAC-TATA--TGTGT-GGTTCGTTGGATAATTTGTCCC--A-T--CT--AAT-ATAT-ATTATGTT-TGTACTTGG----TCCACATTTGGGTTCTGTGT--GAAC--ATATAT----TTTTTTTTTATGAC-CTCAGCTCAAGCAAGAATACCC-GCTGAACTTAAGCATATCAATAAGCGGAGGAAAAGAAACTAACAAGGATTCCCCTAGTAACGGCGAGTGAAGAGGGAAAAGCTCAAATTTTAAATCTACC-TGGT--TTT--CTAGGTCGAATTGTAATTTGAAGAAGCGATATCTTA-TT-TTGAGGTCTGGTTTAAGTCTTTTGGAACAAGACATCAT-GG-AGGGTGAGAATCCCGTGCATGATCAGA-CC---GAAAT--ATATTAAT---ATTCGTTTTCTAAGAGTCGAGTTGTTTGGGGATGCAGCTCAAAATGGG-T-GGTAGAC-TTCACCTAAGGCTAAATATCGGCGAGAGACCGATAGCGAACAAGTACTGTG-AA-GGAAAGATGAAAAGAACTTTGAAAAGAGAGTTAAATAGTACGTGAAATTGTTGAAAGGGAAACGATTGAAGTCAGTCATGCCAG-TGATAAATCAGTTTAACGGA-TTGAT-GGTTCGGGGT---TGAGGCAGGGT---CAA---ACCGTCTCTCTTTTGAACTTGAAATTTGGTAAATGTACTTTTTCT-TTGGCAGGTCAGTGTCGATTTC-GGAGGTTGTACA---------ATAACTGGGGG-AAAGTAGCTCTGCTTCGGGA---GAGTG-TTATAGACTCTGGGGGATGCAGCCTGCGGGATCGAGGATTGCAGCAAATGC------------------CCCCCC--CCC-CCCCCCCCCCCTCTCCCG-ACGTTACCTCATTTGTGACAACATT-TTTGACACCGGTGAGTACTAATGGCCAATAGGTTAGAACGATCAAAAA-TTTTGCTAAGGATGCTGACGTAATGGCTTTAAACGACCCGTCTTGAAACACGGACCAAGGAGTCTAACATATATGCGAG--------------------------------------------------------------------------------ggagaagatgatttgactcacaaattgtccgacattttgaaggccaatcaaaacgtaaagcgttatgaagctgatggtcatcccccacacgttgtaaatgaatttgaagcattgttacaggttcttaataata----------------------------------------------------------------------------------------------------attatataa-tttca--------a-ttaatttaatatcaa--ttgaaa--tttatacttaaa-tttattatttacttgcaatgcaaacagtttcattgtgctacttatatggacaatgaaatggctggtcaacctcaagctcttcagaaatctggtagacctttaaaatcaatacgcgcacgtctcaagggtaaagaaggacgtttacgcggtaatctgatgggaaaacgtgtagatttctctgctcgtacagtaattaccggtgatccaaatatttcagttgatgaagtcggagttccgaaaagcatagctcaaaacttgacctttccagaattggtgaccccctttaatattgactatcttcaaaaattagtagaaaatggtccttctacacatcctggggctaaatacgtaattagagatactggcgaaaggattgatttaaaacatatatcaggcatgactggtggcttaagattacactacggttggaaagttgagcgtcatctcaatgatggtgacatcgttatattcaatcgtcagccatctttgcacaaaatgtcaatgatggg-------------------------------------------------------------------------------------------------------------------------------------------------------------------------------------------------------------------------------------------------------------------------------------

>Corymbiglomus_corymbiforme_KF060295_MG459179

AGGAATCCCTAGTAAGCGTGAGTCATCAGCTCACG-TTGATTACGTCCCTGCCCTTTGTACACACCGCCCGTCGCTACTACCGATTGAATGGCTTAGTGAGACCTTTGGATTGAAT-TTTGGAAGCGGCAAC----GTGACCAATGTTCGAGAAGTCGGTCAAACTTGGTCATTTAGAGGAAGTAAAAGTCGTAACAAGGTTTCCGTAGGTGAACCTGCGGAAGGATCATT-AAAATTT-TTTTT-TTTA------------ACCTCCT----C--------TTCGGGGG--AGG-GGTATTTGTATTCAAA-TTCCACTCTTCAAATTT-TT-TTAA-ACA---------TTCATTTTATTATAC-ATAAT-A-ATAAAAAGACAACTTTCAACAACGGATCTCTTGGCTCTCGCATCGATGAAGAACGCAGCGAAATGCGATACGTAGTGTGAATTGCAGGTTTATGTGAATCATCGAA-TCTTCGAACACAAATTGTACTTCTCAG-TTTCTGGGGAGTATGCCTGGTTGAGGGTCATTTCAATAACA-ATCGCGAA----------------TTTTCG-------------CGGATCTGGG----TTCTTCC--GGTG---TT-TTTTT--------------------TA-TATACGCTGGTGACCTT-AAAATA-ATTT-------TTTTTCGGTACAAGTC--AAAACGTGC-TATGT-GACGT-GGTTCGTT-GAAAACTTGACCT-------CA----T-ACAT-ATTATGTT-TGGGTTGAT----CTCATTGGAGTGATCGATGTC-AAGC--ATATA-----TTTTTTTTAATGAC-CTCAACTCAAGCAAGGATACCC-GCTGAACTTAAGCATATCAATAAGCGGAGGAAAAGAAACTAACAAGGATTCCCTCAGTAACGGCGAGTGAAGTGGGAAAAGCTCAAATTTTAAATCTACCTTGG---TCACACCAGGTCGAATTGTAATTTGAAGAAGCGTTTTC-GA-TATTTTTGGTTTGGTTGAAGGCCTTTGGAACAAGGCATCAT-GGGAGGGTGAGAATCCCGTACATGGTCAGA-CC---GATAT--GT-CACAG---ATGCGCTCTCTAAGAGTCGAGTTGTTTGGGAATGCAGCTCAAAATGGG-T-GGTAGAC-TTCACCTAAGGCTAAATATTAGCGAGAGACCGATAGCGAACAAGTACCGTG-AG-GGAAAGATGAAAAGTACTTTGAAAAGAGAGTTAAATAGTACGTGAAATTGTTGAAAGGGAAACGATTGAAGTCAGTCGTGCCAGTTAAGGAATCAACCCGGTGTGGTTTCG-GGTTCGCGAGT--CGAGATAGGGT---CAA---ACCATCTCTCTCTCGAACTTGGGGTTCGTCGGGTGTACTTTCTTG-TTGGCAGGTCAACGTCGATTTT-GGGGGTTGTAAA---------ATAACTGGGGG-AAAGTAGCTC--CTCTCGGG---GAGTG-TTATAGACCCTGGGGGATGCAGCCTGTGGGATCGAGGATTGCAGCAAATGC------------------TTTTTT--TGC-TTGTCGCCTGTCCGCTGA-TCGTCGCCCCGCTAGTGGCAACATTTCTTGACACTTTGT-GGACCTGTCGGTTACCGGTTAGAGCGATCTAAAA--TTTGCTAAGGATGTTGACGTAATGGCTTTAAACGACCCGTCTTGAAACACGGACCAAGGAGTCTAACATGTGTGCGAG-------------------------------------------------------------------------------tggagaagatgatttgacacataaattgtcagacattttgaaggctaatcaaaacgtaaagcgttatgaggctgatggtcatcccccacacgtcctaaacgaatttgaagctttgttacaggtttgatttttt---------------------------------------------------------------------------------------------------------tacaa-cttcatttaattta-ttgatttaatatcgattttgaaa--tttatacttaaa-tttat------------atgcgaatagtttcattgtgcaacttatatggataacgaaatggcaggtcaacctcaagctcttcaaaaatccggtagacctttaaagtcaatacgcgcgcgcctcaagggtaaagaaggacgtttacgtggtaatctgatgggaaagcgtgtagatttctctgctcgtacagtaattacgggtgatccaaatatttcagttgacgaagtcggggttccgagaagcatagctcaaaatttgacttttccagaattggtgaccccctttaacattgattatcttcaaaaattagtagagaatggcccttctacacatccgggggctaaatacgtaattagagataccggtgaaagaattgatcttaaacatatatcaggcatgactggtggtttaagattacactacggttggaaggttgaacgtcatctcaatgatggtgacatcgttatattcaatcgtcaaccatctttacataaaatgtcaatgatggg-------------------------------------------------------------------------------------------------------------------------------------------------------------------------------------------------------------------------------------------------------------------------------------

>Corymbiglomus_corymbiforme_KF060298_MG45918

AGGAATCCCTAGTAAGCGTGAGTCATCAGCTCACG-TTGATTACGTCCCTGCCCTTTGTACACACCGCCCGTCGCTACTACCGATTGAATGGCTTAGTGAGACCTTTGGATTGAAT-TTTGGAAGCGGCAAC----GTGACCAATGTTCGAGAAGTCGGTCAAACTTGGTCATTTAGAGGAAGTAAAAGTCGTAACAAGGTTTCCGTAGGTGAACCTGCGGAAGGATCATT-AATAA---TTTTT-TTAA------------CCCTTCT----C--------ATCGGG-G------GTTATTTGTATTCAAA-TTCCACTCTTCAAA-TT-TT-TTGA-ACA---------TTCATTTTATTATAC-ATAAT-A-ATAAAAAGACAACTTTCAACAACGGATCTCTTGGCTCTCGCATCGATGAAGAACGCAGCGAAATGCGATACGTAGTGTGAATTGCAGGTTTATGTGAATCATCGAA-TCTTCGAACACAAATTGTACTTCTCAGTTTTCTGGGGAGTATGCCTGGTTGAGGGTCATTTCAATAACA-ATCGCGAA----------------TTTTCG-------------CGGATCTGGG----TTCTTCC--GGTG--TTT-TTTTT--------------------TA-TATACGCTGGTGACCTT-AAAATA-ATTT-------TTTTTCGGTACAAGTC--AAAACGTGC-TATGT-GACGG-GGTTCGTT-GAAAACTTGACCT-------CA----T-ACAT-ATTATGTT-TGGGTTGAT----CTTATTGGAGTGATCGATGTT-AAGC--ATATT-----TTTTTTTAAATGAC-CTCAACTCAAGCAAGGATACCC-GCTAAACTTAAGCATATCAATAAGCGGAGGAAAAGAAACTAACAAGGATTCCCTCAGTAACGGCGAGTGAAGTGGGAAAAGCTCAAATTTTAAATCTACC-TGG---TTACACCAGGTCAAATTGTAATTTGAAGAAGCGTTTTC-GA-TA-TTTTGGTCTGGCTGAAGCCCTTTGGAACAAGGCATCAT-GGAAGGGTGAGAATCCCGTACATGGTCAGA-CC---GATAT--GT-CACAG---ATGCGCTCTCTAAGAGTCGAGTTGTTTGGGAATGCAGCTCAAAATGGG-T-GGTAGAC-TTCACCTAAGGCTAAATATTAGCGAGAGACCGATAGCGAACAAGTACCGTG-AG-GGAAAGATGAAAAGTACTTTGAAAAGAGAGTTAAATAGTACGTGAAATTGTTGAAAGGGAAACGATTGAAGTCAGTCGTGCCAGTTAAGGAATCAACCCGGTGTGGTTTCG-GGTTCGCGAGT--TGAGATAGGGT---CAA---ACCATCTCTCTCTCGAACTTGGGGTTCGTCGGGTGTACTTTCTTG-TTGGCAGGTCAACGTCGATTTT-GGGGGTTGTAAA---------ATAACCGGGGG-AAAGTAGCTC--CTCTCGGG---GAGTG-TTATAGACCCTGGGGGATGCAGCCCGCGGGATCGAGGATTGCAGCAAATGC--------------------TTTT--TGC-TTGTCGCCTGTCCGCTGG-TCGTCGCCCCGCTGGTGGCAACATTTCTTGACACTTTGTGGGACCTGTCGGTTACCGGTTAGAGCGATCTAAAA--TTTGCTAAGGATGTTGACGTAATGGCTTTAAACGACCCGTCTTGAAACACGGACCAAGGAGTCTAACATATATGCGAG-------------------------------------------------------------------------------tggagaagatgatttgacacataaattgtcagacattttgaaggctaatcaaaacgtaaagcgttatgaggctgatggtcatcccccacacgtcgtaaacgaatttgaagctttgttacaggtttgatttttt---------------------------------------------------------------------------------------------------------tacaa-cttcatttaattta-ttgatttaatatcgattttgaaa--tttatacttaaa-tttat------------atgcgaatagtttcattgtgcaacttatatggataacgaaatggcaggtcaacctcaagctcttcaaaaatccggtagacctttaaagtcaatacgcgcgcgcctcaagggtaaagaaggacgtttacgtggtaatctgatgggaaagcgtgtagatttctctgctcgtacagtaattacgggtgatccaaatatttcagttgacgaagtcggggttccgagaagcatagctcaaaatttgacttttccagaattggtgaccccctttaacattgattatcttcaaaaattagtagagaatggcccttctacacatccgggggctaaatacgtaattagagataccggtgaaagaattgatcttaaacatatatcaggcatgactggtggtttaagattacactacggttggaaggttgaacgtcatctcaatgatggtgacatcgttatattcaatcgtcaaccatctttacataaaatgtcaatgatggg-------------------------------------------------------------------------------------------------------------------------------------------------------------------------------------------------------------------------------------------------------------------------------------

>Corymbiglomus_corymbiforme_KF060296_MG459181

AGGAATCCCTAGTAAGCGTGAGTCATCAGCTCACG-TTGATTACGTCCCTGCCCTTTGTACACACCGCCCGTCGCTACTACCGATTGAATGGCTTAGTGAGACCTTTGGATTGAAT-TTTGGAAGCGGCAAC----GTGACCAATGTTCGAGAAGTCGGTCAAACTTGGTCATTTAGAGGAAGTAAAAGTCGTAACAAGGTTTCCGTAGGTGAACCTGCGGAAGGATCATT-AATAATT-TTTTT-TTAA------------CCCTCCT----C--------ATCGGGGG------GTTATTTGTATTCAAA-TTCCACTCTTCAAA-TT-TT-TATA-ACA---------TTCATTTTATTATAC-ATAAT-A-ATAAAAAGACAACTTTCAACAACGGATCTCTTGGCTCTCGCATCGATGAAGAACGCAGCGAAATGCGATACGTAGTGTGAATTGCAGGTTTATGTGAATCATCGAA-TCTTCGAACACAAATTGTACTTCTCAGTTTTCTGGGGAGTATGCCTGGTTGAGGGTCATTTCAATAACA-ATCGCGAA----------------TTTTCG-------------CGGATCTGGG----TTCTTCC--GGTGTTTTT-TTTTT--------------------TA-TATACGCTGGTGACCTT-AAAATA-ATTT-------TTTTTCGGTACAAGTC--AAAACGTGC-TATGT-GACGT-GGTTCGTT-GAAAACTTGACCT-------CA----T-ACAT-ATTATGTT-TGGATTGAT----CTCATTGGAGTGATCGATGTT-AAGC--ATATA-----TTTTTTTTAATGAC-CTCAACTCAAGCAAGGATACCC-GCTGAACTTAAGCATATCAATAAGCGGAGGAAAAGAAACTAACAAGGATTCCCTCAGTAACGGCGAGTGAAGTGGGAAAAGCTCAAATTTTAAATCTACC-TGG---TCACACCAGGTCGAATTGTAATTTGAAGAAGCGTTTTC-GA-TA-TTTTGGTCTGGCTGAAGCCCTTTGGAACAAGGCATCAT-GG-AGGGTGAGAATCCCGTACATGGTCAGA-CC---GATAT--GT-CACAG---ATGTGCTCTCTAAGAGTCGAGTTGTTTGGGAATGCAGCTCAAAATGGGTT-GGTAGAC-TTCACCTAAGGCTAAATATTAGCGAGAGACCGATAGCGAACAAGTACCGTG-AG-GGAAAGATGAAAAGTACTTTGAAAAGAGAGTTAAATAGTACGTGAAATTGTTGAAAGGGAAACGATTGAAGTCAGTCGTGCCAGTTAAGGAATCAACCCGGTGTGGTTTCG-GGTTCGCGAGT--CGAGATAGGGT---TAA---ACCATCTCTCTTTCGAACTTGGGGTTCGTCGGGTGTACTTTCTTG-TTGGCAGGTCAACGTCGATTTT-GGGGGTTGTAAA---------ATAACCGGAGG-AAAGTAGCTC--CTCTTAGA---GAGTG-TTATAGACCCTGGGGGATGCAGCCCACGGGATCGAGGATTGCAGCAAATGC--------------------TTTT--TGC-TTGTCACCTATCCGCTGA-TCATCATCCCACTGGTGGCAATATTTCTTGACACCCTGTGGGACTTGAGGGTTACTGGTTAGAGCAATCTAAAA--TTTGCTAAGGATGTTGACGTAATGGCTTTAAACGACCCGTTTTGAAACACGGACCAAGAAGTCTAACATGTGTGCGAG-------------------------------------------------------------------------------tggagaagatgatttgacacataaattgtcagacattttgaaggctaatcaaaacgtaaagcgttatgaggctgatggtcatcccccacacgtcgtaaacgaatttgaagctttgttacaggtttgatttttt---------------------------------------------------------------------------------------------------------tacaa-cttcatttaattta-ttgatttaatatcgattttgaaa--tttatacttaaa-tttat------------atgcgaatagtttcattgtgcaacttatatggataacgaaatggcaggtcaacctcaagctcttcaaaaatccggtagacctttaaagtcaatacgcgcgcgcctcaagggtaaagaaggacgtttacgtggtaatctgatgggaaagcgtgtagatttctctgctcgtacagtaattacgggtgatccaaatatttcagttgacgaagtcggggttccgagaagcatagctcaaaatttgacttttccagaattggtgaccccctttaacattgattatcttcaaaaattagtagagaatggcccttctacacatccgggggctaaatacgtaattagagataccggtgaaagaattgatcttaaacatatatcaggcatgactggtggtttaagattacactacggttggaaggttgaacgtcatctcaatgatggtgacatcgttatattcaatcgtcaaccatctttacataaaatgtcaatgatggg-------------------------------------------------------------------------------------------------------------------------------------------------------------------------------------------------------------------------------------------------------------------------------------

>Redeckera_megalocarpum_HG518627

-------CCTAGTAAGCGTGAGTCATCAGCTCATG-TTGATTACGTCCCTGCCCTTTGTACACACCGCCCGTCGCTACTACCGATTGAATGGCTTAGTGAGACCTTTGGATTGAAG-TTTGGGAGCGGCAAC----GTAACCTTATTTCGAGAAGTCGGTCAAACTTGGTCATTTAGAGGAAGTAAAAGTCGTAACAAGGTTTCCGTAGGTGAACCTGCGGAAGGATCATT-AATAATT-TTTT-------------------TCCCTC----T--------TTTTGGGGTTCAA-TTTTATTGTATTCAAATTCCCACTCTTT-------AA-AAAT-TTAATCATAATAAATTTTATGTATAAT-ATAAA---TGAAAAAGACAACTTTCAACAACGGATCTCTTGGTTCTCGCATCGATGAAGAACGCAGCGAAATGCGATACGTAGTGTGAATTGCAGGTTTACGTGAATCATCGAA-TCTTCGAACGCAAATTGCACTTCCCAGTAATCTGGGGAGTATGCATGGTTGAGGGTCATCCAATTAACA-TTCGTGAATTT----------TTTTTTTCG-------------CGGATTTGAG----TTTT-CC--GGTG--TTT-TCACAT---------AA--------GA-GGGATGCTGGTGACTTT-AAAATG-ATTT---------TTTCGGTTCAAGTC--AAAACGTGC-TATGT-GACGT-GGTTCGTT-GAAAACTTGACCT----C--AA----C-ATAT-ATTATGTCTTGCATCGAT----CTCGTTCGAGTGATGGGTGT-CAAGC--ATATAT----TTTTTTTTCATGAC-CTCAACTCAGGCAAGGATACCC-GCTGAACTTAAGCATATCAATAAGCGGAGGAAAAGAAACTAACAAGGATTCCCTTAGTAACGGCGAGTGAAGTGGGAAAAGCTCAAATTTTAAATCTGCC-TGG---CACAACCAGGTCGAGTTGTAATTTGAAGAAGCGCTTTC-GG-TG-TTTTGATCTGGTTAAAGTTCTTTGGAACAAGACATCAT-GG-AGGGTGAGAATCCCGTGCATGATCAGA-TC---GAAAT--AC-TCCAG---ATGCGCTCTCTAAGAGTCGAGTTGTTTGGGAATGCAGCTCAAATTGGG-T-GGTAGAC-TTCACCTAAGGCTAAATATCAGCGAGAGACCGATAGCGAACAAGTACCGTG-AG-GGAAAGATGAAAAGAACTTTGAAAAGAGAGTTAAACAGTACGTGAAATTGTTGAAAGGGAAACGATTGAAGTCAGTCGTGCCGT-TGAGAAATCAGCCTGATGGG-TTTTTGGGTTCGTGGGT--TGAGGTAGGGT---CAAACCACCACCTCTCTTTCGGACTCGATGCTTGCCGGGTGTACTTTCTTT-TTGGCAGGTCAACGTCGATTTT-GGGGGTTGTAAA---------ATAACTGGGGG-AATGTAGCTC--CTTTC--G---GAGTG-TTATAGACCCTGGGGGATGCAACCCGTGGGATCGAGGATTGCAGCAAATGC-------------------TTCAT--TGC-TTGTCGCCTGCCCGCTGG-ACGTCGCCCCGCTTGTGACAACATTTCTTGACACATTGTGGGACTTGTCGGTTACCGGTTAGAACGATCTAAAA--TTTGCTAAGGATGTTGACGTAATGGTTTCAAACGACCCGTCTTGAAACACGGACCAAGGAG---------------------------------------------------------------------------------------------------------------------------------------------------------------------------------------------------------------------------------------------------------------------------------------------------------------------------------------------------------------------------------------------------------------------------------------------------------------------------------------------------------------------------------------------------------------------------------------------------------------------------------------------------------------------------------------------------------------------------------------------------------------------------------------------------------------------------------------------------------------------------------------------------------------------------------------------------------------------------------------------------------------------------------------------------------------------------------------------------------------------------------------------------------------------------------------------------------------------------

>Redeckera_megalocarpum_HG518628

-------CCTAGTAAGCGTGAGTCATCAGCTCATG-TTGATTACGTCCCTGCCCTTTGTACACACCGCCCGTCGCTACTACCGATTGAATGGCTTAGTGAGACCTTTGGATTGAAG-TTTGGGAGCGGCAAC----GTGACCTTACTTCGAGAAGTCGGTCAAACTTGGTCATTTAGAGGAAGTAAAAGTCGTAACAAGGTTTCCGTAGGTGAACCTGCGGAAGGATCATT-AATAATT-TTTT-------------------CCCCCT----T--------TTTGGGGGTTCAATTTTTATTGTATTCAAATTCCCACTCTTT-------AA-AAATTTTAATCATAATAAATTTTATATATAAT-ATAAA-T-GAAAAAAGACAACTTTCAACAACGGATCTCTTGGTTCTCGCATCGATGAAGAACGCAGCGAAATGCGATACGTAGTGTGAATTGCAGGTTTACGTGAATCATCGAA-TCTTCGAACGCAAATTGCACTTCCCAGTAATCTGGGGAGTATGCCTGGTTGAGGGTCATCCAATTAACA-TTCGTGAATTT----------TTTTTTTCG-------------CGGATTTGAG----TTTT-CC--GGTG--TTT-TCACAT---------AA--------GA-GGGATGCTGGTGACTTT-AAAATG-ATTT---------TTTCGGTTCAAGTC--AAAACGTGC-TATGT-GACGTGGGTTCGTT-GAAAACTTGACCT----C--AA----C-ATAT-ATTATGTCTTGCATCGAT----CTCGTTCGAGTGATAGGTGT-CAAGC--ATATAT----TTTTTTTTCATGAC-CTCAACTCAGGCAAGGATACCC-GCTGAACTTAAGCATATCAATAAGCGGAGGAAAAGAAACTAACAAGGATTCCCTTAGTAACGGCGAGTGAAGTGGGAAAAGCTCAAATTTTAAATCTGTC-TGG---TACCACCAGGTCGAGTTGTAATTTGAAGAAGCGCTTTC-GG-TG-TTTTGATCTGGTTAAAGTTCTTTGGAACAAGACATCAT-GG-AGGGTGAGAATCCCGTGCATGATCAGA-TC---GAAAT--AC-TTCAG---ATGCGCTCTCTAAGAGTCGAGTTGTTTGGGAATGCAGCTCAAATTGGG-T-GGTAGAC-TTCACCTAAGGCTAAATATCAGCGAGAGACCGATAGCGAACAAGTACCGTG-AG-GGAAAGATGAAAAGAACTTTGAAAAGAGAGTTAAACAGTACGTGAAATTGTTGAAAGGGAAACGATTGAAGTCAGTCGTGCCGT-TGAGAAATCAGCCTGATGGG-TTTTTGGGTTCGTGGGT--TGAGGTAGGGT---CAAACCACCACCTCTCTTTCGGACTCGATGCTTGCCGGGTGTACTTTCTTT-TTGGCAGGTCAACGTCGATTTT-GGGGGTTGTAAA---------ATAACTGGGGG-AATGTAGCTC--CTTTC--G---GAGTG-TTATAGACCCTGGGGGATGCAACCCGTGGGATCGAGGATTGCAGCAAATGC-------------------TTCAT--TGC-TTGTCGCCTGCCCGCTGG-ACGTCGCCCCGCTTGTGACAATATTTCTTGACACATTGTGGGACTTGTCGGTTACCGGTTAGAGCGATCTAAAA--TTTGCTAAGGATGTTGACGTAATGGCTTCAAACGACCCGTCTTGAAACACGGACCAAGGAG---------------------------------------------------------------------------------------------------------------------------------------------------------------------------------------------------------------------------------------------------------------------------------------------------------------------------------------------------------------------------------------------------------------------------------------------------------------------------------------------------------------------------------------------------------------------------------------------------------------------------------------------------------------------------------------------------------------------------------------------------------------------------------------------------------------------------------------------------------------------------------------------------------------------------------------------------------------------------------------------------------------------------------------------------------------------------------------------------------------------------------------------------------------------------------------------------------------------------

>Redeckera_megalocarpum_HG518629

-------CCTAGTAAGCGTGAGTCATCAGCTCATG-TTGATTACGTCCCTGCCCTTTGTACACACCGCCCGTCGCTACTACCGATTGAATGGCTTAGTGAGACCTTTGGATTGAAG-TTTGGGAGCGGCAAC----GTGACCTTACTTCGAGAAGTCGGTCAAACTTGGTCATTTAGAGGAAGTAAAAGTCGTAACAAGGTTTCCGTAGGTGAACCTGCGGAAGGATCATT-AATAATT-TTTT-------------------CCCCCT----T--------TTTGGGGGTTCAATTTTTATTGTATTCAAATTCCCACTCTTT-------AA-AAATTTTAATCATAATAAATTTTATATATAAT-ATAAA---TGAAAAAGACAACTTTCAACAACGGATCTCTTGGTTCTCGCATCGATGAAGAACGCAGCGAAATGCGATACGTAGTGTGAATTGCAGGTTTACGTGAATCATCGAA-TCTTCGAACGCAAATTGCACTTCCCAGTAATCTGGGGAGTATGCCTGGTTGAGGGTCATCCAATTAACA-TTCGTGAATTT----------TTTTTTTCG-------------CGGATTTGAG----TTTT-CC--GGTG--TTT-TCACAT---------AA--------GA-GGGATGCTGGTGACTTT-AAAATG-ATTT---------TTTCGGTTCAAGTC--AAAACGTGC-TATGT-GACGTGGGTTCGTT-GAAAACTTGACCT----C--AA----C-ATAT-ATTATGTCTTGCATCGAT----CTCGTTCGAGTGATGGGTGT-CAAGC--ATATAT----TTTTTTTTCATGAC-CTCAACTCAGGCAAGGATACCC-GCTGAACTTAAGCATATCAATAAGCGGAGGAAAAGAAACTAACAAGGATTCCCTTAGTAACGGCGAGTGAAGTGGGAAAAGCTCAAATTTTAAATCTGCC-TGG---CACAACCAGGTCGAGTTGTAATTTGAAGAAGCGCTTTC-GG-TG-TTTTGATCTGGTTAAAGTTCTTTGGAACAAGACATCAT-GG-AGGGTGAGAATCCCGTGCATGATCAGA-TC---GAAAT--AC-TCCAG---ATGCGCTCTCTAAGAGTCGAGTTGTTTGGGAATGCAGCTCAAATTGGG-T-GGTAGAC-TTCACCTAAGGCTAAATATCAGCGAGAGACCGATAGCGAACAAGTACCGTG-AG-GGAAAGATGAAAAGAACTTTGAAAAGAGAGTTAAACAGTACGTGAAATTGTTGAAAGGGAAACGATTGAAGTCAGTCGTGCCAT-TGAGAAATCAGCCTGGTGGG-TTTTTGGGTTCGTGGGT--TGAGGTAGGGT---CAA---ACCATCTCTCTTTCGGACTCGATGCTTACCCGGTGTACTTTCTTT-TTGGCAGGTCAACGTCGATTTT-GGGGGTTGTAAA---------ATAACTGGGGG-AATGTAGCTC--CTTTCGGG---GAGTG-TTATAGACCCTGGGGGATGCAACCCGTGGGATCGAGGATTGCAGCAAATGC-------------------TTCAT--CGC-TTGTCGCCTGCCCGCTGG-ACGTCGCCCCGCTTGTGACAATATTTCTTGACACATTGTGGGACTTGTCGGTTACCGGTTAGAGCGATCTAAAA--TTTGCTAAGGATGTTGACGTAATGGCTTCAAACGACCCGTCTTGAAACACGGACCAAGGAG---------------------------------------------------------------------------------------------------------------------------------------------------------------------------------------------------------------------------------------------------------------------------------------------------------------------------------------------------------------------------------------------------------------------------------------------------------------------------------------------------------------------------------------------------------------------------------------------------------------------------------------------------------------------------------------------------------------------------------------------------------------------------------------------------------------------------------------------------------------------------------------------------------------------------------------------------------------------------------------------------------------------------------------------------------------------------------------------------------------------------------------------------------------------------------------------------------------------------

>Siverdingia_tortuosa_JF439094

AGGAATCCCTAGTAAGCGTGAGTCATCAGCTCACG-TTGATTACGTCCCTGCCCTTTGTACACACCGCCCGTCGCTACTACCGATTGAATGGCTTAGTGAGACCTTTGGATTGGG--TTTGGGAACGGCAAC----GTAACCTTATTCCGAGAAGTCGGTCAAACTTGGTCATTTAGAGGAAGTAAAAGTCGTAACAAGGTTTCCGTAGGTGAACCTGCGGAAGGATCATT-AATAATT-TTTTT-------------C---CTTCCAT----T--------TTTTTGGGGGGGA-ATTATTTGTATTCAAA-TTCCACTCTTAAAAATTCCT-AAAT-ACA---------AAATTTATATAACAA-ATATA-A-TAAAAAAGACAACTTTCAACAACGGATCTCTTGGCTCTCGCATCGATGAAGAACGCAGCGAAATGCGATACGTAGTGTGAATTGCAGGTTTACGTGAATCATCGAA-TCTTTGAACGCAAATTGCACTTTCCAGTTTTCTGGGAAGTATGCCTGGTTGAGGGTCGTTAAAATAACA-ATCGTAAA-------------ATTTTTTTA-------------CGGATTTGGG----TATT-CC--GGCG--TTT--------------------------TT-TAAATGCTGGTAACTTT-AAAATG-ATCT--------TTTTTGGTTTAAGTT--AAAACGTTC-TATAATTACAT-GGTTCGTT-GAAAACTTACCTAA-TAT--GT----T-ATAT-ATTATGTT-TACACTTGT----CATTTTA-ATCGATTCGTGC--AAGC--ATATA------TTTTTTTTATGAC-CTCAACTCAAGCAAGATTACCC-GCTGAACTTAAGCATATCAATAAGCGGAGGAAAAGAAACTAACAAGGATTCCCTTAGTAACGGCGAGTGAAGTGGGAAAAGCTCAAATTTTAAATCTACC-TGG---TTT--CCAGGTCGAATTGTAATTTGAAGAAGCGTTTTC-GG-TG-TTTCGATCTGGTTGAAGTTCTTTGGAACAAGACATCAT-GG-AGGGTGAGAATCCCGTGCATGATCAGA-TC---GAGAT--AC-TCCAG---ATACGCTCTCTAAGAGTCGAGTTGTTTGGGAATGCAGCTCAAAATGGG-T-GGTAGAC-TTCACCTAAGGCTAAATATCAGCGAGAGACCGATAGCGAACAAGTACCGTG-AG-GGAAAGATGAAAAGAACTTTGAAAAGAGAGTTAAATAGTACGTGAAATTGTTGAAAGGGAAACGATTGAAGTCAGTCGTGCCGC-TGAGAAATCGACTTAGCGAG-TTTCA-GTTTTT-AATT--CGAGATAGGGT---CAA---ACCATCACGGTTTTTAAATTGGGACTTGTTGAGTGTACTTTCTTT-TTGGCAGGTCAGTGTCGATTTT-GGGGGTTGTAAA---------ATGACTGGGGG-AATGTAGCTC--CCTTCGGG---GAGTG-TTATAGACCCTGGGGGATGCAGCCCGCGAGATCGAGGATTGCAGCAAATGC---------------------TTC--AGC-TTGTCGTCTATCCGTTAA-ACGTCGCCCTGCTTGTGACAATATT-CTTGACACTTTGTGGGACTTGGCGGTTATAGGTTAGAACGTTCTAAAA--TTTGCTAAGGATGCTGACGTAATGGCTTTAAACGACCCGTCTTGAAACACGGACCAAGGAGTCTAACATGTATGCGAG----------------------------------------------------------------------------------------------------------------------------------------------------------------------------------------------------------------------------------------------------------------------------------------------------------------------------------------------------------------------------------------------------------------------------------------------------------------------------------------------------------------------------------------------------------------------------------------------------------------------------------------------------------------------------------------------------------------------------------------------------------------------------------------------------------------------------------------------------------------------------------------------------------------------------------------------------------------------------------------------------------------------------------------------------------------------------------------------------------------------------------------------------------------------------------------------------------

>Siverdingia_tortuosa_JF439096

AGGAATCCCTAGTAAGCGTGAGTCATCAGCTCACG-TTGATTACGTCCCTGCCCTTTGTACACACCGCCCGTCGCTACTACCGATTGAATGGCTTAGTGAGACCTTTGGATTGGG--TTTGGGAACGGCAAC----GTAACCTTATTCCGAGAAGTCGGTCAAACTTGGTCATTTAGAGGAAGTAAAAGTCGTAACAAGGTTTCCGTAGGTGAACCTGCGGAAGGATCATT-AATAATT-TTTTT-------------C---CTTCCAT----T--------TTTTTGGGGGGGA-ATTATTTGTATTCAAA-TTCCACTCTTAAAAATTCCT-AAAT-ACA---------AAATTTATATAACAA-ATATA-A-TAAAAAAGACAACTTTCAACAACGGATCTCTTGGCTCTCGCATCGATGAAGAACGCAGCGAAATGCGATACGTAGTGTGAATTGCAGGTTTACGTGAATCATCGAA-TCTTTGAACGCAAATTGCACTTTCCAGTTTTCTGGGAAGTATGCCTGGTTGAGGGTCGTTAAAATAACA-ATCGTAAA-------------ATTTTTTTA-------------CGGATTTGGG----TATT-CC--GGCG--TTT--------------------------TT-TAAATGCTGGTAACTTT-AAAATG-ATCT--------TTTTTGGTTTAAGTT--AAAACGTTC-TATAATTACAT-GGCTCGTT-GAAAACTTACCTAA-TAT--GT----T-ATAT-ATTATGTT-TACACTTGT----CATTTTA-ATCGATTCGTGC--AAGC--ATATA------TTTTTTTTATGAC-CTCAACTCAAGCAAGATTACCC-GCTGAACTTAAGCATATCAATAAGCGGAGGAAAAGAAACTAACAAGGATTCCCTTAGTAACGGCGAGTGAAGTGGGAAAAGCTCAAATTTTAAATCGACC-TGG---TTT--CCAGGTCGAATTGTAATTTGAAGAAGCG-TTTC-GG-TG-TTTCGATCTGGTTAAAGTTCTTTGGAACAAGACATCAT-GG-AGGGTGAGAATCCCGTGCATGATCAGA-TC---GAGAT--AC-TCCAG---ATACGCTCTCTAAGAGTCGAGTTGTTTGGGAATGCAGCTCAAAATGGG-T-GGTAGAC-TTCACCTAAGGCTAAATATCAGCGAGAGACCGATAGCGAACAAGTACCGTG-AG-GGAAAGATGAAAAGAACTTTGAAAAGAGAGTTAAATAGTACGTGAAATTGTTGAAAGGGAAACGATTGAAGTCAGTCGTGCCGT-TGAGAAATCAACTTAGCGAG-TTTCA-GTTTTTGAATC--TTTGATAGGGT---CAA---ACCATCAAGGTTTTTAAGTTGGGACTTGTTGAGTGTACTTTCTTT-TTGGCAGGTCAGCGTCGATTTT-GGGGGTTGTAAA---------ATGACTGGGAG-AATGTAGCTC--CCTTCGGG---GAGTG-TTATAGACCCTGGGGGATGCAGCCCGCGGGATCGAGGATTGCAGCAAATGC---------------------TTC--AGC-TTGTCGTCTATCCGTTAA-ACGTTACCCTGCTTGTGACAATATT-CTTGACACTTTGTGGGACTTGACGGTTATAGGTTAGAACGTTCTAAAA--TTTGCTAAGGATGCTGACGTAATGGCTTTAAACGACCCGTCTTGAAACACGGACCAAGGAGTCTAACATGTGTGCGAG----------------------------------------------------------------------------------------------------------------------------------------------------------------------------------------------------------------------------------------------------------------------------------------------------------------------------------------------------------------------------------------------------------------------------------------------------------------------------------------------------------------------------------------------------------------------------------------------------------------------------------------------------------------------------------------------------------------------------------------------------------------------------------------------------------------------------------------------------------------------------------------------------------------------------------------------------------------------------------------------------------------------------------------------------------------------------------------------------------------------------------------------------------------------------------------------------------

>Siverdingia_tortuosa_JF439095

AGGAATCCCTAGTAAGCGTGAGTCATCAGCTCACG-TTGATTACGTCCCTGCCCTTTGTACACACCGCCCGTCGCTACTACCGATTGAATGGCTTAGTGAGACCTTTGGATTGGG--TTTGGGAACGGCAAC----GTAACCTTATTCCGAGAAGTCGGTCAAACTTGGTCATTTAGAGGAAGTAAAAGTCGTAACAAGGTTTCCGTAGGTGAACCTGCGGAAGGATCATT-AATAATT-TTTT---------------------CCAT----C--------CATTTTTGGGGGA-ATTATTTGTATTCAAA-TTCCACTCTTA--AATTTTT-AAAA-ATA---------AAATTTATATAACAA-ATAT------AAAAAGACAACTTTCAACAACGGATCTCTTGGCTCTCGCATCGATGAAGAACGCAGCGAAATGCGATACGTAGTGTGAATTGCAGGTTTACGTGAATCATCGAA-TCTTTGAACGCAAATTGCACTTTCCAGTACTCTGGGAAGTATGCCTGGTTGAGGGTCGTTAAAACAACA-ATCGTAAA-------------A-TTTTTTA-------------CGGATTTGGG----TTTT-CC--GGCA--TTT--------------------------TT-TAAATGCTGGTAACTTT-AAAATG-ATCT-------TTTTTTGGTTCAAGTT--AAAACGTTC-TATATTTACAT-GGTTCGTT-GAAAACTTACCTAA-TAT--GT----T-ATAT-ATTATGTT-TACACTTGT----CATTTTA-ATCGATTCGTGC--AAGC--ATATA------TTTTTTTTATGAC-CTCAACTCAAGCAAGATTACCC-GCTGAACTTAAGCATATCAATAAGCGGAGGAAAAGAAACTAACAAGGATTCCCTTAGTAACGGCGAGTGAAGTGGGAAAAGCTCAAATTTTAAATCTACC-TGG---TTT--CCAGGTCGAATTGTAATTTGAAGAAGCGTTTTC-GG-TG-TTTCGATCTGGTTGAAGTTCTTTGGAACAAGACATCAT-GG-AGGGTGAGAATCCCGTGCATGATCAGA-TC---GAGAT--AC-TCCAG---ATACGCTCTCTAAGAGTCGAGTTGTTTGGGAATGCAGCTCAAAATGGG-T-GGTAGAC-TTCACCTAAGGCTAAATATCAGCGAGAGACCGATAGCGAACAAGTACCGTG-AG-GGAAAGATGAAAAGAACTTTGAAAAGAGAGTTAAATAGTACGTGAAATTGTTGAAAGGGAAACGATTGAAGTCAGTCGTGCCGT-TGAGAAATCAACTTAGCGAG-TTTCA-GTTTTTGAATC--TTTGATAGGGT---CAA---ACCATCAAGGTTTTTAAGTTGGGACTTGTTGAGTGTACTTTCTTT-TTGGCAGGTCAGCGTCGATTTT-GGGGGTTGTAAA---------ATGACTGGGAG-AATGTAGCTC--CCTTCGGG---GAGTG-TTATAGACCCTGGGGGATGCAGCCCGCGGGATCGAGGATTGCAGCAAATGC---------------------TTC--AGC-TTGTCGTCTATCCGTTAA-ACGTTACCCTGCTTGTGACAATATT-CTTGACACTTTGTGGGACTTGACGGTTATAGGTTAGAACGTTCTAAAA--TTTGCTAAGGATGCTGACGTAATGGCTTTAAACGACCCGTCTTGAAACACGGACCAAGGAGTCTAACATATATGCGAG----------------------------------------------------------------------------------------------------------------------------------------------------------------------------------------------------------------------------------------------------------------------------------------------------------------------------------------------------------------------------------------------------------------------------------------------------------------------------------------------------------------------------------------------------------------------------------------------------------------------------------------------------------------------------------------------------------------------------------------------------------------------------------------------------------------------------------------------------------------------------------------------------------------------------------------------------------------------------------------------------------------------------------------------------------------------------------------------------------------------------------------------------------------------------------------------------------

>Desertispora_omaniana_KF154770_MG459206

AGGAATCCCTAGTAAGCGTGAGTCATCAGCTCACG-TTGATTACGTCCCTGCCCTTTGTACACACCGCCCGTCGCTACTACCGATTGAATGGCTTAGTGAGACCTTTGGATTGGG--TTCGGGGATTGGAAA---CATTTCCCTTATTCGAGAAGTTGGTCAAACTTGGTCATTTAGAGGAAGTAAAAGTCGTAACAAGGTTTCCGTAGGTGAACCTGCGGAAGGATCATT-AGAAATT-TAATT-CCCGGGAATTCT--------TGG----T--------TCCCGG-G------GGTATTTGTATTCAAA-TTCCACTCTTT-------AA-AAAA-TTA-------------ATACATTTAAT--AAAA-T-AAAATGAGACAACTTTCAACAACGGATCTCTTGGCTCTCGCATCGACGAAGAACGCAGCGAAATGCGATACGTAGTGTGAATTGCAG-TTTACGTGAATCAACGAA-TCTTCGAACGCAAATTGCACTTTCCAGTAATCTGGGGAGTATGCCTGGTTGAGGGTCATTGAAACAAGA-TCGCGAAATTTATTTTTTTTATTTATTTCG-------------CGGATTTGAG----TTTT-CC--GGAG--ATC-ATACATATGT------A--------TG-TGATTACCGGTGACTTT-AAAATG-AATT-----------CTGGTTCAAGTC-AAAAACGTTC-TATGCAATCGT-GGTTCGTT-GACAACTTGACCT--T-C--AT----T-ATGT-TCCCCACC-TTCTCGGAG------------ATTGGTGCGCGG---GAC--ATATA-----ATTTTTTTTATGAC-CTCAGGTCAGGCAAGGAAACCC-GCTGAACTTAAGCATATCAATAAGCGGAGGAAAAGAAACTAACAAGGATTCCCTCAGTAACGGCGAGTGAAGTGGGAAAAGCTCAAATTTTAAATCTACC-TGGTA-CCC--CCAGGTCGAGTTGTAATTTGAAGAAGCGTTTTC-GG-TG-CTTCGGTCTGGTCCAAGTCCTTTGGAACAAGGCATCAC-GG-AGGGTGAGAATCCCGTGCATGGTCAGA-CC---CGAAG--TT-CCAATCAGATACGCTCTCGAAGAGTCGAGTTGTTTGGGAATGCAGCTCAAATTGGG-T-GGTAGAC-TTCACCTAAGGCTAAATATCAGCGAGAGACCGATAGCGAACAAGTACCGTG-AG-GGAAAGATGAAAAGAACTTTGAAAAGAGAGTTAAACAGTACGTGAAATTGTTGAAAGGGAAACGATTGAAGTCAGTCGTGCCGA-TGAGAAATCAACTTGACGGG-TTTCGGATTTCTTGGGT--CGAGTCAGGGT---CAA---ACCGTCTCTCCCTTGAATTCGGGATTTGTTGGGTGTACTTTCTTTGTTGGAAGGTCAGCGTCGATTTC-GGGGGTCGTAAA---------ATAACTGGGGG-AATGTAGCTC--CCTTCGGG---GAGTG-TTATAGACCCTGGGGGATGCGGTCCGCGAGATCGAGGATTGCAGCAAATGC-------------------TCTCTG-GGC-TTGTCGCCTATCACCTGG-AAGTCGCCCCGCGGGTGACAACATT-CTTGACATTCGTGGGTCTTGACGACCA-CGGGTTAGAGCGTTCGGAAA-TTTTGCTAAGGATGCTGACGTAATGGCTTTAAACGACCCGTCTTGAAACACGGACCAAGGAGTCTAACATATGTGCGAG------------------------------------------------------------------------------gtggagaagatgatttgacacacaaattgtcggacattttgaaggccaatcaaaacgtaaaacgttatgaagctgatggtcatcccccacacgtcgtaaacgaatttgaggctttgttacaggtatcttgactccttctctctttctctcttctcttccctctctcttctcttcctcttccctcttccctctctcttcctcttccctctctcttcctctccccccccccttcccccttccctga-ccattttcaatc-a-tcaaattcactcatg--ttttca--ttattacttatt-catatcacgcgg-----gaaaaatcagtttcattgtgcaacttatatggacaatgaaatggccggtcaacctcaagcccttcagaaatccggtagacctttaaagtcaatacgtgcgcgacttaagggtaaggaaggtcggttacgtgggaacttgatggggaagcgagtagatttctccgcccgtacagttatcacgggggacccaaatatttcagttgatgaggtaggggtaccgaagagcatagctcaaaacttgaccttcccggaattggtgacccccttcaacattgattaccttcaaaggttggtggagaatggtccctccacccaccccggagccaagtacgtgattagggataccggtgaaaggatcgaccttaaacatatttccggtatgaccggtgggttaaggttacactacggttggaaggttgaacgtcacctcgtcgacggtgacatcgtcatattcaatcgtcaaccatctctacacaaaatgtcaatgat----------------------------------------------------------------------------------------------------------------------------------------------------------------------------------------------------------------------------------------------------------------------------------------

>Desertispora_omaniana_MG459208_MG459194

AGGAATCCCTAGTAAGCGTGAGTCATCAGCTCACG-TTGATTACGTCCCTGCCCTTTGTACACACCGCCCGTCGCTACTACCGATTGAATGGCTTAGTGAGACCTTTGGATTGGG--TTTGGAATTGTGCAA---ACTTTTCCTTATCCGAGAAGTTGGTCAAACTTGGTCATTTAGAGGAAGTAAAAGTCGTAACAAGGTTTCCGTAGGTGAACCTGCGGAAGGATCATT-AGAAATT-TAATT-CCCGGGAATTCT--------TGG----T--------TCCCGG-G------GGTATTTGTATTCAAA-TTCCACTCTTT-------AA-AAAA-TTA-------------ATACATTTAAT--AAAA-T-AAAATGAGACAACTTTCAACAACGGATCTCTTGGCTCTCGCATCGATGAAGAACGCAGCGAAATGCGATACGTAGTGTGAATTGCAG-TTTACGTGAATCATCGAA-TCTTCGAACGCAAATTGCACTTTCCAGTAATCTGGGGAGTATGCCTGGTTGAGGGTCATTGAAACAAGA-TCGCGAAATTTATTTTTTTTATTTATTTCG-------------CGGATTTGAG----TTTT-CC--GGAG--ATC-ATACATATGT------A--------TG-TGATTACCGGTGACTTT-AAAATG-AATT-----------CTGGTTCAAGTC-AAAAACGTTC-TATGCAATCGT-GGTTCGTT-GACAACTTGACCT--T-C--AT----T-ATGT-TCCCCACC-TTCTCGGAG------------ATTGGTGCGCGG---GAC--ATATA-----ATTTTTTTTATGAC-CTCAGCTCAGGCAAGGAAACCC-GCTGAACTTAAGCATATCAATAAGCGGAGGAAAAGAAACTAACAAGGATTCCCTCAGTAACGGCGAGTGAAGTGGGAAAAGCTCAAATTTTAAATCTACC-TGGTA-CCC--CCAGGTCGAGTTGTAATTTGAAGAAGCGTTTTC-GG-TG-CTTCGGTCTGGTCCAAGTCCTTTGGAACAAGGCATCAC-GG-AGGGTGAGAATCCCGTGCATGGTCAGA-CC---CGAAG--TT-CCAATCAGATACGCTCTCGAAGAGTCGAGTTGTTTGGGAATGCAGCTCAAATTGGG-T-GGTAGAC-TTCACCTAAGGCTAAATATCAGCGAGAGACCGATAGCGAACAAGTACCGTG-AG-GGAAAGATGAAAAGAACTTTGAAAAGAGAGTTAAACAGTACGTGAAATTGTTGAAAGGGAAACGATTGAAGTCAGTCGTGCCGA-TGAGAAATCAACTTGACGGG-TTTCGGATTTCTTGGGT--AGAGTCAGGGT---TAA---ACCGTCTCTCCCTTGAATTCGGGATTTGTTGGGTGTACTTTCTTTGTTGGCAGGTCAGCGTCGATTTC-GGGGGTCGTAAA---------ATAACTGGGGG-AATGTAGCTC--CCTTCGGG---GAGTG-TTATAGACCCTGGGGGATGCGGTCCGCGAGATCGAGGATTGCAGCAAATGC-------------------TCTCTG-GGC-TTGTCGCCTATCACCTGG-AAGTCGCCCCGCGGGTGACAACATT-CTTGACATTCGTGGGTCTTGACGACCA-CGGGTTAGAGCGTTCTGAAA-TTTTGCTAAGGATGCTGACGTAATGGCTTTAAACGACCCGTCTTGAAACACGGACCAAGGAGTCTAACATATGTGCGAG------------------------------------------------------------------------------gtggagaagatgatttgacacacaaattgtcggacattttgaaggccaatcaaaacgtaaaacgttatgaagctgatggtcatcccccacacgtcgtaaacgaatttgaggctttgttacaggtatcttgactccttctctctttctctcttctcttccctctctcttctcttcctcttccctcttccctctctcttcctcttccctctctcttcctctccccccccccttcccccttccctga-ccattttcaatc-a-tcaaattcactcatg--ttttca--ttattacttatt-catatcacgcgg-----gaaaaatcagtttcattgtgcaacttatatggacaatgaaatggccggtcaacctcaagcccttcagaaatccggtagacctttaaagtcaatacgtgcgcgacttaagggtaaggaaggtcggttacgtgggaacttgatggggaagcgagtagatttctccgcccgtacagttatcacgggggacccaaatatttcagttgatgaggtaggggtaccgaagagcatagctcaaaacttgaccttcccggaattggtgacccccttcaacattgattaccttcaaaggttggtggagaatggtccctccacccaccccggagccaagtacgtgattagggataccggtgaaaggatcgaccttaaacatatttccggtatgaccggtgggttaaggttacactacggttggaaggttgaacgtcacctcgtcgacggtgacatcgtcatattcaatcgtcaaccatctctacacaaaatgtcaatgatgg--------------------------------------------------------------------------------------------------------------------------------------------------------------------------------------------------------------------------------------------------------------------------------------

>Desertispora_omaniana_KF154769

AGGAATCCCTAGTAAGCGTGAGTCATCAGCTCACG-TTGATTACGTCCCTGCCCTTTGTACACACCGCCCGTCGCTACTACCGATTGAATGGCTTAGTGAGACCTTTGGATTGGG--TTTGGAGTTGTGCAA---ACTTTTCCTTATCCGAGAAGTTGGTCAAACTTGGTCATTTAGAGGAAGTAAAAGTCGTAACAAGGTTTCCGTAGGTGAACCTGCGGAAGGATCATT-AGAAATT-TAATT-CCCGGGAATTCT--------TGG----T--------TCCCGG-G------GGTATTTGTATTCAAA-TTCCACTCTTT-------AA-AAAA-TTA-------------ATACATTTAAT--AAAA-T-AAAATGAGACAACTTTCAACAACGGATCTCTTGGCTCTCACATCGATGAAGAACGCAGCGAAATGCGATACGTAGTGTGAATTGCAG-TTTACGTGAATCATCGAA-TCTTCGAACGCAAATTGCACTTTCCAGTAATCTGGGGAGTATGCCTGGTTGAGGGTCATTGAAACAAGA-TCGCGAAATTTATTTTTTTTATTTATTTCG-------------CGGATTTGAG----TTTT-CC--GGAG--ATC-ATATGTATGTGTGGTAA--------TG-TGATTACCGGTGACTTTAAAAATG-AATT-----------CTGGTTCAAGTC-AAAAACGTTC-TATGCAATCGT-GGTTTGTT-GACAACTTGTCCT--T-C--AT----T-ATGT-TCCCCACC-TAATCGGAG------------ATAGGTGCGCGG---GAC--ATATAA----TTTTTTTTTATGACTCTCAGGCTCAGGCAGGTTACCC-GCTGAACTTAAGCCTATCAATAAGCGGAGGAAAAGAAACTAACAAGGATTCCCTCAGTAACGGCGAGTGAAGTGGGAAAAGCTC-AATTTTAAATCTACC-TGGTATCCC--CCAGGTCGAGTTGTAATTTGAAGAAGCGTTTTC-GG-TG-CTTCGGTCTGGTCCAAGTCCTTTGGAACAAGGCATCAC-GG-AGGGTGAGAATCCCGTGCATGGTCAGA-CC---CGAAG--TT-CCAATCAGATACGCTCTCGAAGAGTCGAGTTGTTTGGGAATGCAGCTCAAATTGGG-T-GGTAGAC-TTCACCTAAGGCTAAATATCAGCGAGAGACCGATAGCGAACAAGTACCGTG-AG-GGAAAGATGAAAAGAACTTTGAAAAGAGAGTTAAACAGTACGTGAAATTGTTGAAAGGGAAACGATTGAAGTCAATCGTGCCGA-TGAGAAATCAACTTGATGGG-TTTCGGATTTCTTGGGT--TGAGTCAGGGT---CAA---ACCGTCTCTCCCTTGAATTCGGGACTTGGTGGGTGTACTTTCTTTGTTGGCAGGTCAGCGTCGATTTC-GGGGGTCGTAAA---------ATAACTGGGGG-AATGTAGCTC--CCTTCGAG---GAGTG-TTATAGACCCTGGGGGATGCGGTCCGCGAGATCGAGGATTGCAGCAAATGC-------------------TCTCTG-GGCTTTGTCGCCTATCACCTGG-AAGTCGCCCCGCGGGTGACAATATT-CTTGACATTCGTGGGTCTTGACGACCA-CGGGTTAGAGCGTTCTGAAA-TTTTGCTAAGGATGCTGACGTAATGGCTTTAAACGACCCGTCTTGAAACACGGACCAAGGAGTCTAACATATGTGCGAG------------------------------------------------------------------------------gtggagaagatgatttgacacacaaattgtcggacattttgaaggccaatcaaaacgtaaaacgttatgaagctgatggtcatcccccacacgtcgtaaacgaatttgaggctttgttacaggtatcttgactccttctctctttctctcttctcttccctctctcttctcttcctcttccctcttccctctctcttcctcttccctctctcttcctctccccccccccttcccccttccctga-ccattttcaatc-a-tcaaattcactcatg--ttttca--ttattacttatt-catatcacgcgg-----gaaaaatcagtttcattgtgcaacttatatggacaatgaaatggccggtcaacctcaagcccttcagaaatccggtagacctttaaagtcaatacgtgcgcgacttaagggtaaggaaggtcggttacgtgggaacttgatggggaagcgagtagatttctccgcccgtacagttatcacgggggacccaaatatttcagttgatgaggtaggggtaccgaagagcatagctcaaaacttgaccttcccggaattggtgacccccttcaacattgattaccttcaaaggttggtggagaatggtccctccacccaccccggagccaagtacgtgattagggataccggtgaaaggatcgaccttaaacatatttccggtatgaccggtgggttaaggttacactacggttggaaggttgaacgtcacctcgtcgacggtgacatcgtcatattcaatcgtcaaccttctctacacaaaatgtcaatgatggg-------------------------------------------------------------------------------------------------------------------------------------------------------------------------------------------------------------------------------------------------------------------------------------
