# Supplementary material for: Three new species of arbuscular mycorrhizal fungi (Glomeromycota) and Acaulospora gedanensis revised
Source: Front Microbiol. 2024 Feb 12;15:1320014. doi: 10.3389/fmicb.2024.1320014 (PMC10896085; doi:10.3389/fmicb.2024.1320014)
Supplement: Supplementary Table 3 — An alignment used to produce Figure 3. [file Table_3.DOCX]

>431_1

CTCTTTAACGAGGAATCCCTAGTAAGCATGAGTCATCAGCTCATGCTGATTACGTCCCTGCCCTTTGTACACACCGCCCGTCGCTACTACCGATTGAATGGCTTAGTGAGACCCTCGGATCGACTCGCGGAAGCTTTAACCGGCATCCGTTTGTTGAGAAGTTGGTCAAACTTGGTCATTTAGAGGAAGTAAAAGTCGTAACAAGGTTTCCGTAGGTGAACCTGCGGAAGGATCATT-AAAA-ATATGAGGTATTT-ATACCTCTT-------------TTGTATTTAAAACCAA--CTCTTTA--------------AAACTTATTTTTTTTT------AATAAAAATAAAAACTTTCAACAACGGATCTC-TTGGCTCTCGCATCGATGAAGAACGCAGCGAAATGCGAAAAGTAATGTGAATTGCAGAATTCCGTGAATCATTAAATCTTTGAACGCAAATTGCACTCCTTGGTATTCCGAGGAGTACACTTGCTTGAGGGTCAGTTAAATAAA-------ATATCGTAACATCTTTTTTGGTGTAGCGGATCTGAGTTATCCGATTTT-AAATCGGTTACTTAAAATTAAAGATTAT--ATATAATGTGATATGTACTAAAAT-AAAGGT--------CGTTAATCATTATT-AAAACTTA--ATATATATATTGCCTCTAAT----------TTTTTTTTTTTAGTTGGGTAGT-----TATATAATTTCTGAGAATGACCTCAAGTCAAGTGAGAGTA-CCCGCTGAACTTAAGCATATCAATAA----GCGGAGGAAAAGAAACTAACAAGGATTCCCCTAGTAACGGCGAGCGAAGCGGGAAGAGCACAAATTTTAAATCTATCTGG-TTTACCAGGTCGAGTTGTAATTTGAAGAAATGTTTTTAATGTTCCGGGTTGGTTTAAATCCTTTGGGATAAGG-TATCATGGAGGGTGAGAATCCCGTGTAT-ATCAACCGCTGGGATGTTAT-AAATACATTTTCTAAGAGTCGAGTTGTTTGGGAATGCAGCTCTAAATGGGTGGTAAATTTCACCTAATGCTAAATATAAGCGAGAGACCGATAGCGAACAAGTACCGTGAGGGAAAGATGAAAAGAACTTTGAAAAGAGAGTTAAATAGTACGTGAAATTGTTGAAAGGGAAACGATTGAAGTCAGTCATGCTAGCGGGAATC----------------------------------AACTT---------------------GGAATAACTTTCAAGTGCATTTCTTCGCTTGGCAGGTTAGCGTCGATTTTGAACGTCATAAAATGATTGGGGGAAGGTAGCT-CT-------TTCGAGAGTGTTATAGCCCTTGGTAAATGTGATGTTTAGGATCGAGGTTTGCAACGGATACCTTTTA-GGCTAGCCGCCTGGCCTCTGATTCGATGTCGGGTTATAGACAGCATGCTGACTATGATCTGAT-TATTGGTCAAAAGGTTAGAGCGAGCATAAATTCGTTAAGGACGCTGACGTAATGG

>431_3

CTCTTTAACGAGGAATCCCTAGAAAGCATGAGTCATCAGCTCATGCTGATTACGTCCCTGCCCTTTGTACACACCGCCCGTCGCTACTACCGATTGAATGGCTTAGTGAGACCCTCGGATCGACTCGCGGAAGCTTTAACCGGCATCCGTTTGTTGAGAAGTTGGTCAAACTTGGTCATTTAGAGGAAGTAAAAGTCGTAACAAGGTTTCCGTAGGTGAACCTGCGGAAGGATCATT-AAAA-ATATGAGGTATTT-ATACCTCTT-------------TTGTATTTAAAACCAA--CTCTTTA--------------AAACTTATTTTTTTTT------AATAAAAATAAAAACTTTCAACAACGGATCTC-TTGGCTCTCGCATCGATGAAGAACGCAGCGAAATGCGAAAAGTAATGTGAATTGCAGAATTCCGTGAATCATTAAATCTTTGAACGCAAATTGCACTCCTTGGTATTCCGAGGAGTACACTTGCTTGAGGGTCAGTTAAATAAA-------ATATCGTAACATCTTTTTTGGTGTAGCGGATCTGAGTTATCCGATTTT-AAATCGGTTACTTAAAATTAAAGATTAT--ATATAATGTGATATGTACTAAAAT-AAAGGT--------CGTTAATCATTATT-AAAACTTAATATATATATATTGCCTCTAAT----------TTTTTTTTTTTAGTTGGGTAGT-----TATATAATTTCTGAGAATGACCTCAAGTCAAGTGAGAGTA-CCCGCTGAACTTAAGCATATCAATAA----GCGGAGGAAAAGAAACTAACAAGGATTCCCCTAGTAACGGCGAGCGAAGCGGGAAGAGCACAAATTTTAAATCTACCTGG-TTTACCGGGTCGAGTTGTAATTTGAAGAAATGTTTTTAATGTTCCGGGTTGGTTTAAATCCTTTGGGATAAGG-TATCATGGAGGGTGAGAATCCCGTGTAT-ATCAACCGCTGGGATGTTAT-AAATACATTCTCTAAGAGTCGAGTTGTTTGGGAATGCAGCTCTAAATGGGTGGTAAATTTCACCTAATGCTAAATATAAGCGAGAGACCGATAGCGAACAAGTACCGTGAGGGAAAGATGAAAAGAACTTTGAAAAGAGAGTTAAATAGTACGTGAAATTGTTGAAAGGGAAACGATTGAAGTCAGTCATGCTGGTGAGAATC----------------------------------AACTT---------------------GGAATAACTTTCAAGTGCATTTCTTCGCTTGGCAGGTTAGCGTCGATTTTGAACGTCATAAAATGATTGGGGGAAGGTAGCT-CT-------TTCGAGAGTGTTATAGCCCTTGGTAAATGTGATGTTTAGGATCGAGGTTTGCAACGGATACCTTTTA-GGCTAGCCGCCTGGCCTCTGATTCGATGTCGGGTTATAGACAGCATGCTGACTATGATCTGAT-TATTGGTCAAAAGGTTAGAGCGAGCATAAATTCGTTAAGGACGCTGACGTAATGG

>431_5_SSU_ITS_LSU_25_06_2020

CTCTTTAACGAGGAATCCCTAGTAAGCATGAGTCATCAGCTCATGCTGATTACGTCCCTGCCCTTTGTACACACCGCCCGTCGCTACTACCGATTGAATGGCTTAGTGAGACCCTCGGATCGACTCGCGGAAGCTTTAACCGGCATCCGTTTGTTGAGAAGTTGGTCAAACTTGGTCATTTAGAGGAAGTAAAAGTCGTAACAAGGTTTCCGTAGGTGAACCTGCGGAAGGATCATT-AAAA-ATATGAGGTATTT-ATACCTCTT-------------TTGTATTTAAAACCAA--CTCTTTA--------------AAACTTATTTTTTTTT------AATAAAAATAAAAACTTTCAACAACGGATCTC-TTGGCTCTCGCATCGATGAAGAACGCAGCGAAATGCGAAAAGTAATGTGAATTGCAGAATTCCGTGAATCATTAAATCTTTGAACGCAAATTGCACTCCTTGGTATTCCGAGGAGTACACTTGCTTGAGGGTCAGTTAAATAAA-------ATATCGTAACATCTTTTTTGGTGTAGCGGATCTGAGTTATCCGATTTT-AAATCGGTTACTTAAAATTAAAGATTAT--ATATAATGTGATATGTACTAAAAT-AAAGGT--------CGTTAATCATTATT-AAAACTTAATATATATATATTGCCTCTAAT----------TTTTTTTTTTTAGTTGGGTAGT-----TATATAATTTCTGAGAATGACCTCAAGTCAAGTGAGAGTA-CCCGCTGAACTTAAGCATATCAATAA----GCGGAGGAAAAGAAACTAACAAGGATTCCCCTAGTAACGGCGAGCGAAGCGGGAAGAGCACAAATTTTAAATCTACCTGG-TTTACCGGGTCGAGTTGTAATTTGAAGAAATGTTTTTAATGTTCCGGGTTGGTTTAAATCCTTTGGGATAAGG-TATCATGGAGGGTGAGAATCCCGTGTAT-ATCAACCGCTGGGATGTTAT-AAATACATTCTCTAAGAGTCGAGTTGTTTGGGAATGCAGCTCTAAATGGGTGGTAAATTTCACCTAATGCTAAATATAAGCGAGAGACCGATAGCGAACAAGTACCGTGAGGGAAAGATGAAAAGAACTTTGAAAAGAGAGTTAAATAGTACGTGAAATTGTTGAAAGGGAAACGATTGAAGTCAGTCATGCTGGTGAGAATC----------------------------------AACTT---------------------GGAATAACTTTCAAGTGCATTTCTTCGCTTGGCAGGTTAGCGTCGATTTTGAACGTCATAAAATGATTGGGGGAAGGTAGCT-CT-------TTCGAGAGTGTTATAGCCCTTGGTAAATGTGATGTTTAGGATCGAGGTTTGCAACGGATACCTTTTA-GGCTAGCCGCCTGGCCTCTGATTCGATGTCGGGTTATAGACAGCATGCTGACTATGATCTGAT-TATTGGTCAAAAGGTTAGAGCGAGCATAAATTCGTTAAGGACGCTGACGTAATGG

>431_4_SSU_ITS_LSU_25_06_2020

CTCTTTAACGAGGAATCCCTAGTAAGCATGAGTCATCAGCTCATGCTGATTACGTCCCTGCCCTTTGTACACACCGCCCGTCGCTACTACCGATTGAATGGCTTAGTGAGACCCTCGGATCGACTCGCGGAAGCTTTAACCGGCATCCGTTTGTTGAGAAGTTGGTCAAACTTGGTCATTTAGAGGAAGTAAAAGTCGTAACAAGGTTTCCGTAGGTGAACCTGCGGAAGGATCATTAAAAA-ATATGGGGTATTT-ATACCTCTT-------------TTGTATTTAAAACCAA--CTCTTTT--------------AAACTTA--TTTTTTT------AATAAAAATAAAAACTTTCAACAACGGATCTCTTTGGCTCTCGCATCGATGAAGAACGCAGCGAAATGCGAAAAGTAATGTGAATTGCAGAATTCCGTGAATCATTAAATCTTTGAACGCAAATTGCACTCCTTGGTATTCCGAGGAGTACACTTGCTTGAGGGTCAGTTAAATAAA-------ATATCGTAACATCTTTTTTGGTGTAGCGGATCTGAGTTATCCGATTTT-AAATCGGTTACTTAAAATTAAAGATTAT--ATATAATGTGATATGTACTAAAAT-AAAGGT--------CGTTAATCATTATT-AAAACTTAATATATATATATTGCCTCTAAT----------TTTTTTTTTTTAGTTGGGTAGT-----TATATAATTTCTGAGAATGACCTCAAGTCAAGTGAGAGTA-CCCGCTGAACTTAAGCATATCAATAA----GCGGAGGAAAAGAAACTAACAAGGATTCCCCTAGTAACGGCGAGCGAAGCGGGAAGAGCACAAATTTTAAATCTACCTGG-TTTACCGGGTCGAGTTGTAATTTGAAGAAATGTTTTTAATGTTCCGGGTTGGTTTAAATCCTTTGGGATAAGG-TATCATGGAGGGTGAGAATCCCGTGTAT-ATCAACCGCTGGGATGTTAT-AAATACATTCTCTAAGAGTCGAGTTGTTTGGGAATGCAGCTCTAAATGGGTGGTAAATTTCACCTAATGCTAAATATAAGCGAGAGACCGATAGCGAACAAGTACCGTGAGGGAAAGATGAAAAGAACTTTGAAAAGAGAGTTAAATAGTACGTGAAATTGTTGAAAGGGAAACGATTGAAGTCAGTCATGCTGGTGAGAATC----------------------------------AACTT---------------------GGAATAACTTTCAAGTGCATTTCTTCGCTTGGCAGGTTAGCGTCGATTTTGAACGTCATAAAATGATTGGGGGAAGGTAGCT-CT-------TTCGAGAGTGTTATAGCCCTTGGTAAATGTGATGTTTAGGATCGAGGTTTGCAACGGATACCTTTTA-GGCTAGCCGCCTGGCCTCTGATTCGATGTTGGGTTATAGACAGCATGCTGACTATGATCCGAT-TATTGGTCAAAAGGTTAGAGCGAGCATAAATTCGTTAAGGACGCTGACGTAATGG

>431_8_SSU_ITS_LSU_25_06_2020

CTCTTCAACGAGGAATCCCTAGTAAGCATGAGTCATCAGCTCATGCTGATTACGTCCCTGCCCTTTGTACACACCGCCCGTCGCTACTACCGATTGAATGGCTTAGTGAGACCCTCGGATCGACTCGCGGAAGCTTTAACCGGCATCCGTTTGTTGAGAAGTTGGTCAAACTTGGTCATTTAGAGGAAGTAAAAGTCGTAACAAGGTTTCCGTAGGTGAACCTGCGGAAGGATCATT-AAAA-ATATGAGGTATTT-ATACCTCTT-------------TTGTATTTAAAACCAA--CTCTTTA--------------AAACTTATTTTTTTTT------AATAAAAATAAAAACTTTCAACAACGGATCTC-TTGGCTCTCGCATCGATGAAGAACGCAGCGAAATGCGAAAAGTAATGTGAATTGCAGAATTCCGTGAATCATTAAATCTTTGAACGCAAATTGCACTCCTTGGTATTCCGAGGAGTACACTTGCTTGAGGGTCAGTTAAATAAA-------ATATCGTAACATCTTTTTTGGTGTAGCGGATCTGAGTTATCCGATTTT-AAATCGGTTACTTAAAATTAAAGATTAT--ATATAATGTGATATGTACTAAAAT-AAAGGT--------CGTTAATCATTATT-AAAACTTAATATATATATATTGCCTCTAAT----------TTTTTTTTTTTAGTTGGGTAGT-----TATATAATTTCTGAGAATGACCTCAAGTCAAGTGAGAGTA-CCCGCTGAACTTAAGCATATCAATAA----GCGGAGGAAAAGAAACTAACAAGGATTCCCCTAGTAACGGCGAGCGAAGCGGGAAGAGCACAAATTTTAAATCTACCTGG-TTTACCGGGTCGAGTTGTAATTTGAAGAAATGTTTTTAATGTTCCGGGTTGGTTTAAATCCTTTGGGATAAGG-TATCATGGAGGGTGAGAATCCCGTGTAT-ATCAACCGCTGGGATGTTAT-CAATACATTTTCTAAGAGTCGAGTTGTTTGGGAATGCAGCTCTAAATGGGTGGTAAATTTCACCTAATGCTAAATATAAGCGAGAGACCGATAGCGAACAAGTACCGTGAGGGAAAGATGAAAAGAACTTTGAAAAGAGAGTTAAATAGTACGTGAAATTGTTGAAAGGGAAACGATTGAAGTCAGTCATGCTAGCGGGAATC----------------------------------AATTT---------------------GAGGGAAACTTCAAATGCATTTCCTTGCTTGGCAGGTTAGCGTCGATTTTGAACGTCATAAAATGATTGGGGGAAGGTAGCT-CC-------TTCGGGAGTATTATAGCCCTTGATAAATGTGATGTTTGGGATCGAGGATTGCAACGGATACCTTTTA-GGCTAGCGGCCTGGCCTCTGATTCGATGTTGGGTTATAGACAGCATGCTGATTATGATCTGAT-TATTGGTCAAAAGGTTAGAGCGAGCATAAATTCGTTAAGGACGCTGACGTAATGG

>431_2_SSU_ITS_LSU_13_06_2020_cor

CTCTTCAACGAGGAATCCCTAGTAAGCATGAGTCATCAGCTCATGCTGATTACGTCCCTGCCCTTTGTACACACCGCCCGTCGCTACTACCGATTGAATGGCTTAGTGAGACCCTCGGATCGACTCGCGGAAGCTTTAACCGGCATCCGTTTGTTGAGAAGTTGGTCAAACTTGGTCATTTAGAGGAAGTAAAAGTCGTAACAAGGTTTCCGTAGGTGAACCTGCGGAAGGATCATT-AAAA-ATATGAGGTATTT-ATACCTCTT-------------TTGTATTTAAAACCAA--CTCTTTA--------------AAACTTATTTTTTTTT------AATAAAAATAAAAACTTTCAACAACGGATCTC-TTGGCTCTCGCATCGATGAAGAACGCAGCGAAATGCGAAAAGTAATGTGAATTGCAGAATTCCGTGAATCATTAAATCTTTGAACGCAAATTGCACTCCTTGGTATTCCGAGGAGTACACTTGCTTGAGGGTCAGTTAAATAAA-------ATATCGTAACATCTTTTTTGGTGTAGCGGAACTGAGTTATCCGATTTT-AAATCGGTTACTTGAAATTAAAGATTAT--ATATAATGTGATACGTACTAAAATAAAAAGT--------CGTTAATCATTATT-AAAACTTA----ATATATATTGCCTCTAAT----------TTTTTTTTTTTAGTTTAGTGGT-----TATATAATTTTTGAGAATGACCTCAAGTCAAGTGAGAGCA-CCCGCTGAACTTAAGCATATCAATAA----GCGGAGGAAAAGAAACTAACAAGGATTCCCCTAGTAACGGCGAGCGAAGCGGGAAGAGCACAAATTTTAAATCTACCTGG-TTTACCAGGTCGAGTTGTAATTTGAAGAAATGTTTTTAATGTTCCGGGTTGGTTTAAATCCTTTGGGATAAGG-TATCATGGAGGGTGAGAATCCCGTGTAT-ATCAACCGCTGGGATGTTAT-TAATACGTTTTCTAAGAGTCGAGTTGTTTGGGAATGCAGCTCTAAATGGGTGGTAAATTTCACCTAATGCTAAATATAAGCGAGAGACCGATAGCGAACAAGTACCGTGAGGGAAAGATGAAAAGAACTTTGAAAAGAGAGTTAAATAGTACGTGAAATTGTTGAAAGAGAAACGATTGAAGTCAGTCATGCTAGCGGGAATC----------------------------------AATTTG--------------------GGGAAAACTCTGAAATGCACTTCTTCGTTTGGCAGGTTAGCGTCGATTTTGAACGTTATAAAATGATTGGGGGAAGGTAGCT-CT-------TTCGAGAGTGTTATAGCCCTTGGTAAATGTGATGTTTAGGATCGAGGTTTGCAACGGATACCTTTTA-GGCTAGCCGCCTGGCCTCTGATTCGATGTCGGGTTATAGACAGCATGCTGACTATGATCTGAT-TATTGGTCAAAAGGTTAGAGCGAGCATAAATTCGTTAAGGACGCTGACGTAATGG

>437_2_SSU_ITS_LSU_7_12_2020

CTCTTCAACGAGGAATCCCTAGCCAGCATGAGTCATCAGCTCATGCTGATTACGTCCCTGCCCTTTGTACACACCGCCCGTCGCTACTACCGATTGAATGGCTTAGTGAGACCCTCGGATCGACTCGCGGAAGCTTTAACCGGCATCCGCTTGTTGAAAAGTTGGTCAAACTTGGTCATTTAGAGGAAGTAAAAGTCGTAACAAGGTTTCCGTAGGTGAACCTGCGGAAGGATCATTAAAAA-ATATGAGGTATTT-ATACCTC---------------TTGTATTTAAAACCAA--CTCTTTT--------------AAAAT--TTATTTTTT------AATAAAAATAAAAACTTTCAACAACGGATCTC-TTGGCTCTCGCATCGATGAAGAACGCAGCGAAATGCGAAAAGTAATGTGAATTGCAGAATTCCGTGAATCATTAAATCTTTGAACGCAAATTGCACTCCTTGGTATTCCGAGGAGTACACTTGCTTGAGGGTCAGTTAAATAAA-------ATATCGTAACATC-TTTTTGGTGTTGCGGATCTGAGTTATCCGGTTTT-AAGTCGGTTACTTGAAATTAAAGATTA----TAAAATGTGATACGTACTAAGAT-AAAAGT--------CGTTAATC---ATT-AAATTTTAATATA-ATATATTGCTTCTAAA----------TTTTTTTTTTTTAGTTAGTAGTATATAAATATAATTTCTGAAAATGACCTCAAGTCAAGTGAGAGTA-CCCGCTGAACTTAAGCATATCAATAA----GCGGAGGAAAAGAAACTAACAAGGATTCCCCTAGTAACGGCGAGCGAAGCGGGAAGAGCACAAATTTTAAATCTACCTGG-TTTACCAGGTCGAGTTGTAATTTGAAGAAATGTTTTTAATGTTCCGGGTTGGTTTAAATCCTTTGGGATAAGG-TATCATGGAGGGTGAGAATCCCGTGTAT-ATCAACCGCTGGGATGTTAT-TAATACATTCTCTAAGAGTCGAGTTGTTTGGGAATGCAGCTCTAAATGGGTGGTAAATTTCACCTAATGCTAAATATAAGCGAGAGACCGATAGCGAACAAGTACCGTGAGGGAAAGATGAAAAGAACTTTGAAAAGAGAGTTAAATAGTACGTGAAATTGTTGAAAGGGAAACGATTGAAGTCAGTCATGCCGGTGAGAATC----------------------------------AACTT---------------------GGAATAACTTTCAAGTGCACTTCTTCGCTTGGCAGGTTAGCGTCGATTTTGAATGTCATAAAATGATTGGGGGAAGGTAGCT-CC-------TTCGGGAGTGTTATAGCCCTTGATTAATGTGATGTTTGGGATCGAGGTTTGCAACGGATACCTTTTA-GGCTAGCCGCCTGGCCTCTGATTCGATGTCGGGTTATAGACAGCATGCTGACTATGATCAGAT-TATTGGTCAAAAGGTTAGAGCGAGTATAAATTCGTTAAGGACGCTGACGTAATGG

>437_3_SSU_ITS_LSU_7_12_2020

TTCTTCAACGAGGAATCCCTAGTAAGCATGAGTCATCAGCTCATGCTGATTACGTCCCTGCCCTTTGTACACACCGCCCGTCGCTACTACCGATTGAATGGCTTAGTGAGACCCTCGGATCGACTCGCGGAAGCTTTAACCGGCATCCGCTTGTTGAAAAGTTGGTCAAACTTGGTCATTTAGAGGAAGTAAAAGTCGTAACAAGGTTTCCGTAGGTGAACCTGCGGAAGGATCATTAAAAA-ATATGAGGTATTT-ATACCTC---------------TTGTATTTAAAACCAA--CTCTTTT--------------AAAAT--TTATTTTTT------AATAAAAATAAAAACTTTCAACAACGGATCTC-TTGGCTCTCGCATCGATGAAGAACGCAGCGAAATGCGAAAAGTAATGTGAATTGCAGAATTCCGTGAATCATTAAATCTTTGAACGCAAATTGCACTCCTTGGTATTCCGAGGAGTACACTTGCTTGAGGGTCAGTTAAATAAA-------ATATCGTAACATC-TTTTTGGTGTTGCGGATCTGAGTTATCCGGTTTT-AAGTCGGTTACTTGAAATTAAAGATTA----TAAAATGTGATACGTACTAAGAT-AAAAGT--------CGTTAATC---ATT-AAATTTTAATATA-ATATATTGCTTCTAAA----------TTTTTTTTTTTTAGTTAGTAGTATAT-AATATAATTTCTGAAAATGACCTCAAGTCAAGTGAGAGTA-CCCGCTGAACTTAAGCATATCAATAA----GCGGAGGAAAAGAAACTAACAAGGATTCCCCTAGTAACGGCGAGCGAAGCGGGAAGAGCACAAATTTTAAATCTACCTGG-TTTACCAGGTCGAGTTGTAATTTGAAGAAATGTTTTTAATGTTCCGGGTTGGTTTAAATCCTTTGGGATAAGG-TATCATGGAGGGTGAGAATCCCGTGTAT-ATCAACCGCTGGGATGTTAT-TAATACATTCTCTAAGAGTCGAGTTGTTTGGGAATGCAGCTCTAAATGGGTGGTAAATTTCACCTAATGCTAAATATAAGCGAGAGACCGATAGCGAACAAGTACCGTGAGGGAAAGATGAAAAGAACTTTGAAAAGAGAGTTAAATAGTACGTGAAATTGTTGAAAGGGAAACGATTGAAGTCAGTCATGCCGGTGAGAATC----------------------------------AACTT---------------------GGAATAACTTTCAAGTGCACTTCTTCGCTTGGCAGGTTAGCGTCGATTTTGAATGTCATAAAATGATTGGGGGAAGGTAGCT-CC-------TTCGGGAGTGTTATAGCCCTTGATTAATGTGATGTTTGGGATCGAGGTTTGCAACGGATACCTTTTA-GGCTAGCCGCCTGGCCTCTGATTCGATGTCGGGTTATAGACAGCATGCTGACTATGATCAGAT-TATTGGTCAAAAGGTTAGAGCGAGTATAAATTCGTTAAGGACGCTGACGTAATGG

>437_9_SSU_ITS_LSU_7_12_2020

CTCTTCAACGAGGAATCCCTAGTAAGCATGAGTCATCAGCTCATGCTGATTACGTCCCTGCCCTTTGTACACACCGCCCGTCGCTACTACCGATTGAATGGCTTAGTGAGACCCTCGGATCGACTCGCGGAAGCTTTAACCGGCATCCGCTTGTTGAAAAGTTGGTCAAACTTGGTCATTTAGAGGAAGTAAAAGTCGTAACAAGGTTTCCGTAGGTGAACCTGCGGAAGGATCATTAAAAA-ATATGAGGTATTT-ATACCTC---------------TTGTATTTAAAACCAA--CTCTTTT--------------AAAAT--TTATTTTTT------AATAAAAATAAAAACTTTCAACAACGGATCTC-TTGGCTCTCGCATCGATGAAGAACGCAGCGAAATGCGAAAAGTAATGTGAATTGCAGAATTCCGTGAATCATTAAATCTTTGAACGCAAATTGCACTCCTTGGTATTCCGAGGAGTACACTTGCTTGAGGGTCAGTTAAATAAA-------ATATCGTAACATC-TTTTTGGTGTTGCGGATCTGAGTTATCCGGTTTT-AAGTCGGTTACTTGAAATTAAAGATTA----TAAAATGTGATACGTACTAAGAT-AAAAGT--------CGTTAATC---ATT-AAATTTTAATATA-ATATATTGCTTCTAAA----------TTTTTTTTTTTTAGTTAGTAGTATAT-AATATAATTTCTGAAAATGACCTCAAGTCAAGTGAGAGTA-CCCGCTGAACTTAAGCATATCAATAA----GCGGAGGAAAAGAAACTAACAAGGATTCCCCTAGTAACGGCGAGCGAAGCGGGAAGAGCACAAATTTTAAATCTACCTGG-TTTACCAGGTCGAGTTGTAATTTGAAGAAATGTTTTTAATGTTCCGGGTTGGTTTAAATCCTTTGGGATAAGG-TATCATGGAGGGTGAGAATCCCGTGTAT-ATCAACCGCTGGGATGTTAT-TAATACATTCTCTAAGAGTCGAGTTGTTTGGGAATGCAGCTCTAAATGGGTGGTAAATTTCACCTAATGCTAAATATAAGCGAGAGACCGATAGCGAACAAGTACCGTGAGGGAAAGATGAAAAGAACTTTGAAAAGAGAGTTAAATAGTACGTGAAATTGTTGAAAGGGAAACGATTGAAGTCAGTCATGCCGGTGAGAATC----------------------------------AACTT---------------------GGAATAACTTTCAAGTGCACTTCTTCGCTTGGCAGGTTAGCGTCGATTTTGAATGTCATAAAATGATTGGGGGAAGGTAGCT-CC-------TTCGGGAGTGTTATAGCCCTTGATTAATGTGATGTTTGGGATCGAGGTTTGCAACGGATACCTTTTA-GGCTAGCCGCCTGGCCTCTGATTCGATGTCGGGTTATAGACAGCATGCTGACTATGATCAGAT-TATTGGTCAAAAGGTTAGAGCGAGTATAAATTCGTTAAGGACGCTGACGTAATGG

>437_8_SSU_ITS_LSU_26_11_2020

CTCTTCAACGAGGAATCCCTAGTAAGCATGAGTCATCAGCTCATGCTGATTACGTCCCTGCCCTTTGTACACACCGCCCGTCGCTACTACCGATTGAATGGCTTAGTGAGACCCTCGGATCGACTCGCGGAAGCTTTAACCGGCATCCGCTTGTTGAAAAGTTGGTCAAACTTGGTCATTTAGAGGAAGTAAAAGTCGTAACAAGGTTTCCGTAGGTGAACCTGCGGAAGGATCATTAAAAA-ATATGAGGTATTT-ATACCTC---------------TTGTATTTAAAACCAATTCTCTTTT--------------AAAAT--TTATTTTTT------AATAAAAATAAAAACTTTCAACAACGGATCTC-TTGGCTCTCGCATCGATGAAGAACGCAGCGAAATGCGAAAAGTAATGTGAATTGCAGAATTCCGTGAATCATTAAATCTTTGAACGCAAATTGCACTCCTTGGTATTCCGAGGAGTACACTTGCTTGAGGGTCAGTTAAATAAA-------ATATCGTAACATC-TTTTTGGTGTTGCGGATCTGAGTTATCCGGTTTT-AAGTCGGTTACTTGAAATTAAAGATTA----TAAAATGTGATACGTACTAAGAT-AAAAGT--------CGTTAATC---ATT-AAATTTTAATATA-ATATATTGCTTCTAAA----------TTTTTTTTTTTTAGTTAGTAGTATAT-AATATAATTTCTGAAAATGACCTCAAGTCAAGTGAGAGTA-CCCGCTGAACTTAAGCATATCAATAA----GCGGAGGAAAAGAAACTAACAAGGATTCCCCTAGTAACGGCGAGCGAAGCGGGAAGAGCACAAATTTTAAATCTACCTGG-TTTACCAGGTCGAGTTGTAATTTGAAGAAATGTTTTTAATGTTCCGGGTTGGTTTAAATCCTTTGGGATAAGG-TATCATGGAGGGTGAGAATCCCGTGTAT-ATCAACCGCTGGGATGTTAT-TAATACATTCTCTAAGAGTCGAGTTGTTTGGGAATGCAGCTCTAAATGGGTGGTAAATTTCACCTAATGCTAAATATAAGCGAGAGACCGATAGCGAACAAGTACCGTGAGGGAAAGATGAAAAGAACTTTGAAAAGAGAGTTAAATAGTACGTGAAATTGTTGAAAGGGAAACGATTGAAGTCAGTCATGCCGGTGAGAATC----------------------------------AACTT---------------------GGAATAACTTTCAAGTGCACTTCTTCGCTTGGCAGGTTAGCGTCGATTTTGAATGTCATAAAATGATTGGGGGAAGGTAGCT-CC-------TTCGGGAGTGTTATAGCCCTTGATTAATGTGATGTTTGGGATCGAGGTTTGCAACGGATACCTTTTA-GGCTAGCCGCCTGGCCTCTGATTCGATGTCGGGTTATAGACAGCATGCTGACTATGATCAGAT-TATTGGTCAAAAGGTTAGAGCGAGTATAAATTCGTTAAGGACGCTGACGTAATGG

>437_7_SSU_ITS_LSU_7_12_2020

CTCTTAAACGAGGAATCCCTAGTAAGCATGAGTCATCAGCTCATGCTGATTACGTCCCTGCCCTTTGTACACACCGCCCGTCGCTACTACCGATTGAATGGCTTAGTGAGACCCTCGGATCGACTCGCGGAAGCTTTAACCGGCATCCGCTTGTTGAAAAGTTGGTCAAACTTGGTCATTTAGAGGAAGTAAAAGTCGTAACAAGGTTTCCGTAGGTGAACCTGCGGAAGGATCATTAAAAA-ATATGAGGTATTT-ATACCTC---------------TTGTATTTAAAACCAA--CTCTTTT--------------AAAAT--TTATTTTTT------AATAAAAATAAAAACTTTCAACAACGGATCTC-TTGGCTCTCGCATCGATGAAGAACGCAGCGAAATGCGAAAAGTAATGTGAATTGCAGAATTCCGTGAATCATTAAATCTTTGAACGCAAATTGCACTCCTTGGTATTCCGAGGAGTACACTTGCTTGAGGGTCAGTTAAATAAA-------ATATCGTAACATC-TTTTTGGTGTTGCGGATCTGAGTTATCCGGTTTT-AAGTCGGTTACTTGAAATTAAAGATTA----TAAAATGTGATACGTACTAAGAT-AAAAGT--------CGTTAATC---ATT-AAATTTTAATATA-ATATATTGCTTCTAAA----------TTTTTTTTTTTTAGTTAGTAGTATAT-AATATAATTTCTGAAAATGACCTCAAGTCAAGTGAGAGTA-CCCGCTGAACTTAAGCATATCAATAA----GCGGAGGAAAAGAAACTAACAAGGATTCCCCTAGTAACGGCGAGCGAAGCGGGAAGAGCACAAATTTTAAATCTACCTGG-TTTACCAGGTCGAGTTGTAATTTGAAGAAATGTTTTTAATGTTCCGGGTTGGTTTAAATCCTTTGGGATAAGG-TATCATGGAGGGTGAGAATCCCGTGTAT-ATCAACCGCTGGGATGTTAT-TAATACATTCTCTAAGAGTCGAGTTGTTTGGGAATGCAGCTCTAAATGGGTGGTAAATTTCACCTAATGCTAAATATAAGCGAGAGACCGATAGCGAACAAGTACCGTGAGGGAAAGATGAAAAGAACTTTGAAAAGAGAGTTAAATAGTACGTGAAATTGTTGAAAGGGAAACGATTGAAGTCAGTCATGCCGGTGAGAATC----------------------------------AACTT---------------------GGAATAACTTTCAAGTGCACTTCTTCGCTTGGCAGGTTAGCGTCGATTTTGAATGTCATAAAATGATTGGGGGAAGGTAGCT-CC-------TTCGGGAGTGTTATAGCCCTTGATTAATGTGATGTTTGGGATCGAGGTTTGCAACGGATACCTTTTA-GGCTAGCCGCCTGGCCTCTGATTCGATGTCGGGTTATAGACAGCATGCTGACTATGATCAGAT-TATTGGTCAAAAGGTTAGAGCGAGTATAAATTCGTTAAGGACGCTGACGTAATGG

>437_4_SSU_ITS_LSU_7_12_2020

TTCTTCAACGAGGAATCCCTAGTCCGCATGAGTCATCAGCTCATGCTGATTACGTCCCTGCCCTTTGTACACACCGCCCGTCGCTACTACCGATTGAATGGCTTAGTGAGACCCTCGGATCGACTCGCGGAAGCTTTAACCGGCATCCGCTTGTTGAAAAGTTGGTCAAACTTGGTCATTTAGAGGAAGTAAAAGTCGTAACAAGGTTTCCGTAGGTGAACCTGCGGAAGGATCATTAAAAA-ATATGAGGTATTT-ATACCTC---------------TTGTATTTAAAACCAA--CTCTTTT--------------AAAAT--TTATTTTTT------AATAAAAATAAAAACTTTCAACAACGGATCTC-TTGGCTCTCGCATCGATGAAGAACGCAGCGAAATGCGAAAAGTAATGTGAATTGCAGAATTCCGTGAATCATTAAATCTTTGAACGCAAATTGCACTCCTTGGTATTCCGAGGAGTACACTTGCTTGAGGGTCAGTTAAATAAA-------ATATCGTAACATC-TTTTTGGTGTTGCGGATCTGAGTTATCCGGTTTT-AAGTCGGTTACTTGAAATTAAAGATTA----TAAAATGTGATACGTACTAAGAT-AAAAGT--------CGTTAATC---ATT-AAATTTTAATATA-ATATATTGCTTCTAAA----------TTTTTTTTTTTTAGTTAGTAGTATATAAATATAATTTCTGAAAATGACCTCAAGTCAAGTGAGAGTA-CCCGCTGAACTTAAGCATATCAATAA----GCGGAGGAAAAGAAACTAACAAGGATTCCCCTAGTAACGGCGAGCGAAGCGGGAAGAGCACAAATTTTAAATCTACCTGG-TTTACCAGGTCGAGTTGTAATTTGAAGAAATGTTTTTAATGTTCCGGGTTGGTTTAAATCCTTTGGGATAAGG-TATCATGGAGGGTGAGAATCCCGTGTAT-ATCAACCGCTGGGATGTTAT-TAATACATTCTCTAAGAGTCGAGTTGTTTGGGAATGCAGCTCTAAATGGGTGGTAAATTTCACCTAATGCTAAATATAAGCGAGAGACCGATAGCGAACAAGTACCGTGAGGGAAAGATGAAAAGAACTTTGAAAAGAGAGTTAAATAGTACGTGAAATTGTTGAAAGGGAAACGATTGAAGTCAGTCATGCCGGTGAGAATC----------------------------------AACTT---------------------GGAATAACTTTCAAGTGCACTTCTTCGCTTGGCAGGTTAGCGTCGATTTTGAATGTCATAAAATGATTGGGGGAAGGTAGCT-CC-------TTCGGGAGTGTTATAGCCCTTGATTAATGTGATGTTTGGGATCGAGGTTTGCAACGGATACCTTTTA-GGCTAGCCGCCTGGCCTCTGATTCGATGTCGGGTTATAGACAGCATGCTGACTATGATCAGAT-TATTGGTCAAAAGGTTAGAGCGAGTATAAATTCGTTAAGGACGCTGACGTAATGG

>Scutellospora_ovalis_KY362434

-----------------CCTAGTAAGCGTGAGTCATCAGCTCACGCTGATTACGTCCCTGCCCTTTGTACACACCGCCCGTCGCTACTGCCGATTGAATGGCTTAGTGAGACCCTCGGATCGACTCGCGGAAGCCTTAACCGGCATCCGTTTGTTGAGAAGTTGGTCAAACTTGGTCATTTAGAGGAAGTAAAAGTCGTAACAAGGTTTCCGTAGGTGAACCTGCGGAAGGATCATTAAAAAAATCCGAGGTATTT-ATACCTC---------------TTGTATCTAAAACCCAA-CTCTTAAC-------------------TTATTTCTTA------ATAAGAAGTAAAAACTTTCAACAACGGATCTC-TTGGCTCTCGCATCGATGAAGAACGCAGCGAAATGCGAAAAGTAATGTGAATTGCAGTATTCCGTGAATCATCAAATCTTTGAACGCAAATTGCACTCCT-----TTCCGAGGAGTACGCTTGCTTGAGGGTCAGTTAAAAAAAT------ATATCGTTACATC-TTTTTGGTGTTCCGGATCTGGGTTATCCGGTTTT-AAATCGGTTACCTAAAATTATTAGTTACT-TTATAATGTGATACGTACTAAAAT----AGT--------CGTTAATC---ATT-AAAACTTTA-ATATATATGTTATCTCTAA------------TTTTTTTTTTGGTTGGTAATG-----TATATAATTTCTGAAAATGACCTCAAGTCAAGTGAGAGTACCCCGCTGAACTTAAGCATATCAATAAAGGTGGGAGGAAAAGGAAACTAACAAGGATTCCCCTAGTAACGGCGAGCGAAGCGGGAAGAGCACAAATTTTAAATCTACCTGG-TTTACTGGGTCGAGTTGTAATTTGAAGAAATGTTTTTAATGTTCCGGGTTGGTTTAAATCCTTTGGGATAAGG-TATCATGGAGGGTGAGAATCCCGTGTAT-ATCAACCGCTGGGATGTTAT-AAATACATTCTCCAAGAGTCGAGTTGTTTGGGAATGCAGCTCTAAATGGGTGGTAAATTTCACCTAATGCTAAATATAAGCGAGAGACCGATAGCGAACAAGTACCGTGAGGGAAAGATGAAAAGAACTTTGAAAAGAGAGTTAAATAGTACGTGAAATTGTTGAAAGGGAAACGATTGAAGTCAGTCATGCCGGTGAGAATC----------------------------------AACTT---------------------GGGATAACTTCCAAGTGCATTTCTTCGCTTGGCAGGTTAGCGTCGATTTTGAACGTCATAAAATGATTGGGGGAAGGTAGCT-CT-------TTCGGGAGTGTTATAGCCCTTAGTTAATGTGATGTTCGGGATCGAGGTTTGCAACGGATACCTTTTA-GGCTAGCCGCCTGGCCTCTGATTCGATGTCGGGTTATAGACAGCATGCTGACTATGGTCTGAT-TATTGGTCAAAAGGTTAGAGCGAGCATAAATTCGTTAAGGACGCTGACGTAATGG

>Scutellospora_ovalis_KY362435

CTCTTCAACGAGGAATCCCTAGTAAGCGTGAGTCATCAGCTCACGCTGATTACGTCCCTGCCCTTTGTACACACCGCCCGTCGCTACTGCCGATTGAATGGCTTAGTGAGACCCTCGGATCGACTCGCGGAAGCCTTAACCGGCATCCGTTTGTTGAGAAGTTGGTCAAACTTGGTCATTTAGAGGAAGTAAAAGTCGTAACAAGGTTTCCGTAGGTGAACCTGCGGAAGGATCATTAAAAAAATCCGAGGTATTT-ATACCTC---------------TTGTATCTAAAACCCAA-CTCTTAAC-------------------TTATTTCTTA------ATAAGAAGTAAAAACTTTCAACAACGGATCTC-TTGGCTCTCGCATCGATGAAGAACGCAGCGAAATGCGAAAAGTAATGTGAATTGCAGTATTCCGTGAATCATCAAATCTTTGAACGCAAATTGCACTCCT-----TTCCGAGGAGTACGCTTGCTTGAGGGTCAGTTAAAAAAAT------ATATCGTTACATC-TTTTTGGTGTTCCGGATCTGGGTTATCCGGTTTT-AAATCGGTTACCTAAAATTATTAGTTACT-TTATAATGTGATACGTACTAAAAT----AGT--------CGTTAATC---ATT-AAAACTTTA-ATATATATGTTATCTCTAA------------TTTTTTTTTTGGTTGGTAATG-----TATATAATTTCTGAAAATGACCTCAAGTCAAGTGAGAGTACCCCGCTGAACTTAAGCATATCAATAAAGGTGGGAGGAAAAGGAAACTAACAAGGATTCCCCTAGTAACGGCGAGCGAAGCGGGAAGAGCACAAATTTTAAATCTACCTGG-TTTACTGGGTCGAGTTGTAATTTGAAGAAATGTTTTTAATGTTCCGGGTTGGTTTAAATCCTTTGGGATAAGG-TATCATGGAGGGTGAGAATCCCGTGTAT-ATCAACCGCTGGGATGTTAT-AAATACATTCTCCAAGAGTCGAGTTGTTTGGGAATGCAGCTCTAAATGGGTGGTAAATTTCACCTAATGCTAAATATAAGCGAGAGACCGATAGCGAACAAGTACCGTGAGGGAAAGATGAAAAGAACTTTGAAAAGAGAGTTAAATAGTACGTGAAATTGTTGAAAGGGAAACGATTGAAGTCAGTCATGCCGGTGAGAATC----------------------------------AACTT---------------------GGGATAACTTCCAAGTGCATTTCTTCGCTTGGCAGGTTAGCGTCGATTTTGAACGTCATAAAATGATTGGGGGAAGGTAGCT-CT-------TTCGGGAGTGTTATAGCCCTTAGTTAATGTGATGTTCGGGATCGAGGTTTGCAACGGATACCTTTTA-GGCTAGCCGCCTGGCCTCTGATTCGATGTCGGGTTATAGACAGCATGCTGACTATGGTCTGAT-TATTGGTCAAAAGGTTAGAGCGAGCATAAATTCGTTAAGGACGCTGACGTAATGG

>Scutellospora_alterata_HF93501

CTCTTCAACGAGGAATCCCTAGTAAGCGTGAGTCATCAGCTCATGCTGATTACGTCCCTGCCCTTTGTACACACCGCCCGTCGCTACTACCGATTGAATGGCTTAGTGAGACCCTCGGATCGACGAATGGAAGCCTTAACCGGCATCTGTTTGATGAGAAGTTGGTCAAACTTGGTCATTTAGAGGAAGTAAAAGTCGTAACAAGGTTTCCGTAGGTGAACCTGCGGAAGGATCATTAAAAATTTTTGAGGTATTTTATACCTC---------------TTGTATTTAAAACCCAA-CTCTTTC--------------AAACCTAAATTTTTTAATAAAAATAAAAAAAAAAAACTTTCAACAACGGATCTC-TTGGCTCTCGCATCGATGAAGAACGCAGCGAAATGCGAAAAGTAATGTGAATTGCAAAATTCCGTGAATCATTAAATCTTTGAACGCAAATTGCATTCCTTGGTATTCCGAGGAGTACACTTGCTTGAGGGTCAGTTAAAAATAA------GCATCGTTACATC--TTTTGGTGTTGCGGATCTGGGTTATTCAATTTT-ATGTCGATTACCTAAAATTAAGAATTA----TATAATGTGATACGTACTA--AT-AAAAGT--------CGTTAATC---ATT-AAAGTTTAT------TACATTATCAACTA------------ATATATATTTAG--GAGTTAG-----TGTATAATTTCTGAGAATGACCTCAAGTCAAGTGAGAGTA-CCCGCTGAACTTAAGCATATCAATAA----GCGGAGGAAAAGAAACTAACAAGGATTCCCCTAGTAACGGCGAGTGAAGCGGGAAGAGCACAAATTTTAAATCTACTTGGTTTTACCAGGTCGAGTTGTAATCTGAAGAAACGTTTTTAATGTTCCAGGTTGGTTTAAATCCTTTGGGATAAGG-TATCATGGAGGGTGAGAATCCCGTGTAT-ATCAACCTCTGGGATGTTAT-CAATACGTTTTCTAAGAGTCGAGTTGTTTGGGAATGCAGCTCTAAATGGGTGGTAAATTTCACCTAATGCTAAATATAAGCGAGAGACCGATAGCGAACAAGTACCGTGAGGGAAAGATGAAAAGAACTTTGAAAAGAGAGTTAAATAGTACGTGAAATTGTTGAAAGGGAAACGATTGAAGTCAGTCATGCTGGCAGGAATC----------------------------------AATTTTATGGAAAGGGGATTTTTTTGAACCTAACCTTGAAATGCATTTCTTTGCTTGGCAGGTTAGCGTCGATTTTGAACGTCATAAAATGATTGGGAGAAGGTAGCT-CT-------TTCGGGAGTGTTATAGCTCTTAATTAATGTGATGTTTGGGATCGAGGGTTGCAACGGATACCTTTTA-GGCTAGCCGCCTGGCCTCTAATTCGATGTTGGGTTATAGACAGCATGCTGACTATGATCTAAT-TATTGATTAAAAGGTTAGAGCGAGCATAAATTCGTTAAGGACGCTGACGTAATGG

>Scutellospora_alterata_HF935022

CTCTTCAACGAGGAATCCCTAGTAAGCGTGAGTCATCAGCTCATGCTGATTACGTCCCTGCCCTTTGTACACACCGCCCGTCGCTACTACCGATTGAATGGCTTAGTGAGACCCTCGGATCGACGAATGGAAGCCTTAACCGGCATCTGTTTGATGAGAAGTTGGTCAAACTTGGTCATTTAGAGGAAGTAAAAGTCGTAACAAGGTTTCCGTAGGTGAACCTGCGGAAGGATCATTAAAAATTTTTGAGGTATTTTATACCTC---------------TTGTATTTAAAACCCAA-CTCTTTC--------------AAACCTAAATTTTTTAATAAAAATAAAAAAAAAAAACTTTCAACAACGGATCTC-TTGGCTCTCGCATCGATGAAGAACGCAGCGAAATGCGAAAAGTAATGTGAATTGCAAAATTCCGTGAATCATTAAATCTTTGAACGCAAATTGCACTCCTTGGTATTCCGAGGAGTACACTTGCTTGAGGGTCAGTTAAAAATAA------ACATCGTTACATC--TTTTGGTGTTGCGGATCTGGGTTATTCAATTTT-ATGTCGATTACCTAAAATTAAGAATTA----TATAATGTGATACGTACTA--AT-AAAAGT--------CGTTAATC---ATT-AAAGTTTAT------TACATTATCAACTA------------ATATATATTTAG--GAGTTAG-----TGTATAATTTCTGAGAATGACCTCAAGTCAAGTGAGAGTA-CCCGCTGAACTTAAGCATATCAATAA----GCGGAGGAAAAGAAACTAACAAGGATTCCCCTAGTAACGGCGAGTGAAGCGGGAAGAGCACAAATTTTAAATCTACTTGGTTTTACCAGGTCGAGTTGTAATTTGAAGAAACGTTTTTAATGTTCCAGGTTGGTTTAAATCCTTTGGGATAAGG-TATCATGGAGGGTGAGAATCCCGTGTAT-ATCAACCTCTGGGATGTTAT-CAATACGTTTTCTAAGAGTCGAGTTGTTTGGGAATGCAGCTCTAAGTGGGTGGTAAATTTCACCTAATGCTAAATATAAGCGAGAGACCGATAGCGAACAAGTACCGTGAGGGAAAGATGAAAAGAACTTTGAAAAGAGAGTTAAATAGTACGTGAAATTGTTGAAAGGGAAACGATTGAAGTCAGTCATGCTGGCAGGAATC----------------------------------AATTTTATGGAAAGGGGATTTTTTTGAACCTAACCTTGAAATGCATTTCTTTGCTTGGCAGGTTGGCGTCGATTTTGAACGTCATAAAATGATTGGGAGAAGGTAGCT-CT-------TTCGGGAGTGTTATAGCTCTTAATTAATGTGATGTTTGGGATCGAGGGTTGCAACGGATACCTTTTA-GGCTAGCCGCCTGGCCTCTAATTCGATGTTGGGTTATAGACAGCATGCTGACTATGATCTAAT-TATTGATTAAAAGGTTAGAGCGAGCATAAATTCGTTAAGGACGCTGACGTAATGG

>Scutellospora_alterata_HF935020

CTCTTCAACGAGGAATCCCTAGTAAGCGTGAGTCATCAGCTCATGCTGATTACGTCCCTGCCCTTTGTACACACCGCCCGTCGCTACTACCGATTGAATGGCTTAGTGAGGCCCTCGGATCGACGAATGGAAGCCTTAACCGGCATCTGTTTGATGAGAAGTTGGTCAAACTTGGTCATTTAGAGGAAGTAAAAGTCGTAACAAGGTTTCCGTAGGTGAACCTGCGGAAGGATCATTAAAAATTTTTGAGGTATTTTATACCTC---------------TTGTATTTAAAACCCAA-CTCTTTC--------------AAACCTAAATTTCTTAATAAAAAT-AAAAAAAAGAACTTTCAACAACGGATCTC-TTGGCTCTCGCATCGATGAAGAACGCAGCGAAATGCGAAAAGTAATGTGAATTGCAGAATTCCGTGAATCATTAAATCTTTGAACGCAAATTGCACTCCTTGGTATTCCGAGGAGTACACTTGCTTGAGGGTCAGTTAAAAATAA------ACATCGTTACATC--TTTTGGTGTTGCGGATCTGGGTTATTCAATTTT-ATGTCGATTACCTAAAATTAAGAATTA----TATAATGTGATACGTACTA--AT-AAAAGT--------CGTTAATC---ATT-AAAGTTTAT------TACATTATCAACTA------------ATATATATTTAG--GAGTTAG-----TGTATAATTTCTGAGAATGACCTCAAGTCAAGTGAGAGTA-CCCGCTGAACTTAAGCATATCAATAA----GCGGAGGAAAAGAAACTAACAAGGATTCCCCTAGTAACGGCGAGTGAAGCGGGAAGAGCACAAATTTTAAATCTACTTGGTTTTACCAGGTCGAGTTGTAATTTGAAGAAACGTTTTTAATGTTCCAGGTTGGTTTAAATCCTTTGGGATAAGG-TATCATGGAGAGTGAGAATCCCGTGTAT-ATCAACCTCTGGGATGTTAT-CAATACGTTTTCTAAGAGTCGAGTTGTTTGGGAATGCAGCTCTAAATGGGTGGTAAATTTCACCTAATGCTAAATATAAGCGAGAGACCGATAGCGAACAAGTACCGTGAGGGAAAGATGAAAAGAACTTTGAAAAGAGAGTTAAATAGTACGTGAAATTGTTGAAAGGGAAACGATTGAAGTCAGTCATGCTGGCAGGAATC----------------------------------AATTTTATGGAAAGGGGATTTTTTTGAACCTAACCTTGAAATGCATTTCTTTGCTTGGCAGGTTAGCGTCGATTTTGAACGTCATAAAATGATTGGGAGAAGGTAGCT-CT-------TTCGGGAGTGTTATAGCTCTTAATTAATGTGATGTTTGGGATCGAGGGTTGCAACGGATACCTTTTA-GGCTAGCCGCCTGGCCTCTAATTCGATGTTGGGTTATAGACAGCATGCTGACTATGATCTAAT-TATTGATTAAAAGGTTAGAGCGAGCATAAATTCGTTAAGGACGCTGACGTAATGG

>Scutellospora_alterata_HF935021

CTCTTCAACGAGGAATCCCTAGTAAGCGTGAGTCATCAGCTCATGCTGATTACGTCCCTGCCCTTTGTACACACCGCCCGTCGCTACTACCGATTGAATGGCTTAGTGAGACCCTCGGATCGACGAATGGAAGCCTTAACCGGCATCTGTTTGATGAGAAGTTGGTCAAACTTGGTCATTTAGAGGAAGTAAAAGTCGTAACAAGGTTTCCGTAGGTGAACCTGCGGAAGGATCATTAAAAATTTTTGAGGTATTTTATACCTC---------------TTGTATTTAAAACCCAA-CTCTTTC--------------AAACCTAAATTTTTTAATAAAAAT-AAAAAAAAAAACTTTCAACAACGGATCTC-TTGGCTCTCGCATCGATGAAGAACGCAGCGAAATGCGAAAAGTAATGTGAATTGCAGAATTCCGTGAATCATTAAATCTTTGAGCGCAAATTGCACTCCTTGGTATTCCGAGGAGTACACTTGCTTGAGGGTCAGTTAAAAATAA------ACATCGTTACATC--TTTTGGTGTTGCGGATCTGGGTTATTCAATTTT-ATGTCGATTACCTAAAATTAAGAATTA----TATAATGTGATACGTACTA--AT-AAAAGT--------CGTTAATC---ATT-AAAGTTTAT------TACATTATCAACTA------------ATATATATTTAG--GAGTTAG-----TGTATAATTTCTGAGAATGACCTCAAGTCAAGTGAGAGTA-CCCGCAGAACTTAAGCATATCAATAA----GCGGAGGAAAAGAAACTAACAAGGATTCCCCTAGTAACGGCGAGTGAAGCGGGAAGAGCACAAATTTTAAATCTACTTGGTTTTACCAGGTCGAGTTGTAATTTGAAGAAACGTTTTTAATGTTCCAGGTTGGTTTAAATCCTTTGGGATAAGG-TATCATGGAGGGTGAGAATCCCGTGTAT-ATCAACCTCCGGGATGTTAT-CAATACGTTTTCTAAGAGTCGAGTTGTTTGGGAATGCAGCTCTAAATGGGTGGTAAATTTCACCTAATGCTAAATATAAGCGAGAGACCGATAGCGAACAAGTACCGTGAGGGAAAGATGAAAAGAACTTTGAAAAGAGAGTTAAATAGTACGTGAAATTGTTGAAAGGGAAACGATTGAAGTCAGTCATGCTGGCAGGAATC----------------------------------AATTTTATGGAAAGGGGATTTTTTTGAACCTAACCTTGAAATGCATTTCTTTGCTTGGCAGGTTAGCGTCGATTTTGAACGTCATAAAATGATTGGGAGAAGGTAGCT-CC-------TTCGGGAGTGTTATAGCTCTTAATTAATGTGATGTTTGGGATCGAGGGTTGCAACGGATACCTTTTA-GGCTAGCCGCCTGGCCTCTAATTCGATGTTGGGTTATAGACAGCATGCTGACTATGATCTAAT-TATTGATTAAAAGGTTAGAGCGAGCATAAATTCGTTAAGGACGCTGACGTAATGG

>Scutellospora_calospora_EU346867

-----------------------------------------------------------------------------------------------------------------------------------------------------------------------------------------------------------------------------------------------------------------------------------------------------------------------------------------------------------------------------------------------------------------------------------------------------------------------------------------------------------------------------------------------------------------------------------------------------------------------------------------------------------------------------------------------------------------------------------------------------------------------------------------------------------GCATATCAATAA----GCGGAGGAAAAGAAACTAACAAGGATTCCCCTAGTAACGGCGAGTGAAGCGGGAAGAGCACAAATTTTAAATCTACCTGGGTTTACTAGGTCGAGTTGTAATTTGAAGAAACGTTTTTAATGTTCCGGGTTGGTTTAAATCCTTTGGGATAAGG-TATCATGGAGGGTGAGAATCCCGTGTAT-ATCAACCGCTGGGATGTTAT-TAATACGTTCTCTAAGAGTCGAGTTGTTTGGGAATGCAGCTCTAAATGGGTGGTAAATTTCACCTAATGCTAAATATAAGCGAGAGACCGATAGCGAACAAGTACCGTGAGGGAAAGATGAAAAGTACTTTGAAAAGAGAGTTAAATAGTACGTGAAATTGTTGAAAGGGAAACGATTGAAGTCAGTCATGCCGGCGGGAATC----------------------------------AATTTTGAGGAAGGGGGA-TTTTTTGAACCTAACCTTGAAATGCACTTCCTCGCTTGGTAGGTTAGCGTCGATTTTGAACGTCATAAAATGATTGGGGGAAGGTAGCT-CT-------TTCGGGAGTGTTATAGCCCTTAATTAATGTGATGTTTGGGATCGAGGATTGCAACGGATACCTTTTA-GGCTAGCCGCCTGGCCTCTGATTCGATGTCGGGTTATAGACAGCACGCTGACTATGATTTGAT-TATTGGTCAAAAGGTTAGAGCGAGCATAAATTCGTTAAGGACGCTGACGTAATGG

>Scutellospora_calospora_EU252109

-----------------------------------------------------------------------------------------------------------------------------------------------------------------------------------------------------------------------------------------------------------------------------------------------------------------------------------------------------------------------------------------------------------------------------------------------------------------------------------------------------------------------------------------------------------------------------------------------------------------------------------------------------------------------------------------------------------------------------------------------------------------------------------------------------------GCATATCAATAA----GCGGAGGAAGAGAAACTAACAAGGATTCCCCTAGTAACGGCGAGTGAAGCGGGAAGAGCACAAATTTTAAATCTACCTGGTTTTACTAGGTCGAGTTGTAATTTGAAGAAACGTTTTTAATTTTCCGGGTTGGTTTAAATCCTTTGGGATAAGG-TATCGTGGAGGGTGAGAATCCCGTGTAT-ATCAACCGCTGGGATGTTAT-TAATACGTTCTCTAAGAGTCGAGTTGTTTGGAAATGCAGCTCTAAATGGGTGGTAAATTTCACCTAATGCTAAATATAAGCGAGAGACCGATAGCGAACAAGTACCGTGAGGGAAAGATGAAAAGTACTTTGAAAAGAGAGTTAAATAGTACGTGAAATTGTTGAAAGGGAAACGATTGAAGTCAGTCATGCCGGCGGGAATC----------------------------------AATTTTGAGGAAGGGGGA-TTTTTTGAACCTAACCTTGAAATGCACTTCTTCGCTTGGCAGGTTAGCGTCGATTTTGAACGTCATAAAATGATTGGGGGAAGGTAGCT-CT-------TTCGGGAGTGTTATAGCCCTTAATTAATGTGATGTTTGGGATCGAGGATTGCAACGGATACCTTTTA-GGCTAGCCGCCTGGCCTCTGATTCGATGTCGGGTTATAGACAGCACGCTGACTATGATTTGAT-TATTGGTCAAAAGGTTAGAGCGAGCATAAATTCGTTAAGGACGCTGACGTAATGG

>Scutellospora_dipurpurescens_FJ461868

------------------------------------------------------------------------------------------------------------------------------------------------------------------------------------------------------------------------------------------------------------------------------------------------------------------------------------------------------------------------------------------------------------------------------------------------------------------------------------------------------------------------------------------------------------------------------------------------------------------------------------------------------------------------------------------------------------------------------------------------------------------------------------------------------------------------------------------CTAACAAGGATTCCCCTAGTAACGGCGAGTGAAGCGGGAAGAGCACAAATTTTAAATCTACCTGGTTTTACTAGGTCGAATTGTAATTTAAAGAAACGTTTTTAATGTTCCGGGTTGGTTTAAATCCTTTGGGATAAGG-TATCATGGAGGGTGAGAATCCCGTGTAT-ATCAACCGCTGGGATGTTAT-TAATACGTTCTCTAAGAGTCGAGTTGTTTGGGAATGCAGCTCTAAATGGGTGGTAAATTTCACCTAATGCTAAATATAAGCGAGAGACCGATAGCGAACAAGTACCGTGAGGGAAAGATGAAAAGAACTTTGAAAAGAGAGTTAAATAGTACGTGAAATTGTTGAAAGGGAAACGATTGAAGTCAGTCATGCCGGCGGGAATC----------------------------------AATTTTGAGGAAGGGGGATTTTTTTGAACCTAACTTTGAAATGCACTTCTTCGCTTGGCAGGTTAGCGTCGATTTTGAACGTCATAAAATGATTGGGGGAAGGTAGCT-CT-------TTCGGGAGTGTTATAGCCCTTAATTAATGTGATGTTTGGGATCGAGGATTGCAACGGATACCTTTTA-GGCTAGCCACCTAGCCTCTGATTCGATGTCGAGTTATAGACAGCATGCTGACTATGATTTGAT-TATTGGTCAAAAGGTTAGAGTGAGCATAAATTCGTTAAGGACGC-----------

>Scutellospora_alterata_HF935024

CTCTTCAACGAGGAATCCCTAGTAAGCGTGAGTCATCAGCTCATGCTGATTACGTCCCTGCCCTTTGTACACACCGCCCGTCGCTACTACCGATTGAATGGCTTAGTGAGACCCTCGGATCGACAAATGGAAGCCTTAACCGGCATCTGTTTGATGAGAAGTTGGTCAAACTTGGTCATTTAGAGGAAGTAAAAGTCGTAACAAGGTTTCCGTAGGTGAACCTGCGGAAGGATCATTAAAAA-ATTTGAGGTATTTTATA--TC---------------TTGTATTTAAAACCCAA-CTCTTTC--------------AAACCTAAATTTTTTA---TAATAAAAATAAAAAAACTTTCAACAACGGATCTC-TTGGCTCTCGCATCGATGAAGAACGCAGCGAAATGCGAAAAGTAATGTGAATTGCAGAATTCCGTGAATCATTAAATCTTTGAACGCAAATTGCACTCCTTGGTATTCCGAGGAGTACACTTGCTTGAGGGTCAGTTAAAAAATA------ATATCGTTACATC-TTTTTGGTGTTGCGGATCTGGGCTATCCGGTTTTTAAGTCGGTTACCTAAAATTAAATATTAT---TATAATGTGATACGTACTAAGAT-AAAGGT--------CGTTAATC---ATTAAAAATTTAA----TATATATTATCTCTAA-------------TTCTTTTTTAGTTGGTAGTG-----TATATAATTTCTGAGAATGACCTCAAGTCAAGTGAGAGTA-CCCGCTGAACTTAAGCATATCAATAA----GCGGAGGAAAAGAAACTAACAAGGATTCCCCTAGTAACGGCGAGTGAAGCGGGAAGAGCACAAATTTTAAATCTACTTGGTTTTACCAGGTCGAGTTGTAATTTGAAGAAACGTTTTTAATGTTCCAGGTTGGTTTAAATCCTTTGGGATAAGG-TATCATGGAGGGTGAGAATCCCGTGTAT-ATCAACCTCTGGAATGTTAT-CAATACGTTTTCTAAGAGTCGAGTTGTTTGGGAATGCAGCTCTAAATGGGTGGTAAATTTCACCTAATGCTAAATATAAGCGAGAGACCGATAGCGAACAAGTACCGTGAGGGAAAGATGAAAAGAACTTTGAAAAGAGAGTTAAATAGTACGTGAAATTGTTGAAAGGGAAACGATTGAAGTCAGTCATGCCGGCAGGAATC----------------------------------AATTTTATGGAAAGGGGATTTTTTTGAACCTAACCTTGAAATGCATTTCTTTGCTTGGCAGGTTAGCGTCGATTTTGAACGTCATAAAATGATTGGGAGAAGGTAGCT-CC-------TTC-GGAGTGTTATAGCTCTTAATTAATGTGATGTTTGGGATCGAGGGTTGCAACGGATACCTTTTA-GGCTAGCCGCCTGGCCTCTAATTCGATGTTGGGTTATAGACAGCATGCTGACTATGATCTAAT-TATTGATTAAAAGGTTAGAGCGAGCATAAATTCGTTAAGGACGCTGACGTAATGG

>Scutellospora_deformata_MZ234124_MZ234127

------------------------------------------------------------------------------------------------------------------------------------------ACCGGCATCCGTTTGTTGAGAAGTTGGTCAAACTTGGTCATTTAGAGGAAGTAAAAGTCGTAACAAGGTTTCCGTAGGTGAACCTGCGGAAGGATCATTAGAAA-AATTGAGGTATATTATACCTC---------------TTGTATTTAAAATCCAA-CTCTATAT-------------AAACCTAAATTTTTTA------ATAAAAAATAAAAACTTTCAACAACGGATCTC-TTGGCTCTCGCATCGATGAAGAACGCAGCGAAATGCGATAAGTAATGTGAATTGCAGAATTCCGTGAATCATTAAATCTTTGAACGCAAATTGCACTCCTTGGTATTCCGAGGAGTACACTTGCTTGAGGGTCAGTTAAATAAA-------ATATCGTTACATC-TTTTTGGTGTTGCGGATCTGGGTTTTCCGGTTTTAAAGTCGGTTACCTAAAATTAAGGATTA----TATAATGTGATACGTACTAAAAT-AAAAGT--------CGTTAATC---ATT-AATCTTTAT------TACATTACCAACTA------------TTATGTTTTTAGTAGGAGGAG-----TGTATAATTTTT--------------------------------------------------------------------------------------------------------------GGAAGAGCACAAATTTTAAATCTATCTGGTTCTACCAGGTCGAGTTGTAATTTGAAGAAACGTTTTTAATGTTCCGGGTTGGTTTAAATCCTTTGGGATAAGG-TATCATGGAGGGTGAGAATCCCGTGTAT-ATCAACCGCTGGGATGTTAT-TAATACGTTCTCTAAGAGTCGAGTTGTTTGGGAATGCAGCTCTAAATGGGTGGTAAATTTCACCTAATGCTAAATATAAGCGAGAGACCGATAGCGAACAAGTACCGTGAGGGAAAGATGAAAAGAACTTTGAAAAGAGAGTTAAATAGTACGTGAAATTGTTGAAAGGGAAACGATTGAAGTCAGTCATGCCGGTGAGAATC----------------------------------AACTTTGGGGAAGGGGGATTTTTTTGAACCTAACCTTGAAGTGCACTTCTTCGCTTGGCAGGTTAGCGTCGATTTTAAACGTCATAAAATGATTGGGGGAAGGTAGCT-CT-------TTCGGGAGTGTTATAGCCCTTAATTAATGTG------------------------------------------------------------------------------------------------------------------------------------------------------------

>Scutellospora_spinosissima_FR750149_SSU_ITS_LSU

------------------------------------------------ATTACGTCCCTGCCCTTTGTACACACCGCCCGTCGCTACTACCGATTGAATGGCTTAGTGAGACCTTCGGATCGACGAATAGAAGCTTTAACCGGCATCCGTTTGTTGAAAAGTTGGTCAAACTTGGTCATTTAGAGGAAGTAAAAGTCGTAACAAGGTTTCCGTAGGTGAACCTGCGGAAGGATCATTAAAAA-AAATGAGGTATTT-ATACCTC---------------TTGTATTTAAAATCAAA-CCTTTTTT-------------AAACT--TTATTTTTA------ATATAAAATAAAAACTTTCAACAACGGATCTC-TTGGCTCTCGCATCGATGAAGAACGCAGCGAAATGCGAAAAGTAATGTGAATTGCAGAATTCCGTGAATCATTAAATCTTTGAACGCAAATTGCACTTCTTGGTATTCCGAGGAGTACACTTGCTTGAGGGTCAGTTAAATAAA-------GTATCGTAATATC--TTTTGGTGTTGCGGATCTGGGTTGTCCGGTTTT-AAATCGGTTACCTAAAATTATTAATTATTATTATAATGTAATACGTACTAAAA--AAAAGT--------CGTTAATC---ATTAAAATTTTAATATATATATATTAC------------------CTTTTCGGTTAGTATAATATA-----TATGTTATTTTTGAGAATGACCTCAAGTCAAGTGAGAGTA-CCCGCTGAACTTAAGCATATCAATAA----GCGGAGGAAAAGAAACTAACAAGGATTCCCCTAGTAACGGCGAGTGAAGCGGGAAAAGCACAAAATTTAAATCTACCTGG--TTACCAGGTCGAGTTGTAATTTGAAGAAAGTGTTTTAATGTTCCGGGTTGGTTTAAATCCTTTGGGATAAGG-TATCATGGAGGGTGAGAATCCCGTGTAT-ATCAACCGCTGGGATGTTAT-TAATACATTTTCGAAGAGTCGAGTTGTTTGGGAATGCAGCTCTAAATGGGTGGTAAATTTCACCTAATGCTAAATATAAGCGAGAGACCGATAGCGAACAAGTACCGTGAGGGAAAGATGAAAAGTACTTTGAAAAGAGAGTTAAATAGTACGTGAAATTGTTGAAAGGGAAACGATTGAAGTCAGTCATGCCGGCAAGAATC----------------------------------AATTT---------------------GGAATAACATCCGAATGTATTTCTTCGCTTGGCAGGTTAGCGTCGATTTTGAACGTCATAAAATGATTGGGGGAAGGTAGCT-TC-------TTCGGAAGTGTTATAGCCTTTAGTTAATGTGATGTTTGGGATCGAGGATTGCAACGGATACCTTTTA-GGCTAGCCGCCTGACCTCTGATTCGATGTTGGGTTATAGACAGCATGCTGACTATGATTTGAT-TATCGGTCAAAAGGTTAGAGTGAGCATAAATTCGTTAAGGACGCTGACGTAATGG

>Orbispora_pernambucana_JF965445

-----------------------------------------------------------------------------------------------------------------------------------------------------------------------------------------------------------------------------------------------------------------------------------------------------------------------------------------------------------------------------------------------------------------------------------------------------------------------------------------------------------------------------------------------------------------------------------------------------------------------------------------------------------------------------------------------------------------------------------------------------------------------------------------------------------GCATATCAATAA----GCGGAGGAAAAGAAACTAACAAGGATTCCCCTAGTAACGGCGAGTGAAGCGGGAGGAGCACAAATTTTAAATCTATCCGGTTTTGCTGGGTCGAGTTGTAATTTAAAGAAACGTTTTTGACGTTCCTGGTTGGTTTAAATCCTTTGGGATAAGGTTATCATAGAGGGTGAGAATCCCGTATAT-ACTAACCGCTGGGATGTTAT-TAATACGTTTTCGAAGAGTCGAGCTGTTTGGGAATGCAGCTCTAAATGGGTGGTAAATTTCACCTAATGCTAAATATAAGCGAGAGACCGATAGTGAACAAGTACCGTGAGGGAAAGATGAAAAGAACTTTGAAAAGAGAGTTAAATAGTACGTGAAATTGTTGAAAGGGAAACGGTTGAAGTCAGTCATGCTGGCAGGAATC----------------------------------AATCTTA-------------------AAGTTAAATTTGAGGTGCACTTCCTTGCTTGGCAGGTTAGCATCGATTTTAAACGTCATAAAATGATTGGGGGAAGGTAGCTCCT-------TTTAGGAGTGTTATAGCCCTTAGTTAATGTGATGTTCGGGATCGAGGATTGCAACGGATACCCATTAGGGCTAGCCGCCTAGCCTCTGATATGATGTTGGGTCATGGACAGCACGCTGACTGTGATCTGAT-TATCTTTCAAAAGGTTAGAGTGAACATAAATTCGTTAAGGATG-TGACGTAAT--

>Orbispora_pernambucana_JF965446

-----------------------------------------------------------------------------------------------------------------------------------------------------------------------------------------------------------------------------------------------------------------------------------------------------------------------------------------------------------------------------------------------------------------------------------------------------------------------------------------------------------------------------------------------------------------------------------------------------------------------------------------------------------------------------------------------------------------------------------------------------------------------------------------------------------GCATATCAATAA----GCGGAGGAAAAGAAACTAACAAGGATTCCCCTAGTAACGGCGAGTGAAGCGGGAGGAGCACAAATTTTAAATCTACCCGGTTTTGCTGGGTCGAGTTGTAATTTAAAGAAACGTTTTTGACGTTCCTGGTTGGTTTAAATCCTTTGGGATAAGGTTATCATAGAGGGTGAGAATCCCGTATAT-ACTAACCGCTGGGATGTTAT-TAATACGTTTTCGAAGAGTCGAGTTGTTTGGGAATGCAGCTCTAAATGGGTGGTAAATTTCACCTAATGCTAAATATAAGCGAGAGACCGATAGTGAACAAGTACCGTGAGGGAAAGATGAAAAGAACTTTGAAAAGAGAGTTAAATAGTACGTGAAATTGTTGAAAGGGAAACGGTTGAAGTCAGTCATGCTGGCAGGAATC----------------------------------AATCTTA-------------------AAGTTAAATTTGAGGTGCACTTCCTTGCTTGGCAGGTTAGCATCGATTTTAAACGTCATAAAATGATTGGGGGAAGGTAGCTCCT-------TTTAGGAGTGTTATAGCCCTTAGTTAATGTGATGTTCGGGATCGAGGATTGCAACGGATACCCATTAGGGCTAGCCGCCTAGCCTCTGATATGATGTTGGGTCATGGACAGCACGCTGACTGTGATCTGAT-TATCTTTCAAAAGGTTAGAGTGAACATAAATTCGTTAAGGATG-TGACGTAATGG

>Orbispora_pernambucana_JQ340917

-----------------------------------------------------------------------------------------------------------------------------------------------------------------------------------------------------------------------------------------------------------------------------------------------------------------------------------------------------------------------------------------------------------------------------------------------------------------------------------------------------------------------------------------------------------------------------------------------------------------------------------------------------------------------------------------------------------------------------------------------------------------------------------------------------------GCATATCAATAA----GCGGAGGAAAAGAAACTAACAAGGATTCCCCTAGTAACGGCGAGTGAAGCGGGAGGAGCACAAATTTTAAATCTACCCGGTTTTGCTGGGTCGAGTTGTAATTTAAAGAAACGTTTTTGACGTTCCTGGTTGGTTTAAATCCTTTGGGATAAGGTTATCATAGAGGGTGAGAATCCCGTATAT-ACTAACCGCTGGGATGTTAT-TAATACGTTTTCGAAGAGTCGAGTTGTTTGGGAATGCAGCTCTAAATGGGTGGTAAATTTCACCTAATGCTAAATATAAGCGAGAGACCGATAGTGAACAAGTACCGTGAGGGAAAGATGAAAAGAACTTTGAAAAGAGAGTTAAATAGTACGTGAAATTGTTGAAAGGGAAACGGTTGAAGTCAGTCATGCTGGCAGGAATC----------------------------------AATCTTA-------------------AAGTTAAATTCGAGGTGCACTTCCTTGCTTGGCAGGTTAGCATCGATTTTAAACGTCATAAAATGATTGGGGGAAGGTAGCTCCT-------TTTAGGAGTGTTATAGCCCTTAGTTAATGTGATGTTCGGGATCGAGGATTGCAACGGATACCCATTAGGGCTAGCCGCCTAGCCTCTGATATGATGTTGGGTCATGGACAGCACGCTGACTGTGATCTGAT-TATCTTTCAAAAGGTTAGAGTGAACATAAATTCGTTAAGGATG-TGACGTAATGG

>Orbispora_pernambucana_JQ340918

-----------------------------------------------------------------------------------------------------------------------------------------------------------------------------------------------------------------------------------------------------------------------------------------------------------------------------------------------------------------------------------------------------------------------------------------------------------------------------------------------------------------------------------------------------------------------------------------------------------------------------------------------------------------------------------------------------------------------------------------------------------------------------------------------------------GCATATCAATAA----GCGGAGGAAAAGAAACTAACAAGGATTCCCCTAGTAACGGCGAGTGAAGCGGGAGGAGCACAAATTTTAAATCTACCCGGTTTTGCTGGGTCGAGTTGTAATTTAAAGAAACGTTTTCGACGTTCCTGGTTGGTTTAAATCCTTTGGGATAAGGTTATCATAGAGGGTGAGAATCCCGTATAT-ACTAACCGCTGGGATGTTAT-TAATACGTTTTCGAAGAGTCGAGTTGTTTGGGAATGCAGCTCTAAATGGGTGGTAAATTTCACCTAATGCTAAATATAAGCGAGAGACCGATAGTGAACAAGTACCGTGAGGGAAAGATGAAAAGAACTTTGAAAAGAGAGTTAAATAGTACGTGAAATTGTTGAAAGGGAAACGGTTGAAGTCAGTCATGCTGGCAGGAATC----------------------------------AATCTTA-------------------AAGTTAAATTTGAGGTGCACTTCCTTGCTTGGCAGGTTAGCACCGATTTTAAACGTCATAAAATGATTGGGGGAAGGTAGCTCCT-------TTTAGGAGTGTTATAGCCCTTAGTTAATGTGATGTTCGGGATCGAGGATTGCAACGGATACCCATTAGGGCTAGCCGCCTAGCCTCTGATATGATGTTGGGTCATGGACAGCACGCTGACTGTGATCTGAT-TATCTTTCAAAAGGTTAGAGTGAACATAAATTCGTTAAGGATG-TGACGTAATGG
