# Supplementary material for: Three new species of arbuscular mycorrhizal fungi (Glomeromycota) and Acaulospora gedanensis revised
Source: Front Microbiol. 2024 Feb 12;15:1320014. doi: 10.3389/fmicb.2024.1320014 (PMC10896085; doi:10.3389/fmicb.2024.1320014)
Supplement: Supplementary Table 4 — An alignment used to produce Figure 7. [file Table_4.DOCX]

>Acaulospora_brasiliensis_FN825902

CCTAGTAAGCGTGAGTCATCAGCTCACGTTGATTACGTCCCT-GCCCTTTGTACACACCGCCCGTCGCTACTACCGATTGAAT-GGCTTAGTGAGACTCTCGGATTAAGGCTAAGGAATCGGAAACGGTTTCTTTTTCTCGAGAAGTTTGTCAAACTTGGTCATTTAGAGGAAGTAAAAGTCGTAACAAGGTTTCCGTAGGTGAACCTGCGGAAGGATCATTAGAATA-TTTA------------------------------------------TAC------TTTATTC-AAAAA-TTTTCAATCTAT----AAAT-TTTATTTTTTTTTTTTTTA-------------------AGATATTAAAAGATAACTTTCAACAACGGATCTCTTGGCTCTTGCATCGATGAAGAACGCAGCGAAATGCGATAAGTAATATGAATTGCAGAATTCCGTGAATCATTAAATCTTTGAACGCAAATTGCACTTCTT-GGTATTCCGAGGAGTATGCTTGCTTGAGGGTTGGTCTAC-AAAAAAATCGT-GAAAT-------------TAATTTTTT-------------CGCGGATCTGGG---TTTTCCAAAG---AC---AT-----------------------TTTTTTTGGTAACCTT-AAATTTATCTTAACAAT-TTTAGGT-----ATTAA-AA-TTAG-AAATGGTT--TTAT-CTTACATA------------------TTGTAAGT-TGTT-CA-TTGATAATTAT-----ACC---AATTGTTACATCGCG--TTATTCACCCAATC-----------------------------TT-TTTT--GA----TT-AGG--T-GAAATAA-TTCAA-TACA-----TTTTT--AATCCTACCTCAAGTC-AAGTAAG-AATACCCGCTGAAC-TT-AAGCATATCAATAAGCGGAGGAAAAGAAACTAAC-TAGGATTCCC-TTAG-TAAC-GGCGAGTGAACTGGGAAAAGCTCAAA-TTTTAAATCAC------------CTCGGTGAATTGTAATTTGAAGAAAGT-G-TTTTGAC-TTTTCAGTTTAATC-TAAATCCTTTGGGA--TGAGGTATCAT-AG-AGGGTGAGAATCCCGTTCTTGATTAAACCTCG---GAT--TGCACTAAATTCACTTTCCAAGAGTCGAGTTG-TTTGGGATTGCAGCTCCAAATGGGTGGTAAATTTCACCTAAGGCTAAATATATGCAAGAGACCGATAGCGAACAAGTACCGT-GAGGGAAAGA--TGAAAAGTACTTTGAAAAGAGAGTTAAACAGTACGTGAAATTGTTGAAAGGGAAACGATTGAAGTCAGTCATGCTAGTGAGGAATCAACTTGGTGG----------------------------------------------------------TTTACTTCCTTGTGCATTTTCTC--GCTTGGCAGGTTAGCATCAA-TTTTGGTTGTCATCAAAGAATT-GGGGGAATGTGACTTT---TCTTCGGTAAAGTG-TTATAGACCCTGAT-TGATGTGGCGACTGGGATTGAGGTTTGCAGCGGATGCC-TCTTG----TGGCTAGTCACCTGT-CTTCTGACC-GTCACCTC--GTTTTTGACAGC-TTGCTGATGATTGCGGGATTTGGCGATCTAG-GA-GTTAGAGTGAACATAAA-TTCGCTAAGGATGCTGACGTAATGGCTTTAAACGACCCGTCTTGAAACACGGACCAAGGAGTCTAAC-ATATGTGCGAGTGTT

>Acaulospora_brasiliensis_FN825904

CCTAGTAAGCGTGAGTCATCAGCTCACGTTGATTACGTCCCT-GCCCTTTGTACACACCGCCCGTCGCTACTACCGATTGAAT-GGCTTAGTGAGACTCTCGGATTAAGGCTAAGGAATCGGAAACGGTTTCTTTTTCTCGAGAAGTTTGTCAAACTTGGTCATTTAGAGGAAGTAAAAGTCGTAACAAGGTTTCCGTAGGTGAACCTGCGGAAGGATCATTAGAATA-TTTA------------------------------------------TAC------TTTATTC-AAAAA-TTTTCAATCTAT----AAAT-TTTA-----TTTTTTTTTA-------------------AGATATT-AAAGATAACTTTCAACAACGGATCTCTTGGCTCTTGCATCGATGAAGAACGCAGCGAAATGCGATAAGTAATATGAATTGCAGAATTCCGTGAATCATTAAATCTTTGAACGCAAATTGCACTTCTT-GGTATTCCGAGGAGTATGCTTGCTTGAGGGTTGGTCTAC-AAAAAAATCGT-GAAAT-------------TAATTTTTT-------------CGCGGATCTGGG---TTTTCCAAAG---AC---AT-----------------------TTTTTTTGGTAACCTT-AAATTTATCTTAACAAT-TTTAGGT-----ATTAA-AA-TTAG-AAATGGTT--TTAT-CTTACATA------------------TTGTAAGT-TGTT-CA-TTGATAATTAT-----ACC---AATTGTTACATCGCG--TTATTCACCCAATC-----------------------------TT-TTTT--GA----TT-AGG--T-GAAATAA-TTCAA-TACA-----TTTTT--AATCCTACCTCAAGTC-AAGTAAG-AATACCCGCTGAAC-TT-AAGCATATCAATAAGCGGAGGAAAAGAAACTAAC-TAGGATTCCC-TTAG-TAAC-GGCGAGTGAACTGGGAAAAGCTCAAA-TTTTAAATCAC------------CTCGGTGAATTGTAATTTGAAGAAAGT-G-TTTTGAC-TTTTCAGTTTAATC-TAAATCCTTTGGGA--TGAGGTATCAT-AG-AGGGTGAGAATCCCGTTCTTGATTAAACCTCG---GAT--TGCACTAAATTCACTTTCCAAGAGTCGAGTTG-TTTGGGATTGCAGCTCCAAATGGGTGGTAAATTTCACCTAAGGCTAAATATATGCAAGAGACCGATAGCGAACAAGTACCGT-GAGGGAAAGA--TGAAAAGTACTTTGAAAAGAGAGTTAAACAGTACGTGAAATTGTTGAAAGGGAAACGATTGAAGTCAGTCATGCTAGTGAGGAATCAACTTGGTGG----------------------------------------------------------TTTACTTCCTTGTGCATTTTCTC--GCTTGGCAGGTTAGCATCAA-TTTTGGTTGTCATCAAAGAATT-GGGGGAATGTGACTTT---TCTTCGGTAAAGTG-TTATAGACCCTGAT-TGATGTGGCGACTGGGATTGAGGTTTGCAGCGGATGCC-TCTTG----TGGCTAGTCACCTGT-CTTCTGACC-GTCACCTC--GTTTTTAACAGC-TTGCTGATGATTGCGGGATTTGGCGATCTAG-GA-GTTAGAGTGAACATAAA-TTCGCTAAGGATGCTGACGTAATGGCTTTAAACGACCCGTCTTGAAACACGGACCAAGGAGTCTAAC-ATATGTGCGAGTGTT

>Acaulospora_brasiliensis_FN825905

CCTAGTAAGCGTGAGTCATCAGCTCACGTTGATTACGTCCCT-GCCCTTTGTACACACCGCCCGTCGCTACTACCGATTGAAT-GGCTTAGTGAGACTCTCGGATTAAGGCTAAGGAATCGGAAACGGTTTCTTTTTCTCGAGAAGTTTGTCAAACTTGGTCATTTAGAGGAAGTAAAAGTCGTAACAAGGTTTCCGTAGGTGAACCTGCGGAAGGATCATTAGAATA-TTCAGGAAT------------------TTATTTTCCT----AATATTAC------TTTATTC-AAAAA-TTTTCAATCTAT----AAAT-TTTATTTTTTTTTTTTTTA-------------------AGATATTAAAAGATAACTTTCAACAACGGATCTCTTGGCTCTTGCATCGATGAAGAACGCAGCGAAATGCGATAAGTAATATGAATTGCAGAATTCCGTGAATCATTAAATCTTTGAACGCAAATTGCACTTCTT-GGTATTCCGAGGAGTATGCTTGCTTGAGGGTTGGTCTAC-AAAAAAATCGT-GAAAT-------------TAATTTTTT-------------CGCGGATCTGGG---TTTTCCAAAG---AC---AT-----------------------TTTTTTTGGTAACCTT-AAATTTATCTTAACAAT-TTTAGGT-----ATTAA-AA-TTAG-AAATGGTT--TTAT-CTTACATA------------------TTGTAAGT-TGTT-CA-TTGATAATTAT-----ACC---AATTGTTACATCGCG--TTATTCACCCAATC-----------------------------TT-TTTT--GA----TT-AGG--T-GAAATAA-TTCAA-TACA-----TTTTT--AATCCTACCTCAAGTC-AAGTAAG-AATACCCGCTGAAC-TT-AAGCATATCAATAAGCGGAGGAAAAGAAACTAAC-TAGGATTCCC-TTAG-TAAC-GGCGAGTGAACTGGGAAAAGCTCAAA-TTTTAAATCAC------------CTCGGTGAATTGTAATTTGAAGAAAGT-G-TTTTGAC-TTTTCAGTTTAATC-TAAATCCTTTGGGA--TGAGGTATCAT-AG-AGGGTGAGAATCCCGTTCTTGATTAAACCTCG---GAT--TGCACTAAATTCACTTTCCAAGAGTCGAGTTG-TTTGGGATTGCAGCTCCAAATGGGTGGTAAATTTCACCTAAGGCTAAATATATGCAAGAGACCGATAGCGAACAAGTACCGT-GAGGGAAAGA--TGAAAAGTACTTTGAAAAGAGAGTTAAACAGTACGTGAAATTGTTGAAAGGGAAACGATTGAAGTCAGTCATGCTAGTGAGGAATCAACTTGGTGG----------------------------------------------------------TTTACTTCCTTGTGCATTTTCTC--GCTTGGCAGGTTAGCATCAA-TTTTGGTTGTCATCAAAGAATT-GGGGGAATGTGACTTT---TCTTCGGTAAAGTG-TTATAGACCCTGAT-TGATGTGGCGACTGGGATTGAGGTTTGCAGCGGATGCC-TCTTG----TGGCTAGTCACCTGT-CTTCTGACC-GTCACCTC--GTTTTTAACAGC-TTGCTGATGATTGCGGGATTTGGCGATCTAG-GA-GTTAGAGTGAACATAAA-TTCGCTAAGGATGCTGACGTAATGGCTTTAAACGACCCGTCTTGAAACACGGACCAAGGAGTCTAAC-ATATGTGCGAGTGTT

>Acaulospora_brasiliensis_FN825909

CCTAGTAAGCGTGAGTCATCAGCTCACGTTGATTACGTCCCT-GCCCTTTGTACACACCGCCCGTCGCTACTACCGATTGAAT-GGCTTAGTGAGACTCTCGGATTAAGGCTAAGGAATCGGAAACGGTTTCTTTTTCTCGAGAAGTTTGTCAAACTTGGTCATTTAGAGGAAGTAAAAGTCGTAACAAGGTTTCCGTAGGTGAACCTGCGGAAGGATCATTAGAATA-TTCAGGAAT------------------TTATTTTCCT----AATATTAC------TTTATTC-AAAAA-TTTTCAATCTAT----AAAT-TTTATTTTTTTTTTTTTTA-------------------AGATATTAAAAGATAACTTTCAACAACGGATCTCTTGGCTCTTGCATCGATGAAGAACGCAGCGAAATGCGATAAGTAATATGAATTGCAGAATTCCGTGAATCATTAAATCTTTGAACGCAAATTGCACTTCTT-GGTATTCCGAGGAGTATGCTTGCTTGAGGGTTGGTCTAC-AAAAAAATCGT-GAAAT-------------TAATTTTTT-------------CGCGGATCTGGG---TTTTCCAAAG---AC---AT-----------------------TTTTTTTGGTAACCTT-AAATTTATCTTAACAAT-TTTAGGT-----ATTAA-AA-TTAG-AAATGGTT--TTAT-CTTACATA------------------TTGTAAGT-TGTT-CA-TTGATAATTAT-----ACC---AATTGTTACATCGCG--TTATTCACCCAATC-----------------------------TT-TTTT--GA----TT-AGG--T-GAAATAA-TTCAA-TACA-----TTTTT--AATCCTACCTCAAGTC-AAGTAAG-AATACCCGCTGAAC-TT-AAGCATATCAATAAGCGGAGGAAAAGAAACTAAC-TAGGATTCCC-TTAG-TAAC-GGCGAGTGAACTGGGAAAAGCTCAAA-TTTTAAATCAC------------CTCGGTGAATTGTAATTTGAAGAAAGT-G-TTTTGAC-TTTTCAGTTTAATC-TAAATCCTTTGGGA--TGAGGTATCAT-AG-AGGGTGAGAATCCCGTTCTTGATTAAACCTCG---GAT--TGCACTAAATTCACTTTCCAAGAGTCGAGTTG-TTTGGGATTGCAGCTCCAAATGGGTGGTAAATTTCACCTAAGGCTAAATATATGCAAGAGACCGATAGCGAACAAGTACCGT-GAGGGAAAGA--TGAAAAGTACTTTGAAAAGAGAGTTAAACAGTACGTGAAATTGTTGAAAGGGAAACGATTGAAGTCAGTCATGCTAGTGAGGAATCAACTTGGTGG----------------------------------------------------------TTTACTTCCTTGTGCATTTTCTC--GCTTGGCAGGTTAGCATCAA-TTTTGGTTGTCATCAAAGAATT-GGGGGAATGTGACTTT---TCTTCGGTAAAGTG-TTATAGACCCTGAT-TGATGTGGCGACTGGGATTGAGGTTTGCAGCGGATGCC-TCTTG----TGGCTAGTCACCTGT-CTTCTGACC-GTCACCTC--GTTTTTAACAGC-TTGCTGATGATTGCGGGATTTGGCGATCTAG-GA-GTTAGAGTGAACATAAA-TTCGCTAAGGATGCTGACGTAATGGCTTTAAACGACCCGTCTTGAAACACGGACCAAGGAGTCTAAC-ATATGTGCGAGTGTT

>Acaulospora_brasiliensis_FN825906

CCTAGTAAGCGTGAGTCATCAGCTCACGTTGATTACGTCCCT-GCCCTTTGTACACACCGCCCGTCGCTACTACCGATTGAAT-GGCTTAGTGAGACTCTCGGATTAGGGCTAAGGAATCGGAAACGGTTTCTTTTTCTCGAGAAGTTTGTCAAACTTGGTCATTTAGAGGAAGTAAAAGTCGTAACAAGGTTTCCGTAGGTGAACCTGCGGAAGGATCATTAGAATA-TTTA------------------------------------------TAC------TTTATTCAAAAAA-TTTTCAATCTAT----AAAT-TT--ATATATTTTTTTTTA-------------------AGATATT-AAAGATAACTTTCAACAACGGATCTCTTGGCTCTTGCATCGATGAAGAACGCAGCGAAATGCGATAAGTAATGTGAATTGCAGAATTCCGTGAATCATTAAATCTTTGAACGCAAATTGCACTTCTT-GGTATTCCGAGGAGTATGCTTGCTTGAGGGTTGGTCTAC-AAAAAAATCGT-GAAAT-------------TAATTTTTT-------------CGCGGATCTGGG---TTTTCCAAAG---AC---AT-----------------------TTTTTTTGGTAACCTT-AAATTTATCTTAACAAT-TTTAGGT-----ATTAA-AA-TTAG-AAATGGTT--TTAT-CTTACATA------------------TTGTAAGT-TGTT-CA-TTGATAATTAT-----ACC---AATTGTTACATCGCG--TTATTCACCCAATC-----------------------------TT-TTTT--GA----TT-AGG--T-GAAATAA-TTCAA-TACA-----TTTTT--AATCCTACCTCAAGTC-AAGTAAG-AATACCCGCTGAAC-TT-AAGCATATCAATAAGCGGAGGAAAAGAAACTAAC-TAGGATTCCC-TTAG-TAAC-GGCGAGTGAACTGGGAAAAGCTCAAA-TTTTAAATCAC------------CTCGGTGAATTGTAATTTGAAGAAAGT-G-TTTTGAC-TTTTCAGTTTAATC-TAAATCCTTTGGGA--TGAGGTATCAT-AG-AGGGTGAGAATCCCGTTCTTGATTAAACCTCG---GAT--TGCACTAAATTCACTTTCTAAGAGTCGAGTTG-TTTGGGATTGCAGCTCCAAATGGGTGGTAAATTTCACCTAAGGCTAAATATATGCAAGAGACCGATAGCGAACAAGTACCGT-GAGGGAAAGA--TGAAAAGTACTTTGAAAAGAGAGTTAAACAGTACGTGAAATTGTTGAAAGGGAAACGATTGAAGTCAGTCATGCTAGTGAGGAATCAACTTGGTGG----------------------------------------------------------TTTACTTCCTTGTGCATTTTCTC--GCTTGGCAGGTTAGCATCAA-TTTTGGTTGTCATCAAAGAATT-GGGGGAATGTGACTTT---TCTTCGGTAAAGTG-TTATAGACCCTGAT-TGATGTGGCGACTGGGATTGAGGTTTGCAGCGGATGCC-TCTTG----TGGCTAGTCACCTGT-CTTCTGACC-GTCACCTC--GTTTTTAACAGC-TTGCTGATGATTGCGGGATTTGGCGATCTAG-GA-GTTAGAGTGAACATAAA-TTCGCTAAGGATGCTGACGTAATGGCTTTAAACGACCCGTCTTGAAACACGGACCAAGGAGTCTAAC-ATATGTGCGAGTG--

>Acaulospora_brasiliensis_FN825907

CCTAGTAAGCGTGAGTCATCAGCTCACGTTGATTACGTCCCT-GCCCTTTGTACACACCGCCCGTCGCTACTACCGATTGAAT-GGCTTAGTGAGACTCTCGGATTAAGGCTAAGGAATCGGAAACGGTTTCTTTTTCTCGAGAAGTTTGTCAAACTTGGTCATTTAGAGGAAGTAAAAGTCGTAACAAGGTTTCCGTAGGTGAACCTGCGGAAGGATCATTAGAATA-TTTA------------------------------------------TAC------TTTATTC-AAAAA-TTTTCAATCTAT----AAAT-TTTATTTTTTTTTTTTTTA-------------------AGATATTAAAAGATAACTTTCAACAACGGATCTCTTGGCTCTTGCATCGATGAAGAACGCAGCGAAATGCGATAAGTAATATGAATTGCAGAATTCCGTGAATCATTAAATCTTTGAACGCAAATTGCACTTCTT-GGTATTCCGAGGAGTATGCTTGCTTGAGGGTTGGTCTAC-AAAAAAATCGT-GAAAT-------------TAATTTTTT-------------CGCGGATCTGGG---TTTTCCAAAG---AC---AT-----------------------TTTTTTTGGTAACCTT-AAATTTATCTTAACAAT-TTTAGGT-----ATTAA-AA-TTAG-AAATGGTT--TTAT-CTTACATA------------------TTGTAAGT-TGTT-CA-TTGATAATTAT-----ACC---AATTGTTACATCGCG--TTATTCACCCAATC-----------------------------TT-TTTT--GA----TT-AGG--T-GAAATAATTTCAA-TACA-----TTTTT--AATCCTACCTCAAGTC-AAGTAAG-AATACCCGCTGAAC-TT-AAGCATATCAATAAGCGGAGGAAAAGAAACTAAC-TAGGATTCCC-TTAG-TAACGGGCGAGTGAACTGGGAAAAGCTCAAATTTTTAATTCAC------------CTCGGTGAATTGTAATTTGAAGAAAGT-G-TTTTGAC-TTTTCAGTTTAATC-TAAATCCTTTGGGA--TGAGGTATCAT-AG-AGGGTGAGAATCCCGTTCTTGATTAAACCTCG---GAT--TGCACTAAATTCACTTTCCAAGAGTCGAGTTG-TTTGGGATTGCAGCTCCAAATGGGTGGTAAATTTCACCTAAGGCTAAATATATGCAAGAGACCGATAGCGAACAAGTACCGT-GAGGGAAAGA--TGAAAAGTACTTTGAAAAGAGAGTTAAACAGTACGTGAAATTGTTGAAAGGGAAACGATTGAAGTCAGTCATGCTAGTGAGGAATCAACTTGGTGG----------------------------------------------------------TTTACTTCCTTGTGCATTTTCTC--GCTTGGCAGGTTAGCATCAA-TTTTGGTTGTCATCAAAGAATT-GGGGGAATGTGACTTT---TCTTCGGTAAAGTG-TTATAGACCCTGAT-TGATGTGGCGACTGGGATTGAGGTTTGCAGCGGATGCC-TCTTG----TGGCTAGTCACCTGT-CTTCTGACC-GTCACCTC--ACTTTTGACAGC-TTGCTGATGATTGCGGGATTTGGCGACCTAG-AA-GTTAGAGTGAACATAAA-TTCGCTAAGGATGCTGACGTAATGGCTTTAAACGACCCGTCTTGAAACACGGACCAAGGAGTCTAAC-ATATGTGCGAGTGTT

>Acaulospora_brasiliensis_FN825903

CCTAGTAAGCGTGAGTCATCAGCTCACGTTGATTACGTCCCT-GCCCTTTGTACACACCGCCCGTCGCTACTACCGATTGAAT-GGCTTAGTGAGACTCTCGGATTAGGGCTAAGGAATCGGAAACGGTTTCTTTTTCTCGAGAAGTTTGTCAAACTTGGTCATTTAGAGGAAGTAAAAGTCGTAACAAGGTTTCCGTAGGTGAACCTGCGGAAGGATCATTAGAATA-TTCAGGAAT------------------TTATTTTCCT----AATAATAC------TTTATTCAAAAAA-TTTTCAATCTAT----AAAT-TT--ATATATTTTTTTTTA-------------------AGATATT-AAAGATAACTTTCAACAACGGATCTCTTGGCTCTTGCATCGATGAAGAACGCAGCGAAATGCGATAAGTAATGTGAATTGCAGAATTCCGTGAATCATTAAATCTTTGAACGCAAATTGCACTTCTT-GGTATTCCGAGGAGTATGCTTGCTTGAGGGTTGGTCTAC-AAAAAAATCGT-GAAAT-------------TTA-TTTTT-------------CACGGATCTGGG---TTTTCCAAAG---AC---AT-----------------------TTTTTTTGGTAACCTT-AAATTTATCTTAACAAT-TTTAGGT-----ATTAA-AA-TTAG-AAATGGTT--TTAT-CTTACATA------------------TTGTAAGT-TGTT-CA-TTGATAATTAT-----ACC---AATTGTTACATCGCG--TTATTCACCCAATC-----------------------------TT-TTTT--GA----TT-AGG--T-GAAATAA-TTCAA-TACA-----TTTTTTAATCCCTACCTCAAGTCAAAGTAAG-AATACCCGCTGAACTTT-AAGCATATCAATAAGCGGAGGAAAAGAAACTAAC-TAGGATTCCC-TTAG-TAAC-GGCGAGTGAACTGGGAAAAGCTCAAA-TTTTAAATCAC------------CTCGGTGAATTGTAATTTGAAGAAAGT-G-TTTTGAC-TTTTCAGTTTAATC-TAAATCCTTTGGGA--TGAGGTATCAT-AG-AGGGTGAGAATCCCGTTCTTGATTAAACCTCG---GAT--TGCACTAAATTCACTTTCCAAGAGTCGAGTTG-TTTGGGATTGCAGCTCCAAATGGGTGGTAAATTTCACCTAAGGCTAAATATATGCAAGAGACCGATAGCGAACAAGTACCGT-GAGGGAAAGA--TGAAAAGTACTTTGAAAAGAGAGTTAAACAGTACGTGAAATTGTTGAAAGGGAAACGATTGAAGTCAGTCATGCTAGTGAGGAATCAACTTGGTGG----------------------------------------------------------TTTACTTCCTTGTGCATTTTCTC--GCTTGGCAGGTTAGCATCAA-TTTTGGTTGTCATCAAAGAATT-GGGGGAATGTGACTTT---TCTTCGGTAAAGTG-TTATAGACCCTGAT-TGATGTGGCGACTGGGATTGAGGTTTGCAGCGGATGCC-TCTTG----TGGCTAGTCACCTGT-CTTCTGACC-GTCACCTC--GTTTTTAACAGC-TTGCTGATGATTGCGGGATTTGGCGATCTAG-GA-GTTAGAGTGAACATAAA-TTCGCTAAGGATGCTGACGTAATGGCTTTAAACGACCCGTCTTGAAACACGGACCAAGGAGTCTAAC-ATATGTGCGAGTGTT

>Acaulospora_brasiliensis_FN825908

CCTAGTAAGCGTGAGTCATCAGCTCACGTTGATTACGTCCCT-GCCCTTTGTACACACCGCCCGTCGCTACTACCGATTGAAT-GGCTTAGTGAGACTCTCGGATTAGGGCTAAGGAATCGGAAACGGTTTCTTTTTCTCGAGAAGTTTGTCAAACTTGGTCATTTAGAGGAAGTAAAAGTCGTAACAAGGTTTCCGTAGGTGAACCTGCGGAAGGATCATTAGAATA-TTTAGGAAT------------------TTATTTTCCT----AATAATAC------TTTATTC-AAAAA-TTTTCAATCTAT----AAAT-TT-----TTTTTTTTTTTA-------------------AGATATTAAAAGATAACTTTCAACAACGGATCTCTTGGCTCTTGCATCGATGAAGAACGCAGCGAAATGCGATAAGTAATGTGAATTGCAGAATTCCGTGAATCATTAAATCTTTGAACGCAAATTGCACTTCTT-GGTATTCCGAGGAGTATGCTTGCTTGAGGGTTGGTCTAC-AAAAAAATCGG-GAAAT-------------TAATTTTTT-------------CGCGGATCTGGG---TTTTCCAAAG---AC---AT-----------------------TTTTTTTGGTAACCTT-AAATTTATCTTAACAAT-TTTAGGT-----ATTAA-AA-TTAG-AAATGGTT--TTAT-CTTACATA------------------TTGTAAGT-TATT-CA-TTGATAATTAT-----ACC---AATTGTTACATCGCG--TTATTCACTCAATC-----------------------------TT-TTTT--GA----TT-AGG--T-GAAATAA-TTCAA-TACA-----TTTTT--AATCCTACCTCAAGTC-AAGTAAG-AATACCCGCTGAAC-TT-AAGCATATCAATAAGCGGAGGAAAAGAAACTAAC-TAGGATTCCC-TTAG-TAAC-GGCGAGTGAACTGGGAAAAGCTCAAA-TTTTAAATCAC------------CTCGGTGAATTGTAATTTGAAGAAAGT-G-TTTTGAC-TTTTCAGTTTAATC-TAAATCCTTTGGGA--TGAGGTATCAT-AG-AGGGTGAGAATCCCGTTCTTGATTAAACCTCG---GAT--TGCACTAAATTCACTTTCCAAGAGTCGAGTTG-TTTGGGATTGCAGCTCCAAATGGGTGGTAAATTTCACCTAAGGCTAAATATATGCAAGAGACCGATAGCGAACAAGTACCGT-GAGGGAAAGA--TGAAAAGTACTTTGAAAAGAGAGTTAAACAGTACGTGAAATTGTTGAAAGGGAAACGATTGAAGTCAGTCATGCTAGTGAGGAATCAACTTGGTGG----------------------------------------------------------TTTACTTCCTTGTGCATTTTCTC--GCTTGGCAGGTTAGCATCAA-TTTTGGTTGTCATCAAAGAATTGGGGGGAATGTGACTTT---TCTTTGGTAAAGTG-TTATAGACCCTGAT-TGATGTGGCGACTGGGATTGAGGTTTGCAGCGGATGCC-TCTTG----TGGCTAGTCACCTGT-CTTCTGACC-GTCACCTC--GTTTTTGACAGC-TTGCTGATGATTGCGGGATTTGGCGATCTAG-GA-GTTAGAGTGAACATAAA-TTCGCTAAGGATGCTGACGTAATGGCTTTAAACGACCCGTCTTGAAACACGGACCAAGGAGTCTAAC-ATATGTGCGAGTGTT

>Acaulospora_brasiliensis_FN825910

-CTAGTAAGCGTGAGTCATCAGCTCACGTTGATTACGTCCCT-GCCCTTTGTACACACCGCCCGTCGCTACTACCGATTGAAT-GGCTTAGTGAGACTCTCGGATTAGGGCTAAGGAATCGGAAACGGTTTCTTTTTCTCGAGAAGTTTGTCAAACTTGGTCATTTAGAGGAAGTAAAAGTCGTAACAAGGTTTCCGTAGGTGAACCTGCGGAAGGATCATTAGAATA-TTCAGGAAT------------------TTATTTTCCT----AATAATAC------TTTATTC-AAAAA-TTTTCAATCTAC----AAAT-TT--ATATATTTTTTTTAA-------------------AGATATTAAAAGATAACTTTCAACAACGGATCTCTTGGCTCTTGCATCGATGAAGAACGCAGCGAAATGCGATAAGTAATATGAATTGCAGAATTCCGTGAATCATTAAATCTTTGAACGCAAATTGCACTTCTT-GGTATTCCGAGGAGTATGCTTGCTTGAGGGTTGGTCTAC-AAAAAAATCGT-GAAAT-------------TAATTTTTT-------------CGCGGACCTGGG---TTTTCCAAAG---ACTTTTT-----------------------TTTTTTTGGTAACCTT-AAATTTATCTTAACAAT-TTTAGGT-----ATTAA-AA-TTAG-AAATGGTT--TTAT-CTTACATA------------------TTGTAAGT-TGTT-CA-TTGATAATTAT-----ACC---AATTGTTACATCGCG--TTATTCACCCAATC-----------------------------TT-TTTT--GA----TT-AGG--T-GAAATAA-TTCAA-TACA-----TTTTT--AATCCTACCTCAAGTC-AAGTAAG-AATACCCGCTGAAC-TT-AAGCATATCAATAAGCGGAGGAAAAGAAACTAAC-TAGGATTCCC-TTAG-TAAC-GGCGAGTGAACTGGGAAAAGCTCAAA-TTTTAAATCAC------------CTCGGTGAATTGTAATTTGAAGAAAGT-G-GTTTGAC-TTTTCAGTTTAATC-TAAATCCTTTGGGGATGAAGGTATCAT-AG-AGGGTGAGAATCCCGTTCTTGATTAAACCTCG---GAT--TGCACTAAATTCACTTTCTAAGAGTCGAGTTG-TTTGGGATTGCAGCTCCAAATGGGTGGTAAATTTCACCTAAGGCTAAATATATGCAAGAGACCGATAGCGAACAAGTACCGT-GAGGGAAAGA--TGAAAAGTACTTTGAAAAGAGAGTTAAACAGTACGTGAAATTGTTGAAAGGGAAACGATTGAAGTCAGTCATGCTAGTGAGGAATCAACTTGGTGG----------------------------------------------------------TTTACTTCCTTGTGCATTTTCTC--GCTTGGCAGGTTAGCATCAA-TTTTGGTTGTCATCAAAGAATT-GGGGGAATGTGACTTT---TCTTCGGTAAAGTG-TTATAGACCCTGAT-TGATGTGGCGACTGGGATTGAGGTTTGTAGCGGATGCC-TCTTG----TGGCTAGTCACCTAT-CTTCTGACC-GTCACCTC--ACTTTTGACAGC-TTGCTGATGATTGCGGGATTTGGCGACCTAG-AA-GTTAGAGTGAACATAAA-TTCGCTAAGGATGCTGACGTAATGGCTTTAAACGACCCGTCTTGAAACACGGACCAAGGAGTCTAAC-ATATGTGCGAGTGTT

>Acaulospora_brasiliensis_FN825911

CCTAGTAAGCGTGAGTCATCAGCTCACGTTGATTACGTCCCT-ACCCTTTGTACACACCGCCCGTCGCTACTATCGATAGAAT-GGCTTAGTGAGACTCTCGGATTAGGGCTAAGGAATCGGAAACGGTTTCTTTTTCTCGAGAAGTTTGTCAAACTTGGTCATTTAGAGGAAGTAAAAGTCGTAACAAGGTTTCCGTAGGTGAACCTGCGGAAGGATCATTAGAATA-TTCAGGAAT------------------TTATTTTCCT----AATAATAC------TTTATTC-AAAAA-TTTTCAATCTAC----AAAT-TT--ATATATTTTTTTTAA-------------------AGATATTAAAAGATAACTTTCAACAACGGATCTCTTGGCTCTTGCATCGATGAAGAACGCAGCGAAATGCGATAAGTAATATGAATTGCAGAATTCCGTGAATCATTAAATCTTTGAACGCAAATTGCACTTCTT-GGTATTCCGAGGAGTATGCTTGCTTGAGGGTTGGTCTAC-AAAAAAATCGT-GAAAT-------------TAATTTTTT-------------CGCGGACCTGGG---TTTTCCAAAG---AC--TTT-----------------------TTTTTTTGGTAACCTT-AAATTTATCTTAACAAT-TTTAGGT-----ATTAA-AA-TTAG-AAATGGTT--TTAT-CTTACATA------------------TTGTAAGT-TGTT-CA-TTGATAATTAT-----ACC---AATTGTTACATCGCG--TTATTCACCCAATC-----------------------------TT-TTTT--GA----TT-AGG--T-GAAATAA-TTCAA-TACA-----TTTTT--AATCCTACCTCAAGTC-AAGTAAG-AATACCCGCTGAAC-TT-AAGCATATCAATAAGCGGAGGAAAAGAAACTAAC-TAGGATTCCC-TTAG-TAAC-GGCGAGTGAACTGGGAAAAGCTCAAA-TTTTAAATCAC------------CTCGGTGAATTGTAATTTGAAGAAAGT-G-GTTTGAC-TTTTCAGTTTAATC-TAAATCCTTTGGGA--TGAGGTATCAT-AG-AGGGTGAGAATCCCGTTCTTAATTAAACCTCG---GAT--TGCACTAAATTCACTTTCCAAGAGTCGAGTTG-TTTGGGATTGCAGCTCCAAATGGGTGGTAAATTTCACCTAAGGCTAAATATATGCAAGAGACCGATAGCGAACAAGTACCGT-GAGGGAAAGAG-TGAAAAGTACTTTGAAAAGAGAGTTAAACAGTACGTGAAATTGTTGAAAGGGAAACGATTGAAGTCAGTCATGCTAGTGAGGAATCAACTTGGTGG----------------------------------------------------------TTTACTTCCTTGTGCATTTTCTC--GCTTGGCAGGTTAGCATCAA-TTTTGGTTGTCATCAAAGAATT-GGGGGAATGTGACTTT---TCTTCGGTAAAGTG-TTATAGACCCTGAT-TGATGTGGCGACTGGGATTGAGGTTTGCAGCGGATGCC-TCTTG----TGGCTAGTCACCTGT-CTTCTGACC-GTCACCTC--ACTTTTGACAGC-TTGCTGATGATTGCGGGATTTGGCGACCTAG-AA-GTTAGAGTGAACATAAA-TTCGCTAAGGATGCTGACGTAATGGCTTTAAACGACCCGTCTTGAAACACGGACCAAGGAGTCTAAC-ATATGTGCGAGTGTT

>Acaulospora_gedanensis_1_SSU_ITS_LSU_5_08_2022

CCTAGTAAGCGTGAGTCATCAGCTCACGTTGATTACGTCCCT-GCCCTTTGTACACACCGCCCGTCGCTACTACCGATTGAAT-GGCTTAGTGAGACTCTCGGATTAGGGTTAAGGAATCGGAAACGGTTTCTTTTCCTCGAGAAGTTTGTCAAACTTGGTCATTTAGAGGAAGTAAAAGTCGTAACAAGGTTTCCGTAGGTGAACCTGCGGAAGGATCATTAGAATA-TTCAGGAAT------------------TTATTTTCCT----AATAATAC------TTTATTCAAAAAA-TTTTCAATCTAC----AAAT-TT-----TATTTTTTTAAAAAAAA-AAT-------AACAAATAAATAAAGATAACTTTCAACAACGGATCTCTTGGCTCTTGCATCGATGAAGAACGCAGCGAAATGCGATAAGTAATGTGAATTGCAGAATTCCGTGAATCATTAAATCTTTGAACGCAAATTGCACTTCTT-GGTATTCCGAGGAGTATGCTTGCTTGAGGGTTGGTCTAC-AAAAAAATCGT-GAAAT-------------TT-CTTTTT-------------CGCGGATCTGGG---TTTTCCAAAG---AC----------------------------TTTTTTTGGTAACCTT-AAATTTATCTTAACAAT-TTTAGGT-----ATTAA-AA-TTAG-AAATGGTT--TTAT-CTTACATA------------------TTGTAAGT-TGTT-CA-TTGATAATTAT-----ACC---AATTGTTACATCGCG--TTATTCGTCCAATCT---------------------------TTT-TTTT--GA----TT-AGG--T-GAAATAA-TTCAA-TATA-----TTCTT--AATCCTACCTCAAGTC-AAGTAAG-AATACCCGCTGAAC-TT-AAGCATATCAATAAGCGGAGGAAAAGAAACTAAC-TAGGATTCCC-TTAG-TAAC-GGCGAGTGAACTGGGAAAAGCTCAAA-TTTTAAATCAC------------CTCGGTGAATTGTAATTTGAAGAAAGT-G-TTTTGAC-TTTTCAGTTTAATC-TAAATCCTTTGGGA--TGAGGTATCAT-AG-AGGGTGAGAATCCCGTTCTTGATTAAACCTCG---GAT--TGCACTAAATTCACTTTCCAAGAGTCGAGTTG-TTTGGGATTGCAGCTCCAAATGGGTGGTAAATTTCACCTAAGGCTAAATATATGCAAGAGACCGATAGCGAACAAGTACCGT-GAGGGAAAGA--TGAAAAGAACTTTGAAAAGAGAGTTAAACAGTACGTGAAATTGTTGAAAGGGAAACGATTGAAGTCAGTCATGCTAGTGAGGAATCAACTTGGTGG----------------------------------------------------------TTTACTTCCTCGTGCATTTTCTC--GCTTGGCAGGTTAGCATCAA-TTTTGGTTGTCATCAAAGAATT-GGGGGAATGTGGCTTT---TCTTCGGTAAAGTG-TTATAGACCCTGAT-TGATGTGGCGACTGGGATTGAGGTTTGCAGCGGATGCC-TCTTG----TGGCTAGTCACCTGC-CTTCTGACC-GTCACCTC--GCTTTTGACAGC-TTGCTGATGATTGCGGGATTTGGCGATCTAG-AA-GTTAGAGTGAACATAAA-TTCGCTAAGGATGCTGACGTAATGGCTTTAAACGACCCGTCTTGAAACACGGACCAAGGAGTCTAAC-ATATATGCGAGTGTT

>Acaulospora_gedanensis_5_SSU_ITS_LSU_5_08_2022

CCTAGTAAGCGTGAGTCATCAGCTCACGTTGATTACGTCCCT-GCCCTTTGTACACACCGCCCGTCGCTACTACCGATTGAAT-GGCTTAGTGAGACTCTCGGATTAGGGTTAAGGAATCGGAAACGGTTTCTTTTCCTCGAGAAGTTTGTCAAACTTGGTCATTTAGAGGAAGTAAAAGTCGTAACAAGGTTTCCGTAGGTGAACCTGCGGAAGGATCATTAGAATA-TTCAGGAAT------------------TTATTTTCCT----AATAATAC------TTTATTCAAAAAA-TTTTCAATCTAC----AAAT-TT-----TATTTTTTT-AAAAAAA-AAT-------AACAAATAAATAAAGATAACTTTCAACAACGGATCTCTTGGCTCTTGCATCGATGAAGAACGCAGCGAAATGCGATAAGTAATGTGAATTGCAGAATTCCGTGAATCATTAAATCTTTGAACGCAAATTGCACTTCTT-GGTATTCCGAGGAGTATGCTTGCTTGAGGGTTGGTCTAC-AAAAAAATCGT-GAAAT-------------TT-CTTTTT-------------CGCGGATCTGGG---TTTTCCAAAG---AC----------------------------TTTTTTTGGTAACCTT-AAATTTATCTTAACAAT-TTTAGGT-----ATTAA-AA-TTAG-AAATGGTT--TTAT-CTTACATA------------------TTGTAAGT-TGTT-CA-TTGATAATTAT-----ACC---AATTGTTACATCGCG--TTATTCGTCCAATC-----------------------------TT-TTTT--GA----TT-AGG--T-GAAATAA-TTCAA-TATA-----TTCTT--AATCCTACCTCAAGTC-AAGTAAG-AATACCCGCTGAAC-TT-AAGCATATCAATAAGCGGAGGAAAAGAAACTAAC-TAGGATTCCC-TTAG-TAAC-GGCGAGTGAACTGGGAAAAGCTCAAA-TTTTAAATCAC------------CTCGGTGAATTGTAATTTGAAGAAAGT-G-TTTTGAC-TTTTCAGTTTAATC-TAAATCCTTTGGGA--TGAGGTATCAT-AG-AGGGTGAGAATCCCGTTCTTGATTAAACCTCG---GAT--TGCACTAAATTCACTTTCCAAGAGTCGAGTTG-TTTGGGATTGCAGCTCCAAATGGGTGGTAAATTTCACCTAAGGCTAAATATATGCAAGAGACCGATAGCGAACAAGTACCGT-GAGGGAAAGA--TGAAAAGAACTTTGAAAAGAGAGTTAAACAGTACGTGAAATTGTTGAAAGGGAAACGATTGAAGTCAGTCATGCTAGTGAGGAATCAACTTGGTGG----------------------------------------------------------TTTACTTCCTCGTGCATTTTCTC--GCTTGGCAGGTTAGCATCAA-TTTTGGTTGTCATCAAAGAATT-GGGGGAATGTGGCTTT---TCTTCGGTAAAGTG-TTATAGACCCTGAT-TGATGTGGCGACTGGGATTGAGGTTTGCAGCGGATGCC-TCTTG----TGGCTAGTCACCTGT-CTTCTGACC-GTCACCTC--GCTTTTGACAGC-TTGCTGATGATTGCGGGATTTGGCGACCTAG-AA-GTTAGAGTGAACATAAA-TTCGCTAAGGATGCTGACGTAATGGCTTTAAACGACCCGTCTTGAAACACGGACCAAGGAGTCTAAC-ATGTATGCGAGTGTT

>Acaulospora_gedanensis_4_SSU_ITS_LSU_5_08_2022

CCTAGTAAGCGTGAGTCATCAGCTCACGTTGATTACGTCCCT-GCCCTTTGTACACACCGCCCGTCGCTACTACCGATTGAAT-GGCTTAGTGAGACTCTCGGATTAGGGTTAAGGAATCGGAAACGGTTTCTTTTCCTCGAGAAGTTTGTCAAACTTGGTCATTTAGAGGAAGTAAAAGTCGTAACAAGGTTTCCGTAGGTGAACCTGCGGAAGGATCATTAGAATA-TTCAGGAAT------------------TTATTTTCCT----AATAATAC------TTTATTCAAAAAA-TTTTCAATCTAC----AAAT-TT-----TATTTTTTAAAAAAAAA-AAT-------AACAAATAAATAAAGATAACTTTCAACAACGGATCTCTTGGCTCTTGCATCGATGAAGAACGCAGCGAAATGCGATAAGTAATGTGAATTGCAGAATTCCGTGAATCATTAAATCTTTGAACGCAAATTGCACTTCTT-GGTATTCCGAGGAGTATGCTTGCTTGAGGGTTGGTCTAC-AAAAAAATCGT-GAAAT-------------TT-CTTTTT-------------CGCGGATCTGGG---TTTTCCAAAG---AC----------------------------TTTTTTTGGTAACCTT-AAATTTATCTTAACAAT-TTTAGGT-----ATTAA-AA-TTAG-AAATGGTT--TTAT-CTTACATA------------------TTGTAAGT-TGTT-CA-TTGATAATTAT-----ACC---AATTGTTACATCGCG--TTATTCGTCCAATCT---------------------------TTT-TTTT--GA----TT-AGG--T-GAAATAA-TTCAA-TATA-----TTCTT--AATCCTACCTCAAGTC-AAGTAAG-AATACCCGCTGAAC-TT-AAGCATATCAATAAGCGGAGGAAAAGAAACTAAC-TAGGATTCCC-TTAG-TAAC-GGCGAGTGAACTGGGAAAAGCTCAAA-TTTTAAATCAC------------CTCGGTGAATTGTAATTTGAAGAAAGT-G-TTTTGAC-TTTTCAGTTTAATC-TAAATCCTTTGGGA--TGAGGTATCAT-AG-AGGGTGAGAATCCCGTTCTTGATTAAACCTCG---GAT--TGCACTAAATTCACTTTCCAAGAGTCGAGTTG-TTTGGGATTGCAGCTCCAAATGGGTGGTAAATTTCACCTAAGGCTAAATATATGCAAGAGACCGATAGCGAACAAGTACCGT-GAGGGAAAGA--TGAAAAGAACTTTGAAAAGAGAGTTAAACAGTACGTGAAATTGTTGAAAGGGAAACGATTGAAGTCAGTCATGCTAGTGAGGAATCAACTTGGTGG----------------------------------------------------------TTTACTTCCTCGTGCATTTTCTC--GCTTGGCAGGTTAGCATCAA-TTTTGGTTGTCATCAAAGAATT-GGGGGAATGTGGCTTT---TCTTCGGTAAAGTG-TTATAGACCCTGAT-TGATGTGGCGACTGGGATTGAGGTTTGCAGCGGATGCC-TCTTG----TGGCTAGTCACCTGC-CTTCTGACC-GTCACCTC--GCTTTTGACAGC-TTGCTGATGATTGCGGGATTTGGCGATCTAG-AA-GTTAGAGTGAACATAAA-TTCGCTAAGGATGCTGACGTAATGGCTTTAAACGACCCGTCTTGAAACACGGACCAAGGAGTCTAACAATATGTGCGAGTGTT

>Acaulospora_gedanensis_1_SSU_ITS_LSU_5_09_2022

CCTAGTAAGCGTGAGTCATCAGCTCACGTTGATTACGTCCCT-GCCCTTTGTACACACCGCCCGTCGCTACTACCGATTGAAT-GGCTTAGTGAGACTCTCGGATTAGGGTTAAGGAATCGGAAACGGTTTCTTTTCCTCGAGAAGTTTGTCAAACTTGGTCATTTAGAGGAAGTAAAAGTCGTAACAAGGTTTCCGTAGGTGAACCTGCGGAAGGATCATTAGAATA-TTCAGGAAT------------------TTATTTTCCT----AATAATAC------TTTATTCAAAAAA-TTTTCAATCTAC----AAAT-TT-----TATTTTTTTAAAAAAAA-AAT-------AACAAATAAATAAAGATAACTTTCAACAACGGATCTCTTGGCTCTTGCATCGATGAAGAACGCAGCGAAATGCGATAAGTAATGTGAATTGCAGAATTCCGTGAATCATTAAATCTTTGAACGCAAATTGCACTTCTT-GGTATTCCGAGGAGTATGCTTGCTTGAGGGTTGGTCTAC-AAAAAAATCGT-GAAAT-------------TT-CTTTTT-------------CGCGGATCTGGG---TTTTCCAAAG---AC----------------------------TTTTTTTGGTAACCTT-AAATTTATCTTAACAAT-TTTAGGT-----ATTAA-AA-TTAG-AAATGGTT--TTAT-CTTACATA------------------TTGTAAGT-TGTT-CA-TTGATAATTAT-----ACC---AATTGTTACATCGCG--TTATTCGTCCAATC-----------------------------TT-TTTT--GA----TT-AGG--T-GAAATAA-TTCAA-TATA-----TTCTT--AATCCTACCTCAAGTC-AAGTAAG-AATACCCGCTGAAC-TT-AAGCATATCAATAAGCGGAGGAAAAGAAACTAAC-TAGGATTCCC-TTAG-TAAC-GGCGAGTGAACTGGGAAAAGCTCAAA-TTTTAAATCAC------------CTCGGTGAATTGTAATTTGAAGAAAGT-G-TTTTGAC-TTTTCAGTTTAATC-TARATCCTTTGGGA--TGAGGTATCAT-AG-AGGGTGAGAATCCCGTTCTTGATTAAACCTCG---GAT--TGCACTAAATTCACTTTCCAAGAGTCGAGTTG-TTTGGGATTGCAGCTCCAAATGGGTGGTAAATTTCACCTAAGGCTAAATATATGCAAGAGACCGATAGCGAACAAGTACCGT-GAGGGAAAGA--TGAAAAGAACTTTGAAAAGAGAGTTAAACAGTACGTGAAATTGTTGAAAGGGAAACGATTGAAGTCAGTCATGCTAGTGAGGAATCAACTTGGTGG----------------------------------------------------------TTTACTTCCTCGTGCATTTTCTC--GCTTGGCAGGTTAGCATCAA-TTTTGGTTGTCATCAAAGAATT-GGGGGAATGTGGCTTT---TCTTCGGTAAAGTG-TTATAGACCCTGAT-TGATGTGGCGACTGGGATTGAGGTTTGCAAAGGATGCC-TCTTG----TGGCTAGTCACCTGT-CTTCTGACC-GTCACCTC--GCTTTTGACAGC-TTGCTGATGATTGCGGGATTTGGCGACCTAG-AA-GTTAGAGTGAACATAAA-TTCGCTAAGGATGCTGACGTAATGGCTTTAAACGACCCGTCTTGAAACACGGACCAAGGAGTCTAAC-ATGTGTGCGAGTGTT

>Acaulospora_gedanensis_3_SSU_ITS_LSU_5_09_2022

CCTAGTAAGCGTGAGTCATCAGCTCACGTTGATTACGTCCCT-GCCCTTTGTACACACCGCCCGTCGCTACTACCGATTGAAT-GGCTTAGTGAGACTCTCGGATTAGGGTTAAGGAATCGGAAACGGTTTCTTTTCCTCGAGAAGTTTGTCAAACTTGGTCATTTAGAGGAAGTAAAAGTCGTAACAAGGTTTCCGTAGGTGAACCTGCGGAAGGATCATTAGAATA-TTCAGGAAT------------------TTATTTTCCT----AATAATAC------TTTATTCAAAAAA-TTTTCAATCTAC----AAAT-TT-----TATTTTTTTAAAAAAAA-AAT-------AACAAATAAATAAAGATAACTTTCAACAACGGATCTCTTGGCTCTTGCATCGATGAAGAACGCAGCGAAATGCGATAAGTAATGTGAATTGCAGAATTCCGTGAATCATTAAATCTTTGAACGCAAATTGCACTTCTT-GGTATTCCGAGGAGTATGCTTGCTTGAGGGTTGGTCTAC-AAAAAAATCGK-GAAAT-------------TNATTCTTT-------------CGCGGATCTGGG---TTTTCCAAAG---AC----------------------------TTTTTTTGGTAACCTT-AAATTTATCTTAACAAT-TTTAGGT-----ATTAA-AA-TTAG-AAATGGTT--TTAT-CTTACATA------------------TTGTAAGT-TGTT-CA-TTGATAATTAT-----ACC---AATTGTTACATCGCG--TTATTCGTCCAATC-----------------------------TT-TTTT--GA----TT-AGG--T-GAAATAA-TTCAA-TATA-----TTCTT--AATCCTACCTCAAGTC-AAGTAAG-AATACCCGCTGAAC-TT-AAGCATATCAATAAGCGGAGGAAAAGAAACTAAC-TAGGATTCCC-TTAG-TAAC-GGCGAGTGAACTGGGAAAAGCTCAAA-TTTTAAATCAC------------CTCGGTGAATTGTAATTTGAAGAAAGT-G-TTTTGAC-TTTTCAGTTTAATC-TAAATCCTTTGGGA--TGAGGTATCAT-AG-AGGGTGAGAATCCCGTTCTTGATTAAACCTCG---GAT--TGCACTAAATTCACTTTCCAAGAGTCGAGTTG-TTTGGGATTGCAGCTCCAAATGGGTGGTAAATTTCACCTAAGGCTAAATATATGCAAGAGACCGATAGCGAACAAGTACCGT-GAGGGAAAGA--TGAAAAGAACTTTGAAAAGAGAGTTAAACAGTACGTGAAATTGTTGAAAGGGAAACGATTGAAGTCAGTCATGCTAGTGAGGAATCAACTTGGTGG----------------------------------------------------------TTTACTTCCTCGTGCATTTTCTC--GCTTGGCAGGTTAGCATCAA-TTTTGGTTGTCATCAAAGAATT-GGGGGAATGTGGCTTT---TCTTCGGTAAAGTG-TTATAGACCCTGAT-TGATGTGGCGACTGGGATTGAGGTTTGCAGCGGATGCC-TCTTG----TGGCTAGTCACCTGT-CTTCTGACC-GTCACCTC--GCTTTTGACAGC-TTGCTGATGATTGCGGGATTTGGCGACCTAG-AA-GTTAGAGTGAACATAAA-TTCGCTAAGGATGCTGACGTAATGGCTTTAAACGACCCGTCTTGAAACACGGACCAAGGAGTCTAAC-ATATGTGCGAGTGTT

>Acaulospora_gedanensis_2_SSU_ITS_LSU_5_09_2022

CCTAGTAAGCGTGAGTCATCAGCTCACGTTGATTACGTCCCT-GCCCTTTGTACACACCGCCCGTCGCTACTACCGATTGAAT-GGCTTAGTGAGACTCTCGGATTAGGGTTAAGGAATCGGAAACGGTTTCTTTTCCTCGAGAAGTTTGTCAAACTTGGTCATTTAGAGGAAGTAAAAGTCGTAACAAGGTTTCCGTAGGTGAACCTGCGGAAGGATCATTAGAATA-TTCAGGAAT------------------TTATTTTCCT----AATAATAC------TTTATTCAAAAAA-TTTTCAATCTAC----AAAT-TT-----TATTTTTTTAAAAAAAA-AAT-------AACAAATAAATAAAGATAACTTTCAACAACGGATCTCTTGGCTCTTGCATCGATGAAGAACGCAGCGAAATGCGATAAGTAATGTGAATTGCAGAATTCCGTGAATCATTAAATCTTTGAACGCAAATTGCACTTCTT-GGTATTCCGAGGAGTATGCTTGCTTGAGGGTTGGTCTAC-AAAAAAATCGT-GAAAT-------------TAATTCTTT-------------CGCGGATCTGGG---TTTTCCAAAG---AC----------------------------TTTTTTTGGTAACCTT-AAATTTATCTTAACAAT-TTTAGGT-----ATTAA-AA-TTAG-AAATGGTT--TTAT-CTTACATA------------------TTGTAAGT-TGTT-CA-TTGATAATTAT-----ACC---AATTGTTACATCGCG--TTATTCGTCCAATC-----------------------------TT-TTTT--GA----TT-AGG--T-GAAATAA-TTCAA-TATA-----TTCTT--AATCCTACCTCAAGTC-AAGTAAG-AATACCCGCTGAAC-TT-AAGCATATCAATAAGCGGAGGAAAAGAAACTAAC-TAGGATTCCC-TTAG-TAAC-GGCGAGTGAACTGGGAAAAGCTCAAA-TTTTAAATCAC------------CTCGGTGAATTGTAATTTGAAGAAAGT-G-TTTTGAC-TTTTCAGTTTAATC-TAAATCCTTTGGGA--TGAGGTATCAT-AG-AGGGTGAGAATCCCGTTCTTGATTAAACCTCG---GAT--TGCACTAAATTCACTTTCCAAGAGTCGAGTTG-TTTGGGATTGCAGCTCCAAATGGGTGGTAAATTTCACCTAAGGCTAAATATATGCAAGAGACCGATAGCGAACAAGTACCGT-GAGGGNAAAGATGNAAAAGAACTTTGAAAAGAGAGTTAAACAGTACGTGAAATTGTTGAAAGGGAAACGATTGAAGTCAGTCATGCTAGTGAGGAATCAACTTGGTGG----------------------------------------------------------TTTACTTCCTCGTGCATTTTCTC--GCTTGGCAGGTTAGCATCAA-TTTTGGTTGTCATCAAAGAATT-GGGGGAATGTGGCTTT---TCTTCGGTAAAGTG-TTATAGACCCTGAT-TGATGTGGCGACTGGGATTGAGGTTTGCAGCGGATGCC-TCTTG----TGGCTAGTCACCTGT-CTTCTGACC-GTCACCTC--GCTTTTGACAGC-TTGCTGATGATTGCGGGATTTGGCGACCTAG-AA-GTTAGAGTGAACATAAA-TTCGCTAAGGATGCTGACGTAATGGCTTTAAACGACCCGTCTTGAAACACGGACCAAGGAGTCTAAC-ATATGTGCGAGTGTT

>Acaulospora_gedanensis_6_SSU_ITS_LSU_5_08_2022

CTAGTTAAGCGTGAGTCATCAGCTCACGTTGATTACGTCCCT-GCCCTTTGTACACACCGCCCGTCGCTACTACCGATTGAAT-GGCTTAGTGAGACTCTCGGATTAGGGTTAAGGAATCGGAAACGGTTTCTTTTCCTCGAGAAGTTTGTCAAACTTGGTCATTTAGAGGAAGTAAAAGTCGTAACAAGGTTTCCGTAGGTGAACCTGCGGAAGGATCATTAGAATA-TTCAGGAAT------------------TTATTTTCCT----AATAATAC------TTTATTCAAAAAA-TTTTCAATCTAC----AAAT-TT-----TATTTTTTTAAAAAAAA-AAT-------AACAAATAAATAAAGATAACTTTCAACAACGGATCTCTTGGCTCTTGCATCGATGAAGAACGCAGCGAAATGCGATAAGTAATGTGAATTGCAGAATTCCGTGAATCATTAAATCTTTGAACGCAAATTGCACTTCTT-GGTATTCCGAGGAGTATGCTTGCTTGAGGGTTGGTCTAC-AAAAAAATCGT-GAAAT-------------TT-CTTTTT-------------CGCGGATCTGGG---TTTTCCAAAG---AC----------------------------TTTTTTTGGTAACCTT-AAATTTATCTTAACAAT-TTTAGGT-----ATTAA-AA-TTAG-AAATGGTT--TTAT-CTTACATA------------------TTGTAAGT-TGTT-CA-TTGATAATTAT-----ACC---AATTGTTACATCGCG--TTATTCGTCCAATCT---------------------------TTT-TTTT--GA----TT-AGG--T-GAAATAA-TTCAA-TATA-----TTCTT--AATCCTACCTCAAGTC-AAGTAAG-AATACCCGCTGAAC-TT-AAGCATATCAATAAGCGGAGGAAAAGAAACTAAC-TAGGATTCCC-TTAG-TAAC-GGCGAGTGAACTGGGAAAAGCTCAAA-TTTTAAATCAC------------CTCGGTGAATTGTAATTTGAAGAAAGT-G-TTTTGAC-TTTTCAGTTTAATC-TAAATCCTTTGGGA--TGAGGTATCAT-AG-AGGGTGAGAATCCCGTTCTTGATTAAACCTCG---GAT--TGCACTAAATTCACTTTCCAAGAGTCGAGTTG-TTTGGGATTGCAGCTCCAAATGGGTGGTAAATTTCACCTAAGGCTAAATATATGCAAGAGACCGATAGCGAACAAGTACCGT-GAGGGAAAGA--TGAAAAGAACTTTGAAAAGAGAGTTAAACAGTACGTGAAATTGTTGAAAGGGAAACGATTGAAGTCAGTCATGCTAGTGAGGAATCAACTTGGTGG----------------------------------------------------------TTTACTTCCTCGTGCATTTTCTC--GCTTGGCAGGTTAGCATCAA-TTTTGGTTGTCATCAAAGAATT-GGGGGAATGTGGCTTT---TCTTCGGTAAAGTG-TTATAGACCCTGAT-TGATGTGGCGACTGGGATTGAGGTTTGCAGCGGATGCC-TCTTG----TGGCTAGTCACCTGT-CTTCTGACC-GTCACCTC--GCTTTTGACAGC-TTGCTGATGATTGCGGGATTTGGCGACCTAG-AA-GTTAGAGTGAACATAAA-TTCGCTAAGGATGCTGACGTAATGGCTTTAAACGACCCGTCTTGAAACACGGACCAAGGAGTCTAAC-ATATGTGCGAGTGTT

>Acaulospora_gedanensis_3_SSU_ITS_LSU_12_09_2022

CCTAGTAAGAGTGAGTCATCAGCTCACGTTGATTACGTCCCT-GCCCTTTGTACACACCGCCCGTCGCTACTACCGATTGAAT-GGCTTAGTGAGACTCTCGGATTAGGGTTAAGGAATCGGAAACGGTTTCTTTTCCTCGAGAAGTTTGTCAAACTTGGTCATTTAGAGGAAGTAAAAGTCGTAACAAGGTTTCCGTAGGTGAACCTGCGGAAGGATCATTAGAATA-TTCAGGAAT------------------TTATTTTCCT----AATAATAC------TTTATTAAAAAAA-TTTTCAATCTAC----AAAT-TT-----TATTTTTTTAAAAAAAA-AAT-------AACAAATAAATAAAGATAACTTTCAACAACGGATCTCTTGGCTCTTGCATCGATGAAGAACGCAGCGAAATGCGATAAGTAATGTGAATTGCAGAATTCCGTGAATCATTAAATCTTTGAACGCAAATTGCACTTCTT-GGTATTACGAGGAGTATGCTTGCTTGAGGGTTGGTCTAC-AAAAAAATCGT-GAAAT-------------TT-CTTTTT-------------CGCGGATCTGGG---TTTTCCAAAG---AC----------------------------TTTTTTTGGTAACCTT-AAATTTATCTTAACAAT-TTTAGGT-----ATTAA-AA-TTAG-AAATGGTT--TTAT-CTTACATA------------------TTGTAAGT-TGTT-CA-TTGATAATTAT-----ACC---AATTGTTACATCGCG--TTATTCGTCCAATC-----------------------------TT-TTTT--GA----TT-AGG--T-GAAATAA-TTCAA-TATA-----TTCTT--AATCCTACCTCAAGTC-AAGTAAG-AATACCCGCTGAAC-TT-AAGCATATCAATAAGCGGAGGAAAAGAAACTAAC-TAGGATTCCC-TTAG-TAAC-GGCGAGTGAACTGGGAAAAGCTCAAA-TTTTAAATCAC------------CTCGGTGAATTGTAATTTGAAGAAAGT-G-TTTTGAC-TTTTAAGTTTAATC-TAAATCCTTTGGGA--TGAGGTATCAT-AG-AGGGTGAGAATCCCGTTCTTGATTAAACCTCG---GAT--TGCACTAAATTCACTTTCCAAGAGTAGAGTTG-TTTGGGATTGCAGCTCCAAATGGGTGGTAAATTTCACCTAAGGCTAAATATATGCAAGAGACCGATAGCGAACNAGTACCGT-GAGGGAAAGA--TGAAAAGAACTTTGAAAAGAGAGTTAAACAGTACGTGAAATTGTTGAAAGGGAAACGATTGAAGTAAGTCATGCTAGTGAGGAATCAACTTGGTGG----------------------------------------------------------TTTACTTCCTCGTGCATTTTCTC--GCTTGGCAGGTTAGCATCAA-TTTTGGTTGTCATCAAAGAATT-GGGGGAATGTGGCTTT---TCTTCGGTAAAGTG-TTATAGACCCTGAT-TGATGTGGCGACTGGGATTGAGGTTTGCAGCGGATGCC-TCTTG----TGGCTAGTCACCTGT-CTTCTGACC-GTCACCTC--GCTTTTGACAGC-TTGCTGATGATTGCGGGATTTGGCGACCTAG-AA-GTTAGAGTGAACATAAA-TTCGCTAAGGATGCTGACGTAATGGCTTTAAACGACCCGTCTTGAAACACGGACCAAGGAGTCTAAC-ATGTGTG-GAGTGTT

>HF567941_Acaulospora_pustulata

CCTAGTAAGCGTGAGTCATCAGCTCACGTTGATTACGTCCCT-GCCCTTTGTACACACCGCCCGTCGCTACTACCGATTGAAT-GGCTTAGTGAGACTCTCGGATTAGGGCTAAGGAATCGGAAACGGTTTCTTTTTCTCGAGAAGTTTGTCAAACTTGGTCATTTAGAGGAAGTAAAAGTCGTAACAAGGTTTCCGCAGGTGAACCTGCGGAAGGATCATTAGAATA-TTCGGGAAT------------------TTAATTTCC-------TAATAC------TTTATTCAAAAAA-TTTTCAATCTAC----AAAT--------TTTTTTTTAAAAAAATT-ACA-------AATAAAAATTTAAAGATAACTTTCAACAACGGATCTCTTGGCTCTTGCATCGATGAAGAACGCAGCGAAATGCGATAAGTAATGTGAATTGCAGAATTCCGTGAATCATTAAATCTTTGAACGCAAATTGCACTTCTT-GGTATTCCGAGGAGTATGCTTGCTTGAGGGTTGGTCTAC--AAAAAATCGT-GAAAT-------------TTAATTTTT-------------CGCGGATCTGGG---TTTTCTAAAA---AAG-ACT-----------------------TTTTTTTGGTAACCTT-AAATTTATCTTAACGAT-TTTAGGT-----ATTAA-AA-TTAG-AAATGGTT--TTAT-CTTACATA------------------TTGTAAGT-TGTT-CG-TTGATAATTAT-----ACC---AATTGTTACATCGCG--TTATTCGCCCGATCA--------------------------ATTT-TTTT--TG----AT-TAG--T-GAAATAA-TTCAA-TATA-----TTTTT--AATCCTACCTCAAGTC-AAGTAAGAAATACCCGCTGAAC-TT-AAGCATACCAATAAGCGGAGGAAAAGAAACTAAC-TAGGATTCCC-TTAG-TAAC-GGCGGGTGAACTGGGAAAAGCTCAAA-TTTTAAATCAC------------CTCGGTGAATTGTAATTTGAAGAAAGT-G-TTTTGAC-TTTTCAGTTTAATC-TAAATCCTTTGGGA--TGAGGTATCAT-AG-AGGGTGAGAATCCCGTTCCTGATTAAACCTCG---AAT--TGCACT-AATTCACTTTCCAAGAGTCGAGTTG-TTTGGGATTGCAGCTCCAAATGGGTGGTAAATTTCACCTAAGGCTAAATATATGCAAGAGACCGATAGCGAACAAGTACCGT-GAGGGAAGGA--TGAAAAGAACTTTGAAAAGAGAGTTAAACAGTACGTGAAATTGTTGAAAGGGAAACGATTGAAGTCAGTCATGCTAGTGAGGAATCAACTTGGTGG----------------------------------------------------------TTTACTTCCTCGTGCATTTTCTC--GCTTGGCAGGTTAGCATCAG-TTTTGGTTGTCATCAAAGAATT-GGGGGAATGTGACTTT---TCTTCGGTAAAGTG-TTATAGACCCTGAT-TGATGTGGCGACTGGGATTGAGGTTTGCAGCGGAAGCC-TTTTT----GGGCTAGTCACCTGT-CTTCTGACC-GTCACCTC--GCTTTTGACAGC-TTGCTGATGATTGCGGGATTTGTCGATCTAG-AA-GTTAGAGTGAACATAAA-TTCGCTAAGGATGCTGACGTAATGGCTTTAAACGACCCGTCTTGAAACACGGACCAAGGAGTCTAAC-ATATGTGCGAGTGTT

>HF567939_Acaulospora_pustulata

CCTAGTAAGCGTGAGTCATCAGCTCACGTTGATTACGTCCCT-GCCCTTTGTACACACCGCCCGTCGCTACTACCGATTGAAT-GGCTTAGTGAGACTCTCGGATTAGGGCTAAGGAATCGGAAACGGTTTCTTTTTCTCGAGAAGTTTGTCAAACTTGGTCATTTAGAGGAAGTAAAAGTCGTAACAAGGTTTCCGTAGGTGAACCTGCGGAAGGATCATTAGAATA-TTCGGGAAT------------------TTAATTTCC-------TAATAC------TTTATTCAAAAAA-TTTTCAATCTAC----AAAT-TT-----TTTTTTTTAAAAAAATT-ACA-------AATAAAAATTTAAAGATAACTTTCAACAACGGATCTCTTGGCTCTTGCATCGATGAAGAACGCAGCGAAATGCGATAAGTAATGTGAATTGCAGAATTCCGTGAATCATTAAATCTTTGAACGCAAATTGCACTTCTT-GGTATTCCGAGGAGTATGCTTGCTTGAGGGTTGGTCTAC--AAAAAATCGC-GAAAT-------------TTAATTTTT-------------CGCGGATCTGGG---TTTTCCAAAA---AGACTTT-----------------------TTTTTTTGGTAACCTT-AAATTTATCTTAACAAT-TTTAGGT-----ATTAA-AA-TTAG-AAATGGTT--TTAT-CTTACATA------------------ATGTAAGT-TGTT-CG-TTGATAATTAT-----ACC---AATTGTTACATCGCG--TTATTCGCCCGATCA---------------------------TTT-TTTT--TG----AT-TAG--T-GAAATAA-TTCAA-TATA-----TTTTT--AATCCTACCTCAAGCC-AAGTAAG-AATACCCGCTGAGC-TT-AAGCATATCAATAAGCGGAGGAAAAGAAACTAAC-TAGGATTCCC-TTAG-TAAC-GGCGAGTGAACTGGGAAAAGCTCAAA-TTTTAAATCAC------------CTCGGTGAATTGTAATTTGAAGAAAGT-G-GTTTGAC-TTTTCAGTTTAATC-TAAATCCTTTGGGA--TGAGGTATCAT-AG-AGGGTGAGAATCCCGTTCTTGATTAAACCTCG---AAT--TGCACT-AATTCACTTTCCAAGAGTCGAGTTG-TTTGGGATTGCAGCTCCAAATGGGTGGTAAATTTCACCTAAGGCTAAATATATGCAAGAGACCGATAGCGAACAAGTACCGT-GAGGGAAAGA--TGAAAAGAACTTTGAAAAGAGAGTTAAACAGTACGTGAAATTGTTGAAAGGGAAACGATTGAAGTCAGTCATGCTAGTGAGGAATCAACTTGGTGG----------------------------------------------------------TTTACTTCCTTGTGCATTTTCTC--GCTTGGCAGGTTAGCATCAA-TTTTGGTTGTCATCAAAGAATT-GGGGGAATGTGACTTT---TCTTCGGTAAAGTG-TTATAGACCCTGAT-TGATGTGGCGACTGGGATTGAGGTTTGCAGCGGAAGCC-TTTT------GGCTAGTCACCTGT-CTTCTGATC-GTCACCTC--GCTTTTGACAGC-TTGCTGATGATTGCGGGATTTGGCGATCTAG-AA-GTTAGAGTGAACATAAA-TTCGCTAAGGATGCTGACGTAATGGCTTTAAACAACCCGTCTTGAAACACGGACCAAGGAGTCTAAC-ATATGTGCGAGTGTT

>HF567938_Acaulospora_pustulata

CCTAGTAAGCGTGAGTCATCAGCTCACGTTGATTACGTCCCT-GCCCTTTGTACACACCGCCCGTCGCTACTACCGATTGAAT-GGCTTAGTGAGACTCTCGGATTAGGGCTAAGGAATCGGAAACGGTTTCTTTTTCTCGAGAAGTTTGTCAAACTTGGTCATTTAGAGGAAGTAAAAGTCGTAACAAGGTTTCCGTAGGTGAACCTGCGGAAGAATCATTAGAATA-TTCGGGAAT------------------TTAATTTCC-------TAATAC------TTTATTCAAAAAA-TTTTCAATCTAC----AAAT-TT-----TTTTTTTTAAAAAAATT-ACA-------AATAAAAATTTAAAGATAACTTTCAACAACGGATCTCTTGGCTCTTGCATCGATGAAGAACGCAGCGAAACGCGATAAGTAATGTGAATTGCAGGATTCCGTGAATCATTAAATCTTTGAACGCAAATTGCACTTCTT-GGTATTCCGAGGAGTATGCTTGCTTGAGGGTTGGTCTAC--AAAAAATCGC-GAAAT-------------TTAATTTTT-------------CGCGGATCTGGG---TTTTCCAAAA---AG--ACT-----------------------TTTTTTTGGTAACCTT-AAATTTATCTTAACAAT-TTTAGGT-----ATTAA-AA-TTAG-AAATGGTT--TTAT-CTTACATA------------------TTGTAAGT-TGTT-CG-TTGATAATTAT-----ACC---AATTGTTACATCGCG--TTATTCGCCCGATCA---------------------------TTT-TTTT--TG----AT-TAG--T-GAAATAA-TTCAA-TATA-----TTTTT--AATCCTACCTCAAGTC-AAGTAAG-AATACCCGCTGAAC-TT-AAGCATATCAATAAGCGGAGGAAAAGAAACTAAC-TAGGATTCCC-TTAG-TAAC-GGCGAGTGAACTGGGAAAAGCTCAAA-TTTTAAATCAC------------CTCGGTGAATTGTAATTTGAAGAAAGT-G-GTTTGAC-TTTTCAGTTTAATC-TAAATCCTTTGGGA--TGAGGTATCAT-AG-AGGGTGAGAATCCCGTTCTTGATTAAACCTCG---AAT--TGCACT-AATTCACTTTCCAAGAGTCGAGTTG-TTTAGGATTGCAGCTCCAAATGGGTGGTAAATTTCACCTAAGGCTAAATATATGCAAGAGACCGATAGCGAACAAGTACCGT-GAGGGAAAGA--TGAAAAGAACTTTGAAAAGAGAGTTAAACAGTACGTGAAATTGTTGAAAGGGAAACGATTGAAGTCAGTCATGCTAGTGAGGAATCAACTTGGTGG----------------------------------------------------------TTTACTTCCTTGTGCATTTTCTC--GCTTGGCAGGTTAGCATCAA-TTTTGGTTGTCATCAAAGGATT-GGGGGAATGTGACTTT---TCTTCGGTAAAGTG-TTATGGACCCTGAT-TGATGTGGCGACTGGGATTGAGGTTTGCAGCGGAAGCC-TTTT------GGCTAGTCACCTGT-CTTCTGATC-GTCACCTC--GCTTTTGACAGC-TTGCTGATGATTGCGGGATTTGGCGATCTAG-AA-GTTAGAGTGAACATAAA-TTCGCTAAGGATGCTGACGCAATGGCTTTAAACGACCCGTCTTGAAACACGGACCAAGGAGTCTAAC-ATATGTGCGAGTGTT

>Acaulospora_brasiliensis_FN825912

-CTAGTAAGCGTGAGTCATCAGCTCACGTTGATTACGTCCCT-GCCCTTTGTACACACCGCCCGTCGCTACTACCGATTGAAT-GGCTTAGTGAGACCCTCGGATCGACTCATGGAAACCTTCACGGGTATCTATTTGTTGAAAAGTTGGTCAAACTTGGTCATTTAGAGGAAGTAAAAGTCGTAACAAGGTTTCCGTAGGTGAACCTGCGGAAGGATCATTAAAAAA-TTGAGGTAT------------------TAGTACCTC----------TTT------TGTATTT-AAAAC-CTTACTCTCATG----AAAC-CT----------------AAAAAA-TTT-------TATATATGAAAATGAAAAACTTTCAACAATGGATCTCTTGGTTCTCGCATCGATGAAGAACGCAGCGAAATGCGATAAGTAATATGAATTGCAGAATTCCGTGAATCATTAAATCTTTGAACGCAAATTGCACTTCTT-GGTATTCCGAGGAGTATGCTTGCTTGAGGGTTGGTCTAC-AAAAAAATCGT-GAAAT-------------TAATTTTTT-------------CGCGGACCTGGG---TTTTCCAAAG---AC--TTT-----------------------TTTTTTTGGTAACCTT-AAATTTATCTTAACAAT-TTTAGGT-----ATTAA-AA-TTAG-AAATGGTT--TTAT-CTTACATA------------------TTGTAAGT-TGTT-CA-TTGATAATTAT-----ACC---AATTGTTACATCGCG--TTATTCACCCAATC-----------------------------TT-TTTT--GA----TT-AGG--T-GAAATAA-TTCAA-TACA-----TTTTT--AATCCTACCTCAAGTC-AAGTAAG-AATACCCGCTGAAC-TT-AAGCATATCAATAAGCGGAGGAAAAGAAACTAAC-TAGGATTCCC-TTAG-TAAC-GGCGAGTGAACTGGGAAAAGCTCAAA-TTTTAAATCAC------------CTCGGTGAATTGTAATTTGAAGAAAGT-G-GTTTGAC-TTTTCAGTTTAATC-TAAATCCTTTGGGA--TGAGGTATCAT-AG-AGGGTGAGAATCCCGTTCTTGATTAAACCTCG---GAT--TGCACTAAATTCACTTTCTAAGAGTCGAGTTG-TTTGGGATTGCAGCTCCAAATGGGTGGTAAATTTCACCTAAGGCTAAATATATGCAAGAGACCGATAGCGAACAAGTACCGT-GAGGGAAAGA--TGAAAAGTACTTTGAAAAGAGAGTTAAACAGTACGTGAAATTGTTGAAAGGGAAACGATTGAAGTCAGTCATGCTAGTGAGGAATCAACTTGGTGG----------------------------------------------------------TTTACTTCCTTGTGCATTTTCTC--GCTTGGCAGGTTAGCATCAA-TTTTGGTTGTCATCAAAGAATT-GGGGGAATGTGACTTT---TCTTCGGTAAAGTG-TTATAGACCCTGAT-TGATGTGGCGACTGGGATTGAGGTTTGCAGCGGATGCC-TCTTG----TGGCTAGTCACCTAT-CTTCTGACC-GTCACCTC--ACTTTTGACAGC-TTGCTGATGATTGCGGGATTTGGCGACCTAG-AA-GTTAGAGTGAACATAAA-TTCGCTAAGGATGCTGACGTAATGGCTTTAAACGACCCGTCTTGAAACACGGACCAAGGAGTCTAAC-ATATGTGCGAGTGTT

>FR681927_Acaulospora_alpina

CCTAGTAAGCGTGAGTCATCAGCTCACGTTGATTACGTCCCT-GCCCTTTGTACACACCGCCCGTCGCTACTACCGATTGAAT-GGCTTAGTGAGACTCTCGGATTAGGATTTAGAGATCGGCAACGGTTTCTATTTATTGAGAAGTTTGTCAAACTTGGTCATTTAGAGGAAGTAAAAGTCGTAACAAGGTTTCCGTAGGTGAACCTGCGGAAGGATCATTAGAATA-CTAGGAATT------------------TATTTTCCTAATACTTTATTAC------TTTATTT-AAAAA-TTTCCAATCTAC----AAAATTT-----TTTTTT-----TAAAAT-AAG-------AATATAAATCATAAGATAACTTTTAACAACGGATCTCTTGGCTCTTGCATCGATGAAGAACGCAGCGAAATGCGATAAGTAATGTGAATTGCAGAATTCCGTGAATCATTAAATCTTTGAACGCAAATTGCACTTCTT-GGTATTCCGAGGAGTATACTTGCTTGAGGGTTGGTCTAC-AAAAAAATCGT-GAAAT--------------TTTTTTTT-------------CGCGGACCTGGG---TTTTCCAAAG----------------------------------AATTTTGGTAACCTT-AAATTTATCTTAACAATCTTTAGGT-----ATTAA-TA-TTAG-AAATGG-T--TTAT-CTTACATA------------------TTGTAA----GTT-CA-TTGATAATTAT-----ACC---AATTGTTACATCGCG--TTATTCGCCTAGTA----------------------------TTT-TTTC--GA----TT-AGG--T-GAAATAA-TTCAA-C--A-----TTTTT--AATCCTACCTCAAGTC-AAGTAAG-AATACCCGCTGAAC-TT-AAGCATATCAATAAGCGGAGGAAAAGAAACTAAC-TAGGATTCCC-TTAG-TAAC-GGCGAGTGAACTGGGAAGAGCTCAAA-TTTTAAATCAC------------CTTGGTGAATTGTAATTTGAAGAAAGT-G-TTTTGAC-ATTTCAGTTTAATC-TAAATCCTTTGGGA--TGAGGTATCAC-AG-AGGGTGAGAATCCCGTATGTGATTAGACTTCG---GAA--TGCATT-AATTCACTTTCTAAGAGTCGAGTTG-TTTGGGATTGCAGCTCAAAAAGGGTGGTAAATTTCACCTAAGGCTAAATATATGCAAGAGACCGATAGCGAACAAGTACCGT-GAGGGAAAGA--TGAAAAGAACTTTGAAAAGAGAGTTAAACAGTACGTGAAATTGTTAAAAGGGAAACGATTGAAGTCAGTCATGCTAGTAAGGAATCAACTTGGTGGAG-------TGAGTTTGGGTTTGTAAAGTAGGGTAACCACTTGCATATCTTTT-TTCTCTCCCTTCCTTGTGTACTTTCTT--TCTTGGCAAGTTAGCATCAA-TTTTAGTTGTCATCAAAGAATT-GGGGAAATGTGGCTTC---TCTTCGAGGAAGTGTTTATAGACCCTGAT-AGATGTGGCGACTGGGATTGAGGATTGCAGCGGATGCC-TTT-------GGCTAGTCTCCTGT-CTTCTGACG-GTCACCTC--GCTTTTGACAGC-TTGCTGATGATTGTGGGATTTGACAGTTTAG-AA-GTTAGAGTGAACATAAA-TTCGCTAAGGATGCTGACGTAATGGCTTTAAACGACCCGTCTTGAAACACGGACCAAGGAGTCTAAC-ATATGTGCGAGTGTT

>FR681930_Acaulospora_alpina

CCTAGTAAGCGTGAGTCATCAGCTCACGTTGATTACGTCCCT-GCCCTTTGTACACACCGCCCGTCGCTACTACCGATTGAAT-GGCTTAGTGAGACTCTCGGATTAGGATTTAGAGATCGGCAACGGTTTCTATTTATTGAAAAGTTTGTCAAACTTGGTCATTTAGAGGAAGTAAAAGTCGTAACAAGGTTTCCGTAGGTGAACCTGCGGAAGGATCATTAGAATA-CTGGGAATT------------------TATTTTTCC-------TAATAC------TTTATTC-AAAAA-TTTCCAATCTAC----AAAA-AT-----TTTTTT-----TAAAAT-AAG-------AATATAAATCATAAGATAACTTTTAACAACGGATCTCTTGGCTCTTGCATCGATGAAGAACGCAGCGAAATGCGATAAGTAATGTGAATTGCAGAATTCCGTGAATCATTAAATCTTTGAACGCAAATTGCACTTCTT-GGTATTCCGAGGAGTATATTTGCTTGAGGGTTGGTCTACAAAAAAAATCGT-GAAAA--------------TTTTTTTT-------------CGCGGACCTGGG---TTTTCCAAAG---------------------------------AATTTTTGGTAACCTT-AAATTTATCTTAACAATCTTTAGGT-----ATTAA-TA-TTAG-AAATGG-T--TTAT-CTTACATA------------------TTGTAA----GTT-CA-TTGATAATTAT-----ACC---AATTGTTACATCGCG--TTATTCGCCTAGTA-----------------------------TT-TTTT--GA----TT-AGG--T-GAAATAA-TTCAA-C--A-----TTTTT--AATCTTACCTCAAGTC-AAGTAAG-AATACCCGCTGAAC-TT-AAGCATATCAATAAGCGGAGGAAAAGAAACTAAC-TAGGATTCCC-TTAG-TAAC-GGCGAGTGAACTGGGAAGAGCTCAAA-TTTTAAATCAC------------CTTGGTGAATTGTAATTTGAAGAAAGT-G-TTTTGAC-ATTTCAGTTTAATC-TAAATCCTTTGGGA--TGAGGTATCAC-AG-AGGGTGAGAATCCCGTATGTGATTAGACTTCG---AAA--TGCATT-AATTCACTTTCTAAGAGTCGAGTTG-TTTGGGATTGCAGCTCAAAAAGGGTGGTAAATTTCACCTAAGGCTAAATATATGCAAGAGACCGATAGCGAACAAGTACCGT-GAGGGAAAGA--TGAAAAGAACTTTGAAAAGAGAGTTAAACAGTACGTGAAATTGTTAAAAGGGAAACGATTGAAGTCAGTCATGCTAGTAAGGAATCAACTTGGTGGAG-------TGAGTTTGGGTTTGTAAAGTAGGGTAACCACTTGCATATCTTTT-TTCTCTCCCTTCCTTGTGTACTTTCTT--TCTTGGCAAGTTAGCATCAA-TTTTAGTTGTCATCAAAGAATT-GGGGAAATGTGGCTTC---TCTTCGGGGAAGTGTTTATAGACCCTGAT-AGATGTGGCGACTGGGATTGAGGATTGCAGCGGATGCC-TTT-------GGCTAGTCACCTGT-CTTCTGACG-GTCACCTC--GCTTTTGATAGC-TTGCTGATGATTGTGGGATTTGACAGTTTAG-AA-GTTAGAGTGAACATAAA-TTCGCTAAGGATGCTGACGTAATGGCTTTAAACGACCCGTCTTGAAACACGGACCAAGGAGTCTAAC-ATATGTGCGAGTGTT

>Acaulospora_fanjing_MW723429

CCTAGTAAGCGTGAGTCATCAGCTCACGTTGATTACGTCCCT-GCCCTTTGTACACACCGCCCGTCGCTACTACCGATTGAAT-GGCTTAGTGAGACTCTCGGATTAGGGTTTAGAGATCGGCAACGGTTTCTTTATCTTGAGAAGTTTGTCAAACTTGGTCATTTAGAGGAAGTAAAAGTCGTAACAAGGTTTCCGTAGGTGAACCTGCGGAAGGATCATTAGAATA-ATGGGAAAT-------------------TATTTTCC-------TAATAC------TTTATTC-AAAAA-ATCTCATTCTAT----AAAT-TT-----TTTTTT-----AAAATA-TAA-------AACTTAAATTATAAGATAACTTTCAACAACGGATCTCTTGGCTCTTGCATCGATGAAGAACGCAGCGAAATGCGATAAGTAATGTGAATTGCAGAATTCCGTGAATCATCAAATCTTTGAACGCAAATTGCACTTCTT-GGTATTCCGAGGAGTATGCTTGCTTGAGGGTTGGTCTAC---AAAAATCGT-GAAAAAAAAATATATATATTTTTTTTT-------------CGCGGATCTGGG---TTTTCCGAGT---TT--TTA-----------------------AGACTTTGGTGACTTA-AAATTTATCTTAACAATCTTTAGGT-----ATTGA-CA-TTAG-AAATGT-T--CTAT-CTTACATA------------------TTGTAA----GTT-CA-TTGATAATTAT-----ACC---AATTGTTACATCGCG--TTATTCACCTAGTA----------------------------TCT-TTTT--GA----TT-AGG--T-GAAATAA-TTCAA-CATA-----TTTTT--AATCCTACCTCAAGTC-AAGTAAG-AATACCCGCTGAAC-TT-AAGCATATCAATAAGCGGAGGAAAAGAAACTAAC-TAGGATTCCC-TTAG-TAAC-GGCGAGTGAACTGGGAAGAGCTCAAA-TTTTAAATCAC-----------TTTTGGTGAATTGTAATTTGAAGAATGT-G-TTTTGAC-GTTTCGGTTTATTC-TAAATCCTTTGGGA--TGAGGTATCAT-AG-AGGGTGAGAATCCCGTTTGTGATTAAGCCTAG---GAATTGTCATTTAATTCATTTTCTAAGAGTCGAGTTG-TTTGGGATTGCAGCTCAAAATGGGTGGTAAATTTCACCTAAGGCTAAATATATGCAAGAGACCGATAGCGAACAAGTACCGT-GAGGGAAAGA--TGAAAAGAACTTTGAAAAGAGAGTTAAACAGTACGTGAAATTGTTGAAAGGGAAACGATTGAAGTCAGTCATGCTAGTGAGGAATCAACTTGGTGGTG-------TGAGTT---------------------------------CTTTT-TTCTTGCTTTTCCTTGTGTACTTTCTT--GCTTGGCAGGTTAGCATCAA-TTTTAGTTGTCATTAAAGAATT-GGGGAAATGTGGCTTC---TCTTCGGAGGAGTGTTTATAGACCCTGGT-AGATGTGACGACTGGGATTGAGGATTGCAGCGAATGCC-TTT------AGGCTAGTCACCTGT-CTTCTGACG-GTCACCTC--GCTTTTGACAGC-TTGCTGATGATTGTGGGATTTGGCAGTCTAG-AA-GTTAGAATGATCATAAA-TTCGCTAAGGATGCTGACGTAATGGCTTTAAACGACCCGTCTTGAAACACGGACCAAGGAGTCTAAC-ATGTGTGCGAGTGTT

>Acaulospora_fanjing_MW723431

CCTAGTAAGCGTGAGTCATCAGCTCACGTTGATTACGTCCCT-GCCCTTTGTACACACCGCCCGTCGCTACTACCGATTGAAT-GGCTTAGTGAGACTCTCGGATTAGGGTTTAGAGATCGGCAACGGTTTCTTTATCTTGAGAAGTTTGTCAAACTTGGTCATTTAGAGGAAGTAAAAGTCGTAACAAGGTTTCCGTAGGTGAACCTGCGGAAGGATCATTAGAATA-ATGGGAAAT-------------------TATTTTCC-------TAATAC------TTTATTC-AAAAA-ATCTCATTCTAT----AAAT-TT-----TTTTTT-----AAAATA-TAA-------AACTTAAATTATAAGATAACTTTCAACAACGGATCTCTTGGCTCTTGCATCGATGAAGAACGCAGCGAAATGCGATAAGTAATGTGAATTGCAGAATTCCGTGAATCATCAAATCTTTGAACGCAAATTGCACTTCTT-GGTATTCCGAGGAGTATGCTTGCTTGAGGGTTGGTCTAC---AAAAATCGT-GAAAAAAAAATATATATATTTTTTTTT-------------CGCGGATCTGGG---TTTTCCGAGT---TT--TTA-----------------------AGACTTTGGTGACTTA-AAATTTATCTTAACAATCTTTAGGT-----ATTGA-CA-TTAG-AAATGT-T--CTAT-CTTACATA------------------TTGTAA----GTT-CA-TTGATAATTAT-----ACC---AATTGTTACATCGCG--TTATTCACCTAGTA----------------------------TCT-TTTT--GA----TT-AGG--T-GAAATAA-TTCAA-CATA-----TTTTT--AATCCTACCTCAAGTC-AAGTAAG-AATACCCGCTGAAC-TT-AAGCATATCAATAAGCGGAGGAAAAGAAACTAAC-TAGGATTCCC-TTAG-TAAC-GGCGAGTGAACTGGGAAGAGCTCAAA-TTTTAAATCAC-----------TTTTGGTGAATTGTAATTTGAAGAATGT-G-TTTTGAC-GTTTCGGTTTATTC-TAAATCCTTTGGGA--TGAGGTATCAT-AG-AGGGTGAGAATCCCGTTTGTGATTAAGCCTAG---GAATTGTCATTTAATTCATTTTCTAAGAGTCGAGTTG-TTTGGGATTGCAGCTCAAAATGGGTGGTAAATTTCACCTAAGGCTAAATATATGCAAGAGACCGATAGCGAACAAGTACCGT-GAGGGAAAGA--TGAAAAGAACTTTGAAAAGAGAGTTAAACAGTACGTGAAATTGTTGAAAGGGAAACGATTGAAGTCAGTCATGCTAGTGAGGAATCAACTTGGTGGTG-------TGAGTT---------------------------------CTTTT-TTCTTGCTTTTCCTTGTGTACTTTCTT--GCTTGGCAGGTTAGCATCAA-TTTTAGTTGTCATTAAAGAATT-GGGGAAATGTGGCTTC---TCTTCGGAGGAGTGTTTATAGACCCTGGT-AGATGTGACGACTGGGATTGAGGATTGCAGCGAATGCC-TTT------AGGCTAGTCACCTGT-CTTCTGACG-GTCACCTC--GCTTTTGACAGC-TTGCTGATGATTGTGGGATTTGGCAGTCTAG-AA-GTTAGAATGATCATAAA-TTCGCTAAGGATGCTGACGTAATGGCTTTAAACGACCCGTCTTGAAACACGGACCAAGGAGTCTAAC-ATGTGTGCGAGTGTT

>HF567933_Acaulospora_tortuosa

CCTAGTAAGCGTGAGTCATCAGCTCACGTAGATTACGTCCCT-GCCCTTTGTACACACCGCCCGTCGCTACTACCGATTGAAT-GGCTTAGTGAGACTCTCGGATCAGGATTAAGTGATCGGAAACGTTTTCTTTGTCTTAAGAAGTTTGTCAAACTTGGTCATTTAGAGGAAGTAAAAGTCGTAACAAGGTTTCCGTAGGTGAACCTGCGGAAGGATCATTAAGAATATCAGGAAAT-----------------TTATTTTTCC-------TAATAC------TTTATTC-AAATAATTTTCAATCTAC----AATA-TT-----TTTTTT-----AAAAAA-AAT-------ATATAAAATACAAAGACAACTTTCAACAACGGATCTCTTGGCTCTTGCATCGATGAAGAACGCAGCGAAATGCGATAAGTAATGTGAATTGCAGGATTCCGTGAATCATTAAATCTTTGAACGCAAATTGCACCTCTT-GGTATTCCGAGGAGTATGCTTGCTTGAGGGTTGGTCTAC--AAAAAATCGT-GAAAAAAAA------AAATTTTTTTTT-------------CGCGGATCTGGG---TTTTCCAAGA---AT--CAT-----------------------TTTTTTTGGTAACCTT-AAATTTAACTTAACAAT-CTTAGGT-----ATTGA-TA-TTAG-AAATGTTT--TTAT-CTTACATA------------------TTGTAA----GTT-CA-TTGATAATTAT-----ACC---AATTGTTACATCGCG--TTATTCGCCTA--------------------------------TC-TTTC--GA----AT-GGG--T-GAAATAA-TTCAA-ATTTT----TTTTT--AATCCTACCTCAAGTC-AAGTAAG-AATACCCGCTGAAC-TT-AAGCATATCAATAAGCGGAGGAAAAGAAACTAAC-TAGGATTCCC-TTAG-TAAC-GGCGAGTGAACTGGGAAGAGCTCAAA-TTTTAAATCAC-----------CTTTGGTGAATTGTATTTTGAAGAAAGT-G-TTTGGAC-TTTTCAGTTTAATCTTAAATCCTTTGGGA--TGAGGTATCAT-AG-AGGGTGGGAATCCCGTTTGTGATTAAACTTTG---GAA--TGCATT-AATTCACTTTCTAAGAGTCGAGTTG-TTTGGGATTGCGGCTCAAAATGGGTGGTAAATTTCACCTAAGGCTAAATATATGCAAGAGACCGATAGCGAACAAGTACCGT-GAGGGAAAGA--TGAAAAGAACTTTGAACAGAGAGTTAAACAGTACGTGAAATTGTTGAAAGGGAAACGATTGAAGTCAGTCATGCTAATGAGGAATCAACAAGTTGG---------------------------------------------------------TTTTACTTCCTTGTGTACTTTCTCA----TGGCAGGTTAGCATCAATTTTTGGTTATCATCAAAGAATT-GGGGGAATGTGGCTTC---TCTTCGGAGAAGTGTTTATAGACCCTGGT-AGATGTGGTGACTGGGATTGAGGATTGCAGCGAATGCT-TTT-------GGCTAGTCACCTAT-CTTCTGACT-GTCACCTC--ACTTTTGACAGC-TTGCTAATGATCGTGGGATTTGGTGGTTTAG-AAGGTTAGAGTGTACATAAA-TTCGCTAAGGATGCTGACGTAATGGCTTTAAACGACCCGTCTTGAGACACGGACCAAGGAGTCTAAC-ATATGTGCGAGTGTT

>HF567936_Acaulospora_tortuosa

CCTAGTAAGCGTGAGTCATCAGCTCACGTTGATTACGTCCCT-GCCCTTTGTACACACCGCCCGTCGCTACTACCGATTGAAT-GGCTTAGTGAGACTCTCGGATCAGGATTAAGTGATCGGAAACGTTTTCTTTGTCTTAAGAAGTTTGTCAAACTTGGTCATTTAGAGGGAGTAAAAGTCGTAACAAGGTTTCCGTAGGTGAACCTGCGGAAGGATCATTAAGAATATCAGGAAAT-----------------TTATTTTTCC-------TAATAC------TTTATTC-AAATAATTTCCAATCTAC----AA----------TTTTTT-----ATAAAA-AAA-------ATTTAAAATAAAAAGATAACTTTCAACAACGGATCTCTTGGCTCTTGCATCGATGAAGAACGCAGCGAAATGCGATAAGTAATGTGAATTGCAGAATTCCGTGAATCATTAAATCTTTGAACGCAAATTGCACTTCTT-GGTATTCCGAGGAGTATGCTTGCTTGAGGGTTGGTCTAC--AAAGAATCGT-GAAAAAAAA------AATTTTTTTTTT-------------CGCGGATCTGGG---TTTTCCAAGA---AT--CAT-----------------------TTTTTTTGGTAACCTT-AAATTTATCTTAACAAT-CTTAGGT-----ATTGA-TA-TTAG-AAATGTTT--TTAT-CTTACATA------------------TTGTAA----GTT-CATTTGATAATTAT-----ACC---AATTGTTACACCGCG--TTATTCGCCTA--------------------------------TC-TTTC--GA----TT-GGG--T-GAAATGA-TTCAA-TTTTT----TTTTA--AATCCTACCTCAAGTC-AAGTAAG-AATACCCGCTGAAC-TTAAAGCATATCAATAAGCGGAGGAAAAGAAACTAAC-TAGGATTCCC-TTAG-TAAC-GGCGAGTGAACTGGGAAGAGCTCAAA-TTTTAAATCAC-----------CTTTGGTGAATTGTATTTTGAAGAAAGT-G-TTTGGAC-TTTTCAGTTTAATCTTAAATCCTTTGGGA--TGAGGTATCAT-AG-AGGGTGAGAATCCCGTTTGTGATTAAACTTTG---GAA--TGCATT-AATTCACTTTCTAAGAGTCGAGTTG-TTTGGGATTGCAGCTCAAAATGGGTGGTAAATTTCACCTAAGGCTAAATATATGCAAGAGACCGATAGCGAACAAGTACCGT-GAGGGAAAGA--TGAAAAGAACTTTGAAAAGAGAGTTAAACAGTACGTGAAATTGTTGAAAGGGAAACGATTGAAGTCAGTCATGCTAATGAGGAATCAACAAGTTGG---------------------------------------------------------TTTTACTTCCTTGTGTACTTTCTCA----TGGCAGGTTAGCATCAATTTTTGGTTATCATCAAAGAATT-GGGGGAATGTGGCTTC---TCTTCGGAGAAGTGTTTATAGACCCTGGT-AGATGTGGTGACTGGGATTGAGGATTGCAGCGAATGCT-TTT-------GGCTAGTCACCTAT-CTTCTGACT-GTCACCTC--ACTTTTGACAGC-TTGCTAATGATCGTGGGATTTGGTGGTTTAG-AAGGTTAGAGTGTACATAAA-TTCGCTAAGGATGCTGACGTAATGGCTTTAAACGACCCGTCTTGAAACACGGACCAAGGAGTCTAAC-ATATGTGCGAGTGTT

>GU326346_Acaulospora_colliculosa

----------------------------------------------------------------------------------------------------------------------------------------------------------------------------------------------------------------------------------------------------------------------------------------------------------------------------------------------------------------------------------------------------------------------------------------------------------------------------------------------------------------------------------------------------------------------------------------------------------------------------------------------------------------------------------------------------------------------------------------------------------------------------------------------------------------------------------------------------------------------------------------------------------------------------------------------------------------------------------------------------AAAGAAACTAAC-TAGGATTCCC-TTAG-TAAC-GGCGAGTGAACTGGGAAGAGCTCAAA-TTTTAAATCAC------------TTTGGTGAATTGTAATTTGAAGAAAGT-GCTTTTGAC-TTTTCGGTTTATTC-TAAATCCTTTGGGA--TGAGGTATCAT-AG-AGGGCGAGAATCCCGTTCATGATTAAACCTTGA--AAA--TGCATTAGATTCACTTTCTAAGAGTCGAGTTG-TTTGGGATTGCAGCTCAAAATGGGTGGTAAATTTCACCTAAGGCTAAATATATGCAAGAGACCGATAGCGAACAAGTACCGT-GAGGGAAAGA--TGAAAAGAACTTTGAAAAGAGAGTTAAACAGTACGTGAAATTGTTGAAAGGGAAACGATTAAAGTCAGTCATGCCTGTGAGTAATCAACTTGGTGG---------------------------------------------------------TTTTACTTCCTTGTGTACTTTCTC--ACTTGGCAGGTTAACATCAA-TTTTGGTTGTCATCAAATAATT-GGGGAAATGTGGCTTC---TCTTCGGAAAAGTGTTTATAGTCCCTGAT-TAATGTGGTAACCGGGATTGAGGATTGCAGTGAATGCC-TTTT-----TGGCTAGTCACCTAT-CTTCTGACT-ATCGTCTT--GCTTTTTTCAGC-TTGCTGATGATTGTGAGATTTGGTGGTTTAG-AT-GTTAGAGTGTACATAAA-TTCACTAAGGATGTT--------------------------------------------------------------------

>GU326352_Acaulospora_colliculosa

----------------------------------------------------------------------------------------------------------------------------------------------------------------------------------------------------------------------------------------------------------------------------------------------------------------------------------------------------------------------------------------------------------------------------------------------------------------------------------------------------------------------------------------------------------------------------------------------------------------------------------------------------------------------------------------------------------------------------------------------------------------------------------------------------------------------------------------------------------------------------------------------------------------------------------------------------------------------------------------------------AAAGAAACTAAC-TAGGATTCCC-TTAG-TAAC-GGCGAGTGAACTGGGAAGAGCTCAAA-TTTTAAATCAC------------TTTGGTGAATTGTAATTTGAAGAAAGT-GCTTTTGAC-TTTTCGGTTTATTC-TAAATCCTTTGGGA--TGAGGTATCAT-AG-AGGGTGAGAATCCCGTTCATGATTAAACCTTGA--AAA--TGCACTAGATTCACTTTCTAAGAGTCGAGTTG-TTTGGGATTGCAGCTCAAAATGGGTGGTAAATTTCACCTAAGGCTAAATATATGCAAGAGACCGATAGCGAACAAGTACCGT-GAGGGAAAGA--TGAAAAGAACTTTGAAAAGAGAGTTAAACAGTACGTGAAATTGTTGAAAGGGAAACGATTAAAGTCAGTCATGCCTGTGAGTAATCAACTTGGTGG---------------------------------------------------------TTTTACTTCCTTGTGTACTTTCTC--ACTTGGCAGGTTAACATCAA-TTTTGGTTGTCATCAAATAATT-GGGGAAATGTGGCTTC---TCTTCGGAAAAGTGTTTATAGTCCCTGAT-TAATGTGGTAACCGGGATTGAGGATTGCAGTGAATGCC-TTTT-----TGGCTAGTCACCTAT-CTTCTGACT-ATCGTCTT--GCTTTTTTCAGC-TTGCTGATGATTGTGAGATTTGGTGGTTTAG-AT-GTTAGAGTGTACATAAA-TTCACTAAGGATGTT--------------------------------------------------------------------

>MH333280_Acaulospora_tsugae

------------------------------------------------------------------------------------------------------------------------------------------------------------------------------------------------------------------------------AGAATA-CTAGGAA-------------------TTTATTTTCC-------TAATAC------TTTATTC-AAAAAATTTTAACCT----------------AAATTTTCTA----AAAAT-----------GTAAAATTTAAATAAGATAACTTTCAACAACGGATCTCTTGGCTCTTGCATCGATGAAGAACGCAGCGAAATGCGATAAGTAATGTGAATTGCAGAATTCCGTGAATCATTAAATCTTTGAACGCAAATTGCGCTTCTT-GGTATTCCGAGGAGTATGCTTGCTTGAGGGTTGGTCTACAAAATAAATCGT-GAAAAA----------TTTTTTTTTTT-------------CGCGGATCTGGG---TTTTTCCAAA---AG--TCA-----------------------ATTTTTTGGTAACCTT-AAATTTATCTTAACAATCATTAGGT-----ATTGA-TA-TTAG-AGATGT----TTAT-CTTACG----------------------------------CA-TTGATAATTAT-----ACC---AATTGTTACATCGCG--TTATTCATCTAG------------------------------TAT-CCTT--TA----TT-AGG--T-GTAATAA-TTCAA-TCTT-----TTT----AATCCTAC----------------------------------------------------------------------------------------------------------------------------------------------------------------------------------------------------------------------------------------------------------------------------------------------------------------------------------------------------------------------------------------------------------------------------------------------------------------------------------------------------------------------------------------------------------------------------------------------------------------------------------------------------------------------------------------------------------------------------------------------------------------------------------------------------------------------------------------------------------------------------------------------------------------------------------

>MH045498_Acaulospora_tsugae

-------------------------------------------------------------------------------------------------------------------------------------------------------------------------------------------------TCCGTAGGTGAACCTGCGGAAGGATCATTAGAACA-CTAGGAA-------------------TTTATTTTCC-------TAATAC------TTTATTC-AAAAAATTTTAATCT----------------AAATTTTCTA----AAAAC-----------GTAAAATTTAAATAAGATAACTTTCAACAACGGATCTCTTGGCTCTTGCATCGATGAAGAACGCGGCGAAATGCGATAAGTAATGTGAATTGCAGAATTCCGTGAATCATTAAATCTTTGAACGCAAATTGCACTTCTT-GGTATTCCGAGGAGTATGCTTGCTTGAGGGTTGGTCTACAAAATAAATCGT-GAAAAATT-------TTTTTTTTTTTT-------------CGCGGATCTGGG---TTTTTCCAAA---AA--TCA-----------------------ATTTTTTGGTAACCTT-AAATTTATCTTAACAATCATTAGGT-----ATTGA-TA-TTAG-AAATGT----TTAT-CTTACG----------------------------------CA-TTGATAATTAT-----ACC---AATTGTTACATCGCG--TTATTCATCTAG------------------------------TAT-CCTT--TA----TT-AGG--T-GTAATAA-CTCAA-TCTT-----TTT----AATCCTACCTCAAGTC-AAGTAAG-AATACCCGCTGAAC-TT-AAGCATATCAATAAGCGGAGGA-------------------------------------------------------------------------------------------------------------------------------------------------------------------------------------------------------------------------------------------------------------------------------------------------------------------------------------------------------------------------------------------------------------------------------------------------------------------------------------------------------------------------------------------------------------------------------------------------------------------------------------------------------------------------------------------------------------------------------------------------------------------------------------------------------------------------------------------------------------------------

>KP756453_Acaulospora_mellea

CCTAGTAAGCGTGAGTCATCAGCTCATGTTGATTACGTCCCT-GCCCTTTGTACACACCGCCCGTCGCTACTACCGATTGAAT-GGCTTAGTGAGACTCTCGGATCGGGTTTTAGGAACCGGCAACGGTACCTTTCTTCTGAGAAGTTCGTCAAACTTGGTCATTTAGAGGAAGTAAAAGTCGTAACAAGGTTTCCGTAGGTGAACCTGCGGAAGGATCATTAGAATA-TTAGGAAAGATT------------TTTAAATCTTTC-------TAATAC-------TTATTCAAAAAA-TTTCTATCTTAT--------ACT--ATATATATT-----TAAACA-TTTTTA--------AAAAATTTTTGATAACTTTCAACAACGGATCTCTTGGCTCTTGCATCGATGAAGAACGCAGTGAAATGCGATAGGTAATGTGAATTGCAGAATTCCGTGAATCATCAAATCTTTGAACGCAGATTGCACTCTCT-GGTATTCCGGGGAGTATGCTTGCTTGAGGGTTAGTTTA---TACAAATCGT-AAAAAGAA--------------ATTTT-------------TACGGATCTGGA---TTTTCCAAAG---TT-----------------------------CTTTTTGGTAATCTA-AAATTTATCTTAACAATA------------GGTGA-TATTTGG-AAATGT-T--TATC-TTT--------------------------------AATT-CA-TTAACAATTATT----ACT-TAATTTGTTATGTCGTG--TTATTTTCCTAA-------------------------------TT-TCTT--AA----TT-AGG--T-AAATTAA-TACAA-TTTTTTT--ATTGA--AATCTGACCTCAAGTC-AAGTAAG-AATACCCGCTGAAC-TT-AAGCATATCAATAAGCGGAGGAAAAGAAACTAAC-AAGGATTCCC-TTAG-TAAC-GGCGAGTGAACTGGGAAGAGCTCAAA-TTTTAAATCTCCTAGG-TTA--CCTAGGCGAGTTGTAATTTGAAGAAAGT-G-TTTTGAT-GCTCTGGTTTTATC-TAAATCCTTTGGGA--TAAGGTATCAT-AG-AGGGTGAGAATCCCGTTCATGATTAAACCCTG---GGT--GTCACCTAATTCACTTTCTAAGAGTCGAGTTG-TTTGGGATTGCAGCTCAAAATGGGTGGTAAATTTCACCTAAGGCTAAATATATGCAAGAGACTGATAGCGAACAAGTACCGT-GAGGGAAAGA--TGAAAAGAACTTTGAAAAGAGAGTTAAATAGTACGTGAAATTGTTGAAAGGGAAACGATTGAAGTCAGTCATGCTTGCAAGGAATCAGCATGGTGGTT----------------------------------------------------------TTCTTCCTTGTGCACTTCCTT--GCTTAGCAGGTTAGCATCAA-TTTTGATGGTCATTAAAGAATT-GGGGGAATGTGACTTC---TTC----GGGAGTG-TTATAGACCCTGAT-AGATGTGGCGATCGGGATTGAGGATTGCAGCGAATGCT-TCTT------GGCTAGTCACCTGG-CTTCTGATA-GTTACCTT--GCTTCCGACAGC-TTGCTAACGGTGGTGAGACTCGATTATCTAG-AT-GTCAGAGTGAACATAAA-TTCGCTAAGGATGCTGACGTAATGGCTTTAAACGACCCGTCTTG-AACACGGACCAAGGAG----------------------

>KP756456_Acaulospora_mellea

CCTAGTAAGCGTGAGTCATCAGCTCATGTTGATTACGTCCCT-GCCCTTTGTACACACCGCCCGTCGCTACTACCGATTGAAT-GGCTTAGTGAGACTCTCGGATCGGGTTTTAGGAACCGGCAACGGTACCTTTCTTCTGAGAAGTTTGTCAAACTTGGTCATTTAGAGGAAGTAAAAGTCGTAACAAGGTTTCCGTAGGTGAACCTGCGGAAGGATCATTAGAATA-TTAGGAAAGATT------------TTTAAATCTTTC-------CAATAC-------TTATTC-AAAAA-TTTCTATCTTAT--------ACT--ATATATATT-----TAAACA-TTTTTA---------AAAAATTTTGATAACTTTCAACAACGGATCTCTTGGCTCTTGCATCGATGAAGAACGCAGTGAAATGCGATAGGTAATGTGAATTGCAGAATTCCGTGAATCATCAAATCTTTGAACGCAGATTGCACTCTCT-GGTATTCCGGGGAGTATGCTTGCTTGAGGGTTAGTTTA---TACAAATCGT-AAAAAGAA--------------ATTTT-------------TACGGATCTGGA---TTTTCCAAAG---TT----------------------------CTTTTTTGGTAATCTA-AAATTTATCTTAACAATA------------GGTGA-TATTTGG-AAATGT-T--TATA-TTT--------------------------------AATT-CA-TTAACAACTATT----ACC-AAATTTGTTATGCCGTG--TTATTTTTCTAA-------------------------------TT-TCTT--AA----TT-AGG--T-AAAGTAA-TACAA-TTTTTC---ATTGA--AATCTGACCTCAAGTC-AAGTAAG-AATACCCGCTGAAC-TT-AAGCATATCAATAAGCGGAGGAAAAGAAACTAAC-AAGGATTCCC-TTAG-TAAC-GGCGAGTGAACTGGGAAGAGCTCAAA-TTTTAAATCTCCTAGG-TTA--CCTAGGCGAGTTGTAATTTGAAGAAAGT-G-TTTTGAT-ACTCTGGTTTTGTC-TAAATCCTTTGGGA--TAAGGTATCAT-AG-AGGGTGAGAATCCCGTTCATGATTAAACCCTG---GGT--GTCACCTAATTCACTTTCTAAGAGTCGAGTTG-TTTGGGATTGCAGCTCAAAATGGGTGGTAAATTTCACCTAAGGCTAAATATATGCAAGAGACCGATAGCGAACAAGTACCGT-GAGGGAAAGA--TGAAAAGAACTTTGAAAAGAGAGTTAAATAGTACGTGAAATTGTTGAAAGGGAAACGATTGAAGTCAGTCATGCTTGCAGGGAATCAGCATGGTGGTT----------------------------------------------------------TTCTTCCTTGTGCACTTCCTT--GCTTGGTAGGTTAGCATCAA-TTTTAATTGTCATTAAAGAATC-GGGGAAATGTGACTTC---TTC----GGGAGTG-TTATAGACCTCGGT-AGATGTGGCGATTGGGATTGAGGATTGCAGCGAATGCT-TCTT------GGCTAGTCACCTGA-TTCCTTACG-GTCACCTT--GCTTTCGACAGC-TTGCTAACGTTAGTGAGATTTGATTGTTTAG-GT-GTTAGACTGATCATAAA-TTCGCTAAGGATGCTGACGTAATGGCTTTAAACGACCCGTCTTG-AACACGGACCAAGGAG----------------------

>KY565427_Acaulospora_koreana

------------------TCAGCTC-CGTTGATTACGTCCCT-GCCCTTTGTACACACCGCCCGTCGCTACTACCGATTGAAT-GGCTTAGTGAGACTCTCGGATCGGGTTTTAGGAACCGGCAACGGTACCTTTCTTCTGAGAAGTTTGTCAAACTTGGTCATTTAGAGGAAGTAAAAGTCGTAACAAGGTTTCCGTAGGTGAACCTGCGGAAGGATCATTAGAATA-TTAGGAAGATTT------------TTTAAATCTTTC-------TAACAC-------TTATTC-AAAAA-TTTCGATCTTAT----ATATACT--ATATATTT------TAAACA-TTTTAA-------AAAAAATTTCTGATAACTTTCAACAACGGATCTCTTGGCTCTTGCATCGATGAAGAACGCAGCGAAATGCGATAGGTAATGTGAATTGCAGAATTCCGTGAATCATCAAATCTTTGAACGCAAATTGCACTCTCT-GGTATTCCGGGGAGTATGCTTGCTTGAGGGTTAGTGGT---TACAAATCGT-AAAAAGAA--------------ATTTT-------------TACGGATCTGGA---TTTTCCAAAG---TT---------------------------CTTTTTTTGGTAATCTA-AAATTTATCTTGACAATA------------GGTGA-TA-TTGG-AAATGT-T--TATC-TTT--------------------------------AATT-CA-TTAACAATTATT----ATC--AATTTGTTATGTCGTG--TTATTTTTCTAA-------------------------------TT-TCTT--AA----TT-AGG--T-AAAGTAA-TACAC-TTTTTC---TTTGA--AATCTGACCTCAAGTC-AAGTAAG-AATACCCGCTGAAC-TT-AAGCATATCAATAAGCGGAGGAAAAGAAACTAAC-AAGGATTCCC-TTAG-TAAC-GGCGAGTGAACTGGGAAGAGCTCAAA-TTTTAAATCTCCTAGG-TTA--CCTAGGCGAGTTGTAATTTGAAGAAAGT-G-TTTTGAT-GCTCTGGTTTTATC-TAAATCCTTTGGGA--TAAGGTATCAT-AG-AGGGTGAGAATCCCGTTCATGATTAAA-CCTG---GGT--GTCACCTAATTCACTTTCTAAGAGTCGAGTTG-TTTGGGCTTGCAGTTCAAAATGGGTGGTAAATTTCACCTAAGGCTAAATATGTGCAAGAGACCGATAGCGAACAAGTACCGT-GAGGGAAAGA--TGAAAAGAACTTTGAAAAGAGAGTTAAATAGTACGTGAAATTGTTGAAAGGGAAACGATTGAAGTCAGTCATGCTTGCAGGGAATCAGCATGGTGGTT----------------------------------------------------------TTCTTCCTTGTGCACTTCCTT--GCTTGGCAGGTTAGCATCAA-TTTTGATTGTCATTAAAGAACC-GGGGGAATGTGGCTTC---TTC----GGGAGTG-TTATAGACCTTGGT-AGATGTGGCGATTGGGATTGAGGATTGCAGCGAATGCT-TCTT------GGCTAGTCACCTGG-CTTCTGATG-GTCACCTT--GCTTTCGATAGC-TTGCTAACGTTGGTGAGATTTGATTGTCTAG-AT-GTTAGTGTGATCATAAA-TTCGCTAAGGATGCTGACGTAATGGC-TTAAACGACCCGTC------------------------------------------

>KY565429_Acaulospora_koreana

------------------TCAGCTC-CGTTGATTACGTCCCT-GCCCTTTGTACACACCGCCCGTCGCTACTACCGATTGAAT-GGCTTAGTGAGACTCTCGGATCGGGTTTTAGGAACCGGCAACGGTACCTTTCTTCTGAGAAGTTTGTCAAACTTGGTCATTTAGAGGAAGTAAAAGTCGTAACAAGGTTTCCGTAGGTGAACCTGCGGAAGGATCATT-----------GAAGATTT------------TTTAAATCTTTC-------TAACAC-------TTATTC-AAAAA-TTTCGATCTTAT----ATATACT--ATATATTT------TAAACA-TTTTAA-------A-----------ATAACTTTCAACAACGGATCTCTTGGCTCTTGCATCGATGAAGAACGCAGCGAAATGCGATAGGTAATGTGAATTGCAGAATTCCGTGAATCATCAAATCTTTGAACGCAAATTGCACTCTCT-GGTATTCCGGGGAGTATGCTTGCTTGAGGGTTAGTG-------------GT-AAAAAGAA--------------ATTTT-------------TACGGATCTGGA---TTTTCCATAG---TT---------------------------CTTTTTTTGGTAATCTA-AAATTTATCTTGACAATA------------GGTGA-TA-TTGG-AAATGT-T--TATC-TTT--------------------------------AATT-CA-TTAACAATTATT----ATC--AATTTGTTATGTCGTG--TTATTTTTCTAA-------------------------------TT-TCTT--AA----TT-AGG--T-AAAGTAA-TACAC-TTTTTC---------------GACCTCAAGTC-AAGTAAG-AATACCCGCTGAAC-TT-AAGCATATCAATAAGCGGAGGAAAAGAAACTAAC-AAGGATTCCC-TTAG-TAAC-GGCGAGTGAACTGGGAAGAGCTCAAA-TTTTAAATCTCCTAGG-TTA--CCTAGGCGAGTTGTAATTTGAAGAAAGT-G-TTTTGAT-GCTCTGGTTTTATC-TAAATCCTTTGGGA--TAAGGTATCAT-AG-AGGGTGAGAATCCCGTTCATGATTAAA-CCTG---GGT--GTCACCTAATTCACTTTCTAAGAGTCGAGTTG-TTTGGGATTGCAGCTCAAAATGGGTGGTAAATTTCACCTAAGGCTAAATATGTGCAAGAGACCGATAGCGAACAAGTACCGT-GAGGGAAAGA--TGAAAAGAACTTTGAAAAGAGAGTTAAATAGTACGTGAAATTGTTGAAAGGGAAACGATTGAAGTCAGTCATGCTTGCAGGGAATCAGCATGGTGGTT----------------------------------------------------------TTCTTCCTTGTGCACTTCCTT--GCTTGGCAGGTTAGCATCAA-TTTTGATTGTCATTAAAGAACC-GGGGGAATGTGGCTTC---TTC----GGGAGTG-TTATAGACCTTGGT-AGATGTGGCGATTGGGATTGAGGATTGCAGCGAATGCT-TCTT------GGCTAGTCACCTGG-CTTCTGATG-GTCACCTT--GCTTTCGATAGC-TTGCTAACGTTAGTGAGATTTGATTGTCTAG-AT-GTTAGTGTGATCATAAA-TTCGCTAAGGATGCTGACGTAATGGC-TTAAACGACCCGTC------------------------------------------

>KP756584_Acaulospora_lacunosa

CCTAGTAAGCGTGAGTCATCAGCTCACGTTGATTACGTCCCT-GCCCTTTGTACACACCGCCCGTCGCTACTACCGATTGAAT-GGCTTAGTGAGACTCTCGGATCGGGTTTTAGGAACCGGCAACGGTACCTTTCTTCTGAGAAGTTTGTCAAACTTGGTCATTTAGAGGAAGTAAAAGTCGTAACAAGGTTTCCGTAGGTGAACCTGCGGAAGGATCATTAGAATA-TTAGGAAGATTT------------TTT--ATCTTTC-------CAATAC------TTTACTC-AAAAT-TTTCGGTCTTAT----AT-------ATATATACT-----TTAACC-ATTTTT----TTAAAAAAAAATTTGATAACTTTCAACAACGGATCTCTTGGCTCTTGCATCGATGAAGAACGCAGCGAAATGCGATAGGTAATGTGAATTGCAGAATTCCGTGAATCATCAAATCTTTGAACGCAAATTGCACTTCCTGGGTATTCCGGGGAGTATGCTTGCTTGAGGGCTATTCAGA--TACAAATCGT-GAAAAGAA--------------ATTTT-------------TACGGATCTGGA---TTTTCCAAAA---AT--TCA----------------------TTTTTTTTGGTAATCTA-AAATTTATCCTAACAATA------------GGTGA-TA-TTGG-AAATGT-T--TATC-TTTG-------------------------------AATT-CA-TCAACAATTATT----ACC--AATTTGTTACGTCGTG--TTATTTTCCTAA-------------------------------TT-TCTT--GA----TT-AGG--TAAAAGCAA-CACAA-TTTTTCC--TTTGA--AATCTGACCTCAAGTC-AAGTAAG-AATACCCGCTGAAC-TT-AAGCATATCAATAAGCGGAGGAAAAGAAACTAAC-AAGGATTCCC-TTAG-TAAC-GGCGAGTGAACCGGGAAGAGCTCAAA-TTTTAAATCACCTAGG-TTA--CCTAGGCGAGTTGTAATTTGAAGAAAGTGG-TTTTGAC-GCTCTGGTTTGGTC-CAAATCCTTTGGGA--TAGGGTATCAT-AG-AGGGTGAGAATCCCGTTCATGATTAAACCCTG---GGT--GTCACTTAACCCACTTTCTAAGAGTCGAGTTG-TTTGGGATTGCAGCTCAAAATGGGTGGTAAATTTCACCTAAGGCTAAATATGTGCAAGAGACCGATAGCGAACAAGTACCGT-GAGGGAAAGA--TGAAAAGAACTTTGAAAAGAGAGTTAAATAGTACGTGAAATTGTTGAAAGGGAAACGATTGAAGTCAGTCATGCTTGCAGGGAATCAGCATGGTGGTT--------------------------------------------------------TATTCTTCCTTGTGCACTTCCTT--GCTTGGCAGGTTAGCATCAA-TTTTGATTGTCATTAAAGAACT-GGGGGAATGTGACTTC---TTC----GGGAGTG-TTATAGACCCTGGT-AGATGTGGCGATCGGGATTGAGGATTGCAGCGAATGCT-TCTT------GGCTAGTCACCTGG-CTTCTGATG-GTTGCCTC--GCTTCCGATAGC-TTGCTAATGGTGGTGAGACTCGATTGTCTAG-AT-GTTGGAGTGATCATAAA-TTCGCTAAGGATGCTGACGTAATGGCTTTAAACGACCCGTCTTG-AACACGGACCAAGGAG----------------------

>Acaulospora_lacunosa_KP756434

CCTAGTAAGCGTGAGTCATCAGCTCACGTTGATTACGTCCCT-GCCCTTTGTACACACCGCCCGTCGCTACTACCGATTGAAT-GGCTTAGTGAGACTCTCGGATCGGGTTTTAGGAACCGGCAACGGTACCTTTCTTCTGAGAAGTTTGTCAAACTTGGTCATTTAGAGGAAGTAAAAGTCGTAACAAGGTTTCCGTAGGTGAACCTGCGGAAGGATCATTAGAATA-TTAGGAAGA-TT------------TCTAAATCTTTC-------CAATAC-------TTATTC-AAAAA-TTTCGATCTTAT----ATATACT--ATATATATT-----TAAACA-TTTTTT--------AAAAAAATTTGATAACTTTCAACAACGGATCTCTTGGCTCTTGCATCGATGAAGGACGCAGCGAAATGCGATAGGTAATGTGAATTGCAGAATTCCGTGAATCATCAAATCTTTGAACGCAAATTGCACTTCCTGGGTATTCCGGGGAGTATGCTTGCTTGAGGGCTATTCAT---GTATAATCGT-GAAAAGAA--------------ATTTT-------------TACGGATCTGGA---TTTTCCAAAA---GT--TCG-----------------------TTTTTTTGGTAATCTA-AAATTTATCCTAACAATA------------GGTGA-TA-TTGG-AAATGT-T--TATC-TTTG-------------------------------AATT-CA-TCAACAATTATT----ACC--AATTTGTTACGTCGTG--TTATTTTCCTAA-------------------------------TT-TCTT--GA----TT-AGG--TAAAAGCAA-CACAA-TTTTTCCT-TTTGA--AATATGACCTCAAGTC-AAGTAAG-AATACCCGCTGAAC-TT-AAGCATATCAATAAGCGGAGGAAAAGAAACTAAC-AAGGATTCCC-TTAG-TAAC-GGCGAGTGAACTGGGAAGAGCTCAAA-TTTTAAATCACCTAGG-TTA--CCTAGGCGAGTTGTAATTTGAAGAAAGT-G-TTTTGAT-GCTCTGGTTTGGTC-CAAATCCTTTGGGA--TAGGGTATCAT-AG-AGGGTGAGAATCCCGTTCATGATTAAATCCTG---GGT--GTCACTTAATTCACTTTCTAAGAGTCGAGTTG-TTTGGGATTGCAGCTCAAAATGGGTGGTAAATTTCACCTAAGGCTAAATATGTGCAAGAGACCGATAGCGAACAAGTACCGT-GAGGGAAAGA--TGAAAAGAACTTTGAAAAGAGAGTTAAATAGCACGTGAAATTGTTGAAAGGGAAACGATTGAAGTCAGTCATGCTTGCAGGGAATCAACATGGTGGTT--------------------------------------------------------TATTCTTCCTTGTGCACTTCCTT--GCTTGGCAGGTTAGCATCAA-TTTTGATTGTCATTAAAGAATT-GGGGGAATGTGGCTTC---TTC----GGGAGTG-TTATAGACCCTGGT-AGATGTGGCGATCGGGATTGAGGATTGCAGCGAATGCT-TCTT------GGCTAGTCACCTGG-CTTCTGATA-GTTGCCTT--GCCTCCGATAGC-TTGCTAATGGTGGTGAGACTCGATTGTCTAG-AT-GTTAGAGTGAACATAAA-TTCGCTAAGGATGCTGACGTAATGGCTTTAAACGACCCGTCTTG-AACACGGACCAAGGAG----------------------

>LN736022_Acaulospora_foveata

CCTAGTAAGCGTGAGTCATCAGCTCATGTTGATTACGTCCCTCGCCCTTTGTACATACCGCCCGTCGCTACTACTGATTGAATAGGCTTAGTGAGACTCTCGGATCGGGTTTTAGGAACCGGCAACGGTACCTTTCTTCTGAGAAGTTTGTCAAACTTGGTCATTTAGAGGAAGTAAAAGTCGTAACAAGGTTTCCGTAGGTGAACCTGCGGAAGGATCATTAGAATT-ATTAGAAAGATT------------TTTTAATCTTTC-------CAATAC------TTTACTC-AAAAT-TTTCGGTCTTAT----A--------ATACATATT-----CAAATA-TTTTTA-------AAAA--AATTTGATAACTTTCAACAACGGATCTCTTGGCTCTTGCATCGATGAAGAACGCAGCTAAATGCGATAGGTAATGTGAATTGCAGAATTCCGTGAATCATCAAATCTTTGAACGCAAATTGCACTCTCT-GGTATTCCGGGGAGTATACTTGCTTGAGGGTTAGTGAT---TACAAATCGT-GAAAAGAA--------------ATTTT-------------TACGGATCTGGA---TTTTCCAAAA--------------------------------GTTTTTTTGGTAATCTA-AAATTTATCTTAACAATAGCGATAG-----GTGAT-TA-TTGG-AAATGT-T--TATC-TTT--------------------------------AATT-CA-TTAACAATTATT----GCC--AATTTGTTACGTCGTG--TTATTTTCCTAA-------------------------------TC-TCTT--GA----TT-GGG--TAAGAGCAA-CACAA-TTTTTCC--TTTGA--AATCTGACCTCAAGTC-AAGTAAG-AATACCCGCTGAAC-TT-AAGCATATCAATAAGCGGAGGAAAAGAAACTAAC-AAGGATTCCC-TTAG-TAAC-GGCGAGTGAACTGGGAAGAGCTCAAA-TTTTAAATCG------------CCCAGGCGAGTTGTAATTTGAAGAAAGT-G-TTTTGAT-GCTCTGGTTTAGCC-TAAATCCTTTGGGA--TAAGGTATCAT-AG-AGGGTGAGAATCCCGTTCATGACTAAACCGTG---GGT--GTCACCTAATTCACTTTCCAAGAGTCGAGTTG-TTTGGGATTGCAGCTCAAAATGGGTGGTAAATTTCACCTAAGGCTAAATATGTGCAAGAGACCGATAGCGAACAAGTACCGT-GAGGGAAAGA--TGAAAAGAACTTTGAAAAGAGAGTTAAATAGTACGTGAAATTGTTGAAAGGGAAACGATTGAAGTCAGTCATGCTTGCAGGGAATCAACAAAGTGGTT----------------------------------------------------------TTCTTCCTTGTGCACTTCCTT--GCTTGGCAGGTTAGCATCAA-TTTTGATTGTCATTAAAGAACT-GGGGGAATGTGGCTTC---TTC----GGGAGTG-TTATAGACCCTGGT-AGATGTGGCGATTGGGATTGAGGATTGCAGCGAATGCT-TCTT------GGCTAGTCACCTGG-CTTCTGACG-GTTACCTT--GCTTCCGATAGC-TCGCTAACGGTTGTGAGACTCGATTGTCTAG-AT-GTTAGAGTGAACATAAA-TTCGCTAAGGATGCTGACGTAATGGCTTTAAACGACCCGTCTTGAAACACGGACCAAGGAGTCTAAC-ATGTGTGCGAGTGTT

>LN736025_Acaulospora_foveata

CCTAGTAAGCGTGAGTCATCAGCTCATGTTGATTACGTCCCT-GCCCTTTGTACACACCGCCCGTCGCTACTACCGATTGAAT-GGCTTAGTGAGACTCTCGGATCGGGTTTTAGGAACCGGCAACGGTACCTTTCTTCTGAGAAGTTTGTCAAACTTGGTCATTTAGAGGAAGTAAAAGTCGTAACAAGGTTTCCGTAGGTGAACCTGCGGAAGGATCATTAGAATT-ATTAGGAAGATT------------TTTTAATCTTTC-------CAATAC------TTTACTC-AAAAT-TTTCGGTCTTAT----AT-------ACATATATT-----TAAATA-TATTTA-------AAAATTTTTTTGATAACTTTCAACAACGGATCTCTTGGTTCTTGCATCGATGAAGAACGCAGCTAAATGCGATAGGTAATGTGAATTGCAGAATTCCGTGAATCATCAAATTTTTGAACGCAAATTGCACTCTCT-GGTATTCCGGGGAGTATACTTGCTTGAGGGTTAGTGAT---TACAAATCGT-GAAAAGAA--------------ATTTT-------------TACGGATCTGGA---TTTTCCGAAA---AA---------------------------GTTTTTTTGGTAATCCA-AAATTTATCTTAACTATA------------GGTGA-TA-TTGG-AAATGT-T--TATC-TTTTTTT----------------------------TTTT-CA-TGGACAATTATT----ACC--TATTTGTTACGTCGTG--TTATTTTCCTAA-------------------------------TT-CCTT--GA----TT-GGG--TAAGAGTAA-CACAA-TTTTTCC--TTTGA--AATCTGACCTCAAGTC-AAGTAAG-AATACCCGCTGAAC-TT-AAGCATATCAATAAGCGGAGGAAAAGAAACTAAC-AAGGATTCCC-TTAG-TAAC-GGCGAGTGAACTGGGAAAAGCTCAAA-TTTTAAATCA------------CCTAGGTGAGTTGTAATTTGAAGAAAGT-G-TTTTGAT-GCTCTGGTTTAGCC-TAAATCCTTTGGGA--TAAGGTATCAT-AG-AGGGTGAGAATCCCGTTCATGACTAAACCGTG---GGT--GTCACCTAATTCACTTTCCAAGAGTCGAGTTG-TTTGGGATTGCAGCTCAAAATGGGTGGTAAATTTCACCTAAGGCTAAGTATGTGCAAGAGACCGATAGCGAACAAGTACCGT-GAGGGAAAGA--TGAAAAGAACTTTGAAAAGAGAGTTAAATAGTACGTGAAATTGTTGAAAGGGAAACGATTGAAGTCAGTCATGCTTGCAAGGAATCAACAAAGTGGTT----------------------------------------------------------TTCTTCCTTGTGTACTTCCTT--GCTTGGCAGGTTAGCATCAA-TTTTGATTGTCATTAAAGAACT-GGGGGAATGTGGCTTC---TTC----GGGAGTG-TTATAGACCCTGGT-AGATGTGGCGATTGGGATCGAGGATTGCAGCGAATGCT-TCTT------GGCTAGTCACCTGG-CTTCTGATG-GTCACCTT--GCTTTCGATAGC-TTGCTAACGTTGGTGAGATTTGATTGTCTAG-AT-GTTAGAGTGAACATAAA-TTCGCTAAGGATGCGGACGTAATGGCTTTAAACGACCCGTCTTGAAACACGGACCAAGGAGTCTAAC-ATATGTGCGAGTGTT

>AF133764_Acaulospora_colossica

------------------------------------------------------------------------------------------------------------------------------------------------------------------------------------------------------TAGGGAACCTGCGGAAGGATCATTAGAATTCCCGTGGAAGATT---------------CTTTCTTCC-------CGGCATCTTCTTATTATTC-AAAAATTTTCTATCTT------------T--TTAATCTTT-----TAAAAA-AAAACA--AAATTTAAATTATTAAGACAACTTTCAACAACGGATCTCTTGGCTCTTGCATCGATGAAGAACGCAGCGAAATGCGATAAGTAATGTGAATTGCAGAATCCCGTGAATCATCAAATCTTTGAACGCAAATTGCACTCTTT-GGTATTCCGAAGAGTATGCTTGCTTGAGGGTTGGATCA---CAAATATCGT-GAATGAAA--------------TTTTT-------------TACGGATCTGGG---TTTTCCGAAA---TT--TAA---------------------------TTTGGTAACCTA-AAATTTATCTCAACAATATTGAGG------TATGA-TA-TTGG-AAATGT-T--CTAT-CTTGCATA--------------TTTTTTGTAA----GTTCCG-TTGACAATGATT----ACC---GATTGTTACGTCGTG--TCATTCAT--------------------------------------CTTT--AT----TT-AGG--C-GAAATGA-TTCAA-TCT------TTTGA--ATTTCAACCTCAAGTC-AAGTGAG-AATACCCGCTGAAC-TT-AAGCATATCAATA----------------------------------------------------------------------------------------------------------------------------------------------------------------------------------------------------------------------------------------------------------------------------------------------------------------------------------------------------------------------------------------------------------------------------------------------------------------------------------------------------------------------------------------------------------------------------------------------------------------------------------------------------------------------------------------------------------------------------------------------------------------------------------------------------------------------------------------------------------------------------------

>AF133768_Acaulospora_colossica

-------------------------------------------------------------------------------------------------------------------------------------------------------------------------------------------------------AGGGAACCTGCGGAAGGATCATTAGAATTCCCGTGGAAGATT---------------CTTTCTTCC-------CGGCATCTTCTTATTATTC-AAAAATTTTCTATCTT------------T--TTAATCTTT-----TAAAAA-AAAACA--AAATTTAAATTATTAAGACAACTTTCAACAACGGATCTCTTGGCTCTTGCATCGATGAAGAACGCAGCGAAATGCGATAAGTAATGTGAATTGCAGAATTCCGTGAATCATCAAATCTTTGAACGCAAATTGCACTCTTT-GGTATTCCGAAGAGTATGCTTGCTTGAGGGTTGGATCA---CAAATATCGT-GAATGAAA--------------TTTTT-------------TACGGATCTGGG---TTTTCCGAAA---TT--TAA---------------------------TTCGGTAACCTA-AAATTTATCTTAACAATATTGAGG------TATGA-TA-TTGA-AAATGT-T--CTAT-CTTGCATA--------------TTTTTTGTAA----GTT-CG-TTGACAATGATT----ACC---GATTGTTACGTCGCG--TCATTCATC-------------------------------------CTTT--AT----TT-AGG--T-GAGATGA-TTCAA-TCT------TTTGA--ATTTCAACCTCAAGTC-AAGTGAG-AATACCCGCTGAAC-TT-AAGCATATCAATAAGCGGAA---------------------------------------------------------------------------------------------------------------------------------------------------------------------------------------------------------------------------------------------------------------------------------------------------------------------------------------------------------------------------------------------------------------------------------------------------------------------------------------------------------------------------------------------------------------------------------------------------------------------------------------------------------------------------------------------------------------------------------------------------------------------------------------------------------------------------------------------------------------------------

>Acaulospora_laevis_FN547511

CCTAGTAAGCGTGAGTCATCAGCTCATGTTGATTACGTCCCT-GCCCTTTGTTCACACCGCCCGTCGCTACTACCGATTGAAT-GGCTTAGTGAGACTCTCGGATCGGGATTTCGGAACCGGCAACGG-CCCCTTTTTCTGAGAAGTTTGTCAAACTTGGTCATTTAGAGGAAGTAAAAGTCGTAACAAGGTTTCCGTAGGTGAACCTGCGGAAGGATCATTAGAATT-CCGTGGATGATT---------------CTTTCTTCC-------CGGCATCTTCTTAATATTC-AAAAATTTTCTACCTT------------T--TTAATCTTT-----AAAAAA-AAC-----ATTTTAAGTTAGTAAAGACAACTTTCAACAACGGATCTCTTGGCTCTTGCATCGATGAAGAACGCAGCGAAATGCGATAATTAATGTGAATTGCAGAATTCCGTGAATCATTAAATCTTTGAACGCAAATTGCACTCTTT-GGTATTCCGAAGAGTATGCTTGCTTGAGGGTTGGATCA---CAAAAATCGT-GAATGAAA-------------TTTTTT-------------TACGGATCTGGG---TTTTCCGAAA---TT--TAA---------------------------TTTGGTAACCTA-AAATTTATCTTAACAATATTGAGG------TATGA-TA-TTGG-AAATGT-T--CTAT-CTTGCATA--------------TTTTTTGTAA----GTTCCG-TTGACAATGATT----ACC--GATTTGTTACGTCGTG--TCATTCATC-------------------------------------TTTT--AT----TT-AGG--T-GATATGA-TTCAA-TCT------TTTGA--ATTTCAACCTCAAGTC-AAGTGAG-AATACCCGCTGAAC-TT-AAGCATATCAATAAGCGGAGGAAAAGAAACTAAC-AAGGATTCCC-TCAG-TAAC-GGCGAGTGAACCGGGAAAAGCTCAAA-TTTAAAATCGCTTGGG--TTTACCTGAGTGAGTTGTAATTTGAAGAGAGT-G-TTTCGACTTTTCGGGTTTGATC-CAAATCCTTTGGGA--TGAGGTATCAT-AG-AGGGTGAGAATCCCGTTCATGATTAAACCTGA---GTT--GTCA-ATGATTCATTTTCTAAGAGTCGAGTTG-TTTGGGATTGCAGCTCAAAATGGGTGGTAAATTTCACCTAAGGCTAAATATCTGCGAGAGACCGATAGCGAACAAGTACCGT-GAGGGAAAGA--TGAAAAGAACTTTGAAAAGAGAGTTAAAAAGTACGTGAAATTGTTGAAAGGGAAACGATTGAAGTCAGTCATGCTAGCGAGGAATCAACTTGGTGGTG-------TGAGTC---------------------------------------TCTCATCTCCTCCGTGTGCACTTCCTC--GTCTGGCAGGTTAGCATCAA-TTTTGATTGTCATCAAATAATT-GGGGTAATGTGGCTTC---GCTTCGGTGGAGTG-TTATAGACCCTGGT-GGATGTGGCGATTGGGATTGAGGATTGCAGCGGATGCC-TCTC------GGCTAGTCATCCGA-TTCCTGGCT-GTCACCTC--GCTTTCGACAGC-TTGCTGACGATCGTGGGACTCGGCGGTCTAG-GT-GTCGGTCTGATCTTAAA-TTCGCTAAGGATGCTGACGTAATGGCTTTAAACGACCCGTCTTGAAACACGGACCAAGGAGTCTAAC-ATGTGTGCGAGTGTT

>Acaulospora_laevis_FN547516

CCTAGTAAGCGTGAGTCATCAGCTCATGTTGATTACGTCCCT-GCCCTTTGTACACACCGCCCGTCGCTACTACCGATTGAAT-GGCTTAGTGAGACTCTCGGATCGGGATTTCGGAACCGGCAACGGTCCCTTTTTTCTGAGAAGTTTGTTAAACTTGGTCATTTAGAGGAAGTAAAAGTCGTAACAAGGTTTCCGTAGGTGAACCTGCGGAAGGATCATTAGAATTCCCGTGGAAGATT---------------CTTTCTTCC-------CGGCATCTTCTTCATATTC-AAAAATTTTCTACCTT------------T--TTAATCTTT-----AAAAAA-AAC--A--AATTTAAATTATTAAAGACAACTTTCAACAACGGATCTCTTGGCTCTTGCATCGATGAAGAACGCAGCGAAATGCGATAAGTAATGTGAATTGCAGAATTCCGTGAATCATCAAATCTTTGAACGCAAATTGCACTCTTT-GGTATTCCGAAGAGTATGCTTGCTTGAGGGTTGGATCA---CAAAAATCGT-GAATGAAA--------------TTTTT-------------TACGGATCTGGG---TTTTCCGAAA---TT--TAA---------------------------CTTGGTAACCTA-AAATTTATCTTAACAATATTGAGG------TATGA-TA-TTGG-AAATGT-T--CTAT-CTTGCATA--------------TTTTTTGTAA----GTTCCG-TTGACAATGATT----ACC--GATTTGTTACGTCGTG--TCATTCATC-------------------------------------TTTT--AT----TT-AGG--T-GATATGA-TTCAA-TCT------TTTGA--ATTTCAACCTCAAGTC-AAGTGAG-AATACCCGCTGAAC-TT-AAGCATATCAATAAGCGGAGGAAAAGAAACTAACAAAGGATTCCC-TCAG-TAAC-GGCGAGTGAACCGGGAAAAGCTCAAA-TTTAAAATCGCTTGGG--TTTACCTGAGTGAGTTGTAATTTGAAGAGAGT-G-TTTCGACTTTTCGGGTTTGATC-CAAATCCTTTGGGA--TGAGGTATCAT-AG-AGGGTGAGAATCCCGTTCATGATTAAACCTGA---GTT--GTCA-ATGATTCATTCTCTAAGAGTCGAGTTG-TTTGGGATTGCAGCTCAAAATGGGTGGTAAATTTCACCTAAGGCTAAATATCTGCGAGAGACCGATAGCGAACAAGTACCGT-GAGGGAAAGA--TGAAAAGAACTTTGAAAAGAGAGTTAAAAAGTGCGTGAATTTGTTGAAAGGGAAACGATTGAAGTCAGTCATGCTAGCGAGGAATCAACTTGGTGGTG-------TGAGTC---------------------------------------TCTTATCTCCTCCGTGTGCACTTCCTC--GTCTGGCAGGTTAGCATTAA--TTTGATTGTTATCAAATAATT-GGGGTAATGTGGCTTC---TCTTCGGTGGAGTG-TTATAGACCCTGGT-GGATGTGGCGATTGGGATTGAGGATTGCAGCGGATGTC-TCTT------GGCTAGTCATCCGA-TTCCTGGCT-GTCACTTC--GCTTTCGACAGC-TTGCTGACGATTGTGGGACTCGGCG----------GTCGGTCTGATCTTAAA-TTCGCTAAGGATGCTGACGTAATGGCTTTAAACGACCCGTCTTGAAACACGGACCAAGGAGTCTAAC-ATGTGTGCGAGTGTT

>FR750173_Acaulospora_entreriana

CCTAGTAAGCGTGAGTCATCAGCTCATGTTGATTACGTCCCT-GCCCTTTGTACACACCGCCCGTCGCTACTACCGATTGAAT-GGCTTAGTGAGACTCTCGGATTGGGATTTCGGGACCGGCAACGGTCCCTTTCTTCTGAGAAGTTTGTCAAACTTGGTCATTTAGAGGAAGTAAAAGTCGTAACAAGGTTTCCGTAGGTGAACCTGCGGAAGGATCATTAGAATT-CCGTGGAAGATTA--------------TTTTCTTCC-------CGGCATCTTCTTAATATTCAAAAAATTTTCTATCTTCC----------T--TTGATCTTT-----GAAAAA-AAATTA--AAAATTAAATATATAAGACAACTTTCAACAACGGATCTCTTGGCTCTTGCATCGATGAAGAACGCAGCGAAATGCGATAAGTAATGTGAATTGCAGAATTCCGTGAATCATCAAATCTTTGAACGCAAATTGCACTCTTT-GGTATTCCGAAGAGTATGCTTGCTTGAGGGTTGGATCA---CAAAAATCGT-GAATGAAA--------------ATTTT-------------TACGGATCTGGG---TTTTCCGAGA---AA--TTG------------------------ATTCTTGGTAACCTA-AAATTTATCCTAACAATATTTAGG------TATGA-TA-TTGG-AAATGT-T--CTAT-CTTGCATT--------------TTTTTTGTAA----GTT-CG-TTGACAATGATT----ACC---GATTGTTACGTCGTG--TCATTCATC---------------------------------TTT-TTTT--TA----TT-AGG--T-GAAATGA-TTCAA-TCT------TTTGA--ATTTCAACCTCAAGTC-AAGTGAG-AATACCCGCTGAAC-TT-AAGCATATCAATAAGCGGAGGAAAAGAAACTAAC-AAGGATTCCC-TTAG-TAAC-GGCGAGTGAACCGGGAAAAGCTCAAA-TTTAAAATCGCTTGGG--TTTACCTGTGCGAGTTGTAATTTGAAGAATGT-G-TTTCGACTTTTTTGGTTTGATC-CAAATCCTTTGGGA--TGAGGTATCAT-AG-AGGGTGAGAATCCCGTTCATGATTAAACCTTA---GTT--GTCAATTGATTCACTTTCTAAGAGTCGAGTTG-TTTGGGATTGCAGCTCAAAATGGGTGGTAAATTTCACCTAAGGCTAAATATTTGCAAGAGACCGATAGCGAACAAGTACCGT-GAGGGAAAGA--TGAAAAGAACTTTGAAAAGAGAGTTAAAAAGTACGTGAAATTGTTGAAAGGGAAACGATTGAAGTCAGTCATGCTAGTGAGGAATCAACATGGTGGTG-------TGAGTC---------------------------------------TCTCATCTCTTCCGTGTGCACTTCCTC--GTCTGGCAGGTTAGCATCAA-TTTCGATTGTCATCAAATAATT-GGGGTAATGTGGCTTC---GCTTCGGTGGAGTG-TTATAGACCTTGGT-GGATGTGGCGATTGGGATTGAGGATTGCAGCGGATGCC-TCTT------GGCTAGTCACCTGA-CTCCTGACT-GTCACCTC--GCTTTCGACAGC-TTGCTGACGATTGTGGGACACGGCGGTCTAG-AA-GTTAGAGTGATCTTAAA-TTCGCTAAGGATGCTGACGTAATGGCTTTAAACGACCCGTCTTGAAACACGGACCAAGGAGTCTAAC-ATGTGTGCGAGTGTT

>FR750169_Acaulospora_entreriana

CCTAGTAAGCGTGAGTCATCAGCTCATGTTGATTACGTCCCT-GCCCTTTGTACACACCGCCCGTCGCTACTACCGATTGAAT-GGCTTAGTGAGACTCTCGGATTGGGATTTCGGGACCGGCAACGGTCCCTTTCTTCTGAGAAGTTTGTCAAACTTGGTCATTTAGAGGAAGTAAAAGTCGTAACAAGGTTTCCGTAGGTGAACCTGCGGAAGGATCATTAGAATT-CCGTGGAAGATTA--------------TTTTCTTCC-------CGGCATCTTCTTAATATTCAAAAAATTTTCTATCTTCC----------T--TTGATCTTT-----GAAAAA-AAATTA--AAAATTAAATATATAAGACAACTTTCAACAACGGATCTCTTGGCTCTTGCATCGATGAAGAACGCAGCGAAATGCGATAAGTAATGTGAATTGCAGAATTCCGTGAATCATCAAATCTTTGAACGCAAATTGCACTCTTT-GGTATTCCGAAGAGTATGCTTGCTTGAGGGTTGGATCA---CAAAAATCGT-GAATGAAA--------------ATTTT-------------TACGGATCTGGG---TTTTCCGAGA---AA--TTG------------------------ATTCTTGGTAACCTA-AAATTTATCCTAACAATATTTAGG------TATGA-TA-TTGG-AAATGT-T--CTAT-CTTGCATA--------------TTTTTTGTAA----GTT-CG-TTGACAATGATT----ACC---GATTGTTACGTCGTG--TCATTCATCTTT------------------------------TTT-TTTT--TG----TT-AGG--T-GAAATGA-TTCAA-TCT------TTTGA--ATTTCAACCTCAAGTC-AAGTGAG-AATACCCGCTGAAC-TT-AAGCATATCAATAAGCGGAGGAAAAGAAACTAAC-AAGGATTCCC-TTAG-TAAC-GGCGAGTGAACCGGGAAAAGCTCAAA-TTTAAAATCGCTTGGG--TTTACCTGTGCGAGTTGTAATTTGAAGAATGT-G-TTTCGACTTTTTTGGTTTGATC-CAAATCCTTTGGGA--TGAGGTATCAT-AG-AGGGTGAGAATCCCGTTCATGATTAAACCTTA---GTT--GTTAATTGATTCACTTTCTAAGAGTCGAGTTG-TTTGGGATTGCAGCTCAAAATGGGTGGTAAATTTCACCTAAGGCTAAATAT-TGCAAGAGACCGATAGCGAACAAGTACCGT-GAGGGAAAGA--TGAAAAGAACTTTGAAAAGAGAGTTAAAAAGTACGTGAAATTGTTGAAAGGGAAACGATTGAAGTCAGTCATGCTAGTAAGGAATCAACATGGTGGTG-------TGAGTC---------------------------------------TCTCATCTCTTCCGTGTGCACTTCCTC--GTCTGGCAGGTTAGCATCAA-TTTCGATTGTCATCAAATAATT-GGGGTAATGTGGCTTC---ACTTCGGTGGAGTG-TTATAGACCCTGGT-GGATGTGGCGATTGGGATTGAGGATTGCAGCGGATGCC-TCTT------GGCTAGTCACCTGA-CTCCTGGCT-GTCACCTC--GCTTTCGACAGC-TTGCTGACGATCGTGGGACACGGCGGTCTAG-GGTGTTGGTCTGATCTTAAA-TTCGCTAAGGATGCTGACGTAATGGCTTTAAACGACCCGTCTTGAAACACGGACCAAGGAGTCTAAC-ATGTGTGCGAGTGTT

>HG421736_Acaulospora_viridis

CCTAGTAAGCGTGAGTCATCAGCTCACGTTGATTACGTCCCT-GCCCTTTCTACACACCGCCCGTCGCTACTACCGATTGAAT-GGCTTAGTGAGACCCTCGGATCGGGATTTGGGGATCGGCAACGTTCCCTCTTTTCTGAGAAGTTTGTCAAACTTGGTCATTTAGAGGAAGTAAAAGTCGTGACAAGGTTTCCGTAGGTGAACCTGCGGAAGGATCATTAGAATA-TTACGGGAATTTAATTAATTAATTTATTTATTTCCC-------TGATGT------CTTATTC-AAAAAATTTTCAGATCTTA--------TC--GTAATCTTT----AAAAAAA-AAA-------AACAATTATATTAAGACAACTTTCAACAACGGATCTCTTGGCTCTTGCATCGATGAAGAACGCAGCGAAATGCGATAAGTAATGTGAATTGCAGAATTCCGTGAATCATCAAATCTTTGAACGCAAATTGCACTCCTT-GGTATTCCGAGGAGTATGCTTGCTTGAGGGTTGTTTCAC--CAAAAATCGT-GAAAAAAAA-------------CATTT-------------CACGGATCTGGG---TTTTTCCAAA---GT--TTT-----------------------GAATTTTGGTGACCTA-AAATTTATCTTAACAATATTTAGG------TATTA-TA-TTGGAAAATGT-T--CTAT-CTTACATG---------------TTTTTGTAA----GTT-CA-TTAATAATTATT----ACC---AATTGTTACGTCGTG--TTATTCATCTAGTG----------------------------TTT-TTTT--AA----TT-AGG--T-GAAATAA-TTCAA-TCTA-----TTTTA--ATTTCAACCTCAAGTC-AAGTAAG-AATACCCGCTGAAC-TT-AAGCATATCAATAAGCGGAGGAAAAGAAACTAAC-AAGGATTCCC-TTAG-TAAC-GGCGAGTGAACCGGGAAGAGCTCAAA-TTTAAAATCGCCTTGGGTTTTACCTGGGCGAGTTGTAATTTGAAGAAAGT-G-TTTGGAC-ATCTGGGTTTGATC-TAAATCCTTTGGGA--TGAGGTATCAT-AG-AGGGTGAAAATCCCGTTCATGACTAAACCTCG---GAT--GTCACTTAATTCACTTTCCAAGAGTCGAGTTG-TTTGGGATTGCAGCTCAAAATGGGTGGTAAATTTCACCTAAGGCTAAATATGTGCAAGAGACCGATAGCGAACAAGTACCGT-GAGGGAAAGA--TGAAAAGAACTTTGAAAAGAGAGTTAAATAGTACGTGAAATTGTTGAAAGGGAAACGATTAAAGTCAGTCATGCTAGTAAGGAATCAACATGGTGGTT---------------------------------------------------------TTTCTGCCGTGTGCACTTTCTT--GCCTTGCAGGTTAGCATCAA-TTTTGGTTGTCATCAAAGAACC-GGGGAAATGTGTCTCT---TCTTCGGAGGAGTGTTTATAGACCTTGGT-AGATGTGGCGACTGGGATTGAGGATTGCAGTGAATGCC-TTTT------GGCTAGTCACCTGG-CTTCTAACG-GTCACCCC--GCTTTCGATAGC-TTGCTGACGATCGTGGGACTCGACAGTCTAG-AT-GTTAGAGTGATCTTAAA-TTCACTAAGGATGCTGACGTAATGGCTTTAAACGACCCGTCTTGAAACACGGACCAAGGAGTCTAAC-ATATGTGCGAGTGTT

>HG421738_Acaulospora_viridis

CCTAGTAAGCGTGAGTCATCAGCTCACGTTGATTACGTCCCT-GCCCTTTGTACACACCGCCCGTCGCTACTACCGATTGAAT-GGCTTAGTGAGACCTTCGGATCGGGATTT-GGGATCGGCAACGTTCCCTCTTTTCTGAGAAGTTTGTCAAACTTGGTCATTTAGGGGAAGTAAAAGTCGTAACAAGGTTTCCGTAGGTGAACCTGCGGAAGGATCATTAGAATA-TTACGGGAATTTAATTAATTTATTTATTTATTTCCC-------TGATGT------CTTATTC-AAAAAATTTTCAGATCTTA--------TC--TTAATCTTT-----AAAAAA-AAA-------AACAATTATATTAAGACAACTTTTAACAACGGATCTCTTGGCTCTTGCATCGATGAAGAACGCAGCGAAATGCGATAAGTAATGTGAATTGCAGAATTCCGTGAATCATCAAATCTTTGAACGCAAATTGCACTCCTT-GGTATTCCGAGGAGTATGCTTGCTTGAGGGTTGTTTCAC--CAAAAATCGT-GAAAAAAAA-------------CATTT-------------CACGGATCTGGG---TTTTTCCAAA---GT--TTT-----------------------GAATTTTGGTGACCTA-AAATTTATCATAACAATATTTAGG------TATTA-TA-TTGG-AAATGT-T--CTAT-CTTACATG---------------TTTTTGTAA----GTT-CA-TTAATAATTATT----ACC---AATTGTTACGTCGTG--TTATTCATCTAGTG-----------------------------TT-TTTT--AA----TT-AGG--T-GAAATAA-TTCAA-TCTA-----TTTTA--ATTTCAACCTCAAGTC-AAGTAAG-AATACCCGCTGAAC-TT-AAGCATATCAATAAGCGGAGGAAAAGAAACTAAC-AAGGATTCCC-TTAG-TAAC-GGCGAGTGAACCGGGAAGAGCTCAAA-TTTAAAATCGCCTTGGGTTTTACCTGGGCGAGTTGTAATTTGAAGAAAGT-G-TTTGGAC-ATCTGGGTTTGATC-TAAATCCTTTGGGA--TGGGGTATCAT-AG-AGGGTGAGAATCCCGTTCATGACTAAACCTCG---GAT--GTCACTTAATTCACTTTCCAAGAGTCGAGTTG-TTTGGGATTGCAGCTCAAAATGGGTGGTAAATTTCACCTAAGGCTAAATATGTGCAAGAGACCGATAGCGAACAAGTACCGT-GAGGGAAAGA--TGAAAAGAACTTTGAAAAGAGAGTTAAATAATACGTGAAATTGTTGAAAGGGAAACGATTAAAGTCAGTCATGCTAGTAAGGAATCAACATGGTGGTT--------------------------------------------------------TTTTCTGCCGTGTGCACTTTCTT--GCCTTGCAGGTTAGCATCAA-TTTTGGTTGTCATCAAAGAACT-AGGGAAATGTGTCTTC---TCTTCGGAGAAGTGTTTATAGACCTTGGT-AGATGTGGCGACTGGGATTGAGGATTGCAGTGAATGCC-TTTT------GGCTAGTCACCTGG-CTTCTGACG-GTCACCCC--GCTTTCGATAGC-TTGCTGACGATCGTGGGACTCGTCAGTCTAG-AT-GTTAGAGTGATCTTAAA-TTCACTAAGGATGCTGACGTAATGGCTTTAAACGACCCGTCTTGAAACACGGACCAAGGAGTCTAAC-ATGTGTGCGAGTGTT

>Acaulospora_intravesiculata_OL661628

-------------------------------ATTACGTCCCT-GCCCTTTGTACACACCGCCCGTCGCTACTACCGATTGAAT-GGCTTAGTGAGACTCTCGGATCGGGTTTTAGGAACCGGCAACGGATCCTTTCTTCTGAGAAGTTTGTCAAACTTGGTCATTTAGAGGAAGTAAAAGTCGTAACAAGGTTTCCGTAGGTGAACCTGCGGAAGGATCATTAGAAT--------------------------------TTTTAT-------ATATAT------CTTATTC-AAAAA-TTTCCATCCTATT---AAAATAA--AAATTTTTT-----TAAAT----------TTTATTAAAAAATTAAGACAACTTTCAACAACGGGTCTCTTGGCTCTTGCATCGATGAAGAACGCAGTGAAATGCGATAGGTAATGTGAATTGCAGAATTCCGTGAATCATCAAATCTTTGAACGCAAATTGCACTCCTT-GGTATTCCGAGGAGTATGCTTGCTTGAGGGTCGGATTAT---------------------A------TTATCGTTTTTT-------------AACGGATCTGGG---ATTTTCCAAG-TTTT--TTT-----------------------AAATCTTGGTAACCTA-AAATTTATCTTAATAATATT----------TGTAA-TA-TTGA-AAATGT----GTAT-CTTACG-----------------TTCACGTGA----GTT-CA-TTGACAATTATT----ATG--AATTTATTACGTCGTA--TTATTCACTCGG------------------------------TTT-TTTTT-AA-CGATC-GGG--TCGAAATGA-TACAA-TTT------TTTGA--AATTCGACCTCAAGTC-AAGTAAG-AATACCCGCTGAAC-TT-AAGCATATCAATAAGCGGAGGAAAAGAAACTAAC-AAGGATTCCC-TTAG-TAAC-GGCGAGTGAACTGGGAAGAGCTCAAA-TTTAAAATCATTAGGG-TTT--CCCTGATGAGTTGTAATTTGAAGAAAGT-G-TTTTGAT-ATTCGGGTTTTATC-TAAATCCTTTGGGA--TGAGGTATCAT-AG-AGGGTGAGAATCCCGTTCGTGATTAAACCTCG---GAT--GTCACTTAATTCATTTTCTAAGAGTCGAGTTG-TTTGGGATTGCAGCTCAAAATGGGTGGTAAATTTCACCTAAGGCTAAATATATGCGAGAGACCGATAGCGAACAAGTACCGT-GAGGGAAAGA--TGAAAAGAACTTTGAAAAGAGAGTTAAACAGTACGTGAAATTGTTGAAAGGGAAACGA---------------------------------------------------------------------------------------------------------------------------------------------------------------------------------------------------------------------------------------------------------------------------------------------------------------------------------------------------------------------------------------------------------------------------------------------------------------

>Acaulospora_intravesiculata_OL661642

-------------------------------ATTACGTCCCT-GCCCTTTGTACACACCGCCCGTCGCTACTACCGATTGAAT-GGCTTAGTGAGACTCTCGGATCGGGTTTTAGGAACCGGCAACGGATCCTTTCTTCTGAGAAGTTTGTCAAACTTGGTCATTTAGAGGAAGTAAAAGTCGTAACAAGGTTTCCGTAGGTGAACCTGCGGAAGGATCATTAGAAT--------------------------------TTTTAT-------ATATAT------CTTATTC-AAAAA-TTTCCATCCTATT---AAAATAA--AAATTTTTT-----TAAAT----------TTTATTAAAAAATTAAGACAACTTTCAACAACGGATCTCTTGGCTCTTGCATCGATGAAGAACGCAGTGAAATGCGATAGGTAATGTGAATTGCAGAATTCCGTGAATCATCAAATCTTTGAACGCAAATTGCACTCCTT-GGTATTCCGAGGAGTATGCTTGCTTGAGGGTCGGATTAT---------------------A------TTATCGTTTTTT-------------AACGGATCTGGG---ATTTTCCAAG-TTTT--TTT-----------------------AAATCTTGGTAACCTA-AAATTTATCTTAATAATATT----------TGTAA-TA-TTGA-AAATGT----GTAT-CTTACG-----------------TTCACGTGA----GTT-CA-TTGACAATTATT----ATG--AATTTATTACGTCGTA--TTATTCACTCGG------------------------------TTT-TTTTT-AA-CGATC-GGG--TCGAAATGA-TACAA-TTT------TTTGA--AATTCGACCTCAAGTC-AAGTAAG-AATACTCGCTGAAC-TT-AAGCATATCAATAAGCGGAGGAAAAGAAACTAAC-AAGGATTCCC-TTAG-TAAC-GGCGAGTGAACTGGGAAGAGCTCAAA-TTTAAAATCATTAGGG-TTT--CCCTGATGAGTTGTAATTTGAAGAAAGT-G-TTTTGAT-ATTCGGGTTTTATC-TAAATCCTTTGGGA--TGAGGTATCAT-AG-AGGGTGAGAATCCCGTTCGTGATTAAACCTCG---GAT--GTCACTTAATTCATTTTCCAAGAGTCGAGTTG-TTTGGGATTGCAGCTCAAAATGGGTGGCAAATTTCACCTAAGGCTAAATATATGCGAGAGACCGATAGCGAACAAGTACCGT-GAGGGAAAGA--TGAAAAGAACTTTGAAAAGAGAGTTAAACAGTACGTGAAATTGTTGAAAGGGAAACGAAT-------------------------------------------------------------------------------------------------------------------------------------------------------------------------------------------------------------------------------------------------------------------------------------------------------------------------------------------------------------------------------------------------------------------------------------------------------------

>KP191475_Acaulospora_koskei

CCTAGTAAGCGTGAGTCATCAGCTCATGTTGATTACGTCCCT-GCCCTTTGTACACACCGCCCGTCGCTACTACCGATTGAAT-GGCTTAGTGAGACTCTCGGATCGGGTTTTGGGAACCGGCAACGGATCCTTTCTTCTGAGAAGTTTGTCAAACTTGGTCATTTAGAGGAAGTAAAAGTCGTAACAAGGTTTCCGTAGGTGAACCTGCGGAAGGATCATTAGAATA-TTCGGGAAT------------------TTTTTCCCT-------AAATAT------CTTATTC-AAAAAATTTCCATCCTATT---AAAAATA--TAATTTTTT-----AAATT------TT--ATCAATAAAAAATAAAGACAACTTTCAACAACGGATCTCTTGGCTCTTGCATCGATGAAGAACGCAGTGAAATGCGATACGTAATGTGAATTGCAGAATTCCGTGAATCATCAAATCTTTGAACGCAAATTGCAYTCCTT-GGTATTCCGAGGAGTATGCTTGCTTGAGGGTCGAATCAT---------------------A------TTATCGTTTTTT-------------AACGGATCTGGG---TTTTTCCAAG-TTTT--TTT-----------------------AAACCTTGGTAACCTA-AAATTTATCTTAATAATATT----------TGTAA-TA-TTGA-AAATGT----GTAT-CTTACG-----------------TTCACGTGA----GTT-TA-TTGACAATTATT----ATG--AATTTATTACGTCGTA--TTATTCACCCGG------------------------------TTT-TTTT--AA-CGATCGGGG--C-GAAATAA-TACAA-TTT------TTTAA--AATTCGACCTCAAGTC-AAGTAAG-AATACCCGCTGAAC-TT-AAGCATATCAATAAGCGGAGGAAAAGAAACTAAC-AAGGATTCCC-TTAG-TAAC-GGCGAGTGAACTGGGAAGAGCTCAAA-TTTAAAATCATCAGGG-TTT--CCTTGATGAGTTGTAATTTGAAGAAAGT-G-TTTTGAT-GTTCGGGTTTTATC-TAAATCCTTTGGGA--TAAGGTATCAT-AG-AGGGTGAGAATCCCGTTCGTGATTAAACCTCG---GAT--GTCACTTAATTCATTTTCTAAGAGTCGAGTTG-TTTGGGATTGCAGCTCAAAATGGGTGGTAAATTTCACCTAAGGCTAAATATATGCGAGAGACCGATAGCGAACAAGTACCGT-GAGGGAAAGA--TGAAAAGAACTTTGAAAAGAGAGTTAAATAGTACGTGAAATTGTTGAAAGGGAAACGATTGAAGTCAGTCATGCTAGTGAGGAATCAACTCGGTGGTT----------------------------------------------------------TTCTTCCGGGTGTACTTTCTC--GCCTGGCAGGTTAGCATCAA-TTTCGTTCATCATTAAATGATC-GGGGTAATGTGGCTTC---TCTTCGGAGTAGTG-TTATAGACCTTGGT-AGATGTGATGATCGGGATTGAGGATTGCAGCGAATGCT-ATTT------GGCTAGTCCCCTGA-TTTCTGATG-GTCACCTC--GCCTTCGACAGC-TTGCTGACGTGGGTGGGTTCTGATTGTCTAG-AT-GTTAGTGTGATCATATA-TTCGCTAAGGATGCTGACGTAATGGCTTTAAACGACCCGTCTTGAAACACGGACCAAGGAGTCTAAC-ATGTATGCGAGTGT-

>KP191476_Acaulospora_koskei

CCTAGTAAGCGTGAGTCATCAGCTCATGTTGATTACGTCCCT-GCCCTTTGTACACACCGCCCGTCGCTACTACCGATTGAAT-GGCTTAGTGAGACTCTCGGATCGGGTTTTGGGAACCGGCAACGGATCCTTTCTTCTGAGAAGTTTGTCAAACTTGGTCATTTAGAGGAAGTAAAAGTCGTAACAAGGTTTCCGTAGGTGAACCTGCGGAAGGATCATTAGAATA-TTCGGGAAT------------------TTTTTTCCT-------AAATAT------CTTATTC-AAAAAATTTCCATTCTATT---AAAAA-A--TAATTTTTT-----AAACT------TT--ATTA--AAAAAATAAAGACAACTTTCAACAACGGATCTCTTGGCTCTTGCATCGATGAAGAACGCAGTGAAATGCGATACGTAATGTGAATTGCAGAATTCCGTGAATCATCAAATCTTTGAACGCAAATTGCACTCCTT-GGTATTCCGAGGAGTATGCTTGCTTGAGGGTCGAATCAT---------------------A------TTATCGTTTTAT-------------AACGGATCTGGG---TTTTTCCAAGTTTTT--TTT-----------------------AAACCTTGGTAACCTA-AAATTTATCTTAATAATATT----------TGTAA-TA-TTGA-AAATGT----GTAT-CTTACG-----------------TTCACGTGA----GTT-TA-TTGACAATCATT----ATG--AATTTATTACGTCGTA--TTATTCACTCGA------------------------------TTT-TTTT--AA-CGATCGGGG--CGAAAATGA-TACAA-TTT------TTTAA--AATTCGACCTCAAGTC-AAGTAAG-AATACCCGCTGAAC-TT-AAGCATATCAATAAGCGGAGGAAAAGAAACTAAC-AAGGATTCCC-TTAG-TAAC-GGCGAGTGAACTGGGAAGAGCTCAAA-TTTAAAATCATCAGGG-TTT--CCTTGATGAGTTGTAATTTGAAGAAAGT-G-TTTTGAT-GTTCGGGTTTCATC-TAAATCCTTTGGGA--TAAGGTATCAT-AG-AGGGTGAGAATCCCGTTCGTGATTAAACCTCG---GAT--GTCACTTAATTCATTTTCTAAGAGTCGAGTTG-TTTGGGATTGCAGCTCAAAATGGGTGGTAAATTTCACCTAAGGCTAAATATATGCGAGAGACCGATAGCGAACAAGTACCGT-GAGGGAAAGA--TGAAAAGAACTTTGAAAAGAGAGTTAAATAGTACGTGAAATTGTTGAAAGGGAAACGATTGAAGTCAGTCATGCTAGTGAGGAATCAACTCGGTGGTT----------------------------------------------------------TTCTTCCGGGTGTACTTTCTC--GCCTGGCAGGTTAGCATCAA-TTTCGTTCATCATTAAATGATC-GGGGTAATGTGGCTTC---TCTTCGGAGTAGTG-TTATAGACCTTGGT-AGATGTGATGATCGGGATTGAGGATTGCAGCGAATGCT-ATTT------GGCTAGTCCCCTGA-TTTCTGATG-GTCACCTC--GCCTTCGACAGC-TTGCTGACGTGGGTGGGTTCTGATTGTCTAG-AT-GTTAGAGTGATCATATA-TTCGCTAAGGATGCTGACGTAATGGCTTTAAACGACCCGTCTTGAAACACGGACCAAGGAGTCTAAC-ATATGTGCGAGTGTT

>FR750063_Acaulospora_colombiana

CCTAGTAAGCGTGAGTCATCAGCTCACGTTGATTACGTCCCT-GCCCTTTGTACACACCGCCCGTCGCTACTACCGATTGAAT-GGCTTAGTGAGACTCTCGGATCGGGTTTGAGGAGCTGTAAAAGGCTCCTTATTTCTGAGAAGTTTGTCAAACTTGGTCATTTAGAGGAAGTAAAAGTCGTAACAAGGTTTCCGTAGGTGAACCTGCGGAAGGATCATTAGTAAT-TTCACTTTTCGGGTTGAA------TTCATTTCTTCC-------CGATTGGTCAAACGTATTC-AAAAAATTTACATCCTTTC---AAAT-----AAATCTTTTA----AAAAAA-TTTTTA--TTAAATGATAATGAAAGACAACTTTCAACAACGGATCTCTTGGCTCTTGCATCGATGAAGAACGCAGTGAAATGCGATAGTTAATGTGAATTGCAGAATTCCGTGAATCATCAAATCTTTGAACGCAAATTGCGCTTCTT-GGTATTCCGAGGAGCATGCTTGCTTGAGTGTTAGTCCA---TAAAAATATC-GTGAAATA--------------TATTT-------------CGCGGATCTGGG---TTTTTCCAAG---GT--TTC------------------GAATGAATCGTTGGTAACCTG-AAATTGATTTTAACGATATT----------CGTGATTT-TTGA-AAATGT----TTAT-CTCATT-----------------CTCTTATGG----GTT-CT-TTGACGATCATC----TCG--AATTTGTTACGTCGTG--TCATTTGAT--------C----------------------------TCGGC-GAG------TGG--T-CATCTGA-CACAA-TTT------TTTGA--ATTCTGGCCTCAAGTC-AAGTAAG-ATTACCCGCTGAAC-TT-AAGCATATCAATAAGCGGAGGAAAAGAAACTAAC-AAGGATTCCC-TTAG-TAAC-GGCGAGTGAAGTGGGAAGAGCTCAAA-TTTTAAATCCCCGGGG-TTC--CCCCGGTGAATTGTATTTTGAAGAAAGT-G-TTTTGAC-ATTC-GGTTTAATC-TAAATCCTTTGGGA--TAAGGTATCAC-AG-AGGGTGAGAATCCCGTTCGTGATTAGGCATCG---GGT--GTCATCCAATTCACTTTCTAAGAGTCGAGTTG-TTTGGGATTGCAGCTCAAAATGGGTGGTAAATTTCACCTAAGGCTAAATATCAGCAAGAGACCGATAGCGAACAAGTACCGT-GAGGGAAAGA--TGAAAAGAACTTTGAAAAGAGAGTTAAATAGTACGTGAAATTGTTGAAAGGGAAACGATTGAAGTCAGTCATGCTGGTGAGGAATCAACACGAGGGTT----------------------------------------------------------CGCTTTCGTGTGCACTTCTTC--GCTTGGCAGGTTAGCATCAA-TTTTGGTCGTCATAAAATTATC-GGGGTAAGGTGGCTCC---CCTC--GGGGAGTG-TTATAGACCTTGGT-AGATGTGATGACCGGGATTGAGGATTGCAGCGAATGCT-ACTT------GGCTAGTCACCTGGATTTCTGTTG-ATTACCTC--GCCGTCGATAGCTTTGCTAACCTCGGTGGGATCTGATTAACTAG-AG-ATTAGACTGATCGTAAA-TTCGCTAAGGATGCTGACGTAATGGCTTTAAACGACCCGTCTTGAAACACGGACCAAGGAGTCTAAC-ATGTGTGCGAGTGTT

>FJ461804_Acaulospora_colombiana

-----------------------------------------------------------------------------------------------------------------------------------------------------------------------------------------------------------------------------------------------------------------------------------------------------------------------------------------------------------------------------------------------------------------------------------------------------------------------------------------------------------------------------------------------------------------------------------------------------------------------------------------------------------------------------------------------------------------------------------------------------------------------------------------------------------------------------------------------------------------------------------------------------------------------------------------------------------------------------------------------------------CTAAC-AAGGATTCCC-TTAG-TAAC-GGCGAGTGAAGTGGGAAGAGCTCAAA-TTTTAAATCCCCGGGG-TTC--CCCCGGTGAATTGTATTTTGAAGAAAGT-G-TTTTGAC-ATTC-GGTTTAATC-TAAATCCTTTGGGA--TGAGGTATCAC-AG-AGGGTGAGAATCCCGTTCGTGATTAGGCATCG---GGT--GTCATCCAATTCACTTTCTAAGAGTCGAGTTG-TTTGGGATTGCAGCTCAAAATGGGTGGTAAATTTCACCTAAGGCTAAATATCAGCAAGAGACCGATAGCGAACAAGTACCGT-GAGGGAAAGA--TGAAAAGAACTTTGAAAAGAGGGTTAAATAGTACGTGAAATTGTTGAAAGGGAAACGATTGAAGTCAGTCATGCTGGTGAGGAATCAACACGAGGGTT----------------------------------------------------------CGCTCTCGTGTGCACTTCTTC--GCTTGGCAGGTTAGCATCAA-TTTTGGTCGTCATAAAATTATC-GGGGTAAGGTGGCTCC---CCTC--GGGGAGTG-TTATAGACCCTGGT-AGATGTGATGACCGGGATTGAGGATTGCAGCGAATGCT-ACTT------GGCTAGTCACCTGGATTTCTGTTG-ATCACCTC--GCCGTCGATAGCTTTGCTAACCTCGGTGGGATCTGATTAACTAG-AG-ATTAGACTGATCGTAAA-TTCGCTAAGGATGC---------------------------------------------------------------------

>Acaulospora_flavopapillosa_OK360960

CCTAGTAAGCGTGAGTCATCAGCTCACGTTGATTACGTCCCT-GCCCTTTGTACACACCGCCCGTCGCTACTACCGATTGAAT-GGCTTAGTGAGACTCTCGGATCGGGTTTTAGGAACCGGAAACGGATCCTTTTTTCTGAGAAGTTTGTCAAACTTGGTCATTTAGAGGAAGTAAAAGTCGTAACAAGGTTTCCGTAGGTGAACCTGCGGAAGGATCATTAGAAA--------------------------------TTTT------------------TTATGTATTC-AAAA--TTTCAATCTATAT---AAAAT-------TTTTTATT---TATAT----------AAAAT-----AAAAAAGACAACTTTCAACAACGGATCTCTTGGCTCTTGCATCGATGAAGAACGCAGCGAAATGCGATAAGTAATGTGAATTGCAGAATTCCGTGAATCATCAAATCTTTGAACGCAAATTGCACTCTTT-GGTATTCCGAAGAGTATGCTTGCTTGAGGGTTATTTTA---ATAATATCA--------------------TTTTTT-------ATAATGAAAAATGGATCTGAG---TTTTCCAAGG-TTTT--TTA-----------------------GAACTTTGGTAACTTC-AAATTTATCTTAACGATATTTTAG------TA--A-TA-TTGG-AAATGT-T--TTAT-CTTGCATC------------------ACGTGAG---TTT-CA-TTAACAATTAAT---TACT--AAATCGTTAGAGCGTG--TTATTTACCTAA------------------------------TTT-TTAT--AA----TT-GGGTTT-TGTATAA-TACAA-ATTT-----T------TTTTAAACCTCAAGTC-AAGTAAG-ATTACCCGCTGAAC-TT-AAGCATATCAATAAGCGGAGGAAAAGAAACTAAC-AAGGATTCCC-TTAG-TAAC-GGCGAGTGAAGTGGGAAAAGCTCAAA-TTTTAAATCACCGGGG-TTC--CCTTGGTGAATTGTAATTTGAAGAAAGT-G-TTTTGAC-GTTCGGGTTCGATT-TAAATCCTTTGGGA--TAGGGTATCAT-AG-AGGGTTAGAATCCCGTTTATGATTGATCCTTT---GAA--TGTCTATAATTCACTTTCCAAGAGTCGAGTTG-TTTGGGATTGCAGCTCAAAAAGGGTGGTAAATTTCACCTAAGGCTAAATATGTGCAAGAGACCGATAGCGAACAAGTACCGT-GAGGGAAAGA--TGAAAAGAACTTTGAAAAGAGAGTCAAATAGTACGTGAAATTGTTGAAAGGGAAACGATTAAAGTCAGTCATGTTGGTAGGGAATCAACTTGTTGGTA-------TGCGGG----------------------------------TTTA-CTCGTGTCCTTTCCGGTGCACTTCCTT--ACTT-ACAGGTTAGCATCAA-TTTTGGTTATCATATAAATATT-GAGGGAAGGTGGCTTT---TCTTCGGAAAAGTG-TTATAGACCTTGGT-AAATGTGGTGACCGGGATTGAGGATTGCAGCGGATACC-CTTTT----GGGCTAGTCACCTGG-CTTCTGATG-GTCAACCC--GTCTTCGATAGC-TTGCTAACGTTGGTGGGATCTGATTGTCTAG-AT-GTTGGAGTGATCATAAA-TTCGCTAAGGATGCTGACGTAATGGCTTTAAACGACCCGTCTTGAAACACGGACCAAGGAGTCTAAC-ATGTGTGCGAGTGTT

>Acaulospora_flavopapillosa_OK360962

CCTAGTAAGCGTGAGTCATCAGCTCACGTTGATTACGTCCCT-GCCCTTTGTACACACCGCCCGTCGCTACTACCGATTGAAT-GGCTTAGTGAGACTCTCGGATCGGGCTTTAGGAACCGGAAACGGATCCTTTTTTCTGAGAAGTTTGTCAAACTTGGTCATTTAGAGGAAGTAAAAGTCGTAACAAGGTTTCCGTAGGTGAACCTGCGGAAGGATCATTAGAAA--------------------------------TTTT------------------TTATGTATTC-AAAA--TTTCAATCTATAA--AATAAT-------TTTTTATT---TATAT----------AAAAT-----AAAAAAGACAACTTTCAACAACGGATCTCTTGGCTCTTGCATCGATGAAGAACGCAGCGAAATGCGATAAGTAATGTGAATTGCAGAATTCCGTGAATCATCAAATCTTTGAACGCAAATTGCACTCTTT-GGTATTCCGAAGAGTATGCTTGCTTGAGGGTTATTCTA---ATAATATCA--------------------TTTTT--------ATAATGAAAAATGGATCTGAG---TTTTCCAAGG-TTTT--TTA-----------------------GGACTTTGGTAACTTT-AAATTTATCTTAACGATATTTTAG------TA--A-TA-TTGG-AAATGT-T--TTAT-CTTGCATC------------------TCGCGAG---TTT-CA-TTAACAATTAAT---TATT--AAATCGTTAGAGCGTA--TTATTTATCTAA-------------------------------CT-TTAT--AA----TT-GGG--T-TATATAA-TACAA-TTTT-----T------TTTTAAACCTCAAGTC-AAGTAAG-ATTACCCGCTGAAC-TT-AAGCATATCAATAAGCGGAGGAAAAGAAACTAAC-AAGGATTCCC-TTAG-TAAC-GGCGAGTGAAGTGGGAAAAGCTCAAA-TTTTAAATCATCGGGG-TTC--CCTCGGTGAATTGTAATTTGAAGAAAGT-G-TTTTGAC-GTTCGGGTTCGATT-TAAATCCTTTGGGA--TAAGGTATCAT-AG-AGGGTTAGAATCCCGTTTATGATTGATCCTTT---GAA--TGTCTATAATTCACTTTCCAAGAGTCGAGTTG-TTTGGGATTGCAGCTCAAAAAGGGTGGTAAATTTCACCTAAGGCTAAATATGTGCAAGAGACCGATAGCGAACAAGTACCGT-GAGGGAAAGA--TGAAAAGAACTTTGAAAAGAGAGTTAAATAGTACGTGAAATTGTTGAAAGGGAAACGATTAAAGTCAGTCATGTTGGTAGGGAATCAACTTGTTGGTA-------TGCGGG----------------------------------TTTA-CTCGTGTCCTTTCCGGTGCACTTCCTT--ACTT-GCAGGTTAGCATCAA-TTTTGGTTATCATATAAATATT-GAGGGAAGGTGGCTTT---TCTTCGGAAAAGTG-TTATAGACCTTGGT-AAATGTGGTGACCGGGATTGAGGATTGCAGCGGATACC-CTTTC----GGGCTAGTCACCTGG-CTTCTGATG-GTCAACCC--GTCTTCGATAGC-TTGCTAACGTTGGTGGGACCTGATTGTCTAG-AT-GTTGGAGTGATCATAAA-TTCGCTAAGGATGCTGACGTAATGGCTTTAAACGACCCGTCTTGAAACACGGACCAAGGAGTCTAAC-ATATGTGCGAGTGTT

>LN884303_Acaulospora_papillosa

CCTAGTAAGCGTGAGTCATCAGCTCACGTTGATTACGTCCCT-GCCCTTTGTACACACCGCCCGTCGCTACTACCGATTGAAT-GGCTTAGTGAGACTCTCGGATCGGGTTTTAGGAACCGGAAACGGATCCTTTTTTCTGAGAAGTTTGTCAAACTTGGTCATTTAGAGGAAGTAAAAGTCGTAACAAGGTTTCCGTAGGTGAACCTGCGGAAGGATCATTAGAAA---------------------------------TTT------------------TTATGTATTC-AAAA--TTTCAATCTTTAT--AAAAGTTT--ATATTTTTATT---TATAT----------AAAAT-----AAAAAAGACAACTTTCAACAACGGATCTCTTGGCTCTTGCATCGATGAAGAACGCAGCGAAATGCGAAAAGTAATGTGAATTGCAGAATTCCGTGAATCATCAAATCTTTGAACGCAAATTGCACTCTTT-GGTATTCCGAAGAGTATGCTTGCTTGAGGGTTGTTTCA---ATAATATCG--------------------TTTTTTT-----ATAAT-AAAAAACGGAACTGAG---TTTTCCAAAG-TTTT--TTA-----------------------TAACTTTGGTAACTTT-AAATTTATCTTAACGATATTAAG-------TAT-C-TA-TTGG-AAATGT-T--TTAT-CTTACATC------------------TCGTGAG---TTT-CA-TTAACAATTATT---TACT--AAGTCGTTAGAGCGTG--TTATTTGCCTAA------------------------------TTT-TTAT--AA----TTTGGGTTA-TATATAA-TACAA-TTTT-----TTT----TTTCAAACCTCAAGTC-AAGTAAG-ATTACCCGCTGAAC-TT-AAGCATATCAATAAGCGGAGGAAAAGAAACTAAC-AAGGATTCCC-TTAG-TAAC-GGCGAGTGAAGTGGGAAAAGCTCAAA-TTTTAAATCACCCGGG-TTC--CCTTGGTGAATTGTAATTTGAAGAAAGT-G-TTTTGAC-GTTCGGGTTCGATT-TAAATCCTTTGGGA--TAAGGTATCAT-AG-AGGGTTAGAATCCCGTTTATGATTGATCCTTT---GAA--TGTCTATAATTCACTTTCCAAGAGTCGAGTTG-TTTGGGATTGCAGCTCTAAAAGGGTGGTAAATTTCACCTAAGGCTAAATATGTGCAAGAGACCGATAGCGAACAAGTACCGT-GAGGGAAAGA--TGAAAAGAACTTTGAAAAGAGAGTCAAATAGTACGTGAAATTGTTGAAAGGGAAACGATTAAAGTCAGTCATGTTGGTGGGGAATCAACTTGATAGTA-------TGCGGG----------------------------------TTTACCTCGTGTCCTTTCTAGTCCACTTTCTC--ACTT-GCAGGTTAGCATCAA-TTTCGGTTATCATATAAAAATT-GAGGAAAGGTGGCTTT---TCTTCGGAAAAGTGTTTATAGACCTTGAG-AAATGTGGTGACCGGGATTGAGGATTGCAGCGGATACC-CATTT----GGGCTAGTCACCTGG-CTTCTGATG-GTTGCCCC--GTCTTCGATAGC-TTGCTAACGTTGGTGGGATTCGATTATCTAG-AT-GTTGGAGTGATCATAAA-TTCGCTAAGGATGCTGACGTAATGGCTTTAAGCGACCCGTCTTGAAACACGGACCAAGGAGTCTAAC-ATGTATGCGAGTGTT

>LN884302_Acaulospora_papillosa

CCTAGTAAGCGTGAGTCATCAGCTCACGTTGATTACGTCCCT-GCCCTTTGTACACACCGCCCGTCGCTACTACCGATTGAAT-GGCTTAGTGAGACTCTCGGATCGGGTTTTAGGAACCGGAAACGGATCCTTTTTTCTGAGAAGTTTGTCAAACTTGGTCATTTAGAGGAAGTAAAAGTCGTAACAAGGTTTCCGTAGGTGAACCTGCGGAAGGATCATTAGAAA---------------------------------TTT------------------TTATGTATTC-AAAA--TTTCAATCTTTAT--AAAAGTTT--ATATTTTTATT---TATAT----------AAAAT-----AAAAAAGACAACTTTCAACAACGGATCTCTTGGCTCTTGCATCGATGAAGAACGCAGCGAAATGCGAAAAGTAATGTGAATTGCAGAATTCCGTGAATCATCAAATCTTTGAACGCAAATTGCACTCTTT-GGTATTCCGAAGAGTATGCTTGCTTGAGGGTTGTTTCA---ATAATATCG--------------------TTTTTTT-----ATAATAAAAAAACGGAACTGAG---TTTTCCAAAG-TTTT--TTA-----------------------TAACTTTGGTAACTTT-AAATTTATCTTAACGATATTAAG-------TAT-C-TA-TTGG-AAATGT-T--TTAT-CTTACATC------------------TCGTGAG---TTT-CA-TTAACAATTATT---TACT--AAGTCGTTAGAGCGTG--TTATTTGCCTAA------------------------------TTT-TTAT--AA----TTTGGGTTA-TATATAA-TACAA-TTTT-----TTT----TTTCAAACCTCAAGTC-AAGTAAG-ATTACCCGCTGAAC-TT-AAGCATATCAATAAGCGGAGGAAAAGAAACTAAC-AAGGATTCCC-TTAG-TAAC-GGCGAGTGAAGTGGGAAAAGCTCAAA-TTTTAAATCACCCGGG-TTC--CCTTGGTGAATTGTAATTTGAAGAAAGT-G-TTTTGAC-GTTCGGGTTCGATT-TAAATCCTTTGGGA--TAAGGTATCAT-AG-AGGGTTAGAATCCCGTTTATGATTGATCCTTT---GAA--TGTCTATAATTCACTTTCCAAGAGTCGAGTTG-TTTGGGATTGCAGCTCAAAAAGGGTGGTAAATTTCACCTAAGGCTAAATATGTGCAAGAGACCGATAGCGAACAAGTACCGT-GAGGGAAAGA--TGAAAAGAACTTTGAAAAGAGAGTCAAATAGTACGTGAAATTGTTGAAAGGGAAACGATTAAAGTCAGTCATGTTGGTGGGGAATCAACTTGATAGTA-------TGCGGG----------------------------------TTTACCTCGTGTCCTTTCTAGTGCACTTTCTC--ACTT-GCAGGTTAGCATCAA-TTTCGGTTATCATATAAAAATT-GAGGAAAGGTGGCTTT---TCTTCGGAAAAGTGTTTATAGACCTTGAG-AAATGTGGTGACCGGGATTGAGGATTGCAGCGGATACC-CATTT----GGGCTAGTCACCTGG-CTTCTGATG-GTTGCCCC--GTCTTCGATAGC-TTGCTAACGTTGGTGGGATTCGATTATCTAG-AT-GTTGGAGTGATCATAAA-TTCGCTAAGGATGCTGACGTAATGGCTTTAAGCGACCCGTCTTGAAACACGGACCAAGGAGTCTAAC-ATGTGTGCGAGTGTT

>JF439093_Acaulospora_delicata

CCTAGTAAGCGTGAGTCATCAGCTCACGTTGATTACGTCCCT-GCCCTTTGTACACACCGCCCGTCGCTACTACCGATTGAAT-GGCTTAGTGAGACTCTCGGATCGGGTTTTAGGAACCGGCAACGGATCCTTTCTTCTGAGAAGTTTGTCAAACTTGGTCATTTAGAGGAAGTAAAAGTCGTAACAAGGTTTCCGTAGGTGAACCTGCGGAAGGATCATTAGAAA---------------------------------TTT------------------TTATGTATTC-AAAA--TTTCAATCTTTAT-AAAAAAA-------TTTTTATT---TATAT----------AAAAT-----AAAAAAGACAACTTTCAACAACGGATCTCTTGGCTCTTGCATCGATGAAGAACGCAGCGAAATGCGATAAGTAATGTGAATTGCAGAATTCCGTGAATCATCAAATCTTTGAACGCAAATTGCACTCTTT-GGTATTCCGAAGAGTATACTTGCCTGAGGGTTGTTCTA---ACAATATCG--------------------TTTTTTTTAT--AATAAAAAAAAACGGATCTGAG---TTTTCTGAGG-TTTT--TAT-----------------------AACCTTTGGTAACTTT-AAATTTATCTTAACGATATATTAG------TAT-A-TA-TTGG-AAATGT-T--TAAT-CTTGCATC------------------TCGTGAG---TTT-CA-TTAACAATTAAT---TACT--AAATCGTTAGAGCGTT--TTATTTACCTAA------------------------------TTT-TTAT--AA----TT-GGG--T-TATATAA-TACAA-TCTT-----TTT----TTTCAAACCTCAAGTC-AAGTAAG-ATTACCCGCTGAAC-TT-AAGCATATCAATAAGCGGAGGAAAAGAAACTAAC-AAGGATTCCC-TTAG-TAAC-GGCGAGTGAAGTGGGAAAAGCTCAAA-TTTTAAATCACCCGGG-TTA--CCTTGGTGAATTGTAATTTGAAGAAAGT-G-TTTTGAC-GTTCGGGTTCGATT-TAAATCCTTTGGGA--TAAGGTATCAT-AG-AGGGTTAGAATCCCGTTTATGATTGATCCTTT---GAA--TGTCTATAATTCACTTTCCAAGAGTCGAGTTG-TTTGGGATTGCAGCTCAAAATGGGTGGTAAATTTCACCTAAGGCTAAATATATGCAAGAGACCGATAGCGAACAAGTACCGT-GAGGGAAAGA--TGAAAAGAACTTTGAAAAGAGAGTTAAATAGTACGTGAAATTGTTGAAAGGGAAACGATTAAAGTCAGTCATGTTGGTAGGGAATCAACTTGATGGTA-------TGCGGG----------------------------------TTTA-CTCGTGTTCTTTCGGGTGCACTTCCTT--ACTT-GCAAGTTAGCATCAA-TTTTGATTATCATATAAAAATC-GAGGGAAGGTGGCTTT---TCTTCGGAAAAGTG-TTATAGACCTCGAG-AAATGTGGTGACCGGGATTGAGGATTGCAGCGGATACC-CTTTT----GGGCTAGTCACCTGG-CTTCTGATG-GTTACCCT--GTCTTCGACAGC-TTGCTGACGTTGATGGAATTCGATTGTCTAG-AT-GTTAGAGTGATCATAAA-TTCGCTAAGGATGCTGACGTAATGGCTTTAAACGACCCGTCTTGAAACACGGACCAAGGAGTCTAAC-ATGTATGCGAGTGTT

>JF439203_Acaulospora_delicata

CCTAGTAAGCGTGAGTCATCAGCTCACGTTGATTACGTCCCT-GCCCTTTGTACACACCGCCCGTCGTTACTACCGATTGAAT-GGCTTAGTGAGACTCTCGGATCGGGTTTTAGGAACCGGCAACGGATCCTTTCTTCTGAGAAGTTTGTCAAACTTGGTCATTTAGAGGAAGTAAAAGTCGTAACAAGGTTTCCGTAGGTGAACCTGCGGAAGGATCATTAGAAA--------------------------------TTTT------------------TTATGTATTC-AAAA--TTTCAATCTTTATAAAAAAAA-------ATTTTATT---TATAT----------AAAATAAAAAAAAAAAGACAACTTTCAACAACGGATCTCTTGGCTCTTGCATCGATGAAGAACGCAGCGAAATGCGATAAGTAATGTGAATTGCAGAATTCCGTGAATCATCAAATCTTTGAACGCAAATTGCACTCTTT-GGTATTCCGAAGAGTATACTTGCTTGAGGGTTGTTCTA---ATAGTATCG--------------------TTTTTTTAAT--AAT-AAAAAAAACGGATCTGAG---TTTTCCAAGG--TTT--TAT-----------------------AAACTTTGGTAACTTT-AAATTTATCTTAATGATATTTTAG------TAT-A-TA-TTGA-AAATGT-T--TTAT-CTTACATC------------------TCGTGAG---TTT-CA-TTAACAATTAAT---TACT--AAATCATTATAGCGTA--TTATTTACTTAA------------------------------TTT-TTAT--AA----TT-AAG--T-TATATGA-TACAATTTTT-----TTT----TTTCAAACCTCAAGTC-AAGTAAG-ATTACCCGCTGAAC-TT-AAGCATATCAATAAGCGGAGGAAAAGAAACTAAC-AAGGATTCCCTTTAG-TAAC-GGCGAGTGAAGTGGGAAAAGCTCAAA-TTTTAAATCACCCAGG-TTA--CCTTGGTGAATTGTAATTTGAAGAAAGT-G-TTTTGAC-GTTCGGGTTCGATT-TAAATCCTTTGGGA--TAAGGTATCAT-AG-AGGGTTAGAATCCCGTTTATGATTGATCCTTT---GAA--TGTCTATAATTCACTTTCCAAGAGTCGAGTTG-TTTGGGACTGCAGCTCAAAATGGGTGGTAAATTTCACCTAAGGCTAAATATATGCAAGGGACCGATAGCGAACAAGTACCGT-GAGGGAAAGA--TGAAAAGAACTTTGAAAAGAGAGTTAAATAGTACGTGAAATTGTTGAAAGGGAAACGATTAAAGTCAGTCATGTTGGTAGGGAATCAACTTGATGGTA-------TGCGAG----------------------------------TTTA-CTCGTGTTCTTTCGGGTGCACTTCCTT--ACTT-GCAAGTTAGCATCAA-TTTTGGTTATCATAAAAAAATT-GAGGGAAGGTGGCTTT---TCTTCGGAAAAGTG-TTATAGACCTTGAG-AAATGTGATGACTGGGATTGAGGATTGCAGCGGATACC-CTTTT----GGGCTAGTCACCTGG-CTTCTGATG-GTTACCCC--GTCTTCGACAGC-TTGCTGACGTTGATGGGACTCGATTGTCTAG-AT-GTTGGAGTGATCGTAAA-TTCGCTAAGGATGCTGACGTAATGGCTTTAAACGACCCGTCTTGAAACACGGACCAAGGAGTCTAAC-ATGTGTGCGAGTGTT

>MT832212_Acaulospora_dilatata

---------------------------------------------------------------------------------------------------------------------------------------------------------------------------------------------------------------------------------------------------------------------------------------------------------------------------------------------------------------------------------------------------------------------------------------------------------------------------------------------------------------------------------------------------------------------------------------------------------------------------------------------------------------------------------------------------------------------------------------------------------------------------------------------------------------------------------------------------------------------------------------------------------------------------------------------------------------------------------------------------------------------------------------GCGAGTGAAGTGGGAAAAGCTCAAA-TTTTAAATCACCTGGG-TTT--CCTTGGTGAATTGTAATTTGAAGAAAGT-G-TTTTGAC-GTTCGGGTTCGATT-TAAATCCTTTGGGA--TAAGGTATCAT-AG-AGGGTTAGAATCCCGTTTATGATTGTTCCTTT---GAA--TGTCTATAATTCACTTTCCAAGAGTCGAGTTG-TTTGGGATTGCAGCTCAAAAAGGGTGGTAAATTTCACCTAAGGCTAAATATATGCAAGAGACCGATAGCGAACAAGTACCGT-GAGGGAAAGA--TGAAAAGAACTTTGAAAAGAGAGTTAAATAGTACGTGAAATTGTTGAAAGGGAAACGATTAAAGTCAGTCATGTTGGTAGGGAATCAACTTGATGGTT-------TGCGGG----------------------------------TTTA-CTCGTGTTCTTTCGAGTGCACTTCCTT--ACTT-GCTAGTTAGCATCAA-TTTTGGTTATCATATAAAAATT-GAGGGAAGGTGGCTTT---TCTTCGGAAAAGTG-CTATAGACCTTGAT-TAATGTGGTGACCGGGATTGAGGATTGCAGCGGATACC-CTTT------GGCTAGTCACCTTG-CTTCTGATG-GTTACCCC--GTCTTCGATAGC-TTGCTAACGTTGATGGGATTTAATTGTCTAG-AT-GTTGGAGTGATCATAAA-TTCGCTAAGGATGC---------------------------------------------------------------------

>FJ461792_Acaulospora_dilatata

-----------------------------------------------------------------------------------------------------------------------------------------------------------------------------------------------------------------------------------------------------------------------------------------------------------------------------------------------------------------------------------------------------------------------------------------------------------------------------------------------------------------------------------------------------------------------------------------------------------------------------------------------------------------------------------------------------------------------------------------------------------------------------------------------------------------------------------------------------------------------------------------------------------------------------------------------------------------------------------------------------------CTAAC-AAGGATTCCC-TTAG-TAAC-GGCGAGTGAAGTGGGAAAAGCTCAAA-TTTTAAATCACCTGGG-TTT--CCTTGGTGAATTGTAATTTGAAGAAAGT-G-TTTTGAC-GTTCGGGTTCGATT-TAAATCCTTTGGGA--TAAGGTATCAT-AG-AGGGTTAGAATCCCGTTTATGATTGTTCCTTT---GAA--TGTCTATAATTCACTTTCCAAGAGTCGAGTTG-TTTGGGATTGCAGCTCAAAAAGGGTGGTAAATTTCACCTAAGGCTAAATATATGCAAGAGACCGATAGCGAACAAGTACCGT-GAGGGAAAGA--TGAAAAGAACTTTGAAAAGAGAGTTAAATAGTACGTGAAATTGTTGAAAGGGAAACGATTAAAGTCAGTCATGTTGGTAGGGAATCAACTTGATGGTT-------TGCGGG----------------------------------TTTA-CTCGTGTTCTTTCGAGTGCACTTCCTT--ACTT-GCTAGTTAGCATCAA-TTTTGGTTATCATATAAAAATT-GAGGGAAGGTGGCTTT---TCTTCGGAAAAGTG-CTATAGACCTTGAT-TAATGTGGTGACCGGGATTGAGGATTGCAGCGGATACC-CTTT-----GGGCTAGTCACCTTG-CTTCTGATG-GTTACCCC--GTCTTCGATAGC-TTGCTAACGTTGATGGGATTTAATTGTCTAG-AT-GTTGGAGTGATCATAAA-TTCGCTAAGGATGC---------------------------------------------------------------------

>LN881565_Acaulospora_rugosa

CCTAGTAAGCGTGAGTCATCAGCTTACGTTGATTACGTCCCT-GCCCTTTGTACACACCGCCCGTCGCTACTACCGATTGAAT-GGCTTAGTGAGACTCTCGGATCGGGTTTTAGGAACCGGCAACGGATCCTTTCTTCTGAGAAGTTTGTCAAACTTGGTCATTTAGAGGAAGTAAAAGTCGTAACAAGGTTTCCGTAGGTGAACCTGCGGAAGGATCATTAGAAA--------------------------------TTTTA-----------------TTATGTATTC-AAAA--TTTCAATCTATA--AAAAAAT-------TTTTTATT---TATAT----------AAAAT-AAAAAAAAAAGACAACTTTCAACAACGGATCTCTTGGCTCTTGCATCGATGAAGAACGCAGCGAAATGCGATAAGTAATGTGAATTGCAGAATTCCGTGAATCATCAAATCTTTGAACGCAAATTGCACTCTTT-GGTATTCCGAAGAGTATACTTGCTTGAGGGTTGTTTTA---ATAATATCG--------------------TTTTTTT-AT--AAT-AAAAAAAACGGATCTGAG---TTTTCCAAGG-TTTT--TTA-----------------------TAACTTTGGTAACTTT-AAATTTATCTTAACGATATATTGG------TAT-A-TA-TTGG-AAATGT-T--TTAT-CTTACATC------------------TCGTGAG---TTT-CC-TTTCCAATTAAT---TTCT--AAATCGTTATAGCGTA--TTATTTATTTAA------------------------------TTT-TTAT--AA----TT-GGG--T-TATATAA-TACATTTTTT-----TTT----TTTCAAACCTCAAGTC-AAGTAAG-ATTACCCGCTGAAC-TT-AAGCATATCAATAAGCGGAGGAAAAGAAACTAAC-AAGGATTCCC-TTAG-TAAC-GGCGAGTGAAGTGGGAAAAGCTCAAA-TTTTAAATCACCCGGG-TTT--CCTTGGTGAATTGTAATTTGAAGAAAGT-G-TTTTGAC-GTTCGGGTTCGATT-TAAATCCTTTGGGA--TAAGGTATCAT-AG-AGGGTTAGAATCCCGTTTATGATTGTTCCTTT---GAA--TGTCTATAATTCACTTTCCAAGAGTCGAGTTG-TTTGGGATTGCAGCTCAAAAAGGGTGGTAAATTTCACCTAAGGCTAGATATATGCAAGAGACCGATAGCGAACAAGTACCGT-GAGGGAAAGA--TGAAAAGAACTTTGAAAAGAGAGTTAAATAGTACGTGAAATTGTTGAAAGGGAAACGATTGAAGTCAGTCATGTTGGTAGGGAATCAACTTTGATGGTA-----ATGCGGG----------------------------------TTTA-CTCGTGTTCTTTCGAGTGCACTTCCTT--ACTT-ACTAGTTAGCATCAA-TTTTGGTTATCATATAAAAATT-GAGGGAAGGTGGCTTT---TCTTCGGAAAAGTG-TTATAGACCTTGAG-AAATGTGGTGACCGGGATTGAGGATTGCAGCGGATACC-TTTTT----GGGCTAGTCACCTGA-CTTCTGATG-GTTACCCC--GTCTTCGATAGC-TTGCTAACGTTGATGGGATTTAATTGTCTAG-AT-GTTGGAGTGATCATAAA-TTCGCTAAGGATGCTGACGTAATGGCTTTAAACGACCCGTCTTGAAACACGGACCAAGGAGTCTAAC-ATATGTGCGAGTGTT

>AM040291_Acaulospora_longula

------------------------------------------------------------------------------------------------------------------------------------------------------------------------------------------------------------------------------------------------------------------------------------------------------------------------------------------------------------------------------------------------------------------------------------------------------------------------------------------------------------------------------------------------------------------------------------------------------------------------------------------------------------------------------------------------------------------------------------------------------------------------------------------------------------------------------------------------------------------------------------------------------------------------------------------------------------------------------------------------------AGAAACTAAC-AAGGATTCCC-TTAGCTAAC-GGCGAGTGAAGTGGGAAAAGCTCAAA-CTTTAAATCACCCGGG-TTT--CCTTGGTGAATTGTAATTTGAAGAAAGT-G-TTTTGAC-GTTCGGGATCGATT-TAAATCCTTTGGGA--TAAGGTATCAT-AG-AGGGTTAGAATCCCGTTTATGATTGTTCCTTT---GAA--TGTCTATAATTCACTTTCCAAGAGTCGAGTTG-TTTGGGATTGCAGCTCAAAAAGGGTGGTAAATTTCACCTAAGGCTAAATATATGCAAGAGACCGATAGCGAACAAGTACCGT-GAGGGAAAGA--TGAAAAGAACTTTGAAAAGAGAGTTAAATAGTACGTGAAATTGTTGAAAGGGAAACGATTAAAGTCAGTCATGTTGGTAGGGAATCAACTTGATGGTT-------TGCGGG----------------------------------TTTA-CTCGTGTTCTTTCGAGTGCACTTCCTT--ACTT-GCTAGTTAGCATCAA-TTTTGATTATCATATAAAAATT-GAGGGAAGGTGGCTTT---TCTTCGGAAAAGTG-TTATAGGCCTCGAG-AAATGTGGTGATCGGGATTGAGGATTGCAGCGGATACC-CTTTT---AGGGCTAGTCACCTGG-CTTCTGATG-ATTACCTC--GCCTTCGATAGC-TTGCTGACGTTGGTGGGACTCGATTGTCTAG-AT-GTTGGAGTGATCATAAA-TTCGTTAAGGATGT-GACGA---------------------------------------------------------------

>AM040292_Acaulospora_longula

--------------------------------------------------------------------------------------------------------------------------------------------------------------------------------------------------------------------------------------------------------------------------------------------------------------------------------------------------------------------------------------------------------------------------------------------------------------------------------------------------------------------------------------------------------------------------------------------------------------------------------------------------------------------------------------------------------------------------------------------------------------------------------------------------------------------------------------------------------------------------------------------------------------------------------------------------------------------------------GCATATCAATAAGCGGAGGAAAAGAAACTAAC-AAGGATTCCC-TTAG-TAAC-GGCGAGTGAAGTGGGAAAAGCTCAAA-TTTTAAATCACCTGGG-TTT--CCTTGGTGAATTGTAATTTGAAGAAAGT-G-TTTTGAC-GTTCGGGAACGATT-TAAATCCTTTGGGA--TAAGGTGTCAT-AG-AGGGTTAGAATCCCGTTTATGATTGTTCCTTT---GAA--TGTCTATAATTCACTTTCCAAGAGTCGAGTTG-TTTGGGATTGCAGCTCAAAAAGGGTGGTAAATTTCACCTAAGGCTAAATATATGCAAGAGACCGATAGCGAACAAGTACCGT-GAGGGAAAGA--TGAAAAGAACTTTGAAAAGAGAGTTAAATAGTACGTGAAATTGTTGAAAGGGAAACGATTAAAGTCAGTCATGTTGGTAGGGAATCAACTTGATGGTT-------TGCGGG----------------------------------TTTA-CTCGTGTTCTTTCGAGTGCACTTCCTT--ACTT-GCTAGTTAGCATCAA-TTTTGATTATCATATAAAAATT-GAGGGAAGGTGGCTTT---TCTTCGGAAAAGTG-TTATAGACCTTGAT-TAATGTGGTGATCGGGATTGAGGATTGCAGCGGATACC-CTTTT---AGGGCTAGTCACCTGG-CTTCTGATG-ATTACCTC--GCCTTCGATAGC-TTGCTGACGTTGGTGGGACTCGATTGTCTAG-AT-GTTAGAATGATCATAAA-TTCGTTAAGGATGT-GACGTA--------------------------------------------------------------

>LN881566_Acaulospora_rugosa

CCTAGTAAGCGTGAGTCATCAGCTCACGTTGATTACGTCCCT-GCCCTTTGTACACACCGCCCGTCGCTACTACCGATTGAAT-GGCTTAGTGAGACTCTCGGATCGGGTTTTAGGAACCGGTAACGGATCCTTTCTTCTGAGAAGTTTGTCAAACTTGGTCATTTAGAGGAAGTAAAAGTCGTAACAAGGTTTCCGTAGGTGAACCTGCGGAAGGATCATTAGAAA--------------------------------TTTTA-----------------TTATGTATTC-AAAA--TTTCAATCTATA--AAAAAAT-------TTTTTATT---TATAT----------AAAAT---AAAAAAAAGACAACTTTCAACAACGGATCTCTTGGCTCTTGCATCGATGAAGAACGCAGCGAAATGCGATAGGTAATGTGAATTGCAGAATTCCGTGAATCATCAAATCTTTGAACGCAAATTGCACTCTTT-GGTATTCCGAAGAGTATACTTGCTTGAGGGTTGTTCTA---ATAATATCG--------------------TTTTTTTTAT--AATAAAAAAAAACGGATCTGAG---TTTTCCAAGG-TTTT---------------------------TTATCTTTGGTAACTTT-AAATTTATCTTAACGATATATTAG------TAT-A-TA-TTGG-AAATGT-T--TTAT-CTTACATC------------------TCGTGAG---TTT-CA-TTAATAATTAAT---TACT--AAATCGTTAGAGCGTT--TTATTTGCCTAA-------------------------------TT-TTAT--AA----TT-GGG--T-TACATAA-TACAA-TCTT-----TTT----TTTCAAACCTCAAGTC-AAGTAAG-ATTACCCGCTGAAC-TT-AAGCATATCAATAAGCGGAGGAAACGAAACTAAC-AAGGATTCCC-TTAG-TAAC-GGCGAGTGAAGTGGGAAAAGCTCAAA-TTTTAAATCACCCGGG-TTT--CCTTGGTGAATTGTAATTTGAAGAAAGT-G-TTTTGAC-GTTCGGGTTCGATT-TAAATCCTTTGGGA--TAAGGTATCAT-AG-AGGGTTAGAATCCCGTTTATGATTGTTCCTTT---GAA--TGTCTATAATTCACTTTCCAAGAGTCGAGTTG-TTTGGGATTGCAGCTCAAAAAGGGTGGTAAATTTCACCTAAGGCTAAATATATGCAAGAGACCGATAGCGAACAAGTACCGT-GAGGGAAAGA--TGAAAAGAACTTTGAAAAGAGAGTTAAATAGTACGTGAAATTGTTGAAAGGGAAACGATTGAAGTCAGTCATGTTGGTAGGGAATCAACTTTGATGGTA-----ATGCGGG----------------------------------TTTA-CTCGTGTTCTTTCGAGTGCACTTCCTT--ACTT-ACTAGTTAGCATCAA-TTTTGGTTATCATATAAAAATT-GAGGGAAGGTGGCTTT---TCTTCGGAAAAGTG-TTATAGACCTCGAG-AAATGTGGTGACCGAGATTGAGGATTGCAGCGGATACC-TTTTT----GGGCTAGTCACCTGA-CTTCTGATT-GTTACCCC--ACCCACCTTAGC-AAGCTAACTTGGGTGGGG-TTGATTCTCTAG-TT-GTTGGAGTGATCATAAA-TTCGCTAAGGATGCTGACGTAATGGCTTTAAACGACCCGTCTTGAAACACGGACCAAGGAGTCTAAC-ATGTGTGCGAGTGTT

>KY362433_Acaulospora_fragilissima

-------------------------------ATTACGTCCCT-GCCCTTTGTACACACCGCCCGTCGCTACTACCGATTGAAT-GGCTTAGTGAGACTCTCGGATTGGGCTTTAGGAACCGGCAACGGTCCCTTTTTTCTGAGAAGTTTGTCAAACTTGGTCATTTAGAGGAAGTAAAAGTCGTAACAAGGTTTCCGTAGGTGAACCTGCGGAAGGATCATTAGAAAT-------------------------------TTTT------------------TTATGTATTC-AAAA--TTTCAATCTTTAT-AAAAAAGTT--TTTTATTTATA---TATAT----------AAAAT----AATAAAAGACAACTTTCAACAACGGATCTCTTGGCTCTTGCATCGATGAAGAACGCAGCGAAATGCGATAAGTAATGTGAATTGCAGAATTCCGTGAATCATCAAATCTTTGAACGCAAATTGCACTCTTT-GGTATTCCGAAGAGTATGCTTGCTTGAGGGTTGTTATAA--ACAATATCG--------------------TTTTTTT-----ATAATAAAAAAACGGATCTGAG---TGTTCCAAGG-TTTT--TTA-----------------------AAACTTTGGTAACTTT-AAATTTATCTTAACGATATTTTGG------TATAA-TA-TTAG-AAATGT-T--TTAT-CTTACATC------------------TCGTAAG---TTT-CA-TTAATAATTAAT---TACC---GATCGTTATAGCGTA--TTATTTACCTAAATT---------------------------TTT-TTAT--AA----TT-GGG--T-TATATAA-TACAATTTTT-----TTT----TTTCAAACCTCAAGTC-AAGTAAG-ATTACCCGCTGAAC-TT-AAGCATATCAATAAGCGGAGGAAAAGAAACTAAC-AAGGATT-CC-TTAG-TAAC-GGCGAGTGAACTGGGAAGAGCTCAAA-TTTTAAATCACCTGGG-TT---TCTTGGTGAATTGTAATTTGAAGAAAGT-G-TTTTGAC-GTTCGGGTTCGATT-TAAATCCTTTGGGA--TAAGGTATCAT-AG-AGGGTGAGAATCCCGTTTATGATTGTTCCTTT---GAA--TGTCTATAATTCACTTTCTAAGAGTCGAGTTG-TTTGGGATTGCAGCTCAAAATGGGTGGTAAATTTCACCTAAGGCTAAATATGTGCAAGAGACCGATAGCGAACAAGTACCGT-GAGGGAAAGA--TGAAAAGAACTTTGAAAAGAGAGTTAAATAGTACGTGAAATTGTTGAAAGGGAAACGATTAAAGTCAGTCATGTTAGTAGGGAATCAACTTGTTGGTA-------TGCGAG----------------------------------TTTT-CTTGTATCCTTTCAAGTGCACTTCCTT--ACTT-ACAGGTTAGCATCAA-TTTTGGTTATCATAAAAAAATT-GAGGTAAGGTGGCTTC---TCTTCGGAGAAGTG-TTATAGACCTTGAT-AAATGTGATGACTGGGATTGAGGATTGCAGCGGATACC-CTTTT--AAGGGCTAGTCACCTGG-CTTCTGATG-GTTACCCC--GTCTTCGATAGC-TTGCTAACGTTGGTGGGACTCGATTATCTAG-AT-GTTAGAGTGATCATAAA-TTCGCTAAGGATGCTGACGTAATGGCTTTAAACGACCCGTCTTGAAACACGGACCAAGGA-----------------------

>KY362432_Acaulospora_fragilissima

-------------------------------ATTACGTCCCT-GCCCTTTGTACACACCGCCCGTCGCTACTACCGATTGAAT-GGCTTAGTGAGACTCTCGGATTGGGCTTTAGGAACCGGTAACGGTCCCTTTTTTCTGAGAAGTTTGTCAAACTTGGTCATTTAGAGGAAGTAAAAGTCGTAACAAGGTTTCCGTAGGTGAACCTGCGGAAGGATCATTAGAAAT-------------------------------TTTT------------------TTATGTATTC-AAAG--TTTCAATCTTTAT-AAAAAAGTT-----TTTATTTA---TATAT----------AAAAT----AATAAAAGACAACTTTCAACAACGGATCTCTTGGCTCTTGCATCGATGAAGAACGCAGCGAAATGCGATAAGTAATGTGAATTGCAGAATTCCGTGAATCATCAAATCTTTGAACGCAAATTGCACTCTTT-GGTATTCCGAAGAGTATGCTTGCTTGAGGGTTGTTATAA--ACAATATCG--------------------TTTTTTT-----ATAATAAAAAAACGGATCCGAG---TGTTCCAAGG-TTTT--TTA-----------------------AAACTTTGGTAACTTT-AAATTTATCTTAACGATATTTTGG------TATAA-TA-TTAG-AAATGT-T--TTAT-CTTACATC------------------TCGTAAG---TTT-CA-TTAATAATTAAT---TACC---GATCGTTATAGCGTA--TTATTTACCTAATTT---------------------------TTT-TTAT--AA----TT-GGG--T-TATATAA-TACAA-TTTT-----TTT----TTTCAAACCTCAAGTC-AAGTAAG-ATTACCCGCTGAAC-TT-AAGCATATCAATAAGCGGAGGAAAAGAAACTAAC-AAGGATT-CC-TTAG-TAAC-GGCGAGTGAACTGGGAAGAGCTCAAA-TTTTAAATCACTGGG--TTT--CCTTGGTGAATTGTAATTTGAAGAAAGT-G-TTTTGAC-GTTCGGGTTCGATT-TAAATCCTTTGGGA--TAAGGTATCAT-AG-AGGGTGAGAATCCCGTTTATGATTGTTCCTTT---GAA--TGTCTATAATTCACTTTCTAAGAGTCGAGTTG-TTTGGGATTGCAGCTCAAAATGGGTGGTAAATTTCACCTAAGGCTAAATATGTGCAAGAGACCGATAGCGAACAAGTACCGT-GAGGGAAAGA--TGAAAAGAACTTTGAAAAGAGAGTTAAATAGTACGTGAAATTGTTGAAAGGGAAACGATTAAAGTCAGTCATGTTAGTAGGGAATCAACTTGTTGGTA-------TGCGAG----------------------------------TTTC-CTTGTGTCCTTTCAAGTGCACTTCCTT--ACTT-ACAGGTTAGCATCAA-TTTTGGTTATCATAAAAAAATC-GAGGTAAGGTGGCTTT---TCTTCGGAAAAGTG-TTATAAACCTTGAT-AAATGTGATGACCGGGATTGAGGATTGCAGCGGATACC-CTTT---AAGGGCTAGTCACCTGG-CTTCTGATG-GTTACCCC--GTCTTCGATAGC-TTGCTAACGTTGTTGGGACTCGATTATCTAG-AT-GTTAGAGTGATCATAAA-TTCGCTAAGGATGCTGACGTAATGGCTTTAAACGACCCGTCTTGAAACACGGACCAAGGA-----------------------

>AJ242500_Acaulospora_morrowiae

------------------------------------------------------------------------------------------------------------------------------------------------------------------------------------------------------------------------------AGAAA--------------------------------TTTT------------------TTATGTATTC-AAAA--TTTCAATTTTTAT---AAAGTTT--TTATTTTTATT---TATAT----------AAAAT-----AAAAAATACAACTTTCAACAACGGATCTCTTGGCTCTTGCATCGATGAAGAACGCAGCGAAATACGAAAAGTAATGTGAATTGCAGAATTCCGTGAATCATCAAATCTTTGAACGCAAATTGCACTCTTT-GGTATTCCGAAGAGTATGCTTGCTTGAGGGTTGTTTCA---ATAATATCG--------------------TTTTTTAT-----AATAAAAAAAACGGAACTGAG---TTTTCCAAAG-------TTA-----------------------TAACTTTGGTAACTTT-AAATTTAACTTAACGATATTAAGT------AT--C-TA-TTGG-AAATGT-T--TTAT-CTTACATC------------------TCGTGAG---TTT-CG-TTAACAATTATT---TACT--AAATCGTTAGAGCGTA--TTATTTGCCTAA------------------------------TTT-TTAT--AA----TT--GG--T-TATATAA-TACAA-TTTT-----TTT----TTCAAAC-----------------------------------------------------------------------------------------------------------------------------------------------------------------------------------------------------------------------------------------------------------------------------------------------------------------------------------------------------------------------------------------------------------------------------------------------------------------------------------------------------------------------------------------------------------------------------------------------------------------------------------------------------------------------------------------------------------------------------------------------------------------------------------------------------------------------------------------------------------------------------------------------------------------------------------

>KY362428_Acaulospora_saccata

-------------------------------ATTACGTCCCT-GCCCTTTGTACACACCGCCCGTCGCTACTATCGATTGAAT-GGCTTAGTGAGACTCTCGGATCGGGTTTTAGGAACCGGTAACGGATCCTTTTTTCTGAGAAGTTTGTCAAACTTGGTCATTTAGAGGAAGTAAAAGTCGTAACAAGGTTTCCGTAGGTGAACCTGCGGAAGGATCATTAGAAA--------------------------------TT--------------------TTTTGTATTC-AAAA--TTTCAATCTTTAT---AAGTT-------TTTTTATT---TATAT----------AAAAT----AAAAAAAGACAACTTTCAACAACGGATCTCTTGGCTCTTGCATCGATGAAGAACGCAGCGAAATGCGATAAGTAATGTGAATTGCAGAATTCCGTGAATCATCAAATCTTTGAACGCAAATTGCACTCTTT-GGTATTCCGAAGAGTATGCTTGCTTGAGGGTTGTTCTA---ACAATATCG--------------------TTTTTTTTAT--AATAAAAAAAAACGGAACTGAG---TTTTTCTAAA-GTTT--TTA-----------------------TAACTTTAGTAACTTT-AAATTTAACTTAACGATAGA--AG------TAT-C-TA-TTGG-AAATGT-T--TTAT-CTTACATC------------------TCGTGAG---TTT-CA-TTGACAATTATT---TACT--AAATCGTTAGAGCGTA--TTATTTACCTAA------------------------------TTT-TTAT--AA----TT-GGGATA-TATATAA-TTCAA-TATT-----TTT----TT--CAACCTC-AGTC-AAGTAAG-ATTACCCGCTGAAC-TT-AAGCATATCA--TAGCGGAGGAAAAG-----AAC-TACAGGATCC-TTAG-TAAC-GGCGAGTGAAGTGGGAAAGCTCAA-----TTTAATCACCCGGG-TTC--CCTTGGTGAATTGTAA-TTGAAGAAAGT-G-TTTTGAC-GTTCGGGTTCGATT-TAAATCC-TTGGGA--TAAGGTATCAT-AG-AGGGTTAGAATCCCGTTTATGATTGATCCTTT---GAA--TGTCTATAATTCACTTTCCAAGAGTCGAGTTG-TTTGGGATTGCAGCTCAAAATGGGTGGTAAATTTCACCTAAGGCTAAATATGTGCAAGAGACCGATAGCGAACAAGTACCGT-GAGGGAAAGA--TGAAAAGAACTTTGAAAAGAGAGTTAAATAGTACGTGAAATTGTTGAAAGGGAAACGATTAAAGTCAGTCATGTTGGTAGGGAATCAACTTGATAGTA-------TGCGGG----------------------------------TTTA-CTCGTGTCCTTTCTGGTGCACTTCCTT--ACTT-GCAGGTTAGCATCAA-TTTCGGTTATCATATAAAAATT-GAGAGAAGGTGGCTTT---TCTTCGGAAAAGTG-TTATAGACCTCGAT-AAATGTGGCGATCGGGATTGAGGATTGCAGCGGATACC-CTTT-----GGGCTAGTCACCTGG-CATCTGATG-GTTATCCC--ATCGACGTTAGC-AAGCTAACGTTGATGGGACTCGATTATCTTG-AT-GTTAGATTGATCATAAA-TTCGCTAAGGATGCTGACGTAATGGCTTTAAACGACCCGTCTTGAAACACGGACCAAGGA-----------------------

>KY362429_Acaulospora_saccata

-------------------------------ATTACGTCCCT-GCCCTTTGTACACACCGCCCGTCGCTACTATCGATTGAAT-GGCTTAGTGAGACTCTCGGATCGGGTTTTAGGAACCGGTAACGGATCCTTTTTTCTGAGAAGTTTGTCAAACTTGGTCATTTAGAGGAAGTAAAAGTCGTAACAAGGTTTCCGTAGGTGAACCTGCGGAAGGATCATTAGAAA--------------------------------TT--------------------TTTTGTATTC-AAAA--TTTCAATCTTTAT---AAGTT-------TTTTTATT---TATAT----------AAAAT----AAAAAAAGACAACTTTCAACAACGGATCTCTTGGCTCTTGCATCGATGAAGAACGCAGCGAAATGCGATAAGTAATGTGAATTGCAGAATTCCGTGAATCATCAAATCTTTGAACGCAAATTGCACTCTTT-GGTATTCCGAAGAGTATGCTTGCTTGAGGGTTGTTCTA---ACAATATCG--------------------TTTTTTTTAT--AATAAAAAAAAACGGAACTGAG---TTTTTCTAAA-GTTT--TTA-----------------------TAACTTTAGTAACTTT-AAATTTAACTTAACGATAGA--AG------TAT-C-TA-TTGG-AAATGT-T--TTAT-CTTACATC------------------TCGTGAG---TTT-CA-TTGACAATTATT---TACT--AAATCGTTAGAGCGTA--TTATTTACCTAA------------------------------TTT-TTAT--AA----TT-GGGATA-TATATAA-TTCAA-TATT-----TTT----TT--CAACCTC-AGTC-AAGTAAG-ATTACCCGCTGAAC-TT-AAGCATATCA--TAGCGGAGGAAAAG-----AAC-TACAGGATCC-TTAG-TAAC-GGCGAGTGAAGTGGGAAAGCTCAA-----TTTAATCACCCGGG-TTC--CCTTGGTGAATTGTAA-TTGAAGAAAGT-G-TTTTGAC-GTTCGGGTTCGATT-TAAATCC-TTGGGA--TAAGGTATCAT-AG-AGGGTTAGAATCCCGTTTATGATTGATCCTTT---GAA--TGTCTATAATTCACTTTCCAAGAGTCGAGTTG-TTTGGGATTGCAGCTCAAAATGGGTGGTAAATTTCACCTAAGGCTAAATATGTGCAAGAGACCGATAGCGAACAAGTACCGT-GAGGGAAAGA--TGAAAAGAACTTTGAAAAGAGAGTTAAATAGTACGTGAAATTGTTGAAAGGGAAACGATTAAAGTCAGTCATGTTGGTAGGGAATCAACTTGATAGTA-------TGCGGG----------------------------------TTTA-CTCGTGTCCTTTCTGGTGCACTTCCTT--ACTT-GCAGGTTAGCATCAA-TTTCGGTTATCATATAAAAATT-GAGAGAAGGTGGCTTT---TCTTCGGAAAAGTG-TTATAGACCTCGAT-AAATGTGGCGATCGGGATTGAGGATTGCAGCGGATACC-CTTT-----GGGCTAGTCACCTGG-CATCTGATG-GTTATCCC--ATCGACGTTAGC-AAGCTAACGTTGATGGGACTCGATTATCTTG-AT-GTTAGATTGATCATAAA-TTCGCTAAGGATGCTGACGTAATGGCTTTAAACGACCCGTCTTGAAACACGGACCAAGGA-----------------------

>LN810999_Acaulospora_baetica

CCTAGTAAGCGTGAGTCATCAGCTCACGTTGATTACGTCCCT-GCCCTTTGTACACACCGCCCGTCGCTACTACCGATTGAAT-GGCTTAGTGAGACTCTCGGATCGGGTTTTTGGAACCGGCAACGGTCCCTTTCATCTGAGAAGTTTGTCAAACTTGGTCATTTAGAGGAAGTAAAAGTCGTAACAAGGTTTCCGTAGGTGAACCTGCGGAAGGATCATTAGAAAT-------------------------------TTTT-------------------TATGTATTC-AAAAAATTTTCAATCTTTT---T-AT-CC--AAATTTAT------TATAAG-ATG-------TATATAATTCATGAGATAACTTTCAACAACGGATCTCTTGGCTCTTGCATCGATGAAGAACGCAGCGAAATGCGATAAGTAATGTGAATTGCAGAATTCCGTGAATCATCAAATCTTTGAACGCAAATTGCACTCCTT-GGTACTCCGAGGAGTATGCTTGTTTGAGGGTTGTTCCA---ATAAATTCG--------------------ATTTTTTTT---------AAAAAACGGATCTGAG---TTTTCCAAGG---TT--TTA-----------------------CGATTTTGGTGACTTT-AAATTTATCCTAACGATATCTTTGG-----TATGA-TA-TTGG-AAATGG-TCATATT-CCTACA------------------TATTGTGAG---TTT-CA-TTGACAATCGAT---TACC---TGTCGTTACGTCGTA--TCATTTGCCTAA------------------------------TCT-TTTT--AA----TT-GGG--T-TACATGA-TACAA-ATTT-----TCT----TTCAACACCTCAAGTC-AAGTAAG-AATACCCGCTGAAC-TT-AAGCATATCAATAAGCGGAGGAAAAGAAACTAAC-AAGGATTCCC-TTAG-TAAC-GGCGAGTGAAGTGGGAAAAGCTCAAA-TTTTAAATCTCCGAGG-TTT--CCTCGGCGAGTTGTAATTTGAAGAGAGT-G-TTTGGAC-ATTCAGGTTTAATC-CAAATCCTTTGGGA--TAAGGTATCAT-AG-AGGGTGAGAATCCCGTTTATGATTAGATCTCG---TGT--GTCACTTAACTCACTTTCCAAGAGTCGAGTTG-TTTGGGATTGCAGCTCAAAACGGGTGGTAAATTTCACCTAGGGCTAAATATATGCAAGAGACCGATAGCGAACAAGTACCGT-GAGGGAAAGA--TGAAAAGAACTTTGAAAAGAGAGTTAAATAGTACGTGAAATTGTTGAAAGGGAAACGATTGAAGTCAGTCATGCTAGTGGGGAATCAACTTGATGGTG-------TGTGGG----------------------------------TTCT-CTCGCACACTTTCCGGTGCACTTCCTC--ACTTGGCAGGTTAGCATCAA-TTTCGGTTATCATAAAAAATTT-GAGGGAATGTGGCTTT---CCTTCGGGAAAGTG-TTAAAGACCTCGAT-CTATGTGGTGACCGGGATTGAGGATTGCAGCGGATACC-CTCGT-----GGCTTATTACCTGG-CTTCTGACG-GTTACCCC--GCTTTCGACAGC-TTGCTGACGTTGGTGGGACTCGATTGTCTAG-AT-GTCAGAGTGATTCTAT--TTCGCTAAGGATGCTGACGTAATGGCTTTAAACGACCCGTCTTGAAACACGGACCAAGGAGTCTAAC-ATATGTGCGAGTGTT

>LN811001_Acaulospora_baetica

CCTAGTAAGCGTGAGTCATCAGCTCACGTTGATTACGTCCCTCGCCCTTTGTACACACCGCCCGTCGCTACTACCGATTGAAT-GGCTTAGTGAGACTCTCGGATCGGGTTTTTGGAACCGGCAACGGTCCCTTTCATCTGAGAAGTTTGTCAAACTTGGTCATTTAGAGGAAGTAAAAGTCGTAACAAGGTTTCCGTAGGTGAACCTGCGGAAGGATCATTAGAAA--------------------------------TTTT-------------------TATGTATTC--AAAAATTTTCAATCTTTT---T-AT-CC--AAATTTAT------TATAAG-ATG-------TATATAATTCATGAGATAACTTTCAACAACGGATCTCTTGGCTCTTGCATCGATGAAGAACGCAGCGAAATGCGATAAGTAATGTGAATTGCAGAATTCCGTGAATCATCAAATCTTTGAACGCAAATTGCACTCCTT-GGTATTCCGAGGAGTATGCTTGTTTGAGGGTTGTTCCA---ATAAATTCG--------------------ATTTTTTAA---------AAAAAACGGATCTGAG---TTTTCCAAGG---TT--TTA-----------------------CGATTTTGGTAACTTG-AAATTTATCTTAACGATATCTTTGG-----TATGA-TA-TTGG-AAATGG-TCATATT-CTTACA------------------TATTGTGAG---TTT-CA-TTGACAATCGAT---TACC---TGTCGTTACGTCGTA--TCATTTGCCTAA------------------------------TCT-TTTT--AA----TT-GGG--C-TACATGA-TACAA-ATTT-----TCT----TTCAACACCTCAAGTC-AAGTAAG-AATACCCGCTGAAC-TT-AAGCATATCAATAAGCGGAGGAAAAGAAACTAAC-AAGGATTCCC-TTAG-TAAC-GGCGAGTGAAGTGGGAAAAGCTCAAA-TTTTAAATCTCCGAGG-TTT--CCTTGGCGAGTTGTAATTTGAAGAGAGT-G-TTTGGAC-ATTCAGGTTTAATC-CAAATCCTTTGGGA--TAAGGTATCAT-AG-AGGGTGAGAATCCCGTTTATGATTAGATCTCG---TGT--GTCACTTAATTCACTTTCCAAGAGTCGAGTTG-TTTGGGATTGCAGCTCAAAACGGGTGGTAAATTTCACCTAAGGCTAAATATATGCAAGAGACCGATAGCGAACAAGTACCGT-GAGGGAAAGA--TGAAAAGAACTTTGAAAAGAGAGTTAAATAGTACGTGAAATTGTTGAAAGGGAAACGATTGAAGTCAGTCATGCTAGTGGGGAATCAACTTGATGGTG-------TGTGGG----------------------------------TTCT-CTCGCACACTTTCCGGTGCACTTCCTC--ACTTGGCAGGTTAGCATCAA-TTTCGGTTATCATAAAAAATTT-GAGGGAATGTGGCTTT---CCTTCGGGAAAGTG-TTATAGACCTCGAT-CTATGTGGTGACCGGGATTGAGGATTGCAGCGGATACC-TTCGT-----GGCTTGTCACCTGG-CTTCTGACG-GTTGCCCC--GCTTTCGACAGC-TTGCTGACGTTAGTGGGATTCGATTGTCTAG-AT-GTTTGAGTGATTCTAT--TTCGCTAAGGATGCTGACGTAATGGCTTTAAACGACCCGTCTTGAAACACGGACCAAGGAGTCTAAC-ATATATGCGAGTGTT

>KP191471_Acaulospora_ignota

CCTAGTAAGCGTGAGTCATCAGCTCACGTTGATTACGTCCCT-GCCCTTTGTACACACCGCCCGTCGCTACTACCGATTGAAT-GGCTTAGTGAGACTCTCGGATCGGGTTTTTGGAACCGGCAACGGTCCCTTTCATCTGAGAAGTTTGTCAAACTTGGTCATTTAGAGGAAGTAAAAGTCGTAACAAGGTTTCCGTAGGTGAACCTGCGGAAGGATCATTAGAAA--------------------------------TTTT-------------------TATGTATTC-AAAAAATTTTCAATCTTTC---T-AT-TCC-AAATTTAT------TATAAT--TG-------TATATAATTCATGAGAAAACTTTCAACAACGGATCTCTTGGCTCTTGCATCGATGAAGAACGCAGCGAAATGCGATAAGTAATGTGAATTGCAGAATTCCGTGAATCATCAAATCTTTGAACGCAAATTGCACTCCTT-GGTATTCCGAGGAGTATGCTTGATTGAGGGTTGTTCCA---ATAAATTCG--------------------TTTTTTTTA-------AAAAAGAGCGGAACTGAG---TTTTCCAAGG---TT--TTA-----------------------CGATTTTGGTAACTTT-AAATTTATCTTAACGATATCTTTGG-----TATGA-TA-TTGA-AAATGG-TCATATT-CTTACA------------------TATTGTGAG---TTT-CA-TTGACAATCGAT---TACC---TGTCGTTACGTCGTA--TCATTTATCTAAT-----------------------------TCT-TTTT--AA----TT-GGG--T-TACATGA-TACAA--ATT-----TCT----TTCAACACCTCAAGTC-AAGTAAG-AATACCCGCTGAAC-TT-AAGCATATCAATAAGCGGAGGAAAAGAAACTAAC-AAGGATTCCC-TTAG-TAAC-GGCGAGTGAAGTGGGAAAAGCTCAAA-TTTTAAATCTCCGAGG-TTC--CCTCGGCGAGTTGTAATTTGAAGAGAGT-G-TTTGGAC-ATTCAGGTTTAATC-CAAATCCTTTGGGA--TAAGGTATCAT-AG-AGGGTGAGAATCCCGTTTATGATTAGATCTCG---TGT--GTCACTTAATTCACTTTCCAAGAGTCGAGTTG-TTTGGGATTGCAGCTCAAAACGGGTGGTAAATTTCACCTAAGGCTAAATATGTGCAAGAGACCGATAGCGAACAAGTACCGT-GAGGGAAAGA--TGAAAAGAACTTTGAAAAGAGAGTTAAATAGTACGTGAAATTGTTGAAAGGGAAACGATTGAAGTCAGTCATGCTAGTGGGGAATCAACTTGATGGTG-------TGTGGG----------------------------------TTCT-CTCGCATACTTTCCGGTGCACTTCCTC--ACTCGGCAGGTTAGCATCAA-TTTCGGTTATCATAAAAAATTT-GAGGGAATGTGGCTTT---CCTTCGGGAAAGTG-TTATAGACCTCGAT-CTATGTGGTGACCGGGATTGAGGATTGCAGCGGATACC-CTCGT-----GGCTTATTACCTGG-CTTCTGGCG-GTTACCCC--GCTTTCGACAGC-TTGCTGACGTTAGTGGGACTCGATTGTCTAG-AT-GTCAGAGTGATTTTAT--TTCGCTAAGGATGCTGACGTAATGGCTTTAAACGACCCGTCTTGAAACACGGACCAAGGAGTCTAAC-ATATGTGCGAGTGTT

>KP191472_Acaulospora_ignota

CCTAGTAAGCGTGAGTCATCAGCTCACGTTGATTACGTCCCT-GCCCTTTGTACACACCGCCCGTCGCTACTACCGATTGAAT-GGCTTAGTGAGACTCTCGGATCGGGTTTTTGGAACCGGCAACGGTCCCTTTCATCTGAGAAGTTTGTCAAACTTGGTCATTTAGAGGAAGTAAAAGTCGTAACAAGGTTTCCGTAGGTAAACCTGCGGAAGGATCATTAGAAA--------------------------------TTTT-------------------TATGTATTC-AAAAAATTTTCAATCTTTC---T-AT-TCC-AAATTTAT------TATAATTGTA-------TATATAATTCATGAGAAAACTTTCAACAACGGATCTCTTGGCTCTTGCATCGATGAAGAACGCAGCGAAATGCGATAAGTAATGTGAATTGCAGAATTCCGTGAATCATCAAATCTTTGAACGCAAATTGCACTCCTT-GGTATTCCGAGGAGTATGCTTGATTGAGGGTTGTTCCA---ATAAATTCG--------------------TTTTTTTTA-------AAAAAGAGCGGAACTGAG---TTTTCCAAGG---TT--TTA-----------------------CGATTTTGGTAACTTTAAAATTTATCTTAACGATATCTTTGG-----TATGA-TA-TTGA-AAATGG-TCATATT-CTTACA------------------TATTGTGAG---TTT-CA-TTGACAATCGAT---TACC---TGTCGTTACGTCGTA--TCATTTATCTAAT-----------------------------TCT-TTTT--AA----TT-GGG--T-TACATGA-TACAA--TTT-----TCT----TTCAACACCTCAAGTC-AAGTAAG-AATACCCGCTGAAC-TT-AAGCATATCAATAAGCGGAGGAAAAGAAACTAAC-AAGGATTCCC-TTAG-TAAC-GGCGAGTGAAGTGGGAAAAGCTCAAA-TTTTAAATCTCCGAGG-TTC--CCTCGGCGAGTTGTAATTTGAAGAGAGT-G-TTTGGAC-ATTCAGGTTTAATC-CAAATCCTTTGGGA--TAAGGTATCAT-AGAAGGGTGAGAATCCCGTTTATGATTAGATCTCG---TGT--GTCACTTAATTCACTTTCCAAGAGTCGAGTTGTTTTGGGATTGCAGCTCAAAACGGGTGGTAAATTTCACCTAAGGCTAAATATGTGCAAGAGACCGATAGCGAACAAGTACCGT-GAGGGAAAGA--TGAAAAGAACTTTGAAAAGAGAGTTAAATAGTACGTGAAATTGTTGAAAGGGAAACGATTGAAGTCAGTCATGCTAGTGGGGAATCAACTTGATGGTG-------TGTGGG----------------------------------TTCT-CTCGCATACTTTCCGGTGCACTTCCTC--ACTCGGCAAGTTAGCATCAA-TTTCGGTTATCATAAAAAATTT-GAGGGAATGTGGCTTT---CCTTCGGGAAAGTG-TTATAGACCTCGAT-CTATGTGGTGACCGGGATTGAGGATTGCAGCGGATACC-CTCGT-----GGCTTATTACCTGG-CTTCTGGCG-GTTACCCC--GCTTTCGACAGC-TTGCTGACGTTAGTGGGACTCGATTGTCTAG-AT-GTCAGAGTGATTTTAT--TTCGCTAAGGATGCTGACGTAATGGCTTTAAACGACCCGTCTTGAAACACGGACCAAGGAGTCTAAC-ATATGTGCGAGTGTT

>HE603641_Acaulospora_nivalis

CCTAGTAAGCGTGAGTCATCAGCTCACGTTGATTACGTCCCT-GCCCTTTGTACACACCGCCCGTCGCTACTACCGATTGAAT-GGCTTAGTGAGACTCTCGGATCGGGTTTTTGGAACCGGCAACGGTCCCTTTCATCTGAGAAGTTTGTCAAACTTGGTCATTTAGAGGAAGTAAAAGTCGTAACAAGGTTTCCGTAGGTGAACCTGCGGAAGGATCATTAGGAA--------------------------------TTTT-------------------TATGTAATC-AAAAAATTTTCAATCTTTC---T-AT-CC--AAA-AAAA------TAAGAT-AAA-------TGTATAATTCATGAGAAAACTTTCAACAACGGATCTCTTGGCTCTTGCATCGATGAAGAACGCAGCGAAATGCGATAAGTAATGTGAATTGCAGAATTCCGTGAGTCATCAAATCTTTGAACGCAAATTGCACTCCTT-GGTATTCCGAGGAGTATGCTTGTTTGAGGGTTGTTCCA---ATAAAATCGT-------------------TTTTTTT-----------AAAAGACGGATCTGAG---TTTTCC--------------------------------------GATTTTGGTAACTTT-AAATTTATCTTAACGATATCTTTGG-----TATGA-TA-TTGG-AAATGG-TTATATT-CTTACA------------------TATTGTGAG---TTT-CA-TTAACAATCGAT---TACC---TGTCGTTACGTCGTA--TCATTTGCCTGA------------------------------TCC-TTTC--GA----TT-GGG--T-TCTATGA-TACAA-ATTT-----TCT----TTCAACACCTCAAGTC-AAGTAAG-AATACCCGCTGAAC-CT-AAGCATATCAATAAGCGGAGGAAAAGAAACTAAC-AAGGATTCCC-TCAG-TAAT-GGCGAGTGAAGTGGGAAAAGCTCAAA-TTTTAAATCTCCGAGG-TCT--CCTTGGCGAGTTGTAATATGAAGAGAGT-G-TTTTGAC-ATTCAGGTTTAATC-CAAATCCTTTGGGA--TAAGGTATCAT-AG-AGGGTGAGAATCCCGTTTATGATAGAATCTTG---GGT--GTCACCTAGTTCACTTTCCAAGAGTCGAGTTG-TTTGGGATTGCAGCTCAAAACGGGTGGTAAATTTCACCTAAGGCTAAATATGTGCAAGAGACCGATAGCGAACAAGTACCGT-GAGGGAAAGA--TGAAAAGAACTTTGAAAAGAGAGTTAAATAGTACGTGAAATTGTTGAAAGGGAAACGATTGAAGTCAGTCATGCTCGTGAGGAATCAACTTGATGGTG-------TGTGGG----------------------------------TTTT-CTCGCATACTTTCCGGTGCACTTCCTT--ACTCGGCAGGTTAGCATCAA-TTTCGGTTATCATAAAAAATTT-GAGGGAACGTGGCTTT---CCTTCGGGAAAGTG-TTATAGACCTCGAT-CTATGTGGTGACCGGGATTGAGGATTGCAGCGGATGCC-CTCGT-----GGCTTGTCACCTGG-CTTCTGACG-GTTACCCC--GCTTTCGACAGC-TTGCTAACGTTAGTGGGACTCGATTGCCTAG-AT-GTCAGAGTGATTCTAT--TTCGCTAAGGATGCTGACGTAATGGCTTTAAACGACCCGTCTTGAAACACGGACCAAGGAGTCTAAC-ATGTGTGCGAGTGTT

>HE603644_Acaulospora_nivalis

CCTAGTAAGCGTGAGTCATCAGCTCACGTTGATTACGTCCCT-GCCCTTTGTACACACCGCCCGTCGCTACTACCGATTGAAT-GGCTTAGTGAGACTCTCGGATCGGGTTTTTGGAACCGGCAACGGTCCCTTTCATCTGAGAAGTTTGTCAAACTTGGTCATTTAGAGGAAGTAAAAGTCGTAACAAGGTTTCCGTAGGTGAACCTGCGGAAGGATCATTAGAAA--------------------------------TTTT-------------------TATGTATTC-AAAAATTTTCCAATCTTTC---T-AT-CT--AA--AAAA------TATTAT-AAA-------TGTATAAATCATGAGATAACTTTCAACAACGGATCTCTTGGCTCTTGCATCGATGAAGAACGCAGCGAAATGCGATAAGTAATGTGAATTGCAGAATTCCGTGAATCATCAAATCTTTGAACGCAAATTGCACTCCTT-GGTATTCCGAGGAGTATGCTTGTTTGAGGGTTGTTCCA---ATAAAATCG--------------------TTTCTTT-----------AAAAGACGGATCTGAG---TTTTCTGAGA---TT--TTA-----------------------CGATTTTGGTAACTTT-AAATTTATCTTAACGATATCTTTGG-----TATGA-TA-TTGG-AAATGA-TTATATT-CTTACA------------------TATTGTGAG---TTT-CA-TTAACAATCGAT---TACC---TGTCGTTACGTCGTA--TTATTTACCTAA------------------------------TTC-TTTT--GA----TT-GGG--T-TCTATGA-TACAA-ATTT-----TCT----TTCAACACCTCAAGTC-AAGTAAG-AATACCCGCTGAAC-TT-AAGCATATCAATAAGCGGAGGAAAAGAAACTAAC-AAGGATTCCC-TTAG-TAAC-GGCGAGTGAAGTGGGAAAAGCTCAAA-TTTTAAATCTCCGAGG-TTT--CCTTGGCGAGTTGTAATTTGAAGAGAGT-G-TTTTGAC-ATTCAGGTTTAATC-CAAATCCTTTGGGA--TAAGGTATCAT-AG-AGGGTGAGAATCCCGTTTATGATTAAATCTTG---GGT--GTCACTTAATTCACTTTCCAAGAGTCGAGTTG-TTTGGGATTGCAGCTCAAAACGGGTGGTAAATTTCACCTAAGGCTAAATATGTGCAAGAGACCGATAGCGAACAAGTACCGT-GAGGGAAAGA--TGAAAAGAACTTTGAAAAGAGAGTTAAATAGTACGTGGAATTGTTGAAAGGGAAACGATTGAAGTCAGTCATGCTCGTGAGGAATCAACTTGATGGTG-------TGTGGG----------------------------------TTCT-CTCGCATACTTTCCGGTGCACTTCCTT--ACTCGGCAGGTTAGCATCAA-TTTCGGTTATCATAAAAAATTT-GAGGGAACGTGGCTTT---CCTTCGGGAAAGTG-TTATAGACCTTGAT-CTATGTGGTGACCGGGATTGAGGATTGCAGCGGATGCC-CTCGT-----GGCTTGTTACCTGG-CTTCTGACG-GTTACCTC--GCTTTCGACAGC-TTGCTAACGTTAGTGGGACTCGATTGTCTAG-AT-GTCAGAGTGATTTTAT--TTCGCTAAGGATGCTGACGTAATGGCTTTAAACGACCCGTCTTGAAACACGGACCAAGGAGTCTAAC-ATGTGTGCGAGTGTT

>FM876789_Acaulospora_cavernata

CCTAGTAAGCGTGAGTCATCAGCTCACGTTGATTACGTCCCT-GCCCTTTGTACACACCGCCCGTCGCTACTACCGATTGAAT-GGCTTAGTGAGACTCTCGGATCGGGTTTTTGGAACCGGCAACGGTCCCTTTCATCTGAGAAGTTTGTCAAACTTGGTCATTTAGAGGAAGTAAAAGTCGTAACAAGGTTTCCGTAGGTGAACCTGCGGAAGGATCATTAGAAA--------------------------------TTTT-------------------TATGTATTC-AAAAAATTTTAAATCTTTT---T-TT-TT--A---ATTT------AAAAAA-TTG-------AAATAAATTATTAAGATAACTTTCAACAACGGATCTCTTGGCTCTTGCATCGATGAAGAACGCAGCGAAATGCGATAAGTAATGTGAATTGCAGAATTCCGTGAATCATCAAATCTTTGAACGCAAATTGCACTCTTT-GGTATTCCGATGAGTATGCTTGTTTGAGGGTTGTTCCA---ATCAAATCG--------------------TTTTTTT-----------AAAAAACGGATCTTAG---TTTTCCAAGG---TT--TTA-----------------------TTATTTTGGTAACTTT-AAATTTATCTTAACGATATCTTTGG-----TATGA-TA-TTGG-AAATGG-TTATATT-CTTACATC---------------TTCTTGTGAG---TTT-CA-TTAACAATTGAT---TACC---TGTCGTTACGTCGTA--TTATTTGCCTGA------------------------------TCC-TTTT--AA----TT-GGG--T-TACATAA-TACAA-TTTT-----TCT----TTCAACACCTCAAGTC-AAGTAAG-AATACCCGCTGAAC-TT-AAGCATATCAATAAGCGGAGGAAAAGAAACTAAC-AAGGATTCCC-TTAG-TAAC-GGCGAGTGAAGTGGGAAGAGCTCAAA-TTTTAAATCTCCGAGG-TTT--CCTTGGCGAGTTGTAATTTGAAGAGAGT-G-TTTTGAC-ATTCAGGTTTAATT-TAAATCCTTTGGGA--TAAGGTATCAT-AG-AGGGTGAGAATCCCGTTTATGATTAAATCTTG---AGT--GTCACTTAATTCACTTTCCAAGAGTCGAGTTG-TTTGGGATTGCAGCTCAAAACGGGTGGTAAATTTCACCTAAGGCTAAATATATGCAAGAGACCGATAGCGAACAAGTACCGT-GAGGGAAAGA--TGAAAAGAACTTTGAAAAGAGAGTTAAATAGTACGTGAAATTGTTGAAAGGGAAACGATTGAAGTCAGTCATGCTAGTGGGGAATCAACTTGATGGTG-------TGTGGG----------------------------------TTCT-CTCGCATACTTTCCGGTGCACTTCCTT--ACTCGGCAGGTTAGCATCAA-TTTCGGTTATCATCCAAAATTT-GAGGGAATGTGGCTCT---TCTTCGGAAAAGTG-TTATAGACCTTGAT-CTATGTGGTGACCGGGATTGAGGATTGCAGCGGATACC-CTCGT-----GGCTTGTCACCTGG-CTTCTGACG-GTTACCCC--GCTTTCGATAGC-TCGCTAACGTTAGTGGGACTCGATTGTCTAG-AT-GTTAGAGTGATTGATT--TTCGCTAAGGATGCTGACGTAATGGCTTTAAACGACCCGTCTTGAAACACGGACCAAGGAGTCTAAC-ATGTGTGCGAGTGTT

>FM876788_Acaulospora_cavernata

CCTAGTAAGCGTGAGTCATCAGCTCACGTTGATTACGTCCCT-GCCCTTTGTACACACCGCCCGTCGCTACTACCGATTGAAT-GGCTTAGTGAGACTCTCGGATCGGGTTTTTGGAACCGGCAACGGTCCCTTTCATCTGAGAAGTTTGTCAAACTTGGTCATTTAGAGGAAGTAAAAGTCGTAACAAGGTTTCCGTAGGTGAACCTGCGGAAGGATCATTAGAAA--------------------------------TTTT-------------------TATGTATTC-AAAAAATTTTAAATCTTTT---T-TT-TT--A---ATTT------AAAAAA-TTG-------AAATAAATTATTAAGATAACTTTCAACAACGGATCTCTTGGCTCTTGCATCGATGAAGAACGCAGCGAAATGCGATAAGTAATGTGAATTGCAGAATTCCGTGAATCATCAAATCTTTGAACGCAAATTGCACTCTTT-GGTATTCCGAGGAGTATGCTTGTTTGAGGGTTGTTCCA---ATAAAATCG--------------------ATTTTTT-----------AAAAAACGGATCTGAG---TTTTCCAAGG---TT--TTA-----------------------TTATTTTGGTAACTTT-AAATTTATCTTAACGATATCTTTGG-----TATGA-TA-TTGG-AAATGG-TTATATT-CTTACA------------------TCTTGTGAG---TTT-CA-TTAACAATTGAT---TACC---TGTCGTTACGTCGTA--TTATTTGCCTGA------------------------------TCC-TTTT--AA----TT-GGG--T-TACATAA-TACAA-TTTT-----TCT----TTCAACACCTCAAGTC-AAGTAAG-AATACCCGCTGAAC-TT-AAGCATATCAATAAGCGGAGGAAAAGAAACTAAC-AAGGATTCCC-TTAG-TAAC-GGCGAGTGAAGTGGGAAGAGCTCAAA-TTTTAAATCTCCGAGG-TTT--CCTTGGCGAGTTGTAATTTGAAGAGAGT-G-TTTTGAC-ATTCAGGTTTAATT-TAAATCCTTTGGGA--TAAGGTATCAT-AG-AGGGTGAGAATCCCGTTTATGATTAAATCTTG---AGT--GTCACTTAATTCACTTTCCAAGAGTCGAGTTG-TTTGGGATTGCAGCTCAAAACGGGTGGTAAATTTCACCTAAGGCTAAATATATGCAAGAGACCGATAGCGAACAAGTACCGT-GAGGGAAAGA--TGAAAAGAACTTTGAAAAGAGAGTTAAATAGTACGTGAAATTGTTGAAAGGGAAACGATTGAAGTCAGTCATGCTAGTGGGGAATCAACTTGATGGTG-------TGTGGG----------------------------------TTCT-CTCGCATACTTTCCGGTGCACTTCCTT--ACTCGGCAGGTTAGCATCAA-TTTCGGTTATCATCCAAAATTT-GAGGGAATGTGGCTCT---TCTTCGGAAAAGTG-TTATAGACCTTGAT-CTATGTGGTGACCGGGATTGAGGATTGCAGCGGATACC-CTCGT-----GGCTTGTCACCTGG-CTTCTGACG-GTTACCCC--GCTTTCGATAGC-TCGCTAACGTTAGTGGGACTCGATTGTCTAG-AT-GTTAGAGTGAATAATT--TTCGCTAAGGATGCTGACGTAATGGCTTTAAACGACCCGTCTTGAAACACGGACCAAGGAGTCTAAC-ATGTGTGCGAGTGTT

>FR846382_Acaulospora_punctata

CCTAGTAAGCGTGAGTCATCAGCTCACGTTGATTACGTCCCT-GCCCTTTGTACACACCGCCCGTCGCTACTACCGATTGAAT-GGCTTAGTGAGACTCTCGGATCGGGTTTTTGGAACCGGCAACGGTCCCTTTCATCTGAGAAGTTTGTCAAACTTGGTCATTTAGAGGAAGTAAAAGTCGTAACAAGGTTTCCGTAGGTGAACCTGCGGAAGGATCATTAGAAA---------------------------------TTT-------------------TATGTATTC-AAAAATTTTAAATCATTTT---T----TA--A---TTTA------AAAAAA-TCG-------AAATAAATTATTAAGATAACTTTCAACAACGGATCTCTTGGCTCTTGCATCGATGAAGAACGCAGCGAAATGCGATAAGTAATGTGAATTGCAGAATTCCGTGAATCATCAAATCTTTGAACGCAAATTGCACTCTTT-GGTATTCCGAGGAGTATGCTTGTTTGAGGGTTGTTCCA---ACAAAATCG--------------------TTTTTTT-----------AAAAAACGGATCTGAG---TTTTCCAAGG---TT--TTA-----------------------TTATTTTGGTAACTTT-AAATTTATCTTAACGATATCTTTGG----TTATGA-TA-TTGG-AAATGG-TTATATT-CTTACATC---------------ATCTTGTGAG---TTT-CA-TTAACAATTGAT---TACC---TGTCGTTACGTCGTA--TTATCTGCCTAA------------------------------TCCTTTTT--AA----TT-GGG--T-TACATAA-TACAA-ATTT-----TCT----TTCAACACCTCAAGTC-AAGTAAG-AATACCCGCTGAAC-TT-AAGCATATCAATAAGCGGAGGAAAAGAAACTAAC-AAGGATTCCC-TTAG-TAAC-GGCGAGTGAAGTGGGAAGAGCTCAAA-TTTTAAATCTCCGAGG-TTT--CCTTGGCGAGTTGTAATTTGAAGAGAGT-G-TTTTGAC-ATTCAGGTTTAATT-TAAATCCTTCGGGA--TAAGGTATCAT-AG-AGGGTGAGAATCCCGTTTATGATTAAATCTCG---AGT--GTCACCTAATTCACTTTCCAAGAGTCGAGTTG-TTTGGGATTGCAGCTCAAAACGGGTGGTAAATTTCACCTAAGGCTAAATATGTGCAAGAGACCGATAGCGAACAAGTACCGT-GAGGGAAAGA--TGAAAAGAACTTTGAAAAGAGAGTTAAATAGTACGTGAAATTGTTGAAAGGGAAACGATTGAAGTCAGTCATGCTAGTGGGGAATCAACTTGATGGTG-------TGTGGG----------------------------------TTCT-CTCGCATACTTTCCGGTGCACTTCCTT--ACTTGGCAGGTTAGCATCAA-TTTCGGTTATCATCCAAAATTT-GAGGGAATGTGGCTTT---TCTTCGGAAAAGTG-TTATAGACCTTGAT-CTATGTGGTGACCGGGATTGAGGATTGCAGCGGATACC-CTCGT-----GGCTTGTCACCTGG-CTTCTGACG-GTTACCCC--GCTTTCGATAGC-TCGCTAACGTTAGTGGGACTCGATTGTCTAG-AT-GTTAGAGTGATTAATT--TTCGCTAAGGATGCTGACGTAATGGCTTTAAGCGACCCGTCTTGAAACACGGACCAAGGAGTCTAAC-ATGTGTGCGAGTGTT

>FR846385_Acaulospora_punctata

CCTAGTAAGCGTGAGTCATCAGCTCACGTTTATTACGTCCCT-GCCCTTTGTACACACCGCCCGTCGCTACTACCGATTGAAT-GGCTTAGTGAGACTCTCGGATCGGGTTTTTGGAACCGGCAACGGTTCCTTTCATCTGAGAAGTTTGTCAAACTTGGTCATTTAGAGGAAGTAAAAGTCGTAACAAGGTTTCCGTAGGTGAACCTGCGGAAGGATCATTAGAAA---------------------------------TTT-------------------TATGTATTT-AAAAATTTTAAATCTTTTT--------TA--A---TTTA------AAAAAG-TCG-------AAATAAATTATTAAGATAACTTTCAACAACGGATCTCTTGGCTCTTGCATCGATGAAGAACGCAGCGAAATGCGATAAGTAATGTGAATTGCAGAATTCCGTGAATCATCAAATCTTTGAACGCAAATTGCACTCTTT-GGTATTCCGAGGAGTATGCTTGTTTGAGGGTTGTTCCA---ATAAAATCG--------------------TTTTTCT-----------AAAAAACGGATCTGAG---TTTTCCAAGG---TT--TTA-----------------------TTATTTTGGTAACTTT-AAATTTATCTTAACGATATCTTTGG----TTATGA-TA-TTGG-AAATGG-TTATATT-CTTACATC---------------TTCTTGTGAG---TTT-CA-TTAACAATTGAT---TACC---TGTCGTTACGTCGTA--TTATTTGCCTGA------------------------------TCCTTTTT--AA----TT-GGG--T-TACATAA-TACAA-TTTT-----TCT----TTCAACACCTCAAGTC-AAGTAAG-AATACCCGCTGAAC-TT-AAGCATATCAATAAGCGGAGGAAAAGAAACTAAC-AAGGATTCCC-TTAG-TAAC-GGCGAGTGAAGTGGGAAGAGCTCAAA-TTTTAAATCTCCGAGG-TTT--CCTTGGCGAGTTGTAATTTGAAGAGAGT-G-TTTTGAC-ATTCAGGTTTAATT-TAAATCCTTTGGGA--TAAGGTATCAT-AG-AGGGTGAGAATCCCGTTTATGATTAAATCTCG---AAT--GTCACTTAATTCACTTTCCAAGAGTCGAGTTG-TTTGGGATTGCAGCTCAAAACGGGTGGTAAATTTCACCTAAGGCTAAATATGTGCAAGAGACCGATAGCGAACACGTACCGT-GAGGGAAAGA--TGAAAAGAACTTTGAAAAGAGAGTTAAATAGTACGTGAAATTGTTGAAAGGGAAACGATTGAAGTCAGTCATGCTAGTGGGGAATCAACTTGATGGTG-------TGTGGG----------------------------------TTCT-CTCGCATACTTTCCGGTGCACTTCCTT--ACTTGGCAGGTTAGCATCAA-TTTCGGTTATCATCCAAAATTT-GAGGGAATGTGGCTTT---TCTTCGGAAAAGTG-TTATAGACCTTGAT-CTATGTGGTGACCGGGATTGAGGATTGCAGCGGATACC-TTCGT-----GGCTTGTCACCTGG-CTTCTGACG-GTTACCCC--GCTTTCGATAGC-TCGCTAACGTTAGTGGGACTCGATTGTCTAG-AT-GTTAGAGTGATTAATT--TTCGCTAAGGATGCTGACGTAATGGCTTTAAACGACCCGTCTTGAATCACGGACCAAGGAGTCTAAC-ATGTGTGCGAGTGTT

>KY413817_Acaulospora_spinulifera

-----------------------------------------------------------------------------------------------------------------------------------------------------------------------------------------------------------------------------------------------------------------------------------------------------------------------------------------------------------------------------------------------------------------------------------------------------------------------------------------------------------------------------------------------------------------------------------------------------------------------------------------------------------------------------------------------------------------------------------------------------------------------------------------------------------------------------------------------------------------------------------------------------------------------------------------------------------------------------------------------------------------------------------AC-GGCGAGTGAGTGGGAGAG---CTCAA-TTTTAAATCTCCGAGG-TTT--CCTTGGCGAGTTGTAATTTGAAGAAAGT-G-TTTTGAC-ATTCGTATTTAATT-TAAATCCTTTGGGA--TAAGGTATCAT-AG-AGGGTGAGAATCCCGTTTATGATTAAATCTCG---TGT--GTCACCTAATTCACTTTCCAAGAGTCGAGTTG-TTTGGGATTGCAGCTCAAAACGGGTGGTAAATTTCACCTAAGGCTAAATATTTGCAAGAGACCGATAGCGAACAAGTACCGT-GAGGGAAAGA--TGAAAAGAACTTTGAAAAGAGAGTTAAATAGTACGTGAAATTGTTGAAAGGGAAACGATTGAAGTCAGTCATGCTAGTGAGGAATCAACTTGATGGTC-------TGTGGG----------------------------------TTCG-CTCGCATACTTTCCGGTGCACTTCCTC--ACTCGGCAGGTTAGCATCAA-TTTCGGTTATCATAAAAAATTT-GAGGGAATGTGACTTT---TCTTCGGAAAAGTG-TTATAGACCTTGAT-CTATGTGGTGACCGGGATTGAGGATTGCAGCGGATACC-CTCGT-----GGCTGGTCACCTGG-CTTCTGACG-GTTACCCC--GCTTTCGATAGC-TCGCTAACGTTAGTGGGACTCGATTGTCTAG-AT-GTCAGAGTGATTCTA--------------------------------------------------------------------------------------

>KY413814_Acaulospora_spinulifera

-----------------------------------------------------------------------------------------------------------------------------------------------------------------------------------------------------------------------------------------------------------------------------------------------------------------------------------------------------------------------------------------------------------------------------------------------------------------------------------------------------------------------------------------------------------------------------------------------------------------------------------------------------------------------------------------------------------------------------------------------------------------------------------------------------------------------------------------------------------------------------------------------------------------------------------------------------------------------------------------------------------------------------------AC-GGCGAGTGAGTGGGAGAG---CTCAA-TTTTAAATCTCCGAGG-TTT--CCTTGGCGAGTTGTAATTTGAAGAAAGT-G-TTTTGAC-ATTCGTATTTAATT-TAAATCCTTTGGGA--TAAGGTATCAT-AG-AGGGTGAGAATCCCGTTTATGATTAAATCTCG---TGT--GTCACCTAATTCACTTTCCAAGAGTCGAGTTG-TTTGGGATTGCAGCTCAAAACGGGTGGTAAATTTCACCTAAGGCTAAATATTTGCAAGAGACCGATAGCGAACAAGTACCGT-GAGGGAAAGA--TGAAAAGAACTTTGAAAAGAGAGTTAAATAGTACGTGAAATTGTTGAAAGGGAAACGATTGAAGTCAGTCATGCTAGTGAGGAATCAACTTGATGGTC-------TGTGGG----------------------------------TTCG-CTCGCATACTTTCCGGTGCACTTCCTC--ACTCGGCAGGTTAGCATCAA-TTTCGGTTATCATAAAAAATTT-GAGGGAATGTGACTTT---TCTTCGGAAAAGTG-TTATAGACCTTGAT-CTATGTGGTGACCGGGATTGAGGATTGCAGCGGATACC-CTCGT-----GGCTGGTCACCTGG-CTTCTGACG-GTTACCCC--GCTTTCGATAGC-TCGCTAACGTTAGTGGGACTCGATTGTCTAG-AT-GTCAGAGTGATTCTA--------------------------------------------------------------------------------------

>AJ891120_Acaulospora_paulinae

-----------------------------------------------------------------------------------------------------------------------------------------------------------------------------------------------------------------------------AGAAAT--------------------------------TTTT-------------------TATGTATCC-AAAAATTTTAAATCTATGT---T-AT-AT--T---GTAT------TATTTG-TTA-------T-TTAAATAAACGAGATAACTTTCAACAACGGATCTCTTGGCTCTTGCATCGATGAAGAACGCAGCGAAATGCGATAAGTAATGTGAATTGCAGAATTCCGTGAATCATCAAATCTTTGAACGCAAATTGCACTCCTT-TGTATTCCGAGGAGTATGCTTGTTTGAGGGTTGTTCCA---ATAAATTCG--------------------TTTTTTT------------AAAAATGGATCTGAG---TTTTCCA--------------------------------------ATTTTGGTAACTTG-AAATTTATCTTAACGATATCTTTGG-----TATGA-TA-TTGA-AAATGG-TCATATT-CTTACA------------------TCTCGTGAG---TTT-CA-TTAACAATTGAT---TACC---TGTCGTTACGTCGTG--TCATTTGTCTAA------------------------------TCCTTTTT--TG----AT-GGG--T-CATGTGA-TACAA-TTCT-----TC-----TT-------------------------------------------GCATATCAATAAGCGGAGGAAAAGAAACTAAC-AAGGATTCCC-CTAG-TAAC-GGCGAGTGTAGCGGGAAGAGCTCAAA-TTTTAAATCTCCGAGG-TTT--CCTTGGCGAGTTGTAATTTGAAGAGAGT-G-TTTTGAC-ATTCGGGTTTAATC-CAAATCCTTTGGGA--TAAGGTATCAT-AG-ATGGTGAGAATCCCGTTTATGATTGAATCTCG---TGT--GTCGCTTAATTCACTTTCCAAGAGTCGAGTTG-TCTGGGATTGCAGCTCAAAACGGGTGGTAAATTTCACCTAAGGCTAAATATGTGCAAGAGACCGATAGCGAACAAGTACCGT-GAGGGAAAGA--TGAAAAGAACTTTGAAAAGAGAGTTAAATAGTACGTGAAATTGTTGAAAGGGAAACGATTGAAGTCAGTCATGCTAGTGAGGAATCAACCTGATGGTG-------TGTGGG----------------------------------TTTT-CCCTCGTACTTTCTGGTGCACTTTCTC--ACCCGGCAGGTTAGCATCAA-TTTCGGTTATCATAAAAAATTT-GAGAGAATGTGGCTTT---TCTTCGGAAAAGTG-TTATAGATCTCGAT-CTATGTGGTGACCGGGATTGAGGATTGCAGCGGATACC-CTCGT-----GGCTCGTCACCTGG-CTTCTGACG-GATGCCCC--GCCTTCGATAGC-TCGCTAACGTCGGTGGGACTCGATTGTCTAG-AT-GTCGGAGTGATTGATT--TTCGCTAAGGATGCTGACGTAATGGCTTTAAACGAC-----------------------------------------------

>AJ891119_Acaulospora_paulinae

-----------------------------------------------------------------------------------------------------------------------------------------------------------------------------------------------------------------------------AGAAAT--------------------------------TTTT-------------------TATGTATTC-AAAAATTTTAAATCTATGT---T-AT-AT--T---GTAT------TATTTG-TTA-------T-TTAAATAAACGAGATAACTTTCAACAACGGATCTCTTGGCTCTTGCATCGATGAAGAACGCAGCGAAATGCGATAAGTAATGTGAATTGCAGAATTCCGTGAATCATCAAATCTTTGAACGCAAATTGCACTCCTT-GGTATTCCGAGGAGTATGCTTGTTTGAGGGTTGTTCCA---ATAAATTCG--------------------TTTTTTT------------AAAAATGGATCTGAG---TTTTCCA--------------------------------------ATTTTGGTAACTTG-AAATTTATCTTAACGATATC-TTGG-----TATGA-TA-TTGA-AAATGG-TCATATT-C-TACA------------------TCTCGTGAG---TTT-CA-TTAACAATTGAT---TACC---TGTCGTTACGTCGTG--TCATTTGCCCAA------------------------------TCCTTTTT--GA----TT-GGG--T-CATGTGA-TACAA-TTCT-----TCT----TT-------------------------------------------GCATATCAATAAGCGGAGGAAAAGAAACTAAC-AAGGATTCCC-TTAG-TAAC-GGCGAGTGAAGCGGGAAGAGCTCAAA-TTTTAAATCTCCGAGGTTTT--CCTTGGCGAGTTGTAATTTGAAGAGAGT-G-TTTTGACTGTTCGGGTTCAATC-CAAATCCTTTGGGA--TAAGGTATCAT-AG-AGGGTGAGAATCCCGTTTATGATTGAATCTCG---AGT--GTCGCTTAATTCACTTTCCAAGAGTCGAGTTG-TTTGGGATTGCAGCTCAAAACGGGTGGTAAATTTCACCTAAGGCTGAATATGTGCAAGAGACCGATAGCGAACAAGTACCGT-GAGGGAAAGA--TGAAAAGAACTTTGAAAAGAGAGTTAAATAGTACGTGAAATTGTTAAAAGGGAAACGATTGAAGTCAGTCATGCTAGTGAGGAATCAACCGGATGGTG-------CGTGGG----------------------------------TTCT-CCCTCGTACTTTCCGGTGCACTTTCTC--ACCCGGCAGGTTAGCATCAA-TTTCGGTTATCATAAAAAATTT-GAGAGAATGTGGCTTT---TCTTCGGGAAAGTG-TTATAGATCTCGAT-CTATGTGGTGACCGGGATTGAGGATTGCAGCGGATACC-CTCGT-----GGCTCGTCACCTGG-CTTCTGACG-GTTACCCCA-GCTTTCGATAGC-TCGCTAACGTTAGTGGGACTCGATTGTCTAG-AT-GTCAGAGTGATTGATT--TTCGCTAAGGATGCTGACGTAATGGCTTTAAACGAC-----------------------------------------------

>AM076384_Acaulospora_sieverdingii

-----------------------------------------------------------------------------------------------------------------------------------------------------------------------------------------------------------------------------AGAAAT--------------------------------TTTT-------------------TATGTATTC-AAAAATTTTAAATCTTTAT---T-AC--------------------ATATA-ATA-------T-TTAAATAAACGAGATAACTTTCAACAACGGATCTCTTGGCTCTTGCATCGATGAAGAACGCAGCGAAATGCGATAAGTAATGTGAATTGCAGAATTCCGTGAATCATCAAATCTTTGAACGCAAATTGCACTCCTT-GGTATTCCGAGGAGTATGCTTGGTTGAGGGTTGTTCCA---ATAAATTCG--------------------TTTTTTC-----------AAAGAACGGATCTGAG---TTTTCCAAA--------TTA-----------------------TAAATTTGGTAACTTG-AAATTTATCTTAACGATATCTTTGG-----TATGA-TA-TTG--AAATGG-TCATATT-CTTGCA------------------TCTCGCGAG---TTT-CA-TTCACAATTAAT---TACC---TGTCGTTACGTCGTA--TCATTTGCCTAA------------------------------TCC-TTTT--GA----TT-GGG--T-CATGTGA-TACAA-TTTT-----CT-----------------------------------------------------------------------------------------------------------------------------------------------------------------------------------------------------------------------------------------------------------------------------------------------------------------------------------------------------------------------------------------------------------------------------------------------------------------------------------------------------------------------------------------------------------------------------------------------------------------------------------------------------------------------------------------------------------------------------------------------------------------------------------------------------------------------------------------------------------------------------------------------------------------------------------------------

>AM076382_Acaulospora_sieverdingii

----------------------------------------------------------------------------------------------------------------------------------------------------------------------------------------------------------------------------AGAAATT--------------------------------TTTT-------------------TATGTATTC-AAAAATTTTAAATCTTTTT---T-AC--------------------ATATA-ATA-------T-TTAAATAAACGAGATAACTTTTAACAACGGATCTCTTGGCTCTTGCATCGATGAAGAACACAGCGAAATGCGATAAGTAATGTGAATTGCAGAATTCCGTGAATCATCAAATCTTTGAACGCAAATTGCACTCCTT-GGTATTCCGAGGAGTATGCCTGGTTGAGGGTTGTTCCA---ATAAATTCG--------------------TTTTTTC-----------AAAGAACGGATCTGAG---TTTTCCAAA--------TTC-----------------------TAAATTTGGTAACTTG-AAATTTATCTTAACGATATCTTTGG-----TATGA-TA-TTGA-AAATGG-TCATATT-CTTGCT------------------TCTCGCGAG---TTT-CA-TTAGCAATTGAT---TACC---TGTCGTTACGTCGTA--TCATTTGCCTAA-------------------------------CC-TTTT--GA----TT-GGG--T-CATGTGA-TACAA-TTTT-----CT-----------------------------------------------------------------------------------------------------------------------------------------------------------------------------------------------------------------------------------------------------------------------------------------------------------------------------------------------------------------------------------------------------------------------------------------------------------------------------------------------------------------------------------------------------------------------------------------------------------------------------------------------------------------------------------------------------------------------------------------------------------------------------------------------------------------------------------------------------------------------------------------------------------------------------------------------

>AJ239115_Acaulospora_denticulata

------------------------------------------------------------------------------------------------------------------------------------------------------------------------------------------------------------------------------GAAAT--------------------------------TTTT-------------------TATGTATTC-AAAAAATTTTAAATCTTCT---TTAT-------------T-----GGAAAA-ATATTTATGTAAAATAATTCATGAGACAACTTTCAACAACGGATCTCTTGGCTCTTGCATCGATGAAGAACGCAGCGAAATGCGATAAGTAATGTGAATTGCAGAATTCCGTGAATCATCAAATCTTTGAACGCAAATTGCACTCTTT-GGTATTCCGAGGAGTATGCTTGTTTGAGGGTTGTTCCA---ATAAATTCG--------------------TTTCTTA--------AAAACAAAACGGATCTGAG---TTTTCCAAGATTTTT--TTA-----------------------TGATTTTGGTAACTTG-AAATTTATCTTAACGATATCTTTGG-----TATGA-TA-TTGG-AAATGG-TCATATT-CTTACATA---------------TTCCTGTGAG---TTT-CA-TTAACAATTGAT---TACC---TGTCGTTACGTCGTA--TTATTTGCCCGA------------------------------TCCACCTT--AA----TT-GGG--T-CACATGA-TACAA-TTTT-----CCC----T----------------------------------------------------------------------------------------------------------------------------------------------------------------------------------------------------------------------------------------------------------------------CA-----------------------------------------------------------------------------------------------------------------------------------------------------------------------------------------------------------------------------------------------------------------------------------------------------------------------------------------------------------------------------------------------------------------------------------------------------------------------------------------------------------------------------------------------------------------------------------------------------------------------------------------------------------------

>MT112118_Acaulospora_denticulata

-------------------------------------------------------------------------------------------TGAGACAC-------------------------------------------------------------------------------------------------------------------------------------------------------------------------------------------------------------------------------------------------------------------------------CAACCTTCAACAACGGATCTCTTGGCTCTTGCATCGATGAAGAACGCAGCGAAATGCGATAAGTAATGTGAATTGCAGAATTCCGTGAATCATCAAATCTTTCGGAGCAAATTGCACTCTTT-GGTATTCCGAGGAGTATGCTTGTTTGAGGGTTGTTCCA---ATAAATTCG--------------------TTTCTTA--------CGAACAAAACCTAGCTGAG---TCTTCCAAGATTTTT--TTA-----------------------TGATTTTGGTAACTTG-AAATTTATCTTAACGATATCTTTGG-----TATGA-TA-TTGG-AAATGG-TCATATT-CTTACATA---------------TTCCTGTGAG---TTT-CA-TTAACCTATGAC---TGTC---GCTGCTTACGTCGTA--TTATTTGCCC----------------------------------------------------------------------------------------------------------------------------------------------------------------------------------------------------------------------------------------------------------------------------------------------------------------------------------------------------------------------------------------------------------------------------------------------------------------------------------------------------------------------------------------------------------------------------------------------------------------------------------------------------------------------------------------------------------------------------------------------------------------------------------------------------------------------------------------------------------------------------------------------------------------------------------------------------------------------------------------------------------------------------------

>Acaulospora_mendoncae_OK392597

CCTAGTAAGCGTGAGTCATCAGCTCACGTTGATTACGTCCCT-GCCCTTTGTACACACCGCCCGTCGCTACTACCGATTGAAT-GGCTTAGTGAGACTCTCGGATTGGATCTAATGAACCGGCAACGGTTCCTTTTTTCTGAAAAGTTTGTCAAACTTGGTCATTTAGAGGAAGTAAAAGTCGTAACAAGGTTTCCGTAGGTGAACCTGCGGAAGGATCATTAGAATT----------------------------------------------------TCTTTTTATTC-AAAAATTTCCAACCTTATAT--------------TTTTATAA---AAAAA-----------AAATATTATTATTAAGACAACTTTCAACAACGGATCTCTTGGCTCTTGCATCGATGAAGAACGCAGCGAAATGCGATAAGTAATGTGAATTGCAGAATTCCGTGAATCATCAAATCTTTGAACGCAAATTGCACTCTTT-GGTATTCCGAAGAGTATGCTTGGTTGAGGGTTGTTCTA---CTAAATTCG--------------------TTTT-TCTA-------------TACGGATCTGAG---TTTTCCAAAG--TTT--TTA-----------------------AAATTTTGGTAACTTT-AAATTTACCTTAATGATAT-----------TATAA-TA-TTAG-AAATAT-T--TTAT-CTTACATA---------------TGTAAGT-----TTTT-TA-TTGATAATTATTA--TAAT--TAATCATTATACCATA--TTATTTATCTAA-------------------------------TT-TTTT--AA----TT-AGG--T-TAAGTAA-TATAA-TTAT-----TTTTT--TCTTCAACCTCAAGTC-AAGTAAG-GTTACCCGCTGAAC-TT-AAGCATATCAATAAGCGGAGGAAAAGAAACTAAC-AAGGATTCCC-TTAG-TAAC-GGCGAGTGAAGTGGGAAGAGCTCAAA-TTTTAAATCACTAAGG-TTTCACTTTAGTGAGTTGTAATTTGAAGAAAGT-G-TTTTGAT-ATTCGGGTTTGATT-TAAATCCTTTGGGA--TAAGGTATCAT-AG-AGGGTGAGAATCTCGTTCATGATTAAACCTTG---GGT--GTCACATAATTCACTTTCCAAGAGTCGAGTTG-TTTGGGATTGCAGCTCAAAATGGGTGGTAAATTTCACCTAAGGCTAAATATATGCAAGAGACCGATAGCGAACAAGTACCGT-GAGGGAAAGA--TGAAAAGAACTTTGAAAAGAGAGTTAAATAGTACGTGAAATTGTTGAAAGGGAAACGATTAGAGTCAGTCATGCTAGTAGGGAATCAGCTTGATGGAT-------CGTAAG--------------------------------ATTTAT-CTTATGATCTTTCTTGTGTACTTCCTT--GCTTGGCAGGTTAGCATCAA-TTTTGGTCATTATTAAAAAATC-TCGGGAATGTAGCTTT---TCTTCGGGAAAGTG-TTATAGACCTTGAT-AAGTGTGGTGACCGGGATTGGGGATTGCAGCGGATACC-TTTT----AAGGCTAGTTACCTGG-CTTCTGACG-GTTACCCC--GCTTTTGATAGC-TTGCTAATGATTGCGGGATTCGACAGTCTAGAAT-GTTAGAGTTATCATAAA-TTCGCTAAGGATGCTGACGTAATGGCTTTAAACGACCCGTCTTGAAACACGGACCAAGGAGTCTAAC-ATATGTGCGAGTGTT

>Acaulospora_mendoncae_OK392598

CCTAGTAAGCGTGAGTCATCAGCTCACGTTGATTACGTCCCT-GCCCTTTGTACACACCGCCCGTCGCTACTACCGATTGAAT-GGCTTAGTGAGACTCTCGGATTGGATCTAATGAACCGGCAACGGTTTCTTTTTTCTGAAAAGTTTGTCAAACTTGGTCATTTAGAGGAAGTAAAAGTCGTAACAAGGTTTCCGTAGGTGAACCTGCGGAAGGATCATTAGAAAT---------------------------------------T------------TTAATTTATTC-AAAAATTTCCAACCTTATAT--------------TTTTTT-----AAAAA-----------AAATAATAATAATAAGACAACTTTCAACAACGGATCTCTTGGCTCTTGCATCGATGAAGAACGCAGCGAAATGCGATAAGTAATGTGAATTGCAGAATTCCGTGAATCATCAAATCTTTGAACGCAAATTGCACTCTTT-GGTATTCCGAAGAGTATGCTTGCTTGAGGGTTGTTTTA---CTAAATTCG--------------------TTTT-TCTA-------------TACGGATCTGAG---TTTTCCAAAG--TTT--TTA-----------------------AAATTTTGGTAACTTC-AAATTTACCTTGATGATAT-----------TATAA-TA-TTAG-AAATAT-T--TTAT-CTTACATA---------------TGTAAGT-----TTTT-TA-TTGATAATTATTA--TAAT--TAATCATTATACCATA--TTATTTATCTAA-------------------------------TT-TTTT--AA----TT-AGG--T-TAAGTAA-TATAA-TTTT-----TTTTT--TCTTCAACCTCAAGTC-AAGTAAG-GTTACCCGCTGAAC-TT-AAGCATATCAATAAGCGGAGGAAAAGAAACTAAC-AAGGATTCCC-TTAG-TAAC-GGCGAGTGAAGTGGGAAGAGCTCAAA-TTTTAAATCACTAAGG-TTTCATTTTAGTGAGTTGTAATTTGAAGAAAGT-G-TTTTGAT-ATTCGGGTTTGATT-TAAATCCTTTGGGA--TAAGGTATCAT-AG-AGGGTGAGAATCCCGTTCATGATTAAACCTTG---GGT--GTCACATAATTCACTTTCCAAGAGTCGAGTTG-TTTGGGATTGCAGCTCAAAATGGGTGGTAAATTTCACCTAAGGCTAAATATATGCAAGAGACCGATAGCGAACAAGTACCGT-GAGGGAAAGA--TGAAAAGAACTTTGAAAAGAGAGTTAAATAGTACGTGAAATTGTTGAAAGGGAAACGATTAAAGTCAGTCATGCTAGTAGGGAATCAACTTGATGGAT-------CGTAAG--------------------------------ATTTAT-CTTATGATCTTTCTTGTGTACTTCCTT--GCTTGGCAGGTTAGCATCAA-TTTTGGTCATTATTAAAAAATC-GAGGGAATGTAGCTTT---TCTTCGGGAAAGTG-TTATAGACCTTGAT-AAATGTGGTGACTGGGATTGAGGATTGCAGCGGATGCC-TTTT----AAGGCTAGTTACCTGG-CTTCTGACG-GTTACCCC--GCTTTTGATAGC-TTGCTAATGATTGCGGGATTCGACAGTCTAGAAT-GTTAGAGTTATCATAAA-TTCGCTAAGGATGCTGACGTAATGGCTTTAAACGACCCGTCTTGAAACACGGACCAAGGAGTCTAAC-ATGTGTGCGAGTGTC

>MN081001_Acaulospora_aspera

CCTAGTAAGCGTGAGTCATCAGCTCACGTTGATTACGTCCCT-GCCCTTTGTACACACCGCCCGTCGCTACTACCGATTGAAT-GGCTTAGTGAGACTCTCGGATTGGATCTAATGAACCGGCAACGGTTCCTTTTTTCTGAAAAGTTTGTCAAACTTGGTCATTTAGAGGAAGTAAAAGTCGTAACAAGGTTTCCGTAGGTGAACCTGCGGAAGGATCATTAGAATT---------------------------------------T------------TTTAATTATTC-AAAAATTTCCAACCTTATAT--------------TTTATTAA---AGAAA-----------AAATAATGATAATAAGACAACTTTCAACAACGGATCTCTTGGCTCTTGCATCGATGAAGAACGCAGCGAAATGCGATAAGTAATGTGAATTGCAGAATTCCGTGAATCATCAAATCTTTGAACGCAAATTGCACTCTTT-GGTATTCCGAAGAGTATGCTTGCTTGAGGGTTGTTCTA---CTAAATTCG--------------------TTTT-TCTA-------------TACGGATCTGAG---TTTTCCAAAG-TTTT--TTA-----------------------AGATTTTGGTAACTTC-AAATTTATCTTGGTGATAT-----------TATAA-TG-TTAG-AAATAT-T--TTAT-CTTACATA---------------TTTATGTAAG--TTTT-TA-TTGATAATTATTA--TAAT--TAATCATTATA-CATA--TTATTTATCTAA------------------------------TTT-TTTT--AA----TT-AGG--T-TAAGTAA-TATAA--TTT-----TTTTT--TCTTCAACCTCAAGTC-AAGTAAG-ATTACCCGCTGAAC-TT-AAGCATATCAATAAGCGGAGGAAAAGAAACTAAC-AAGGATTCCC-TTAG-TAAC-GGCGAGTGAAGTGGGAAGAGCTCAAA-TTTTAAATCACTAAGG-TTTCACTTTAGTGAGTTGTAATTTGAAGAGGGT-G-TTTTGAT-ATTCGGGTTTAATT-TAAATCCTTTGGGA--TAAGGTATCAT-AG-AGGGTGAGAATCCCGTTCATGATTAAACCTTG---GGT--GTCACATAATTCACTTTCTAAGAGTCGAGTTG-TTTGGGATTGCAGCTCAAAATGGGTGGTAAATTTCACCTAAGGCTAAATATATGCAAGAGACCGATAGCGAACAAGTACCGT-GAGGGAAAGA--TGAAAAGAACTTTGAAAAGAGAGTTAAATAGTACGTGAAATTGTTGAAAGGGAAACGATTAAAGTCAGTCATGCTAGTAGGGAATCAACTTGATGGAT-------CGTAAG--------------------------------ATTTAT-CTTATGACCTTTCTTGTGTACTTCCTT--GCTTGGCAGGTTAGCATCAA-TTTTGGTCATCATTAAAAAATC-GAGGGAATGTAGCTTT---TCTTCGGGAAAGTG-TTATAGACCTTGAT-AAATGTGGTGGTCGGGATTGAGGATTGCAGCGGATACCTTTTT----AAGGCTAGTTACCTGG-CTTCTGACA-GTTACCCC--GCTTTCGATAGC-TTGCTAATGATTGTGGGATTCGACAGTCTAGAAT-GTCAGAGTTATCATAAA-TTCGCTAAGGATGCTGACGTAATGGCTTTAAACGACCCGTCTTGAAACACGGACCAAGGAGTCTAAC-ATGTGTGCGAGTGTA

>MN080999_Acaulospora_aspera

CCTAGTAAGCGTGAGTCATCAGCTCACGTTGATTACGTCCCT-GCCCTTTGTACACACCGCCCGTCGCTACTACCGATTGAAT-GGCTTAGTGAGACTCTCGGATTGGATCTAATGAACCGGCAACGGTTCCTTTTTTCTGAAAAGTTTGTCAAACTTGGTCATTTAGAGGAAGTAAAAGTCGTAACAAGGTTTCCGTAGGTGAACCTGCGGAAGGATCATTAGAATT---------------------------------------T------------TTCAATTATTC-AAAAATTTCCAACCTTATAT--------------TTTAT------AAAAG-----------AAAAAATAATAATAAGACAACTTTCAACAACGGATCTCTTGGCTCTTGCATCGATGAAGAACGCAGCGAAATGCGATAAGTAATGTGAATTGCAGAATTCCGTGAATCATCAAATCTTTGAACGCAAATTGCACTCTTT-GGTATTCCGAAGAGTATGCTTGCTTGAGGGTTGTTCTA---CTAAATTCG--------------------TTTT-TCTA-------------TACGGATCTGAG---TTTTCCAAAG--TTT--TTA-----------------------AAATTTTGGTAACTTT-AAATTTATCTTGATGATAT-----------TATAA-TG-TTAG-AAATAT-T--TTAT-CTTACATA---------------TTTATGTAAG--TTTT-TA-TTGATAATTATTA--TAAT--TAATCATTATATCATA--TTATTTATCTAA-----------------------------TTTT-TTTT--AA----TT-AGG--T-TAAGTAA-TATAA---TT-----TTTTT--TCTTCAACCTCAAGTC-AAGTAAG-ATTACCCGCTGAAC-TT-AAGCATATCAATAAGCGGAGGAAAAGAAACTAAC-AAGGATTCCC-TTAG-TAAC-GGCGAGTGAAGTGGGAAGAGCTCAAA-TTTTAAATCACTAAGG-TTTCACTTTAGTGAGTTGTAATTTGAAGAGAGT-G-TCTTGAT-ATTCGGGTTTAATT-TAAATCCTTTGGGA--TAAGGTATCAT-AG-AGGGTGAGAATCCCGTTCATGATTAAACCTT----GGT--GTCACATAATTCACTTTCTAAGAGTCGAGTTG-TTTGGGATTGCAGCTCAAAATGGGTGGTAAATTTCACCTAAGGCTAAATATATGCAAGAGACCGATAGCGAACAAGTACCGT-GAGGGAAAGA--TGAAAAGAACTTTGAAAAGAGAGTTAAATAGTACGTGAAATTGTTGAAAGGGAAACGATTAAAGTCAGTCATGCTAGTAGGGAATCAACTTGGTGGAT-------CGTAAG--------------------------------ATTTAT-CTTGTGATCTTTCTTGTGTACTTCCTT--GCTTGGCAGGTTAGCATCAA-TTTTGGTCATCATTAAAAAATC-TAGGGAATGTAGCTTT---TCTTCGGGAAAGTG-TTATAGACCTTAAT-AAATGTGGTGGCCGGGATTGGGGATTGCAGCGGATACC-TTTT----AAGGCTAGTTACCTGA-CTTCTGACG-GTTGCCCC--GCTTTCGATAGC-TTGCTAATGATTGTGGGATTCGACAGTCTAGAAT-GTTGGAGTTATCATAAA-TTCGCTAAGGATGCTGACGTAATGGCTTTAAACGACCCGTCTTGAAACACGGACCAAGGAGTCTAAC-ATGTATGCGAGTGTT

>HG422734_Acaulospora_spinosissima

CCTAGTAAGCGTGAGTCATCAGCTCACGTTGATTACGTCCCT-GCCCTTTGTACACACCGCCCGTCGCTACTACCGATTGAAT-GGCTTAGTGAGACTCTCGGATTAGTTTTAATGAACCGGCAACGGTTCCTTCTTGCTGAGAAGTTTGTCAAACTTGGTCATTTAGAGGAAGTAAAAGTCGTAACAAGGTTTCCGTAGGTGAACCTGCGGAAGGATCATTAGAATT---------------------------------------T-----------CTTCATTTATTC-AAAAATTTCCAACCTTTTAT-----------AAATTTTATAAA--AAAAA-----------AAGAAATAATAATAAGACAACTTTCAACAACGGATCTCTTGGCTCTTGCATCGATGAAGAACGCAGCGAAATGCGATAAGTAATGTGAATTGCAGAATTCCGTGAATCATCAAATCTTTGAACGCAAATTGCACTCTTT-GGTATTCCGAAGAGTATGCTTGCTTGAGGGTTGTCCTA---TTAAATTCG--------------------TTTTATTAA-------------TACGGATCTGAG---CTTTCCAAAG--TTT--TTA-----------------------AAATTTTGGTAACTTT-AAATTCATCTTCATGATATTAT--------TATGA-TA-TTAG-AAATAT-T--TTAT-CTTACATA---------------TTCGTGTAAG--TTTT-TA-TTGATAATTATTA--TAAT--TAATC---ATATCATA--TTATTTGCCTAA--TT-------------------------TTTT-TTTT--AA----TT-AGG--T-TAAATAA-TATAA---------TTTTTT--TCTTCAACCTCAAGTC-AAGTAAG-ATTACCCGCTGAAC-TT-AAGCATATCAATAAGCGGAGGAAAAGAAACTAAC-AAGGATTCCC-TTAG-TAAC-GGCGAGTGAAGTGGGAAGAGCTCAAA-TTTTAAATCACTAAGG-TTTCACTTTAGTGAGTTGTAATTTGAAGAAAGT-G-TTTTGAT-ATTCGGGTTTGATC-TAAATCCTTTGGGA--TAAGGTATCAT-AG-GGGGTGAGAATCCCGTTCATGATTAAACCTTG---GGT--GTCACATAATTCACTTTCCAAGAGTCGAGTTG-TTTGGGATTGCAGCTCAAAATGGGTGGTAAATTTCACCTAAGGCTAAATATATGCAAGAGACCGATAGCGAACAAGTACCGT-GAGGGAAAGA--TGAAAAGAACTTTGAAAAGAGAGTTAAATAGTACGTGAAATTGTTGAAAGGGAAACGATTAAAGTCAGTCATGCTAGTAGGGAATCAACCTGATGGAT-------CGTAAG-----------------------------ATTATTTAT-CTTGTGATCTTTCTTGTGTACTTCCTT--GCTTGGCAGGTTAGCGTCAA-TTTCGTTCATCATTAAAAAATC-TCGGGAATGTAGCTTT---TCTTCGGGAAAGTG-TTATAGACCTTGAT-ATATGTGGTGACCGAGATTGAGGATTGCAGCGGATACC-TTTT----AAGGCTAGTCACCTGG-CTTCTGACG-GTTACCTC--GCTTTCGATAGC-TCGCTAATGATTGTGGGATTCGACAGTCTAGAAC-GTTAGAGTGATCATAAA-TTCGCTAAGGATGCTGACGTAATGGCTTTAAACGACCCGTCTTGAAACACGGACCAAGGAGTCTAAC-ATGTATGCGAGTGTT

>HG422732_Acaulospora_spinosissima

CCTAGTAAGCGTGAGTCATCAGCTCACGTTGATTACGTCCCT-GCCCTTTGTACACACCGCCCGTCGCTACTACCGATTGAAT-GGCTTAGTGAGACTCTCGGATTGGTTTTAATGAACCGGCAACGGTTCCTTCTTGCTGAGAAGTTTGTCAAACTTGGTCATTTAGAGGAAGTAAAAGTCGTAACAAGGTTTCCGTAGGTGAACCTGCGGAAGGATCATTAGAATT---------------------------------------T-----------CTTAATTTATTC-AAAAATTTCCAACCTTTTAT-----------AAATTTTAT-----AAAAA-----------AAAAAGAAATAATAAGACAACTTTCAACAACGGATCTCTTGGCTCTTGCATCGATGAAGAACGCAGCGAAATGCGATAAGTAATGTGAATTGCAGAATTCCGTGAATCATCAAATCTTTGAACGCAAATTGCACTCTTT-GGTATTCCGAAGAGTATGCTTGCTTGAGGGTTGTCCTA---TTAAATTCG--------------------TTTTATTAA-------------TACGGATCTGAG---TTTTCCAAAG--TTT--TTA-----------------------AAATTTTGGTAACTTT-AAATTTATTTTAATGATATTAT--------TATAA-TA-TTAG-AAATAT-T--TTAT-CTTGCATG---------------TTCGTGTAAG--TTTT-TA-TTGATAATTATTA--TAAT--TAATC---ATATCATA--TTATTTGCCTAATTTT-------------------------TTTT-TTTT--AA----TT-GGG--T-TAAGTAA-TATAA----------TTTTT--TCTTCAACCTCAAGTC-AAGTAAG-ATTACCCGCTGAAC-TT-AAGCATATCAATAGGCGGAGGAAAAGAAACTAAC-AAGGATTCCC-TTAG-TAAC-GGCGAGTGAAGTGGGAAGAGCTCAAA-TTTTAAATCACTAAGG-TTTCACTTTAGTGAGTTGTAATTTGAAGAAAGT-G-TTTTGAT-ATTCGGGTTTAATC-TAAATCCTTTGGGA--TAAGGTATCAT-AG-AGGGTGAGAATCCCGTTCATGATTAAACCTTG---GGT--GTCACATAATTTACTTTCTAAGAGTCGAGTTG-TTTGGGATTGCAGCTCAAAATGGGTGGTAAATTTCACCTAAGGCTAAATATATGCAAGAGACCGATAGCGAACAAGTACCGT-GAGGGAAAGA--TGAAAAGAACTTTGAAAAGAGAGTTAAATAGTACGTGAAATTGTTGAAAGGGAAACGATTAAAGTCAGTCATGCTAGTAGGGAATCAACCTGATGGAT-------CGTAAG--------------------------------ACTTAT-CTTGTGATCTTTCTTGTGTATTTCCTT--GCTTGGCAGGTTAGCATCAA-TTTCGTTCATCATTAAAAAATC-TCGGGAATGTAGCTTT---TCTTCGGGAAAGTA-TTATAGACCTTGAT-ATATGTGGTGATCGAGATTGAGGATTGCAGCGGATACC-TTTT----AAGGCTAGTCACCTGG-CTTCTGACG-GTTACCTC--GCTTTCGATAGC-TCGCTAATGATTGTGGGATTCGACAGTCTAGAAC-GTTAGAGTGATCATAAA-TTCGCTAAGGATGCTGACGTAATGGCTTTAAACGACCCGTCTTGAAACACGGACCAAGGAGTCTAAC-ATGTATGCGAGTGTT

>JX135571_Acaulospora_herrerae

--------------------------------------------------------------------------------------------------------------------------------------------------------------------------------------------------------------------------------------------------------------------------------------------------------------------------------------------------------------------------------------------------------------------------------------------------------------------------------------------------------------------------------------------------------------------------------------------------------------------------------------------------------------------------------------------------------------------------------------------------------------------------------------------------------------------------------------------------------------------------------------------------------------------------------------------------------------------------------GCATATCAATAAGCGGAGGAAAAGAAACTAAC-AAGGATTCCC-TTAG-TAAC-GGCGAGTGAAGTGGGAA-AGCTCAAA-TTTTAAATCACTAAGG-TTTCCCTTTAGTGAGTTGTAATTTGAAGAAAGT-G-TTTTGAT-ATCCGGATTTAATC-TAAATCCTTTGGGA--TAAGGTATCAT-AG-AGGGTGAGAATCCCGTTCATGATTTAATCTTG---GGT--GTCACATAATTCACTTTCTAAGAGTCGAGTTG-TTTGGGATTGCAGCTCAAAATGGGTGGTAAATTTCACCTAAGGCTAAATATGTGCAAGAGACCGATAGCGAACAAGTACCGT-GAGGGAAAGA--TGAAAAGAACTTTGAAAAGAGAGTTAAATAGTACGTGAAATTGTTGAAAGGGAAACGATTAAAGTCAGTCATGCTAGTAGGGAATCAACTTGATGGGT-------CGTGAG--------------------------------ATTTAT-CTCGTGATCTACCTTGTGTACTTCCTT--GCTTGGCAGGTTAGCACCAA-TTTTGGTTATCATTAAAAAATC-GAGGGAATGTAGCTTT---TCTTCGGAAAAGTG-TTATAGACCTTGAT-ATATGTGGTGACCGGGATTGAGGATTGCAGCGGATACCTTTTT----AAGGCTAGTCACCTGG-CTTTTGACG-GTTACCCC--GCTTTCGATAGC-TTGCTAACGATTGTGGGA-TAAACAGTCGAG-AT-ATTAGAGTGAACAT-AA-TTCGCTAAGGATGCTGACGTAATG-----------------------------------------------------------

>JX135573_Acaulospora_herrerae

--------------------------------------------------------------------------------------------------------------------------------------------------------------------------------------------------------------------------------------------------------------------------------------------------------------------------------------------------------------------------------------------------------------------------------------------------------------------------------------------------------------------------------------------------------------------------------------------------------------------------------------------------------------------------------------------------------------------------------------------------------------------------------------------------------------------------------------------------------------------------------------------------------------------------------------------------------------------------------GCATATCAATAAGCGGAGGAAAAGAAACTAAC-AAGGATTCCC-TTAG-TAAC-GGCGAGCG-AGTGGGAAAAGCTCAAA-TTTTAAATCACTAAGG-TTTCACTTTAGTGAGTTGTAATTTGAGGAAAGT-G-TTTTGAT-ATCCGGATTTAATC-TAAATCCTTTGGGA--TAAGGTATCAT-AG-AGGGTGAGAATCCCGTTCATGATTTAATCTTG---GGT--GTCACATAATTCACTTTCTAAGAGTCGAGTTG-TTTGGGATTGCAGCCCAAAATGGGTGGTAAATTTCACCTAAGGCTAAATATGTGTAAGAGACCGATAGCGAACAAGTACCGT-GAGGGAAAGA--TGAAAAGAACTTTGAAAAGAGAGTTAAATAGTACGTGAAATTGTTGAAAGGGAAACGATTAAAGTCAGTCATGCTAGTAGGGAATCAACTTGATGGGT-------CGTGAG--------------------------------ATTTAT-CTCGTGATCTATCTTGTGTACTTCCTT--GCTTGGCAGGTTAGCATCAA-TTTTGGTTATCATTAAAAAATC-GAGGGAATGTAGCTTT---TCTTCGGAAAAGTG-TTATAGACCTTGAT-ATATGTGGTGACCGGGATTGAGGATTGCAGCGGATACCTTTTT----AAGGCTAGTCACCTGG-CTTTTGACG-GTTACCCC--GCTTTCGATAGC-TTGCTAACGATTGTGGGA-TAAACAGTCGAG-AT-GTTAGAGTGAACATAAA-TTCGCTAAGGATGCTGACGTAATGG----------------------------------------------------------

>FM876830_Acaulospora_kentinensis

CCTAGTAAGCGTGAGTCATCAGCTCACGTTGATTACGTCCCT-GCCCTTTGTACACACCGCCCGTCGCTACTACCGATTGAAT-GGCTTAGTGAGACTCTCGGATTGGAGTGAAGGAACCGGCAACGGATCCTTTTTTCTGAGAAGTTTGTCAAACTTGGTCATTTAGAGGAAGTAAAAGTCGTAACAAGGTTTCCGTAGGTGAACCTGCGGAAGGATCATTAAAATT------------------------------------------------------TTTATATTC-AAAAATTTCCAACCTAAACT-TAAA------TAGTTCAGTAAA--TCAAT-----------CGAGATTTAAATAATGACAACTTTCAACAACGGATCTCTTGGCTCTTGCATCGATGAAGAACGCAGCGAAATGCGATAAGTAATGTGAATTGCAGAATTCCGTGAATCATCAAATCTTTGAACGCAAATTGCACTCTTT-GGTATTCCGAAGAGTATGCTTGCTTGAGGGTTGTTTAA---ATAAATTCG--------------------TATTTTTTA-------------TACGGATCTGAG---TCTTCCAAAA---TT--TTT-----------------------AAATTTTGGTAACTTA-AAATTTAACTTGACGATAT-----------TATAT-TA-TTAG-AAATGT-T--TTAT-CTTGCATA----------------TTTTGTAAG---TTT-CA-TTAATAATTATTA--TAAT--TAATCGTTATATCGTTA-TTATTTACCTAG------------------------------TTA-TTTCGGAA----TT-AGG--T-TAAGTAA-TACAA-ATTT-----TT-----TTTTCAACCTCAAGTC-AAGTAAG-AGTACCCGCTGAAC-TT-AAGCATATCAATAAGCGGAGGAAAAGAAACTAAC-AAGGATTTCC-TTAG-TAAC-GGCGAGTGAAGTGGTAAAAGCTCAAA-TTTTAAATCACCAAGG-TTT--CTTTGGTGAATTGTAATTTGAAGAAAGTCG-TTTTGAT-ATTCGGGTTTTATC-CAAGTCCTTTGGGA--TAAGGTATCAT-AG-AGGGTGAGAATCCCGTTCATGATTAAACCTT----GAT--GTCACTTAATTCACTTTCTAAGAGTCGAGTTG-TTTGGGATTGCAGCTCAAAATGGGTGGTAAATTTCACCTAAGGCTAAATATATGCAAGAGACCGATAGCGAACAAGTACCGT-GAGGGAAAGA--TGAAAAGAACTTTGAAAAGAGAGTTAAATAGTACGTGAAATTGTTGAAAGGGAAACGATTGAAGTCAGTCATGCTAGTAGGGAATCAACTTGATGGAT-------CGTAGG--------------------------------ACTTAT-CTTATGATCTTTCTTGTGCACTTTCTT--GCTTGGCAGGTTAGCATCAA-TTTCGGGTATCATTAAAAAATC-GAGGGAATGTAGCTTT---CTTTCGGGAAAGTG-TTATAGACCTTGATAAAATGTGGTGACCGAGATTGAGGATTGCAGCGGATACC-TCTT----AAGGCTAGTCACCTTG-CTTCTGACG-GTTACCCT--GCTTTCGAGAGC-TCGCTCACGATTGTGGGATTCAACAGTCTAG-AT-GTTAGAGTGATCTTAAA-TTCGCTAAGGATGCTGACGTAATGGCTTTAAACGACCCGTCTTGAAACACGGACCAAGGAGTCTAAC-ATGTATGCGAGTGTT

>FM876822_Acaulospora_kentinensis

CCTAGTAAGTGTGAGTCATCAGCTCACGTTGATTACGTCCCT-GCCCTTTGTACACACCGCCCGTCGCTACTACCGATTGAAT-GGCTTAGTGAGACTCTCGGATTGGAGTGAAGGAACCGGCAACGGATCCTTTTTTCTGAGAAGTTTGTCAAACTTGGTCATTTAGAGGAAGTAAAAGTCGTAACAAGGTTTCCGTAGGTGAACCTGCGGAAGGATCATTAAAATT------------------------------------------------------TTTATATTC-AAAAATTTCCAACCTAAACT-TAAA------TAGTTCAGTAAA--TCAAT-----------CGAGATTTAAATAATGACAACTTTTAACAACGGATCTCTTGGCTCTTGCATCGATGAAGAACGCAGCGAAATGCGATAAGTAATGTGAATTGCAGAATTCCGTGAATCATCAAATCTTTGAACGCAAATTGCACTCTTT-GGTATTCCGAAGAGTATGCTTGCTTGAGGGTTGTTTAA---ATAAATTCG--------------------TATTTTTTA-------------TACGGATCTGAG---TCNTCCAAAA---TT--TTT-----------------------AAATTTTGGTAACTTA-AAATTTAACTTGACGATAT-----------TATAT-TA-TTAG-AAATGT-T--TTAT-CTTGCATA----------------TTTTGTAAG---TTT-CA-TTAATAATTATTA--TAAT--TAATCGTTATATCGTTATTTATTTACCTAG------------------------------TTA-TTTCGGAA----TT-AGG--T-TAAGTAA-TACAA-ATTT-----TT-----TTTTCAACCTCAAGTC-AAGTAAG-AGTACCCGCTGAAC-TT-AAGCATATCAATAAGCGGAGGAAAAGAAACTAAC-AAGGATTTCC-TTAG-TAAC-GGCGAGTGAAGTGGTAAAAGCTCAAA-TTTTAAATCACCAAGG-TTT--CTTTGGTGAATTGTAATTTGAAGAAAGTCG-TTTTGAT-ATTCGGGTTTTATC-CAAGTCCTTTGGGA--TAAGGTATCAT-AG-AGGGTGAGAATCCCGTTCATGATTAAACCTT----GAT--GTCACTTAATTCACTTTCTAAGAGTCGAGTTG-TTTGGGATTGCAGCTCAAAATGGGTGGTAAATTTCACCTAAGGCTAAATATATGCAAGAGACCGATAGCGAACAAGTACCGT-GAGGGAAAGA--TGAAAAGAACTTTGAAAAGAGAGTTAAATAGTACGTGAAATTGTTGAAAGGGAAACGATTGAAGTCAGTCATGCTAGTAGGGAATCAACTTGATGGAT-------CGTAGG--------------------------------ACTTAT-CTTATGATCTTTCTTGTGCACTTTCTT--GCTTGGCAGGTTAGCATCAA-TTTCGGGTATCATTAAAAAATC-GAGGGAATGTAGCTTT---CTTTCGGGAAAGTG-TTATAGACCTTGATAAAATGTGGTGACCGAGATTGAGGATTGCAGCGGATACC-TCTT----AAGGCTAGTCACCTTG-CTTCTGACG-GTTACCCT--GCTTTCGAGAGC-TCGCTCACGATTGTGGGATTCAACAGTCTAG-AT-GTTAGAGTGATCTTAAA-TTCGCTAAGGATGCTGACGTAATGGCTTTAAACGACCCGTCTTGAAACACTGACCAAGGAGTCTAAC-ATATGTGCGAGTGTT

>KM057074_Acaulospora_excavata

---------------------------------------------------------------------------------------------------------------------------------------------------------------------------------------------------------------------------------------------------------------------------C-------------TAATTTATTC-AAAAATTTCCAACCTTATA------------AATTTTATTAA---AAAAA-----------AAAAAATAATAATAAGACAACTTTCAACAACGGATCTCTTGGCTCTTGCATCGATGAAGAACGCAGCGAAATGCGATAAGTAATGTGAATTGCAGAATTCCGTGAATCATCAAATCTTTGAACGCAAATTGCACTCTTT-GGTATTCCGAAGAGTATGCTTGCTTGAGGGTTGTTCTA---TCAAATTCG--------------------TATTTTTTA-------------TACGGATCTGAG---TTTTCCAAAG--TTT--TTA-----------------------AAATTTTGGTAACTTG-AAATTTATCTTAATGATAT-----------TATAA-TA-TTAG-AAATAT-T--TTAT-CTTACATA---------------TTTGTGTAAGTTTTTT-TA-TTGATAATCGTGTTATAAT--TAATCATGATATCATA--TTATTTAACTAA------------------------------TTT-TCAT--AA----TT-AGA--T-TAAGTAA-TATAA-TTTT-----TTTT---TCTTCAAC----------------------------------------------------------------------------------------------------------------------------------------------------------------------------------------------------------------------------------------------------------------------------------------------------------------------------------------------------------------------------------------------------------------------------------------------------------------------------------------------------------------------------------------------------------------------------------------------------------------------------------------------------------------------------------------------------------------------------------------------------------------------------------------------------------------------------------------------------------------------------------------------------------------------------------

>KM057076_Acaulospora_excavata

---------------------------------------------------------------------------------------------------------------------------------------------------------------------------------------------------------------------------------------------------------------------------C-------------TAATTTATTC-AAAAATTTCCAACCTTATA------------AATTTTATTA----AAAAA-----------AAAAAATAATAATAAGACAACTTTCAACAACGGATCTCTTGGCTCTTGCATCGATGAAGAACGCAGCGAAATGCGATAAGTAATGTGAATTGCAGAATTCCGTGAATCATCAAATCTTTGAACGCAAATTGCACTCTTT-GGTATTCCGAAGAGTATGCTTGCTTGAGGGTTGTTCTA---TCAAATTCG--------------------TATTTTTTA-------------TACGGATCTGAG---TTTTCCAAAG--TTT--TTA-----------------------AAATTTTGGTAACTTG-AAATTTATCTTAATGATAT-----------TATAA-TA-TTAG-AAATAT-T--TTAT-CTTACATA---------------TTTGTGTAAGTTTTTT-TA-TTGATAATTGTGTTATAAT--TAATCATGATATCATA--TTATTTAACTAA------------------------------TTT-TCAT--AA----TT-AGA--T-TAAGTAA-TATAA-TTTT-----TTTT---TCTTCAACCTCAAGTC-AAGTAAG-ATTACCCGCTGAAC-TT-AAGCATATCAATAAGCGGAGGAAAAGAAACTAAC-CAGGATTCCC-TTAG-TAAC-GGCGAGTGAAGTGGGAAGAGCTCAAA-TTTTAAATCACTAAGG-TTTCACTTTAGTGAGTTGTAATTTGAAGAAAGT-G-TTTTGAT------------------------------------------------------------------------------------------------------------------------------------------------------------------------------------------------------------------------------------------------------------------------------------------------------------------------------------------------------------------------------------------------------------------------------------------------------------------------------------------------------------------------------------------------------------------------------------------------------------------------------------------------------------------------------------------------------------------------------------------------

>FR821674_Acaulospora_minuta

CCTAGTAAGCGTGAGTCATCAGCTCACGTTGATTACGTCCCT-GCCCTTTGTACACACCGCCCGTCGCTACTACCGATTGAAT-GGCTTAGTGAGACTCTCGGATTGGGTTTTAAGAACCGGCAACGGATCTTTTCTTCTGAGAAGTTTGTCAAACTTGGTCATTTAGAGGAAGTAAAAGTCGTAACAAGGTTTCCGTAGGTGAACCTGCGGAAGGATCATTAGAAA--------------------------------TTTTGA---------------TTTTTTTATTC-AAAAA-ATTTAATCTTAATA-AAAATATT--TATG-CATATG---TAT-T-----------ATATCATAATATTAAGACAACTTTTAACAACGGATCTCTTGGCTCTTGCATCGATGAAGAACGCAGCGAAATGCGATAAGTAATGTGAATTGCAGAATTCCGTGAATCATCAAATCTTTGAACGCAAATTGCACTCTTT-GGTATTCCGAAGAGTATGCTTGCTTGAGGGTTGTTCAA---ATAAA-TCGT-GAAAA----------------TTTTTT-------------TGCGGACCTGAG---TTTTCCAAAG---TT--TCA-----------------------AAATTTTGGCAACTTC-AAATTTATCTTAACGATACTTTAAAGGTATTATTA-TA-TTGG-AAATGT-T--ATAT-CTCGCATA------------------TTGCAA----GTT-CA-TTGACAATTATTT--TACC---AGTCGTTA-TTCGTG--TTATTTATCTAA------------------------------TAC-CTT---TA----TT-AGG--T-TAAGTGA-TACAT-TTTT-----TCT----TTCAACACCTCAAGTC-AAGTAAG-ATTACCCGCTGAAC-TT-AAGCATATCAATAAGCGGAGGAAAAGAGACTAAC-AAGGATTCCC-TTAG-TAAC-GGCGAGTGAGGTGGGAATAGCTCAAA-TTTTAAATCGCCAAGG-TTTCACTTTGGTGAGTTGTAATTTGAAGAAAGT-G-TTTGGAT-ATTCTGGTTTGACC-CAAATCCTCTGGGA--TGAGGTATCAT-AG-AGGGTGAGAATCCCGTTCATGGTTGAACCTTG---GAT--GTCACTTAATTCACTTTCTAAGAGTCGAGTTG-TTTGGGATTGCAGCTCAAAATGGGTGGTAAATTTCACCTAAGGCTAAATATGTGCAAGAGACCGATAGCGAACAAGTACCGT-GAGGGAAAGA--TGAAAAGAACTTTGAAAAGAGAGTTAAATAGTACGTGAAATTGTTGAAAGGGAAACGATTGAAGTCAGTCATGCTAATAAGGAATCAACTTGATGGGT-------CGTTCC----------------------------------CCCG-GGAGCAATCTTTCTTGTGCACTTCCTT--GTTCGGCAGGTTAGCATCAA-TTTCGGTTGTCATCAAAAAATT-GAGGGAATGTGACTTC---TTTC--GGGAAGTG-TTATAGACCTTGAT-ATATGTGGTGATCGGGATTGAGGGTTGCAGCGGATGCT-TTTG------GGCTAGTCACCTAC-CTTCTGACG-GTTACCCC--GCTTTCGATAGC-TTGCTAACGATTGTGGAATTTAACAGTCTAG-AT-GTTGGAGTGATCTTATA-TTCGCTAAGGATGCTGACGTAATGGCTTTAAACGACCCGTCTTGAAACACGGACCAAGGAGTCTAAC-ATGTGTGCGAGTGTT

>FR692352_Acaulospora_scrobiculata

CCTGGTAAGCGTGAGTCATCAGCTCACGTTGATTACGTCCCT-GCCCTTTGTACACACCGCCCGTCGCTACTACCGATTGAAT-GGCTTAGTGAGACTCTCGGATTGGGTTTTAGGAACCGGCAACGGTCCCTTTCTTCTAAGAAGTTTGTCAAACTTGGTCATTTAGAGGAAGTAAAAGTCGTAACAAGGTTTCCGTAGGTGAACCTGCGGAAGGATCATTAAAAA--------------------------------TTTTAA----------------TTTTTTATTC-AAAAA-ATTTAATCTTAAT---AAATATT--GAAGTTATATG---TATAT-----------ATATCATAATATTAAGACAACTTTCAACAACGGATCTCTTGGCTCTTGCATCGATGAAGAACGCAGCGAAATGCGATAAGTAATGTGAATTGCAGAATTCCGTGAATCATCAAATCTTTGAACGCAAATTGCACTCTTT-GGTATTCCGAGGAGTATGCTTGCTTGAGGGTTGTTCAA---ATAAA-TCGT-AAAAAGAA-------ATTTTTTTTTTT-------------TGCGGACCTGAG---TTTTCCAAAG---TT--TTA-----------------------AAATTTTGGTAACTTT-AAATTTATCTTAACGATACTTTAAAGG---TATTA-TA-TTGG-AAATGT-G--ATAT-CTTGCATA------------------TTGTAA----GTT-CA-TTGACAATTATTT--TACC---AATCGTTA-TTCGTG--TTATTTACCTAA------------------------------TAC-CTT---TA----TT-AGG--T-TAAGTGA-TACAT-TTTT-----TCT----TTCAACACCTCAAGTC-AAGTAAG-ATTACCCGCTGAAC-TT-AAGCATATCAATAAGCGGAGGAAAAGAAACTAAC-AAGGATTCCC-TTAG-TAAC-GGCGAGTGAAGTGGGAATAGCTCAAA-TTTTAAATCGCCAAGG-TTT-ACTTTGGTGAGTTGTAATTTGAAGAAAGT-G-TTTGGAT-ATTCTGGTTTGATC-CAAATCCTTTGGGA--TGAGGTATCAT-AG-AGGGTGAGAATCCCGTTCATGGTTAAACCTTG---GAT--GTCACTTCATTCACTTTCTAAGAGTCGAGTTG-TTTGGGATTGCAGCTCAAAATGGGTGGTAAATTTCACCTAAGGCTAAATATGTGCAAGAGACCGATAGCGAACAAGTACCGT-GAGGGGAAGA--TGAAAAGAACTTTGAAAAGAGAGTTAAATAGTACGTGAAATTGTTGAAAGGGAAACGATTGAAGTCAGTCATGCTAATAGGGAATCAACTTGATGGGT-------CGTTCC----------------------------------CCCG-GGAGCAATCTTTCTTGTGCACTTCCTT--GTTCGGCAGGTTAGCATCAA-TTTCGGTTGTCATCAAAAAATT-GAGGGAATGTGACTTC---TTTC--GGGAAGTG-TTATAGACCTTGAT-ATATGTGGTGATCGGGATTGAGGATTGCAGCGGATGCT-TTTG------GGCTAGTCACCTAC-CTTCTGACG-GTTACCCC--GCTTTCGATAGC-TTGCTAACGATTGTGGGATTTAACAGTCCAG-AT-GTTGGAGTGATCTTATA-TTCGCTAAGGATGCTGACGTAATGGCTTTAAACGACCCGTCTTGAAACACGGACCAAGGAGTCTAAC-ATATATGCGAGTGTT

>FR692354_Acaulospora_scrobiculata

CCTAGTAAGCGTGAGTCATCAGCTCACGTTGATTACGTCCCT-GCCCTTTGTACACACCGCCCGTCGCTACTACCGATTGAAT-GGCTTAGTGAGACTCTCGGATTGGGTTTTAGGAACCGGCAACGGTTCTTTTCTTCTGAGAAGTTTGTCAAACTTGGTCATTTAGAGGAAGTAAAAGTCGTAACAAGGTTTCCGTAGGTGAACCTGCGGAAGGATCATTAGAAA--------------------------------TTTTGA----------------TTTTTTATTC-AAAAA-ATTTAATCTTAATA-TAAATATT--TAAG-TATATG---TATAT-----------ATATCATAATATTAAGACAACTTTCAACAACGGATCTCTTGGCTCTTGCATCGATGAAGAACGCAGCGAAATGCGATAAGTAATGTGAATTGCAGAATTCCGTGAATCATCAAATCTTTGAACGCAAATTGCACTCTTT-GGTATTCCGAGGAGTATGCTTGCTTGAGGGTTGTTCAA---ATAAA-TCGT-AAAAA---------------TTTTCTT-------------TGCGGACCTGAG---TTTTCCAAAG---TT--TCA-----------------------AAATTTTGGTAACTTT-AAATTTATCTTAACGATACTTTAAAGG---TATTA-TA-TTGG-AAATGT-G--ATAC-CTTGCATA------------------TTGTAA----GTT-CA-TTGACAATTATTT--TACC---AATCGTTA-TTCGTG--TTATTTACCTAA------------------------------TAC-CT----------TT-AGG--T-TAAGTGA-TACAC-TTTT-----TCT----TTCAACACCTCAAGTC-AAGTAAG-ATTACCCGCTGAAC-TT-AAGCATATCAATAAGCGGAGGAAAAGAAACTAAC-AAGGATTCCC-TTAG-TAAC-GGCGAGTGAAGTGGGAATAGCTCAAA-TTTTAAATCGCCAAGG-TTT-ACTTTGGTGAGTTGTAATTTGAAGAAAGT-G-TTTGGAT-ATTCTGGTTTGATC-CAAATCCTTTGGGA--TGAGGTATCAT-AG-AGGGTGAGAATCCCGTTCATGATTGAACCTTG---GAC--GTCACTTCATTCACTTTCTAAGAGTCGAGTTG-TTTGGGATTGCAGCTCAAAATGGGTGGTAAATTTCACCTAAGGCTAAATATGTGCAAGAGGCCGATAGCGAACAAGTACCGT-GAGGGAAAGA--TGAAAAGAACTTTGAAAAGAGAGTTAAATAGTACGTGAAATTGTTGAAAGGGAAACGATTGAAGTCAGTCATGCTAATAGGGAATCAACTTGATGGGT-------CGTTCC----------------------------------CCCG-GGAGCAATCTTTCTTGTGCACTTCCTT--GTTCGGCAGGTTAGCATCAA-TTTCGGTTGTCATCAAAAAATT-GAGGGAATGTGACTTC---TTTC--GGGAAGTG-TTATAGACCTTGAT-ATATGTGGTGATCGGGATTGAGGATTGCAGCGGATGCT-TTTG------GGCTAGTCACCTAC-CTCCTGACG-GTTACCCC--GCTTTCGATAGC-TTGCTAACGATTGTGGGATTTAACAGTCTAG-AT-GTTGGAGTGATCTTATA-TTCGCTAAGGATGCTGACGTAATGGCTTTAAACGACCCGTCTTGAAACACGGACCAAGGAGTCTAAC-ATGTATGCGAGTGTT

>AF378440_Acaulospora_tuberculata

-------------------------------------------------------------------------------------------------------------------------------------------------------------------------------------------------------------------------------------------------------------------------------------------------------------------------------------------------------------------------------------------------------------------------------------------------------------------------------------------------------------------------------------------------------------------------------------------------------------------------------------------------------------------------------------------------------------------------------------------------------------------------------------------------------------------------------------------------------------------------------------------------------------------------------------------------------------------------------------------------------------------------------------------------------------------------------------------------------------------------------------------------------------------------------------------------------------------------------------------------------------------------------------------------------------------------------------------GGAAAGA--TGAAAAGAACTTTGAAAAGAGAGTTAAATAGTACGTGAAATTGTTGAAAGGGAAACGATTGAAGTCAGTCATGCTAATAGGGAATCAACTTGATGGGT-------CGTTCC----------------------------------CCCG-GGAGCAATCTTTCTTGTGCACTTCCTT--GTTCGGCAGGTTAGCATCAA-TTTCGGTTGTCATCAAAAAATT-GAGGGAATGTGACTTC---TTTC--GGGAAGTG-TTATAGACCTTAAT-ATATGTGATAACCGGGATTGAGGATTGCAGCGGATGCT-TTTG------GGCTAGTCACCTAC-CTTCTGACG-GTTACCTC--GCTTTCGATAGC-TTGCTAACGATTGTGGGATTCAACAGTCTAG-AT-GTTGGAGTGATCTTATA-TTCGCTAAGGATGCTGACGTAATGGCTTTAAACGACCCGTCTTGAAACACGGACCA---------------------------

>FR692350_Acaulospora_scrobiculata

CCTAGTAAGCGTGAGTCATCAGCTCACGTTGATTACGTCCCT-GCCCTTTGTACACACCGCCCGTCGCTACTACCGATTGAAT-GGCTTAGTGAGACTCTCGGATTGGG-TTTAGGAACCGGCAACGGTCCCTTTCTTCTGAGAAGTTTGTCAAACTTGGTCATTTAGAGGAAGTAAAAGTCGTAACAAGGTTTCCGTAGGTGAACCTGCGGAAGGATCATTAGAAA--------------------------------TTTTGA----------------TTTTTTATTC-AAAAA-ATTTAATCTTAATA-AATAT-TT--TATG-CATATG---TATAT-----------ATATCATAATATTAAGACAACTTTCAACAACGGATCTCTTGGCTCTTGCATCGATGAAGAACGCAGCGAAATGCGATAAGTAATGTGAATTGCAGAATTCCGTGAATCATCAAATCTTTGAACGCAAATTGCACTCTTT-GGTATTCCGAGGAGTATGCTTGCTTGAGGGTTGTTCAA---ATAAA-TCGT-AAA------------------TTTTTT-------------TGCGGACCTGAG---TTTTCC-------------A-----------------------AAATTTTGGTAACTTT-AAATTCATCTTAACGATACTTTAAAAG--GTATTA-TA-TTGG-AAATGT-G--ATAT-CTTGCATA------------------TTGCAA----GTT-CA-TTGACAATTATTT--TACC---AATCGTTATTTCGTG--TTATTTACTTAA------------------------------TGC-CCTTTATT----TT-AAG--C-TAAGTGA-CACAATTTTT-----TTT----TTCAACACCTCAAGTC-AAGTAAG-ATTACCCGCTGAAC-TT-AAGCATATCAATAAGCGGAGGAAAAGAAACTAAC-AAGGATTCCC-TTAG-TAAC-GGCGAGTGAAGTGGGAATAGCTCAAA-TTTTAAATCGCCAAGG-TTT-ACTTTGGTGAGTTGTAATTTGAAGAAAGT-G-TTTGGAT-ATTCTGGTTTGATC-CAAATCCTTTGGGA--TGAGGTATCAT-AG-AGGGTGAGAATCCCGTTCATGGTTGAACCTTG---GAT--GTCACTTCATTCACTTTCCAAGAGTCGAGTTG-TTTGGGATCGCAGCTCAAAACGGGTGGTAAATTTCACCTAAGGCTAAATATGTGCAAGAGACCGATAGCGAACAAGTACCGT-GAGGGAAAGA--TGAAAAGAACTTTGAAAAGAGAGTTAAATAGTACGTGAAATTGTTGAAAGGGAAACGATTGAAGTCAGTCATGCTAATAGGGAATCAACTTGATGGGT-------CGTTCC----------------------------------CCAG-GGAGCAATCTTTCTTGTGCACTTCCTT--GTTCGGCAGGTTAGCATCAA-TTTCGGTTGTCATCGAAAAATTGGGGGGAATGTGACTTC---TTTC--GGGAAGTG-TTATAGACCTTAGT-ATATGTGATAACCGGGATTGAGGATTGCAGCGGATGCT-TTTG------GGCTAGTCACCTAT-CTTCTGACG-GTTACCCC--GCTTTCGATAGC-TTGCTAACGACTGTGGGATTTAACAGTCTAG-AT-GTTGGAGTGATCTTATA-TTCGCTAAGGATGCTGACGTAATGGCTTTAAACGACCCGTCTTGAAACACGGACCAAGGAGTCTAAC-ATGTATGCGAGTGTT

>FJ461799_Acaulospora_tuberculata

-----------------------------------------------------------------------------------------------------------------------------------------------------------------------------------------------------------------------------------------------------------------------------------------------------------------------------------------------------------------------------------------------------------------------------------------------------------------------------------------------------------------------------------------------------------------------------------------------------------------------------------------------------------------------------------------------------------------------------------------------------------------------------------------------------------------------------------------------------------------------------------------------------------------------------------------------------------------------------------------------------------CTAAC-AAGGATTCCC-TTAG-TAAC-GGCGAGTGAAGTGGGAATAGCTCAAA-TTTTAAATCGCCAAGG-TTT-ACTTTGGTGAGTTGTAATTTGAAGAAAGT-G-TTTAGAT-ATTCTGGTTTGATC-TAAATCCTTTGGGA--TGAGGTATCAT-AG-AGGGTGAGGATCCCGTTTATGGTTAAACCTTG---GAT--GTCATTTCATTCACTTTCTAAGAGTCGAGTTG-TTTGGGATTGCAGCTCAAAATGGGTGGTAAATTTCACCTAAGGCTAAATATGTGCAAGAGACCGATAGCGAACAAGTACCGT-AAGGGAAAGA--TGAAAAGAACTTTGAAAAGAGAGTTAAATAGTACGTGAAATTGTTGAAAGGGAAACGATTGAAGTCAGTCATGCTAATAGGGAATCAACTTGATGGGT-------CGTTCC----------------------------------CCCG-GGAGCAATCTTTCTTGTGCACTTCCTT--GTTCGGCAGGTTAGCATCAA-TTTCGGTTGTCATCAAAAAATT-GAGGGAATGTGACTTC---TTTC--GGGAAGTG-TTATAGACCTTGAT-ATATGTGATAACCGGGATTGAGGATTGCAGCGGATGCT-TTTG------GGCTAGTCACCTGC-CTTCTGACG-GTTACCTC--GCTTTCGATAGC-TCGCTAACGATTGTGGGATTTAACAGTCTAG-AT-GTTGGAGTGATCTTATA-TTCGCTAAGGATGC---------------------------------------------------------------------

>FR869691_Acaulospora_minuta

CCTAGTAAGCGTGAGTCATCAGCTCACGTTGATTACGTCCCT-GCCCTTTGTACACACCGCCCGTCGCTACTACCGATTGAAT-GGCTTAGTGAGACTCTCGGATTGGGTTTTAAGAACCGGCAACGGATCTTTTCTTCTGAGAAGTTTGTCAAACTTGGTCATTTAGAGGAAGTAAAAGTCGTAACAAGGTTTCCGTAGGTGAACCTGCGGAAGGATCATTGGAAAA-------------------------------TTTTGA---------------TTTTTTTATTC-AAAAA-ATTTAATCTTAAT---AAATATT--TA---TGTATA---TGTAT-----------ATATCATAATATTAAGACAACTTTCAACAACGGATCTCTTGGCTCTTGCATCGATGAAGAACGCAGCGAAATGCGATAAGTAATGTGAATTGCAGAATTCCGTGAATCATCAAATCTTTGAACGCAAATTGCACTCTTT-GGTATTCCGAGGAGTATGCTTGTTTGAGGGTTGTTCAA---ATAAA-TCGT-GAAAAAAA------------TTTTTTT-------------TGCGGACCTGAG---TTTTCCAAAG---TT--TTA-----------------------AAATTTTGGTAACTTT-AAATTTATCTTAACGATACTTGAAG-GTATTATTA-TA-TTGG-AAATGT-T--ATAT-CTCGCATA------------------TTGCAA----GTT-CA-TTGACAATTATTT--TACC---AGTCGTTA-TTCGTG--TTATTTACCTAA------------------------------TAC-ATT---TA----TT-AGG--T-TAAGTAA-TATAT-TTTT-----TCT----TTCAACACCTCAAGTC-AAGTAAG-ATTACCCGCTGAAC-TT-AAGCATATCAATAAGCGGAGGAAAAGAAACTAAC-AAAGATTCCC-TTAG-TAAC-GGCGAGTGAAGTGGGAATAGCTCAAA-TTTTAAATCACCAAGG-TTTCACTTTGGCGAGTTGTAATTTGAAGAAAGT-G-TTTAGAT-ATTCTGGTTTGATC-TAAATCCTTTGGGA--TGAGGTATCAT-AG-AGGGTGAGAATCCCGTTCATGGTTGAACCTTG---GAT--GTCACTTAATTCACTTTCCAAGAGTCGAGTTG-TTTGGGATTGCAGCTCAAAATGGGTGGTAAATTTCACCTAAGGCTAAATATATGCAAGAGACCGATAGCGAACAAGTACCGT-GAGGGAAAGA--TGAAAAGAACTTTGAAAAGAGAGTTAAATAGTACGTGAAATTGTTGAAAGGGAAACGATTGAAGTCAGTCATGCTAATAGGGAATCAACTTGATGGGT-------CGTTCC----------------------------------CCCG-GGAGCAATCTTTCTTGTGCACTTCCTT--GTTCGGCAGGTTAGCATCAA-TTTCGGTTGTCATCAAAAAATT-GAGGTAATGTGACTTC---TTTC--GGGAGGTG-TTATAGACCTTGAT-ATATGTGGTGGTCGGGATTGAGGATTGCAGCGGATGCT-TTTG------GGCTAGTCACCCAC-CTTCTGACG-GTTACCCC--GCTTTCGATAGC-TTGCTAACGATTGTGGAATTTAACAGTCTAG-AT-GTTGGAGTGATCTTATA-TTCGCTAAGGATGCTGACGTAATGGCTTTAAACGACCCGTCTTGAAACACGGACCAAGGAGTCTAAC-ATATGTGCGAGTGTT

>FR750156_Acaulospora_spinosa

-------------------------------ATTACGTCCCT-GCCCTTTGTACACACCGCCCGTCGCTACTACCGATTGAAT-GGCTTAGTGAGACTCTCGGATTGGGTTTTAAGAACCGGCAACGGATCTTTTCTTCTAAGAAGTTTGTCAAACTTGGTCATTTAGAGGAAGTAAAAGTCGTAACAAGGTTTCCGTAGGTGAACCTGCGGAAGGATCATTAGAAA--------------------------------TTTTGAT-T-----------TTTTTTTTATTC-AAAAA-ATTTAATCTTAATA-AAAATATT--TCAA-TATATG---TATAT-----------ATATCATAATATTAAGACAACTTTCAACAACGGATCTCTTGGCTCTTGCATCGATGAAGAACGCAGCGAAATGCGATAAGTAATGTGAATTGCAGAATTCCGTGAATCATCAAATCTTTGAACGCAAATTGCACTCTTT-GGTATTCCGAAGAGTATGCTTGCTTGAGGGTTGTTCAA---ATAAA-TCGT-AAA-------------------TTTTT-------------TGCGGACCTGAG---TTTTCCAAAG---TT--TTA-----------------------AAATTTTGGTAACTTT-AAATTTATCTTAACGATACTTTAAAGGTATTATTA-TA-TTGA-AAATGT-T--ATAT-CTTGCATA------------------TCGTAA----GTT-CA-TTGACAATTATTT--TACC---AATCGTTATTTCGCG--TTATTTACTTAATACCTTATTAGGTAATACCTTATTAGGTAATAC-CCTT--TA----TT-AAG--C-TAAATAA-CCCAA-CTTT-------T----TTCAACACCTCAAGTC-AAGTAAG-ATTACCCGCTGAAC-TT-AAGCATATCAATAAGCGGAGGAAAAGAAACTAAT-AAGGATTCCC-TTAG-TAAC-GGCGAGTGAAGTGGGAATAGCTCAAA-TTTTAAATCGCCAAGG-TTTCACTTTGGTGAGTTGTAATTTGAAGAAAGT-G-TTTAGAT-ATTCTGGTTTAATC-TAAATCCTTTGGGA--TGAGGTATCAT-AG-AGGGTGAGAATCCCGTTTATGGTTAAACCGTG---GAT--GTCACTTAATTCACTTTCTAAGAGTCGAGTTG-TTTGGGATTGCAGCTCAAAATGGGTGGTAAATTTCACCTAAGGCTAAATATGTGCAAGAGACCGATAGCGAACAAGTACCGT-GAGGGAAAGA--TGAAAAGAACTTTGAAAAGAGAGTTAAATAGTACGTGAAATTGTTGAAAGGGAAACGATTGAAGTCAGTCATGCTAATAGGGAATCAACTTGATGGTT-------CGTTCC----------------------------------CCCG-GGAGTGATCTTTCTTGTGCACTTCCTT--GTTTGGCAAGTTAGCATCAA-TTTCGGTTGTCATCAAAAAATT-GAGGGAATGTAACTTC---TTTC--GGGAAGTG-TTATAGACCTTGATGATATGTGATGGTCGGGATTGAGGATTGCAGCGGATGCT-TTTG------GGCTAGTCACCTAC-CTTCTGACG-GTTACCTC--GCTTTCGATAGC-TTGCTAACGATTGTGGGATTCAACAGTCTAG-AT-GTTAGAGTGATCTTATA-TTCGCTAAGGATGCTGACGTAATGGCTTTAAACGACCCGTCTTGAAACACGGACCA---------------------------

>FR750152_Acaulospora_spinosa

-------------------------------ATTACGTCCCT-GCCCTTTGTACACACCGCCCGTCGCTACTACCGATTGAAT-GGCTTAGTGAGACTCTCGGATTGGGTTTTAAGAACCGGCAACGGATCTTTTCTTCTAAGAAGTTTGTCAAACTTGGTCATTTAGAGGAAGTAAAAGTCGTAACAAGGTTTCCGTAGGTGAACCTGCGGAAGGATCATTAGAAA--------------------------------TTTTGAT--------------TTTTTTTATTCAAAAAA-ATTTAATCTTAATA-AAAATATT--TCAG-TATATG---TATAT-----------ATATCATAATATTAAGACAACTTTCAACAACGGATCTCTTGGCTCTTGCATCGATGAAGAACGCAGCGAAATGCGATAAGTAATGTGAATTGCAGAATTCCGTGAATCATCAAATCTTTGAACGCAAATTGCACTCTTT-GGTATTCCGAAGAGTATGCTTGCTTGAGGGTTGTTCAA---ATAAA-TCGT-AAA-------------------TTTTT-------------TGCGGACCTGAG---TTTTCCAGAG---TT--TTA-----------------------AAATTTTGGTAACTTT-AAATTTATCTTAACGATACTTTAAAGGTATTATTA-TA-TTGG-AAATGT-T--ATAT-CTTACATA------------------TCGTAA----GTT-CA-TTGACAATTATTT--TACC---ATTCGTTATTTCGCG--TTATTTACTTAA------------------------------TAC-CCTT--TA----TT-AAG--C-TAAATGA-CCCAA-TTTT-------T----TTCAACACCTCAAGTC-AAGTAAG-ATTACCCGCTGAAC-TT-AAGCATATCAATAAGCGGAGGAAAAGAAACTAAC-AAGGATTCCC-TTAG-TAAC-GGCGAGTGAAGTGGGAATAGCTCAAA-TTTTAAATCGCCAAGG-TTTCACTTTGGTGAGTTGTAATTTGAAGAAAGT-G-TTTAGAT-ATTCTGGTTTGATC-TAAATCCTTTGGGA--TGAGGTATCAT-AG-AGGGTGAGAATCCCGTTTATGGTTAAACCGTG---GAT--GTCACTTCATTCACTTTCCAAGAGTCGAGTTG-TTTGGGATTGCAGCTCAAAATGGGTGGTAAATTTCACCTAAGGCTAAATATGTGCAAGAGACCGATAGCGAACAAGTACCGT-GAGGGAAAGA--TGAAAAGAACTTTGAAAAGAGAGTTAAATAGTACGTGAAATTGTTGAAAGGGAAACGATTGAAGTCAGTCATGCTAATAGGGAATCAACTTGATGGAT-------CGTTCC----------------------------------CCCG-GGAGCGATCTTTCTTGTGCACTTCCTT--GTTTGGCAAGTTAGCATCAA-TTTCGGTTGTCATCAAAAAATT-GAGGGAATGTGACTTC---TTTC--GGGAAGTG-TTATAGACCTTAGTTATATGTGATAACCGGGATTGAGGATTGCAGCGGATGCT-TTTG------GGCTAGTCACCTAC-CTTCTGACG-GTTACCCT--GCTTTCGATAGC-TTGCTAACGATTGTGGGATTCAACAGTCTAG-AT-TTTAGAGTGATCTTATA-TTCGCTAAGGATGCTGACGTAATGGCTTTAAACGACCCGTCTTGAAACACGGACCA---------------------------

>KM057064_Acaulospora_reducta

-----------------------------------------------------------------------------------------------------------------------------------------------------------------------------------------------------------------------------------------------------------------------------------------------ATTCAAAAAA-ATTTAATCTTAAT--AAAATATT--GAAG-TATATG---TATAT-----------ATATCATAATATTAAGACAACTTTCAACAACGGATCTCTTGGCTCTTGCATTGATGAAGAACGCAGCGAAATGCGATAAGTAATGTGAATTGCAGAATTCCGTGAATCATCAAATCTTTGAACGCAAATTGCACTCTTT-GGTATTCCGAGGAGTATGCTTGCTTGAGGGTTGTTCAA---ATAGA-TCGT-AAA------------------TTTTTT-------------TGCGGACCTGAG---TTTTCCAAAG---TT--TTA-----------------------AAATTTTGGTAACTTT-AAATTTATCTTAACGATACTTTTAAAG--GTATTA-TA-TTGG-AAATGT-G--ATAT-CTTGCATA------------------TTGCGA----GTT-CA-TTGACAATTACTC--TACC---AATCGTTA-TTCGTA--TTATTTACCTAA------------------------------TAC-CTT---TA----TT-AGG--T-TAAGTGA-TACAT-TTTT-----TTCTT--TTCAACAC----------------------------------------------------------------------------------------------------------------------------------------------------------------------------------------------------------------------------------------------------------------------------------------------------------------------------------------------------------------------------------------------------------------------------------------------------------------------------------------------------------------------------------------------------------------------------------------------------------------------------------------------------------------------------------------------------------------------------------------------------------------------------------------------------------------------------------------------------------------------------------------------------------------------------------

>KM057063_Acaulospora_reducta

----------------------------------------------------------------------------------------------------------------------------------------------------------------------------------------------------------------------------------------------------------------------------------------------------------ATTTAATCTTAAT--AAAATATT--GAAG-TATATG---TATAT-----------ATATCATAATATTAAGACAACTTTCAACAACGGACCTCTTGGCTCTTGCATCGATGAAGAACGCAGCGAAATGCGATAAGTAATGTGAATTGCAGAATTCCGTGAATCATCAAATCTTTGAACGCAAATTGCACTCTTT-GGTATTCCGAGGAGTATGCTTGCTTGAGGGTTGTTCAA---ATAAA-TCGT-AAA------------------TTTTTT-------------TGCGGACCTGAG---TTTTCCAAAG---TT--TTA-----------------------GAATTTTGGTAGCTTT-AAATTTATCTTAACGATACTTTTAAAG--GTATTA-TA-TTGG-AAATGT-G--ATAT-CTTGCATA------------------TTGCGA----GTT-CA-TTGACAATTATTT--TACC---AATCGTTA-TTCGTA--TTATTTACCTAA------------------------------TAC-CTT---TA----TT-AGG--T-TAAGTGA-TACAT-TTTT-----TTCTT--TTCAACACCTCAAGTC-AAGTAAG-ATTACCCGCTGAAC-TT-AAGCATATCAATAAGCGGAGGAAAAGAAACTAAC-AAGGATTCCC-TTAG-TAAC-GGCGAGTGAAGTGGGAATAGCTCAAA-TTTTAAATCGCCAAGG-TTT-TCTTTGGTGAGTTGTAATTTGAAGAAAGT-G-TTTGGAT-GTTCTGGTTT-------------------------------------------------------------------------------------------------------------------------------------------------------------------------------------------------------------------------------------------------------------------------------------------------------------------------------------------------------------------------------------------------------------------------------------------------------------------------------------------------------------------------------------------------------------------------------------------------------------------------------------------------------------------------------------------------------------------------------------

>KX355819_Sacculospora_baltica

CCTAGTAAGCGTGAGTCATCAGCTCACGTTGATTACGTCCCT-GCCCTTTGTACACACCGCCCGTCGCTACTACCGATTGAAT-GGCTTAGTGAGACTCTCGGATTGAG-ATTAAGAACCGGAAACGGATCTTGTTCTTCGAGAAGTTAGTCAAACTTGGTCATTTAGAGGAAGTAAAAGTCGTAACAAGGTTTCTGTAGGTGAACCTGCAGAAGGATCATTAGAAAA------------------------------------------------------TTTGTAATC-AAAAA-CCCAAACCATT--TATATATAATT-AAAACTTTTG----AAAATT---------CTAAAAGAAAACTTTAGATAACTTTCAACAACGGATCTCTTGGCTCTCGCATCGATGAAGAACGCAGCGAAATGCGATAAGTAATGTGAATTGCAG-ATTCCGTGAATCATCGAATCTTTGAACGCAAATTGCACTCCTT-GGTATTCCGAGGAGTATGCTTGCTTGAGTGTCCGTTTAA--TAAACATCGC-GATTAA---------ATGTTTAACATT-------------CGCGGATTTGGG---TCATCCGGAT--TCTAACTA----------------------TTGGATTTGGAGMCCTT-AAATT------AAAAATCATTTGG------TATGATCA------AAACCGG--GCTATTATTATGTTAATGTTGTGTATTAATGTATTAAAGT-AGAT-CG-TTGACAATCTC-----ACCAGAATTTTTTAC------------------------------------------------------------AA----TC-AAG-----------------------------------TTTGGACCTCAAGTC-AAGTAAG-GATACCCGCTGAAC-TT-AAGCATATCAATAAGCGGAGGAAAAGAAACTAAC-AAGGATTCCC-TTAG-TAAC-GGCGAGTGAAGCGGGAAAAGCTCAAA-TTTTAAATCTACCTGG-TTCTTCCAAGTCGAGTTGTAATTTGAAGAAACA-C-TTTCGGA-ATTCCGGTCTGGTC-TAAATCCTTTGGAA--TGAGGTATCATGGG-AGGGTGAGAATCCCGTTCCTGATCAGGCATCCGTGAGT-TTCAATGTGAAGTGTTTTCTAAGAGTCGAGTTG-TTTGGGATTGCAGCTCAAAATGGGTGGTATATCTCACCTAAGGCTAAATATCTGCGAGAGACCGATAGCGAACAAGTACCGT-GAGGGAAAGA--TGAAAAGAACTTTGAAAAGAGAGTTAAACAGTACGTGAAATTGTTGAAAGGGAAACGATTGAAGTCAGTCATGCCGGCTGGGAATCAACCTCAAG---------------------------------------------------------TTTTTTTCTTTAGGTGTACTTCCTT---TTCGGCAAGTTAGCATCGA-TTTTATTGGTCATAAAAGAATC-A-GGTAATGTGACTCC---TC------GGAGTGTTTATAGACCTTGGT-GGATGTGACCTGTGAGATCGAGGTTTGCAGCGGACCGC-CCTCTCGAGGGTTTAGTCGCCTAG-CCTCTGATTCGTTATCCTG-GTATTCTTCAGC-TTGTCTGTGGGTTTTAGGTTAAACGTTCAAA-AG-GTTAGAACGATCTTAAGTTTCGCTAAGGATGCTGACGTAATGGCTTTAAACGACCCGTCTTGAAACACGGACCAAGGAGTCTAAC-ATGTGTGCGAGTGTT

>KX355821_Sacculospora_baltica

CCTAGTAAGCGTGAGTCATCAGCTCACGTTGATTACGTCCCT-GCCCTTTGTACACACCGCCCGTCGCTACTACCGATTGAAT-GGCTTAGTGAGACTCTCGGATTGAG-ATTAAGAACCGGAAACGGATCTTGTTCTTCGAGAAGTTAGTCAAACTTGGTCATTTAGAGGAAGTAAAAGTCGTAACAAGGTTTCTGTAGGTGAACCTGCAGAAGGATCATTAGAAAA------------------------------------------------------TTTGTAATC-AAAAA-CCCAAACCATTTATATATATAATT-AAAAGTTTCG----AAAATT---------CTAAAAGAAAACTTTAGATAACTTTCAACAACGGATCTCTTGGCTCTCGCATCGATGAAGAACGCAGCGAAATGCGATAAGTAATGTGAATTGCAG-ATTCCGTGAATCATCGAATCTTTGAACGCAAATTGCACTCCTT-GGTATTCCGAGGAGTATGCTTGCTTGAGTGTCCGTTTAA--TAAATATCGC-GATTAA---------ATGTTTAACATT-------------CGCGGATTTGGG---TCATCC-GAT--TCTAATTATTTGAGTCCATTCGATTCTCGTTTGGATTTGGAGACCTT-AAATT------AAAAATCATTTGG------TATGATTA------AAACGG---GCTATTATTATGTTAATGTTGTGTATTAATGTATAAAGGT-AGAT-CG-TTGACAATCTC-----ACCAGAATTTTTTAC------------------------------------------------------------AA----TC-GAG----------------------------------TTTTGGACCTCAAGTC-AAGTAAG-GATACCCGCTGAAC-TT-AAGCATATCAATAAGCGGAGGAAAAGAAACTAAC-AAGGATTCCC-TTAG-TAAC-GGCGAGTGAAGCGGGAAAAGCTCAAA-TTTTAAATCTACTTGG-TTCTTCTAAGTCGAGTTGTAATTTGAAGAAACA-C-TTTCGGA-ATTCCGGTCTGGTC-TAAATCCTTTGGAA--TGAGGTATCAT-GG-AGGGTGAGAATCCCGTTCCTGATCAGGCATCCGTGAGT-TTCAATGTGAAGTGTTTTCTAAGAGTCGAGTTG-TTTGGGATTGCAGCTCAAAATGGGTGGTATATCTCACCTAAGGCTAAATATCTGCGAGAGACCGATAGCGAACAAGTACCGT-GAGGGAAAGA--TGAAAAGAACTTTGAAAAGAGAGTTAAACAGTACGTGAAATTGTTGAAAGGGAAACGATTGAAGTCAGTCATGCCGGCTGGGAATCAACCTCAAG---------------------------------------------------------TTTTTTTCTTTAGGTGTACTTCCTT---TTCGGCAAGTTAGCATCGA-TTTTATTGGTCATAAAAGAATC-AGGGTAATGTGACTCC---TC------GGAGTGTTTATAGACCTTGGT-GGATGTGACCTGTGAGATCGAGGTTTGCAGCGGACCGC-CCTCTCGAGGGTTTAGTCGTCTAG-CCTCTGATTCGTTATCCTG-GTATTCTTCAGC-TTGTCTGTGGGTTTTAGGTTAAACGTTCAAA-AG-GTTAGAACGATCTTAAGTTTCGCTAAGGATGTTGACGTAATGGCTTTAAACGACCCGTCTTGAAACACGGACCAAGGAGTCTAAC-ATATGTGCGAGTGT-

>KX355818_Sacculospora_baltica

CCTAGTAAGCGTGAGTCATCAGCTCACGTTGATTACGTCCCT-GCCCTTTGTACACACCGCCCGTCGCTACTACCGATTGAAT-GGCTTAGTGAGACTCTCGGATTGAG-ATTAAGAACCGGAAACGGATCTTGTTCTTCGAGAAGTTAGTCAAACTTGGTCATTTAGAGGAAGTAAAAGTCGTAACAAGGTTTCTGTAGGTGAACCTGCAGAAGGATCATTAGAAAA------------------------------------------------------TTTGTAATC-AAAAA-CCCAAACCATT--TATATATAATT-AAAAGTTTC------------------------AAAAAAACTTTAGATAACTTTCAACAACGGATCTCTTGGCTCTCGCATCGATGAAGAACGCAGCGAAATGCGATAAGTAATGTGAATTGCAG-ATTCCGTGAATCATCGAATCTTTGAACGCAAATTGCACTCCTT-GGTATTCCGAGGAGTATGCTTGCTTGAGTGTCCGTTTAA--TAAAAATCGSGGATTGA---------ATGTTTGACATT-------------CGCGGATTTGGG---TTATCC-GAT--TCTAATTATTTGAGTCTATTCGATTCTCGTTTGGATTTGGAGACCTT-AAATT------AAAAATCATTTGG------TATGATTA------AAACGGGCTATTATTATTATGTTAATGTTGTGTATTAATGTATTAAAGT-AGAT-CG-TTGACAATCTC-----ACCAGAATTTTTTAC------------------------------------------------------------AA----TC-AAG-----------------------------------TTTGGACCTCAAGTC-AAGTAAG-GATACCCGCTGAAC-TT-AAGCATATCAATAAGCGGAGGAAAAGAAACTAAC-AAGGATTCCC-TTAG-TAAC-GGCGAGTGAAGCGGGAAAAGCTCAAA-TTTTAAATCTACCTGG-TTCTTCCAAGTCGAGTTGTAATTTGAAGAAACA-C-TTTCGGA-ATTCCGGTCTGGTC-TAAATCCTTTGGAA--TGAGGTATCAT-GG-AGGGTGAGAATCCCGTTCCTGATCAGGCATCCGTGAGT-TTCAATGTGAAGTGTTTTCTAAGAGTCGAGTTG-TTTGGGATTGCAGCTCAAAATGGGTGGTATATCTCACCTAAGGCTAAATATCTGCGAGAGACCGATAGCGAACAAGTACCGT-GAGGGAAAGA--TGAAAAGAACTTTGAAAAGAGAGTTAAACAGTACGTGAAATTGTTGAAAGGGAAACGATTGAAGTCAGTCATGCCGGCTGGGAATCAACCTCAAG--------------------------------------------------------TTTTTTTTCTTTAGGTGTACTTCCTT---TTCGGCAAGTTAGCATCGA-TTTTATCGGTCATAAAAGAATC-AGGGTAATGTGACTCC---TC------GGAGTGTTTATAGACCTTGGT-GGATGTGACCTGTGAGATCGAGGTTTGCAGCGGACCGC-CCTCTCGAGGGTTTAGTCGCCTAG-CCTCTGATTCGTTATCCTG-GTATTCTTCAGC-TTGTCTGTGGGTTTTAGGTTAAACGTTCAAA-AG-GTTAGAACGATCTTAAGTTTCGCTAAGGATGCTGACGTAATGGCTTTAAACGACCCGTCTTGAAACACGGACCAAGGAGTCTAAC-ATGTGTGCGAGTGTT

>KX345938_Sacculospora_felinovii

CCTAGTAAGCGTGAGTCATCAGCTCACGTTGATTACGTCCCT-GCCCTTTGTACACACCGCCCGTCGCTACTACCGATTGAAT-GGCTTAGTGAGACTCTCGGATTGAG-TTTGAGAACCGGAAACGGCTCTCGTTCTTCGAAAAGTTAGTCAAACTTGGTCATTTAGAGGAAGTAAAAGTCGTAACAAGGTTTCTGTAGGTGAACCTGCAGAAGGATCATTAGAAAT---------------------------------------T--------------TTTGTATTC-AAAATCTTCCCAATTCTTTAATAAAAATT-----TTTTTTA----TATATA-A-T-------TTTTTAAAATTATTAATAACTTTCAACAACGGATCTCTTGGCTCTCGCATCGATGAAGAACGCAGCGAAATGCGATAAGTAATGTGAATTGCAG-ATTCCGTGAATCATCGAATCTTTGAACGCAAATTGCACTCTTT-GGTACTCCGAGGAGTATGCTTGCTTGAGTGTCCGTTTAT--TTATTATCGC-GATTA----------ATTTTTTNTATT-------------CGCGGATTTGGG---TTATCCGAAT--------------------------------TTTTTTTTGGAGACTTT-AAATTTAAT-------TCTTTTGGT----ATACGGTCA------AAACGT---GCTATTATTATTTA------------------ATAAAAGT-ATTT-CG-TTGATGATCTT-----ACCAGAACTTTATATATTATG--TTTATCGCTTGTTA----------------------------TCT-TTTC--AAGACTTC-GAG--T-GATTAAA-CATATA--TATTA--TTCGA--GTGTTGACCTCAAGTC-AAGTAAG-GTTACCCGCTGAAC-TT-AAGCATATCAATAAGCGGAGGAAAAGAAACTAAC-AAGGATTCCC-TTAG-TAAC-GGCGAGTGAAGTGGGAAAAGCTCAAA-TTTTAAATCTATCTAG-TCCTTCTAGGTCGAATTGTAATTTGAAGAAGCA-C-TTTCGGTTGCCCTGGTCTGATC-TAAATCCTTTGGGA--TGAGGTATCAT-GG-AGGGTGAGAATCCCGTTCATGATCTGGCATTG---GGT-TACCATATGATGTGTTTTCAAAGAGTCGAGTTG-TTTGGGATTGCAGCTCAAAATGGGTGGTATATCTCACCTAAGGCTAAATATCTGCGAGAGACCGATAGCGAACAAGTACCGTGGAGGGAAAGA--TGAAAAGAACTTTGAAAAGAGAGTTAAACAGTACGTGAAATTGTTGAAAGGGAAACGATTGAAGTCAGTCATGCCAGTAATGAATCAACCTCGAG----------------------------------------------------------TTTATTCTTTAGGTGTATTTCTTTACTTTCGGCAAGTTAGCATCGA-TTTTATCGGTCATAAAATGACT-AGGGTAAGGTGTCACC---TTC----GGGTGTGTTTATAGACCTTAGT-GGATGTGATCGTTAAGATCGAGGTTTGCAGCGAACAAT-CCTCTCGGGGGTTTAGTCGTCTGA-TCTCTGATTCGTTATCCTGTCTTCTCTTCAGC-TTGTCTGTGAGTTGCAGGTTCAACGTTCAAA-AG-GTTAGAACGTTCTTAAGTTTCGCTAAGGATGCTGACGTAATGGCTTTAAACGACCCGTCTTGAAACACGGACCAAGGAGTCTAAC-ATATGTGCGAGTGTT

>KX345939_Sacculospora_felinovii

CCTAGTAAGCGTGAGTCATCAGCTCACGTTGATTACGTCCCT-GCCCTTTGTACACACCGCCCGTCGCTACTACCGATTGAAT-GGCTTAGTGAGACTCTCGGATTGAG-TTTGAGAACCGGAAACGGCTCTCGTTCTTCGAAAAGTTAGTCAAACTTGGTCATTTAGAGGAAGTAAAAGTCGTAACAAGGTTTCTGTAGGTGAACCTGCAGAAGGATCATTAGAAAT---------------------------------------T--------------TTTGTATTC-AAAATCTTCCCAATCCTTTAATAAAAATT-----TTTTTTA----TATATA-ATT-------TTTTTAAAATTATTAATAACTTTCAACAACGGATCTCTTGGCTCTCGCATCGATGAAGAACGCAGCGAAATGCGATAAGTAATGTGAATTGCAG-ATTCCGTGAATCATCGAATCTTTGAACGCAAATTGCACTCTTT-GGTATTCCGAGGAGTATGCTTGCTTGAGTGTCCATTTA---TTATTATCGC-GATTA----------ATTTTTNTTATT-------------CGCGGATTTGGG---TTATCCGAA----------------------------------TTTTTTTGGAGACTTTAAAATTTAAT-------TCTTTTGGTA-TAATACGGTCA------AAACGT---GCTATTATTATTTA------------------ATAAAAGT-ATTT-CG-TTGATGATCTT-----ACCATAACTTTATATACTATG--TTTATCGCTTGTTA----------------------------TCT-TTTC--AAGAC-TC-GAG--T-GATTAAA-CATATATGTATAT--TTCGA--GTGTTGACCTCAAGTC-AAGTAAG-GTTACCCGCTGAAC-TT-AAGCATATCAATAAGCGGAGGAAAAGAAACTAAC-AAGGATTCCC-TTAG-TAAC-GGCGAGTGAAGTGGGAAAAGCTCAAA-TTTTAAATCTATCTAG-TCCTTCTAGGTCGAATTGTAATTTGAAGAAGCA-C-TTTCGGTTGCCCTGGTCTGATC-TAAATCCTTTGGGA--TGAGGTATCAT-GG-AGGGTGAGAATCCCGTTCATGATCTGGCATTG---GGT-TACCATATGATGTGTTTTCAAAGAGTCGAGTTG-TTTGGGATTGCAGCTCAAAATGGGTGGTATATCTCACCTAAGGCTAAATATCTGCGAGAGACCGATAGCGAACAAGTACCGT-GAGGGAAAGA--TGAAAAGAACTTTGAAAAGAGAGTTAAACAGTACGTGAAATTGTTGAAAGGGAAACGATTGAAGTCAGTCATGCCAGTAATGAATCAACCTCGAG----------------------------------------------------------TTTATTCTTTAGGTGTATTTCTTTACTTTCGGCAAGTTAGCATCGA-TTTTATCGGTCATAAAATGACT-AGGGTAAGGTGTCACC---TTC----GGGTGTGTTTATAGACCTTAGT-GGATGTGATCGTTGAGATCGAGGTTTGCAGCGAACAAT-CCTTTCGGGGGTTTAGTCGTCTGA-TCTCTGATTCGTTATCCTGTCTTCTCTTCAGC-TTGTCTGTGAGTTGCAGGTTCAACGTTCAAA-AG-GTTAGAACGTTCTTAAGTTTCGCTAAGGATGCTGACGTAATGGCTTTAAACGACCCGTCTTGAAACACGGACCAAGGAGTCTAAC-ATATGTGCGAGTGTT

>KX345941_Sacculospora_felinovii

CCTAGTAAGCGTGAGTCATCAGCTCACGTTGATTACGTCCCT-GCCCTTTGTACACACCGCCCGTCGCTACTACCGATTGAAT-GGCTTAGTGAGACTCTCGGATTGAG-TTTGAGAACCGGAAACGGCTCTCGTTCTTCGAAAAGTTAGTCAAACTTGGTCATTTAGAGGAAGTAAAAGTCGTAACAAGGTTTCTGTAGGTGAACCTGCAGAAGGATCATTAGAAAT---------------------------------------T--------------TTTGTATTC-AAAATCTTCCCAATCCTTTAATAAAAATT-----TTTTTTA----TATATA-ATT-------TTTTTAAAATTATTAATAACTTTCAACAACGGATCTCTTGGCTCTCGCATCGATGAAGAACGCAGCGAAATGCGATAAGTAATGTGAATTGCAG-ATTCCGTGAATCATCGAATCTTTGAACGCAAATTGCACTCTTT-GGTATTCCGAGGAGTATGCTTGCTTGAGTGTCCATTTA---TTATTATCGC-GATTA----------ATTTTTTTTATT-------------CGCGGATTTGGG---TTATCCGAA----------------------------------TTTTTTTGGAGACTTTAAAATTTAAT-------TCTTTTGGTA-TAATACGGTCA------AAACGT---GCTATTATTATTTA------------------ATAAAAGT-ATTT-CG-TTGATGATCTT-----ACCATAACTTTATATACTATG--TTTATCGCTTGTTA----------------------------TCT-TTTC--AAGAC-TC-GAG--T-GATTAAA-CATATATGTATAT--TTCGA--GTGTTGACCTCAAGTC-AAGTAAG-GTTACCCGCTGAAC-TT-AAGCATATCAATAAGCGGAGGAAAAGAAACTAAC-AAGGATTCCC-TTAG-TAAC-GGCGAGTGAAGTGGGAAAAGCTCAAA-TTTTAAATCTATCTAG-TCCTTCTAGGTCGAATTGTAATTTGAAGAAGCA-C-TTTCGGTTGCCCTGGTCTGATC-TAAATCCTTTGGGA--TGAGGTATCAT-GG-AGGGTGAGAATCCCGTTCATGATCTGGCATTG---GGT-TACCATATGATGTGTTTTCAAAGAGTCGAGTTG-TTTGGGATTGCAGCTCAAAATGGGTGGTATATCTCACCTAAGGCTAAATATCTGCGAGAGACCGATAGCGAACAAGTACCGT-GAGGGAAAGA--TGAAAAGAACTTTGAAAAGAGAGTTAAACAGTACGTGAAATTGTTGAAAGGGAAACGATTGAAGTCAGTCATGCCAGTAATGAATCAACCTCGAG----------------------------------------------------------TTTATTCTTTAGGTGTATTTCTTTACTTTCGGCAAGTTAGCATCGA-TTTTATCGGTCATAAAATGACT-AGGGTAAGGTGTCACC---TTC----GGGTGTGTTTATAGACCTTAGT-GGATGTGATCGTTGAGATCGAGGTTTGCAGCGAACAAT-CCTTTCGGGGGTTTAGTCGTCTGA-TCTCTGATTCGTTATCCTGTCTTCTCTTCAGC-TTGTCTGTGAGTTGCAGGTTCAACGTTCAAA-AG-GTTAGAACGTTCTTAAGTTTCGCTAAGGATGCTGACGTAATGGCTTTAAACGACCCGTCTTGAAACACGGACCAAGGAGTCTAAC-ATATGTGCGAGTGTT
