# Supplementary material for: Three new species of arbuscular mycorrhizal fungi (Glomeromycota) and Acaulospora gedanensis revised
Source: Front Microbiol. 2024 Feb 12;15:1320014. doi: 10.3389/fmicb.2024.1320014 (PMC10896085; doi:10.3389/fmicb.2024.1320014)
Supplement: Supplementary Table 6 — Data obtained from a BI analysis of 45S sequences (see Supplementary Figure S1). [file Table_6.DOCX]

#NEXUS

[ID: 9592689083]

begin taxa;

dimensions ntax=97;

taxlabels

448_3_

448_4_SSU_ITS_LSU_13_08_2021

Diversispora_densissima_MT724382

Diversispora_densissima_MT724383

Diversispora_densissima_MT724384

Diversispora_marina_MT725498

Diversispora_marina_MT725499

Diversispora_marina_MT725501

Diversispora_marina_MT725502

Diversispora_insculpta_KJ850195

Diversispora_insculpta_KJ850196

Diversispora_insculpta_KJ850197

Diversispora_varaderana_KT444708

Diversispora_varaderana_KT444709

Diversispora_varaderana_KT444710

Diversispora_varaderana_KT444711

Diversispora_aestuarii_OL684642

Diversispora_aestuarii_OL684645

Diversispora_aestuarii_OL684648

Diversispora_aestuarii_OL684644

Diversispora_clara_FR873629

Diversispora_clara_FR873632

Diversispora_clara_FR873630

Diversispora_clara_FR873631

Diversispora_peloponnesiaca_MN306206

Diversispora_peloponnesiaca_MN306207

Diversispora_peloponnesiaca_MN306208

Diversispora_peloponnesiaca_MN306205

Diversispora_celata_AM713402

Diversispora_celata_AM713403

Diversispora_celata_AY639225

Diversispora_eburnea_AM713407

Diversispora_eburnea_AM713408

Diversispora_eburnea_AM713411

Diversispora_eburnea_AM713406

Diversispora_slowinskiensis_KT444717

Diversispora_slowinskiensis_KT444719

Diversispora_slowinskiensis_KT444718

Diversispora_slowinskiensis_KT444720

Diversispora_epigaea_FM876814

Diversispora_epigaea_FM876817

Diversispora_epigaea_FM876819

Diversispora_epigaea_FM876818

Diversispora_sporocarpia_MK036785

Diversispora_sporocarpia_MK036786

Diversispora_sporocarpia_MK036788

Diversispora_sporocarpia_MK036789

Diversispora_arenaria_KJ850188

Diversispora_arenaria_KJ850189

Diversispora_arenaria_KJ850187

Diversispora_arenaria_KJ850186

Diversispora_jakucsiae_KJ850181

Diversispora_jakucsiae_KJ850182

Diversispora_jakucsiae_KJ850183

Diversispora_jakucsiae_KJ850184

Diversispora_aurantia_FN547661

Diversispora_aurantia_FN547664

Diversispora_aurantia_FN547655

Diversispora_aurantia_FN547657

Diversispora_alba_OP195880

Diversispora_alba_OP195882

Diversispora_alba_OP195886

Diversispora_alba_OP195889

Diversispora_spurca_MG459207

Diversispora_spurca_FN547637

Diversispora_spurca_FN547644

Diversispora_spurca_FN547639

Diversispora_sabulosa_MG459211

Diversispora_sabulosa_MG459212

Diversispora_sabulosa_MG459215

Diversispora_sabulosa_MG459214

Diversispora_sabulosa_MG459213

Diversispora_valentina_MT985516

Diversispora_valentina_MT985515

Diversispora_gibbosa_KJ850201

Diversispora_gibbosa_KJ850202

Diversispora_gibbosa_KJ850203

Diversispora_gibbosa_KJ850204

Diversispora_peridiata_KT444712

Diversispora_peridiata_KT444715

Diversispora_peridiata_KT444713

Diversispora_peridiata_KT444714

Diversispora_trimurales_KJ850199

Diversispora_trimurales_KJ850200

Diversispora_trimurales_KJ850198

Corymbiglomus_corymbiforme_KF060295

Corymbiglomus_corymbiforme_KF060298

Corymbiglomus_corymbiforme_KF060296

Redeckera_megalocarpum_HG518627

Redeckera_megalocarpum_HG518628

Redeckera_megalocarpum_HG518629

Siverdingia_tortuosa_JF439094

Siverdingia_tortuosa_JF439096

Siverdingia_tortuosa_JF439095

Desertispora_omaniana_KF154770

Desertispora_omaniana_MG459208

Desertispora_omaniana_KF154769

;

end;

begin trees;

translate

1 448_3_,

2 448_4_SSU_ITS_LSU_13_08_2021,

3 Diversispora_densissima_MT724382,

4 Diversispora_densissima_MT724383,

5 Diversispora_densissima_MT724384,

6 Diversispora_marina_MT725498,

7 Diversispora_marina_MT725499,

8 Diversispora_marina_MT725501,

9 Diversispora_marina_MT725502,

10 Diversispora_insculpta_KJ850195,

11 Diversispora_insculpta_KJ850196,

12 Diversispora_insculpta_KJ850197,

13 Diversispora_varaderana_KT444708,

14 Diversispora_varaderana_KT444709,

15 Diversispora_varaderana_KT444710,

16 Diversispora_varaderana_KT444711,

17 Diversispora_aestuarii_OL684642,

18 Diversispora_aestuarii_OL684645,

19 Diversispora_aestuarii_OL684648,

20 Diversispora_aestuarii_OL684644,

21 Diversispora_clara_FR873629,

22 Diversispora_clara_FR873632,

23 Diversispora_clara_FR873630,

24 Diversispora_clara_FR873631,

25 Diversispora_peloponnesiaca_MN306206,

26 Diversispora_peloponnesiaca_MN306207,

27 Diversispora_peloponnesiaca_MN306208,

28 Diversispora_peloponnesiaca_MN306205,

29 Diversispora_celata_AM713402,

30 Diversispora_celata_AM713403,

31 Diversispora_celata_AY639225,

32 Diversispora_eburnea_AM713407,

33 Diversispora_eburnea_AM713408,

34 Diversispora_eburnea_AM713411,

35 Diversispora_eburnea_AM713406,

36 Diversispora_slowinskiensis_KT444717,

37 Diversispora_slowinskiensis_KT444719,

38 Diversispora_slowinskiensis_KT444718,

39 Diversispora_slowinskiensis_KT444720,

40 Diversispora_epigaea_FM876814,

41 Diversispora_epigaea_FM876817,

42 Diversispora_epigaea_FM876819,

43 Diversispora_epigaea_FM876818,

44 Diversispora_sporocarpia_MK036785,

45 Diversispora_sporocarpia_MK036786,

46 Diversispora_sporocarpia_MK036788,

47 Diversispora_sporocarpia_MK036789,

48 Diversispora_arenaria_KJ850188,

49 Diversispora_arenaria_KJ850189,

50 Diversispora_arenaria_KJ850187,

51 Diversispora_arenaria_KJ850186,

52 Diversispora_jakucsiae_KJ850181,

53 Diversispora_jakucsiae_KJ850182,

54 Diversispora_jakucsiae_KJ850183,

55 Diversispora_jakucsiae_KJ850184,

56 Diversispora_aurantia_FN547661,

57 Diversispora_aurantia_FN547664,

58 Diversispora_aurantia_FN547655,

59 Diversispora_aurantia_FN547657,

60 Diversispora_alba_OP195880,

61 Diversispora_alba_OP195882,

62 Diversispora_alba_OP195886,

63 Diversispora_alba_OP195889,

64 Diversispora_spurca_MG459207,

65 Diversispora_spurca_FN547637,

66 Diversispora_spurca_FN547644,

67 Diversispora_spurca_FN547639,

68 Diversispora_sabulosa_MG459211,

69 Diversispora_sabulosa_MG459212,

70 Diversispora_sabulosa_MG459215,

71 Diversispora_sabulosa_MG459214,

72 Diversispora_sabulosa_MG459213,

73 Diversispora_valentina_MT985516,

74 Diversispora_valentina_MT985515,

75 Diversispora_gibbosa_KJ850201,

76 Diversispora_gibbosa_KJ850202,

77 Diversispora_gibbosa_KJ850203,

78 Diversispora_gibbosa_KJ850204,

79 Diversispora_peridiata_KT444712,

80 Diversispora_peridiata_KT444715,

81 Diversispora_peridiata_KT444713,

82 Diversispora_peridiata_KT444714,

83 Diversispora_trimurales_KJ850199,

84 Diversispora_trimurales_KJ850200,

85 Diversispora_trimurales_KJ850198,

86 Corymbiglomus_corymbiforme_KF060295,

87 Corymbiglomus_corymbiforme_KF060298,

88 Corymbiglomus_corymbiforme_KF060296,

89 Redeckera_megalocarpum_HG518627,

90 Redeckera_megalocarpum_HG518628,

91 Redeckera_megalocarpum_HG518629,

92 Siverdingia_tortuosa_JF439094,

93 Siverdingia_tortuosa_JF439096,

94 Siverdingia_tortuosa_JF439095,

95 Desertispora_omaniana_KF154770,

96 Desertispora_omaniana_MG459208,

97 Desertispora_omaniana_KF154769

;

tree con_50_majrule = [&U] (1[&prob=1.00000000e+00,prob_stddev=0.00000000e+00,prob_range={1.00000000e+00,1.00000000e+00},prob(percent)="100",prob+-sd="100+-0"]:7.292555e-04[&length_mean=1.01980664e-03,length_median=7.29255500e-04,length_95%HPD={1.67693300e-06,2.88914400e-03}],2[&prob=1.00000000e+00,prob_stddev=0.00000000e+00,prob_range={1.00000000e+00,1.00000000e+00},prob(percent)="100",prob+-sd="100+-0"]:9.480766e-03[&length_mean=9.59072012e-03,length_median=9.48076600e-03,length_95%HPD={4.62586700e-03,1.51350100e-02}],(((3[&prob=1.00000000e+00,prob_stddev=0.00000000e+00,prob_range={1.00000000e+00,1.00000000e+00},prob(percent)="100",prob+-sd="100+-0"]:4.985139e-04[&length_mean=7.48598017e-04,length_median=4.98513900e-04,length_95%HPD={1.56480400e-06,2.25466000e-03}],4[&prob=1.00000000e+00,prob_stddev=0.00000000e+00,prob_range={1.00000000e+00,1.00000000e+00},prob(percent)="100",prob+-sd="100+-0"]:5.288671e-04[&length_mean=7.33729111e-04,length_median=5.28867100e-04,length_95%HPD={4.51751000e-08,2.17500700e-03}])[&prob=1.00000000e+00,prob_stddev=0.00000000e+00,prob_range={1.00000000e+00,1.00000000e+00},prob(percent)="100",prob+-sd="100+-0"]:6.197901e-03[&length_mean=6.39017558e-03,length_median=6.19790100e-03,length_95%HPD={2.29392600e-03,1.09150100e-02}],5[&prob=1.00000000e+00,prob_stddev=0.00000000e+00,prob_range={1.00000000e+00,1.00000000e+00},prob(percent)="100",prob+-sd="100+-0"]:6.535193e-03[&length_mean=6.83061139e-03,length_median=6.53519300e-03,length_95%HPD={2.72253800e-03,1.17387500e-02}])[&prob=1.00000000e+00,prob_stddev=0.00000000e+00,prob_range={1.00000000e+00,1.00000000e+00},prob(percent)="100",prob+-sd="100+-0"]:1.302065e-02[&length_mean=1.34219505e-02,length_median=1.30206500e-02,length_95%HPD={6.48163400e-03,1.96432000e-02}],(((6[&prob=1.00000000e+00,prob_stddev=0.00000000e+00,prob_range={1.00000000e+00,1.00000000e+00},prob(percent)="100",prob+-sd="100+-0"]:5.393721e-04[&length_mean=7.77242439e-04,length_median=5.39372100e-04,length_95%HPD={1.15326300e-06,2.34336700e-03}],7[&prob=1.00000000e+00,prob_stddev=0.00000000e+00,prob_range={1.00000000e+00,1.00000000e+00},prob(percent)="100",prob+-sd="100+-0"]:5.090510e-04[&length_mean=7.95445019e-04,length_median=5.09051000e-04,length_95%HPD={5.84962000e-07,2.41069100e-03}])[&prob=9.78695073e-01,prob_stddev=1.88310727e-03,prob_range={9.77363515e-01,9.80026631e-01},prob(percent)="98",prob+-sd="98+-0"]:1.316147e-03[&length_mean=1.59096118e-03,length_median=1.31614700e-03,length_95%HPD={2.30703600e-05,3.71416700e-03}],(8[&prob=1.00000000e+00,prob_stddev=0.00000000e+00,prob_range={1.00000000e+00,1.00000000e+00},prob(percent)="100",prob+-sd="100+-0"]:5.606703e-04[&length_mean=7.63534449e-04,length_median=5.60670300e-04,length_95%HPD={3.09861800e-07,2.34256300e-03}],9[&prob=1.00000000e+00,prob_stddev=0.00000000e+00,prob_range={1.00000000e+00,1.00000000e+00},prob(percent)="100",prob+-sd="100+-0"]:5.579421e-04[&length_mean=7.66163246e-04,length_median=5.57942100e-04,length_95%HPD={8.42986100e-07,2.24437500e-03}])[&prob=9.52729694e-01,prob_stddev=9.41553637e-04,prob_range={9.52063915e-01,9.53395473e-01},prob(percent)="95",prob+-sd="95+-0"]:1.232370e-03[&length_mean=1.50350040e-03,length_median=1.23237000e-03,length_95%HPD={3.00158200e-05,3.58882800e-03}])[&prob=1.00000000e+00,prob_stddev=0.00000000e+00,prob_range={1.00000000e+00,1.00000000e+00},prob(percent)="100",prob+-sd="100+-0"]:1.741739e-02[&length_mean=1.78310301e-02,length_median=1.74173900e-02,length_95%HPD={1.12267200e-02,2.66589900e-02}],(((10[&prob=1.00000000e+00,prob_stddev=0.00000000e+00,prob_range={1.00000000e+00,1.00000000e+00},prob(percent)="100",prob+-sd="100+-0"]:5.648599e-04[&length_mean=7.90470184e-04,length_median=5.64859900e-04,length_95%HPD={1.50173400e-06,2.31811800e-03}],11[&prob=1.00000000e+00,prob_stddev=0.00000000e+00,prob_range={1.00000000e+00,1.00000000e+00},prob(percent)="100",prob+-sd="100+-0"]:5.261295e-04[&length_mean=7.57304695e-04,length_median=5.26129500e-04,length_95%HPD={1.41245300e-06,2.20843700e-03}],12[&prob=1.00000000e+00,prob_stddev=0.00000000e+00,prob_range={1.00000000e+00,1.00000000e+00},prob(percent)="100",prob+-sd="100+-0"]:5.086613e-04[&length_mean=7.74585220e-04,length_median=5.08661300e-04,length_95%HPD={1.16623900e-06,2.38143900e-03}])[&prob=1.00000000e+00,prob_stddev=0.00000000e+00,prob_range={1.00000000e+00,1.00000000e+00},prob(percent)="100",prob+-sd="100+-0"]:1.483009e-02[&length_mean=1.51641365e-02,length_median=1.48300900e-02,length_95%HPD={8.45595000e-03,2.29008500e-02}],((13[&prob=1.00000000e+00,prob_stddev=0.00000000e+00,prob_range={1.00000000e+00,1.00000000e+00},prob(percent)="100",prob+-sd="100+-0"]:6.880350e-03[&length_mean=7.18164441e-03,length_median=6.88035000e-03,length_95%HPD={2.48875700e-03,1.18394400e-02}],((14[&prob=1.00000000e+00,prob_stddev=0.00000000e+00,prob_range={1.00000000e+00,1.00000000e+00},prob(percent)="100",prob+-sd="100+-0"]:5.517803e-03[&length_mean=5.79044239e-03,length_median=5.51780300e-03,length_95%HPD={1.95398000e-03,1.02361100e-02}],16[&prob=1.00000000e+00,prob_stddev=0.00000000e+00,prob_range={1.00000000e+00,1.00000000e+00},prob(percent)="100",prob+-sd="100+-0"]:6.603326e-03[&length_mean=6.70625054e-03,length_median=6.60332600e-03,length_95%HPD={2.76909000e-03,1.10846900e-02}])[&prob=9.78695073e-01,prob_stddev=9.41553637e-03,prob_range={9.72037284e-01,9.85352863e-01},prob(percent)="98",prob+-sd="98+-1"]:2.316001e-03[&length_mean=2.56905359e-03,length_median=2.31600100e-03,length_95%HPD={1.90859900e-04,5.37547900e-03}],15[&prob=1.00000000e+00,prob_stddev=0.00000000e+00,prob_range={1.00000000e+00,1.00000000e+00},prob(percent)="100",prob+-sd="100+-0"]:1.370306e-03[&length_mean=1.60375770e-03,length_median=1.37030600e-03,length_95%HPD={8.19393600e-05,3.76167000e-03}])[&prob=9.70039947e-01,prob_stddev=1.03570900e-02,prob_range={9.62716378e-01,9.77363515e-01},prob(percent)="97",prob+-sd="97+-1"]:2.074043e-03[&length_mean=2.36117423e-03,length_median=2.07404300e-03,length_95%HPD={8.74257600e-05,5.12309400e-03}])[&prob=9.98002663e-01,prob_stddev=2.82466091e-03,prob_range={9.96005326e-01,1.00000000e+00},prob(percent)="100",prob+-sd="100+-0"]:3.815643e-03[&length_mean=4.19053502e-03,length_median=3.81564300e-03,length_95%HPD={4.65445800e-04,8.46400100e-03}],((17[&prob=1.00000000e+00,prob_stddev=0.00000000e+00,prob_range={1.00000000e+00,1.00000000e+00},prob(percent)="100",prob+-sd="100+-0"]:1.208213e-02[&length_mean=1.22397736e-02,length_median=1.20821300e-02,length_95%HPD={6.03084500e-03,1.89861100e-02}],(19[&prob=1.00000000e+00,prob_stddev=0.00000000e+00,prob_range={1.00000000e+00,1.00000000e+00},prob(percent)="100",prob+-sd="100+-0"]:1.134768e-02[&length_mean=1.16302244e-02,length_median=1.13476800e-02,length_95%HPD={5.10800800e-03,1.80658900e-02}],20[&prob=1.00000000e+00,prob_stddev=0.00000000e+00,prob_range={1.00000000e+00,1.00000000e+00},prob(percent)="100",prob+-sd="100+-0"]:2.519338e-02[&length_mean=2.53336699e-02,length_median=2.51933800e-02,length_95%HPD={1.62986100e-02,3.56061100e-02}])[&prob=9.96005326e-01,prob_stddev=5.64932182e-03,prob_range={9.92010652e-01,1.00000000e+00},prob(percent)="100",prob+-sd="100+-1"]:9.577512e-03[&length_mean=9.92261000e-03,length_median=9.57751200e-03,length_95%HPD={4.82275300e-03,1.64649800e-02}])[&prob=9.80026631e-01,prob_stddev=0.00000000e+00,prob_range={9.80026631e-01,9.80026631e-01},prob(percent)="98",prob+-sd="98+-0"]:4.139024e-03[&length_mean=4.46661536e-03,length_median=4.13902400e-03,length_95%HPD={5.58988800e-04,9.15290900e-03}],18[&prob=1.00000000e+00,prob_stddev=0.00000000e+00,prob_range={1.00000000e+00,1.00000000e+00},prob(percent)="100",prob+-sd="100+-0"]:1.541370e-02[&length_mean=1.56236019e-02,length_median=1.54137000e-02,length_95%HPD={8.91529700e-03,2.32859200e-02}])[&prob=9.95339547e-01,prob_stddev=2.82466091e-03,prob_range={9.93342210e-01,9.97336884e-01},prob(percent)="100",prob+-sd="100+-0"]:5.542602e-03[&length_mean=5.94061974e-03,length_median=5.54260200e-03,length_95%HPD={1.00220200e-03,1.10118700e-02}])[&prob=9.58055925e-01,prob_stddev=2.35388409e-02,prob_range={9.41411451e-01,9.74700399e-01},prob(percent)="96",prob+-sd="96+-2"]:6.335949e-03[&length_mean=6.60691410e-03,length_median=6.33594900e-03,length_95%HPD={2.09511100e-03,1.15276900e-02}])[&prob=1.00000000e+00,prob_stddev=0.00000000e+00,prob_range={1.00000000e+00,1.00000000e+00},prob(percent)="100",prob+-sd="100+-0"]:1.515391e-02[&length_mean=1.55466921e-02,length_median=1.51539100e-02,length_95%HPD={8.39570100e-03,2.36055500e-02}],((((((21[&prob=1.00000000e+00,prob_stddev=0.00000000e+00,prob_range={1.00000000e+00,1.00000000e+00},prob(percent)="100",prob+-sd="100+-0"]:7.488314e-03[&length_mean=7.58548585e-03,length_median=7.48831400e-03,length_95%HPD={2.63054000e-03,1.30520700e-02}],23[&prob=1.00000000e+00,prob_stddev=0.00000000e+00,prob_range={1.00000000e+00,1.00000000e+00},prob(percent)="100",prob+-sd="100+-0"]:1.870297e-03[&length_mean=2.16362162e-03,length_median=1.87029700e-03,length_95%HPD={2.15273600e-04,4.88643400e-03}],24[&prob=1.00000000e+00,prob_stddev=0.00000000e+00,prob_range={1.00000000e+00,1.00000000e+00},prob(percent)="100",prob+-sd="100+-0"]:3.633714e-03[&length_mean=3.88701598e-03,length_median=3.63371400e-03,length_95%HPD={1.01147700e-03,7.20631300e-03}])[&prob=5.33954727e-01,prob_stddev=1.12986436e-02,prob_range={5.25965379e-01,5.41944075e-01},prob(percent)="53",prob+-sd="53+-1"]:3.447028e-03[&length_mean=3.64056330e-03,length_median=3.44702800e-03,length_95%HPD={9.54575400e-04,7.58525500e-03}],22[&prob=1.00000000e+00,prob_stddev=0.00000000e+00,prob_range={1.00000000e+00,1.00000000e+00},prob(percent)="100",prob+-sd="100+-0"]:6.366528e-03[&length_mean=6.73755849e-03,length_median=6.36652800e-03,length_95%HPD={2.03706300e-03,1.19631900e-02}])[&prob=9.99334221e-01,prob_stddev=9.41553637e-04,prob_range={9.98668442e-01,1.00000000e+00},prob(percent)="100",prob+-sd="100+-0"]:4.736718e-03[&length_mean=5.11997096e-03,length_median=4.73671800e-03,length_95%HPD={1.52956300e-03,9.89210900e-03}],(25[&prob=1.00000000e+00,prob_stddev=0.00000000e+00,prob_range={1.00000000e+00,1.00000000e+00},prob(percent)="100",prob+-sd="100+-0"]:7.302811e-03[&length_mean=7.67879236e-03,length_median=7.30281100e-03,length_95%HPD={3.07151600e-03,1.26194000e-02}],((26[&prob=1.00000000e+00,prob_stddev=0.00000000e+00,prob_range={1.00000000e+00,1.00000000e+00},prob(percent)="100",prob+-sd="100+-0"]:2.254502e-03[&length_mean=2.58151714e-03,length_median=2.25450200e-03,length_95%HPD={1.89671700e-04,5.48017800e-03}],27[&prob=1.00000000e+00,prob_stddev=0.00000000e+00,prob_range={1.00000000e+00,1.00000000e+00},prob(percent)="100",prob+-sd="100+-0"]:9.153472e-03[&length_mean=9.46419135e-03,length_median=9.15347200e-03,length_95%HPD={4.12278900e-03,1.46850700e-02}])[&prob=1.00000000e+00,prob_stddev=0.00000000e+00,prob_range={1.00000000e+00,1.00000000e+00},prob(percent)="100",prob+-sd="100+-0"]:6.218280e-03[&length_mean=6.50460622e-03,length_median=6.21828000e-03,length_95%HPD={2.00712600e-03,1.10906400e-02}],28[&prob=1.00000000e+00,prob_stddev=0.00000000e+00,prob_range={1.00000000e+00,1.00000000e+00},prob(percent)="100",prob+-sd="100+-0"]:1.656390e-02[&length_mean=1.70585529e-02,length_median=1.65639000e-02,length_95%HPD={9.27765500e-03,2.45358300e-02}])[&prob=7.22370173e-01,prob_stddev=1.03570900e-02,prob_range={7.15046605e-01,7.29693742e-01},prob(percent)="72",prob+-sd="72+-1"]:2.785256e-03[&length_mean=3.16057749e-03,length_median=2.78525600e-03,length_95%HPD={2.71825700e-04,7.16683400e-03}])[&prob=8.09587217e-01,prob_stddev=1.50648582e-02,prob_range={7.98934754e-01,8.20239680e-01},prob(percent)="81",prob+-sd="81+-2"]:3.584000e-03[&length_mean=3.83163698e-03,length_median=3.58400000e-03,length_95%HPD={6.48091600e-04,7.43864000e-03}])[&prob=1.00000000e+00,prob_stddev=0.00000000e+00,prob_range={1.00000000e+00,1.00000000e+00},prob(percent)="100",prob+-sd="100+-0"]:2.315264e-02[&length_mean=2.33685584e-02,length_median=2.31526400e-02,length_95%HPD={1.49296100e-02,3.21663500e-02}],(((29[&prob=1.00000000e+00,prob_stddev=0.00000000e+00,prob_range={1.00000000e+00,1.00000000e+00},prob(percent)="100",prob+-sd="100+-0"]:3.787018e-03[&length_mean=3.94998070e-03,length_median=3.78701800e-03,length_95%HPD={8.38871900e-04,7.42286800e-03}],(30[&prob=1.00000000e+00,prob_stddev=0.00000000e+00,prob_range={1.00000000e+00,1.00000000e+00},prob(percent)="100",prob+-sd="100+-0"]:2.078486e-03[&length_mean=2.62387441e-03,length_median=2.07848600e-03,length_95%HPD={1.41505000e-06,6.91692800e-03}],31[&prob=1.00000000e+00,prob_stddev=0.00000000e+00,prob_range={1.00000000e+00,1.00000000e+00},prob(percent)="100",prob+-sd="100+-0"]:2.494641e-03[&length_mean=3.11127411e-03,length_median=2.49464100e-03,length_95%HPD={7.10648200e-05,7.51242400e-03}])[&prob=9.78029294e-01,prob_stddev=9.41553637e-04,prob_range={9.77363515e-01,9.78695073e-01},prob(percent)="98",prob+-sd="98+-0"]:4.460123e-03[&length_mean=4.93218782e-03,length_median=4.46012300e-03,length_95%HPD={4.01697200e-04,9.92821700e-03}])[&prob=1.00000000e+00,prob_stddev=0.00000000e+00,prob_range={1.00000000e+00,1.00000000e+00},prob(percent)="100",prob+-sd="100+-0"]:2.241244e-02[&length_mean=2.28299281e-02,length_median=2.24124400e-02,length_95%HPD={1.36815400e-02,3.28000400e-02}],(((32[&prob=1.00000000e+00,prob_stddev=0.00000000e+00,prob_range={1.00000000e+00,1.00000000e+00},prob(percent)="100",prob+-sd="100+-0"]:5.745683e-04[&length_mean=8.25510011e-04,length_median=5.74568300e-04,length_95%HPD={1.06071700e-06,2.57508500e-03}],33[&prob=1.00000000e+00,prob_stddev=0.00000000e+00,prob_range={1.00000000e+00,1.00000000e+00},prob(percent)="100",prob+-sd="100+-0"]:5.767499e-04[&length_mean=8.19817149e-04,length_median=5.76749900e-04,length_95%HPD={5.72370100e-07,2.48129100e-03}])[&prob=1.00000000e+00,prob_stddev=0.00000000e+00,prob_range={1.00000000e+00,1.00000000e+00},prob(percent)="100",prob+-sd="100+-0"]:2.077204e-03[&length_mean=2.34579442e-03,length_median=2.07720400e-03,length_95%HPD={1.54489500e-04,4.93165600e-03}],34[&prob=1.00000000e+00,prob_stddev=0.00000000e+00,prob_range={1.00000000e+00,1.00000000e+00},prob(percent)="100",prob+-sd="100+-0"]:5.325458e-04[&length_mean=7.56170596e-04,length_median=5.32545800e-04,length_95%HPD={6.68160400e-07,2.29343800e-03}])[&prob=9.74034621e-01,prob_stddev=1.03570900e-02,prob_range={9.66711052e-01,9.81358189e-01},prob(percent)="97",prob+-sd="97+-1"]:2.132763e-03[&length_mean=2.39216835e-03,length_median=2.13276300e-03,length_95%HPD={1.33767800e-04,5.26246800e-03}],35[&prob=1.00000000e+00,prob_stddev=0.00000000e+00,prob_range={1.00000000e+00,1.00000000e+00},prob(percent)="100",prob+-sd="100+-0"]:8.353218e-03[&length_mean=8.49660400e-03,length_median=8.35321800e-03,length_95%HPD={3.74889200e-03,1.35298300e-02}])[&prob=1.00000000e+00,prob_stddev=0.00000000e+00,prob_range={1.00000000e+00,1.00000000e+00},prob(percent)="100",prob+-sd="100+-0"]:1.074064e-02[&length_mean=1.09515959e-02,length_median=1.07406400e-02,length_95%HPD={4.36304400e-03,1.73748500e-02}])[&prob=1.00000000e+00,prob_stddev=0.00000000e+00,prob_range={1.00000000e+00,1.00000000e+00},prob(percent)="100",prob+-sd="100+-0"]:1.375799e-02[&length_mean=1.40327089e-02,length_median=1.37579900e-02,length_95%HPD={5.89182600e-03,2.15373500e-02}],(((((56[&prob=1.00000000e+00,prob_stddev=0.00000000e+00,prob_range={1.00000000e+00,1.00000000e+00},prob(percent)="100",prob+-sd="100+-0"]:5.219284e-04[&length_mean=7.28069799e-04,length_median=5.21928400e-04,length_95%HPD={9.64265500e-07,1.95191300e-03}],57[&prob=1.00000000e+00,prob_stddev=0.00000000e+00,prob_range={1.00000000e+00,1.00000000e+00},prob(percent)="100",prob+-sd="100+-0"]:5.684465e-04[&length_mean=8.01893275e-04,length_median=5.68446500e-04,length_95%HPD={1.55541800e-06,2.36854100e-03}])[&prob=1.00000000e+00,prob_stddev=0.00000000e+00,prob_range={1.00000000e+00,1.00000000e+00},prob(percent)="100",prob+-sd="100+-0"]:5.836810e-03[&length_mean=6.06569430e-03,length_median=5.83681000e-03,length_95%HPD={1.84206100e-03,1.00958700e-02}],(58[&prob=1.00000000e+00,prob_stddev=0.00000000e+00,prob_range={1.00000000e+00,1.00000000e+00},prob(percent)="100",prob+-sd="100+-0"]:2.046807e-03[&length_mean=2.35325227e-03,length_median=2.04680700e-03,length_95%HPD={2.52384500e-04,4.99604100e-03}],59[&prob=1.00000000e+00,prob_stddev=0.00000000e+00,prob_range={1.00000000e+00,1.00000000e+00},prob(percent)="100",prob+-sd="100+-0"]:2.736094e-03[&length_mean=3.04004096e-03,length_median=2.73609400e-03,length_95%HPD={3.44271600e-04,6.02197200e-03}])[&prob=1.00000000e+00,prob_stddev=0.00000000e+00,prob_range={1.00000000e+00,1.00000000e+00},prob(percent)="100",prob+-sd="100+-0"]:4.613802e-03[&length_mean=4.94138760e-03,length_median=4.61380200e-03,length_95%HPD={1.29230100e-03,9.15255900e-03}])[&prob=1.00000000e+00,prob_stddev=0.00000000e+00,prob_range={1.00000000e+00,1.00000000e+00},prob(percent)="100",prob+-sd="100+-0"]:1.596800e-02[&length_mean=1.63059615e-02,length_median=1.59680000e-02,length_95%HPD={9.65027800e-03,2.42816000e-02}],(64[&prob=1.00000000e+00,prob_stddev=0.00000000e+00,prob_range={1.00000000e+00,1.00000000e+00},prob(percent)="100",prob+-sd="100+-0"]:2.497925e-02[&length_mean=2.52445195e-02,length_median=2.49792500e-02,length_95%HPD={1.68672100e-02,3.56309100e-02}],((65[&prob=1.00000000e+00,prob_stddev=0.00000000e+00,prob_range={1.00000000e+00,1.00000000e+00},prob(percent)="100",prob+-sd="100+-0"]:8.827778e-03[&length_mean=9.00485755e-03,length_median=8.82777800e-03,length_95%HPD={4.17218100e-03,1.44172000e-02}],66[&prob=1.00000000e+00,prob_stddev=0.00000000e+00,prob_range={1.00000000e+00,1.00000000e+00},prob(percent)="100",prob+-sd="100+-0"]:2.911143e-03[&length_mean=3.21760646e-03,length_median=2.91114300e-03,length_95%HPD={4.98087300e-04,6.81491400e-03}])[&prob=8.84154461e-01,prob_stddev=2.25972873e-02,prob_range={8.68175766e-01,9.00133156e-01},prob(percent)="88",prob+-sd="88+-2"]:3.481508e-03[&length_mean=3.75678313e-03,length_median=3.48150800e-03,length_95%HPD={5.37984600e-04,7.46243100e-03}],67[&prob=1.00000000e+00,prob_stddev=0.00000000e+00,prob_range={1.00000000e+00,1.00000000e+00},prob(percent)="100",prob+-sd="100+-0"]:8.303826e-03[&length_mean=8.65179791e-03,length_median=8.30382600e-03,length_95%HPD={3.79192100e-03,1.43687300e-02}])[&prob=1.00000000e+00,prob_stddev=0.00000000e+00,prob_range={1.00000000e+00,1.00000000e+00},prob(percent)="100",prob+-sd="100+-0"]:1.736990e-02[&length_mean=1.76297786e-02,length_median=1.73699000e-02,length_95%HPD={9.97077500e-03,2.61025400e-02}])[&prob=9.90679095e-01,prob_stddev=3.76621455e-03,prob_range={9.88015979e-01,9.93342210e-01},prob(percent)="99",prob+-sd="99+-0"]:5.867564e-03[&length_mean=6.18180039e-03,length_median=5.86756400e-03,length_95%HPD={1.58953300e-03,1.13765400e-02}])[&prob=6.79094541e-01,prob_stddev=1.88310727e-03,prob_range={6.77762983e-01,6.80426099e-01},prob(percent)="68",prob+-sd="68+-0"]:4.961165e-03[&length_mean=5.30742401e-03,length_median=4.96116500e-03,length_95%HPD={8.60674000e-05,1.08056400e-02}],((60[&prob=1.00000000e+00,prob_stddev=0.00000000e+00,prob_range={1.00000000e+00,1.00000000e+00},prob(percent)="100",prob+-sd="100+-0"]:7.200097e-03[&length_mean=7.49765280e-03,length_median=7.20009700e-03,length_95%HPD={2.92624800e-03,1.22619400e-02}],(62[&prob=1.00000000e+00,prob_stddev=0.00000000e+00,prob_range={1.00000000e+00,1.00000000e+00},prob(percent)="100",prob+-sd="100+-0"]:3.537109e-03[&length_mean=3.76978754e-03,length_median=3.53710900e-03,length_95%HPD={1.93137900e-04,7.40248300e-03}],63[&prob=1.00000000e+00,prob_stddev=0.00000000e+00,prob_range={1.00000000e+00,1.00000000e+00},prob(percent)="100",prob+-sd="100+-0"]:8.587641e-03[&length_mean=8.80277977e-03,length_median=8.58764100e-03,length_95%HPD={4.11084100e-03,1.42295000e-02}])[&prob=1.00000000e+00,prob_stddev=0.00000000e+00,prob_range={1.00000000e+00,1.00000000e+00},prob(percent)="100",prob+-sd="100+-0"]:8.777721e-03[&length_mean=9.08425775e-03,length_median=8.77772100e-03,length_95%HPD={3.30403500e-03,1.45090500e-02}])[&prob=8.24234354e-01,prob_stddev=1.69479655e-02,prob_range={8.12250333e-01,8.36218375e-01},prob(percent)="82",prob+-sd="82+-2"]:2.944689e-03[&length_mean=3.20341107e-03,length_median=2.94468900e-03,length_95%HPD={2.13602500e-04,6.56921000e-03}],61[&prob=1.00000000e+00,prob_stddev=0.00000000e+00,prob_range={1.00000000e+00,1.00000000e+00},prob(percent)="100",prob+-sd="100+-0"]:4.603430e-03[&length_mean=4.97172967e-03,length_median=4.60343000e-03,length_95%HPD={1.42923900e-03,9.50735300e-03}])[&prob=1.00000000e+00,prob_stddev=0.00000000e+00,prob_range={1.00000000e+00,1.00000000e+00},prob(percent)="100",prob+-sd="100+-0"]:1.550031e-02[&length_mean=1.57562419e-02,length_median=1.55003100e-02,length_95%HPD={7.42065300e-03,2.44313900e-02}])[&prob=1.00000000e+00,prob_stddev=0.00000000e+00,prob_range={1.00000000e+00,1.00000000e+00},prob(percent)="100",prob+-sd="100+-0"]:1.654813e-02[&length_mean=1.70270393e-02,length_median=1.65481300e-02,length_95%HPD={8.91152600e-03,2.59464400e-02}],((68[&prob=1.00000000e+00,prob_stddev=0.00000000e+00,prob_range={1.00000000e+00,1.00000000e+00},prob(percent)="100",prob+-sd="100+-0"]:5.635469e-04[&length_mean=7.92176514e-04,length_median=5.63546900e-04,length_95%HPD={4.87062200e-07,2.36227400e-03}],69[&prob=1.00000000e+00,prob_stddev=0.00000000e+00,prob_range={1.00000000e+00,1.00000000e+00},prob(percent)="100",prob+-sd="100+-0"]:5.102925e-04[&length_mean=7.57618553e-04,length_median=5.10292500e-04,length_95%HPD={5.02859200e-07,2.35780800e-03}])[&prob=1.00000000e+00,prob_stddev=0.00000000e+00,prob_range={1.00000000e+00,1.00000000e+00},prob(percent)="100",prob+-sd="100+-0"]:5.996042e-03[&length_mean=6.33648684e-03,length_median=5.99604200e-03,length_95%HPD={1.98232500e-03,1.13257900e-02}],((70[&prob=1.00000000e+00,prob_stddev=0.00000000e+00,prob_range={1.00000000e+00,1.00000000e+00},prob(percent)="100",prob+-sd="100+-0"]:4.717854e-04[&length_mean=7.30400920e-04,length_median=4.71785400e-04,length_95%HPD={5.02908400e-07,2.23688000e-03}],71[&prob=1.00000000e+00,prob_stddev=0.00000000e+00,prob_range={1.00000000e+00,1.00000000e+00},prob(percent)="100",prob+-sd="100+-0"]:5.056656e-04[&length_mean=7.46621215e-04,length_median=5.05665600e-04,length_95%HPD={7.64378600e-07,2.25293200e-03}])[&prob=9.90679095e-01,prob_stddev=3.76621455e-03,prob_range={9.88015979e-01,9.93342210e-01},prob(percent)="99",prob+-sd="99+-0"]:1.300321e-03[&length_mean=1.56938022e-03,length_median=1.30032100e-03,length_95%HPD={2.55336200e-05,3.63383600e-03}],72[&prob=1.00000000e+00,prob_stddev=0.00000000e+00,prob_range={1.00000000e+00,1.00000000e+00},prob(percent)="100",prob+-sd="100+-0"]:4.842083e-04[&length_mean=7.40776357e-04,length_median=4.84208300e-04,length_95%HPD={4.38240900e-07,2.21307700e-03}])[&prob=1.00000000e+00,prob_stddev=0.00000000e+00,prob_range={1.00000000e+00,1.00000000e+00},prob(percent)="100",prob+-sd="100+-0"]:7.290086e-03[&length_mean=7.62145268e-03,length_median=7.29008600e-03,length_95%HPD={3.01046300e-03,1.29499900e-02}])[&prob=1.00000000e+00,prob_stddev=0.00000000e+00,prob_range={1.00000000e+00,1.00000000e+00},prob(percent)="100",prob+-sd="100+-0"]:4.008722e-02[&length_mean=4.05492742e-02,length_median=4.00872200e-02,length_95%HPD={2.80364000e-02,5.38465600e-02}])[&prob=1.00000000e+00,prob_stddev=0.00000000e+00,prob_range={1.00000000e+00,1.00000000e+00},prob(percent)="100",prob+-sd="100+-0"]:1.307777e-02[&length_mean=1.35087108e-02,length_median=1.30777700e-02,length_95%HPD={6.33005700e-03,2.10989600e-02}])[&prob=9.59387483e-01,prob_stddev=1.03570900e-02,prob_range={9.52063915e-01,9.66711052e-01},prob(percent)="96",prob+-sd="96+-1"]:5.765643e-03[&length_mean=6.14843903e-03,length_median=5.76564300e-03,length_95%HPD={9.44351300e-04,1.26296900e-02}])[&prob=8.32889481e-01,prob_stddev=8.47398273e-03,prob_range={8.26897470e-01,8.38881491e-01},prob(percent)="83",prob+-sd="83+-1"]:5.142516e-03[&length_mean=5.53103743e-03,length_median=5.14251600e-03,length_95%HPD={9.12397900e-04,1.04393800e-02}],((((((36[&prob=1.00000000e+00,prob_stddev=0.00000000e+00,prob_range={1.00000000e+00,1.00000000e+00},prob(percent)="100",prob+-sd="100+-0"]:4.419242e-03[&length_mean=4.62480810e-03,length_median=4.41924200e-03,length_95%HPD={1.29545000e-03,8.61191300e-03}],37[&prob=1.00000000e+00,prob_stddev=0.00000000e+00,prob_range={1.00000000e+00,1.00000000e+00},prob(percent)="100",prob+-sd="100+-0"]:1.376599e-03[&length_mean=1.63313741e-03,length_median=1.37659900e-03,length_95%HPD={4.08428100e-05,4.03144300e-03}])[&prob=9.96005326e-01,prob_stddev=1.88310727e-03,prob_range={9.94673768e-01,9.97336884e-01},prob(percent)="100",prob+-sd="100+-0"]:3.396540e-03[&length_mean=3.67642416e-03,length_median=3.39654000e-03,length_95%HPD={6.32127700e-04,7.03769600e-03}],38[&prob=1.00000000e+00,prob_stddev=0.00000000e+00,prob_range={1.00000000e+00,1.00000000e+00},prob(percent)="100",prob+-sd="100+-0"]:6.524647e-04[&length_mean=9.55118760e-04,length_median=6.52464700e-04,length_95%HPD={1.30047100e-06,2.79980600e-03}])[&prob=1.00000000e+00,prob_stddev=0.00000000e+00,prob_range={1.00000000e+00,1.00000000e+00},prob(percent)="100",prob+-sd="100+-0"]:1.029252e-02[&length_mean=1.07617284e-02,length_median=1.02925200e-02,length_95%HPD={5.10799600e-03,1.69708900e-02}],39[&prob=1.00000000e+00,prob_stddev=0.00000000e+00,prob_range={1.00000000e+00,1.00000000e+00},prob(percent)="100",prob+-sd="100+-0"]:3.388866e-03[&length_mean=3.72473436e-03,length_median=3.38886600e-03,length_95%HPD={6.83767800e-04,7.48708400e-03}])[&prob=1.00000000e+00,prob_stddev=0.00000000e+00,prob_range={1.00000000e+00,1.00000000e+00},prob(percent)="100",prob+-sd="100+-0"]:1.610222e-02[&length_mean=1.62776332e-02,length_median=1.61022200e-02,length_95%HPD={9.39028300e-03,2.40632900e-02}],(((48[&prob=1.00000000e+00,prob_stddev=0.00000000e+00,prob_range={1.00000000e+00,1.00000000e+00},prob(percent)="100",prob+-sd="100+-0"]:6.623094e-03[&length_mean=6.91228149e-03,length_median=6.62309400e-03,length_95%HPD={2.91479400e-03,1.15327600e-02}],(49[&prob=1.00000000e+00,prob_stddev=0.00000000e+00,prob_range={1.00000000e+00,1.00000000e+00},prob(percent)="100",prob+-sd="100+-0"]:1.854071e-03[&length_mean=2.07231902e-03,length_median=1.85407100e-03,length_95%HPD={2.00400100e-05,4.59524500e-03}],50[&prob=1.00000000e+00,prob_stddev=0.00000000e+00,prob_range={1.00000000e+00,1.00000000e+00},prob(percent)="100",prob+-sd="100+-0"]:2.706572e-03[&length_mean=3.06906171e-03,length_median=2.70657200e-03,length_95%HPD={5.72431000e-04,6.21622800e-03}])[&prob=7.45672437e-01,prob_stddev=3.76621455e-03,prob_range={7.43009321e-01,7.48335553e-01},prob(percent)="75",prob+-sd="75+-0"]:1.855369e-03[&length_mean=2.08393491e-03,length_median=1.85536900e-03,length_95%HPD={1.47867500e-04,4.83100300e-03}])[&prob=9.38748336e-01,prob_stddev=3.76621455e-03,prob_range={9.36085220e-01,9.41411451e-01},prob(percent)="94",prob+-sd="94+-0"]:2.171556e-03[&length_mean=2.38557920e-03,length_median=2.17155600e-03,length_95%HPD={1.43984200e-04,5.12718500e-03}],51[&prob=1.00000000e+00,prob_stddev=0.00000000e+00,prob_range={1.00000000e+00,1.00000000e+00},prob(percent)="100",prob+-sd="100+-0"]:3.055296e-03[&length_mean=3.38835673e-03,length_median=3.05529600e-03,length_95%HPD={7.30010600e-04,6.76573400e-03}])[&prob=1.00000000e+00,prob_stddev=0.00000000e+00,prob_range={1.00000000e+00,1.00000000e+00},prob(percent)="100",prob+-sd="100+-0"]:7.920031e-03[&length_mean=8.27271276e-03,length_median=7.92003100e-03,length_95%HPD={3.50793400e-03,1.35663700e-02}],((52[&prob=1.00000000e+00,prob_stddev=0.00000000e+00,prob_range={1.00000000e+00,1.00000000e+00},prob(percent)="100",prob+-sd="100+-0"]:5.800595e-04[&length_mean=7.95647172e-04,length_median=5.80059500e-04,length_95%HPD={1.14584300e-06,2.24094100e-03}],53[&prob=1.00000000e+00,prob_stddev=0.00000000e+00,prob_range={1.00000000e+00,1.00000000e+00},prob(percent)="100",prob+-sd="100+-0"]:4.829798e-04[&length_mean=6.91978565e-04,length_median=4.82979800e-04,length_95%HPD={7.25351500e-07,2.01711200e-03}])[&prob=1.00000000e+00,prob_stddev=0.00000000e+00,prob_range={1.00000000e+00,1.00000000e+00},prob(percent)="100",prob+-sd="100+-0"]:8.017482e-03[&length_mean=8.23119782e-03,length_median=8.01748200e-03,length_95%HPD={3.05878200e-03,1.34663000e-02}],(54[&prob=1.00000000e+00,prob_stddev=0.00000000e+00,prob_range={1.00000000e+00,1.00000000e+00},prob(percent)="100",prob+-sd="100+-0"]:8.289650e-04[&length_mean=1.07538149e-03,length_median=8.28965000e-04,length_95%HPD={2.46829200e-06,3.10762800e-03}],55[&prob=1.00000000e+00,prob_stddev=0.00000000e+00,prob_range={1.00000000e+00,1.00000000e+00},prob(percent)="100",prob+-sd="100+-0"]:8.631850e-04[&length_mean=1.10901903e-03,length_median=8.63185000e-04,length_95%HPD={1.09023700e-06,2.93728200e-03}])[&prob=1.00000000e+00,prob_stddev=0.00000000e+00,prob_range={1.00000000e+00,1.00000000e+00},prob(percent)="100",prob+-sd="100+-0"]:4.042027e-03[&length_mean=4.20912840e-03,length_median=4.04202700e-03,length_95%HPD={9.06129200e-04,7.61411100e-03}])[&prob=1.00000000e+00,prob_stddev=0.00000000e+00,prob_range={1.00000000e+00,1.00000000e+00},prob(percent)="100",prob+-sd="100+-0"]:6.469266e-03[&length_mean=6.66917907e-03,length_median=6.46926600e-03,length_95%HPD={2.30064300e-03,1.20884200e-02}])[&prob=1.00000000e+00,prob_stddev=0.00000000e+00,prob_range={1.00000000e+00,1.00000000e+00},prob(percent)="100",prob+-sd="100+-0"]:2.075686e-02[&length_mean=2.12714887e-02,length_median=2.07568600e-02,length_95%HPD={1.24517600e-02,2.96406000e-02}])[&prob=8.73501997e-01,prob_stddev=5.64932182e-03,prob_range={8.69507324e-01,8.77496671e-01},prob(percent)="87",prob+-sd="87+-1"]:4.812894e-03[&length_mean=5.13164840e-03,length_median=4.81289400e-03,length_95%HPD={1.00237100e-03,9.91930100e-03}],(73[&prob=1.00000000e+00,prob_stddev=0.00000000e+00,prob_range={1.00000000e+00,1.00000000e+00},prob(percent)="100",prob+-sd="100+-0"]:5.972735e-03[&length_mean=6.28199655e-03,length_median=5.97273500e-03,length_95%HPD={1.72368900e-03,1.10594900e-02}],74[&prob=1.00000000e+00,prob_stddev=0.00000000e+00,prob_range={1.00000000e+00,1.00000000e+00},prob(percent)="100",prob+-sd="100+-0"]:1.470707e-02[&length_mean=1.49315524e-02,length_median=1.47070700e-02,length_95%HPD={8.73927200e-03,2.27322600e-02}])[&prob=1.00000000e+00,prob_stddev=0.00000000e+00,prob_range={1.00000000e+00,1.00000000e+00},prob(percent)="100",prob+-sd="100+-0"]:2.427566e-02[&length_mean=2.45614504e-02,length_median=2.42756600e-02,length_95%HPD={1.42759100e-02,3.39109800e-02}])[&prob=8.28894807e-01,prob_stddev=2.82466091e-03,prob_range={8.26897470e-01,8.30892144e-01},prob(percent)="83",prob+-sd="83+-0"]:4.079449e-03[&length_mean=4.37904693e-03,length_median=4.07944900e-03,length_95%HPD={3.00213900e-04,8.50141400e-03}],((((40[&prob=1.00000000e+00,prob_stddev=0.00000000e+00,prob_range={1.00000000e+00,1.00000000e+00},prob(percent)="100",prob+-sd="100+-0"]:6.192557e-03[&length_mean=6.51819208e-03,length_median=6.19255700e-03,length_95%HPD={2.16105700e-03,1.15814400e-02}],41[&prob=1.00000000e+00,prob_stddev=0.00000000e+00,prob_range={1.00000000e+00,1.00000000e+00},prob(percent)="100",prob+-sd="100+-0"]:3.333520e-03[&length_mean=3.65989825e-03,length_median=3.33352000e-03,length_95%HPD={4.38428700e-04,7.41322500e-03}])[&prob=1.00000000e+00,prob_stddev=0.00000000e+00,prob_range={1.00000000e+00,1.00000000e+00},prob(percent)="100",prob+-sd="100+-0"]:9.150590e-03[&length_mean=9.45126701e-03,length_median=9.15059000e-03,length_95%HPD={4.08386000e-03,1.48777800e-02}],42[&prob=1.00000000e+00,prob_stddev=0.00000000e+00,prob_range={1.00000000e+00,1.00000000e+00},prob(percent)="100",prob+-sd="100+-0"]:7.795792e-03[&length_mean=8.12025403e-03,length_median=7.79579200e-03,length_95%HPD={2.99593600e-03,1.34611200e-02}])[&prob=1.00000000e+00,prob_stddev=0.00000000e+00,prob_range={1.00000000e+00,1.00000000e+00},prob(percent)="100",prob+-sd="100+-0"]:5.694836e-03[&length_mean=6.03232245e-03,length_median=5.69483600e-03,length_95%HPD={1.43029000e-03,1.07259200e-02}],43[&prob=1.00000000e+00,prob_stddev=0.00000000e+00,prob_range={1.00000000e+00,1.00000000e+00},prob(percent)="100",prob+-sd="100+-0"]:1.334731e-02[&length_mean=1.37602319e-02,length_median=1.33473100e-02,length_95%HPD={6.63443000e-03,2.10012600e-02}])[&prob=1.00000000e+00,prob_stddev=0.00000000e+00,prob_range={1.00000000e+00,1.00000000e+00},prob(percent)="100",prob+-sd="100+-0"]:1.172980e-02[&length_mean=1.19979426e-02,length_median=1.17298000e-02,length_95%HPD={5.56724600e-03,1.91262200e-02}],((44[&prob=1.00000000e+00,prob_stddev=0.00000000e+00,prob_range={1.00000000e+00,1.00000000e+00},prob(percent)="100",prob+-sd="100+-0"]:5.552943e-04[&length_mean=8.04543551e-04,length_median=5.55294300e-04,length_95%HPD={5.49238800e-08,2.42030200e-03}],45[&prob=1.00000000e+00,prob_stddev=0.00000000e+00,prob_range={1.00000000e+00,1.00000000e+00},prob(percent)="100",prob+-sd="100+-0"]:5.814115e-03[&length_mean=6.02202083e-03,length_median=5.81411500e-03,length_95%HPD={2.42068000e-03,1.03419200e-02}])[&prob=9.87350200e-01,prob_stddev=9.41553637e-04,prob_range={9.86684421e-01,9.88015979e-01},prob(percent)="99",prob+-sd="99+-0"]:3.706945e-03[&length_mean=4.00656679e-03,length_median=3.70694500e-03,length_95%HPD={7.48138300e-04,8.30199600e-03}],(46[&prob=1.00000000e+00,prob_stddev=0.00000000e+00,prob_range={1.00000000e+00,1.00000000e+00},prob(percent)="100",prob+-sd="100+-0"]:3.661031e-03[&length_mean=3.94841612e-03,length_median=3.66103100e-03,length_95%HPD={1.15933700e-03,7.73601700e-03}],47[&prob=1.00000000e+00,prob_stddev=0.00000000e+00,prob_range={1.00000000e+00,1.00000000e+00},prob(percent)="100",prob+-sd="100+-0"]:2.825327e-03[&length_mean=3.09907076e-03,length_median=2.82532700e-03,length_95%HPD={3.14770800e-04,6.30460700e-03}])[&prob=1.00000000e+00,prob_stddev=0.00000000e+00,prob_range={1.00000000e+00,1.00000000e+00},prob(percent)="100",prob+-sd="100+-0"]:4.944064e-03[&length_mean=5.29178588e-03,length_median=4.94406400e-03,length_95%HPD={1.08631100e-03,9.83637400e-03}])[&prob=1.00000000e+00,prob_stddev=0.00000000e+00,prob_range={1.00000000e+00,1.00000000e+00},prob(percent)="100",prob+-sd="100+-0"]:9.138713e-03[&length_mean=9.44460957e-03,length_median=9.13871300e-03,length_95%HPD={3.65201100e-03,1.52227100e-02}])[&prob=1.00000000e+00,prob_stddev=0.00000000e+00,prob_range={1.00000000e+00,1.00000000e+00},prob(percent)="100",prob+-sd="100+-0"]:2.422018e-02[&length_mean=2.46218152e-02,length_median=2.42201800e-02,length_95%HPD={1.52273100e-02,3.59263000e-02}])[&prob=5.80559254e-01,prob_stddev=2.82466091e-02,prob_range={5.60585885e-01,6.00532623e-01},prob(percent)="58",prob+-sd="58+-3"]:2.877781e-03[&length_mean=3.38136362e-03,length_median=2.87778100e-03,length_95%HPD={5.14147900e-05,7.75693700e-03}])[&prob=9.56724368e-01,prob_stddev=1.03570900e-02,prob_range={9.49400799e-01,9.64047936e-01},prob(percent)="96",prob+-sd="96+-1"]:7.491501e-03[&length_mean=7.88771717e-03,length_median=7.49150100e-03,length_95%HPD={2.37002700e-03,1.42913400e-02}],(((((75[&prob=1.00000000e+00,prob_stddev=0.00000000e+00,prob_range={1.00000000e+00,1.00000000e+00},prob(percent)="100",prob+-sd="100+-0"]:9.177776e-04[&length_mean=1.20618174e-03,length_median=9.17777600e-04,length_95%HPD={1.99623900e-06,3.29572000e-03}],76[&prob=1.00000000e+00,prob_stddev=0.00000000e+00,prob_range={1.00000000e+00,1.00000000e+00},prob(percent)="100",prob+-sd="100+-0"]:5.308251e-04[&length_mean=7.89315345e-04,length_median=5.30825100e-04,length_95%HPD={6.52604300e-07,2.39626900e-03}],77[&prob=1.00000000e+00,prob_stddev=0.00000000e+00,prob_range={1.00000000e+00,1.00000000e+00},prob(percent)="100",prob+-sd="100+-0"]:4.604836e-04[&length_mean=6.99518568e-04,length_median=4.60483600e-04,length_95%HPD={3.67623100e-07,2.06196900e-03}])[&prob=5.02663116e-01,prob_stddev=1.97726264e-02,prob_range={4.88681758e-01,5.16644474e-01},prob(percent)="50",prob+-sd="50+-2"]:1.462835e-03[&length_mean=1.81058103e-03,length_median=1.46283500e-03,length_95%HPD={3.09834100e-06,4.37103500e-03}],78[&prob=1.00000000e+00,prob_stddev=0.00000000e+00,prob_range={1.00000000e+00,1.00000000e+00},prob(percent)="100",prob+-sd="100+-0"]:3.027814e-03[&length_mean=3.18549630e-03,length_median=3.02781400e-03,length_95%HPD={5.07069200e-04,6.32636600e-03}])[&prob=1.00000000e+00,prob_stddev=0.00000000e+00,prob_range={1.00000000e+00,1.00000000e+00},prob(percent)="100",prob+-sd="100+-0"]:2.311749e-02[&length_mean=2.35106092e-02,length_median=2.31174900e-02,length_95%HPD={1.49225600e-02,3.28632700e-02}],(79[&prob=1.00000000e+00,prob_stddev=0.00000000e+00,prob_range={1.00000000e+00,1.00000000e+00},prob(percent)="100",prob+-sd="100+-0"]:2.520116e-03[&length_mean=2.86755026e-03,length_median=2.52011600e-03,length_95%HPD={4.62155700e-04,6.04050000e-03}],((80[&prob=1.00000000e+00,prob_stddev=0.00000000e+00,prob_range={1.00000000e+00,1.00000000e+00},prob(percent)="100",prob+-sd="100+-0"]:2.028929e-03[&length_mean=2.24095269e-03,length_median=2.02892900e-03,length_95%HPD={2.72403200e-04,4.61063600e-03}],81[&prob=1.00000000e+00,prob_stddev=0.00000000e+00,prob_range={1.00000000e+00,1.00000000e+00},prob(percent)="100",prob+-sd="100+-0"]:5.202406e-04[&length_mean=7.44334893e-04,length_median=5.20240600e-04,length_95%HPD={4.11739500e-07,2.26966700e-03}])[&prob=8.75499334e-01,prob_stddev=6.59087546e-03,prob_range={8.70838881e-01,8.80159787e-01},prob(percent)="88",prob+-sd="88+-1"]:1.324410e-03[&length_mean=1.57320453e-03,length_median=1.32441000e-03,length_95%HPD={5.02962500e-05,3.75204300e-03}],82[&prob=1.00000000e+00,prob_stddev=0.00000000e+00,prob_range={1.00000000e+00,1.00000000e+00},prob(percent)="100",prob+-sd="100+-0"]:4.198402e-03[&length_mean=4.47694703e-03,length_median=4.19840200e-03,length_95%HPD={1.25206800e-03,7.98748500e-03}])[&prob=8.93475366e-01,prob_stddev=0.00000000e+00,prob_range={8.93475366e-01,8.93475366e-01},prob(percent)="89",prob+-sd="89+-0"]:1.339751e-03[&length_mean=1.58678633e-03,length_median=1.33975100e-03,length_95%HPD={5.44290300e-05,3.78663400e-03}])[&prob=1.00000000e+00,prob_stddev=0.00000000e+00,prob_range={1.00000000e+00,1.00000000e+00},prob(percent)="100",prob+-sd="100+-0"]:9.843229e-03[&length_mean=1.01571970e-02,length_median=9.84322900e-03,length_95%HPD={4.38044000e-03,1.73943600e-02}])[&prob=1.00000000e+00,prob_stddev=0.00000000e+00,prob_range={1.00000000e+00,1.00000000e+00},prob(percent)="100",prob+-sd="100+-0"]:1.267474e-02[&length_mean=1.28374338e-02,length_median=1.26747400e-02,length_95%HPD={5.87087300e-03,1.97500600e-02}],((83[&prob=1.00000000e+00,prob_stddev=0.00000000e+00,prob_range={1.00000000e+00,1.00000000e+00},prob(percent)="100",prob+-sd="100+-0"]:8.191599e-04[&length_mean=1.09702683e-03,length_median=8.19159900e-04,length_95%HPD={4.84914100e-07,3.15442900e-03}],84[&prob=1.00000000e+00,prob_stddev=0.00000000e+00,prob_range={1.00000000e+00,1.00000000e+00},prob(percent)="100",prob+-sd="100+-0"]:8.453598e-04[&length_mean=1.08031066e-03,length_median=8.45359800e-04,length_95%HPD={1.71298600e-07,3.02265400e-03}])[&prob=8.27563249e-01,prob_stddev=1.78895191e-02,prob_range={8.14913449e-01,8.40213049e-01},prob(percent)="83",prob+-sd="83+-2"]:2.613755e-03[&length_mean=2.88012006e-03,length_median=2.61375500e-03,length_95%HPD={4.01039600e-04,5.98556400e-03}],85[&prob=1.00000000e+00,prob_stddev=0.00000000e+00,prob_range={1.00000000e+00,1.00000000e+00},prob(percent)="100",prob+-sd="100+-0"]:2.203494e-02[&length_mean=2.24867324e-02,length_median=2.20349400e-02,length_95%HPD={1.44557600e-02,3.19650500e-02}])[&prob=1.00000000e+00,prob_stddev=0.00000000e+00,prob_range={1.00000000e+00,1.00000000e+00},prob(percent)="100",prob+-sd="100+-0"]:9.349339e-03[&length_mean=9.77451567e-03,length_median=9.34933900e-03,length_95%HPD={4.35979500e-03,1.73946800e-02}])[&prob=1.00000000e+00,prob_stddev=0.00000000e+00,prob_range={1.00000000e+00,1.00000000e+00},prob(percent)="100",prob+-sd="100+-0"]:5.369014e-02[&length_mean=5.39155736e-02,length_median=5.36901400e-02,length_95%HPD={3.66552800e-02,7.14933200e-02}],((((86[&prob=1.00000000e+00,prob_stddev=0.00000000e+00,prob_range={1.00000000e+00,1.00000000e+00},prob(percent)="100",prob+-sd="100+-0"]:9.112880e-03[&length_mean=9.37019272e-03,length_median=9.11288000e-03,length_95%HPD={3.10185000e-03,1.56383400e-02}],(87[&prob=1.00000000e+00,prob_stddev=0.00000000e+00,prob_range={1.00000000e+00,1.00000000e+00},prob(percent)="100",prob+-sd="100+-0"]:1.019181e-02[&length_mean=1.06102543e-02,length_median=1.01918100e-02,length_95%HPD={4.52436200e-03,1.65426300e-02}],88[&prob=1.00000000e+00,prob_stddev=0.00000000e+00,prob_range={1.00000000e+00,1.00000000e+00},prob(percent)="100",prob+-sd="100+-0"]:2.126299e-02[&length_mean=2.13640321e-02,length_median=2.12629900e-02,length_95%HPD={1.40483900e-02,2.98504100e-02}])[&prob=8.25565912e-01,prob_stddev=5.46101109e-02,prob_range={7.86950732e-01,8.64181092e-01},prob(percent)="83",prob+-sd="83+-5"]:4.079730e-03[&length_mean=4.45708810e-03,length_median=4.07973000e-03,length_95%HPD={4.35578600e-04,9.04646700e-03}])[&prob=1.00000000e+00,prob_stddev=0.00000000e+00,prob_range={1.00000000e+00,1.00000000e+00},prob(percent)="100",prob+-sd="100+-0"]:9.085111e-02[&length_mean=9.13315816e-02,length_median=9.08511100e-02,length_95%HPD={6.78786200e-02,1.15914500e-01}],((89[&prob=1.00000000e+00,prob_stddev=0.00000000e+00,prob_range={1.00000000e+00,1.00000000e+00},prob(percent)="100",prob+-sd="100+-0"]:8.790121e-03[&length_mean=9.05163455e-03,length_median=8.79012100e-03,length_95%HPD={4.38929600e-03,1.42062800e-02}],91[&prob=1.00000000e+00,prob_stddev=0.00000000e+00,prob_range={1.00000000e+00,1.00000000e+00},prob(percent)="100",prob+-sd="100+-0"]:4.511607e-03[&length_mean=4.71658886e-03,length_median=4.51160700e-03,length_95%HPD={1.41280200e-03,8.57260000e-03}])[&prob=7.66311585e-01,prob_stddev=1.78895191e-02,prob_range={7.53661784e-01,7.78961385e-01},prob(percent)="77",prob+-sd="77+-2"]:3.433568e-03[&length_mean=3.66856994e-03,length_median=3.43356800e-03,length_95%HPD={1.14177700e-04,7.19091700e-03}],90[&prob=1.00000000e+00,prob_stddev=0.00000000e+00,prob_range={1.00000000e+00,1.00000000e+00},prob(percent)="100",prob+-sd="100+-0"]:3.230344e-03[&length_mean=3.58508811e-03,length_median=3.23034400e-03,length_95%HPD={3.80782400e-05,7.64151000e-03}])[&prob=1.00000000e+00,prob_stddev=0.00000000e+00,prob_range={1.00000000e+00,1.00000000e+00},prob(percent)="100",prob+-sd="100+-0"]:5.078232e-02[&length_mean=5.17220037e-02,length_median=5.07823200e-02,length_95%HPD={3.35745800e-02,7.00082300e-02}])[&prob=1.00000000e+00,prob_stddev=0.00000000e+00,prob_range={1.00000000e+00,1.00000000e+00},prob(percent)="100",prob+-sd="100+-0"]:3.271749e-02[&length_mean=3.36061891e-02,length_median=3.27174900e-02,length_95%HPD={1.62369500e-02,5.19281800e-02}],(92[&prob=1.00000000e+00,prob_stddev=0.00000000e+00,prob_range={1.00000000e+00,1.00000000e+00},prob(percent)="100",prob+-sd="100+-0"]:4.081702e-03[&length_mean=4.40780544e-03,length_median=4.08170200e-03,length_95%HPD={2.29091000e-04,8.94636200e-03}],(93[&prob=1.00000000e+00,prob_stddev=0.00000000e+00,prob_range={1.00000000e+00,1.00000000e+00},prob(percent)="100",prob+-sd="100+-0"]:3.296828e-03[&length_mean=3.64414533e-03,length_median=3.29682800e-03,length_95%HPD={8.16862500e-04,7.22382000e-03}],94[&prob=1.00000000e+00,prob_stddev=0.00000000e+00,prob_range={1.00000000e+00,1.00000000e+00},prob(percent)="100",prob+-sd="100+-0"]:1.407277e-02[&length_mean=1.42951430e-02,length_median=1.40727700e-02,length_95%HPD={8.23075900e-03,2.06443900e-02}])[&prob=1.00000000e+00,prob_stddev=0.00000000e+00,prob_range={1.00000000e+00,1.00000000e+00},prob(percent)="100",prob+-sd="100+-0"]:7.878509e-03[&length_mean=8.04343915e-03,length_median=7.87850900e-03,length_95%HPD={3.17985400e-03,1.34540800e-02}])[&prob=1.00000000e+00,prob_stddev=0.00000000e+00,prob_range={1.00000000e+00,1.00000000e+00},prob(percent)="100",prob+-sd="100+-0"]:8.783841e-02[&length_mean=8.83017869e-02,length_median=8.78384100e-02,length_95%HPD={6.44433100e-02,1.14443800e-01}])[&prob=1.00000000e+00,prob_stddev=0.00000000e+00,prob_range={1.00000000e+00,1.00000000e+00},prob(percent)="100",prob+-sd="100+-0"]:4.317366e-02[&length_mean=4.41093888e-02,length_median=4.31736600e-02,length_95%HPD={2.28746100e-02,6.39980000e-02}],(95[&prob=1.00000000e+00,prob_stddev=0.00000000e+00,prob_range={1.00000000e+00,1.00000000e+00},prob(percent)="100",prob+-sd="100+-0"]:8.194863e-03[&length_mean=8.64060133e-03,length_median=8.19486300e-03,length_95%HPD={2.56235600e-03,1.49362900e-02}],(96[&prob=1.00000000e+00,prob_stddev=0.00000000e+00,prob_range={1.00000000e+00,1.00000000e+00},prob(percent)="100",prob+-sd="100+-0"]:2.401546e-03[&length_mean=2.71885620e-03,length_median=2.40154600e-03,length_95%HPD={2.94048300e-06,5.98546000e-03}],97[&prob=1.00000000e+00,prob_stddev=0.00000000e+00,prob_range={1.00000000e+00,1.00000000e+00},prob(percent)="100",prob+-sd="100+-0"]:1.983933e-02[&length_mean=2.01446489e-02,length_median=1.98393300e-02,length_95%HPD={1.17678700e-02,2.82004100e-02}])[&prob=8.48202397e-01,prob_stddev=1.31817509e-02,prob_range={8.38881491e-01,8.57523302e-01},prob(percent)="85",prob+-sd="85+-1"]:4.712702e-03[&length_mean=4.97345748e-03,length_median=4.71270200e-03,length_95%HPD={5.55330100e-04,9.31303600e-03}])[&prob=1.00000000e+00,prob_stddev=0.00000000e+00,prob_range={1.00000000e+00,1.00000000e+00},prob(percent)="100",prob+-sd="100+-0"]:1.413489e-01[&length_mean=1.41830373e-01,length_median=1.41348900e-01,length_95%HPD={1.11745300e-01,1.77589900e-01}])[&prob=1.00000000e+00,prob_stddev=0.00000000e+00,prob_range={1.00000000e+00,1.00000000e+00},prob(percent)="100",prob+-sd="100+-0"]:9.349986e-02[&length_mean=9.45549880e-02,length_median=9.34998600e-02,length_95%HPD={6.95698200e-02,1.24129600e-01}])[&prob=9.99334221e-01,prob_stddev=9.41553637e-04,prob_range={9.98668442e-01,1.00000000e+00},prob(percent)="100",prob+-sd="100+-0"]:2.145490e-02[&length_mean=2.19781122e-02,length_median=2.14549000e-02,length_95%HPD={8.42502700e-03,3.49013400e-02}])[&prob=7.08388815e-01,prob_stddev=2.63635018e-02,prob_range={6.89747004e-01,7.27030626e-01},prob(percent)="71",prob+-sd="71+-3"]:3.695647e-03[&length_mean=4.49149210e-03,length_median=3.69564700e-03,length_95%HPD={9.16522400e-07,1.12347300e-02}])[&prob=1.00000000e+00,prob_stddev=0.00000000e+00,prob_range={1.00000000e+00,1.00000000e+00},prob(percent)="100",prob+-sd="100+-0"]:9.298172e-03[&length_mean=9.49483253e-03,length_median=9.29817200e-03,length_95%HPD={3.52093500e-03,1.54998400e-02}])[&prob=9.98668442e-01,prob_stddev=1.88310727e-03,prob_range={9.97336884e-01,1.00000000e+00},prob(percent)="100",prob+-sd="100+-0"]:6.836514e-03[&length_mean=7.15711529e-03,length_median=6.83651400e-03,length_95%HPD={1.98010300e-03,1.25734500e-02}])[&prob=1.00000000e+00,prob_stddev=0.00000000e+00,prob_range={1.00000000e+00,1.00000000e+00},prob(percent)="100",prob+-sd="100+-0"]:8.532854e-03[&length_mean=8.77888297e-03,length_median=8.53285400e-03,length_95%HPD={3.35755000e-03,1.40261900e-02}]);

end;
