# Supplementary material for: Three new species of arbuscular mycorrhizal fungi (Glomeromycota) and Acaulospora gedanensis revised
Source: Front Microbiol. 2024 Feb 12;15:1320014. doi: 10.3389/fmicb.2024.1320014 (PMC10896085; doi:10.3389/fmicb.2024.1320014)
Supplement: Supplementary Table 7 — Data obtained from a RAxML-NG analysis of 45S sequences (see Supplementary Figure S1). [file Table_7.DOCX]

(((Diversispora_valentina_MT985516:0.005809,Diversispora_valentina_MT985515:0.015388)100:0.025764,((Diversispora_slowinskiensis_KT444720:0.002775,((Diversispora_slowinskiensis_KT444719:0.000827,Diversispora_slowinskiensis_KT444717:0.004090)81:0.003262,Diversispora_slowinskiensis_KT444718:0.000001)95:0.011042)100:0.016866,(((Diversispora_arenaria_KJ850188:0.006538,(Diversispora_arenaria_KJ850187:0.002487,Diversispora_arenaria_KJ850189:0.001525)53:0.001498)78:0.001606,Diversispora_arenaria_KJ850186:0.002562)99:0.007902,((Diversispora_jakucsiae_KJ850184:0.000001,Diversispora_jakucsiae_KJ850183:0.000793)81:0.003460,(Diversispora_jakucsiae_KJ850182:0.000001,Diversispora_jakucsiae_KJ850181:0.000001)100:0.008425)98:0.005613)100:0.021962)40:0.003783)38:0.003691,(((Diversispora_sporocarpia_MK036785:0.000001,Diversispora_sporocarpia_MK036786:0.005728)86:0.003597,(Diversispora_sporocarpia_MK036789:0.002465,Diversispora_sporocarpia_MK036788:0.003295)100:0.004702)89:0.009198,(Diversispora_epigaea_FM876818:0.014050,((Diversispora_epigaea_FM876814:0.006242,Diversispora_epigaea_FM876817:0.002875)99:0.009380,Diversispora_epigaea_FM876819:0.007671)94:0.005478)99:0.011889)100:0.027174,(((((Diversispora_insculpta_KJ850195:0.000001,(Diversispora_insculpta_KJ850197:0.000001,Diversispora_insculpta_KJ850196:0.000001)30:0.000001)100:0.015306,((Diversispora_aestuarii_OL684645:0.016144,(Diversispora_aestuarii_OL684642:0.012490,(Diversispora_aestuarii_OL684648:0.011330,Diversispora_aestuarii_OL684644:0.026706)54:0.009595)44:0.003390)81:0.005587,((Diversispora_varaderana_KT444710:0.000853,(Diversispora_varaderana_KT444711:0.006490,Diversispora_varaderana_KT444709:0.005377)62:0.001908)56:0.001640,Diversispora_varaderana_KT444708:0.006754)72:0.003276)58:0.006149)99:0.016161,(((Diversispora_marina_MT725499:0.000001,Diversispora_marina_MT725498:0.000001)92:0.000861,(Diversispora_marina_MT725502:0.000001,Diversispora_marina_MT725501:0.000001)68:0.000813)99:0.017838,((448_4_SSU_ITS_LSU_13_08_2021:0.009664,448_3_:0.000001)97:0.008470,((Diversispora_densissima_MT724382:0.000001,Diversispora_densissima_MT724383:0.000001)100:0.006194,Diversispora_densissima_MT724384:0.006378)100:0.013103)75:0.007236)83:0.009556)61:0.002732,((((Desertispora_omaniana_MG459208:0.002498,Desertispora_omaniana_KF154769:0.020757)54:0.004587,Desertispora_omaniana_KF154770:0.007788)100:0.161847,((Siverdingia_tortuosa_JF439094:0.003551,(Siverdingia_tortuosa_JF439095:0.014621,Siverdingia_tortuosa_JF439096:0.003044)84:0.008289)100:0.099345,((Redeckera_megalocarpum_HG518628:0.002068,(Redeckera_megalocarpum_HG518627:0.008705,Redeckera_megalocarpum_HG518629:0.004614)65:0.003459)100:0.055837,(Corymbiglomus_corymbiforme_KF060295:0.008457,(Corymbiglomus_corymbiforme_KF060296:0.022254,Corymbiglomus_corymbiforme_KF060298:0.010563)71:0.003875)100:0.100805)93:0.035071)100:0.049530)100:0.108139,((Diversispora_trimurales_KJ850200:0.000001,(Diversispora_trimurales_KJ850199:0.000001,Diversispora_trimurales_KJ850198:0.025204)46:0.000817)100:0.010966,((Diversispora_peridiata_KT444712:0.002398,(Diversispora_peridiata_KT444714:0.003992,(Diversispora_peridiata_KT444713:0.000001,Diversispora_peridiata_KT444715:0.001580)82:0.000778)64:0.000790)99:0.010515,(Diversispora_gibbosa_KJ850201:0.000001,((Diversispora_gibbosa_KJ850202:0.000001,Diversispora_gibbosa_KJ850203:0.000001)71:0.000001,Diversispora_gibbosa_KJ850204:0.003168)47:0.000787)100:0.024253)80:0.011801)100:0.058660)77:0.021367)58:0.007165,((((((Diversispora_sabulosa_MG459214:0.000001,Diversispora_sabulosa_MG459215:0.000001)86:0.000811,Diversispora_sabulosa_MG459213:0.000001)100:0.007176,(Diversispora_sabulosa_MG459211:0.000001,Diversispora_sabulosa_MG459212:0.000001)100:0.006012)100:0.043374,((Diversispora_alba_OP195882:0.004420,((Diversispora_alba_OP195886:0.003095,Diversispora_alba_OP195889:0.008719)96:0.009249,Diversispora_alba_OP195880:0.007096)54:0.002272)99:0.015884,(((Diversispora_aurantia_FN547655:0.001682,Diversispora_aurantia_FN547657:0.002399)94:0.004260,(Diversispora_aurantia_FN547661:0.000001,Diversispora_aurantia_FN547664:0.000001)100:0.005738)100:0.017181,((Diversispora_spurca_FN547639:0.008296,(Diversispora_spurca_FN547637:0.009150,Diversispora_spurca_FN547644:0.002378)54:0.003073)100:0.017472,Diversispora_spurca_MG459207:0.026373)65:0.005582)36:0.004237)99:0.016591)91:0.013602,(((Diversispora_eburnea_AM713411:0.000001,(Diversispora_eburnea_AM713408:0.000001,Diversispora_eburnea_AM713407:0.000001)98:0.001640)98:0.001634,Diversispora_eburnea_AM713406:0.008331)99:0.010691,(Diversispora_celata_AM713402:0.003403,(Diversispora_celata_AM713403:0.000001,Diversispora_celata_AY639225:0.001835)93:0.006162)100:0.023831)100:0.013564)65:0.005601,((Diversispora_peloponnesiaca_MN306206:0.006712,(Diversispora_peloponnesiaca_MN306205:0.017389,(Diversispora_peloponnesiaca_MN306207:0.001816,Diversispora_peloponnesiaca_MN306208:0.009570)75:0.006052)62:0.002683)73:0.003433,(Diversispora_clara_FR873629:0.005899,(Diversispora_clara_FR873630:0.001605,(Diversispora_clara_FR873631:0.003213,Diversispora_clara_FR873632:0.008109)2:0.000001)42:0.003045)80:0.004444)100:0.023062)51:0.005141)17:0.002716);
