# Supplementary material for: Three new species of arbuscular mycorrhizal fungi (Glomeromycota) and Acaulospora gedanensis revised
Source: Front Microbiol. 2024 Feb 12;15:1320014. doi: 10.3389/fmicb.2024.1320014 (PMC10896085; doi:10.3389/fmicb.2024.1320014)
Supplement: Supplementary Table 8 — Data obtained from a BI analysis of 45S+rpb1 sequences (see Figure 1). [file Table_8.DOCX]

#NEXUS

[ID: 7590616702]

begin taxa;

dimensions ntax=97;

taxlabels

448_3_

448_4_SSU_ITS_LSU_13_08_2021

Diversispora_densissima_MT724382

Diversispora_densissima_MT724383

Diversispora_densissima_MT724384

Diversispora_marina_MT725498

Diversispora_marina_MT725499

Diversispora_marina_MT725501

Diversispora_marina_MT725502

Diversispora_insculpta_KJ850195

Diversispora_insculpta_KJ850196

Diversispora_insculpta_KJ850197

Diversispora_varaderana_KT444708

Diversispora_varaderana_KT444709

Diversispora_varaderana_KT444710

Diversispora_varaderana_KT444711

Diversispora_aestuarii_OL684642

Diversispora_aestuarii_OL684645

Diversispora_aestuarii_OL684648

Diversispora_aestuarii_OL684644

Diversispora_clara_FR873629

Diversispora_clara_FR873632

Diversispora_clara_FR873630

Diversispora_clara_FR873631

Diversispora_peloponnesiaca_MN306206

Diversispora_peloponnesiaca_MN306207

Diversispora_peloponnesiaca_MN306208

Diversispora_peloponnesiaca_MN306205

Diversispora_celata_AM713402

Diversispora_celata_AM713403

Diversispora_celata_AY639225

Diversispora_eburnea_AM713407

Diversispora_eburnea_AM713408

Diversispora_eburnea_AM713411

Diversispora_eburnea_AM713406

Diversispora_slowinskiensis_KT444717

Diversispora_slowinskiensis_KT444719

Diversispora_slowinskiensis_KT444718

Diversispora_slowinskiensis_KT444720

Diversispora_epigaea_FM876814

Diversispora_epigaea_FM876817

Diversispora_epigaea_FM876819

Diversispora_epigaea_FM876818

Diversispora_sporocarpia_MK036785

Diversispora_sporocarpia_MK036786

Diversispora_sporocarpia_MK036788

Diversispora_sporocarpia_MK036789

Diversispora_arenaria_KJ850188

Diversispora_arenaria_KJ850189

Diversispora_arenaria_KJ850187

Diversispora_arenaria_KJ850186

Diversispora_jakucsiae_KJ850181

Diversispora_jakucsiae_KJ850182

Diversispora_jakucsiae_KJ850183

Diversispora_jakucsiae_KJ850184

Diversispora_aurantia_FN547661

Diversispora_aurantia_FN547664

Diversispora_aurantia_FN547655

Diversispora_aurantia_FN547657

Diversispora_alba_OP195880

Diversispora_alba_OP195882

Diversispora_alba_OP195886

Diversispora_alba_OP195889

Diversispora_spurca_MG459207

Diversispora_spurca_FN547637

Diversispora_spurca_FN547644

Diversispora_spurca_FN547639

Diversispora_sabulosa_MG459211

Diversispora_sabulosa_MG459212

Diversispora_sabulosa_MG459215

Diversispora_sabulosa_MG459214

Diversispora_sabulosa_MG459213

Diversispora_valentina_MT985516

Diversispora_valentina_MT985515

Diversispora_gibbosa_KJ850201

Diversispora_gibbosa_KJ850202

Diversispora_gibbosa_KJ850203

Diversispora_gibbosa_KJ850204

Diversispora_peridiata_KT444712

Diversispora_peridiata_KT444715

Diversispora_peridiata_KT444713

Diversispora_peridiata_KT444714

Diversispora_trimurales_KJ850199

Diversispora_trimurales_KJ850200

Diversispora_trimurales_KJ850198

Corymbiglomus_corymbiforme_KF060295

Corymbiglomus_corymbiforme_KF060298

Corymbiglomus_corymbiforme_KF060296

Redeckera_megalocarpum_HG518627

Redeckera_megalocarpum_HG518628

Redeckera_megalocarpum_HG518629

Siverdingia_tortuosa_JF439094

Siverdingia_tortuosa_JF439096

Siverdingia_tortuosa_JF439095

Desertispora_omaniana_KF154770

Desertispora_omaniana_MG459208

Desertispora_omaniana_KF154769

;

end;

begin trees;

translate

1 448_3_,

2 448_4_SSU_ITS_LSU_13_08_2021,

3 Diversispora_densissima_MT724382,

4 Diversispora_densissima_MT724383,

5 Diversispora_densissima_MT724384,

6 Diversispora_marina_MT725498,

7 Diversispora_marina_MT725499,

8 Diversispora_marina_MT725501,

9 Diversispora_marina_MT725502,

10 Diversispora_insculpta_KJ850195,

11 Diversispora_insculpta_KJ850196,

12 Diversispora_insculpta_KJ850197,

13 Diversispora_varaderana_KT444708,

14 Diversispora_varaderana_KT444709,

15 Diversispora_varaderana_KT444710,

16 Diversispora_varaderana_KT444711,

17 Diversispora_aestuarii_OL684642,

18 Diversispora_aestuarii_OL684645,

19 Diversispora_aestuarii_OL684648,

20 Diversispora_aestuarii_OL684644,

21 Diversispora_clara_FR873629,

22 Diversispora_clara_FR873632,

23 Diversispora_clara_FR873630,

24 Diversispora_clara_FR873631,

25 Diversispora_peloponnesiaca_MN306206,

26 Diversispora_peloponnesiaca_MN306207,

27 Diversispora_peloponnesiaca_MN306208,

28 Diversispora_peloponnesiaca_MN306205,

29 Diversispora_celata_AM713402,

30 Diversispora_celata_AM713403,

31 Diversispora_celata_AY639225,

32 Diversispora_eburnea_AM713407,

33 Diversispora_eburnea_AM713408,

34 Diversispora_eburnea_AM713411,

35 Diversispora_eburnea_AM713406,

36 Diversispora_slowinskiensis_KT444717,

37 Diversispora_slowinskiensis_KT444719,

38 Diversispora_slowinskiensis_KT444718,

39 Diversispora_slowinskiensis_KT444720,

40 Diversispora_epigaea_FM876814,

41 Diversispora_epigaea_FM876817,

42 Diversispora_epigaea_FM876819,

43 Diversispora_epigaea_FM876818,

44 Diversispora_sporocarpia_MK036785,

45 Diversispora_sporocarpia_MK036786,

46 Diversispora_sporocarpia_MK036788,

47 Diversispora_sporocarpia_MK036789,

48 Diversispora_arenaria_KJ850188,

49 Diversispora_arenaria_KJ850189,

50 Diversispora_arenaria_KJ850187,

51 Diversispora_arenaria_KJ850186,

52 Diversispora_jakucsiae_KJ850181,

53 Diversispora_jakucsiae_KJ850182,

54 Diversispora_jakucsiae_KJ850183,

55 Diversispora_jakucsiae_KJ850184,

56 Diversispora_aurantia_FN547661,

57 Diversispora_aurantia_FN547664,

58 Diversispora_aurantia_FN547655,

59 Diversispora_aurantia_FN547657,

60 Diversispora_alba_OP195880,

61 Diversispora_alba_OP195882,

62 Diversispora_alba_OP195886,

63 Diversispora_alba_OP195889,

64 Diversispora_spurca_MG459207,

65 Diversispora_spurca_FN547637,

66 Diversispora_spurca_FN547644,

67 Diversispora_spurca_FN547639,

68 Diversispora_sabulosa_MG459211,

69 Diversispora_sabulosa_MG459212,

70 Diversispora_sabulosa_MG459215,

71 Diversispora_sabulosa_MG459214,

72 Diversispora_sabulosa_MG459213,

73 Diversispora_valentina_MT985516,

74 Diversispora_valentina_MT985515,

75 Diversispora_gibbosa_KJ850201,

76 Diversispora_gibbosa_KJ850202,

77 Diversispora_gibbosa_KJ850203,

78 Diversispora_gibbosa_KJ850204,

79 Diversispora_peridiata_KT444712,

80 Diversispora_peridiata_KT444715,

81 Diversispora_peridiata_KT444713,

82 Diversispora_peridiata_KT444714,

83 Diversispora_trimurales_KJ850199,

84 Diversispora_trimurales_KJ850200,

85 Diversispora_trimurales_KJ850198,

86 Corymbiglomus_corymbiforme_KF060295,

87 Corymbiglomus_corymbiforme_KF060298,

88 Corymbiglomus_corymbiforme_KF060296,

89 Redeckera_megalocarpum_HG518627,

90 Redeckera_megalocarpum_HG518628,

91 Redeckera_megalocarpum_HG518629,

92 Siverdingia_tortuosa_JF439094,

93 Siverdingia_tortuosa_JF439096,

94 Siverdingia_tortuosa_JF439095,

95 Desertispora_omaniana_KF154770,

96 Desertispora_omaniana_MG459208,

97 Desertispora_omaniana_KF154769

;

tree con_50_majrule = [&U] (1[&prob=1.00000000e+00,prob_stddev=0.00000000e+00,prob_range={1.00000000e+00,1.00000000e+00},prob(percent)="100",prob+-sd="100+-0"]:1.283135e-03[&length_mean=1.44216447e-03,length_median=1.28313500e-03,length_95%HPD={6.89166100e-05,3.13973900e-03}],2[&prob=1.00000000e+00,prob_stddev=0.00000000e+00,prob_range={1.00000000e+00,1.00000000e+00},prob(percent)="100",prob+-sd="100+-0"]:8.076260e-03[&length_mean=8.30508405e-03,length_median=8.07626000e-03,length_95%HPD={4.53953400e-03,1.27642600e-02}],(((3[&prob=1.00000000e+00,prob_stddev=0.00000000e+00,prob_range={1.00000000e+00,1.00000000e+00},prob(percent)="100",prob+-sd="100+-0"]:3.856017e-04[&length_mean=5.89595390e-04,length_median=3.85601700e-04,length_95%HPD={3.83845800e-07,1.73498800e-03}],4[&prob=1.00000000e+00,prob_stddev=0.00000000e+00,prob_range={1.00000000e+00,1.00000000e+00},prob(percent)="100",prob+-sd="100+-0"]:3.456742e-04[&length_mean=5.13751421e-04,length_median=3.45674200e-04,length_95%HPD={8.11417000e-07,1.55167800e-03}])[&prob=1.00000000e+00,prob_stddev=0.00000000e+00,prob_range={1.00000000e+00,1.00000000e+00},prob(percent)="100",prob+-sd="100+-0"]:3.986311e-03[&length_mean=4.27899778e-03,length_median=3.98631100e-03,length_95%HPD={1.70263900e-03,7.75612900e-03}],5[&prob=1.00000000e+00,prob_stddev=0.00000000e+00,prob_range={1.00000000e+00,1.00000000e+00},prob(percent)="100",prob+-sd="100+-0"]:4.143166e-03[&length_mean=4.29535245e-03,length_median=4.14316600e-03,length_95%HPD={1.68157900e-03,7.51564300e-03}])[&prob=1.00000000e+00,prob_stddev=0.00000000e+00,prob_range={1.00000000e+00,1.00000000e+00},prob(percent)="100",prob+-sd="100+-0"]:8.470076e-03[&length_mean=8.72161290e-03,length_median=8.47007600e-03,length_95%HPD={4.71597400e-03,1.33007400e-02}],(((6[&prob=1.00000000e+00,prob_stddev=0.00000000e+00,prob_range={1.00000000e+00,1.00000000e+00},prob(percent)="100",prob+-sd="100+-0"]:4.109470e-04[&length_mean=5.95864539e-04,length_median=4.10947000e-04,length_95%HPD={1.85452900e-06,1.76650100e-03}],7[&prob=1.00000000e+00,prob_stddev=0.00000000e+00,prob_range={1.00000000e+00,1.00000000e+00},prob(percent)="100",prob+-sd="100+-0"]:4.228616e-04[&length_mean=6.09337288e-04,length_median=4.22861600e-04,length_95%HPD={7.29629900e-07,1.75005500e-03}])[&prob=9.83355526e-01,prob_stddev=9.41553637e-04,prob_range={9.82689747e-01,9.84021305e-01},prob(percent)="98",prob+-sd="98+-0"]:1.099556e-03[&length_mean=1.31274012e-03,length_median=1.09955600e-03,length_95%HPD={5.85108600e-06,3.26765600e-03}],(8[&prob=1.00000000e+00,prob_stddev=0.00000000e+00,prob_range={1.00000000e+00,1.00000000e+00},prob(percent)="100",prob+-sd="100+-0"]:3.970027e-04[&length_mean=5.75843282e-04,length_median=3.97002700e-04,length_95%HPD={1.62883300e-06,1.67300000e-03}],9[&prob=1.00000000e+00,prob_stddev=0.00000000e+00,prob_range={1.00000000e+00,1.00000000e+00},prob(percent)="100",prob+-sd="100+-0"]:4.430375e-04[&length_mean=6.12971865e-04,length_median=4.43037500e-04,length_95%HPD={1.61727300e-06,1.77615800e-03}])[&prob=9.70039947e-01,prob_stddev=4.70776818e-03,prob_range={9.66711052e-01,9.73368842e-01},prob(percent)="97",prob+-sd="97+-0"]:9.973647e-04[&length_mean=1.19423375e-03,length_median=9.97364700e-04,length_95%HPD={3.48228700e-05,2.75734100e-03}])[&prob=1.00000000e+00,prob_stddev=0.00000000e+00,prob_range={1.00000000e+00,1.00000000e+00},prob(percent)="100",prob+-sd="100+-0"]:1.312126e-02[&length_mean=1.32066220e-02,length_median=1.31212600e-02,length_95%HPD={7.35752800e-03,1.83736300e-02}],(((((10[&prob=1.00000000e+00,prob_stddev=0.00000000e+00,prob_range={1.00000000e+00,1.00000000e+00},prob(percent)="100",prob+-sd="100+-0"]:2.147329e-03[&length_mean=2.29573000e-03,length_median=2.14732900e-03,length_95%HPD={1.45020500e-05,4.80323800e-03}],11[&prob=1.00000000e+00,prob_stddev=0.00000000e+00,prob_range={1.00000000e+00,1.00000000e+00},prob(percent)="100",prob+-sd="100+-0"]:4.134474e-04[&length_mean=6.04528055e-04,length_median=4.13447400e-04,length_95%HPD={6.51873600e-07,1.81252300e-03}])[&prob=6.58455393e-01,prob_stddev=6.59087546e-03,prob_range={6.53794940e-01,6.63115846e-01},prob(percent)="66",prob+-sd="66+-1"]:1.677973e-03[&length_mean=1.82040283e-03,length_median=1.67797300e-03,length_95%HPD={1.95260600e-06,4.32464600e-03}],12[&prob=1.00000000e+00,prob_stddev=0.00000000e+00,prob_range={1.00000000e+00,1.00000000e+00},prob(percent)="100",prob+-sd="100+-0"]:1.084012e-03[&length_mean=1.27021774e-03,length_median=1.08401200e-03,length_95%HPD={8.54266700e-06,2.93615500e-03}])[&prob=1.00000000e+00,prob_stddev=0.00000000e+00,prob_range={1.00000000e+00,1.00000000e+00},prob(percent)="100",prob+-sd="100+-0"]:1.104432e-02[&length_mean=1.11749575e-02,length_median=1.10443200e-02,length_95%HPD={6.14307200e-03,1.67702100e-02}],(13[&prob=1.00000000e+00,prob_stddev=0.00000000e+00,prob_range={1.00000000e+00,1.00000000e+00},prob(percent)="100",prob+-sd="100+-0"]:4.234051e-03[&length_mean=4.30355442e-03,length_median=4.23405100e-03,length_95%HPD={1.21130100e-03,7.14987600e-03}],((14[&prob=1.00000000e+00,prob_stddev=0.00000000e+00,prob_range={1.00000000e+00,1.00000000e+00},prob(percent)="100",prob+-sd="100+-0"]:4.439664e-03[&length_mean=4.59154337e-03,length_median=4.43966400e-03,length_95%HPD={1.88423300e-03,7.77689900e-03}],16[&prob=1.00000000e+00,prob_stddev=0.00000000e+00,prob_range={1.00000000e+00,1.00000000e+00},prob(percent)="100",prob+-sd="100+-0"]:4.546639e-03[&length_mean=4.61782467e-03,length_median=4.54663900e-03,length_95%HPD={1.93329100e-03,7.38418100e-03}])[&prob=9.50066578e-01,prob_stddev=1.22401973e-02,prob_range={9.41411451e-01,9.58721704e-01},prob(percent)="95",prob+-sd="95+-1"]:1.566649e-03[&length_mean=1.74587123e-03,length_median=1.56664900e-03,length_95%HPD={3.09761600e-04,3.82990200e-03}],15[&prob=1.00000000e+00,prob_stddev=0.00000000e+00,prob_range={1.00000000e+00,1.00000000e+00},prob(percent)="100",prob+-sd="100+-0"]:1.043979e-03[&length_mean=1.30342557e-03,length_median=1.04397900e-03,length_95%HPD={2.73519200e-05,3.16973200e-03}])[&prob=9.56724368e-01,prob_stddev=9.41553637e-04,prob_range={9.56058589e-01,9.57390146e-01},prob(percent)="96",prob+-sd="96+-0"]:1.660565e-03[&length_mean=1.80925258e-03,length_median=1.66056500e-03,length_95%HPD={1.22674700e-04,3.68605000e-03}])[&prob=9.98002663e-01,prob_stddev=9.41553637e-04,prob_range={9.97336884e-01,9.98668442e-01},prob(percent)="100",prob+-sd="100+-0"]:3.585756e-03[&length_mean=3.80058162e-03,length_median=3.58575600e-03,length_95%HPD={9.80384000e-04,7.36051100e-03}])[&prob=7.70972037e-01,prob_stddev=6.77918618e-02,prob_range={7.23035952e-01,8.18908123e-01},prob(percent)="77",prob+-sd="77+-7"]:3.505234e-03[&length_mean=3.65812451e-03,length_median=3.50523400e-03,length_95%HPD={8.15337100e-04,6.43298400e-03}],((17[&prob=1.00000000e+00,prob_stddev=0.00000000e+00,prob_range={1.00000000e+00,1.00000000e+00},prob(percent)="100",prob+-sd="100+-0"]:6.747393e-03[&length_mean=6.91439802e-03,length_median=6.74739300e-03,length_95%HPD={3.30596500e-03,1.12090900e-02}],(19[&prob=1.00000000e+00,prob_stddev=0.00000000e+00,prob_range={1.00000000e+00,1.00000000e+00},prob(percent)="100",prob+-sd="100+-0"]:9.848357e-03[&length_mean=1.01732882e-02,length_median=9.84835700e-03,length_95%HPD={4.71013000e-03,1.57826600e-02}],20[&prob=1.00000000e+00,prob_stddev=0.00000000e+00,prob_range={1.00000000e+00,1.00000000e+00},prob(percent)="100",prob+-sd="100+-0"]:1.489822e-02[&length_mean=1.50800274e-02,length_median=1.48982200e-02,length_95%HPD={9.54497700e-03,2.10802700e-02}])[&prob=9.98668442e-01,prob_stddev=1.88310727e-03,prob_range={9.97336884e-01,1.00000000e+00},prob(percent)="100",prob+-sd="100+-0"]:5.880156e-03[&length_mean=6.02472009e-03,length_median=5.88015600e-03,length_95%HPD={3.16901600e-03,9.86078000e-03}])[&prob=1.00000000e+00,prob_stddev=0.00000000e+00,prob_range={1.00000000e+00,1.00000000e+00},prob(percent)="100",prob+-sd="100+-0"]:5.855780e-03[&length_mean=6.07955014e-03,length_median=5.85578000e-03,length_95%HPD={1.53470900e-03,1.09178000e-02}],18[&prob=1.00000000e+00,prob_stddev=0.00000000e+00,prob_range={1.00000000e+00,1.00000000e+00},prob(percent)="100",prob+-sd="100+-0"]:1.195129e-02[&length_mean=1.20880666e-02,length_median=1.19512900e-02,length_95%HPD={7.02479600e-03,1.80194700e-02}])[&prob=9.99334221e-01,prob_stddev=9.41553637e-04,prob_range={9.98668442e-01,1.00000000e+00},prob(percent)="100",prob+-sd="100+-0"]:4.990617e-03[&length_mean=5.25963817e-03,length_median=4.99061700e-03,length_95%HPD={1.51042400e-03,1.03393300e-02}])[&prob=1.00000000e+00,prob_stddev=0.00000000e+00,prob_range={1.00000000e+00,1.00000000e+00},prob(percent)="100",prob+-sd="100+-0"]:1.252949e-02[&length_mean=1.27176664e-02,length_median=1.25294900e-02,length_95%HPD={7.50702200e-03,1.83259900e-02}],((((((21[&prob=1.00000000e+00,prob_stddev=0.00000000e+00,prob_range={1.00000000e+00,1.00000000e+00},prob(percent)="100",prob+-sd="100+-0"]:6.620191e-03[&length_mean=6.76657499e-03,length_median=6.62019100e-03,length_95%HPD={3.13524700e-03,1.09019500e-02}],23[&prob=1.00000000e+00,prob_stddev=0.00000000e+00,prob_range={1.00000000e+00,1.00000000e+00},prob(percent)="100",prob+-sd="100+-0"]:1.640799e-03[&length_mean=1.83910916e-03,length_median=1.64079900e-03,length_95%HPD={2.64861600e-04,4.06027700e-03}],24[&prob=1.00000000e+00,prob_stddev=0.00000000e+00,prob_range={1.00000000e+00,1.00000000e+00},prob(percent)="100",prob+-sd="100+-0"]:2.217509e-03[&length_mean=2.42707724e-03,length_median=2.21750900e-03,length_95%HPD={5.25963200e-04,4.48168500e-03}])[&prob=6.98402130e-01,prob_stddev=1.41233046e-02,prob_range={6.88415446e-01,7.08388815e-01},prob(percent)="70",prob+-sd="70+-1"]:2.410332e-03[&length_mean=2.55816438e-03,length_median=2.41033200e-03,length_95%HPD={4.62966600e-04,5.19495600e-03}],22[&prob=1.00000000e+00,prob_stddev=0.00000000e+00,prob_range={1.00000000e+00,1.00000000e+00},prob(percent)="100",prob+-sd="100+-0"]:4.979160e-03[&length_mean=5.24642825e-03,length_median=4.97916000e-03,length_95%HPD={1.62094700e-03,9.53287500e-03}])[&prob=9.98002663e-01,prob_stddev=9.41553637e-04,prob_range={9.97336884e-01,9.98668442e-01},prob(percent)="100",prob+-sd="100+-0"]:3.156693e-03[&length_mean=3.44299891e-03,length_median=3.15669300e-03,length_95%HPD={6.74281000e-04,6.71030300e-03}],(25[&prob=1.00000000e+00,prob_stddev=0.00000000e+00,prob_range={1.00000000e+00,1.00000000e+00},prob(percent)="100",prob+-sd="100+-0"]:7.455402e-03[&length_mean=7.55987861e-03,length_median=7.45540200e-03,length_95%HPD={3.79978500e-03,1.13101100e-02}],((26[&prob=1.00000000e+00,prob_stddev=0.00000000e+00,prob_range={1.00000000e+00,1.00000000e+00},prob(percent)="100",prob+-sd="100+-0"]:1.839559e-03[&length_mean=2.06752170e-03,length_median=1.83955900e-03,length_95%HPD={1.30553600e-04,4.46184600e-03}],27[&prob=1.00000000e+00,prob_stddev=0.00000000e+00,prob_range={1.00000000e+00,1.00000000e+00},prob(percent)="100",prob+-sd="100+-0"]:7.330546e-03[&length_mean=7.37972702e-03,length_median=7.33054600e-03,length_95%HPD={3.22410500e-03,1.13818600e-02}])[&prob=1.00000000e+00,prob_stddev=0.00000000e+00,prob_range={1.00000000e+00,1.00000000e+00},prob(percent)="100",prob+-sd="100+-0"]:4.880857e-03[&length_mean=5.11822581e-03,length_median=4.88085700e-03,length_95%HPD={1.58964900e-03,8.66124500e-03}],28[&prob=1.00000000e+00,prob_stddev=0.00000000e+00,prob_range={1.00000000e+00,1.00000000e+00},prob(percent)="100",prob+-sd="100+-0"]:1.300559e-02[&length_mean=1.31400642e-02,length_median=1.30055900e-02,length_95%HPD={8.64309400e-03,1.82015900e-02}])[&prob=7.15712383e-01,prob_stddev=1.03570900e-02,prob_range={7.08388815e-01,7.23035952e-01},prob(percent)="72",prob+-sd="72+-1"]:1.848457e-03[&length_mean=2.17108222e-03,length_median=1.84845700e-03,length_95%HPD={7.92458600e-05,4.68362000e-03}])[&prob=9.26098535e-01,prob_stddev=5.93178791e-02,prob_range={8.84154461e-01,9.68042610e-01},prob(percent)="93",prob+-sd="93+-6"]:3.805858e-03[&length_mean=3.93327335e-03,length_median=3.80585800e-03,length_95%HPD={8.33731800e-04,7.07808400e-03}])[&prob=1.00000000e+00,prob_stddev=0.00000000e+00,prob_range={1.00000000e+00,1.00000000e+00},prob(percent)="100",prob+-sd="100+-0"]:1.863264e-02[&length_mean=1.88945325e-02,length_median=1.86326400e-02,length_95%HPD={1.28739600e-02,2.54390500e-02}],(((29[&prob=1.00000000e+00,prob_stddev=0.00000000e+00,prob_range={1.00000000e+00,1.00000000e+00},prob(percent)="100",prob+-sd="100+-0"]:2.909599e-03[&length_mean=3.08614759e-03,length_median=2.90959900e-03,length_95%HPD={6.57349100e-04,5.73564800e-03}],(30[&prob=1.00000000e+00,prob_stddev=0.00000000e+00,prob_range={1.00000000e+00,1.00000000e+00},prob(percent)="100",prob+-sd="100+-0"]:1.643008e-03[&length_mean=2.09572498e-03,length_median=1.64300800e-03,length_95%HPD={4.27096200e-07,5.59848800e-03}],31[&prob=1.00000000e+00,prob_stddev=0.00000000e+00,prob_range={1.00000000e+00,1.00000000e+00},prob(percent)="100",prob+-sd="100+-0"]:1.867285e-03[&length_mean=2.37216538e-03,length_median=1.86728500e-03,length_95%HPD={5.40447800e-05,5.73043700e-03}])[&prob=9.79360852e-01,prob_stddev=1.22401973e-02,prob_range={9.70705726e-01,9.88015979e-01},prob(percent)="98",prob+-sd="98+-1"]:3.636988e-03[&length_mean=3.91511414e-03,length_median=3.63698800e-03,length_95%HPD={4.15365300e-04,7.77245300e-03}])[&prob=1.00000000e+00,prob_stddev=0.00000000e+00,prob_range={1.00000000e+00,1.00000000e+00},prob(percent)="100",prob+-sd="100+-0"]:1.832143e-02[&length_mean=1.86356620e-02,length_median=1.83214300e-02,length_95%HPD={1.09827100e-02,2.60061100e-02}],(((32[&prob=1.00000000e+00,prob_stddev=0.00000000e+00,prob_range={1.00000000e+00,1.00000000e+00},prob(percent)="100",prob+-sd="100+-0"]:3.938315e-04[&length_mean=6.00708372e-04,length_median=3.93831500e-04,length_95%HPD={7.26956900e-07,1.89826200e-03}],33[&prob=1.00000000e+00,prob_stddev=0.00000000e+00,prob_range={1.00000000e+00,1.00000000e+00},prob(percent)="100",prob+-sd="100+-0"]:3.849166e-04[&length_mean=5.93426017e-04,length_median=3.84916600e-04,length_95%HPD={4.70693200e-07,1.85806300e-03}])[&prob=1.00000000e+00,prob_stddev=0.00000000e+00,prob_range={1.00000000e+00,1.00000000e+00},prob(percent)="100",prob+-sd="100+-0"]:1.634302e-03[&length_mean=1.78198335e-03,length_median=1.63430200e-03,length_95%HPD={1.80073200e-04,3.76119200e-03}],34[&prob=1.00000000e+00,prob_stddev=0.00000000e+00,prob_range={1.00000000e+00,1.00000000e+00},prob(percent)="100",prob+-sd="100+-0"]:4.767979e-04[&length_mean=6.08887574e-04,length_median=4.76797900e-04,length_95%HPD={9.20285000e-07,1.83495900e-03}])[&prob=9.68708389e-01,prob_stddev=9.41553637e-04,prob_range={9.68042610e-01,9.69374168e-01},prob(percent)="97",prob+-sd="97+-0"]:1.662403e-03[&length_mean=1.83276166e-03,length_median=1.66240300e-03,length_95%HPD={4.35914900e-05,4.07831400e-03}],35[&prob=1.00000000e+00,prob_stddev=0.00000000e+00,prob_range={1.00000000e+00,1.00000000e+00},prob(percent)="100",prob+-sd="100+-0"]:6.550837e-03[&length_mean=6.77787862e-03,length_median=6.55083700e-03,length_95%HPD={2.95020700e-03,1.09016300e-02}])[&prob=1.00000000e+00,prob_stddev=0.00000000e+00,prob_range={1.00000000e+00,1.00000000e+00},prob(percent)="100",prob+-sd="100+-0"]:8.696622e-03[&length_mean=8.95923617e-03,length_median=8.69662200e-03,length_95%HPD={4.35744500e-03,1.43751900e-02}])[&prob=1.00000000e+00,prob_stddev=0.00000000e+00,prob_range={1.00000000e+00,1.00000000e+00},prob(percent)="100",prob+-sd="100+-0"]:1.106999e-02[&length_mean=1.14158289e-02,length_median=1.10699900e-02,length_95%HPD={5.18593900e-03,1.71884100e-02}],((((56[&prob=1.00000000e+00,prob_stddev=0.00000000e+00,prob_range={1.00000000e+00,1.00000000e+00},prob(percent)="100",prob+-sd="100+-0"]:4.097620e-04[&length_mean=5.76091479e-04,length_median=4.09762000e-04,length_95%HPD={1.82349300e-06,1.70030100e-03}],57[&prob=1.00000000e+00,prob_stddev=0.00000000e+00,prob_range={1.00000000e+00,1.00000000e+00},prob(percent)="100",prob+-sd="100+-0"]:4.653112e-04[&length_mean=6.31143172e-04,length_median=4.65311200e-04,length_95%HPD={8.15459200e-07,1.87386700e-03}])[&prob=1.00000000e+00,prob_stddev=0.00000000e+00,prob_range={1.00000000e+00,1.00000000e+00},prob(percent)="100",prob+-sd="100+-0"]:4.168173e-03[&length_mean=4.34926848e-03,length_median=4.16817300e-03,length_95%HPD={1.64459900e-03,7.78187200e-03}],(58[&prob=1.00000000e+00,prob_stddev=0.00000000e+00,prob_range={1.00000000e+00,1.00000000e+00},prob(percent)="100",prob+-sd="100+-0"]:1.667897e-03[&length_mean=1.89503528e-03,length_median=1.66789700e-03,length_95%HPD={2.17993800e-04,4.05286200e-03}],59[&prob=1.00000000e+00,prob_stddev=0.00000000e+00,prob_range={1.00000000e+00,1.00000000e+00},prob(percent)="100",prob+-sd="100+-0"]:1.850355e-03[&length_mean=1.99882994e-03,length_median=1.85035500e-03,length_95%HPD={2.82862400e-04,4.00744700e-03}])[&prob=1.00000000e+00,prob_stddev=0.00000000e+00,prob_range={1.00000000e+00,1.00000000e+00},prob(percent)="100",prob+-sd="100+-0"]:3.244924e-03[&length_mean=3.40941353e-03,length_median=3.24492400e-03,length_95%HPD={1.10480200e-03,6.36991700e-03}])[&prob=1.00000000e+00,prob_stddev=0.00000000e+00,prob_range={1.00000000e+00,1.00000000e+00},prob(percent)="100",prob+-sd="100+-0"]:1.161869e-02[&length_mean=1.17129946e-02,length_median=1.16186900e-02,length_95%HPD={6.78861600e-03,1.68414000e-02}],(((60[&prob=1.00000000e+00,prob_stddev=0.00000000e+00,prob_range={1.00000000e+00,1.00000000e+00},prob(percent)="100",prob+-sd="100+-0"]:5.816857e-03[&length_mean=5.95778057e-03,length_median=5.81685700e-03,length_95%HPD={2.40373400e-03,9.90226400e-03}],(62[&prob=1.00000000e+00,prob_stddev=0.00000000e+00,prob_range={1.00000000e+00,1.00000000e+00},prob(percent)="100",prob+-sd="100+-0"]:2.653515e-03[&length_mean=2.90414585e-03,length_median=2.65351500e-03,length_95%HPD={3.40237100e-04,5.75432000e-03}],63[&prob=1.00000000e+00,prob_stddev=0.00000000e+00,prob_range={1.00000000e+00,1.00000000e+00},prob(percent)="100",prob+-sd="100+-0"]:6.677974e-03[&length_mean=7.06064370e-03,length_median=6.67797400e-03,length_95%HPD={3.22924500e-03,1.14185300e-02}])[&prob=1.00000000e+00,prob_stddev=0.00000000e+00,prob_range={1.00000000e+00,1.00000000e+00},prob(percent)="100",prob+-sd="100+-0"]:7.122681e-03[&length_mean=7.30467304e-03,length_median=7.12268100e-03,length_95%HPD={3.09604100e-03,1.19643800e-02}])[&prob=8.14913449e-01,prob_stddev=1.12986436e-02,prob_range={8.06924101e-01,8.22902796e-01},prob(percent)="81",prob+-sd="81+-1"]:2.092378e-03[&length_mean=2.39183933e-03,length_median=2.09237800e-03,length_95%HPD={9.66533400e-05,5.11722300e-03}],61[&prob=1.00000000e+00,prob_stddev=0.00000000e+00,prob_range={1.00000000e+00,1.00000000e+00},prob(percent)="100",prob+-sd="100+-0"]:3.753058e-03[&length_mean=4.02456361e-03,length_median=3.75305800e-03,length_95%HPD={1.19417400e-03,7.39057600e-03}])[&prob=1.00000000e+00,prob_stddev=0.00000000e+00,prob_range={1.00000000e+00,1.00000000e+00},prob(percent)="100",prob+-sd="100+-0"]:1.225769e-02[&length_mean=1.24835694e-02,length_median=1.22576900e-02,length_95%HPD={7.08675900e-03,1.93712700e-02}],(64[&prob=1.00000000e+00,prob_stddev=0.00000000e+00,prob_range={1.00000000e+00,1.00000000e+00},prob(percent)="100",prob+-sd="100+-0"]:1.440565e-02[&length_mean=1.47559975e-02,length_median=1.44056500e-02,length_95%HPD={9.33191600e-03,2.02740000e-02}],((65[&prob=1.00000000e+00,prob_stddev=0.00000000e+00,prob_range={1.00000000e+00,1.00000000e+00},prob(percent)="100",prob+-sd="100+-0"]:7.117417e-03[&length_mean=7.31352123e-03,length_median=7.11741700e-03,length_95%HPD={3.50089500e-03,1.19396100e-02}],66[&prob=1.00000000e+00,prob_stddev=0.00000000e+00,prob_range={1.00000000e+00,1.00000000e+00},prob(percent)="100",prob+-sd="100+-0"]:2.432268e-03[&length_mean=2.63079840e-03,length_median=2.43226800e-03,length_95%HPD={4.03656300e-04,5.72901800e-03}])[&prob=9.70039947e-01,prob_stddev=2.82466091e-03,prob_range={9.68042610e-01,9.72037284e-01},prob(percent)="97",prob+-sd="97+-0"]:2.894648e-03[&length_mean=3.13948340e-03,length_median=2.89464800e-03,length_95%HPD={5.39076900e-04,5.99621300e-03}],67[&prob=1.00000000e+00,prob_stddev=0.00000000e+00,prob_range={1.00000000e+00,1.00000000e+00},prob(percent)="100",prob+-sd="100+-0"]:5.258113e-03[&length_mean=5.36769177e-03,length_median=5.25811300e-03,length_95%HPD={2.09761500e-03,8.71460600e-03}])[&prob=1.00000000e+00,prob_stddev=0.00000000e+00,prob_range={1.00000000e+00,1.00000000e+00},prob(percent)="100",prob+-sd="100+-0"]:1.068629e-02[&length_mean=1.08571441e-02,length_median=1.06862900e-02,length_95%HPD={6.05399800e-03,1.66499800e-02}])[&prob=1.00000000e+00,prob_stddev=0.00000000e+00,prob_range={1.00000000e+00,1.00000000e+00},prob(percent)="100",prob+-sd="100+-0"]:1.126853e-02[&length_mean=1.13146829e-02,length_median=1.12685300e-02,length_95%HPD={5.17955800e-03,1.83811400e-02}])[&prob=7.10386152e-01,prob_stddev=2.35388409e-02,prob_range={6.93741678e-01,7.27030626e-01},prob(percent)="71",prob+-sd="71+-2"]:4.761443e-03[&length_mean=5.15775539e-03,length_median=4.76144300e-03,length_95%HPD={5.50243100e-04,1.05198800e-02}])[&prob=1.00000000e+00,prob_stddev=0.00000000e+00,prob_range={1.00000000e+00,1.00000000e+00},prob(percent)="100",prob+-sd="100+-0"]:1.526303e-02[&length_mean=1.54591468e-02,length_median=1.52630300e-02,length_95%HPD={8.66747900e-03,2.20337900e-02}],((68[&prob=1.00000000e+00,prob_stddev=0.00000000e+00,prob_range={1.00000000e+00,1.00000000e+00},prob(percent)="100",prob+-sd="100+-0"]:3.450924e-04[&length_mean=5.09564749e-04,length_median=3.45092400e-04,length_95%HPD={7.11825800e-07,1.48563200e-03}],69[&prob=1.00000000e+00,prob_stddev=0.00000000e+00,prob_range={1.00000000e+00,1.00000000e+00},prob(percent)="100",prob+-sd="100+-0"]:4.082137e-04[&length_mean=5.88642652e-04,length_median=4.08213700e-04,length_95%HPD={1.77646800e-06,1.77720400e-03}])[&prob=1.00000000e+00,prob_stddev=0.00000000e+00,prob_range={1.00000000e+00,1.00000000e+00},prob(percent)="100",prob+-sd="100+-0"]:3.945044e-03[&length_mean=4.11517367e-03,length_median=3.94504400e-03,length_95%HPD={1.35255400e-03,7.12733000e-03}],((70[&prob=1.00000000e+00,prob_stddev=0.00000000e+00,prob_range={1.00000000e+00,1.00000000e+00},prob(percent)="100",prob+-sd="100+-0"]:4.146576e-04[&length_mean=6.11366461e-04,length_median=4.14657600e-04,length_95%HPD={4.44203700e-07,1.84381400e-03}],71[&prob=1.00000000e+00,prob_stddev=0.00000000e+00,prob_range={1.00000000e+00,1.00000000e+00},prob(percent)="100",prob+-sd="100+-0"]:4.505771e-04[&length_mean=6.10010144e-04,length_median=4.50577100e-04,length_95%HPD={2.70012700e-08,1.79942000e-03}])[&prob=9.94673768e-01,prob_stddev=1.88310727e-03,prob_range={9.93342210e-01,9.96005326e-01},prob(percent)="99",prob+-sd="99+-0"]:1.062227e-03[&length_mean=1.21154820e-03,length_median=1.06222700e-03,length_95%HPD={1.12399500e-05,2.64491500e-03}],72[&prob=1.00000000e+00,prob_stddev=0.00000000e+00,prob_range={1.00000000e+00,1.00000000e+00},prob(percent)="100",prob+-sd="100+-0"]:4.124121e-04[&length_mean=5.72634081e-04,length_median=4.12412100e-04,length_95%HPD={1.62748400e-07,1.65386000e-03}])[&prob=1.00000000e+00,prob_stddev=0.00000000e+00,prob_range={1.00000000e+00,1.00000000e+00},prob(percent)="100",prob+-sd="100+-0"]:5.204966e-03[&length_mean=5.40169368e-03,length_median=5.20496600e-03,length_95%HPD={2.05010700e-03,9.13242000e-03}])[&prob=1.00000000e+00,prob_stddev=0.00000000e+00,prob_range={1.00000000e+00,1.00000000e+00},prob(percent)="100",prob+-sd="100+-0"]:3.633718e-02[&length_mean=3.65070476e-02,length_median=3.63371800e-02,length_95%HPD={2.65895200e-02,4.56493000e-02}])[&prob=1.00000000e+00,prob_stddev=0.00000000e+00,prob_range={1.00000000e+00,1.00000000e+00},prob(percent)="100",prob+-sd="100+-0"]:8.647109e-03[&length_mean=8.92829359e-03,length_median=8.64710900e-03,length_95%HPD={3.56558600e-03,1.41719300e-02}])[&prob=9.80026631e-01,prob_stddev=1.88310727e-03,prob_range={9.78695073e-01,9.81358189e-01},prob(percent)="98",prob+-sd="98+-0"]:4.393582e-03[&length_mean=4.57272444e-03,length_median=4.39358200e-03,length_95%HPD={6.29276700e-04,8.86685800e-03}])[&prob=6.71105193e-01,prob_stddev=7.53242909e-02,prob_range={6.17842876e-01,7.24367510e-01},prob(percent)="67",prob+-sd="67+-8"]:2.936600e-03[&length_mean=3.17223724e-03,length_median=2.93660000e-03,length_95%HPD={8.14182400e-04,6.54033700e-03}],(((((36[&prob=1.00000000e+00,prob_stddev=0.00000000e+00,prob_range={1.00000000e+00,1.00000000e+00},prob(percent)="100",prob+-sd="100+-0"]:2.792386e-03[&length_mean=2.98917600e-03,length_median=2.79238600e-03,length_95%HPD={6.61146400e-04,5.51526000e-03}],37[&prob=1.00000000e+00,prob_stddev=0.00000000e+00,prob_range={1.00000000e+00,1.00000000e+00},prob(percent)="100",prob+-sd="100+-0"]:1.105138e-03[&length_mean=1.28594019e-03,length_median=1.10513800e-03,length_95%HPD={5.53851700e-05,3.08531600e-03}])[&prob=9.94007989e-01,prob_stddev=9.41553637e-04,prob_range={9.93342210e-01,9.94673768e-01},prob(percent)="99",prob+-sd="99+-0"]:2.318093e-03[&length_mean=2.46808470e-03,length_median=2.31809300e-03,length_95%HPD={5.91474800e-04,4.66644900e-03}],38[&prob=1.00000000e+00,prob_stddev=0.00000000e+00,prob_range={1.00000000e+00,1.00000000e+00},prob(percent)="100",prob+-sd="100+-0"]:4.948308e-04[&length_mean=7.16068980e-04,length_median=4.94830800e-04,length_95%HPD={8.26430600e-08,2.10965600e-03}])[&prob=1.00000000e+00,prob_stddev=0.00000000e+00,prob_range={1.00000000e+00,1.00000000e+00},prob(percent)="100",prob+-sd="100+-0"]:6.786758e-03[&length_mean=7.02668150e-03,length_median=6.78675800e-03,length_95%HPD={3.52297500e-03,1.11418000e-02}],39[&prob=1.00000000e+00,prob_stddev=0.00000000e+00,prob_range={1.00000000e+00,1.00000000e+00},prob(percent)="100",prob+-sd="100+-0"]:2.146922e-03[&length_mean=2.36131954e-03,length_median=2.14692200e-03,length_95%HPD={3.37929100e-04,4.60804000e-03}])[&prob=1.00000000e+00,prob_stddev=0.00000000e+00,prob_range={1.00000000e+00,1.00000000e+00},prob(percent)="100",prob+-sd="100+-0"]:1.563427e-02[&length_mean=1.58814239e-02,length_median=1.56342700e-02,length_95%HPD={1.01825000e-02,2.24043100e-02}],(((48[&prob=1.00000000e+00,prob_stddev=0.00000000e+00,prob_range={1.00000000e+00,1.00000000e+00},prob(percent)="100",prob+-sd="100+-0"]:4.748378e-03[&length_mean=4.91762466e-03,length_median=4.74837800e-03,length_95%HPD={2.13238800e-03,8.18249900e-03}],(49[&prob=1.00000000e+00,prob_stddev=0.00000000e+00,prob_range={1.00000000e+00,1.00000000e+00},prob(percent)="100",prob+-sd="100+-0"]:1.471739e-03[&length_mean=1.64545159e-03,length_median=1.47173900e-03,length_95%HPD={6.24607100e-05,3.69287600e-03}],50[&prob=1.00000000e+00,prob_stddev=0.00000000e+00,prob_range={1.00000000e+00,1.00000000e+00},prob(percent)="100",prob+-sd="100+-0"]:2.139052e-03[&length_mean=2.39491424e-03,length_median=2.13905200e-03,length_95%HPD={4.01601700e-04,4.81168200e-03}])[&prob=8.18242344e-01,prob_stddev=9.41553637e-04,prob_range={8.17576565e-01,8.18908123e-01},prob(percent)="82",prob+-sd="82+-0"]:1.445416e-03[&length_mean=1.69256822e-03,length_median=1.44541600e-03,length_95%HPD={4.19803900e-05,3.82943400e-03}])[&prob=9.02796272e-01,prob_stddev=1.31817509e-02,prob_range={8.93475366e-01,9.12117177e-01},prob(percent)="90",prob+-sd="90+-1"]:1.474955e-03[&length_mean=1.65312628e-03,length_median=1.47495500e-03,length_95%HPD={7.64792200e-05,3.42424400e-03}],51[&prob=1.00000000e+00,prob_stddev=0.00000000e+00,prob_range={1.00000000e+00,1.00000000e+00},prob(percent)="100",prob+-sd="100+-0"]:2.105370e-03[&length_mean=2.26041419e-03,length_median=2.10537000e-03,length_95%HPD={3.23926700e-04,4.19548700e-03}])[&prob=1.00000000e+00,prob_stddev=0.00000000e+00,prob_range={1.00000000e+00,1.00000000e+00},prob(percent)="100",prob+-sd="100+-0"]:6.962786e-03[&length_mean=7.04470329e-03,length_median=6.96278600e-03,length_95%HPD={3.29492200e-03,1.07811700e-02}],((52[&prob=1.00000000e+00,prob_stddev=0.00000000e+00,prob_range={1.00000000e+00,1.00000000e+00},prob(percent)="100",prob+-sd="100+-0"]:3.501342e-04[&length_mean=4.97025626e-04,length_median=3.50134200e-04,length_95%HPD={3.19799900e-07,1.49166500e-03}],53[&prob=1.00000000e+00,prob_stddev=0.00000000e+00,prob_range={1.00000000e+00,1.00000000e+00},prob(percent)="100",prob+-sd="100+-0"]:4.207675e-04[&length_mean=5.98819454e-04,length_median=4.20767500e-04,length_95%HPD={3.46639500e-07,1.81794400e-03}])[&prob=1.00000000e+00,prob_stddev=0.00000000e+00,prob_range={1.00000000e+00,1.00000000e+00},prob(percent)="100",prob+-sd="100+-0"]:5.538011e-03[&length_mean=5.69024104e-03,length_median=5.53801100e-03,length_95%HPD={2.57795300e-03,9.42820500e-03}],(54[&prob=1.00000000e+00,prob_stddev=0.00000000e+00,prob_range={1.00000000e+00,1.00000000e+00},prob(percent)="100",prob+-sd="100+-0"]:6.744644e-04[&length_mean=9.10825362e-04,length_median=6.74464400e-04,length_95%HPD={2.66179300e-06,2.50601000e-03}],55[&prob=1.00000000e+00,prob_stddev=0.00000000e+00,prob_range={1.00000000e+00,1.00000000e+00},prob(percent)="100",prob+-sd="100+-0"]:5.501255e-04[&length_mean=7.49383348e-04,length_median=5.50125500e-04,length_95%HPD={9.89254500e-08,2.22382600e-03}])[&prob=1.00000000e+00,prob_stddev=0.00000000e+00,prob_range={1.00000000e+00,1.00000000e+00},prob(percent)="100",prob+-sd="100+-0"]:2.782158e-03[&length_mean=2.92006056e-03,length_median=2.78215800e-03,length_95%HPD={6.71911300e-04,5.44042700e-03}])[&prob=1.00000000e+00,prob_stddev=0.00000000e+00,prob_range={1.00000000e+00,1.00000000e+00},prob(percent)="100",prob+-sd="100+-0"]:4.411055e-03[&length_mean=4.56929473e-03,length_median=4.41105500e-03,length_95%HPD={1.71810400e-03,7.78757800e-03}])[&prob=1.00000000e+00,prob_stddev=0.00000000e+00,prob_range={1.00000000e+00,1.00000000e+00},prob(percent)="100",prob+-sd="100+-0"]:1.411171e-02[&length_mean=1.44102770e-02,length_median=1.41117100e-02,length_95%HPD={9.26014300e-03,2.11577200e-02}])[&prob=9.57390146e-01,prob_stddev=1.69479655e-02,prob_range={9.45406125e-01,9.69374168e-01},prob(percent)="96",prob+-sd="96+-2"]:3.756205e-03[&length_mean=4.01808579e-03,length_median=3.75620500e-03,length_95%HPD={6.45221900e-04,7.59276600e-03}],(73[&prob=1.00000000e+00,prob_stddev=0.00000000e+00,prob_range={1.00000000e+00,1.00000000e+00},prob(percent)="100",prob+-sd="100+-0"]:5.182311e-03[&length_mean=5.34305661e-03,length_median=5.18231100e-03,length_95%HPD={1.71780700e-03,9.12994700e-03}],74[&prob=1.00000000e+00,prob_stddev=0.00000000e+00,prob_range={1.00000000e+00,1.00000000e+00},prob(percent)="100",prob+-sd="100+-0"]:1.150673e-02[&length_mean=1.18381513e-02,length_median=1.15067300e-02,length_95%HPD={6.21171700e-03,1.76636500e-02}])[&prob=1.00000000e+00,prob_stddev=0.00000000e+00,prob_range={1.00000000e+00,1.00000000e+00},prob(percent)="100",prob+-sd="100+-0"]:1.949259e-02[&length_mean=1.96006091e-02,length_median=1.94925900e-02,length_95%HPD={1.26662400e-02,2.76309200e-02}])[&prob=9.14114514e-01,prob_stddev=1.03570900e-02,prob_range={9.06790945e-01,9.21438083e-01},prob(percent)="91",prob+-sd="91+-1"]:2.862112e-03[&length_mean=3.09143059e-03,length_median=2.86211200e-03,length_95%HPD={2.39799800e-04,6.12227700e-03}],((((40[&prob=1.00000000e+00,prob_stddev=0.00000000e+00,prob_range={1.00000000e+00,1.00000000e+00},prob(percent)="100",prob+-sd="100+-0"]:4.899136e-03[&length_mean=5.17156591e-03,length_median=4.89913600e-03,length_95%HPD={2.03129100e-03,8.46792500e-03}],41[&prob=1.00000000e+00,prob_stddev=0.00000000e+00,prob_range={1.00000000e+00,1.00000000e+00},prob(percent)="100",prob+-sd="100+-0"]:2.630568e-03[&length_mean=2.86307768e-03,length_median=2.63056800e-03,length_95%HPD={6.18247300e-04,5.95489900e-03}])[&prob=1.00000000e+00,prob_stddev=0.00000000e+00,prob_range={1.00000000e+00,1.00000000e+00},prob(percent)="100",prob+-sd="100+-0"]:7.352814e-03[&length_mean=7.53071681e-03,length_median=7.35281400e-03,length_95%HPD={3.46949500e-03,1.15983100e-02}],42[&prob=1.00000000e+00,prob_stddev=0.00000000e+00,prob_range={1.00000000e+00,1.00000000e+00},prob(percent)="100",prob+-sd="100+-0"]:6.234689e-03[&length_mean=6.40866057e-03,length_median=6.23468900e-03,length_95%HPD={2.73665700e-03,1.03977300e-02}])[&prob=9.99334221e-01,prob_stddev=9.41553637e-04,prob_range={9.98668442e-01,1.00000000e+00},prob(percent)="100",prob+-sd="100+-0"]:4.484204e-03[&length_mean=4.70558960e-03,length_median=4.48420400e-03,length_95%HPD={1.31218800e-03,8.21967500e-03}],43[&prob=1.00000000e+00,prob_stddev=0.00000000e+00,prob_range={1.00000000e+00,1.00000000e+00},prob(percent)="100",prob+-sd="100+-0"]:1.090426e-02[&length_mean=1.12437994e-02,length_median=1.09042600e-02,length_95%HPD={6.21366100e-03,1.69066500e-02}])[&prob=1.00000000e+00,prob_stddev=0.00000000e+00,prob_range={1.00000000e+00,1.00000000e+00},prob(percent)="100",prob+-sd="100+-0"]:9.275177e-03[&length_mean=9.50584237e-03,length_median=9.27517700e-03,length_95%HPD={4.49480100e-03,1.45783600e-02}],((44[&prob=1.00000000e+00,prob_stddev=0.00000000e+00,prob_range={1.00000000e+00,1.00000000e+00},prob(percent)="100",prob+-sd="100+-0"]:4.167659e-04[&length_mean=6.05234651e-04,length_median=4.16765900e-04,length_95%HPD={1.88799900e-07,1.93021600e-03}],45[&prob=1.00000000e+00,prob_stddev=0.00000000e+00,prob_range={1.00000000e+00,1.00000000e+00},prob(percent)="100",prob+-sd="100+-0"]:4.696070e-03[&length_mean=4.87389650e-03,length_median=4.69607000e-03,length_95%HPD={1.86288200e-03,8.35267100e-03}])[&prob=9.85352863e-01,prob_stddev=0.00000000e+00,prob_range={9.85352863e-01,9.85352863e-01},prob(percent)="99",prob+-sd="99+-0"]:2.774060e-03[&length_mean=2.98669465e-03,length_median=2.77406000e-03,length_95%HPD={2.31724200e-04,5.81191200e-03}],(46[&prob=1.00000000e+00,prob_stddev=0.00000000e+00,prob_range={1.00000000e+00,1.00000000e+00},prob(percent)="100",prob+-sd="100+-0"]:2.750098e-03[&length_mean=2.98553086e-03,length_median=2.75009800e-03,length_95%HPD={7.82349700e-04,5.77932800e-03}],47[&prob=1.00000000e+00,prob_stddev=0.00000000e+00,prob_range={1.00000000e+00,1.00000000e+00},prob(percent)="100",prob+-sd="100+-0"]:2.289226e-03[&length_mean=2.44316621e-03,length_median=2.28922600e-03,length_95%HPD={2.76191300e-04,4.71442700e-03}])[&prob=1.00000000e+00,prob_stddev=0.00000000e+00,prob_range={1.00000000e+00,1.00000000e+00},prob(percent)="100",prob+-sd="100+-0"]:4.126267e-03[&length_mean=4.30219622e-03,length_median=4.12626700e-03,length_95%HPD={9.15282000e-04,7.79202700e-03}])[&prob=1.00000000e+00,prob_stddev=0.00000000e+00,prob_range={1.00000000e+00,1.00000000e+00},prob(percent)="100",prob+-sd="100+-0"]:6.951201e-03[&length_mean=7.25250937e-03,length_median=6.95120100e-03,length_95%HPD={3.26358300e-03,1.14759300e-02}])[&prob=1.00000000e+00,prob_stddev=0.00000000e+00,prob_range={1.00000000e+00,1.00000000e+00},prob(percent)="100",prob+-sd="100+-0"]:1.986796e-02[&length_mean=2.01236916e-02,length_median=1.98679600e-02,length_95%HPD={1.34648300e-02,2.74049900e-02}])[&prob=5.61917443e-01,prob_stddev=1.26168187e-01,prob_range={4.72703063e-01,6.51131824e-01},prob(percent)="56",prob+-sd="56+-13"]:4.544454e-03[&length_mean=4.75233456e-03,length_median=4.54445400e-03,length_95%HPD={8.53189800e-04,8.71821400e-03}],(((((75[&prob=1.00000000e+00,prob_stddev=0.00000000e+00,prob_range={1.00000000e+00,1.00000000e+00},prob(percent)="100",prob+-sd="100+-0"]:6.335821e-04[&length_mean=8.20449393e-04,length_median=6.33582100e-04,length_95%HPD={1.65547300e-06,2.21494600e-03}],76[&prob=1.00000000e+00,prob_stddev=0.00000000e+00,prob_range={1.00000000e+00,1.00000000e+00},prob(percent)="100",prob+-sd="100+-0"]:4.168042e-04[&length_mean=6.10035121e-04,length_median=4.16804200e-04,length_95%HPD={6.94011900e-08,1.85470900e-03}],77[&prob=1.00000000e+00,prob_stddev=0.00000000e+00,prob_range={1.00000000e+00,1.00000000e+00},prob(percent)="100",prob+-sd="100+-0"]:3.904687e-04[&length_mean=5.90512531e-04,length_median=3.90468700e-04,length_95%HPD={2.37597100e-06,1.71314200e-03}])[&prob=5.72569907e-01,prob_stddev=2.44803946e-02,prob_range={5.55259654e-01,5.89880160e-01},prob(percent)="57",prob+-sd="57+-2"]:1.054014e-03[&length_mean=1.27286612e-03,length_median=1.05401400e-03,length_95%HPD={1.29958700e-05,2.84563500e-03}],78[&prob=1.00000000e+00,prob_stddev=0.00000000e+00,prob_range={1.00000000e+00,1.00000000e+00},prob(percent)="100",prob+-sd="100+-0"]:1.799644e-03[&length_mean=1.98015116e-03,length_median=1.79964400e-03,length_95%HPD={1.35269400e-04,3.90454700e-03}])[&prob=1.00000000e+00,prob_stddev=0.00000000e+00,prob_range={1.00000000e+00,1.00000000e+00},prob(percent)="100",prob+-sd="100+-0"]:1.629977e-02[&length_mean=1.66629504e-02,length_median=1.62997700e-02,length_95%HPD={1.07485800e-02,2.28609500e-02}],(79[&prob=1.00000000e+00,prob_stddev=0.00000000e+00,prob_range={1.00000000e+00,1.00000000e+00},prob(percent)="100",prob+-sd="100+-0"]:1.597802e-03[&length_mean=1.79249748e-03,length_median=1.59780200e-03,length_95%HPD={2.60732300e-04,3.82393600e-03}],((80[&prob=1.00000000e+00,prob_stddev=0.00000000e+00,prob_range={1.00000000e+00,1.00000000e+00},prob(percent)="100",prob+-sd="100+-0"]:1.566317e-03[&length_mean=1.77262136e-03,length_median=1.56631700e-03,length_95%HPD={1.88394300e-04,3.80933000e-03}],81[&prob=1.00000000e+00,prob_stddev=0.00000000e+00,prob_range={1.00000000e+00,1.00000000e+00},prob(percent)="100",prob+-sd="100+-0"]:3.838861e-04[&length_mean=5.79736994e-04,length_median=3.83886100e-04,length_95%HPD={1.32770700e-06,1.84221700e-03}])[&prob=8.89480692e-01,prob_stddev=9.41553637e-03,prob_range={8.82822903e-01,8.96138482e-01},prob(percent)="89",prob+-sd="89+-1"]:1.040013e-03[&length_mean=1.29702837e-03,length_median=1.04001300e-03,length_95%HPD={2.79700100e-06,3.17894700e-03}],82[&prob=1.00000000e+00,prob_stddev=0.00000000e+00,prob_range={1.00000000e+00,1.00000000e+00},prob(percent)="100",prob+-sd="100+-0"]:3.623318e-03[&length_mean=3.86431400e-03,length_median=3.62331800e-03,length_95%HPD={1.40280900e-03,6.96188900e-03}])[&prob=9.46737683e-01,prob_stddev=0.00000000e+00,prob_range={9.46737683e-01,9.46737683e-01},prob(percent)="95",prob+-sd="95+-0"]:1.221908e-03[&length_mean=1.41312633e-03,length_median=1.22190800e-03,length_95%HPD={5.17896600e-05,3.33561700e-03}])[&prob=1.00000000e+00,prob_stddev=0.00000000e+00,prob_range={1.00000000e+00,1.00000000e+00},prob(percent)="100",prob+-sd="100+-0"]:6.220615e-03[&length_mean=6.43751614e-03,length_median=6.22061500e-03,length_95%HPD={2.75017600e-03,1.05073100e-02}])[&prob=1.00000000e+00,prob_stddev=0.00000000e+00,prob_range={1.00000000e+00,1.00000000e+00},prob(percent)="100",prob+-sd="100+-0"]:1.233300e-02[&length_mean=1.25511812e-02,length_median=1.23330000e-02,length_95%HPD={7.28866400e-03,1.82370800e-02}],((83[&prob=1.00000000e+00,prob_stddev=0.00000000e+00,prob_range={1.00000000e+00,1.00000000e+00},prob(percent)="100",prob+-sd="100+-0"]:7.728165e-04[&length_mean=9.69979374e-04,length_median=7.72816500e-04,length_95%HPD={5.59218700e-06,2.43611600e-03}],84[&prob=1.00000000e+00,prob_stddev=0.00000000e+00,prob_range={1.00000000e+00,1.00000000e+00},prob(percent)="100",prob+-sd="100+-0"]:7.043165e-04[&length_mean=9.17106693e-04,length_median=7.04316500e-04,length_95%HPD={4.69083000e-06,2.50456700e-03}])[&prob=8.08255659e-01,prob_stddev=1.69479655e-02,prob_range={7.96271638e-01,8.20239680e-01},prob(percent)="81",prob+-sd="81+-2"]:1.745049e-03[&length_mean=1.91691542e-03,length_median=1.74504900e-03,length_95%HPD={1.95415300e-04,4.06064200e-03}],85[&prob=1.00000000e+00,prob_stddev=0.00000000e+00,prob_range={1.00000000e+00,1.00000000e+00},prob(percent)="100",prob+-sd="100+-0"]:1.458386e-02[&length_mean=1.47084904e-02,length_median=1.45838600e-02,length_95%HPD={9.24706100e-03,2.02353600e-02}])[&prob=1.00000000e+00,prob_stddev=0.00000000e+00,prob_range={1.00000000e+00,1.00000000e+00},prob(percent)="100",prob+-sd="100+-0"]:8.186238e-03[&length_mean=8.29084158e-03,length_median=8.18623800e-03,length_95%HPD={3.48641800e-03,1.31735600e-02}])[&prob=1.00000000e+00,prob_stddev=0.00000000e+00,prob_range={1.00000000e+00,1.00000000e+00},prob(percent)="100",prob+-sd="100+-0"]:4.312096e-02[&length_mean=4.33592836e-02,length_median=4.31209600e-02,length_95%HPD={3.03362800e-02,5.71128700e-02}],((((86[&prob=1.00000000e+00,prob_stddev=0.00000000e+00,prob_range={1.00000000e+00,1.00000000e+00},prob(percent)="100",prob+-sd="100+-0"]:6.802772e-03[&length_mean=6.90795024e-03,length_median=6.80277200e-03,length_95%HPD={2.91996600e-03,1.11923400e-02}],(87[&prob=1.00000000e+00,prob_stddev=0.00000000e+00,prob_range={1.00000000e+00,1.00000000e+00},prob(percent)="100",prob+-sd="100+-0"]:6.823516e-03[&length_mean=6.94283165e-03,length_median=6.82351600e-03,length_95%HPD={3.31396400e-03,1.04246300e-02}],88[&prob=1.00000000e+00,prob_stddev=0.00000000e+00,prob_range={1.00000000e+00,1.00000000e+00},prob(percent)="100",prob+-sd="100+-0"]:1.318072e-02[&length_mean=1.34407570e-02,length_median=1.31807200e-02,length_95%HPD={8.79098200e-03,1.87381600e-02}])[&prob=7.54327563e-01,prob_stddev=4.70776818e-03,prob_range={7.50998668e-01,7.57656458e-01},prob(percent)="75",prob+-sd="75+-0"]:2.473325e-03[&length_mean=2.71966377e-03,length_median=2.47332500e-03,length_95%HPD={1.93787600e-04,5.57141000e-03}])[&prob=1.00000000e+00,prob_stddev=0.00000000e+00,prob_range={1.00000000e+00,1.00000000e+00},prob(percent)="100",prob+-sd="100+-0"]:6.561580e-02[&length_mean=6.62542440e-02,length_median=6.56158000e-02,length_95%HPD={5.03581800e-02,8.26190400e-02}],((89[&prob=1.00000000e+00,prob_stddev=0.00000000e+00,prob_range={1.00000000e+00,1.00000000e+00},prob(percent)="100",prob+-sd="100+-0"]:6.884991e-03[&length_mean=7.03273636e-03,length_median=6.88499100e-03,length_95%HPD={3.73212800e-03,1.11341400e-02}],91[&prob=1.00000000e+00,prob_stddev=0.00000000e+00,prob_range={1.00000000e+00,1.00000000e+00},prob(percent)="100",prob+-sd="100+-0"]:3.773160e-03[&length_mean=3.82966594e-03,length_median=3.77316000e-03,length_95%HPD={8.82692000e-04,6.63736400e-03}])[&prob=7.87616511e-01,prob_stddev=4.70776818e-03,prob_range={7.84287617e-01,7.90945406e-01},prob(percent)="79",prob+-sd="79+-0"]:2.931443e-03[&length_mean=3.16061121e-03,length_median=2.93144300e-03,length_95%HPD={4.63035900e-04,6.36004700e-03}],90[&prob=1.00000000e+00,prob_stddev=0.00000000e+00,prob_range={1.00000000e+00,1.00000000e+00},prob(percent)="100",prob+-sd="100+-0"]:2.369211e-03[&length_mean=2.75481968e-03,length_median=2.36921100e-03,length_95%HPD={1.31642600e-04,6.49637100e-03}])[&prob=1.00000000e+00,prob_stddev=0.00000000e+00,prob_range={1.00000000e+00,1.00000000e+00},prob(percent)="100",prob+-sd="100+-0"]:4.304873e-02[&length_mean=4.35517195e-02,length_median=4.30487300e-02,length_95%HPD={3.06005200e-02,6.01842600e-02}])[&prob=1.00000000e+00,prob_stddev=0.00000000e+00,prob_range={1.00000000e+00,1.00000000e+00},prob(percent)="100",prob+-sd="100+-0"]:2.456693e-02[&length_mean=2.50811338e-02,length_median=2.45669300e-02,length_95%HPD={1.27717000e-02,3.97380500e-02}],(92[&prob=1.00000000e+00,prob_stddev=0.00000000e+00,prob_range={1.00000000e+00,1.00000000e+00},prob(percent)="100",prob+-sd="100+-0"]:3.215085e-03[&length_mean=3.40636179e-03,length_median=3.21508500e-03,length_95%HPD={1.04546500e-05,6.95891800e-03}],(93[&prob=1.00000000e+00,prob_stddev=0.00000000e+00,prob_range={1.00000000e+00,1.00000000e+00},prob(percent)="100",prob+-sd="100+-0"]:2.578877e-03[&length_mean=2.82715756e-03,length_median=2.57887700e-03,length_95%HPD={4.76009000e-04,5.62930400e-03}],94[&prob=1.00000000e+00,prob_stddev=0.00000000e+00,prob_range={1.00000000e+00,1.00000000e+00},prob(percent)="100",prob+-sd="100+-0"]:1.111549e-02[&length_mean=1.13781803e-02,length_median=1.11154900e-02,length_95%HPD={6.76997000e-03,1.72897800e-02}])[&prob=9.99334221e-01,prob_stddev=9.41553637e-04,prob_range={9.98668442e-01,1.00000000e+00},prob(percent)="100",prob+-sd="100+-0"]:6.237311e-03[&length_mean=6.53716199e-03,length_median=6.23731100e-03,length_95%HPD={1.93945400e-03,1.09857100e-02}])[&prob=1.00000000e+00,prob_stddev=0.00000000e+00,prob_range={1.00000000e+00,1.00000000e+00},prob(percent)="100",prob+-sd="100+-0"]:6.929115e-02[&length_mean=6.99105584e-02,length_median=6.92911500e-02,length_95%HPD={5.21800000e-02,8.98748600e-02}])[&prob=1.00000000e+00,prob_stddev=0.00000000e+00,prob_range={1.00000000e+00,1.00000000e+00},prob(percent)="100",prob+-sd="100+-0"]:2.744002e-02[&length_mean=2.83737160e-02,length_median=2.74400200e-02,length_95%HPD={1.44674800e-02,4.50159900e-02}],(95[&prob=1.00000000e+00,prob_stddev=0.00000000e+00,prob_range={1.00000000e+00,1.00000000e+00},prob(percent)="100",prob+-sd="100+-0"]:5.534571e-03[&length_mean=5.61542242e-03,length_median=5.53457100e-03,length_95%HPD={1.83535800e-03,9.74106000e-03}],(96[&prob=1.00000000e+00,prob_stddev=0.00000000e+00,prob_range={1.00000000e+00,1.00000000e+00},prob(percent)="100",prob+-sd="100+-0"]:1.475489e-03[&length_mean=1.60646943e-03,length_median=1.47548900e-03,length_95%HPD={3.64400100e-06,3.50856400e-03}],97[&prob=1.00000000e+00,prob_stddev=0.00000000e+00,prob_range={1.00000000e+00,1.00000000e+00},prob(percent)="100",prob+-sd="100+-0"]:1.280948e-02[&length_mean=1.27060797e-02,length_median=1.28094800e-02,length_95%HPD={7.87775800e-03,1.70986600e-02}])[&prob=6.65778961e-01,prob_stddev=5.83763255e-02,prob_range={6.24500666e-01,7.07057257e-01},prob(percent)="67",prob+-sd="67+-6"]:2.967129e-03[&length_mean=3.20946731e-03,length_median=2.96712900e-03,length_95%HPD={1.35218400e-04,6.08597500e-03}])[&prob=1.00000000e+00,prob_stddev=0.00000000e+00,prob_range={1.00000000e+00,1.00000000e+00},prob(percent)="100",prob+-sd="100+-0"]:1.881166e-01[&length_mean=1.89123612e-01,length_median=1.88116600e-01,length_95%HPD={1.58109900e-01,2.22669700e-01}])[&prob=1.00000000e+00,prob_stddev=0.00000000e+00,prob_range={1.00000000e+00,1.00000000e+00},prob(percent)="100",prob+-sd="100+-0"]:7.657419e-02[&length_mean=7.69641239e-02,length_median=7.65741900e-02,length_95%HPD={5.74731800e-02,9.67678000e-02}])[&prob=1.00000000e+00,prob_stddev=0.00000000e+00,prob_range={1.00000000e+00,1.00000000e+00},prob(percent)="100",prob+-sd="100+-0"]:1.767362e-02[&length_mean=1.78786532e-02,length_median=1.76736200e-02,length_95%HPD={9.41848800e-03,2.75562800e-02}])[&prob=1.00000000e+00,prob_stddev=0.00000000e+00,prob_range={1.00000000e+00,1.00000000e+00},prob(percent)="100",prob+-sd="100+-0"]:8.282693e-03[&length_mean=8.39149712e-03,length_median=8.28269300e-03,length_95%HPD={3.29781500e-03,1.42076200e-02}])[&prob=1.00000000e+00,prob_stddev=0.00000000e+00,prob_range={1.00000000e+00,1.00000000e+00},prob(percent)="100",prob+-sd="100+-0"]:7.975574e-03[&length_mean=8.19210510e-03,length_median=7.97557400e-03,length_95%HPD={4.04262300e-03,1.28065500e-02}])[&prob=1.00000000e+00,prob_stddev=0.00000000e+00,prob_range={1.00000000e+00,1.00000000e+00},prob(percent)="100",prob+-sd="100+-0"]:6.353620e-03[&length_mean=6.57859035e-03,length_median=6.35362000e-03,length_95%HPD={2.87180700e-03,1.10937400e-02}])[&prob=1.00000000e+00,prob_stddev=0.00000000e+00,prob_range={1.00000000e+00,1.00000000e+00},prob(percent)="100",prob+-sd="100+-0"]:6.792855e-03[&length_mean=7.00778897e-03,length_median=6.79285500e-03,length_95%HPD={2.78999300e-03,1.10873800e-02}]);

end;
