# Supplementary material for: Three new species of arbuscular mycorrhizal fungi (Glomeromycota) and Acaulospora gedanensis revised
Source: Front Microbiol. 2024 Feb 12;15:1320014. doi: 10.3389/fmicb.2024.1320014 (PMC10896085; doi:10.3389/fmicb.2024.1320014)
Supplement: Supplementary Table 9 — Data obtained from a RAxML-NG analysis of 45S+rpb1 sequences (see Figure 1). [file Table_9.DOCX]

(((Diversispora_eburnea_AM713406:0.006563,(Diversispora_eburnea_AM713411:0.000001,(Diversispora_eburnea_AM713407:0.000001,Diversispora_eburnea_AM713408:0.000001)98:0.001291)99:0.001285)100:0.008704,((Diversispora_celata_AY639225:0.001445,Diversispora_celata_AM713403:0.000001)94:0.004857,Diversispora_celata_AM713402:0.002689)100:0.018615)100:0.011544,((((((((Diversispora_arenaria_KJ850188_MG459187:0.004532,(Diversispora_arenaria_KJ850189:0.001200,Diversispora_arenaria_KJ850187:0.001960)51:0.001197)72:0.001113,Diversispora_arenaria_KJ850186_MG459186:0.001740)100:0.006768,((Diversispora_jakucsiae_KJ850182:0.000001,Diversispora_jakucsiae_KJ850181_MG459191:0.000001)100:0.005792,(Diversispora_jakucsiae_KJ850183:0.000624,Diversispora_jakucsiae_KJ850184_MG459190:0.000001)82:0.002338)100:0.004109)100:0.014435,(((Diversispora_slowinskiensis_KT444719:0.000652,Diversispora_slowinskiensis_KT444717_MG459197:0.002628)78:0.002095,Diversispora_slowinskiensis_KT444718:0.000001)96:0.006916,Diversispora_slowinskiensis_KT444720_MG459198:0.001825)100:0.016209)55:0.003089,(Diversispora_valentina_MT985515:0.012124,Diversispora_valentina_MT985516:0.004572)100:0.020289)46:0.002947,(((Diversispora_sporocarpia_MK036785_MK036774:0.000001,Diversispora_sporocarpia_MK036786:0.004511)84:0.002911,(Diversispora_sporocarpia_MK036789:0.001940,Diversispora_sporocarpia_MK036788:0.002593)100:0.003523)87:0.006768,(Diversispora_epigaea_FM876818:0.010924,(Diversispora_epigaea_FM876819:0.006022,(Diversispora_epigaea_FM876817:0.002240,Diversispora_epigaea_FM876814_HG315981:0.004937)100:0.007405)95:0.004448)99:0.009251)100:0.022424)16:0.002234,((((((Redeckera_megalocarpum_HG518628:0.001500,(Redeckera_megalocarpum_HG518627:0.006858,Redeckera_megalocarpum_HG518629:0.003629)63:0.002849)99:0.047068,(Corymbiglomus_corymbiforme_KF060295_MG459179:0.006091,(Corymbiglomus_corymbiforme_KF060298_MG45918:0.006735,Corymbiglomus_corymbiforme_KF060296_MG459181:0.014164)64:0.002344)100:0.072863)93:0.025254,((Siverdingia_tortuosa_JF439095:0.011504,Siverdingia_tortuosa_JF439096:0.002394)83:0.006392,Siverdingia_tortuosa_JF439094:0.002909)100:0.078329)95:0.032571,(Desertispora_omaniana_MG459208_MG459194:0.000001,(Desertispora_omaniana_KF154770_MG459206:0.007207,Desertispora_omaniana_KF154769:0.012672)48:0.001751)100:0.225814)100:0.084121,(((Diversispora_gibbosa_KJ850201_MG459196:0.000001,((Diversispora_gibbosa_KJ850203:0.000001,Diversispora_gibbosa_KJ850202:0.000001)94:0.000001,Diversispora_gibbosa_KJ850204_MG459188:0.002025)48:0.000504)100:0.017329,(Diversispora_peridiata_KT444712_MG459195:0.001545,(Diversispora_peridiata_KT444714_MG459196:0.003487,(Diversispora_peridiata_KT444713:0.000001,Diversispora_peridiata_KT444715:0.001241)77:0.000603)73:0.000694)99:0.006213)95:0.012175,((Diversispora_trimurales_KJ850200_MG459200:0.000001,Diversispora_trimurales_KJ850199_MG459201:0.001034)46:0.001337,Diversispora_trimurales_KJ850198_MG459199:0.015136)100:0.007689)100:0.045616)86:0.017558,((((Diversispora_densissima_MT724384_MT733212:0.004134,(Diversispora_densissima_MT724382_MT733211:0.000001,Diversispora_densissima_MT724383:0.000001)100:0.004044)100:0.008619,(448_4_2:0.008623,448_3_5:0.000997)99:0.006857)96:0.006733,((Diversispora_marina_MT725498:0.000001,Diversispora_marina_MT725499:0.000001)79:0.000653,(Diversispora_marina_MT725501:0.000001,Diversispora_marina_MT725502:0.000001)76:0.000641)100:0.013319)97:0.007807,(((((Diversispora_varaderana_KT444711_MG459203:0.004462,Diversispora_varaderana_KT444709:0.004291)60:0.001287,Diversispora_varaderana_KT444710:0.000742)61:0.001321,Diversispora_varaderana_KT444708_MG459202:0.004045)77:0.003124,((Diversispora_insculpta_KJ850196:0.000001,Diversispora_insculpta_KJ850197_OL690414:0.000186)69:0.000746,Diversispora_insculpta_KJ850195_OL690413:0.002871)100:0.012309)60:0.003362,((Diversispora_aestuarii_OL684642_OL690405:0.006661,(Diversispora_aestuarii_OL684648:0.010304,Diversispora_aestuarii_OL684644_OL690406:0.015358)47:0.005902)55:0.006483,Diversispora_aestuarii_OL684645:0.012087)90:0.004048)100:0.013176)98:0.007197)41:0.003862)38:0.002450,((Diversispora_clara_FR873632:0.004186,(Diversispora_clara_FR873629_MG459184:0.007373,(Diversispora_clara_FR873630:0.001263,Diversispora_clara_FR873631_MG459185:0.002076)24:0.000001)57:0.001974)79:0.002565,(Diversispora_peloponnesiaca_MN306206_OL690409:0.007852,(Diversispora_peloponnesiaca_MN306205_OL690410:0.013821,(Diversispora_peloponnesiaca_MN306208:0.007609,Diversispora_peloponnesiaca_MN306207:0.001354)82:0.004380)61:0.001292)84:0.003784)100:0.019980)62:0.003357,((((Diversispora_aurantia_FN547655:0.001337,Diversispora_aurantia_FN547657_OL690408:0.001584)96:0.003083,(Diversispora_aurantia_FN547661_OL690407:0.000001,Diversispora_aurantia_FN547664:0.000001)100:0.004135)100:0.011801,(((Diversispora_spurca_FN547639_OL690412:0.005041,(Diversispora_spurca_FN547644:0.001881,Diversispora_spurca_FN547637:0.007203)65:0.002653)100:0.011185,Diversispora_spurca_MG459207_OL690411:0.014874)94:0.010832,(((Diversispora_alba_OP195889:0.006890,Diversispora_alba_OP195886:0.002413)94:0.007123,Diversispora_alba_OP195880:0.005728)53:0.001911,Diversispora_alba_OP195882:0.003405)99:0.013126)67:0.004427)100:0.016773,((Diversispora_sabulosa_MG459211_MG459182:0.000001,Diversispora_sabulosa_MG459212:0.000001)99:0.003793,(Diversispora_sabulosa_MG459213_MG459183:0.000001,(Diversispora_sabulosa_MG459214:0.000001,Diversispora_sabulosa_MG459215:0.000001)88:0.000639)100:0.005222)100:0.038366)94:0.009324);
