# Supplementary material for: Three new species of arbuscular mycorrhizal fungi (Glomeromycota) and Acaulospora gedanensis revised
Source: Front Microbiol. 2024 Feb 12;15:1320014. doi: 10.3389/fmicb.2024.1320014 (PMC10896085; doi:10.3389/fmicb.2024.1320014)
Supplement: Supplementary Table 10 — Data obtained from a BI analysis of 45S sequences (see Figure 3). [file Table_10.DOCX]

#NEXUS

[ID: 3279457806]

begin taxa;

dimensions ntax=28;

taxlabels

431_1

431_3

431_5_SSU_ITS_LSU_25_06_2020

431_4_SSU_ITS_LSU_25_06_2020

431_8_SSU_ITS_LSU_25_06_2020

431_2_SSU_ITS_LSU_13_06_2020_cor

437_2_SSU_ITS_LSU_7_12_2020

437_3_SSU_ITS_LSU_7_12_2020

437_9_SSU_ITS_LSU_7_12_2020

437_8_SSU_ITS_LSU_26_11_2020

437_7_SSU_ITS_LSU_7_12_2020

437_4_SSU_ITS_LSU_7_12_2020

Scutellospora_ovalis_KY362434

Scutellospora_ovalis_KY362435

Scutellospora_alterata_HF93501

Scutellospora_alterata_HF935022

Scutellospora_alterata_HF935020

Scutellospora_alterata_HF935021

Scutellospora_calospora_EU346867

Scutellospora_calospora_EU252109_

Scutellospora_dipurpurescens_FJ461868

Scutellospora_alterata_HF935024

Scutellospora_deformata_MZ234124_MZ234127

Scutellospora_spinosissima_FR750149_SSU_ITS_LSU

Orbispora_pernambucana_JF965445

Orbispora_pernambucana_JF965446

Orbispora_pernambucana_JQ340917

Orbispora_pernambucana_JQ340918

;

end;

begin trees;

translate

1 431_1,

2 431_3,

3 431_5_SSU_ITS_LSU_25_06_2020,

4 431_4_SSU_ITS_LSU_25_06_2020,

5 431_8_SSU_ITS_LSU_25_06_2020,

6 431_2_SSU_ITS_LSU_13_06_2020_cor,

7 437_2_SSU_ITS_LSU_7_12_2020,

8 437_3_SSU_ITS_LSU_7_12_2020,

9 437_9_SSU_ITS_LSU_7_12_2020,

10 437_8_SSU_ITS_LSU_26_11_2020,

11 437_7_SSU_ITS_LSU_7_12_2020,

12 437_4_SSU_ITS_LSU_7_12_2020,

13 Scutellospora_ovalis_KY362434,

14 Scutellospora_ovalis_KY362435,

15 Scutellospora_alterata_HF93501,

16 Scutellospora_alterata_HF935022,

17 Scutellospora_alterata_HF935020,

18 Scutellospora_alterata_HF935021,

19 Scutellospora_calospora_EU346867,

20 Scutellospora_calospora_EU252109_,

21 Scutellospora_dipurpurescens_FJ461868,

22 Scutellospora_alterata_HF935024,

23 Scutellospora_deformata_MZ234124_MZ234127,

24 Scutellospora_spinosissima_FR750149_SSU_ITS_LSU,

25 Orbispora_pernambucana_JF965445,

26 Orbispora_pernambucana_JF965446,

27 Orbispora_pernambucana_JQ340917,

28 Orbispora_pernambucana_JQ340918

;

tree con_50_majrule = [&U] (1[&prob=1.00000000e+00,prob_stddev=0.00000000e+00,prob_range={1.00000000e+00,1.00000000e+00},prob(percent)="100",prob+-sd="100+-0"]:1.325444e-03[&length_mean=1.59510209e-03,length_median=1.32544400e-03,length_95%HPD={3.70388600e-06,3.83410700e-03}],(5[&prob=1.00000000e+00,prob_stddev=0.00000000e+00,prob_range={1.00000000e+00,1.00000000e+00},prob(percent)="100",prob+-sd="100+-0"]:1.401308e-02[&length_mean=1.42605547e-02,length_median=1.40130800e-02,length_95%HPD={7.34481700e-03,2.12795800e-02}],6[&prob=1.00000000e+00,prob_stddev=0.00000000e+00,prob_range={1.00000000e+00,1.00000000e+00},prob(percent)="100",prob+-sd="100+-0"]:1.392116e-02[&length_mean=1.42121675e-02,length_median=1.39211600e-02,length_95%HPD={7.74393100e-03,2.18093100e-02}])[&prob=8.12916112e-01,prob_stddev=3.67205918e-02,prob_range={7.86950732e-01,8.38881491e-01},prob(percent)="81",prob+-sd="81+-4"]:4.456423e-03[&length_mean=4.66449093e-03,length_median=4.45642300e-03,length_95%HPD={8.61074200e-04,8.59796000e-03}],((2[&prob=1.00000000e+00,prob_stddev=0.00000000e+00,prob_range={1.00000000e+00,1.00000000e+00},prob(percent)="100",prob+-sd="100+-0"]:1.209620e-03[&length_mean=1.42191620e-03,length_median=1.20962000e-03,length_95%HPD={1.72209400e-05,3.22981800e-03}],3[&prob=1.00000000e+00,prob_stddev=0.00000000e+00,prob_range={1.00000000e+00,1.00000000e+00},prob(percent)="100",prob+-sd="100+-0"]:5.147746e-04[&length_mean=7.22785616e-04,length_median=5.14774600e-04,length_95%HPD={3.54615800e-08,2.12140500e-03}],4[&prob=1.00000000e+00,prob_stddev=0.00000000e+00,prob_range={1.00000000e+00,1.00000000e+00},prob(percent)="100",prob+-sd="100+-0"]:3.290606e-03[&length_mean=3.56970008e-03,length_median=3.29060600e-03,length_95%HPD={7.77057000e-04,6.83159300e-03}])[&prob=5.82556591e-01,prob_stddev=3.86036991e-02,prob_range={5.55259654e-01,6.09853529e-01},prob(percent)="58",prob+-sd="58+-4"]:2.090175e-03[&length_mean=2.54179086e-03,length_median=2.09017500e-03,length_95%HPD={2.12727300e-05,6.31752600e-03}],(((7[&prob=1.00000000e+00,prob_stddev=0.00000000e+00,prob_range={1.00000000e+00,1.00000000e+00},prob(percent)="100",prob+-sd="100+-0"]:1.596124e-03[&length_mean=1.86213094e-03,length_median=1.59612400e-03,length_95%HPD={3.10804100e-05,4.11685500e-03}],12[&prob=1.00000000e+00,prob_stddev=0.00000000e+00,prob_range={1.00000000e+00,1.00000000e+00},prob(percent)="100",prob+-sd="100+-0"]:1.915567e-03[&length_mean=2.13149375e-03,length_median=1.91556700e-03,length_95%HPD={1.21075100e-04,4.47616900e-03}])[&prob=5.57922770e-01,prob_stddev=9.41553637e-03,prob_range={5.51264980e-01,5.64580559e-01},prob(percent)="56",prob+-sd="56+-1"]:1.225706e-03[&length_mean=1.44854174e-03,length_median=1.22570600e-03,length_95%HPD={1.63893400e-05,3.49879100e-03}],8[&prob=1.00000000e+00,prob_stddev=0.00000000e+00,prob_range={1.00000000e+00,1.00000000e+00},prob(percent)="100",prob+-sd="100+-0"]:8.374787e-04[&length_mean=1.08025429e-03,length_median=8.37478700e-04,length_95%HPD={3.07357200e-06,2.96048000e-03}],9[&prob=1.00000000e+00,prob_stddev=0.00000000e+00,prob_range={1.00000000e+00,1.00000000e+00},prob(percent)="100",prob+-sd="100+-0"]:4.925046e-04[&length_mean=6.91572126e-04,length_median=4.92504600e-04,length_95%HPD={2.98660400e-07,1.96400500e-03}],10[&prob=1.00000000e+00,prob_stddev=0.00000000e+00,prob_range={1.00000000e+00,1.00000000e+00},prob(percent)="100",prob+-sd="100+-0"]:4.526524e-04[&length_mean=7.16296802e-04,length_median=4.52652400e-04,length_95%HPD={9.27588100e-08,2.32713800e-03}],11[&prob=1.00000000e+00,prob_stddev=0.00000000e+00,prob_range={1.00000000e+00,1.00000000e+00},prob(percent)="100",prob+-sd="100+-0"]:1.225850e-03[&length_mean=1.45894055e-03,length_median=1.22585000e-03,length_95%HPD={2.84797700e-05,3.51022600e-03}])[&prob=1.00000000e+00,prob_stddev=0.00000000e+00,prob_range={1.00000000e+00,1.00000000e+00},prob(percent)="100",prob+-sd="100+-0"]:2.152363e-02[&length_mean=2.18469446e-02,length_median=2.15236300e-02,length_95%HPD={1.29747300e-02,3.13023400e-02}],((13[&prob=1.00000000e+00,prob_stddev=0.00000000e+00,prob_range={1.00000000e+00,1.00000000e+00},prob(percent)="100",prob+-sd="100+-0"]:4.838714e-04[&length_mean=7.29473627e-04,length_median=4.83871400e-04,length_95%HPD={1.46300200e-06,2.17671000e-03}],14[&prob=1.00000000e+00,prob_stddev=0.00000000e+00,prob_range={1.00000000e+00,1.00000000e+00},prob(percent)="100",prob+-sd="100+-0"]:5.079569e-04[&length_mean=7.26946849e-04,length_median=5.07956900e-04,length_95%HPD={4.97280500e-07,2.21137200e-03}])[&prob=1.00000000e+00,prob_stddev=0.00000000e+00,prob_range={1.00000000e+00,1.00000000e+00},prob(percent)="100",prob+-sd="100+-0"]:3.817519e-02[&length_mean=3.85913879e-02,length_median=3.81751900e-02,length_95%HPD={2.44322300e-02,5.16872200e-02}],(((((15[&prob=1.00000000e+00,prob_stddev=0.00000000e+00,prob_range={1.00000000e+00,1.00000000e+00},prob(percent)="100",prob+-sd="100+-0"]:2.584850e-03[&length_mean=2.78991709e-03,length_median=2.58485000e-03,length_95%HPD={5.22408600e-04,5.51061200e-03}],16[&prob=1.00000000e+00,prob_stddev=0.00000000e+00,prob_range={1.00000000e+00,1.00000000e+00},prob(percent)="100",prob+-sd="100+-0"]:1.895311e-03[&length_mean=2.14051497e-03,length_median=1.89531100e-03,length_95%HPD={2.02492600e-04,4.49906400e-03}])[&prob=9.78695073e-01,prob_stddev=3.76621455e-03,prob_range={9.76031957e-01,9.81358189e-01},prob(percent)="98",prob+-sd="98+-0"]:1.177654e-03[&length_mean=1.44886572e-03,length_median=1.17765400e-03,length_95%HPD={2.83207600e-05,3.44377900e-03}],17[&prob=1.00000000e+00,prob_stddev=0.00000000e+00,prob_range={1.00000000e+00,1.00000000e+00},prob(percent)="100",prob+-sd="100+-0"]:3.319009e-03[&length_mean=3.61020209e-03,length_median=3.31900900e-03,length_95%HPD={6.08686100e-04,6.89473100e-03}],18[&prob=1.00000000e+00,prob_stddev=0.00000000e+00,prob_range={1.00000000e+00,1.00000000e+00},prob(percent)="100",prob+-sd="100+-0"]:3.180478e-03[&length_mean=3.40427454e-03,length_median=3.18047800e-03,length_95%HPD={7.71083100e-04,6.72469300e-03}])[&prob=1.00000000e+00,prob_stddev=0.00000000e+00,prob_range={1.00000000e+00,1.00000000e+00},prob(percent)="100",prob+-sd="100+-0"]:1.648427e-02[&length_mean=1.69863483e-02,length_median=1.64842700e-02,length_95%HPD={9.11226300e-03,2.82315200e-02}],22[&prob=1.00000000e+00,prob_stddev=0.00000000e+00,prob_range={1.00000000e+00,1.00000000e+00},prob(percent)="100",prob+-sd="100+-0"]:1.553192e-02[&length_mean=1.58787721e-02,length_median=1.55319200e-02,length_95%HPD={6.46989900e-03,2.46911100e-02}])[&prob=1.00000000e+00,prob_stddev=0.00000000e+00,prob_range={1.00000000e+00,1.00000000e+00},prob(percent)="100",prob+-sd="100+-0"]:2.461538e-02[&length_mean=2.49503805e-02,length_median=2.46153800e-02,length_95%HPD={1.36209000e-02,3.71719100e-02}],(19[&prob=1.00000000e+00,prob_stddev=0.00000000e+00,prob_range={1.00000000e+00,1.00000000e+00},prob(percent)="100",prob+-sd="100+-0"]:5.963435e-03[&length_mean=6.54221982e-03,length_median=5.96343500e-03,length_95%HPD={1.02558600e-03,1.33679800e-02}],20[&prob=1.00000000e+00,prob_stddev=0.00000000e+00,prob_range={1.00000000e+00,1.00000000e+00},prob(percent)="100",prob+-sd="100+-0"]:7.712697e-03[&length_mean=8.47242308e-03,length_median=7.71269700e-03,length_95%HPD={2.03604300e-03,1.65644800e-02}])[&prob=9.63382157e-01,prob_stddev=9.41553637e-04,prob_range={9.62716378e-01,9.64047936e-01},prob(percent)="96",prob+-sd="96+-0"]:4.100848e-03[&length_mean=4.78327331e-03,length_median=4.10084800e-03,length_95%HPD={8.36498000e-06,1.07186300e-02}],21[&prob=1.00000000e+00,prob_stddev=0.00000000e+00,prob_range={1.00000000e+00,1.00000000e+00},prob(percent)="100",prob+-sd="100+-0"]:1.144154e-02[&length_mean=1.20735958e-02,length_median=1.14415400e-02,length_95%HPD={1.98644900e-03,2.42829700e-02}],23[&prob=1.00000000e+00,prob_stddev=0.00000000e+00,prob_range={1.00000000e+00,1.00000000e+00},prob(percent)="100",prob+-sd="100+-0"]:2.096595e-02[&length_mean=2.11558494e-02,length_median=2.09659500e-02,length_95%HPD={6.10033600e-03,3.49961500e-02}],(25[&prob=1.00000000e+00,prob_stddev=0.00000000e+00,prob_range={1.00000000e+00,1.00000000e+00},prob(percent)="100",prob+-sd="100+-0"]:4.348246e-03[&length_mean=4.85650836e-03,length_median=4.34824600e-03,length_95%HPD={3.92257400e-05,1.04426100e-02}],26[&prob=1.00000000e+00,prob_stddev=0.00000000e+00,prob_range={1.00000000e+00,1.00000000e+00},prob(percent)="100",prob+-sd="100+-0"]:1.168259e-03[&length_mean=1.65175215e-03,length_median=1.16825900e-03,length_95%HPD={9.63601000e-07,5.13880800e-03}],27[&prob=1.00000000e+00,prob_stddev=0.00000000e+00,prob_range={1.00000000e+00,1.00000000e+00},prob(percent)="100",prob+-sd="100+-0"]:2.883550e-03[&length_mean=3.38299881e-03,length_median=2.88355000e-03,length_95%HPD={1.81631500e-05,8.13420500e-03}],28[&prob=1.00000000e+00,prob_stddev=0.00000000e+00,prob_range={1.00000000e+00,1.00000000e+00},prob(percent)="100",prob+-sd="100+-0"]:4.429791e-03[&length_mean=5.01507213e-03,length_median=4.42979100e-03,length_95%HPD={6.06561700e-04,1.13130100e-02}])[&prob=1.00000000e+00,prob_stddev=0.00000000e+00,prob_range={1.00000000e+00,1.00000000e+00},prob(percent)="100",prob+-sd="100+-0"]:1.021796e-01[&length_mean=1.03543860e-01,length_median=1.02179600e-01,length_95%HPD={6.51930400e-02,1.39406700e-01}])[&prob=9.99334221e-01,prob_stddev=9.41553637e-04,prob_range={9.98668442e-01,1.00000000e+00},prob(percent)="100",prob+-sd="100+-0"]:1.847460e-02[&length_mean=1.89832253e-02,length_median=1.84746000e-02,length_95%HPD={7.77954900e-03,3.05643200e-02}],24[&prob=1.00000000e+00,prob_stddev=0.00000000e+00,prob_range={1.00000000e+00,1.00000000e+00},prob(percent)="100",prob+-sd="100+-0"]:5.293369e-02[&length_mean=5.33697185e-02,length_median=5.29336900e-02,length_95%HPD={3.71994800e-02,6.98783000e-02}])[&prob=9.36750999e-01,prob_stddev=2.16557336e-02,prob_range={9.21438083e-01,9.52063915e-01},prob(percent)="94",prob+-sd="94+-2"]:1.031511e-02[&length_mean=1.09501484e-02,length_median=1.03151100e-02,length_95%HPD={2.34393500e-03,2.12698700e-02}])[&prob=9.98002663e-01,prob_stddev=9.41553637e-04,prob_range={9.97336884e-01,9.98668442e-01},prob(percent)="100",prob+-sd="100+-0"]:1.216641e-02[&length_mean=1.25172279e-02,length_median=1.21664100e-02,length_95%HPD={4.02242500e-03,2.03314500e-02}])[&prob=1.00000000e+00,prob_stddev=0.00000000e+00,prob_range={1.00000000e+00,1.00000000e+00},prob(percent)="100",prob+-sd="100+-0"]:9.459320e-03[&length_mean=9.63776283e-03,length_median=9.45932000e-03,length_95%HPD={3.59652400e-03,1.64739700e-02}])[&prob=7.72303595e-01,prob_stddev=4.14283600e-02,prob_range={7.43009321e-01,8.01597870e-01},prob(percent)="77",prob+-sd="77+-4"]:3.613910e-03[&length_mean=3.84351241e-03,length_median=3.61391000e-03,length_95%HPD={5.36316200e-04,7.08617400e-03}]);

end;
