# Supplementary material for: Three new species of arbuscular mycorrhizal fungi (Glomeromycota) and Acaulospora gedanensis revised
Source: Front Microbiol. 2024 Feb 12;15:1320014. doi: 10.3389/fmicb.2024.1320014 (PMC10896085; doi:10.3389/fmicb.2024.1320014)
Supplement: Supplementary Table 11 — Data obtained from a RAxML-NG analysis of 45S sequences (see Figure 3). [file Table_11.DOCX]

((Orbispora_pernambucana_JF965445:0.004015,Orbispora_pernambucana_JF965446:0.000001)17:0.000001,Orbispora_pernambucana_JQ340917:0.001987,((Scutellospora_dipurpurescens_FJ461868:0.008630,((Scutellospora_calospora_EU252109:0.007889,Scutellospora_calospora_EU346867:0.005825)78:0.003829,((Scutellospora_alterata_HF935024:0.015925,((Scutellospora_alterata_HF935020:0.002986,(Scutellospora_alterata_HF93501:0.002219,Scutellospora_alterata_HF935022:0.001480)76:0.000753)73:0.000001,Scutellospora_alterata_HF935021:0.002964)99:0.016533)98:0.026502,(Scutellospora_deformata_MZ234124_MZ234127:0.025078,(Scutellospora_spinosissima_FR750149_SSU_ITS_LSU:0.056888,((((((437_8_SSU_ITS_LSU_26_11_2020:0.000001,437_7_SSU_ITS_LSU_7_12_2020:0.000746)25:0.000001,437_9_SSU_ITS_LSU_7_12_2020:0.000001)37:0.000001,(437_3_SSU_ITS_LSU_7_12_2020:0.000001,437_4_SSU_ITS_LSU_7_12_2020:0.001494)41:0.000745)19:0.000001,437_2_SSU_ITS_LSU_7_12_2020:0.001492)100:0.021667,(((431_3:0.000742,431_5_SSU_ITS_LSU_25_06_2020:0.000001)74:0.000001,431_4_SSU_ITS_LSU_25_06_2020:0.002987)68:0.000640,(431_1:0.000776,(431_8_SSU_ITS_LSU_25_06_2020:0.013982,431_2_SSU_ITS_LSU_13_06_2020_cor:0.013863)49:0.004215)45:0.003131)84:0.009247)85:0.011854,(Scutellospora_ovalis_KY362435:0.000001,Scutellospora_ovalis_KY362434:0.000001)100:0.039980)69:0.010060)91:0.020057)26:0.001604)44:0.003633)39:0.006305)100:0.135998,Orbispora_pernambucana_JQ340918:0.003997)21:0.000001);
