# Supplementary material for: Three new species of arbuscular mycorrhizal fungi (Glomeromycota) and Acaulospora gedanensis revised
Source: Front Microbiol. 2024 Feb 12;15:1320014. doi: 10.3389/fmicb.2024.1320014 (PMC10896085; doi:10.3389/fmicb.2024.1320014)
Supplement: Supplementary Table 12 — Data obtained from a BI analysis of 45S sequences (see Figure 7). [file Table_12.DOCX]

#NEXUS

[ID: 2979083984]

begin taxa;

dimensions ntax=76;

taxlabels

Acaulospora_brasiliensis_FN825902

Acaulospora_brasiliensis_FN825904

Acaulospora_brasiliensis_FN825905

Acaulospora_brasiliensis_FN825909

Acaulospora_brasiliensis_FN825906

Acaulospora_brasiliensis_FN825907

Acaulospora_brasiliensis_FN825903

Acaulospora_brasiliensis_FN825908

Acaulospora_brasiliensis_FN825910

Acaulospora_brasiliensis_FN825911

Acaulospora_gedanensis_1_SSU_ITS_LSU_5_08_2022

Acaulospora_gedanensis_5_SSU_ITS_LSU_5_08_2022

Acaulospora_gedanensis_4_SSU_ITS_LSU_5_08_2022

Acaulospora_gedanensis_1_SSU_ITS_LSU_5_09_2022

Acaulospora_gedanensis_3_SSU_ITS_LSU_5_09_2022

Acaulospora_gedanensis_2_SSU_ITS_LSU_5_09_2022

Acaulospora_gedanensis_6_SSU_ITS_LSU_5_08_2022

Acaulospora_gedanensis_3_SSU_ITS_LSU_12_09_2022

HF567941_Acaulospora_pustulata

HF567939_Acaulospora_pustulata

HF567938_Acaulospora_pustulata

FR681927_Acaulospora_alpina

FR681930_Acaulospora_alpina

Acaulospora_fanjing_MW723431

HF567933_Acaulospora_tortuosa

HF567936_Acaulospora_tortuosa

GU326346_Acaulospora_colliculosa

GU326352_Acaulospora_colliculosa

MH045498_Acaulospora_tsugae

KP756456_Acaulospora_mellea

KY565429_Acaulospora_koreana

KP756584_Acaulospora_lacunosa

LN736022_Acaulospora_foveata

AF133764_Acaulospora_colossica

Acaulospora_laevis_FN547516

FR750173_Acaulospora_entreriana

HG421736_Acaulospora_viridis

Acaulospora_intravesiculata_OL661628

KP191475_Acaulospora_koskei

Acaulospora_flavopapillosa_OK360960

LN884303_Acaulospora_papillosa

JF439093_Acaulospora_delicata

MT832212_Acaulospora_dilatata

AM040291_Acaulospora_longula

LN881566_Acaulospora_rugosa

KY362433_Acaulospora_fragilissima

KY362428_Acaulospora_saccata

AJ242500_Acaulospora_morrowiae

Acaulospora_mendoncae_OK392597

MN081001_Acaulospora_aspera

HG422734_Acaulospora_spinosissima

JX135571_Acaulospora_herrerae

FM876830_Acaulospora_kentinensis

FR821674_Acaulospora_minuta

FR692354_Acaulospora_scrobiculata

FJ461799_Acaulospora_tuberculata

FR869691_Acaulospora_minuta

FR750152_Acaulospora_spinosa

KM057063_Acaulospora_reducta

KM057074_Acaulospora_excavata

LN811001_Acaulospora_baetica

KP191472_Acaulospora_ignota

HE603644_Acaulospora_nivalis

FM876788_Acaulospora_cavernata

FR846385_Acaulospora_punctata

KY413814_Acaulospora_spinulifera

AJ891119_Acaulospora_paulinae

AM076382_Acaulospora_sieverdingii

AJ239115_Acaulospora_denticulata

FR750063_Acaulospora_colombiana

KX355819_Sacculospora_baltica

KX355821_Sacculospora_baltica

KX355818_Sacculospora_baltica

KX345938_Sacculospora_felinovii

KX345939_Sacculospora_felinovii

KX345941_Sacculospora_felinovii

;

end;

begin trees;

translate

1 Acaulospora_brasiliensis_FN825902,

2 Acaulospora_brasiliensis_FN825904,

3 Acaulospora_brasiliensis_FN825905,

4 Acaulospora_brasiliensis_FN825909,

5 Acaulospora_brasiliensis_FN825906,

6 Acaulospora_brasiliensis_FN825907,

7 Acaulospora_brasiliensis_FN825903,

8 Acaulospora_brasiliensis_FN825908,

9 Acaulospora_brasiliensis_FN825910,

10 Acaulospora_brasiliensis_FN825911,

11 Acaulospora_gedanensis_1_SSU_ITS_LSU_5_08_2022,

12 Acaulospora_gedanensis_5_SSU_ITS_LSU_5_08_2022,

13 Acaulospora_gedanensis_4_SSU_ITS_LSU_5_08_2022,

14 Acaulospora_gedanensis_1_SSU_ITS_LSU_5_09_2022,

15 Acaulospora_gedanensis_3_SSU_ITS_LSU_5_09_2022,

16 Acaulospora_gedanensis_2_SSU_ITS_LSU_5_09_2022,

17 Acaulospora_gedanensis_6_SSU_ITS_LSU_5_08_2022,

18 Acaulospora_gedanensis_3_SSU_ITS_LSU_12_09_2022,

19 HF567941_Acaulospora_pustulata,

20 HF567939_Acaulospora_pustulata,

21 HF567938_Acaulospora_pustulata,

22 FR681927_Acaulospora_alpina,

23 FR681930_Acaulospora_alpina,

24 Acaulospora_fanjing_MW723431,

25 HF567933_Acaulospora_tortuosa,

26 HF567936_Acaulospora_tortuosa,

27 GU326346_Acaulospora_colliculosa,

28 GU326352_Acaulospora_colliculosa,

29 MH045498_Acaulospora_tsugae,

30 KP756456_Acaulospora_mellea,

31 KY565429_Acaulospora_koreana,

32 KP756584_Acaulospora_lacunosa,

33 LN736022_Acaulospora_foveata,

34 AF133764_Acaulospora_colossica,

35 Acaulospora_laevis_FN547516,

36 FR750173_Acaulospora_entreriana,

37 HG421736_Acaulospora_viridis,

38 Acaulospora_intravesiculata_OL661628,

39 KP191475_Acaulospora_koskei,

40 Acaulospora_flavopapillosa_OK360960,

41 LN884303_Acaulospora_papillosa,

42 JF439093_Acaulospora_delicata,

43 MT832212_Acaulospora_dilatata,

44 AM040291_Acaulospora_longula,

45 LN881566_Acaulospora_rugosa,

46 KY362433_Acaulospora_fragilissima,

47 KY362428_Acaulospora_saccata,

48 AJ242500_Acaulospora_morrowiae,

49 Acaulospora_mendoncae_OK392597,

50 MN081001_Acaulospora_aspera,

51 HG422734_Acaulospora_spinosissima,

52 JX135571_Acaulospora_herrerae,

53 FM876830_Acaulospora_kentinensis,

54 FR821674_Acaulospora_minuta,

55 FR692354_Acaulospora_scrobiculata,

56 FJ461799_Acaulospora_tuberculata,

57 FR869691_Acaulospora_minuta,

58 FR750152_Acaulospora_spinosa,

59 KM057063_Acaulospora_reducta,

60 KM057074_Acaulospora_excavata,

61 LN811001_Acaulospora_baetica,

62 KP191472_Acaulospora_ignota,

63 HE603644_Acaulospora_nivalis,

64 FM876788_Acaulospora_cavernata,

65 FR846385_Acaulospora_punctata,

66 KY413814_Acaulospora_spinulifera,

67 AJ891119_Acaulospora_paulinae,

68 AM076382_Acaulospora_sieverdingii,

69 AJ239115_Acaulospora_denticulata,

70 FR750063_Acaulospora_colombiana,

71 KX355819_Sacculospora_baltica,

72 KX355821_Sacculospora_baltica,

73 KX355818_Sacculospora_baltica,

74 KX345938_Sacculospora_felinovii,

75 KX345939_Sacculospora_felinovii,

76 KX345941_Sacculospora_felinovii

;

tree con_50_majrule = [&U] (1[&prob=1.00000000e+00,prob_stddev=0.00000000e+00,prob_range={1.00000000e+00,1.00000000e+00},prob(percent)="100",prob+-sd="100+-0"]:6.104695e-04[&length_mean=8.96805383e-04,length_median=6.10469500e-04,length_95%HPD={1.62586300e-06,2.67135900e-03}],6[&prob=1.00000000e+00,prob_stddev=0.00000000e+00,prob_range={1.00000000e+00,1.00000000e+00},prob(percent)="100",prob+-sd="100+-0"]:4.919439e-03[&length_mean=5.17072631e-03,length_median=4.91943900e-03,length_95%HPD={1.29171600e-03,9.24573700e-03}],((2[&prob=1.00000000e+00,prob_stddev=0.00000000e+00,prob_range={1.00000000e+00,1.00000000e+00},prob(percent)="100",prob+-sd="100+-0"]:6.324250e-04[&length_mean=9.48598821e-04,length_median=6.32425000e-04,length_95%HPD={1.52720300e-07,2.83140400e-03}],(3[&prob=1.00000000e+00,prob_stddev=0.00000000e+00,prob_range={1.00000000e+00,1.00000000e+00},prob(percent)="100",prob+-sd="100+-0"]:5.373427e-04[&length_mean=8.05111096e-04,length_median=5.37342700e-04,length_95%HPD={2.61931900e-07,2.42020200e-03}],4[&prob=1.00000000e+00,prob_stddev=0.00000000e+00,prob_range={1.00000000e+00,1.00000000e+00},prob(percent)="100",prob+-sd="100+-0"]:5.712108e-04[&length_mean=8.17931747e-04,length_median=5.71210800e-04,length_95%HPD={1.08798300e-06,2.64330500e-03}])[&prob=8.31557923e-01,prob_stddev=1.60064118e-02,prob_range={8.20239680e-01,8.42876165e-01},prob(percent)="83",prob+-sd="83+-2"]:1.431272e-03[&length_mean=1.66368220e-03,length_median=1.43127200e-03,length_95%HPD={2.12410600e-06,3.86498800e-03}])[&prob=6.39813582e-01,prob_stddev=6.59087546e-03,prob_range={6.35153129e-01,6.44474035e-01},prob(percent)="64",prob+-sd="64+-1"]:1.345070e-03[&length_mean=1.70111058e-03,length_median=1.34507000e-03,length_95%HPD={4.81179700e-06,4.04140800e-03}],((5[&prob=1.00000000e+00,prob_stddev=0.00000000e+00,prob_range={1.00000000e+00,1.00000000e+00},prob(percent)="100",prob+-sd="100+-0"]:1.933952e-03[&length_mean=2.19047062e-03,length_median=1.93395200e-03,length_95%HPD={1.04319100e-04,4.91639600e-03}],7[&prob=1.00000000e+00,prob_stddev=0.00000000e+00,prob_range={1.00000000e+00,1.00000000e+00},prob(percent)="100",prob+-sd="100+-0"]:4.110316e-03[&length_mean=4.39396974e-03,length_median=4.11031600e-03,length_95%HPD={1.13157600e-03,8.36847400e-03}],((9[&prob=1.00000000e+00,prob_stddev=0.00000000e+00,prob_range={1.00000000e+00,1.00000000e+00},prob(percent)="100",prob+-sd="100+-0"]:5.601679e-03[&length_mean=5.90043883e-03,length_median=5.60167900e-03,length_95%HPD={2.10605900e-03,1.03962900e-02}],10[&prob=1.00000000e+00,prob_stddev=0.00000000e+00,prob_range={1.00000000e+00,1.00000000e+00},prob(percent)="100",prob+-sd="100+-0"]:3.845494e-03[&length_mean=4.15015779e-03,length_median=3.84549400e-03,length_95%HPD={1.07140300e-03,7.94302600e-03}])[&prob=1.00000000e+00,prob_stddev=0.00000000e+00,prob_range={1.00000000e+00,1.00000000e+00},prob(percent)="100",prob+-sd="100+-0"]:4.844974e-03[&length_mean=5.07320429e-03,length_median=4.84497400e-03,length_95%HPD={1.21536700e-03,9.22214000e-03}],(((11[&prob=1.00000000e+00,prob_stddev=0.00000000e+00,prob_range={1.00000000e+00,1.00000000e+00},prob(percent)="100",prob+-sd="100+-0"]:1.360959e-03[&length_mean=1.63415327e-03,length_median=1.36095900e-03,length_95%HPD={3.52020100e-05,3.91518200e-03}],13[&prob=1.00000000e+00,prob_stddev=0.00000000e+00,prob_range={1.00000000e+00,1.00000000e+00},prob(percent)="100",prob+-sd="100+-0"]:1.312262e-03[&length_mean=1.56810948e-03,length_median=1.31226200e-03,length_95%HPD={1.15804400e-05,3.74533500e-03}])[&prob=9.30758988e-01,prob_stddev=1.31817509e-02,prob_range={9.21438083e-01,9.40079893e-01},prob(percent)="93",prob+-sd="93+-1"]:1.734714e-03[&length_mean=2.02012073e-03,length_median=1.73471400e-03,length_95%HPD={2.01775400e-05,4.68379100e-03}],(12[&prob=1.00000000e+00,prob_stddev=0.00000000e+00,prob_range={1.00000000e+00,1.00000000e+00},prob(percent)="100",prob+-sd="100+-0"]:1.343588e-03[&length_mean=1.61349939e-03,length_median=1.34358800e-03,length_95%HPD={4.97482800e-05,3.77464400e-03}],14[&prob=1.00000000e+00,prob_stddev=0.00000000e+00,prob_range={1.00000000e+00,1.00000000e+00},prob(percent)="100",prob+-sd="100+-0"]:2.237587e-03[&length_mean=2.47475778e-03,length_median=2.23758700e-03,length_95%HPD={4.21188100e-04,5.41915400e-03}],18[&prob=1.00000000e+00,prob_stddev=0.00000000e+00,prob_range={1.00000000e+00,1.00000000e+00},prob(percent)="100",prob+-sd="100+-0"]:5.334676e-03[&length_mean=5.67050049e-03,length_median=5.33467600e-03,length_95%HPD={1.86254200e-03,1.03309300e-02}])[&prob=9.60719041e-01,prob_stddev=1.60064118e-02,prob_range={9.49400799e-01,9.72037284e-01},prob(percent)="96",prob+-sd="96+-2"]:1.306306e-03[&length_mean=1.56711457e-03,length_median=1.30630600e-03,length_95%HPD={3.93253700e-05,3.70285000e-03}],(15[&prob=1.00000000e+00,prob_stddev=0.00000000e+00,prob_range={1.00000000e+00,1.00000000e+00},prob(percent)="100",prob+-sd="100+-0"]:5.553587e-04[&length_mean=8.33062813e-04,length_median=5.55358700e-04,length_95%HPD={1.96655600e-07,2.52327400e-03}],16[&prob=1.00000000e+00,prob_stddev=0.00000000e+00,prob_range={1.00000000e+00,1.00000000e+00},prob(percent)="100",prob+-sd="100+-0"]:3.476034e-03[&length_mean=3.81612452e-03,length_median=3.47603400e-03,length_95%HPD={1.03962600e-03,7.51710900e-03}])[&prob=9.62716378e-01,prob_stddev=1.31817509e-02,prob_range={9.53395473e-01,9.72037284e-01},prob(percent)="96",prob+-sd="96+-1"]:2.169909e-03[&length_mean=2.43009243e-03,length_median=2.16990900e-03,length_95%HPD={6.95685000e-05,5.45783900e-03}],17[&prob=1.00000000e+00,prob_stddev=0.00000000e+00,prob_range={1.00000000e+00,1.00000000e+00},prob(percent)="100",prob+-sd="100+-0"]:3.713301e-03[&length_mean=4.02331962e-03,length_median=3.71330100e-03,length_95%HPD={1.01322200e-03,7.53457100e-03}])[&prob=1.00000000e+00,prob_stddev=0.00000000e+00,prob_range={1.00000000e+00,1.00000000e+00},prob(percent)="100",prob+-sd="100+-0"]:8.264019e-03[&length_mean=8.66915277e-03,length_median=8.26401900e-03,length_95%HPD={2.72549500e-03,1.47502900e-02}],((19[&prob=1.00000000e+00,prob_stddev=0.00000000e+00,prob_range={1.00000000e+00,1.00000000e+00},prob(percent)="100",prob+-sd="100+-0"]:1.097348e-02[&length_mean=1.12491519e-02,length_median=1.09734800e-02,length_95%HPD={5.12650100e-03,1.72068400e-02}],(20[&prob=1.00000000e+00,prob_stddev=0.00000000e+00,prob_range={1.00000000e+00,1.00000000e+00},prob(percent)="100",prob+-sd="100+-0"]:4.638356e-03[&length_mean=4.82558145e-03,length_median=4.63835600e-03,length_95%HPD={1.36944400e-03,8.44884800e-03}],21[&prob=1.00000000e+00,prob_stddev=0.00000000e+00,prob_range={1.00000000e+00,1.00000000e+00},prob(percent)="100",prob+-sd="100+-0"]:6.304691e-03[&length_mean=6.63741704e-03,length_median=6.30469100e-03,length_95%HPD={2.77498100e-03,1.18390200e-02}])[&prob=9.96005326e-01,prob_stddev=0.00000000e+00,prob_range={9.96005326e-01,9.96005326e-01},prob(percent)="100",prob+-sd="100+-0"]:3.327881e-03[&length_mean=3.70944007e-03,length_median=3.32788100e-03,length_95%HPD={3.80998200e-04,7.67249100e-03}])[&prob=1.00000000e+00,prob_stddev=0.00000000e+00,prob_range={1.00000000e+00,1.00000000e+00},prob(percent)="100",prob+-sd="100+-0"]:2.098132e-02[&length_mean=2.13114713e-02,length_median=2.09813200e-02,length_95%HPD={1.21916800e-02,3.10524000e-02}],((((22[&prob=1.00000000e+00,prob_stddev=0.00000000e+00,prob_range={1.00000000e+00,1.00000000e+00},prob(percent)="100",prob+-sd="100+-0"]:7.824017e-03[&length_mean=8.17566754e-03,length_median=7.82401700e-03,length_95%HPD={3.29025900e-03,1.37247800e-02}],23[&prob=1.00000000e+00,prob_stddev=0.00000000e+00,prob_range={1.00000000e+00,1.00000000e+00},prob(percent)="100",prob+-sd="100+-0"]:5.418865e-03[&length_mean=5.76950942e-03,length_median=5.41886500e-03,length_95%HPD={1.72697200e-03,1.06161400e-02}])[&prob=1.00000000e+00,prob_stddev=0.00000000e+00,prob_range={1.00000000e+00,1.00000000e+00},prob(percent)="100",prob+-sd="100+-0"]:2.866332e-02[&length_mean=2.89528131e-02,length_median=2.86633200e-02,length_95%HPD={1.70823900e-02,4.30630300e-02}],(24[&prob=1.00000000e+00,prob_stddev=0.00000000e+00,prob_range={1.00000000e+00,1.00000000e+00},prob(percent)="100",prob+-sd="100+-0"]:3.576167e-02[&length_mean=3.59513859e-02,length_median=3.57616700e-02,length_95%HPD={2.25794800e-02,4.95692400e-02}],(((((30[&prob=1.00000000e+00,prob_stddev=0.00000000e+00,prob_range={1.00000000e+00,1.00000000e+00},prob(percent)="100",prob+-sd="100+-0"]:2.732092e-02[&length_mean=2.75535380e-02,length_median=2.73209200e-02,length_95%HPD={1.75095400e-02,3.96820300e-02}],31[&prob=1.00000000e+00,prob_stddev=0.00000000e+00,prob_range={1.00000000e+00,1.00000000e+00},prob(percent)="100",prob+-sd="100+-0"]:1.326322e-02[&length_mean=1.36956727e-02,length_median=1.32632200e-02,length_95%HPD={5.59176900e-03,2.14279500e-02}])[&prob=8.93475366e-01,prob_stddev=1.88310727e-03,prob_range={8.92143808e-01,8.94806924e-01},prob(percent)="89",prob+-sd="89+-0"]:8.608432e-03[&length_mean=9.17417232e-03,length_median=8.60843200e-03,length_95%HPD={9.63025300e-04,1.76214400e-02}],(32[&prob=1.00000000e+00,prob_stddev=0.00000000e+00,prob_range={1.00000000e+00,1.00000000e+00},prob(percent)="100",prob+-sd="100+-0"]:2.378955e-02[&length_mean=2.43341817e-02,length_median=2.37895500e-02,length_95%HPD={1.41406300e-02,3.53434200e-02}],33[&prob=1.00000000e+00,prob_stddev=0.00000000e+00,prob_range={1.00000000e+00,1.00000000e+00},prob(percent)="100",prob+-sd="100+-0"]:3.719983e-02[&length_mean=3.75733780e-02,length_median=3.71998300e-02,length_95%HPD={2.55817100e-02,5.07101200e-02}])[&prob=1.00000000e+00,prob_stddev=0.00000000e+00,prob_range={1.00000000e+00,1.00000000e+00},prob(percent)="100",prob+-sd="100+-0"]:2.023478e-02[&length_mean=2.07317295e-02,length_median=2.02347800e-02,length_95%HPD={8.14819600e-03,3.23764500e-02}])[&prob=1.00000000e+00,prob_stddev=0.00000000e+00,prob_range={1.00000000e+00,1.00000000e+00},prob(percent)="100",prob+-sd="100+-0"]:8.122118e-02[&length_mean=8.20944040e-02,length_median=8.12211800e-02,length_95%HPD={5.88724600e-02,1.05886100e-01}],((38[&prob=1.00000000e+00,prob_stddev=0.00000000e+00,prob_range={1.00000000e+00,1.00000000e+00},prob(percent)="100",prob+-sd="100+-0"]:2.060942e-02[&length_mean=2.14619636e-02,length_median=2.06094200e-02,length_95%HPD={1.12924000e-02,3.51729700e-02}],39[&prob=1.00000000e+00,prob_stddev=0.00000000e+00,prob_range={1.00000000e+00,1.00000000e+00},prob(percent)="100",prob+-sd="100+-0"]:1.603244e-02[&length_mean=1.63208920e-02,length_median=1.60324400e-02,length_95%HPD={5.85055300e-03,2.69166900e-02}])[&prob=1.00000000e+00,prob_stddev=0.00000000e+00,prob_range={1.00000000e+00,1.00000000e+00},prob(percent)="100",prob+-sd="100+-0"]:6.497420e-02[&length_mean=6.53106541e-02,length_median=6.49742000e-02,length_95%HPD={4.37979300e-02,8.71179000e-02}],70[&prob=1.00000000e+00,prob_stddev=0.00000000e+00,prob_range={1.00000000e+00,1.00000000e+00},prob(percent)="100",prob+-sd="100+-0"]:1.719131e-01[&length_mean=1.73607225e-01,length_median=1.71913100e-01,length_95%HPD={1.37337000e-01,2.14238300e-01}])[&prob=1.00000000e+00,prob_stddev=0.00000000e+00,prob_range={1.00000000e+00,1.00000000e+00},prob(percent)="100",prob+-sd="100+-0"]:3.177895e-02[&length_mean=3.23352555e-02,length_median=3.17789500e-02,length_95%HPD={1.47772400e-02,5.05815800e-02}])[&prob=8.40878828e-01,prob_stddev=5.74347718e-02,prob_range={8.00266312e-01,8.81491345e-01},prob(percent)="84",prob+-sd="84+-6"]:1.648376e-02[&length_mean=1.71803322e-02,length_median=1.64837600e-02,length_95%HPD={3.58273100e-03,3.09385200e-02}],((((((40[&prob=1.00000000e+00,prob_stddev=0.00000000e+00,prob_range={1.00000000e+00,1.00000000e+00},prob(percent)="100",prob+-sd="100+-0"]:2.673476e-02[&length_mean=2.70929900e-02,length_median=2.67347600e-02,length_95%HPD={1.72894600e-02,3.83300000e-02}],((41[&prob=1.00000000e+00,prob_stddev=0.00000000e+00,prob_range={1.00000000e+00,1.00000000e+00},prob(percent)="100",prob+-sd="100+-0"]:6.915760e-03[&length_mean=7.58999359e-03,length_median=6.91576000e-03,length_95%HPD={7.00817100e-04,1.52679300e-02}],48[&prob=1.00000000e+00,prob_stddev=0.00000000e+00,prob_range={1.00000000e+00,1.00000000e+00},prob(percent)="100",prob+-sd="100+-0"]:2.653575e-02[&length_mean=2.69489567e-02,length_median=2.65357500e-02,length_95%HPD={1.24420300e-02,4.05425400e-02}])[&prob=1.00000000e+00,prob_stddev=0.00000000e+00,prob_range={1.00000000e+00,1.00000000e+00},prob(percent)="100",prob+-sd="100+-0"]:1.159797e-02[&length_mean=1.20302980e-02,length_median=1.15979700e-02,length_95%HPD={3.21086000e-03,2.19150800e-02}],47[&prob=1.00000000e+00,prob_stddev=0.00000000e+00,prob_range={1.00000000e+00,1.00000000e+00},prob(percent)="100",prob+-sd="100+-0"]:4.599138e-02[&length_mean=4.63471424e-02,length_median=4.59913800e-02,length_95%HPD={3.15653900e-02,5.95021900e-02}])[&prob=1.00000000e+00,prob_stddev=0.00000000e+00,prob_range={1.00000000e+00,1.00000000e+00},prob(percent)="100",prob+-sd="100+-0"]:9.979389e-03[&length_mean=1.02630741e-02,length_median=9.97938900e-03,length_95%HPD={3.67926100e-03,1.70111600e-02}])[&prob=7.42343542e-01,prob_stddev=1.60064118e-02,prob_range={7.31025300e-01,7.53661784e-01},prob(percent)="74",prob+-sd="74+-2"]:2.903136e-03[&length_mean=3.34865017e-03,length_median=2.90313600e-03,length_95%HPD={7.72519200e-06,7.44741400e-03}],(42[&prob=1.00000000e+00,prob_stddev=0.00000000e+00,prob_range={1.00000000e+00,1.00000000e+00},prob(percent)="100",prob+-sd="100+-0"]:1.780797e-02[&length_mean=1.83504207e-02,length_median=1.78079700e-02,length_95%HPD={9.99406500e-03,2.77087000e-02}],((43[&prob=1.00000000e+00,prob_stddev=0.00000000e+00,prob_range={1.00000000e+00,1.00000000e+00},prob(percent)="100",prob+-sd="100+-0"]:1.958791e-02[&length_mean=2.04971652e-02,length_median=1.95879100e-02,length_95%HPD={8.87575400e-03,3.52957500e-02}],44[&prob=1.00000000e+00,prob_stddev=0.00000000e+00,prob_range={1.00000000e+00,1.00000000e+00},prob(percent)="100",prob+-sd="100+-0"]:2.644404e-02[&length_mean=2.73696780e-02,length_median=2.64440400e-02,length_95%HPD={1.27493800e-02,4.29707100e-02}])[&prob=5.45938748e-01,prob_stddev=7.15580764e-02,prob_range={4.95339547e-01,5.96537949e-01},prob(percent)="55",prob+-sd="55+-7"]:3.466609e-03[&length_mean=4.26534559e-03,length_median=3.46660900e-03,length_95%HPD={1.43076400e-05,1.05163900e-02}],45[&prob=1.00000000e+00,prob_stddev=0.00000000e+00,prob_range={1.00000000e+00,1.00000000e+00},prob(percent)="100",prob+-sd="100+-0"]:2.729791e-02[&length_mean=2.77175240e-02,length_median=2.72979100e-02,length_95%HPD={1.63770400e-02,3.94546100e-02}])[&prob=7.78961385e-01,prob_stddev=8.66229346e-02,prob_range={7.17709720e-01,8.40213049e-01},prob(percent)="78",prob+-sd="78+-9"]:4.837891e-03[&length_mean=5.30538478e-03,length_median=4.83789100e-03,length_95%HPD={5.50378400e-04,1.06554200e-02}])[&prob=1.00000000e+00,prob_stddev=0.00000000e+00,prob_range={1.00000000e+00,1.00000000e+00},prob(percent)="100",prob+-sd="100+-0"]:1.194556e-02[&length_mean=1.23447782e-02,length_median=1.19455600e-02,length_95%HPD={5.74973900e-03,2.01446200e-02}])[&prob=1.00000000e+00,prob_stddev=0.00000000e+00,prob_range={1.00000000e+00,1.00000000e+00},prob(percent)="100",prob+-sd="100+-0"]:1.452587e-02[&length_mean=1.49909812e-02,length_median=1.45258700e-02,length_95%HPD={5.66937600e-03,2.41160600e-02}],46[&prob=1.00000000e+00,prob_stddev=0.00000000e+00,prob_range={1.00000000e+00,1.00000000e+00},prob(percent)="100",prob+-sd="100+-0"]:3.251782e-02[&length_mean=3.34187949e-02,length_median=3.25178200e-02,length_95%HPD={2.05696700e-02,4.75048400e-02}])[&prob=1.00000000e+00,prob_stddev=0.00000000e+00,prob_range={1.00000000e+00,1.00000000e+00},prob(percent)="100",prob+-sd="100+-0"]:4.432970e-02[&length_mean=4.49016447e-02,length_median=4.43297000e-02,length_95%HPD={2.91908500e-02,6.32331300e-02}],(((61[&prob=1.00000000e+00,prob_stddev=0.00000000e+00,prob_range={1.00000000e+00,1.00000000e+00},prob(percent)="100",prob+-sd="100+-0"]:1.314929e-02[&length_mean=1.35101863e-02,length_median=1.31492900e-02,length_95%HPD={6.24955100e-03,2.08974200e-02}],62[&prob=1.00000000e+00,prob_stddev=0.00000000e+00,prob_range={1.00000000e+00,1.00000000e+00},prob(percent)="100",prob+-sd="100+-0"]:1.759835e-02[&length_mean=1.79674423e-02,length_median=1.75983500e-02,length_95%HPD={9.96421800e-03,2.69902500e-02}])[&prob=9.99334221e-01,prob_stddev=9.41553637e-04,prob_range={9.98668442e-01,1.00000000e+00},prob(percent)="100",prob+-sd="100+-0"]:1.063211e-02[&length_mean=1.11817448e-02,length_median=1.06321100e-02,length_95%HPD={4.71360500e-03,1.90296300e-02}],63[&prob=1.00000000e+00,prob_stddev=0.00000000e+00,prob_range={1.00000000e+00,1.00000000e+00},prob(percent)="100",prob+-sd="100+-0"]:2.326686e-02[&length_mean=2.36550338e-02,length_median=2.32668600e-02,length_95%HPD={1.43454900e-02,3.46501700e-02}])[&prob=1.00000000e+00,prob_stddev=0.00000000e+00,prob_range={1.00000000e+00,1.00000000e+00},prob(percent)="100",prob+-sd="100+-0"]:1.816000e-02[&length_mean=1.85079704e-02,length_median=1.81600000e-02,length_95%HPD={6.40807900e-03,3.01809600e-02}],((64[&prob=1.00000000e+00,prob_stddev=0.00000000e+00,prob_range={1.00000000e+00,1.00000000e+00},prob(percent)="100",prob+-sd="100+-0"]:9.063665e-03[&length_mean=9.37592292e-03,length_median=9.06366500e-03,length_95%HPD={3.64448700e-03,1.56251700e-02}],65[&prob=1.00000000e+00,prob_stddev=0.00000000e+00,prob_range={1.00000000e+00,1.00000000e+00},prob(percent)="100",prob+-sd="100+-0"]:1.191766e-02[&length_mean=1.23295462e-02,length_median=1.19176600e-02,length_95%HPD={6.06174000e-03,1.99149300e-02}])[&prob=9.97336884e-01,prob_stddev=1.88310727e-03,prob_range={9.96005326e-01,9.98668442e-01},prob(percent)="100",prob+-sd="100+-0"]:1.270041e-02[&length_mean=1.31935932e-02,length_median=1.27004100e-02,length_95%HPD={4.65633200e-03,2.33211400e-02}],((67[&prob=1.00000000e+00,prob_stddev=0.00000000e+00,prob_range={1.00000000e+00,1.00000000e+00},prob(percent)="100",prob+-sd="100+-0"]:1.363727e-02[&length_mean=1.40682707e-02,length_median=1.36372700e-02,length_95%HPD={2.27918200e-03,2.65106200e-02}],68[&prob=1.00000000e+00,prob_stddev=0.00000000e+00,prob_range={1.00000000e+00,1.00000000e+00},prob(percent)="100",prob+-sd="100+-0"]:4.838920e-02[&length_mean=4.96223237e-02,length_median=4.83892000e-02,length_95%HPD={2.77852100e-02,7.13343800e-02}])[&prob=1.00000000e+00,prob_stddev=0.00000000e+00,prob_range={1.00000000e+00,1.00000000e+00},prob(percent)="100",prob+-sd="100+-0"]:3.300156e-02[&length_mean=3.34087352e-02,length_median=3.30015600e-02,length_95%HPD={1.49001600e-02,5.12132000e-02}],69[&prob=1.00000000e+00,prob_stddev=0.00000000e+00,prob_range={1.00000000e+00,1.00000000e+00},prob(percent)="100",prob+-sd="100+-0"]:4.632166e-02[&length_mean=4.52295388e-02,length_median=4.63216600e-02,length_95%HPD={7.79332500e-03,7.25833900e-02}])[&prob=8.35552597e-01,prob_stddev=2.54219482e-02,prob_range={8.17576565e-01,8.53528628e-01},prob(percent)="84",prob+-sd="84+-3"]:1.771214e-02[&length_mean=1.84771746e-02,length_median=1.77121400e-02,length_95%HPD={6.81021200e-03,3.24877500e-02}])[&prob=6.17842876e-01,prob_stddev=3.76621455e-03,prob_range={6.15179760e-01,6.20505992e-01},prob(percent)="62",prob+-sd="62+-0"]:7.153614e-03[&length_mean=7.53039176e-03,length_median=7.15361400e-03,length_95%HPD={1.37686200e-03,1.46767400e-02}],66[&prob=1.00000000e+00,prob_stddev=0.00000000e+00,prob_range={1.00000000e+00,1.00000000e+00},prob(percent)="100",prob+-sd="100+-0"]:4.359140e-02[&length_mean=4.23767346e-02,length_median=4.35914000e-02,length_95%HPD={1.33420600e-03,6.72164100e-02}])[&prob=1.00000000e+00,prob_stddev=0.00000000e+00,prob_range={1.00000000e+00,1.00000000e+00},prob(percent)="100",prob+-sd="100+-0"]:6.824891e-02[&length_mean=6.88894182e-02,length_median=6.82489100e-02,length_95%HPD={4.75202400e-02,9.05382200e-02}])[&prob=1.00000000e+00,prob_stddev=0.00000000e+00,prob_range={1.00000000e+00,1.00000000e+00},prob(percent)="100",prob+-sd="100+-0"]:3.972602e-02[&length_mean=3.99083579e-02,length_median=3.97260200e-02,length_95%HPD={2.32165200e-02,5.64621600e-02}],(((((49[&prob=1.00000000e+00,prob_stddev=0.00000000e+00,prob_range={1.00000000e+00,1.00000000e+00},prob(percent)="100",prob+-sd="100+-0"]:2.258099e-02[&length_mean=2.30364520e-02,length_median=2.25809900e-02,length_95%HPD={1.34827200e-02,3.36648600e-02}],50[&prob=1.00000000e+00,prob_stddev=0.00000000e+00,prob_range={1.00000000e+00,1.00000000e+00},prob(percent)="100",prob+-sd="100+-0"]:1.768068e-02[&length_mean=1.78863102e-02,length_median=1.76806800e-02,length_95%HPD={9.11326900e-03,2.63755000e-02}])[&prob=9.98002663e-01,prob_stddev=2.82466091e-03,prob_range={9.96005326e-01,1.00000000e+00},prob(percent)="100",prob+-sd="100+-0"]:1.265297e-02[&length_mean=1.31969345e-02,length_median=1.26529700e-02,length_95%HPD={5.23584800e-03,2.25460500e-02}],51[&prob=1.00000000e+00,prob_stddev=0.00000000e+00,prob_range={1.00000000e+00,1.00000000e+00},prob(percent)="100",prob+-sd="100+-0"]:3.072480e-02[&length_mean=3.08565437e-02,length_median=3.07248000e-02,length_95%HPD={1.92009900e-02,4.26671500e-02}])[&prob=6.13848202e-01,prob_stddev=1.50648582e-02,prob_range={6.03195739e-01,6.24500666e-01},prob(percent)="61",prob+-sd="61+-2"]:4.882063e-03[&length_mean=5.32308435e-03,length_median=4.88206300e-03,length_95%HPD={3.49258700e-05,1.13444800e-02}],60[&prob=1.00000000e+00,prob_stddev=0.00000000e+00,prob_range={1.00000000e+00,1.00000000e+00},prob(percent)="100",prob+-sd="100+-0"]:2.673263e-02[&length_mean=2.64695444e-02,length_median=2.67326300e-02,length_95%HPD={5.81769100e-03,4.63420300e-02}])[&prob=5.93874834e-01,prob_stddev=3.76621455e-03,prob_range={5.91211718e-01,5.96537949e-01},prob(percent)="59",prob+-sd="59+-0"]:9.115606e-03[&length_mean=1.00081845e-02,length_median=9.11560600e-03,length_95%HPD={1.76921300e-05,2.15376600e-02}],52[&prob=1.00000000e+00,prob_stddev=0.00000000e+00,prob_range={1.00000000e+00,1.00000000e+00},prob(percent)="100",prob+-sd="100+-0"]:4.050134e-02[&length_mean=3.99534418e-02,length_median=4.05013400e-02,length_95%HPD={1.17790400e-02,6.53788500e-02}])[&prob=9.99334221e-01,prob_stddev=9.41553637e-04,prob_range={9.98668442e-01,1.00000000e+00},prob(percent)="100",prob+-sd="100+-0"]:2.097262e-02[&length_mean=2.13907507e-02,length_median=2.09726200e-02,length_95%HPD={8.53677600e-03,3.48106500e-02}],53[&prob=1.00000000e+00,prob_stddev=0.00000000e+00,prob_range={1.00000000e+00,1.00000000e+00},prob(percent)="100",prob+-sd="100+-0"]:6.566383e-02[&length_mean=6.56622751e-02,length_median=6.56638300e-02,length_95%HPD={4.71806100e-02,8.33724700e-02}])[&prob=1.00000000e+00,prob_stddev=0.00000000e+00,prob_range={1.00000000e+00,1.00000000e+00},prob(percent)="100",prob+-sd="100+-0"]:2.375236e-02[&length_mean=2.46088729e-02,length_median=2.37523600e-02,length_95%HPD={1.17471300e-02,3.97798500e-02}])[&prob=9.51398136e-01,prob_stddev=1.41233046e-02,prob_range={9.41411451e-01,9.61384820e-01},prob(percent)="95",prob+-sd="95+-1"]:1.753280e-02[&length_mean=1.83626799e-02,length_median=1.75328000e-02,length_95%HPD={6.67308100e-03,3.19278000e-02}],(((54[&prob=1.00000000e+00,prob_stddev=0.00000000e+00,prob_range={1.00000000e+00,1.00000000e+00},prob(percent)="100",prob+-sd="100+-0"]:1.153713e-02[&length_mean=1.17630198e-02,length_median=1.15371300e-02,length_95%HPD={5.95132200e-03,1.87362700e-02}],57[&prob=1.00000000e+00,prob_stddev=0.00000000e+00,prob_range={1.00000000e+00,1.00000000e+00},prob(percent)="100",prob+-sd="100+-0"]:1.854844e-02[&length_mean=1.88679686e-02,length_median=1.85484400e-02,length_95%HPD={1.04119300e-02,2.68676500e-02}])[&prob=9.97336884e-01,prob_stddev=1.88310727e-03,prob_range={9.96005326e-01,9.98668442e-01},prob(percent)="100",prob+-sd="100+-0"]:5.648124e-03[&length_mean=5.90286483e-03,length_median=5.64812400e-03,length_95%HPD={1.10586600e-03,1.07276500e-02}],(56[&prob=1.00000000e+00,prob_stddev=0.00000000e+00,prob_range={1.00000000e+00,1.00000000e+00},prob(percent)="100",prob+-sd="100+-0"]:1.343916e-02[&length_mean=1.42208712e-02,length_median=1.34391600e-02,length_95%HPD={4.11647800e-03,2.45513200e-02}],58[&prob=1.00000000e+00,prob_stddev=0.00000000e+00,prob_range={1.00000000e+00,1.00000000e+00},prob(percent)="100",prob+-sd="100+-0"]:2.199244e-02[&length_mean=2.27667268e-02,length_median=2.19924400e-02,length_95%HPD={1.16336800e-02,3.35618900e-02}])[&prob=9.92010652e-01,prob_stddev=1.88310727e-03,prob_range={9.90679095e-01,9.93342210e-01},prob(percent)="99",prob+-sd="99+-0"]:1.078418e-02[&length_mean=1.11037381e-02,length_median=1.07841800e-02,length_95%HPD={2.41371800e-03,1.93336700e-02}])[&prob=6.84420772e-01,prob_stddev=4.89607891e-02,prob_range={6.49800266e-01,7.19041278e-01},prob(percent)="68",prob+-sd="68+-5"]:4.484076e-03[&length_mean=4.86122197e-03,length_median=4.48407600e-03,length_95%HPD={5.36621100e-04,1.00238600e-02}],(55[&prob=1.00000000e+00,prob_stddev=0.00000000e+00,prob_range={1.00000000e+00,1.00000000e+00},prob(percent)="100",prob+-sd="100+-0"]:8.207810e-03[&length_mean=8.58416319e-03,length_median=8.20781000e-03,length_95%HPD={2.62770700e-03,1.50283100e-02}],59[&prob=1.00000000e+00,prob_stddev=0.00000000e+00,prob_range={1.00000000e+00,1.00000000e+00},prob(percent)="100",prob+-sd="100+-0"]:2.065937e-02[&length_mean=2.13159099e-02,length_median=2.06593700e-02,length_95%HPD={1.04851600e-02,3.32100400e-02}])[&prob=7.88948069e-01,prob_stddev=1.22401973e-02,prob_range={7.80292943e-01,7.97603196e-01},prob(percent)="79",prob+-sd="79+-1"]:3.509673e-03[&length_mean=3.98011151e-03,length_median=3.50967300e-03,length_95%HPD={6.44535300e-05,8.95608000e-03}])[&prob=1.00000000e+00,prob_stddev=0.00000000e+00,prob_range={1.00000000e+00,1.00000000e+00},prob(percent)="100",prob+-sd="100+-0"]:8.076277e-02[&length_mean=8.14798596e-02,length_median=8.07627700e-02,length_95%HPD={5.81303000e-02,1.05546800e-01}])[&prob=1.00000000e+00,prob_stddev=0.00000000e+00,prob_range={1.00000000e+00,1.00000000e+00},prob(percent)="100",prob+-sd="100+-0"]:6.545830e-02[&length_mean=6.54401268e-02,length_median=6.54583000e-02,length_95%HPD={4.16926700e-02,8.90858400e-02}])[&prob=7.50332889e-01,prob_stddev=1.22401973e-02,prob_range={7.41677763e-01,7.58988016e-01},prob(percent)="75",prob+-sd="75+-1"]:1.962927e-02[&length_mean=1.98152453e-02,length_median=1.96292700e-02,length_95%HPD={8.17211000e-06,3.89901900e-02}],(((34[&prob=1.00000000e+00,prob_stddev=0.00000000e+00,prob_range={1.00000000e+00,1.00000000e+00},prob(percent)="100",prob+-sd="100+-0"]:1.939562e-02[&length_mean=1.96636945e-02,length_median=1.93956200e-02,length_95%HPD={9.11934100e-03,3.14611900e-02}],35[&prob=1.00000000e+00,prob_stddev=0.00000000e+00,prob_range={1.00000000e+00,1.00000000e+00},prob(percent)="100",prob+-sd="100+-0"]:1.272801e-02[&length_mean=1.31060990e-02,length_median=1.27280100e-02,length_95%HPD={2.68432100e-03,2.29228300e-02}])[&prob=1.00000000e+00,prob_stddev=0.00000000e+00,prob_range={1.00000000e+00,1.00000000e+00},prob(percent)="100",prob+-sd="100+-0"]:3.535682e-02[&length_mean=3.55080303e-02,length_median=3.53568200e-02,length_95%HPD={2.01807900e-02,4.98484100e-02}],36[&prob=1.00000000e+00,prob_stddev=0.00000000e+00,prob_range={1.00000000e+00,1.00000000e+00},prob(percent)="100",prob+-sd="100+-0"]:2.023232e-02[&length_mean=2.04544023e-02,length_median=2.02323200e-02,length_95%HPD={9.78761600e-03,3.15353500e-02}])[&prob=1.00000000e+00,prob_stddev=0.00000000e+00,prob_range={1.00000000e+00,1.00000000e+00},prob(percent)="100",prob+-sd="100+-0"]:6.188490e-02[&length_mean=6.19225807e-02,length_median=6.18849000e-02,length_95%HPD={4.26259400e-02,8.26839200e-02}],37[&prob=1.00000000e+00,prob_stddev=0.00000000e+00,prob_range={1.00000000e+00,1.00000000e+00},prob(percent)="100",prob+-sd="100+-0"]:6.525969e-02[&length_mean=6.58782624e-02,length_median=6.52596900e-02,length_95%HPD={4.58214800e-02,8.48981000e-02}])[&prob=9.63382157e-01,prob_stddev=1.22401973e-02,prob_range={9.54727031e-01,9.72037284e-01},prob(percent)="96",prob+-sd="96+-1"]:2.094028e-02[&length_mean=2.14897292e-02,length_median=2.09402800e-02,length_95%HPD={7.45561400e-03,3.81331900e-02}],(((71[&prob=1.00000000e+00,prob_stddev=0.00000000e+00,prob_range={1.00000000e+00,1.00000000e+00},prob(percent)="100",prob+-sd="100+-0"]:5.870677e-03[&length_mean=6.12573045e-03,length_median=5.87067700e-03,length_95%HPD={9.34793600e-04,1.11186100e-02}],73[&prob=1.00000000e+00,prob_stddev=0.00000000e+00,prob_range={1.00000000e+00,1.00000000e+00},prob(percent)="100",prob+-sd="100+-0"]:7.308196e-03[&length_mean=7.35757042e-03,length_median=7.30819600e-03,length_95%HPD={2.30016400e-04,1.28923500e-02}])[&prob=6.76431425e-01,prob_stddev=1.50648582e-02,prob_range={6.65778961e-01,6.87083888e-01},prob(percent)="68",prob+-sd="68+-2"]:3.948678e-03[&length_mean=4.21300780e-03,length_median=3.94867800e-03,length_95%HPD={9.06643400e-05,8.73138200e-03}],72[&prob=1.00000000e+00,prob_stddev=0.00000000e+00,prob_range={1.00000000e+00,1.00000000e+00},prob(percent)="100",prob+-sd="100+-0"]:6.352636e-03[&length_mean=6.64609619e-03,length_median=6.35263600e-03,length_95%HPD={1.09018300e-03,1.23078500e-02}])[&prob=1.00000000e+00,prob_stddev=0.00000000e+00,prob_range={1.00000000e+00,1.00000000e+00},prob(percent)="100",prob+-sd="100+-0"]:5.636880e-02[&length_mean=5.69258821e-02,length_median=5.63688000e-02,length_95%HPD={2.84005500e-02,8.84384400e-02}],(74[&prob=1.00000000e+00,prob_stddev=0.00000000e+00,prob_range={1.00000000e+00,1.00000000e+00},prob(percent)="100",prob+-sd="100+-0"]:3.703553e-03[&length_mean=4.05552211e-03,length_median=3.70355300e-03,length_95%HPD={4.10267600e-04,8.59765100e-03}],(75[&prob=1.00000000e+00,prob_stddev=0.00000000e+00,prob_range={1.00000000e+00,1.00000000e+00},prob(percent)="100",prob+-sd="100+-0"]:6.007371e-04[&length_mean=8.23642438e-04,length_median=6.00737100e-04,length_95%HPD={6.30851800e-07,2.52864200e-03}],76[&prob=1.00000000e+00,prob_stddev=0.00000000e+00,prob_range={1.00000000e+00,1.00000000e+00},prob(percent)="100",prob+-sd="100+-0"]:5.696091e-04[&length_mean=8.45733960e-04,length_median=5.69609100e-04,length_95%HPD={4.49880900e-07,2.54643500e-03}])[&prob=9.97336884e-01,prob_stddev=0.00000000e+00,prob_range={9.97336884e-01,9.97336884e-01},prob(percent)="100",prob+-sd="100+-0"]:5.733514e-03[&length_mean=5.97671091e-03,length_median=5.73351400e-03,length_95%HPD={1.27897500e-03,1.11582400e-02}])[&prob=1.00000000e+00,prob_stddev=0.00000000e+00,prob_range={1.00000000e+00,1.00000000e+00},prob(percent)="100",prob+-sd="100+-0"]:1.213711e-01[&length_mean=1.21837154e-01,length_median=1.21371100e-01,length_95%HPD={8.74291900e-02,1.57813400e-01}])[&prob=1.00000000e+00,prob_stddev=0.00000000e+00,prob_range={1.00000000e+00,1.00000000e+00},prob(percent)="100",prob+-sd="100+-0"]:3.455895e-01[&length_mean=3.46507505e-01,length_median=3.45589500e-01,length_95%HPD={2.76643900e-01,4.08082900e-01}])[&prob=1.00000000e+00,prob_stddev=0.00000000e+00,prob_range={1.00000000e+00,1.00000000e+00},prob(percent)="100",prob+-sd="100+-0"]:6.157974e-02[&length_mean=6.14656216e-02,length_median=6.15797400e-02,length_95%HPD={3.81066400e-02,8.51653900e-02}])[&prob=9.99334221e-01,prob_stddev=9.41553637e-04,prob_range={9.98668442e-01,1.00000000e+00},prob(percent)="100",prob+-sd="100+-0"]:1.792929e-02[&length_mean=1.81695097e-02,length_median=1.79292900e-02,length_95%HPD={7.14728700e-03,3.01590600e-02}])[&prob=5.22636485e-01,prob_stddev=4.23699137e-02,prob_range={4.92676431e-01,5.52596538e-01},prob(percent)="52",prob+-sd="52+-4"]:1.143581e-02[&length_mean=1.14469899e-02,length_median=1.14358100e-02,length_95%HPD={1.05933100e-04,2.07809700e-02}],29[&prob=1.00000000e+00,prob_stddev=0.00000000e+00,prob_range={1.00000000e+00,1.00000000e+00},prob(percent)="100",prob+-sd="100+-0"]:5.753310e-02[&length_mean=4.94147418e-02,length_median=5.75331000e-02,length_95%HPD={7.51310400e-06,8.51160100e-02}])[&prob=5.95872170e-01,prob_stddev=2.91881627e-02,prob_range={5.75233023e-01,6.16511318e-01},prob(percent)="60",prob+-sd="60+-3"]:1.406031e-02[&length_mean=1.46156312e-02,length_median=1.40603100e-02,length_95%HPD={4.35964400e-03,2.48105100e-02}],((25[&prob=1.00000000e+00,prob_stddev=0.00000000e+00,prob_range={1.00000000e+00,1.00000000e+00},prob(percent)="100",prob+-sd="100+-0"]:1.400079e-02[&length_mean=1.43757397e-02,length_median=1.40007900e-02,length_95%HPD={6.89771200e-03,2.17665600e-02}],26[&prob=1.00000000e+00,prob_stddev=0.00000000e+00,prob_range={1.00000000e+00,1.00000000e+00},prob(percent)="100",prob+-sd="100+-0"]:5.915147e-03[&length_mean=6.42710594e-03,length_median=5.91514700e-03,length_95%HPD={1.36490900e-03,1.19476000e-02}])[&prob=1.00000000e+00,prob_stddev=0.00000000e+00,prob_range={1.00000000e+00,1.00000000e+00},prob(percent)="100",prob+-sd="100+-0"]:3.473362e-02[&length_mean=3.51869477e-02,length_median=3.47336200e-02,length_95%HPD={1.98824100e-02,5.11405400e-02}],(27[&prob=1.00000000e+00,prob_stddev=0.00000000e+00,prob_range={1.00000000e+00,1.00000000e+00},prob(percent)="100",prob+-sd="100+-0"]:4.225454e-03[&length_mean=4.81951856e-03,length_median=4.22545400e-03,length_95%HPD={1.81201400e-04,1.16414600e-02}],28[&prob=1.00000000e+00,prob_stddev=0.00000000e+00,prob_range={1.00000000e+00,1.00000000e+00},prob(percent)="100",prob+-sd="100+-0"]:2.455302e-03[&length_mean=3.25504000e-03,length_median=2.45530200e-03,length_95%HPD={8.78745600e-07,9.18195800e-03}])[&prob=9.70705726e-01,prob_stddev=1.12986436e-02,prob_range={9.62716378e-01,9.78695073e-01},prob(percent)="97",prob+-sd="97+-1"]:6.701349e-02[&length_mean=6.18968862e-02,length_median=6.70134900e-02,length_95%HPD={7.84137100e-06,1.02241000e-01}])[&prob=5.91211718e-01,prob_stddev=3.38959309e-02,prob_range={5.67243675e-01,6.15179760e-01},prob(percent)="59",prob+-sd="59+-3"]:1.257354e-02[&length_mean=1.32945143e-02,length_median=1.25735400e-02,length_95%HPD={2.83870300e-03,2.59757800e-02}])[&prob=1.00000000e+00,prob_stddev=0.00000000e+00,prob_range={1.00000000e+00,1.00000000e+00},prob(percent)="100",prob+-sd="100+-0"]:3.886065e-02[&length_mean=3.90264002e-02,length_median=3.88606500e-02,length_95%HPD={2.53338400e-02,5.31653600e-02}])[&prob=7.01065246e-01,prob_stddev=2.16557336e-02,prob_range={6.85752330e-01,7.16378162e-01},prob(percent)="70",prob+-sd="70+-2"]:5.460506e-03[&length_mean=5.84921949e-03,length_median=5.46050600e-03,length_95%HPD={5.16702100e-04,1.16023900e-02}])[&prob=1.00000000e+00,prob_stddev=0.00000000e+00,prob_range={1.00000000e+00,1.00000000e+00},prob(percent)="100",prob+-sd="100+-0"]:6.956637e-03[&length_mean=7.36688232e-03,length_median=6.95663700e-03,length_95%HPD={2.63693700e-03,1.41424100e-02}])[&prob=1.00000000e+00,prob_stddev=0.00000000e+00,prob_range={1.00000000e+00,1.00000000e+00},prob(percent)="100",prob+-sd="100+-0"]:5.497941e-03[&length_mean=5.75782581e-03,length_median=5.49794100e-03,length_95%HPD={1.59385700e-03,1.01630900e-02}])[&prob=7.74966711e-01,prob_stddev=5.64932182e-03,prob_range={7.70972037e-01,7.78961385e-01},prob(percent)="77",prob+-sd="77+-1"]:2.209693e-03[&length_mean=2.52896406e-03,length_median=2.20969300e-03,length_95%HPD={8.21750000e-05,5.78358700e-03}],8[&prob=1.00000000e+00,prob_stddev=0.00000000e+00,prob_range={1.00000000e+00,1.00000000e+00},prob(percent)="100",prob+-sd="100+-0"]:4.455901e-03[&length_mean=4.83177145e-03,length_median=4.45590100e-03,length_95%HPD={9.69095600e-04,8.93383000e-03}])[&prob=9.84021305e-01,prob_stddev=1.88310727e-03,prob_range={9.82689747e-01,9.85352863e-01},prob(percent)="98",prob+-sd="98+-0"]:3.898368e-03[&length_mean=4.22143612e-03,length_median=3.89836800e-03,length_95%HPD={3.69323900e-04,8.37373700e-03}])[&prob=5.98535286e-01,prob_stddev=4.70776818e-03,prob_range={5.95206391e-01,6.01864181e-01},prob(percent)="60",prob+-sd="60+-0"]:1.169201e-03[&length_mean=1.50432082e-03,length_median=1.16920100e-03,length_95%HPD={3.54854300e-06,3.84315300e-03}]);

end;
