# Supplementary material for: Three new species of arbuscular mycorrhizal fungi (Glomeromycota) and Acaulospora gedanensis revised
Source: Front Microbiol. 2024 Feb 12;15:1320014. doi: 10.3389/fmicb.2024.1320014 (PMC10896085; doi:10.3389/fmicb.2024.1320014)
Supplement: Supplementary Table 13 — Data obtained from a RAxML-NG analysis of 45S sequences (see Figure 7). [file Table_13.DOCX]

((((JX135571_Acaulospora_herrerae:0.045704,(((Acaulospora_mendoncae_OK392597:0.022773,MN081001_Acaulospora_aspera:0.017796)81:0.012902,HG422734_Acaulospora_spinosissima:0.031663)46:0.002236,KM057074_Acaulospora_excavata:0.029954)77:0.007550)92:0.020030,FM876830_Acaulospora_kentinensis:0.067995)98:0.023378,(((((KY362428_Acaulospora_saccata:0.047581,(LN884303_Acaulospora_papillosa:0.006289,AJ242500_Acaulospora_morrowiae:0.027254)90:0.012286)84:0.009704,Acaulospora_flavopapillosa_OK360960:0.027572)72:0.002025,(((AM040291_Acaulospora_longula:0.028122,MT832212_Acaulospora_dilatata:0.020419)36:0.002045,LN881566_Acaulospora_rugosa:0.028330)52:0.003823,JF439093_Acaulospora_delicata:0.018335)94:0.012279)97:0.014408,KY362433_Acaulospora_fragilissima:0.034075)100:0.045854,(((HE603644_Acaulospora_nivalis:0.023400,(LN811001_Acaulospora_baetica:0.013171,KP191472_Acaulospora_ignota:0.017802)79:0.011147)89:0.018252,KY413814_Acaulospora_spinulifera:0.049705)30:0.003862,((AJ239115_Acaulospora_denticulata:0.051677,(AJ891119_Acaulospora_paulinae:0.011858,AM076382_Acaulospora_sieverdingii:0.052824)96:0.032852)56:0.019060,(FR846385_Acaulospora_punctata:0.011737,FM876788_Acaulospora_cavernata:0.008912)87:0.013746)31:0.006297)100:0.073782)99:0.041627)70:0.017844,((((KP756456_Acaulospora_mellea:0.027424,KY565429_Acaulospora_koreana:0.013345)71:0.007781,(KP756584_Acaulospora_lacunosa:0.024415,LN736022_Acaulospora_foveata:0.038763)98:0.020187)100:0.085591,(FR750063_Acaulospora_colombiana:0.187908,(Acaulospora_intravesiculata_OL661628:0.021418,KP191475_Acaulospora_koskei:0.015661)100:0.068183)77:0.031581)46:0.016714,(((KX345938_Sacculospora_felinovii:0.003042,(KX345939_Sacculospora_felinovii:0.000001,KX345941_Sacculospora_felinovii:0.000001)100:0.005670)100:0.132233,((KX355819_Sacculospora_baltica:0.005739,KX355818_Sacculospora_baltica:0.007521)51:0.003372,KX355821_Sacculospora_baltica:0.005118)94:0.054413)100:0.401221,(((((FR681930_Acaulospora_alpina:0.004979,FR681927_Acaulospora_alpina:0.007716)100:0.026999,MH045498_Acaulospora_tsugae:0.070947)49:0.005524,(((HF567933_Acaulospora_tortuosa:0.014238,HF567936_Acaulospora_tortuosa:0.005702)100:0.034105,(GU326346_Acaulospora_colliculosa:0.003713,GU326352_Acaulospora_colliculosa:0.000803)100:0.084748)71:0.012649,(((((Acaulospora_gedanensis_6_SSU_ITS_LSU_5_08_2022:0.003312,(Acaulospora_gedanensis_2_SSU_ITS_LSU_5_09_2022:0.003065,Acaulospora_gedanensis_3_SSU_ITS_LSU_5_09_2022:0.000001)86:0.002037)10:0.000001,((Acaulospora_gedanensis_3_SSU_ITS_LSU_12_09_2022:0.005033,Acaulospora_gedanensis_5_SSU_ITS_LSU_5_08_2022:0.000828)27:0.000001,Acaulospora_gedanensis_1_SSU_ITS_LSU_5_09_2022:0.001656)64:0.000826)33:0.000838,(Acaulospora_gedanensis_4_SSU_ITS_LSU_5_08_2022:0.000822,Acaulospora_gedanensis_1_SSU_ITS_LSU_5_08_2022:0.000829)60:0.000838)88:0.008612,(((Acaulospora_brasiliensis_FN825903:0.004001,Acaulospora_brasiliensis_FN825906:0.001218)50:0.000864,((Acaulospora_brasiliensis_FN825907:0.004445,(((Acaulospora_brasiliensis_FN825909:0.000001,Acaulospora_brasiliensis_FN825905:0.000001)89:0.000996,Acaulospora_brasiliensis_FN825904:0.000001)33:0.000998,Acaulospora_brasiliensis_FN825902:0.000001)24:0.000001)52:0.003423,Acaulospora_brasiliensis_FN825908:0.003497)43:0.001689)75:0.004797,(Acaulospora_brasiliensis_FN825910:0.005148,Acaulospora_brasiliensis_FN825911:0.003500)99:0.004587)97:0.006972)59:0.003970,(HF567941_Acaulospora_pustulata:0.010594,(HF567938_Acaulospora_pustulata:0.005982,HF567939_Acaulospora_pustulata:0.004174)93:0.002911)100:0.020702)99:0.038953)75:0.014277)92:0.017406,Acaulospora_fanjing_MW723431:0.036337)99:0.068986,((FR750173_Acaulospora_entreriana:0.019486,(Acaulospora_laevis_FN547516:0.012178,AF133764_Acaulospora_colossica:0.018735)100:0.036620)100:0.064935,HG421736_Acaulospora_viridis:0.067239)70:0.021923)32:0.019084)46:0.002867)100:0.071460,((FR692354_Acaulospora_scrobiculata:0.007890,KM057063_Acaulospora_reducta:0.020983)57:0.002219,((FR821674_Acaulospora_minuta:0.011313,FR869691_Acaulospora_minuta:0.018984)90:0.005293,(FJ461799_Acaulospora_tuberculata:0.013268,FR750152_Acaulospora_spinosa:0.022478)87:0.011314)30:0.003848)100:0.084386);
